# Systematic Modeling, Prediction, and Comparison of Domain–Peptide Affinities: Does it Work Effectively With the Peptide QSAR Methodology?

**DOI:** 10.3389/fgene.2021.800857

**Published:** 2022-01-14

**Authors:** Qian Liu, Jing Lin, Li Wen, Shaozhou Wang, Peng Zhou, Li Mei, Shuyong Shang

**Affiliations:** ^1^ Center for Informational Biology, School of Life Science and Technology, University of Electronic Science and Technology of China (UESTC), Chengdu, China; ^2^ Institute of Culinary, Sichuan Tourism University, Chengdu, China; ^3^ Institute of Ecological Environment Protection, Chengdu Normal University, Chengdu, China

**Keywords:** computational peptidology, peptide quantitative structure-activity relationship, domain-peptide interaction, amino acid descriptor, statistical modeling, machine learning

## Abstract

The protein–protein association in cellular signaling networks (CSNs) often acts as weak, transient, and reversible domain–peptide interaction (DPI), in which a flexible peptide segment on the surface of one protein is recognized and bound by a rigid peptide-recognition domain from another. Reliable modeling and accurate prediction of DPI binding affinities would help to ascertain the diverse biological events involved in CSNs and benefit our understanding of various biological implications underlying DPIs. Traditionally, peptide quantitative structure-activity relationship (pQSAR) has been widely used to model and predict the biological activity of oligopeptides, which employs amino acid descriptors (AADs) to characterize peptide structures at sequence level and then statistically correlate the resulting descriptor vector with observed activity data *via* regression. However, the QSAR has not yet been widely applied to treat the direct binding behavior of large-scale peptide ligands to their protein receptors. In this work, we attempted to clarify whether the pQSAR methodology can work effectively for modeling and predicting DPI affinities in a high-throughput manner? Over twenty thousand short linear motif (SLiM)-containing peptide segments involved in SH3, PDZ and 14-3-3 domain-medicated CSNs were compiled to define a comprehensive sequence-based data set of DPI affinities, which were represented by the Boehringer light units (BLUs) derived from previous arbitrary light intensity assays following SPOT peptide synthesis. Four sophisticated MLMs (MLMs) were then utilized to perform pQSAR modeling on the set described with different AADs to systematically create a variety of linear and nonlinear predictors, and then verified by rigorous statistical test. It is revealed that the genome-wide DPI events can only be modeled qualitatively or semiquantitatively with traditional pQSAR strategy due to the intrinsic disorder of peptide conformation and the potential interplay between different peptide residues. In addition, the arbitrary BLUs used to characterize DPI affinity values were measured *via* an indirect approach, which may not very reliable and may involve strong noise, thus leading to a considerable bias in the modeling. The *R*_*prd*_^2^ = 0.7 can be considered as the upper limit of external generalization ability of the pQSAR methodology working on large-scale DPI affinity data.

## 1 Introduction

Protein–protein interactions play a key role in cell life. Through formation of the functionally complicated complexes between two or more interacting protein partners, they participate in a variety of signal cascades in cells, thereby regulating the life activities of cells and individuals (Slater et al., 2020). In cell signaling network, intrinsically disordered proteins (IDP) often interacts specifically with the peptide-recognition domain of target protein through a flexible peptide segment on its own surface (Dyson and Wright 2005). In this way, flexible peptides tend to spontaneously fold into regular secondary structures, and then the specific recognition and interaction between peptide-recognition domains (PRDs) and flexible peptides were created in a folding-on-binding or binding-on-folding manner (Dyson and Wright 2002). Different from the permanent and stable complexes that are commonly formed by binding with global rigid globulins, the domain–peptide complexes are generally transient and reversible due to the limited number of residues and small contact area involved in the complex interfaces. This feature makes domain–peptide interactions (DPIs) very suitable to serve as molecular switches in biological signaling pathways that require exquisitely dynamic regulation and are closely related to various cellular processes and major diseases.

Although the high-throughput synthesis techniques such as combinatorial library, phage display and peptide microarray have considerably promoted DPI discovery over the past decades (Engelmann et al., 2014; Gray and Brown 2014; Zambrano-Mila et al., 2020), it is still time-consuming and expensive to practice full systematic screening against all potential peptide segment candidates in the human genome. In addition, a variety of peptide-recognition domains existed in cells also largely intensify the challenge of systematic screening. To tackle this issue, we previously suggested the *computational peptidology* as a new and attractive area to rationally investigate and design bioactive peptides or peptidic agents with *in silico* assistance (Zhou et al., 2013), in which the peptide quantitative structure-activity relationship (pQSAR) is one of the most widely used strategies to model the statistical correlation between peptide structure and biological activity (or toxicity, efficacy and potency) at sequence level (Zhou et al., 2008a). Machine learning has been widely used to perform the pQSAR modeling, but most of previous studies were focused on specific domains and/or limited samples, and thus unable to systematically evaluate the feasibility and applicability of pQSAR methodology in predicting DPI affinities. For example, Hou et al. deployed a series of works to characterize the 3D-structurally physiochemical properties of peptide binding to SH3 domain by using dynamics simulation, molecular field analysis and interaction energy component decomposition, and then they employed support vector machine (SVM) to create the pQSAR relationship between the characterized property parameters and measured DPI affinities (Hou et al., 2006; Hou et al., 2008; Hou et al., 2009). Jin et al. used random forest (RF) to perform structure-based pQSAR study of DPI binding behavior by dissecting residue interaction profile at the complex interface of PDZ domain with its peptide ligands (Jin et al., 2013). We also proposed the Gaussian process (GP) as a promising machine learning approach to predict the binding affinities and biological activities of diverse peptides against different proteins and domains (Zhou et al., 2008b; Zhou et al., 2010).

The key to the development of rapid pQSAR virtual screening technology for genome-wide DPIs is the characterization of interaction binding behavior and the construction of multivariate statistical model. The former parameterizes the sequence, structural, physicochemical and/or energetic properties of DPIs into a set of multidimensional numerical vectors that can be readily processed in computer, and the latter generates a regression relationship by statistically associating the vector set with corresponding DPI affinities with supervised machine learning approach. Recently, we have given a systematic review on the application of machine learning methods (MLMs) to quantitative DPI affinity prediction and its implications for therapeutic peptide design (Li et al., 2019), in which we pointed out that, although a number of pQSAR works have been reported to address the DPI affinity prediction problem, there was no comprehensive evaluation and systematic comparison of the pQSAR modeling performance between the different combinations of peptide-recognition domain types, MLMs and structural characterization strategies, thus lacking a general conclusion for the applicability of pQSAR methodology in DPI affinity modeling and prediction. In this study, we attempted to create, examine and compare a variety of pQSAR predictors built with PLS, SVM, RF and GP on >20,000 SLiM peptides involved in SH3, PDZ and 14-3-3 domain-medicated cell signaling networks. These peptide structures were characterized at traditional sequence level using classical amino acid descriptors (AADs) and their affinities were determined consistently by SPOT peptide syntheses and arbitrary light intensity assays. This work would shed light on the general purpose of pQSAR-based DPI affinity modeling and prediction.

## 2 Materials and Methods

### 2.1 Four Machine Learning Methods That Have Ever Been Applied in Peptide Quantitative Structure-Activity Relationship

Four sophisticated MLMs that have ever been applied in the pQSAR study of DPIs and other protein–peptide binding phenomena were considered in this work, including one linear partial least squares (PLS) and three nonlinear support vector machine (SVM), random forest (RF) and Gaussian process (GP) (Geladi and Kowalski 1986; Cortes and Vapnik 1995; Breiman 2001; Obrezanova et al., 2007). The PLS is a widely used multivariate statistical technique in the QSAR community, which has been intrinsically integrated into the famous 3D-QSAR methods of comparative molecular field analysis (CoMFA) and comparative molecular similarity indices (CoMSIA) as standard modeling tool to perform pQSAR analysis of SH3–peptide interactions at molecular field level (Hou et al., 2006). The method provides a multi-dependent variable to multi-independent variable regression, which can better deal with the problems difficult to be solved by least square regression. The SVM has also been successfully employed to characterize the SH3- and PDZ-mediated DPIs involved in the human genome (Hou et al., 2008, Hou et al., 2009; Li et al., 2011). The method converts quadratic convex programming problem into the corresponding duality problem for solving by Lagrange multiplier method, and constructs a series of kernel functions by using Mercer theorem to realize the high-dimensional inner product operation in the original space (Cortes and Vapnik 1995). In addition, the RF and GP were also introduced previously by our group to investigate DPIs (Zhou et al., 2008b) and other peptide-related issues such as enzyme-inhibitory activity (Zhou et al., 2010) and chromatographic retention behavior (Tian et al., 2009; Zhou et al., 2009). The former is an ensemble learning algorithm based on decision tree proposed by Breiman (2001), which also provides additional features such as variable importance and out-of-bag (OOB) validation that increase its utility for statistical modeling. The latter is based on the Bayesian non-parametric model that has a strict statistical learning theory basis and a strong generalization ability to adjust the model’s flexibility and achieve a certain transparency through so-call “hyperparameters” rather than conventional parameters to avoid fixed basis function in the traditional sense (Obrezanova et al., 2007).

The details of these machine learning modeling processes can be found in our previous publications (Rasmussen and Williams 2006). Briefly, the input variables were standardized by autoscaling for PLS and RF or [–1, +1] scaling for SVM and GP. The model parameters such as the number of latent variables (NLV) for PLS, and the *ε*-insensitive loss function, penalty factor (*C*) and kernel radial (*σ* ^2^) for SVM, the number of trees (*ntree*) and the optimal size of the variable subset (*mtry*) for RF and the hyperparameter set (*Θ*) for GP need to be determined before modeling, and we employed consistent strategies as summarized in Table 1 to optimize these parameters. Here, the PLS, SVM, RF and GP modeling and parameter optimization were carried out with in-house Matlab tool box ZP-explore (Zhou et al., 2009). In addition, the SVM regression was also carried out using the sophisticated LibSVM program (Chang and Lin 2011) for comparison purpose.

**TABLE 1:** Four MLMs used in this study.

MLM	Type	Variable standardization	Model parameter	
			Parameter	Optimization
PLS	Linear	Autoscaling	NLV: number of latent variables	Increase of cumulative cross-validation *q* ^2^ is below 0.097
SVM	Nonlinear	[–1, +1] scaling	ε: ε-insensitive loss function	Systematic grid search for minimizing cross-validation RMSE_cv_
			C: penalty factor	
			σ2: kernel radial	
RF	Nonlinear	[–1, +1] scaling	ntree: number of trees	Systematic grid search for minimizing cross-validation RMSE_cv_
			mtry: size of descriptor subset	
GP	Linear/nonlinear	Autoscaling	Θ: hyperparameter set	Automatic determination

### 2.2 Curation of Comprehensive Sequence-Based Domain–Peptide Interaction Data Set With a Consistent Affinity Expression

A variety of peptide-recognition domains that can specifically recognize and interact with diverse short linear motifs (SLiMs) on their partner protein surfaces have been discovered over the past decades (Kuriyan and Cowburn 1997), including but not limited to SH3, SH2, WW, PDZ, PTB, 14-3-3, EH, GYF, PH, EVH1, UEV, VHS, FHA, WD40 and so on. Here, we mainly selected three most common domain categories with considerably different SLiM properties but highly consistent affinity data for this study, namely, SH3, PDZ and 14-3-3; they can be further divided into different subtypes in terms of their parent proteins. The SH3 domain was first identified in the non-receptor tyrosine kinase *c*-Src and can specifically binds PxxP-containing polyproline-II (PPII) helix peptide segments (Li et al., 2005). The PDZ domain targets the C-terminal free peptide segments of substrate proteins with a plastic pattern (Ivarsson 2012). The 14-3-3 domain has been widely found in hundreds of signaling proteins to mediate protein–protein interactions by recognizing peptide segments of phosphoserine or phosphothreonine residues (Aitken et al., 1995).

Here, we curated totally 21,704 SLiM-containing peptides that separately target ten SH3 domains, seven PDZ domains and one 14-3-3 domain from previous reports (Boisguerin et al., 2004; Landgraf et al., 2004; Vouilleme et al., 2010; Panni et al., 2011) to define a comprehensive sequence-based DPI affinity data set consisting of 18 panels. These peptides were produced using SPOT peptide synthesis technology on cellulose membranes and then their binding affinities to different domains were consistently indicated by Boehringer light units (BLUs) derived from arbitrary light intensity assays (Volkmer et al., 2012). This protocol can fast yield various peptide candidates in a short time scale and test their domain binding in a high-throughput manner, and thus have been widely used to measure DPI affinities. By further excluding few invalid samples such as no binders or no affinity values, we consequently obtained 21,399 valid peptides; their information are summarized in Table 2, and their sequences and BLU values are tabulated in Supplementary Tables S1–S3.

**TABLE 2:** Summary of 21,704 SLiM-containing peptide samples binding to ten SH3, seven PDZ and one 14-3-3 domains.

Panel	Domain	Parent protein	Domain Number	Species	Peptide number
1	SH3	Amphiphysin	1/1	Human	884 Landgraf et al. (2004)
2		Amphyphisin	1/1	Yeast	2032 Landgraf et al. (2004)
3		Boi1	1/1	Yeast	1336 Landgraf et al. (2004)
4		Boi2	1/1	Yeast	1312 Landgraf et al. (2004)
5		Endophilin	1/1	Yeast	1998 Landgraf et al. (2004)
6		Myosin5	1/1	Yeast	1139 Landgraf et al. (2004)
7		Rvs167	1/1	Yeast	1369 Landgraf et al. (2004)
8		Sho1	1/1	Yeast	1015 Landgraf et al. (2004)
9		Yfr024	1/1	Yeast	1282 Landgraf et al. (2004)
10		Yhr016c	1/1	Yeast	1348 Landgraf et al. (2004)
11	PDZ	CALP	1/1	Human	80 Vouilleme et al. (2010)
12		NHERF1	1/2	Human	77 Vouilleme et al. (2010)
13		NHERF1	2/2	Human	80 Vouilleme et al. (2010)
14		NHERF2	1/2	Human	80 Vouilleme et al. (2010)
15		NHERF2	2/2	Human	80 Vouilleme et al. (2010)
16		SYNA1	1/1	Human	56 Vouilleme et al. (2010)
17		PSD95	1/1	Human	6068 Boisguerin et al. (2004)
18	14-3-3	14-3-3	1/1	Yeast	1163 Panni et al. (2011)

### 2.3 Statistical Verification of Peptide Quantitative Structure-Activity Relationship Models With Internal and External Validations

The built pQSAR predictive models should pass rigorous statistical test before practical applications to examine their effectiveness, illness and generalization ability. Here, we used a combination of internal and external validations to verify the statistical stability and predictive power of the models. Internal validation includes goodness-of-fit and 10-fold cross-validation on training set, while for the external validation we randomly divided each sample panel into ∼2/3 as a training set for building pQSAR model, and the remaining ∼1/3 as a test set for blind testing of the built model. In a highly cited paper, Golbraikh and Alexander (2002) pointed out that the internal validation is only a necessary but not sufficient condition to measure the reliability of a QSAR model, and the model predictability must be confirmed further through external validation.

### 2.4 Structural Characterization of Peptide Sequences Using Amino Acid Descriptors

Amino acid descriptors (AADs) are a classical approach to characterize peptide structure at sequence level, which utilize a *n*-dimensional vector to represent each of 20 amino acids and are commonly derived from a large number of original amino acid properties such as topological, physicochemical, 3D-structural and quantum-chemical, by using multivariate statistical techniques such as principal components analysis (PCA) and factor analysis (FA) (Zhou et al., 2008a). An *n*-mer peptide can be parameterized by in turn replacing its each amino acid residue to a corresponding *m*-dimensional AAD array, consequently resulting in *n* × *m* descriptors for the peptide, which define the independent variable space X and can be further correlated statistically with independent variable y (affinity) using machine learning regression. Recently, we have systematically evaluated totally 80 AADs in pQSAR modeling and identified a number of AADs with good performance (Zhou et al., 2021), from which we herein selected four different types of AADs to characterize the 21,399 SLiM-containing peptides listed in Table 2, including MolSurf (quantum-chemical) (Norinder and Svensson 1998), ST_scale (topological) (Yang et al., 2010), VHSE (physicochemical) (Mei et al., 2005) and VSGETAWAY (3D-structural) (Tong and Zhang 2007). Their values are tabulated in Supplementary Tables S4–S7.

## 3 Results and Discussion

There are several indicators that can be used to represent the binding affinity of DPIs, such as the *K*_d_ that can be determined by fluorescence polarization (FP) and surface plasmon resonance (SPR) to indicate the apparent dissociation constant for domain–peptide complex formation, and the ∆*G* that can be measured using isothermal titration calorimetry (ITC) to denote free energy change upon the complex binding. However, neither *K*_d_ nor ∆*G* can be obtained in a high-throughput manner, and thus they are not feasible for characterizing the large-scale DPI affinity data. In recent years, the SPOT peptide synthesis in conjunction with light intensity assays has been used to rapidly screen effective domain binders against massive peptide candidates, where peptides matching the defined patterns were synthesized at high density on cellulose membranes by SPOT synthesis technology and the membranes were probed with GST-fused domain protein, which were then revealed by an anti-GST antibody and by a secondary anti-IgG antibody coupled to horseradish peroxidase (POD) to derive the intensity of each SPOT quantitatively in Boehringer light unit (BLU) as an arbitrary light intensity unit (Landgraf et al., 2004). In this study, all DPI affinity data were expressed consistently as the BLU values collected from Refs (Boisguerin et al., 2004; Landgraf et al., 2004; Vouilleme et al., 2010; Panni et al., 2011).

### 3.1 Effect of Machine Learning Methods on Peptide Quantitative Structure-Activity Relationship Modeling

The PLSR, GP, RF, SVM and LibSVM regressions were employed to create three types of DPI affinity predictors for 18 DPI panels based on training samples, which were then used to blindly predict test samples (all resulting statistics are tabulated in the Supplementary Tables S8–S10). In order to compare different MLMs in the modeling and prediction of DPI affinities, we selected three kinds of samples binding separately to human amphyphisin SH3 (1/1), human SYNA1 PDZ (1/1) and yeast 14-3-3 (1/1) domains, and compared their fitting determination coefficient *R*_*fit*_^2^ on the training set, cross-validation determination coefficient *R*_*cv*_^2^ on the training set and predictive determination coefficient *R*_*prd*_^2^ on test set. As can be in Table 3, the performance of obtained pQSAR models varies considerably over MLMs and domain types. These models have high internal fitting ability but generally exhibit moderate or modest internal stability and external predictability, with *R*_*fit*_^2^ > 0.6 but *R*_*cv*_^2^ < 0.6 and *R*_*prd*_^2^ < 0.5. Among the three types of DPI affinity predictors the predictive power *R*_*prd*_^2^ of nonlinear GP, RF, SVM and LibSVM is generally better than that of linear PLS, suggesting that the DPI events are complicated dynamic process that involve many nonlinear factors, which can be better handled by nonlinear than linear methods. Even so, the modeling performance of both the linear and nonlinear methods is generally moderately, indicated by the high internal fitting ability but relatively low internal stability and predictability, imparting an overfitting phenomenon may exist in these regression models.

**TABLE 3:** Comparison of different MLMs on different DPI samples^a^.

MLM	DPI^b^	Training set				Test set	
		*R*_*fit*_^2c^	RMSE_fit_^*d*^	*R*_*cv*_^2*c*^	RMSE_cv_^*d*^	*R*_*prd*_^2*c*^	RMSE_prd_^*d*^
PLS	SH3	0.8641	0.4765	0.8335	0.5275	0.3072	0.5851
	PDZ	0.9312	0.1062	0.1077	0.3823	0.2263	0.3276
	14-3-3	0.4344	0.7048	0.3341	0.7687	0.3625	0.7446
GP	SH3	0.8668	0.4719	0.8349	0.5252	0.3147	0.5808
	PDZ	0.6984	0.2223	0.1953	0.3631	0.3391	0.3028
	14-3-3	0.4334	0.7091	0.3548	0.7566	0.3669	0.7420
RF	SH3	0.9470	0.2975	0.2074	1.1509	0.4973	0.4987
	PDZ	0.8191	0.1722	0.4005	0.3134	0.3824	0.2928
	14-3-3	0.8116	0.4088	0.2562	0.8124	0.3456	0.7715
SVM	SH3	0.8772	0.4530	0.8352	0.5248	0.3091	0.5843
	PDZ	0.7242	0.2126	0.1880	0.3647	0.2689	0.3594
	14-3-3	0.5211	0.6519	0.3614	0.7527	0.3886	0.7279
LibSVM	SH3	0.7008	0.2971	0.6817	0.3144	0.4254	0.3693
	PDZ	0.8778	0.0813	0.1295	0.1528	0.2766	0.1189
	14-3-3	0.4003	0.6085	0.3097	0.6702	0.3025	0.6342

a VHSE, descriptor was used to characterize peptide sequences.

b Human amphyphisin SH3 (1/1), human SYNA1 PDZ (1/1) and yeast 14-3-3 (1/1) are selected as case analysis.

c *R*_*fit*_^2^, *R*_*cv*_^2^ and *R*_*prd*_^2^ are the determination coefficients of internal fitting in training set, internal cross-validation on training set, and external blind prediction on test set, respectively.

d RMSE_fit_, RMSE_cv_, and RMSE_prd_, are the root-mean-square errors of internal fitting in training set, internal cross-validation on training set, and external blind prediction on test set, respectively.

**FIGURE 1:**
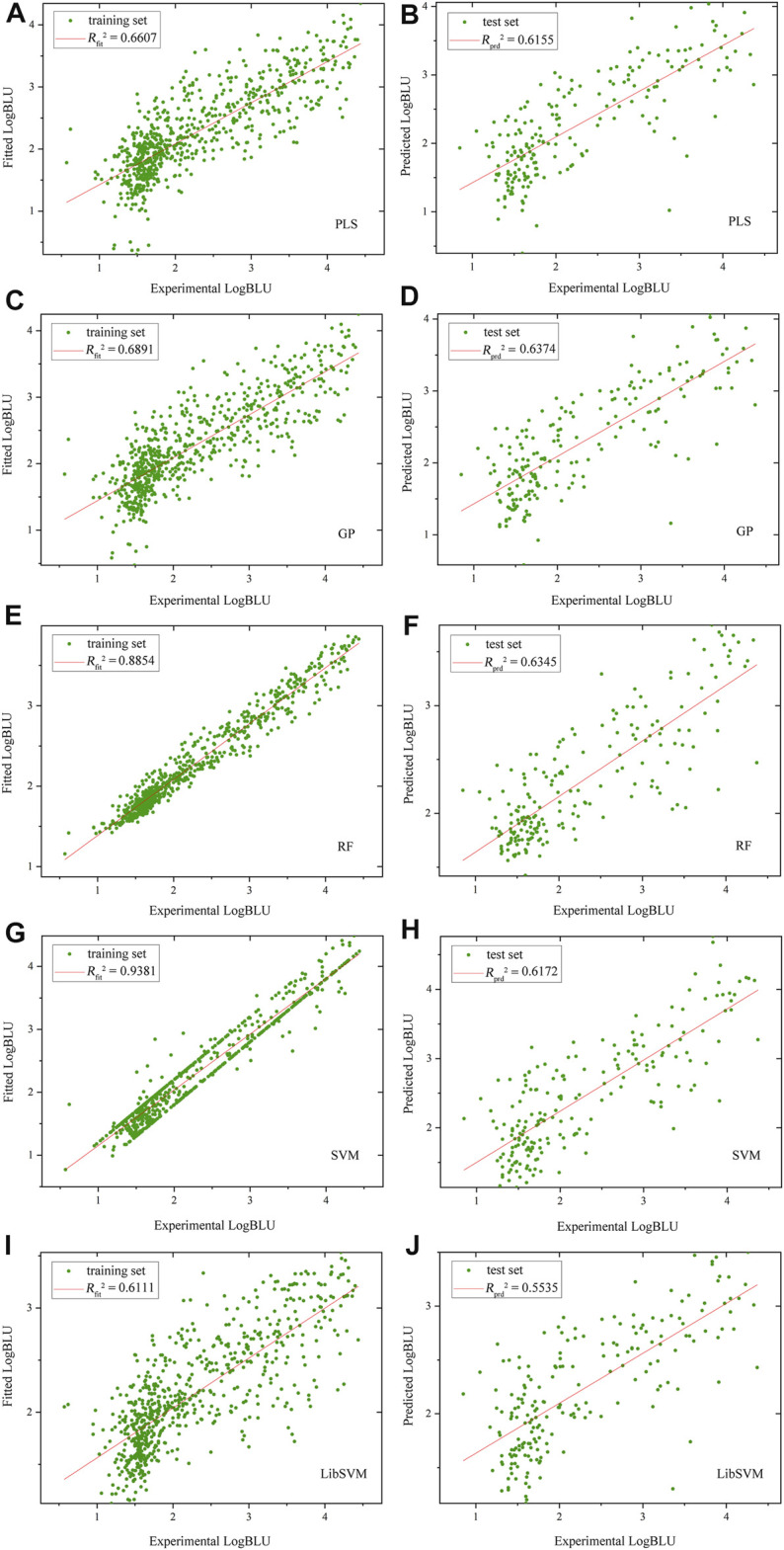
Scatter plots of fitted/predictive against experimental LogBLU values over 884 human amphyphisin SH3 (1/1)-binding peptides with MolSurf characterization and using different MLMs **(A,B)**, PLSR, **(C,D)**, GP; **(E,F)**, RF, **(G,H)**, SVM and **(I,J)**, LibSVM.

The optimal models were built on human amphyphisin SH3 (1/1)-binding peptide panel with MolSurf characterization. Here, the scatter plots of fitted/predictive against experimental LogBLU values over 884 peptide samples using different MLMs are shown in Figure 1. As can be seen, the resulting external predictive *R*_*prd*_^2^ values are generally larger than 0.5, indicating a good generalization ability on this panel. In addition, the internal fitting *R*_fit_^2^ values of all these MLMs (except LibSVM) are significantly higher than 0.65, in which the RF and SVM perform much better than others. However, there are no essential difference between the predictive powers of RF and SVM with PLS and GP (*R*_*prd*_^2^ > 0.6), but are moderately better than LibSVM (*R*_*prd*_^2^ < 0.6). The nonlinear GP, RF and SVM seem to have a good generalization ability relative linear PLS, albeit the difference is not very significant, suggesting that both the linear and nonlinear approaches exhibit similar predictability on test set, although the nonlinear methods can give stronger fitting on training set than linear one. This is also explain why the linear PLS has been successfully used in previous pQSAR modeling of DPI affinities, which can perform similarly but are easier to operate and more readily interpretable than those nonlinear modeling.

**FIGURE 2:**
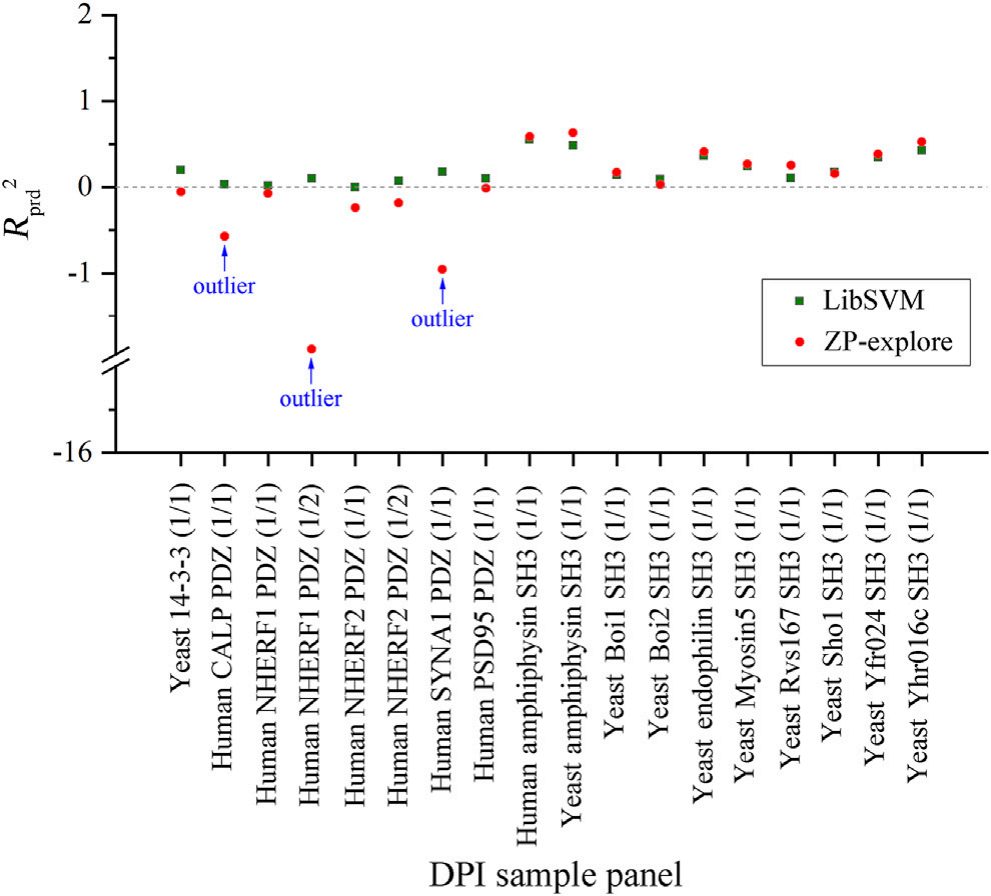
Comparison between the external predictive powers (*R*_*prd*_^2^) of SVM-based pQSAR modeling on different DPI sample panels with ZP-explore and LibSVM.

By comparing the SVM regressions modeled by in-house ZP-explore toolbox (Zhou et al., 2009) and sophisticated LibSVM program (Chang and Lin 2011), it is revealed that the former can perform considerably better than the latter, although both of them used the same machine learning method (SVM), worked on the same data panel (human amphyphisin SH3 (1/1)-binding peptides) and characterized the same AAD (MolSurf). This finding suggested that the pQSAR modeling of DPI affinities are sensitive to not only the data sets measured, but also the software used. This issue is usually neglected by the pQSAR community and previous works have no systematic examination of different tools/programs/software used in modeling. Therefore, we herein further compared the external predictive powers (*R*_*prd*_^2^) of SVM regressions modeled by ZP-explore and LibSVM on all the 18 DPI sample panels in Figure 2. It is revealed that the prediction can achieve a generally consistent power for some panels (e.g., human amphyphisin SH3 (1/1)- and human Biol1 SH3-binding peptides), but varies considerably for some others (e.g., human NHERF1 PDZ (1/2)- and human SYNA1 PDZ (1/1)-binding peptides). It is worth noting that, although the ZP-explore can yield a better prediction on certain panels than LibSVM, the latter appears to be more stable than the former, as characterized by the ZP-explore predictive outliers for three PDZ panels in Figure 2, although for most panels the two tools can work similarly in their predictive behavior.

### 3.2 Effect of Amino Acid Descriptors on Peptide Quantitative Structure-Activity Relationship Modeling

**FIGURE 3:**
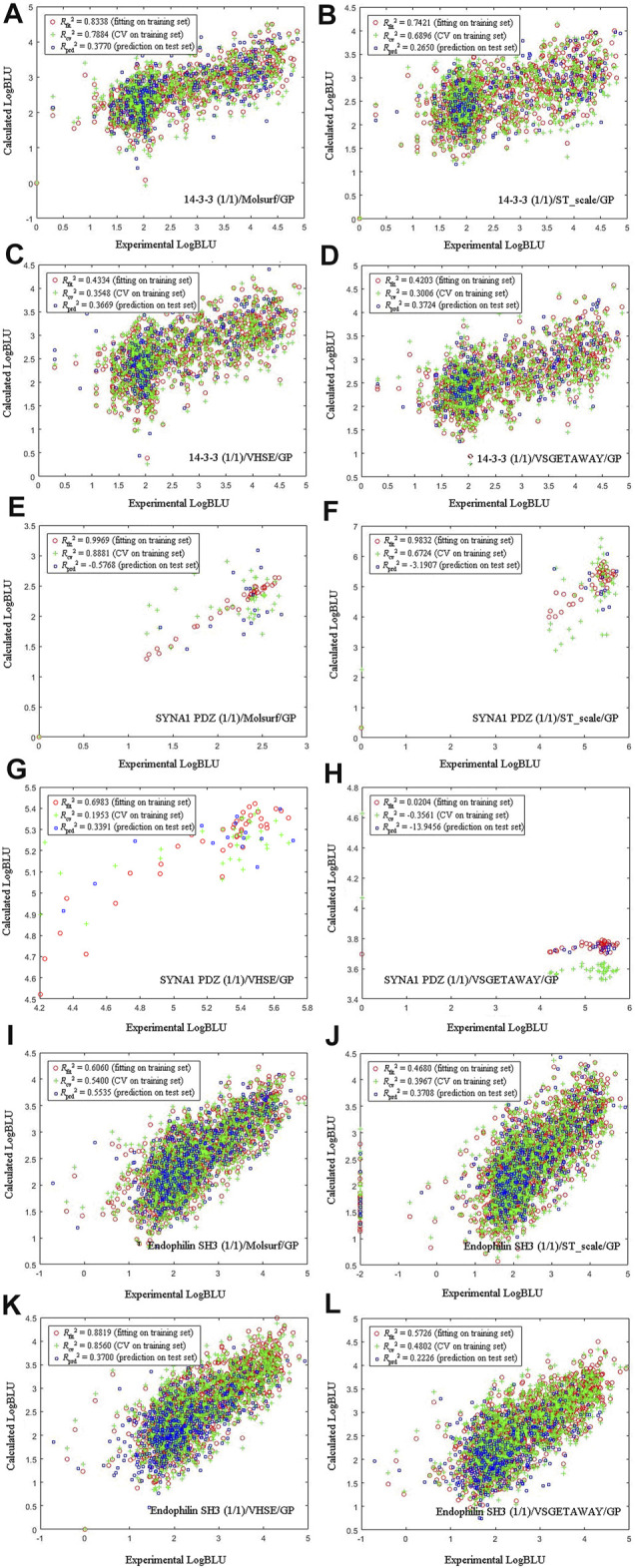
Scatter plots of calculated against experimental LogBLU values over 1193 human amphyphisin SH3 (1/1)-binding peptides **(A-D)**, 56 human SYNA1 PDZ (1/1)-binding peptides **(E-H)** and 2025 yeast endophilin SH3 (1/1)-binding peptides **(I-L)** with GP modeling and using different AADs.

Four amino acid descriptors characterizing different properties of amino acids, namely MolSurf (quantum-chemical), ST_scales (topological), VHSE (physicochemical) and VSGETAWAY (3D-structural), were used to parameterize peptide sequences, which were then correlated with experimental LogBLU values with GP modeling on three selected DPI sample panels: human 14-3-3 (1/1), human SYNA1 PDZ (1/1), and yeast endophilin SH3 (1/1), and the resulting scatter plots of calculated against experimental LogBLU values over these panels are shown in Figure 3. It is evident that the calculated results, including internal fitting ability *R*_*fit*_^2^ on training set, internal cross-validation stability *R*_*cv*_^2^ on training set, and external predictability *R*_*prd*_^2^ on test set, vary considerably over pQSAR models built with different AADs. For the 56 human SYNA1 PDZ (1/1)-binding peptides, the *R*_*fit*_^2^, *R*_*cv*_^2^ and *R*_*prd*_^2^ all exhibit considerable illness, indicating that the pQSAR models cannot work effectively on this panel, no mater which AADs were used. In contrast, pQSAR modeling seems to have a moderate or good performance on the 1193 human 14-3-3 (1/1)- and 2025 yeast endophilin SH3 (1/1)-binding peptides, with a satisfactory profile of internal fitting ability and cross-validation stability (*R*_*fit*_^2^ > 0.6 and *R*_*cv*_^2^ > 0.5), albeit many have only a moderate or modest external predictive power (*R*_*prd*_^2^ < 0.4). In addition, for the same sample panels characterized using different AADs, the pQSAR models generally exhibit a similar performance on both training and test sets, suggesting that the descriptor types would not have significant effect on modeling performance. However, the change in sample panels can lead to a considerable variation on the performance, suggesting that the AADs are not primarily responsible for pQSAR modeling; instead, the sample panels are.

**FIGURE 4:**
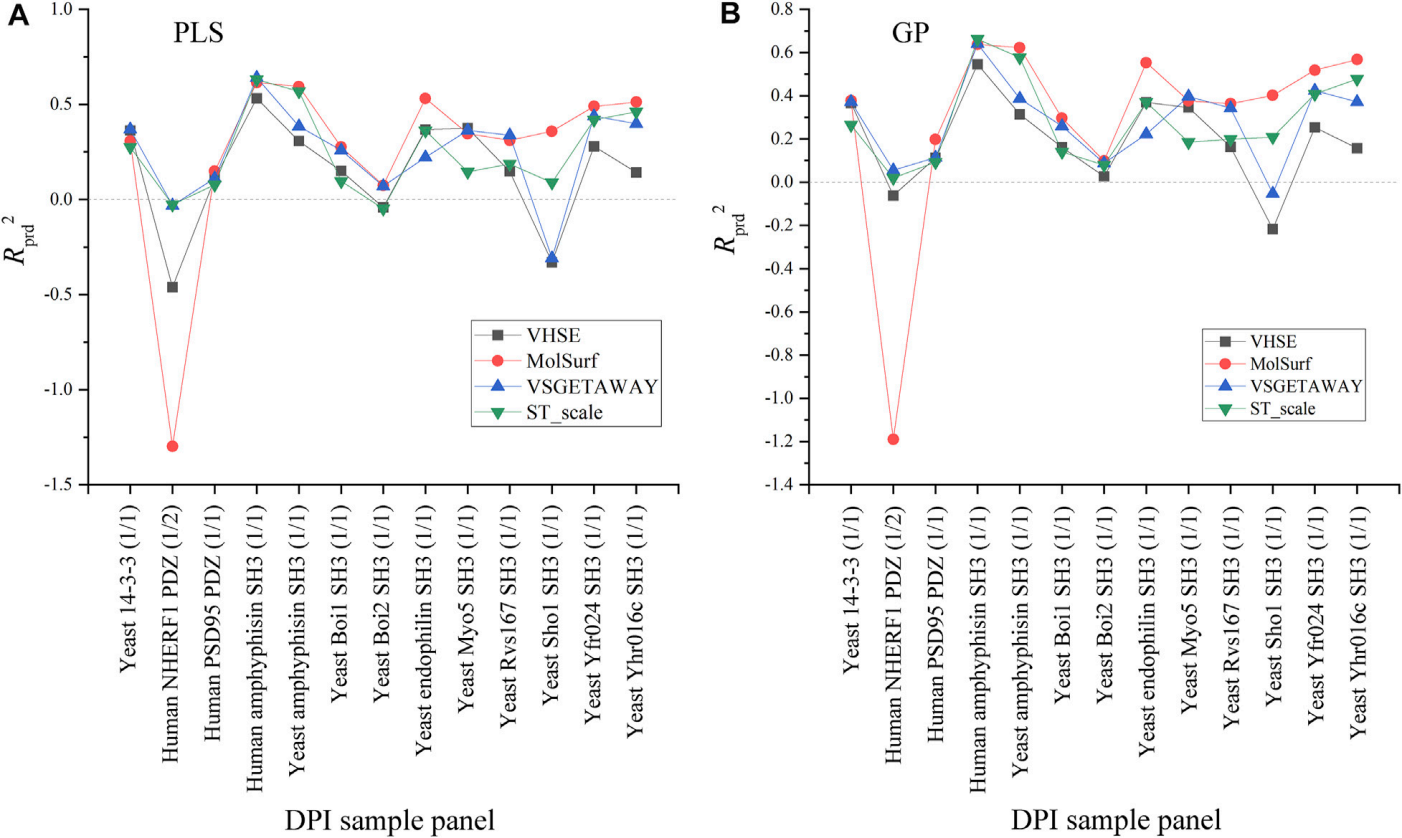
Comparison between the external predictive powers (*R*_*prd*_^2^) of PLS-/GP-based pQSAR modeling on different DPI sample panels with MolSurf, ST_scale, VHSE and VSGETAWAY.

Effects of four AADs on the external predictive powers (*R*_*prd*_^2^) of PLS-/GP-based pQSAR models are compared in Figure 4. As can be seen, the linear PLS (A) and nonlinear GP (B) have a similar profile of *R*_*prd*_^2^ values over these panels, in which the prediction on human NHERF1 PDZ (1/2) and Yeast Sho1 SH3 (1/1) vary significantly and moderately over the four AADs, respectively, while these descriptors exhibit a generally consistent performance for predicting other sample panels. For human NHERF1 PDZ (1/2) panel, the quantum-chemical MolSurf performs much worse, and secondly the physiochemical VHSE, whereas other two descriptors can work normally on this panel. For Yeast Sho1 SH3 (1/1) panel, only the quantum-chemical MolSurf has a particularly low performance as compared to other three descriptors. Besides, the four AADs seem to have a consistent performance on other panels. Even so, the pQSAR *R*_prd_^2^ values obtained with different descriptors on these panels mainly range between 0 and 0.6, imparting that the models have only a moderate or modest predictive power on most sample panels, and the *R*_prd_^2^ variation is primarily influenced by sample panels but not descriptor types.

### 3.3 Effect of Sample Size on Peptide Quantitative Structure-Activity Relationship Modeling

By systematically examining the influence of MLMs and AADs on pQSAR modeling of different DPI sample panels, it is revealed that the these models can perform fairly well on the human PSD95 PDZ (1/1) panel, which contains totally 6,068 peptide samples. Here, the MolSurf was employed to characterize the structure of these peptides at sequence level and then we carried out pQSAR modeling on all the 6,068 samples and two subsets with PLS, GP, RF, SVM and LibSVM regressions. The two subsets separately contain 1,000 and 3,000 sample data extracted randomly from the intact panel. The modeling resulted in 15 pQSAR models, which represent the systematic combination between five MLMs and three subsets with different sample sizes. The external predictive power (*R*_*prd*_^2^) of these models on test set is listed in Table 4. It is seen that the models with fullset-6068 can generally obtain a consistent predictability for most MLMs as compared to other two subsets, except the RF modeling on the subset-3000, which yielded the highest *R*_*prd*_^2^ than subset-1000 and fullset-6068. In contrast, the pQSAR modeling on subset-1000 can only obtain a marginal prediction. However, the *R*_*prd*_^2^ difference is not very significant between different subsets for the same MLMs, but different MLMs can lead to a considerable variation in the *R*_*prd*_^2^ value. In addition, all the five MLMs can reach the highest fitting ability (*R*_*fit*_^2^) with the fullset-6068 relative to subset-1000 and subset-3000. Therefore, it is revealed that the pQSAR performance is primarily determined by MLMs used and, secondarily, sample size. The larger the size is, the higher the performance is. Even so, the *R*_*prd*_^2^ values of pQSAR modeling on the fullset-6068 are all not above the 0.5, indicating that the absolute predictive power of different MLMs is improved with sample size increase, but the increase is quite limited.

**TABLE 4:** Change in external predictive power (*R*_*prd*_^2^) of PLS/GP/RF/SVM/LibSVM-based pQSAR modeling on human PSD95 PDZ (1/1) panel characterized by MolSurf with different sample sizes of subset-1000, subset-3000 and fullset-6068.

Size	*R*_*prd*_^2^				
	PLS	GP	RF	SVM	LibSVM
Subset-1000	0.014	0.080	0.362	0.071	0.084
Subset-3000	0.087	0.088	0.485	0.119	0.117
Fullset-6068	0.149	0.198	0.421	0.204	0.147

## 4 Conclusion

More than 20,000 SLiM-containing peptides as the binders of 3 peptide-recognition domains (PDZ, SH3 and 14-3-3) and 18 domain subtypes were comprehensively collected to perform an investigation of the applicability of pQSAR methodology in peptide affinity prediction. With a systematic combination of five widely used MLMs and four informatively diverse AADs to perform the pQSAR modeling on these peptide samples it is revealed that the domains and MLMs have significant effects on modeling performance, whereas the AADs and sample size can only influence the performance moderately and modestly. However, at most conditions the predictive power of pQSAR models is generally below 0.5 and only very few can be above 0.6, no matter what the combinations of domains, MLMs, AADs and sample size are adopted. This can be attributed to the fact that the high-throughput detection of arbitrary light intensity is a very indirect approach to characterize DPI affinity and the obtained BLU can only give a qualitative or semi-quantitative measure of the affinity values, thus causing a considerable bias in the pQSAR modeling and prediction. Instead, although some other affinity indicators such as *K*_d_ and ∆*G* are quantitative and more reliable, they cannot be tested in a high-through manner and thus are normally unavailable for large-scale DPI samples. Therefore, it is suggested that only focus on pQSAR modeling by optimizing AADs and MLMs is not an essential solution to improve the modeling performance of DPI affinity. Instead, the source of affinity data used to perform the modeling is the current bottleneck to restrict the feasibility and applicability of pQSAR methodology in DPI affinity prediction.

## Data Availability

The original contributions presented in the study are included in the article/Supplementary Material, further inquiries can be directed to the corresponding authors.
