# Supplementary material for: Systematic Modeling, Prediction, and Comparison of Domain–Peptide Affinities: Does it Work Effectively With the Peptide QSAR Methodology?
Source: Front Genet. 2022 Jan 14;12:800857. doi: 10.3389/fgene.2021.800857 (PMC8795790; doi:10.3389/fgene.2021.800857)
Supplement: Supplementary file 1 [file DataSheet1.PDF]

Supplementary Materials

Table S1. Sequences and affinities of 10 SH3-binding peptide panels (Landgraf C, et al. *PLoS Biol* 2004, 2, e14)

Human amphiphysin SH3 (1/1) [884]

Peptide	LogBLU
DPPRRPPRNG	3.91
PPPPRPPKRI	3.55
AVLRRPQKGR	1.93
DCIRRPLRPG	2.93
EALKRPMKQK	1.86
VDIKRPSRGQ	2.82
HHPKRPSRQI	3.60
QRPRRPAKDA	3.92
CVPRRPARAL	4.11
AEPKRPSRPI	3.59
RGLKRPGKKG	1.41
SALRRPWRKC	2.73
QPPKRPFKRM	2.90
PPPKRPLKVS	2.07
LSPTRPPKKP	2.78
AGIRRPEKPP	1.85
GPPRRPSRHD	4.25
LPPRRPERPV	3.54
KDIRRPERRH	2.73
LFLKRPKRSL	3.36
RQPRRPIRIQ	3.50
PALRRPSRAC	3.70
RSPPRPPRLW	4.18
YCFKRPDRCK	2.73
LPPKRPIKEV	2.57
EALKRPIKQK	1.96
PRPRRPPLQW	3.03
LLPRRPPSSS	2.07
VPPRRPPPPK	3.89
RPPRRPPWGR	4.00
GPPRRPPPHI	2.12
GFPRRPPPRG	3.81
KRPKRPPLKE	3.43
ASPRRPPALR	2.93
EPPRRPPPAP	2.57
TLPRRPPVPC	2.49
LLPKRPPKTT	2.45
DFPRRPPWSY	1.73
PVPPRPPRPP	3.73
ILPRRPPTPE	1.76
IPPKRPPRKH	4.24
LLPRRPPPRV	3.65
ASPRRPPGPT	2.32
GFPRRPPPRG	3.48
KRPKRPPLRE	3.60
AGPRRPPGDP	1.57
GPPRRPPRSV	4.31
SRPGRPPKRT	2.98
PPPRRPPGNI	3.01
PRPRRPPPED	1.60
QSPKRPPSPI	2.54
EGPRRPPPRP	3.52
AFPRRPPPRG	3.79
VVPRRPPAHI	3.79
RRPRRPPTVF	3.19
ESPWKPPTYL	1.60
VVPKKPPPSP	1.81
LPPPRPPLAV	3.02
APPPRPPKNQ	2.94
LLPERPPYLD	1.76
YGPHRPPQPL	1.73
PIPSKPPGGF	1.62
QVPPRPPPPR	3.37
GVPGRPPGLE	1.71
ESPGRPPPTA	1.34
ALPPRPPPPA	1.65
PIPERPPPVP	1.58
PLPPKPPFPG	1.64
AIPNRPPDAV	1.70
GPPTRPPGPW	3.12
EPPPKPPRSC	3.67
QPPPRPPSPR	3.12
GRPIKPPKKD	1.69
HPPGRPPAVR	2.53
KGPSRPPLDA	1.46
DFPKKPPQPE	1.81
YRPPRPPKGA	3.58
LSPNRPPLGG	2.27
PPPRRPPCRLS	4.44
GSPRKPPADA	1.56
APPSRPPPPR	3.74
PRPSRPPPPT	3.86
PKPPKPPRPP	3.55
DSPAKPPEGS	1.72
PPPVKPPPSP	1.64
APPTRPPRRC	4.31
APPKKPPRPG	3.39
NIPMKPPTPL	1.77
PPPGRPPGPP	1.52
LLPGKPPKSR	1.70
TPPPRPPLPT	2.85
VGPNRPPTVS	1.71
PKPNRPPNVD	2.50
QPPPRPPPDY	1.84
DKPVKPPTKK	1.68
RLPPKPPPPL	2.95
PVPPRPPAAE	1.70
PSPLRPPAPP	2.48
GKPERPPPQG	1.76
IRPPRPPKVL	4.17
KPPPKPPSPP	1.86
PRPARPPGDL	2.18
APPLRPPGPP	2.55
GRPTKPPPNS	1.82
QPPGRPPTPG	2.49
VPPIKPPKEI	1.64
APPTKPPTTP	1.62
PSPARPPRRR	4.22
LSPLRPPSVL	1.60
QPPQRPPPPT	3.14
IPPDRPPGAS	1.73
PPPGRPPLRL	1.53
SGPERPPALL	1.72
LRPLKPPPPP	2.94
AIPSKPPAAS	1.60
RVPVRPPQQY	2.12
ILPKKPPVKP	1.65
FLPARPPRAQ	4.27
AKPTRPPSES	1.69
IPPYRPPQQG	2.23
FLPPKPPPPI	1.55
TPPSRPPQVK	2.34
AKPHKPPGSM	2.21
ASPKKPPPGE	1.64
GPPQKPPGPA	1.67
DLPRKPPLPS	1.68
PSPDRPPHSQ	1.75
GPPRKPPGTV	1.79
VRPSRPPRGF	4.09
APPAKPPSTQ	1.73
MVPAKPPKGL	1.75
NSPEKPPEAG	1.61
GKPERPPPQG	1.77
GPPQKPPRLG	3.43
DLPKKPPPPE	1.72
RLPSRPPLPG	2.43
HVPAKPPRAA	3.61
GGPPRPPMGM	1.94
ARPPRPPYPP	4.20
ALPGRPPRWL	4.21
GPPCKPPAPE	1.59
PFPSRPPVLT	1.68
QSPKKPPAKD	1.76
ASPLRPPGAG	1.81
LPPARPPTRD	3.24
SKPRKPPMVM	1.77
PFPPRPPGPL	1.83
SSPQKPPTPE	1.64
GRPIKPPRKD	3.95
KIPKKPPAPD	1.75
FLPYRPPRPA	3.86
RRPGRPPKFI	2.70
SLPDKPPPQP	1.76
IPPKKPPTKV	1.59
VSPNKPPAVS	1.62
ALPPKPPKPM	1.95
QGPPRPPQGG	1.77
PLPNKPPPED	1.63
AGPLRPPPPG	1.79
GSPRKPPMDE	1.78
VLPARPPPPP	2.85
IPPNKPPWFD	1.64
PIPKKPPKGK	1.81
VLPQRPPEER	2.40
LPPGKPPSSS	1.37
EPPPRPPPAL	2.98
PPPPKPPRPQ	2.71
PLPIRPPSGT	2.67
DPPERPPPPP	1.64
PLPRRPPRAA	4.37
HVPIRPPRPS	4.09
PKPIKPPTYQ	1.72
GGPGRPPDWT	1.64
ASPAKPPSPG	1.79
VPPARPPTAQ	1.96
QLPPKPPAWA	1.59
AGPSRPPTPG	3.01
IPPPRPPKPH	2.80
CRPIKPPGPP	1.68
GGPWRPPPPG	1.64
HRPPRPPQPP	2.74
SGPERPPEKP	1.59
VLPCRPPIGL	1.61
RKPAKPPVSP	1.73
VLPPRPPKVL	2.54
EGPGRPPLDG	1.67
EVPEKPPAGE	1.60
PVPPRPPPPC	2.16

Yeast amphiphysin SH3 (1/1) [2032]

Peptide	LogBLU
RRDPPRRPPRNGYG	3.91
RLGPPKRPPRKLGP	4.23
ESPLPRRPPRAARS	4.37
REKKPPRPPKKKYQ	1.75
QVIRPPRPPKVLGL	3.95
QLCSPIRPPKYYTY	2.36
AIAPPPRPPKPSQA	3.48
DPPGPPRPPKEPGP	1.87
QVFLPPRPPKVLGL	3.06
HIPPPPRPPKRILE	3.55
WSRSPPRPPRERFE	3.88
PALSPTRPPKKPLD	2.78
LPRSPPRPPRLWSF	4.18
PPPPPPRPPKQQPP	3.00
RPPVPPRPPRPPET	3.73
ASSRPGRPPKRTQS	2.98
IQAPPPRPPKNQGL	2.94
PRQPPSRPPRRPPW	3.83
FYYRPPRPPKGACK	3.58
PPAPPTRPPRRCDS	4.31
RRQRPTRPPRQDKP	3.83
LVIRPPRPPKVLRL	4.17
EGPSPARPPRRRTS	4.22
GGFLPARPPRAQRH	4.27
RCVRPSRPPRGFQP	4.09
LVIRPPRPPKVLGL	4.09
RSRSPGRPPKSARR	2.19
PGRRPGRPPKFIRL	2.70
GGFKPSRPPRPSRP	4.26
ELHVPIRPPRPSCP	4.09
PDIPPPRPPKPHPA	2.80
QAVLPPRPPKVLEF	2.54
GSQVPERPPKPFPR	3.23
PGGKPERPPKPGPP	2.76
KPPKPPRPPKTLKL	3.11
PPLPPPRPPRPPSP	3.75
VRRIPTRPPRAPRL	4.40
QVILPPRPPKVLGL	2.97
MKKGPTRPPRPSTP	4.03
PSARPARPPRVRGP	4.21
PPKRPGRPPKNRES	3.88
ILRPPRRPRKPKTL	3.89
LVVDPRRPFRKLNA	3.47
AQARLKRPLKSLDG	2.07
DGKDPRRPKRPRTI	3.57
SSPLLKRPVRTQAV	3.22
KVTAPRRPQRYSSG	4.21
GNDDPRRPSRSPPS	3.34
PTQELRRPSKSMDK	1.69
NMSDPRRPNKVLRY	2.81
WPAGPRRPHRCSAS	3.86
EAGSPRRPRRQRSD	3.92
TKALLKRPLREIQM	2.94
ANPPPKRPVRASCR	4.15
QPLGPRRPMRPVGH	4.01
RPPGPRRPERGPRG	4.13
VMASLRRPKRIIIR	2.87
PFGQLKRPRRNGLG	2.71
ASSGLRRPEKPPYS	1.76
NGVEPKRPSRPINI	3.67
ASGGPRRPERHLPP	3.52
QYNFLRRPQRMPVS	3.09
PGQGLRRPARPGPA	2.93
VGDVLRRPSKDEPL	1.41
GAWPLKRPGRISSQ	3.18
PTVTPRRPGRPDHR	3.72
FSHILKRPDRVLLI	2.94
RDLIFKRPSRQFQY	3.71
AKRHPRRPPRSPGM	3.09
RAQRIRRPFRPILN	2.26
GLWLPRRPARSVGS	3.92
QVMVIKRPGRKRKA	3.21
LVNTLKRPKRPPLR	2.61
QNQSIRRPSRQLAP	3.16
DEARLRRPPRPSSD	3.44
LDPALRRPGRFDRE	2.82
PGVEPKRPCRPINL	3.94
NVSSFRRPRRSSDK	3.37
EDRLPRRPARSGGP	3.95
TAGPPKRPGRPPKN	3.71
PQPGFRRPARPETQ	3.45
KITPPRRPHRCSSG	3.82
DCSKLRRPLKRNRI	2.04
PRTLPRRPYKVLLA	2.04
LQKQPRRPRRWVTV	3.34
IIPSPKRPVKLLSG	1.87
ISWGLRRPIRLQMQ	2.97
MSSAPRRPAKGADS	2.25
PGPSLRRPSRELDA	2.65
PRAWPRRPRRDYSI	3.19
SSEEPRRPLRSDMV	3.28
ANIRLKRPLKMEDP	1.34
TTKVIRRPRRGRMG	2.07
NKIEFKRPGKAANK	2.09
FFKGIRRPWKGVLM	1.80
SLKIFRRPRKLFMQ	1.94
RSEAPRRPSRKGLR	4.10
DEEKPKRPTRRRRK	4.36
MYRDPRRPARGXLA	3.14
SPGVLRRPRRRPAA	3.39
TSAVLRRPQKGRLA	1.93
QCDCIRRPLRPGQY	2.93
IQEALKRPMKQKRK	1.86
TEVDIKRPSRGQTS	2.82
YQHHPKRPSRQIHK	3.60
GPQRPRRPAKDAQP	3.92
CDTSPRRPLRPTPN	3.55
KKYLPKRPAKDDPE	1.05
IAWGLRRPIRLQMQ	2.69
TRSVLKRPERSQEE	1.95
ISCVPRRPARALHP	4.11
SLPLPKRPRRGAAP	3.74
ATDSPRRPSRCTGG	4.05
NGAEPKRPSRPINI	3.59
HGRGLKRPGKKGSN	1.41
GRSALRRPWRKCGC	2.73
NFQPPKRPFKRMNY	2.90
GIPPPKRPLKVSQK	2.07
LPQWPRRPGRRWPA	2.91
TNAGIRRPEKPPYS	1.85
ILGPPRRPSRHDNS	4.25
GTVYPKRPEKPVPP	1.63
LRQPLRRPFRPNGL	3.23
GALPPRRPERPVGP	3.54
QVKDIRRPERRHSD	2.73
LALFLKRPKRSLFF	3.36
LWRQPRRPIRIQQR	3.50
LLPALRRPSRACCA	3.70
YRQQPKRPQKHIQK	2.01
DAYCFKRPDRCKMN	2.73
NLLPPKRPIKEVLT	2.57
IQEALKRPIKQKRK	1.96
KIGFFKRPLKKKME	2.17
ELPRPRRPPLQWRY	3.03
PGPGPRRPPWEGAD	1.40
TELLPRRPPSSSSS	2.07
PVVPPRRPPPPKKT	3.89
PSRPPRRPPWGRGS	4.00
PQGPPRRPPPHILD	2.12
QAGFPRRPPPRGTR	3.81
TLKRPKRPPLKEFF	3.43
TPASPRRPPALRSQ	2.93
GSEPPRRPPPAPHD	2.57
PAAPPRRPPQSASS	3.52
PCTLPRRPPVPCGQ	2.49
APQSPRRPPHPLPP	2.53
TILLPKRPPKTTED	2.45
VLDFPRRPPWSYEM	1.73
ESQSPKRPPEDFET	1.62
AEILPRRPPTPEAQ	1.76
VKIPPKRPPRKHIV	4.24
QQKPPKRPPIEASV	1.76
PPLLPRRPPPRVPA	3.65
LDASPRRPPGPTTS	2.32
QYGFPRRPPPRGSQ	3.48
TLKRPKRPPLREFF	3.60
GNAGPRRPPGDPST	1.57
QQPPPRRPPQAPEG	2.87
SRGPPRRPPRSVKL	4.31
MKPPPRRPPGNISW	3.01
ELPRPRRPPPEDEE	1.60
FWQSPKRPPSPIKF	2.54
PTEGPRRPPPRPPC	3.52
QAAFPRRPPPRGTR	3.79
PPVVPRRPPAHISS	3.79
SQRRPRRPPTVFRL	3.19
LEESPWKPPTYLFS	1.60
KRVVPKKPPPSPQP	1.81
LILPPPRPPLAVTS	3.02
PPLLPPRPPQGLEP	2.03
YYLLPERPPYLDKY	1.76
GPYGPHRPPQPLPP	1.73
TPPIPSKPPGGFGK	1.62
PSQVPPRPPPPRLP	3.37
CSGVPGRPPGLEEE	1.71
AAESPGRPPPTAAE	1.34
SQALPPRPPPPAPV	1.65
NVPIPERPPPVPPP	1.58
PSPLPPKPPFPGPG	1.64
AVAIPNRPPDAVLT	1.70
DPNFPNKPPLTFDI	1.66
PPGPPTRPPGPWRW	3.12
EMEPPPKPPRSCSA	3.67
APQPPPRPPSPRYE	3.12
SGGRPIKPPKKDLE	1.69
SIHPPGRPPAVRGP	2.53
KTKGPSRPPLDAKI	1.46
GKVVPLRPPPPPKS	2.70

Yeast Boil1 SH3 (1/1) [1336]

Peptide	LogBLU
LTIFKRPPKQPGHQ	2.13
IDKSKLSPRFPELK	2.08
TTTYKSRPDRPKPV	2.78
TELLRIIPEMPKDL	1.90
DIKTRSVPKIPSIE	2.54
NDLVKRFPEQPPLR	4.88
WLDEKRIPSTPVDY	2.03
PPRFKNFPTLPSKI	2.76
SLLAKYQPSLPLVE	1.65
NVLLKAKPNDPAAT	3.25
KIVERGPPYPPDLR	1.87
VSQRKKRPISPHLT	2.33
SMAMRPIPPLPTES	1.57
VKRPRLNPFLPRDE	5.13
SGSGRRYPQTPSYY	2.77
LGPLKYAPHLPFSK	2.12
PSHYRNIPYNPATF	2.27
GENIKITPQKPFWF	1.92
PHFAKHSPDSPRTS	2.18
CNRARYLPQNPDII	2.59
KLKIKILPEQPQLE	3.13
CAKSKSRPNTPGSD	3.24
FNGDRLVPTLPTVS	2.12
NESEKSSPWSPYKV	1.98
GFLSKKVPNTPVVV	2.63
IAVTRSIPRGPDKN	2.41
NELVKRFPGHPPLR	2.87
RRRTRLRPPTPLSQ	2.14
QSKTKPLPVEPASP	1.90
NGKDKSRPPRPPPK	2.82
TPQSRFPPYSPLLP	1.76
NSFRKILPQEPEIW	3.07
TILERLTPLVPNNL	1.71
KFFKREYPDIPMIA	2.30
LFGIRNNPFIPITG	2.45
FIKNKAKPLIPIEL	2.85
LSYLKDLPFVPFLF	1.80
YCPWKLCPLLPILY	1.95
VFYHRPAPKPPVTK	2.94
ILERKNIPRQPAIR	3.25
SKIKRSRPPPPPMD	3.10
DLQKKYEPSPPATH	2.06
GLDLKWEPSTPQRK	3.31
MDGIKELPEFPIKK	1.41
RLVSKYRPNCPIIL	2.39
ISNRRPVPRRPSQP	3.66
MKPQKIAPKFPGTV	3.05
LDTHKESPLRPKVR	3.45
YVVKKNFPVSPNYT	2.37
VRSKRLFPVRPMAT	2.56
LNGLKSSPADPHLS	1.81
KLHEKYFPDLPKEV	1.96
MQSKKEDPNRPKNL	2.95
DDIIKDHPHIPNGQ	2.27
MDMDRNVPLAPNLN	1.62
TNSVKATPVPPTLA	1.73
ATVVKNCPGDPELF	2.81
QFALKNTPITPGSC	2.15
GYNPKTVPFVPISG	1.60
MLNSKHYPYIPVAN	1.76
KTGRKTFPFLPYQI	2.11
PELAKNSPLAPIKK	2.84
ELQIRYKPTPPLKK	1.94
SKNWKLPPRLPHRA	3.89
HFNTKKIPELPSQF	2.07
WEALKSVPIPPRKN	4.72
DSYSRSRPTRPLGY	4.13
RMQQKNSPAFPTHS	3.68
VPIDKKSPFYPGLD	2.18
ASKHKMFPFNPAKI	2.54
ATEAKCLPKIPSIP	2.03
TASGKTSPILPLQN	2.03
IAEWKNDPNNPNFE	1.84
VQIKRADPHFPHDE	1.53
DEALRYCPDAPIVL	1.57
EIAKRLQPGSPVLG	1.43
NQIARQIPFQPWYL	1.36
RTFFKLPPNLPEAE	2.34
EKELKEGPSSPASI	1.67
ILAVKKNPQSPMYT	2.11
TTRRKKDPNAPKRR	2.63
PSSHKRKPVIPWDA	1.97
KYCFKLVPPQPGSK	2.30
LTLTKWDPSKPISL	2.28
DIPRRYIPITPADL	4.15
IKYFKKFPKDPLAM	2.12
AKLHRTKPVKPLSK	2.58
CLRARNGPVPPVHI	2.81
RTPSKPDPMEPENT	1.45
INNNKMRPHLPPLS	3.12
TKKRKLGPKAPNPL	2.69
DFIGRKSPDLPEYL	2.14
SANGKFIPSRPAPK	2.11
KSIPRTKPNVPPLQ	2.79
VAPNRIPPKVPVIN	2.01
EWEEKLTPAGPLCP	1.82
VASSRTEPSTPSRP	2.08
RRHNRQHPPYPHSG	2.48
TEFSRPIPPTPSGF	1.61
EEQVKCNPNRPIYW	2.17
TEIYKLEPREPQVD	2.12
IYHCKKQPLVPIGC	2.26
AQKLKLLPEEPFQR	2.85
AKLRKFHPPEPYKG	2.44
LVGLKYEPLFPYFA	1.70
AGMAKTKPDVPLSY	1.32
TSNTKWTPITPSVI	1.47
SLIPKIEPAEPTYS	2.23
MRRCKDAPIEPPKY	2.74
HELVKSDPHFPYGT	1.57
KRRSKSLPTTPGIR	2.49
KRNSKHAPFIPVKP	2.61
NGTWKYNPTVPNEL	1.86
NITEKSPPKSPKHK	3.53
KKIAKGDPKIPMQN	2.89
EKYGKDQPESPVPE	1.79
KNLKKYDPEDPNRR	2.82
LSKVRGRPMYPRPA	2.68
CFKTKFAPEAPDLF	1.78
PNPLKKAPIPPLAY	1.86
YEGPRGAPGMPEML	1.81
GSQWKNHPDWPFWV	2.12
GIKVKRIPYSPKSI	2.13
NDDKKTEPDKPAFS	1.72
CKYNRQHPANPAAA	2.32
LNRKKVTPQLPKVT	2.58
KIIKKCSPSVPKEE	2.59
IDIPRTNPHIPLYQ	1.70
NSNVRIPPTVPSKI	2.25
PESAKCNPNSPRLK	2.79
EGRCRLFPHCPLGR	4.17
DKFIKLIPMRPVLI	2.07
KKKKKNGPGAPQGY	2.52
ILEGKFEPVIPREW	1.73
FRPHRLAPSAPATK	3.96
EVENKLRPDLPVKN	1.50
PRSKKKKPAPPTLL	1.93
DIPLKQVPENPMPT	2.04
GQQKRFSPSLPSAV	3.08
GRSLRKRPTSPSIS	2.57
VEKRKFTPIEPSLL	2.77
DLKKKYAPDPPITH	2.22
HILEKYSPFDPCGR	1.42
QSINRSIPSAPYGA	1.57
RLPSKTDPLDPHIK	2.51
KVQIKKVPTDPSSS	3.16
RKSDKNAPSVPQVD	3.14
EDLVKKPPFIPSPT	1.61
IAKLKRKPVVPRLK	2.14
EYSPRPSPWLPSKP	2.26
TKRQRGEPSEPQQD	3.31
TGYPRYFPQPPVQR	3.57
IPAPKLVPPPPRTR	4.24
EKNARGIPQAPFIE	1.68
GSLVKTKPTLPLTK	2.96
YGSEKGSPAGPSAV	1.81
NTIIKFDPGRPIYN	1.70
KLYYRHRPQKPVRT	2.78
PVPVRMQPQPPQPM	1.97
ATKRKTIPVYPPVQ	2.42
VSKAKLQPAKPQFT	2.82
IRTYKVVPLGPKAG	2.16
IFVTRYLPYFPIMY	1.78
AALVKFVPSKPMCV	2.03
FLHSKKIPEVPQTD	1.58
NRANKYDPKGPIYN	2.74
NTVEKLQPGLPVII	1.61
TVCDRLIPTSPQKT	1.70
FENRKHQPFTPKGM	2.79
APPPKEEPAAPTST	1.88
GLNLKWEPSTPQRN	2.65
LLVTKERPRFPEDD	2.10
KKNDRPSPLKPIQH	3.34
AFWWKKNPKTPSDY	2.04
LGLHKWLPDIPLFE	2.26
FQYTKGRPLAPKGA	1.78
YAVDKIMPPTPAKK	1.65
KRRSKSLPITPKSI	2.77
TQPVRKNPEAPARR	4.24
KSLQKFEPRYPKTA	2.91
HRLFKCTPGLPKLW	2.35
ADDLKYIPPIPQDF	2.12
RLLFRSLPPHPACL	2.67
LVGMRSFPLYPSGL	2.71
DDNVRPSPISPSIN	1.28
RRIRKEHPGEPNVI	2.96
PEWRRPHPNIPDAK	3.11
DKKKKKYPRKPKKN	2.35

Yeast Boil2 SH3 (1/1) [1312]

Peptide	LogBLU
DETRKLTPCSPGDD	1.22
LTIFKRPPKQPGHQ	1.57
IDKSKLSPRFPELK	1.02
TTTYKSRPDRPKPV	1.37
TELLRIIPEMPKDL	1.20
DIKTRSVPKIPSIE	1.37
NDLVKRFPEQPPLR	3.69
WLDEKRIPSTPVDY	2.26
PPRFKNFPTLPSKI	1.78
SLLAKYQPSLPLVE	1.89
NVLLKAKPNDPAAT	1.47
KIVERGPPYPPDLR	0.67
VSQRKKRPISPHLT	1.33
SMAMRPIPPLPTES	1.69
VKRPRLNPFLPRDE	3.38
SGSGRRYPQTPSYY	1.62
LGPLKYAPHLPFSK	1.85
PSHYRNIPYNPATF	0.93
GENIKITPQKPFWF	1.55
PHFAKHSPDSPRTS	1.04
CNRARYLPQNPDII	1.66
CAKSKSRPNTPGSD	1.37
FNGDRLVPTLPTVS	2.28
NESEKSSPWSPYKV	1.45
GFLSKKVPNTPVVV	0.72
IAVTRSIPRGPDKN	1.54
NELVKRFPGHPPLR	0.90
RRRTRLRPPTPLSQ	1.61
QSKTKPLPVEPASP	0.77
NGKDKSRPPRPPPK	1.50
TPQSRFPPYSPLLP	0.88
NSFRKILPQEPEIW	2.34
KFFKREYPDIPMIA	1.31
LFGIRNNPFIPITG	1.41
FIKNKAKPLIPIEL	1.04
LSYLKDLPFVPFLF	0.82
YCPWKLCPLLPILY	2.00
VFYHRPAPKPPVTK	1.47
ILERKNIPRQPAIR	1.30
SKIKRSRPPPPPMD	1.55
ELDTKTIPELPFCM	1.39
DLQKKYEPSPPATH	0.45
GLDLKWEPSTPQRK	0.79
MDGIKELPEFPIKK	-0.05
RLVSKYRPNCPIIL	1.87
ISNRRPVPRRPSQP	1.76
MKPQKIAPKFPGTV	1.50
LDTHKESPLRPKVR	1.28
YVVKKNFPVSPNYT	1.46
LNGLKSSPADPHLS	1.59
KLHEKYFPDLPKEV	1.18
MQSKKEDPNRPKNL	1.70
DDIIKDHPHIPNGQ	-0.30
MDMDRNVPLAPNLN	1.50
TNSVKATPVPPTLA	0.43
ATVVKNCPGDPELF	1.23
GYNPKTVPFVPISG	0.76
MLNSKHYPYIPVAN	1.70
KTGRKTFPFLPYQI	1.47
ELQIRYKPTPPLKK	1.24
SKNWKLPPRLPHRA	1.47
HFNTKKIPELPSQF	1.55
WEALKSVPIPPRKN	3.24
DSYSRSRPTRPLGY	1.57
RMQQKNSPAFPTHS	2.36
VPIDKKSPFYPGLD	1.49
ASKHKMFPFNPAKI	0.18
ATEAKCLPKIPSIP	1.77
TASGKTSPILPLQN	1.32
IAEWKNDPNNPNFE	1.46
VQIKRADPHFPHDE	1.30
DEALRYCPDAPIVL	1.21
EIAKRLQPGSPVLG	0.95
NQIARQIPFQPWYL	1.56
RTFFKLPPNLPEAE	0.71
EKELKEGPSSPASI	1.12
ILAVKKNPQSPMYT	1.42
TTRRKKDPNAPKRR	1.11
PSSHKRKPVIPWDA	1.61
KYCFKLVPPQPGSK	1.30
LTLTKWDPSKPISL	2.02
HEASKCFPKAPQAS	0.87
DPSDKSLPQQPFHK	1.40
DIPRRYIPITPADL	1.72
IKYFKKFPKDPLAM	0.97
AKLHRTKPVKPLSK	0.66
RTPSKPDPMEPENT	1.55
INNNKMRPHLPPLS	2.81
TKKRKLGPKAPNPL	1.57
DFIGRKSPDLPEYL	1.62
SANGKFIPSRPAPK	0.73
KSIPRTKPNVPPLQ	0.73
VAPNRIPPKVPVIN	1.40
EWEEKLTPAGPLCP	1.12
VASSRTEPSTPSRP	1.02
RRHNRQHPPYPHSG	1.20
EEQVKCNPNRPIYW	1.36
TEIYKLEPREPQVD	1.19
IYHCKKQPLVPIGC	1.32
AQKLKLLPEEPFQR	1.21
AKLRKFHPPEPYKG	1.54
LVGLKYEPLFPYFA	2.03
TSNTKWTPITPSVI	0.83
SLIPKIEPAEPTYS	1.41
VIIGKTTPISPDEE	1.30
MRRCKDAPIEPPKY	2.84
HELVKSDPHFPYGT	1.51
KRRSKSLPTTPGIR	-0.10
KRNSKHAPFIPVKP	1.57
NGTWKYNPTVPNEL	1.38
NITEKSPPKSPKHK	1.27
KKIAKGDPKIPMQN	0.00
EKYGKDQPESPVPE	1.08
LSKVRGRPMYPRPA	1.57
CFKTKFAPEAPDLF	1.18
YEGPRGAPGMPEML	1.42
GSQWKNHPDWPFWV	0.98
GIKVKRIPYSPKSI	0.18
NDDKKTEPDKPAFS	1.24
CKYNRQHPANPAAA	1.69
LNRKKVTPQLPKVT	1.45
KIIKKCSPSVPKEE	-0.22
IDIPRTNPHIPLYQ	0.52
NSNVRIPPTVPSKI	1.23
EGRCRLFPHCPLGR	2.22
DKFIKLIPMRPVLI	-0.52
KKKKKNGPGAPQGY	1.31
ILEGKFEPVIPREW	1.85
FRPHRLAPSAPATK	1.41
EVENKLRPDLPVKN	1.40
PRSKKKKPAPPTLL	1.24
DIPLKQVPENPMPT	1.09
GQQKRFSPSLPSAV	0.97
GRSLRKRPTSPSIS	2.07
VEKRKFTPIEPSLL	1.89
DLKKKYAPDPPITH	1.32
HILEKYSPFDPCGR	1.29
QSINRSIPSAPYGA	2.09
RLPSKTDPLDPHIK	1.45
KVQIKKVPTDPSSS	1.81
EDLVKKPPFIPSPT	1.24
IAKLKRKPVVPRLK	1.60
EYSPRPSPWLPSKP	1.35
TDLGKDIPFFPSSE	1.19
TKRQRGEPSEPQQD	1.32
TGYPRYFPQPPVQR	1.53
IPAPKLVPPPPRTR	1.43
EKNARGIPQAPFIE	1.02
GSLVKTKPTLPLTK	1.41
YGSEKGSPAGPSAV	1.60
NTIIKFDPGRPIYN	1.25
KLYYRHRPQKPVRT	1.43
PVPVRMQPQPPQPM	1.93
ATKRKTIPVYPPVQ	1.78
QELPRAIPVMPFVD	1.35
VSKAKLQPAKPQFT	1.81
IRTYKVVPLGPKAG	1.59
IFVTRYLPYFPIMY	3.06
AALVKFVPSKPMCV	1.90
FLHSKKIPEVPQTD	0.91
NTVEKLQPGLPVII	1.52
TVCDRLIPTSPQKT	1.50
FENRKHQPFTPKGM	-0.15
APPPKEEPAAPTST	0.54
GLNLKWEPSTPQRN	0.66
LLVTKERPRFPEDD	0.88
AFWWKKNPKTPSDY	1.81
LGLHKWLPDIPLFE	1.18
FQYTKGRPLAPKGA	1.17
YAVDKIMPPTPAKK	0.30
KRRSKSLPITPKSI	0.79
TQPVRKNPEAPARR	1.51
HRLFKCTPGLPKLW	2.09
ADDLKYIPPIPQDF	1.20
RLLFRSLPPHPACL	1.83
ISHNKLSPSEPILC	1.53
LIWHKSKPVKPTNK	1.48
LVGMRSFPLYPSGL	2.51
DDNVRPSPISPSIN	1.28
RRIRKEHPGEPNVI	0.95
PEWRRPHPNIPDAK	1.61
VDSFKYEPLAPGND	1.13
GFQDRSLPHFPKNS	1.27
NAPPRIFPKAPIYG	1.60
NRKQRSIPLSPIVP	1.89
QSEEKEKPKGPMAV	1.22
EKEAKSEPQQPEDN	1.25
LNLSKDAPVPPAPE	1.28

Yeast endophilin SH3 (1/1) [1998]

Peptide	LogBLU
RRDPPRRPPRNGYG	3.70
RLGPPKRPPRKLGP	4.02
ESPLPRRPPRAARS	3.91
REKKPPRPPKKKYQ	3.81
QVIRPPRPPKVLGL	4.16
QLCSPIRPPKYYTY	3.47
AIAPPPRPPKPSQA	3.76
DPPGPPRPPKEPGP	2.67
QVFLPPRPPKVLGL	3.43
HIPPPPRPPKRILE	3.62
WSRSPPRPPRERFE	4.26
PALSPTRPPKKPLD	3.24
LPRSPPRPPRLWSF	4.38
PPPPPPRPPKQQPP	3.06
RPPVPPRPPRPPET	3.93
ASSRPGRPPKRTQS	3.76
IQAPPPRPPKNQGL	3.36
PRQPPSRPPRRPPW	3.92
FYYRPPRPPKGACK	3.66
PPAPPTRPPRRCDS	4.08
RRQRPTRPPRQDKP	3.49
LVIRPPRPPKVLRL	4.29
EGPSPARPPRRRTS	3.73
GGFLPARPPRAQRH	4.17
RCVRPSRPPRGFQP	4.12
LVIRPPRPPKVLGL	4.32
RSRSPGRPPKSARR	3.57
PGRRPGRPPKFIRL	4.19
GGFKPSRPPRPSRP	4.36
ELHVPIRPPRPSCP	3.60
PDIPPPRPPKPHPA	3.80
QAVLPPRPPKVLEF	3.20
GSQVPERPPKPFPR	4.13
PGGKPERPPKPGPP	3.65
KPPKPPRPPKTLKL	4.40
PPLPPPRPPRPPSP	3.90
VRRIPTRPPRAPRL	4.16
QVILPPRPPKVLGL	3.70
MKKGPTRPPRPSTP	4.24
PSARPARPPRVRGP	4.33
PPKRPGRPPKNRES	4.14
ILRPPRRPRKPKTL	3.12
LVVDPRRPFRKLNA	2.25
AQARLKRPLKSLDG	2.04
DGKDPRRPKRPRTI	1.93
SSPLLKRPVRTQAV	2.29
KVTAPRRPQRYSSG	3.75
GNDDPRRPSRSPPS	2.49
PTQELRRPSKSMDK	1.54
NMSDPRRPNKVLRY	3.02
WPAGPRRPHRCSAS	3.63
EAGSPRRPRRQRSD	2.58
TKALLKRPLREIQM	2.23
ANPPPKRPVRASCR	3.94
QPLGPRRPMRPVGH	3.46
RPPGPRRPERGPRG	3.70
VMASLRRPKRIIIR	2.25
PFGQLKRPRRNGLG	1.83
ASSGLRRPEKPPYS	2.02
NGVEPKRPSRPINI	1.55
ASGGPRRPERHLPP	4.10
QYNFLRRPQRMPVS	2.88
PGQGLRRPARPGPA	3.12
VGDVLRRPSKDEPL	2.06
GAWPLKRPGRISSQ	2.33
PTVTPRRPGRPDHR	2.62
FSHILKRPDRVLLI	2.47
RDLIFKRPSRQFQY	2.28
AKRHPRRPPRSPGM	2.15
RAQRIRRPFRPILN	1.91
GLWLPRRPARSVGS	3.27
QVMVIKRPGRKRKA	2.38
LVNTLKRPKRPPLR	1.68
QNQSIRRPSRQLAP	2.82
DEARLRRPPRPSSD	2.24
LDPALRRPGRFDRE	2.20
PGVEPKRPCRPINL	2.36
NVSSFRRPRRSSDK	2.02
EDRLPRRPARSGGP	3.96
TAGPPKRPGRPPKN	1.97
PQPGFRRPARPETQ	1.99
KITPPRRPHRCSSG	3.44
DCSKLRRPLKRNRI	2.22
PRTLPRRPYKVLLA	2.94
LQKQPRRPRRWVTV	2.34
IIPSPKRPVKLLSG	3.03
ISWGLRRPIRLQMQ	2.86
MSSAPRRPAKGADS	2.35
PGPSLRRPSRELDA	2.35
PRAWPRRPRRDYSI	2.34
SSEEPRRPLRSDMV	2.04
ANIRLKRPLKMEDP	2.36
TTKVIRRPRRGRMG	2.18
NKIEFKRPGKAANK	2.11
FFKGIRRPWKGVLM	2.20
SLKIFRRPRKLFMQ	1.87
RSEAPRRPSRKGLR	3.99
DEEKPKRPTRRRRK	2.98
MYRDPRRPARGXLA	3.10
SPGVLRRPRRRPAA	2.28
TSAVLRRPQKGRLA	2.14
QCDCIRRPLRPGQY	2.58
IQEALKRPMKQKRK	2.04
TEVDIKRPSRGQTS	1.89
YQHHPKRPSRQIHK	2.64
GPQRPRRPAKDAQP	4.03
CDTSPRRPLRPTPN	3.11
KKYLPKRPAKDDPE	2.29
IAWGLRRPIRLQMQ	2.93
TRSVLKRPERSQEE	1.73
ISCVPRRPARALHP	3.70
SLPLPKRPRRGAAP	3.98
ATDSPRRPSRCTGG	2.67
NGAEPKRPSRPINI	2.02
HGRGLKRPGKKGSN	1.85
GRSALRRPWRKCGC	2.20
NFQPPKRPFKRMNY	2.72
GIPPPKRPLKVSQK	2.84
LPQWPRRPGRRWPA	3.90
TNAGIRRPEKPPYS	2.05
ILGPPRRPSRHDNS	3.76
GTVYPKRPEKPVPP	2.98
LRQPLRRPFRPNGL	2.69
GALPPRRPERPVGP	4.14
QVKDIRRPERRHSD	2.09
LALFLKRPKRSLFF	2.92
LWRQPRRPIRIQQR	3.00
LLPALRRPSRACCA	3.54
YRQQPKRPQKHIQK	2.26
DAYCFKRPDRCKMN	1.66
NLLPPKRPIKEVLT	3.19
IQEALKRPIKQKRK	1.95
KIGFFKRPLKKKME	2.30
ELPRPRRPPLQWRY	3.77
PGPGPRRPPWEGAD	2.72
TELLPRRPPSSSSS	2.53
PVVPPRRPPPPKKT	4.20
PSRPPRRPPWGRGS	4.09
PQGPPRRPPPHILD	3.63
QAGFPRRPPPRGTR	3.87
TLKRPKRPPLKEFF	4.18
TPASPRRPPALRSQ	3.82
GSEPPRRPPPAPHD	3.37
PAAPPRRPPQSASS	3.16
PCTLPRRPPVPCGQ	3.33
APQSPRRPPHPLPP	3.45
TILLPKRPPKTTED	2.96
VLDFPRRPPWSYEM	2.60
ESQSPKRPPEDFET	1.69
AEILPRRPPTPEAQ	1.51
VKIPPKRPPRKHIV	4.17
QQKPPKRPPIEASV	2.92
PPLLPRRPPPRVPA	3.86
LDASPRRPPGPTTS	3.04
QYGFPRRPPPRGSQ	3.33
TLKRPKRPPLREFF	4.40
GNAGPRRPPGDPST	1.88
QQPPPRRPPQAPEG	2.63
SRGPPRRPPRSVKL	4.01
MKPPPRRPPGNISW	3.28
ELPRPRRPPPEDEE	1.57
FWQSPKRPPSPIKF	4.09
PTEGPRRPPPRPPC	3.48
QAAFPRRPPPRGTR	3.81
PPVVPRRPPAHISS	4.25
SQRRPRRPPTVFRL	4.04
LEESPWKPPTYLFS	1.73
KRVVPKKPPPSPQP	2.44
LILPPPRPPLAVTS	3.54
PPLLPPRPPQGLEP	2.52
YYLLPERPPYLDKY	2.03
GPYGPHRPPQPLPP	2.33
TPPIPSKPPGGFGK	3.00
PSQVPPRPPPPRLP	4.09
CSGVPGRPPGLEEE	1.76
AAESPGRPPPTAAE	1.71
SQALPPRPPPPAPV	3.41
NVPIPERPPPVPPP	1.92
PSPLPPKPPFPGPG	2.93
AVAIPNRPPDAVLT	1.90
DPNFPNKPPLTFDI	1.68
PPGPPTRPPGPWRW	3.97
EMEPPPKPPRSCSA	2.47
APQPPPRPPSPRYE	4.16
SGGRPIKPPKKDLE	1.82
SIHPPGRPPAVRGP	3.44
KTKGPSRPPLDAKI	3.35
GKVVPLRPPPPPKS	3.96

Yeast Myosin5 SH3 (1/1) [1139]

Peptide	LogBLU
DETRKLTPCSPGDD	2.36
LTIFKRPPKQPGHQ	2.85
IDKSKLSPRFPELK	2.37
TTTYKSRPDRPKPV	2.49
TELLRIIPEMPKDL	2.36
DIKTRSVPKIPSIE	2.01
NDLVKRFPEQPPLR	4.33
WLDEKRIPSTPVDY	2.38
PPRFKNFPTLPSKI	2.80
SLLAKYQPSLPLVE	2.21
NVLLKAKPNDPAAT	2.19
KIVERGPPYPPDLR	2.14
VSQRKKRPISPHLT	2.91
SMAMRPIPPLPTES	3.12
VKRPRLNPFLPRDE	3.07
SGSGRRYPQTPSYY	2.64
LGPLKYAPHLPFSK	2.63
PSHYRNIPYNPATF	2.24
GENIKITPQKPFWF	2.06
PHFAKHSPDSPRTS	2.37
CNRARYLPQNPDII	2.39
KLKIKILPEQPQLE	2.42
CAKSKSRPNTPGSD	2.18
FNGDRLVPTLPTVS	2.21
NESEKSSPWSPYKV	2.35
GFLSKKVPNTPVVV	1.85
IAVTRSIPRGPDKN	2.20
NELVKRFPGHPPLR	2.48
RRRTRLRPPTPLSQ	3.18
QSKTKPLPVEPASP	2.09
NGKDKSRPPRPPPK	3.42
TPQSRFPPYSPLLP	2.65
NSFRKILPQEPEIW	2.37
TILERLTPLVPNNL	2.40
KFFKREYPDIPMIA	2.19
LFGIRNNPFIPITG	2.23
FIKNKAKPLIPIEL	2.82
LSYLKDLPFVPFLF	2.33
YCPWKLCPLLPILY	2.25
VFYHRPAPKPPVTK	3.96
ILERKNIPRQPAIR	2.78
SKIKRSRPPPPPMD	3.73
ELDTKTIPELPFCM	1.73
DLQKKYEPSPPATH	2.35
GLDLKWEPSTPQRK	2.22
MDGIKELPEFPIKK	2.14
RLVSKYRPNCPIIL	2.46
ISNRRPVPRRPSQP	2.58
MKPQKIAPKFPGTV	2.62
LDTHKESPLRPKVR	2.30
YVVKKNFPVSPNYT	2.29
VRSKRLFPVRPMAT	2.70
LNGLKSSPADPHLS	2.18
KLHEKYFPDLPKEV	2.04
MQSKKEDPNRPKNL	2.19
DDIIKDHPHIPNGQ	1.76
MDMDRNVPLAPNLN	1.96
TNSVKATPVPPTLA	2.23
ATVVKNCPGDPELF	1.88
QFALKNTPITPGSC	2.05
GYNPKTVPFVPISG	2.04
MLNSKHYPYIPVAN	2.23
KTGRKTFPFLPYQI	2.67
PELAKNSPLAPIKK	2.10
ELQIRYKPTPPLKK	2.23
SKNWKLPPRLPHRA	3.75
HFNTKKIPELPSQF	2.67
WEALKSVPIPPRKN	2.58
DSYSRSRPTRPLGY	2.42
RMQQKNSPAFPTHS	3.11
VPIDKKSPFYPGLD	2.18
ASKHKMFPFNPAKI	2.98
ATEAKCLPKIPSIP	2.14
TASGKTSPILPLQN	2.46
IAEWKNDPNNPNFE	1.96
VQIKRADPHFPHDE	2.12
DEALRYCPDAPIVL	2.10
EIAKRLQPGSPVLG	2.24
NQIARQIPFQPWYL	2.39
RTFFKLPPNLPEAE	3.42
EKELKEGPSSPASI	2.32
ILAVKKNPQSPMYT	2.24
TTRRKKDPNAPKRR	3.00
PSSHKRKPVIPWDA	2.46
KYCFKLVPPQPGSK	2.34
LTLTKWDPSKPISL	2.36
HEASKCFPKAPQAS	2.46
DPSDKSLPQQPFHK	2.28
DIPRRYIPITPADL	1.52
IKYFKKFPKDPLAM	2.38
AKLHRTKPVKPLSK	2.70
CLRARNGPVPPVHI	2.32
RTPSKPDPMEPENT	2.27
INNNKMRPHLPPLS	3.00
TKKRKLGPKAPNPL	2.84
DFIGRKSPDLPEYL	2.14
SANGKFIPSRPAPK	3.83
KSIPRTKPNVPPLQ	2.54
VAPNRIPPKVPVIN	4.79
EWEEKLTPAGPLCP	2.47
VASSRTEPSTPSRP	2.25
RRHNRQHPPYPHSG	3.11
TEFSRPIPPTPSGF	2.21
EEQVKCNPNRPIYW	2.04
TEIYKLEPREPQVD	1.46
IYHCKKQPLVPIGC	2.47
AQKLKLLPEEPFQR	2.60
AKLRKFHPPEPYKG	2.08
LVGLKYEPLFPYFA	2.10
AGMAKTKPDVPLSY	2.20
TSNTKWTPITPSVI	2.15
SLIPKIEPAEPTYS	1.93
VIIGKTTPISPDEE	1.73
MRRCKDAPIEPPKY	2.19
HELVKSDPHFPYGT	2.36
KRRSKSLPTTPGIR	2.77
KRNSKHAPFIPVKP	2.58
NGTWKYNPTVPNEL	2.24
NITEKSPPKSPKHK	2.47
KKIAKGDPKIPMQN	2.86
EKYGKDQPESPVPE	2.16
KNLKKYDPEDPNRR	2.32
LSKVRGRPMYPRPA	2.88
CFKTKFAPEAPDLF	2.32
PNPLKKAPIPPLAY	2.14
YEGPRGAPGMPEML	2.22
GSQWKNHPDWPFWV	2.04
GIKVKRIPYSPKSI	2.59
NDDKKTEPDKPAFS	2.15
CKYNRQHPANPAAA	2.28
LNRKKVTPQLPKVT	2.70
KIIKKCSPSVPKEE	2.38
IDIPRTNPHIPLYQ	2.14
NSNVRIPPTVPSKI	2.63
PESAKCNPNSPRLK	2.40
EGRCRLFPHCPLGR	2.15
DKFIKLIPMRPVLI	2.40
KKKKKNGPGAPQGY	2.52
ILEGKFEPVIPREW	1.97
FRPHRLAPSAPATK	3.15
EVENKLRPDLPVKN	1.61
PRSKKKKPAPPTLL	2.62
DIPLKQVPENPMPT	1.80
GQQKRFSPSLPSAV	2.86
GRSLRKRPTSPSIS	2.91
VEKRKFTPIEPSLL	2.23
DLKKKYAPDPPITH	2.27
HILEKYSPFDPCGR	2.22
QSINRSIPSAPYGA	2.32
RLPSKTDPLDPHIK	2.11
KVQIKKVPTDPSSS	2.28
RKSDKNAPSVPQVD	2.60
EDLVKKPPFIPSPT	2.88
IAKLKRKPVVPRLK	2.95
EYSPRPSPWLPSKP	1.87
TDLGKDIPFFPSSE	2.17
TKRQRGEPSEPQQD	1.44
TGYPRYFPQPPVQR	3.77
IPAPKLVPPPPRTR	4.92
EKNARGIPQAPFIE	2.13
GSLVKTKPTLPLTK	2.63
YGSEKGSPAGPSAV	1.70
NTIIKFDPGRPIYN	2.43
KLYYRHRPQKPVRT	2.96
PVPVRMQPQPPQPM	2.27
ATKRKTIPVYPPVQ	2.44
QELPRAIPVMPFVD	2.07
VSKAKLQPAKPQFT	2.40
IRTYKVVPLGPKAG	2.00
IFVTRYLPYFPIMY	2.20
AALVKFVPSKPMCV	2.45
FLHSKKIPEVPQTD	2.46
NRANKYDPKGPIYN	2.31
NTVEKLQPGLPVII	2.45
TVCDRLIPTSPQKT	2.11
FENRKHQPFTPKGM	2.32
APPPKEEPAAPTST	2.07
GLNLKWEPSTPQRN	2.02
LLVTKERPRFPEDD	1.53
KKNDRPSPLKPIQH	2.53
AFWWKKNPKTPSDY	1.81
LGLHKWLPDIPLFE	2.22
FQYTKGRPLAPKGA	2.37
YAVDKIMPPTPAKK	2.04
KRRSKSLPITPKSI	2.96
TQPVRKNPEAPARR	2.37
KSLQKFEPRYPKTA	2.39
HRLFKCTPGLPKLW	2.42

Yeast Rvs167 SH3 (1/1) [1369]

Peptide	LogBLU
DETRKLTPCSPGDD	1.72
LTIFKRPPKQPGHQ	2.20
IDKSKLSPRFPELK	1.74
TTTYKSRPDRPKPV	1.99
TELLRIIPEMPKDL	1.36
DIKTRSVPKIPSIE	4.72
NDLVKRFPEQPPLR	4.36
WLDEKRIPSTPVDY	2.25
PPRFKNFPTLPSKI	2.32
SLLAKYQPSLPLVE	1.84
NVLLKAKPNDPAAT	2.08
KIVERGPPYPPDLR	2.01
VSQRKKRPISPHLT	1.49
SMAMRPIPPLPTES	4.51
VKRPRLNPFLPRDE	3.61
SGSGRRYPQTPSYY	2.14
LGPLKYAPHLPFSK	1.91
PSHYRNIPYNPATF	1.44
GENIKITPQKPFWF	1.87
PHFAKHSPDSPRTS	2.04
CNRARYLPQNPDII	1.56
KLKIKILPEQPQLE	1.76
CAKSKSRPNTPGSD	2.11
FNGDRLVPTLPTVS	3.36
NESEKSSPWSPYKV	1.87
GFLSKKVPNTPVVV	2.00
IAVTRSIPRGPDKN	2.45
NELVKRFPGHPPLR	1.74
RRRTRLRPPTPLSQ	2.63
QSKTKPLPVEPASP	1.95
NGKDKSRPPRPPPK	2.11
TPQSRFPPYSPLLP	1.45
NSFRKILPQEPEIW	2.72
TILERLTPLVPNNL	2.30
KFFKREYPDIPMIA	2.10
LFGIRNNPFIPITG	2.14
FIKNKAKPLIPIEL	2.98
LSYLKDLPFVPFLF	1.65
YCPWKLCPLLPILY	1.82
VFYHRPAPKPPVTK	1.96
ILERKNIPRQPAIR	2.14
SKIKRSRPPPPPMD	2.27
ELDTKTIPELPFCM	1.99
DLQKKYEPSPPATH	1.61
GLDLKWEPSTPQRK	1.86
MDGIKELPEFPIKK	1.51
RLVSKYRPNCPIIL	2.37
ISNRRPVPRRPSQP	4.91
MKPQKIAPKFPGTV	2.14
LDTHKESPLRPKVR	1.53
YVVKKNFPVSPNYT	1.53
VRSKRLFPVRPMAT	2.49
LNGLKSSPADPHLS	1.78
KLHEKYFPDLPKEV	1.76
MQSKKEDPNRPKNL	2.19
DDIIKDHPHIPNGQ	1.40
MDMDRNVPLAPNLN	1.49
TNSVKATPVPPTLA	1.54
ATVVKNCPGDPELF	1.46
QFALKNTPITPGSC	1.90
GYNPKTVPFVPISG	1.40
MLNSKHYPYIPVAN	2.57
KTGRKTFPFLPYQI	1.75
PELAKNSPLAPIKK	1.50
ELQIRYKPTPPLKK	2.27
SKNWKLPPRLPHRA	4.01
HFNTKKIPELPSQF	2.37
WEALKSVPIPPRKN	3.44
DSYSRSRPTRPLGY	2.55
RMQQKNSPAFPTHS	3.06
VPIDKKSPFYPGLD	1.77
ASKHKMFPFNPAKI	1.91
ATEAKCLPKIPSIP	1.68
TASGKTSPILPLQN	1.55
IAEWKNDPNNPNFE	1.31
VQIKRADPHFPHDE	1.80
DEALRYCPDAPIVL	1.52
EIAKRLQPGSPVLG	2.07
NQIARQIPFQPWYL	2.00
RTFFKLPPNLPEAE	2.31
EKELKEGPSSPASI	1.61
ILAVKKNPQSPMYT	1.42
TTRRKKDPNAPKRR	2.15
PSSHKRKPVIPWDA	1.72
KYCFKLVPPQPGSK	1.61
LTLTKWDPSKPISL	1.09
HEASKCFPKAPQAS	1.13
DPSDKSLPQQPFHK	1.74
DIPRRYIPITPADL	2.98
IKYFKKFPKDPLAM	1.93
AKLHRTKPVKPLSK	1.54
CLRARNGPVPPVHI	1.66
RTPSKPDPMEPENT	1.29
INNNKMRPHLPPLS	3.19
TKKRKLGPKAPNPL	2.19
DFIGRKSPDLPEYL	2.21
SANGKFIPSRPAPK	2.17
KSIPRTKPNVPPLQ	1.60
VAPNRIPPKVPVIN	1.75
EWEEKLTPAGPLCP	1.67
VASSRTEPSTPSRP	1.88
RRHNRQHPPYPHSG	1.71
TEFSRPIPPTPSGF	4.54
EEQVKCNPNRPIYW	1.12
TEIYKLEPREPQVD	1.99
IYHCKKQPLVPIGC	2.20
AQKLKLLPEEPFQR	1.89
AKLRKFHPPEPYKG	1.69
LVGLKYEPLFPYFA	1.55
AGMAKTKPDVPLSY	1.79
TSNTKWTPITPSVI	1.54
SLIPKIEPAEPTYS	1.62
VIIGKTTPISPDEE	1.52
MRRCKDAPIEPPKY	3.08
HELVKSDPHFPYGT	1.48
KRRSKSLPTTPGIR	1.25
KRNSKHAPFIPVKP	1.84
NGTWKYNPTVPNEL	1.94
NITEKSPPKSPKHK	2.09
KKIAKGDPKIPMQN	2.17
EKYGKDQPESPVPE	1.62
KNLKKYDPEDPNRR	2.08
LSKVRGRPMYPRPA	1.97
CFKTKFAPEAPDLF	1.67
PNPLKKAPIPPLAY	1.46
YEGPRGAPGMPEML	1.80
GSQWKNHPDWPFWV	1.67
GIKVKRIPYSPKSI	1.76
NDDKKTEPDKPAFS	1.68
CKYNRQHPANPAAA	1.79
LNRKKVTPQLPKVT	1.88
KIIKKCSPSVPKEE	2.25
IDIPRTNPHIPLYQ	0.96
NSNVRIPPTVPSKI	2.19
PESAKCNPNSPRLK	1.91
EGRCRLFPHCPLGR	2.59
DKFIKLIPMRPVLI	1.59
KKKKKNGPGAPQGY	1.37
ILEGKFEPVIPREW	1.90
FRPHRLAPSAPATK	2.36
EVENKLRPDLPVKN	1.64
PRSKKKKPAPPTLL	1.70
DIPLKQVPENPMPT	1.41
GQQKRFSPSLPSAV	2.04
GRSLRKRPTSPSIS	3.05
VEKRKFTPIEPSLL	2.35
DLKKKYAPDPPITH	1.44
HILEKYSPFDPCGR	1.20
QSINRSIPSAPYGA	1.95
RLPSKTDPLDPHIK	2.04
KVQIKKVPTDPSSS	1.65
RKSDKNAPSVPQVD	1.61
EDLVKKPPFIPSPT	1.13
IAKLKRKPVVPRLK	1.47
EYSPRPSPWLPSKP	1.74
TDLGKDIPFFPSSE	1.77
TKRQRGEPSEPQQD	2.28
TGYPRYFPQPPVQR	2.42
IPAPKLVPPPPRTR	2.47
EKNARGIPQAPFIE	1.52
GSLVKTKPTLPLTK	1.32
YGSEKGSPAGPSAV	1.46
NTIIKFDPGRPIYN	1.86
KLYYRHRPQKPVRT	2.18
PVPVRMQPQPPQPM	1.85
ATKRKTIPVYPPVQ	1.70
QELPRAIPVMPFVD	1.76
VSKAKLQPAKPQFT	1.45
IRTYKVVPLGPKAG	1.59
IFVTRYLPYFPIMY	2.57
AALVKFVPSKPMCV	2.02
FLHSKKIPEVPQTD	1.18
NRANKYDPKGPIYN	2.04
NTVEKLQPGLPVII	1.22
TVCDRLIPTSPQKT	2.16
FENRKHQPFTPKGM	2.17
APPPKEEPAAPTST	1.20
GLNLKWEPSTPQRN	1.04
LLVTKERPRFPEDD	1.21
KKNDRPSPLKPIQH	1.69
AFWWKKNPKTPSDY	1.86
LGLHKWLPDIPLFE	1.22
FQYTKGRPLAPKGA	1.35
YAVDKIMPPTPAKK	1.85
KRRSKSLPITPKSI	2.10
TQPVRKNPEAPARR	2.61
KSLQKFEPRYPKTA	2.05
HRLFKCTPGLPKLW	2.78

Yeast Sho1 SH3 (1/1) [1015]

Peptide	LogBLU
DETRKLTPCSPGDD	1.53
LTIFKRPPKQPGHQ	1.97
IDKSKLSPRFPELK	1.55
TTTYKSRPDRPKPV	1.65
TELLRIIPEMPKDL	1.99
DIKTRSVPKIPSIE	1.50
NDLVKRFPEQPPLR	4.08
WLDEKRIPSTPVDY	2.28
PPRFKNFPTLPSKI	3.58
SLLAKYQPSLPLVE	1.77
NVLLKAKPNDPAAT	2.10
KIVERGPPYPPDLR	1.17
VSQRKKRPISPHLT	1.77
SMAMRPIPPLPTES	2.38
VKRPRLNPFLPRDE	2.07
SGSGRRYPQTPSYY	2.22
LGPLKYAPHLPFSK	2.36
PSHYRNIPYNPATF	1.81
GENIKITPQKPFWF	1.66
PHFAKHSPDSPRTS	1.40
CNRARYLPQNPDII	1.55
KLKIKILPEQPQLE	2.74
CAKSKSRPNTPGSD	2.16
FNGDRLVPTLPTVS	1.60
NESEKSSPWSPYKV	1.42
GFLSKKVPNTPVVV	1.68
IAVTRSIPRGPDKN	1.81
NELVKRFPGHPPLR	1.57
RRRTRLRPPTPLSQ	1.88
QSKTKPLPVEPASP	3.99
NGKDKSRPPRPPPK	3.42
TPQSRFPPYSPLLP	1.54
NSFRKILPQEPEIW	3.26
TILERLTPLVPNNL	1.60
KFFKREYPDIPMIA	2.56
LFGIRNNPFIPITG	1.20
FIKNKAKPLIPIEL	2.61
LSYLKDLPFVPFLF	4.36
YCPWKLCPLLPILY	1.80
VFYHRPAPKPPVTK	2.43
ILERKNIPRQPAIR	2.00
SKIKRSRPPPPPMD	2.22
ELDTKTIPELPFCM	1.64
DLQKKYEPSPPATH	1.51
GLDLKWEPSTPQRK	1.80
MDGIKELPEFPIKK	3.58
RLVSKYRPNCPIIL	1.63
ISNRRPVPRRPSQP	2.11
MKPQKIAPKFPGTV	2.15
LDTHKESPLRPKVR	1.67
YVVKKNFPVSPNYT	2.52
VRSKRLFPVRPMAT	2.39
LNGLKSSPADPHLS	1.31
KLHEKYFPDLPKEV	1.48
MQSKKEDPNRPKNL	1.65
DDIIKDHPHIPNGQ	1.37
MDMDRNVPLAPNLN	1.49
TNSVKATPVPPTLA	1.70
ATVVKNCPGDPELF	1.71
QFALKNTPITPGSC	1.56
GYNPKTVPFVPISG	1.40
MLNSKHYPYIPVAN	1.54
KTGRKTFPFLPYQI	2.15
PELAKNSPLAPIKK	1.74
ELQIRYKPTPPLKK	1.88
SKNWKLPPRLPHRA	3.69
HFNTKKIPELPSQF	3.29
WEALKSVPIPPRKN	3.52
DSYSRSRPTRPLGY	1.72
RMQQKNSPAFPTHS	3.08
VPIDKKSPFYPGLD	1.32
ASKHKMFPFNPAKI	1.82
ATEAKCLPKIPSIP	4.28
TASGKTSPILPLQN	-1.00
IAEWKNDPNNPNFE	1.41
VQIKRADPHFPHDE	1.49
DEALRYCPDAPIVL	1.69
EIAKRLQPGSPVLG	1.34
NQIARQIPFQPWYL	1.88
RTFFKLPPNLPEAE	2.19
EKELKEGPSSPASI	1.79
ILAVKKNPQSPMYT	1.41
TTRRKKDPNAPKRR	2.03
PSSHKRKPVIPWDA	1.90
KYCFKLVPPQPGSK	1.85
LTLTKWDPSKPISL	1.43
HEASKCFPKAPQAS	1.85
DPSDKSLPQQPFHK	2.60
DIPRRYIPITPADL	1.53
IKYFKKFPKDPLAM	2.44
AKLHRTKPVKPLSK	2.04
CLRARNGPVPPVHI	1.77
RTPSKPDPMEPENT	1.26
INNNKMRPHLPPLS	2.81
TKKRKLGPKAPNPL	1.40
DFIGRKSPDLPEYL	1.24
SANGKFIPSRPAPK	1.88
KSIPRTKPNVPPLQ	1.67
VAPNRIPPKVPVIN	1.28
EWEEKLTPAGPLCP	1.58
VASSRTEPSTPSRP	1.80
RRHNRQHPPYPHSG	1.81
TEFSRPIPPTPSGF	1.94
EEQVKCNPNRPIYW	1.39
TEIYKLEPREPQVD	1.46
IYHCKKQPLVPIGC	2.16
AQKLKLLPEEPFQR	3.58
AKLRKFHPPEPYKG	2.12
LVGLKYEPLFPYFA	1.88
AGMAKTKPDVPLSY	1.72
TSNTKWTPITPSVI	1.27
SLIPKIEPAEPTYS	1.52
VIIGKTTPISPDEE	1.56
MRRCKDAPIEPPKY	2.17
HELVKSDPHFPYGT	1.07
KRRSKSLPTTPGIR	4.47
KRNSKHAPFIPVKP	1.94
NGTWKYNPTVPNEL	0.57
NITEKSPPKSPKHK	1.87
KKIAKGDPKIPMQN	2.01
EKYGKDQPESPVPE	1.82
KNLKKYDPEDPNRR	1.88
LSKVRGRPMYPRPA	1.92
CFKTKFAPEAPDLF	1.55
PNPLKKAPIPPLAY	1.31
YEGPRGAPGMPEML	1.60
GSQWKNHPDWPFWV	1.16
GIKVKRIPYSPKSI	1.18
NDDKKTEPDKPAFS	1.41
CKYNRQHPANPAAA	1.82
LNRKKVTPQLPKVT	2.42
KIIKKCSPSVPKEE	3.09
IDIPRTNPHIPLYQ	1.61
NSNVRIPPTVPSKI	3.16
PESAKCNPNSPRLK	1.84
EGRCRLFPHCPLGR	1.62
DKFIKLIPMRPVLI	2.05
KKKKKNGPGAPQGY	2.03
ILEGKFEPVIPREW	0.98
FRPHRLAPSAPATK	1.65
EVENKLRPDLPVKN	1.59
PRSKKKKPAPPTLL	1.76
DIPLKQVPENPMPT	1.18
GQQKRFSPSLPSAV	1.67
GRSLRKRPTSPSIS	1.65
VEKRKFTPIEPSLL	1.55
DLKKKYAPDPPITH	0.62
HILEKYSPFDPCGR	1.95
QSINRSIPSAPYGA	1.65
RLPSKTDPLDPHIK	1.68
KVQIKKVPTDPSSS	1.45
RKSDKNAPSVPQVD	1.95
EDLVKKPPFIPSPT	1.25
IAKLKRKPVVPRLK	1.76
EYSPRPSPWLPSKP	1.54
TDLGKDIPFFPSSE	1.69
TKRQRGEPSEPQQD	1.64
TGYPRYFPQPPVQR	1.65
IPAPKLVPPPPRTR	2.00
EKNARGIPQAPFIE	1.38
GSLVKTKPTLPLTK	1.36
YGSEKGSPAGPSAV	1.67
NTIIKFDPGRPIYN	2.16
KLYYRHRPQKPVRT	2.10
PVPVRMQPQPPQPM	1.86
ATKRKTIPVYPPVQ	1.48
QELPRAIPVMPFVD	1.06
VSKAKLQPAKPQFT	1.79
IRTYKVVPLGPKAG	1.79
IFVTRYLPYFPIMY	2.06
AALVKFVPSKPMCV	1.51
FLHSKKIPEVPQTD	3.07
NRANKYDPKGPIYN	1.34
NTVEKLQPGLPVII	0.45
TVCDRLIPTSPQKT	1.78
FENRKHQPFTPKGM	2.10
APPPKEEPAAPTST	1.58
GLNLKWEPSTPQRN	1.80
LLVTKERPRFPEDD	1.68
KKNDRPSPLKPIQH	1.83
AFWWKKNPKTPSDY	2.95
LGLHKWLPDIPLFE	4.03
FQYTKGRPLAPKGA	1.83
YAVDKIMPPTPAKK	2.05
KRRSKSLPITPKSI	4.29
TQPVRKNPEAPARR	1.78
KSLQKFEPRYPKTA	1.59
HRLFKCTPGLPKLW	2.81

Yeast Yfr024 SH3 (1/1) [1282]

Peptide	LogBLU
DETRKLTPCSPGDD	1.79
LTIFKRPPKQPGHQ	3.01
IDKSKLSPRFPELK	2.12
TTTYKSRPDRPKPV	2.42
TELLRIIPEMPKDL	2.12
DIKTRSVPKIPSIE	4.68
NDLVKRFPEQPPLR	3.20
WLDEKRIPSTPVDY	3.49
PPRFKNFPTLPSKI	3.41
SLLAKYQPSLPLVE	1.76
NVLLKAKPNDPAAT	1.80
KIVERGPPYPPDLR	1.97
VSQRKKRPISPHLT	2.48
SMAMRPIPPLPTES	4.70
VKRPRLNPFLPRDE	3.38
SGSGRRYPQTPSYY	2.81
LGPLKYAPHLPFSK	2.33
PSHYRNIPYNPATF	2.00
GENIKITPQKPFWF	1.47
PHFAKHSPDSPRTS	1.52
CNRARYLPQNPDII	2.48
KLKIKILPEQPQLE	0.78
CAKSKSRPNTPGSD	2.09
FNGDRLVPTLPTVS	3.34
NESEKSSPWSPYKV	2.61
GFLSKKVPNTPVVV	1.91
IAVTRSIPRGPDKN	2.74
NELVKRFPGHPPLR	2.32
RRRTRLRPPTPLSQ	2.91
QSKTKPLPVEPASP	1.81
NGKDKSRPPRPPPK	4.11
TPQSRFPPYSPLLP	1.53
NSFRKILPQEPEIW	3.11
TILERLTPLVPNNL	2.30
KFFKREYPDIPMIA	2.08
LFGIRNNPFIPITG	2.53
FIKNKAKPLIPIEL	2.17
LSYLKDLPFVPFLF	2.10
YCPWKLCPLLPILY	2.71
VFYHRPAPKPPVTK	2.19
ILERKNIPRQPAIR	2.55
SKIKRSRPPPPPMD	3.16
ELDTKTIPELPFCM	1.44
GLDLKWEPSTPQRK	3.00
MDGIKELPEFPIKK	2.60
RLVSKYRPNCPIIL	2.75
ISNRRPVPRRPSQP	4.57
MKPQKIAPKFPGTV	2.60
LDTHKESPLRPKVR	1.77
YVVKKNFPVSPNYT	2.21
VRSKRLFPVRPMAT	3.42
LNGLKSSPADPHLS	0.38
KLHEKYFPDLPKEV	1.49
MQSKKEDPNRPKNL	2.86
DDIIKDHPHIPNGQ	1.53
MDMDRNVPLAPNLN	2.68
TNSVKATPVPPTLA	0.88
ATVVKNCPGDPELF	1.44
QFALKNTPITPGSC	1.83
GYNPKTVPFVPISG	1.12
MLNSKHYPYIPVAN	2.77
KTGRKTFPFLPYQI	2.23
PELAKNSPLAPIKK	1.84
ELQIRYKPTPPLKK	2.97
SKNWKLPPRLPHRA	4.59
HFNTKKIPELPSQF	2.31
WEALKSVPIPPRKN	3.98
DSYSRSRPTRPLGY	3.35
RMQQKNSPAFPTHS	3.66
VPIDKKSPFYPGLD	1.51
ASKHKMFPFNPAKI	2.33
ATEAKCLPKIPSIP	1.60
TASGKTSPILPLQN	0.41
VQIKRADPHFPHDE	1.64
DEALRYCPDAPIVL	1.71
EIAKRLQPGSPVLG	2.10
NQIARQIPFQPWYL	2.49
RTFFKLPPNLPEAE	3.02
ILAVKKNPQSPMYT	1.84
TTRRKKDPNAPKRR	2.53
PSSHKRKPVIPWDA	1.82
KYCFKLVPPQPGSK	2.02
LTLTKWDPSKPISL	1.82
HEASKCFPKAPQAS	1.44
DPSDKSLPQQPFHK	1.59
DIPRRYIPITPADL	2.87
IKYFKKFPKDPLAM	2.48
AKLHRTKPVKPLSK	2.21
CLRARNGPVPPVHI	2.20
RTPSKPDPMEPENT	1.90
INNNKMRPHLPPLS	3.69
TKKRKLGPKAPNPL	2.51
DFIGRKSPDLPEYL	1.77
SANGKFIPSRPAPK	2.73
KSIPRTKPNVPPLQ	1.80
VAPNRIPPKVPVIN	2.33
EWEEKLTPAGPLCP	1.91
VASSRTEPSTPSRP	3.56
RRHNRQHPPYPHSG	2.82
TEFSRPIPPTPSGF	4.14
EEQVKCNPNRPIYW	2.15
IYHCKKQPLVPIGC	2.28
AQKLKLLPEEPFQR	1.46
AKLRKFHPPEPYKG	2.11
LVGLKYEPLFPYFA	1.43
AGMAKTKPDVPLSY	1.94
TSNTKWTPITPSVI	1.57
SLIPKIEPAEPTYS	0.00
MRRCKDAPIEPPKY	3.36
HELVKSDPHFPYGT	0.40
KRRSKSLPTTPGIR	2.75
KRNSKHAPFIPVKP	2.88
NGTWKYNPTVPNEL	2.58
NITEKSPPKSPKHK	2.27
KKIAKGDPKIPMQN	2.28
KNLKKYDPEDPNRR	1.66
LSKVRGRPMYPRPA	2.53
CFKTKFAPEAPDLF	1.37
PNPLKKAPIPPLAY	1.18
YEGPRGAPGMPEML	2.12
GSQWKNHPDWPFWV	1.84
GIKVKRIPYSPKSI	2.25
NDDKKTEPDKPAFS	1.80
CKYNRQHPANPAAA	2.10
LNRKKVTPQLPKVT	2.15
KIIKKCSPSVPKEE	2.28
IDIPRTNPHIPLYQ	1.54
NSNVRIPPTVPSKI	3.18
PESAKCNPNSPRLK	1.95
EGRCRLFPHCPLGR	3.35
DKFIKLIPMRPVLI	3.14
KKKKKNGPGAPQGY	2.20
ILEGKFEPVIPREW	1.92
FRPHRLAPSAPATK	3.00
EVENKLRPDLPVKN	3.13
PRSKKKKPAPPTLL	2.19
DIPLKQVPENPMPT	1.13
GQQKRFSPSLPSAV	2.84
GRSLRKRPTSPSIS	2.64
VEKRKFTPIEPSLL	2.25
DLKKKYAPDPPITH	1.22
HILEKYSPFDPCGR	1.92
QSINRSIPSAPYGA	2.25
RLPSKTDPLDPHIK	1.82
KVQIKKVPTDPSSS	1.00
RKSDKNAPSVPQVD	1.14
EYSPRPSPWLPSKP	2.82
TDLGKDIPFFPSSE	0.98
TKRQRGEPSEPQQD	2.20
TGYPRYFPQPPVQR	2.62
IPAPKLVPPPPRTR	3.51
EKNARGIPQAPFIE	2.31
GSLVKTKPTLPLTK	2.71
YGSEKGSPAGPSAV	1.13
NTIIKFDPGRPIYN	1.80
KLYYRHRPQKPVRT	2.88
PVPVRMQPQPPQPM	3.22
ATKRKTIPVYPPVQ	2.31
QELPRAIPVMPFVD	2.84
VSKAKLQPAKPQFT	1.98
IRTYKVVPLGPKAG	2.06
IFVTRYLPYFPIMY	2.83
AALVKFVPSKPMCV	2.26
FLHSKKIPEVPQTD	1.18
NRANKYDPKGPIYN	1.76
NTVEKLQPGLPVII	1.53
TVCDRLIPTSPQKT	1.88
FENRKHQPFTPKGM	2.76
APPPKEEPAAPTST	0.76
GLNLKWEPSTPQRN	3.01
LLVTKERPRFPEDD	2.23
KKNDRPSPLKPIQH	2.41
AFWWKKNPKTPSDY	2.48
LGLHKWLPDIPLFE	1.69
FQYTKGRPLAPKGA	2.28
YAVDKIMPPTPAKK	2.71
KRRSKSLPITPKSI	2.75
TQPVRKNPEAPARR	3.57
KSLQKFEPRYPKTA	2.13
HRLFKCTPGLPKLW	3.45
ADDLKYIPPIPQDF	0.78
RLLFRSLPPHPACL	3.54
LIWHKSKPVKPTNK	3.12
DDNVRPSPISPSIN	2.08
RRIRKEHPGEPNVI	2.76
PEWRRPHPNIPDAK	3.06
DKKKKKYPRKPKKN	2.39
GFQDRSLPHFPKNS	2.25

Yeast Yhr016c SH3 (1/1) [1348]

Peptide	LogBLU
DETRKLTPCSPGDD	1.28
LTIFKRPPKQPGHQ	2.86
IDKSKLSPRFPELK	1.35
TTTYKSRPDRPKPV	1.84
TELLRIIPEMPKDL	1.29
DIKTRSVPKIPSIE	4.56
NDLVKRFPEQPPLR	3.16
WLDEKRIPSTPVDY	2.44
PPRFKNFPTLPSKI	1.94
SLLAKYQPSLPLVE	1.67
NVLLKAKPNDPAAT	2.49
KIVERGPPYPPDLR	1.31
VSQRKKRPISPHLT	1.01
SMAMRPIPPLPTES	4.67
VKRPRLNPFLPRDE	2.54
SGSGRRYPQTPSYY	2.35
LGPLKYAPHLPFSK	2.18
PSHYRNIPYNPATF	1.05
GENIKITPQKPFWF	1.52
PHFAKHSPDSPRTS	1.44
CNRARYLPQNPDII	1.67
KLKIKILPEQPQLE	1.25
FNGDRLVPTLPTVS	2.88
NESEKSSPWSPYKV	1.48
GFLSKKVPNTPVVV	1.39
IAVTRSIPRGPDKN	1.64
NELVKRFPGHPPLR	2.00
RRRTRLRPPTPLSQ	2.02
QSKTKPLPVEPASP	1.82
NGKDKSRPPRPPPK	2.21
TPQSRFPPYSPLLP	1.53
NSFRKILPQEPEIW	3.10
TILERLTPLVPNNL	1.51
KFFKREYPDIPMIA	1.63
LFGIRNNPFIPITG	2.63
FIKNKAKPLIPIEL	1.86
LSYLKDLPFVPFLF	1.27
YCPWKLCPLLPILY	1.61
VFYHRPAPKPPVTK	1.98
ILERKNIPRQPAIR	1.55
SKIKRSRPPPPPMD	2.09
ELDTKTIPELPFCM	1.11
DLQKKYEPSPPATH	1.57
GLDLKWEPSTPQRK	2.53
MDGIKELPEFPIKK	1.61
RLVSKYRPNCPIIL	2.03
ISNRRPVPRRPSQP	4.23
MKPQKIAPKFPGTV	1.61
LDTHKESPLRPKVR	1.52
YVVKKNFPVSPNYT	1.60
VRSKRLFPVRPMAT	2.37
LNGLKSSPADPHLS	1.38
KLHEKYFPDLPKEV	0.65
MQSKKEDPNRPKNL	2.10
DDIIKDHPHIPNGQ	1.87
MDMDRNVPLAPNLN	1.89
TNSVKATPVPPTLA	1.37
ATVVKNCPGDPELF	1.12
QFALKNTPITPGSC	1.53
GYNPKTVPFVPISG	1.57
MLNSKHYPYIPVAN	2.11
KTGRKTFPFLPYQI	1.42
PELAKNSPLAPIKK	1.16
ELQIRYKPTPPLKK	1.63
SKNWKLPPRLPHRA	4.34
HFNTKKIPELPSQF	0.69
WEALKSVPIPPRKN	3.27
DSYSRSRPTRPLGY	2.25
RMQQKNSPAFPTHS	3.18
VPIDKKSPFYPGLD	1.32
ASKHKMFPFNPAKI	1.21
ATEAKCLPKIPSIP	1.83
TASGKTSPILPLQN	0.71
IAEWKNDPNNPNFE	0.98
VQIKRADPHFPHDE	1.62
DEALRYCPDAPIVL	1.36
EIAKRLQPGSPVLG	1.77
NQIARQIPFQPWYL	1.70
RTFFKLPPNLPEAE	1.52
EKELKEGPSSPASI	1.45
ILAVKKNPQSPMYT	1.35
TTRRKKDPNAPKRR	1.29
PSSHKRKPVIPWDA	0.93
KYCFKLVPPQPGSK	1.47
LTLTKWDPSKPISL	1.32
HEASKCFPKAPQAS	1.42
DPSDKSLPQQPFHK	1.56
DIPRRYIPITPADL	2.07
IKYFKKFPKDPLAM	1.77
AKLHRTKPVKPLSK	1.63
CLRARNGPVPPVHI	0.96
RTPSKPDPMEPENT	1.32
INNNKMRPHLPPLS	3.09
TKKRKLGPKAPNPL	1.10
DFIGRKSPDLPEYL	1.57
SANGKFIPSRPAPK	1.17
KSIPRTKPNVPPLQ	1.44
VAPNRIPPKVPVIN	1.25
EWEEKLTPAGPLCP	1.51
VASSRTEPSTPSRP	2.78
RRHNRQHPPYPHSG	1.64
TEFSRPIPPTPSGF	3.98
EEQVKCNPNRPIYW	1.48
TEIYKLEPREPQVD	1.81
IYHCKKQPLVPIGC	1.94
AQKLKLLPEEPFQR	0.53
AKLRKFHPPEPYKG	1.51
LVGLKYEPLFPYFA	1.40
AGMAKTKPDVPLSY	1.44
TSNTKWTPITPSVI	1.32
SLIPKIEPAEPTYS	0.94
VIIGKTTPISPDEE	0.80
MRRCKDAPIEPPKY	3.02
HELVKSDPHFPYGT	1.23
KRRSKSLPTTPGIR	1.54
KRNSKHAPFIPVKP	2.18
NGTWKYNPTVPNEL	1.36
NITEKSPPKSPKHK	2.04
KKIAKGDPKIPMQN	1.95
EKYGKDQPESPVPE	0.20
KNLKKYDPEDPNRR	1.85
LSKVRGRPMYPRPA	1.72
CFKTKFAPEAPDLF	1.28
PNPLKKAPIPPLAY	1.21
YEGPRGAPGMPEML	1.19
GSQWKNHPDWPFWV	1.27
GIKVKRIPYSPKSI	1.20
NDDKKTEPDKPAFS	1.57
CKYNRQHPANPAAA	1.59
LNRKKVTPQLPKVT	1.36
KIIKKCSPSVPKEE	1.08
IDIPRTNPHIPLYQ	0.00
NSNVRIPPTVPSKI	2.05
PESAKCNPNSPRLK	1.50
EGRCRLFPHCPLGR	1.96
DKFIKLIPMRPVLI	1.98
KKKKKNGPGAPQGY	1.75
ILEGKFEPVIPREW	1.61
FRPHRLAPSAPATK	2.04
EVENKLRPDLPVKN	1.17
PRSKKKKPAPPTLL	1.57
DIPLKQVPENPMPT	0.66
GQQKRFSPSLPSAV	2.14
GRSLRKRPTSPSIS	2.02
VEKRKFTPIEPSLL	1.85
DLKKKYAPDPPITH	1.16
HILEKYSPFDPCGR	1.53
QSINRSIPSAPYGA	1.63
RLPSKTDPLDPHIK	1.47
KVQIKKVPTDPSSS	1.13
RKSDKNAPSVPQVD	1.70
EDLVKKPPFIPSPT	0.32
IAKLKRKPVVPRLK	1.94
EYSPRPSPWLPSKP	2.03
TDLGKDIPFFPSSE	1.13
TKRQRGEPSEPQQD	1.64
TGYPRYFPQPPVQR	1.56
IPAPKLVPPPPRTR	2.22
EKNARGIPQAPFIE	1.63
GSLVKTKPTLPLTK	1.91
YGSEKGSPAGPSAV	0.94
NTIIKFDPGRPIYN	1.02
KLYYRHRPQKPVRT	1.94
PVPVRMQPQPPQPM	2.16
ATKRKTIPVYPPVQ	1.45
QELPRAIPVMPFVD	2.07
VSKAKLQPAKPQFT	1.70
IRTYKVVPLGPKAG	1.65
IFVTRYLPYFPIMY	2.26
AALVKFVPSKPMCV	1.74
FLHSKKIPEVPQTD	1.41
NRANKYDPKGPIYN	1.89
NTVEKLQPGLPVII	1.08
TVCDRLIPTSPQKT	1.11
FENRKHQPFTPKGM	1.52
APPPKEEPAAPTST	-0.22
GLNLKWEPSTPQRN	2.18
LLVTKERPRFPEDD	1.45
KKNDRPSPLKPIQH	1.65
AFWWKKNPKTPSDY	2.05
LGLHKWLPDIPLFE	1.65
FQYTKGRPLAPKGA	1.63
YAVDKIMPPTPAKK	1.34
KRRSKSLPITPKSI	1.76
TQPVRKNPEAPARR	2.20
KSLQKFEPRYPKTA	1.71
HRLFKCTPGLPKLW	1.99
ADDLKYIPPIPQDF	1.21

FSPSKPPSGP	1.72
HPPAKPPLPP	1.81
APPHKPPNTR	1.68
PVPARPPPPT	2.18
IGPHRPPELH	1.82
DPPQKPPRSI	3.92
QVPERPPKPF	3.23
SRPRKPPPRK	3.57
PVPHRPPFEP	1.81
GSPEKPPSRR	2.91
TLPPKPPSPR	2.76
GGPQKPPPHT	1.60
PGPAKPPAHR	1.60
GKPERPPKPG	2.76
SPPPRPPPPA	3.15
ALPPKPPKAK	2.09
PRPARPPSPT	2.51
GSPEKPPEDD	1.56
IVPKKPPLLD	1.54
VKPFKPPLSD	1.62
GLPPRPPLSS	2.64
FPPPRPPPPS	3.21
GPPPRPPPRQ	4.25
DLPKKPPKPE	1.21
SRPVKPPKKD	1.72
PGPAKPPAQP	1.89
QGPAKPPPPR	3.12
RRPPRPPGSS	3.50
IRPLRPPQRA	4.29
SKPVRPPAPG	1.81
PPPERPPPIP	1.46
LPPPRPPRPP	3.75
PVPERPPEPR	1.20
EFPFKPPSIY	1.51
SRPSRPPSHP	4.17
CKIFRPPRAS	3.25
QTPERPAKKI	2.45
GIFTRPSKLT	2.51
FHILRPTKAP	2.29
NALIRPGRFD	2.38
GGLLRPERDH	1.90
RIPTRPPRAP	4.40
NAPCRPLRRR	4.28
GLLARPAKLQ	1.99
HAPGRPARGK	3.59
RPILRPRKYP	3.32
SEFDRPLRPI	2.12
SGPSRPGREE	3.40
GQPSRPTKSK	1.91
ILPPRPPKVL	2.97
TALLRPLRPK	2.17
QNFHRPVKVR	1.72
QALGRPEKIH	1.96
SFLARPSRKN	3.38
PIPPRPVKPD	2.81
VPLLRPHRPS	3.13
QLLLRPERPG	1.84
TKLVRPKRKH	3.38
KAFCRPDKLR	2.07
GNLPRPGRGA	2.98
HVPARPYRVL	3.61
KKPPRPPKKK	1.75
ARPARPPRVR	4.21
SSPARPSKHS	2.36
HVISRPLKAP	1.92
IRLPRPPKVL	3.29
PTLERPGRKR	2.70
ANLERPVRYI	3.49
PHLARPIKVE	2.16
WRPWRPDKTK	2.76
FIISRPTKLK	2.03
ANIQRPCRKS	3.51
GRIGRPPKYR	2.21
KRPGRPPKNR	3.88
KYPNRPSKTP	2.04
PKLDRPTKQM	2.06
MGFNRPSKIQ	2.38
TQPLRPPRGT	4.08
ARLSRPERPD	3.15
KVFHRPWRRG	2.90
VDPQRPPRPE	3.44
GFPGRPGRPG	2.14
PSLPRPERKS	2.18
RPPRRPRKPK	3.89
SFIIRPQKEG	2.07
KVFFRPGKFA	2.12
LAICRPLRAR	3.12
YMFSRPFRKH	3.34
LRPLRPCRRY	4.00
EPLHRPKKEL	2.13
WCPMRPKKEA	2.23
VDPRRPFRKL	3.47
AYPNRPDRPV	3.11
QLLQRPPRDA	3.51
VNPPRPFKHS	2.44
RYLCRPRRVE	2.53
ADLIRPKRKY	2.62
AHLIRPRRLT	3.03
PEPPRPAKRP	2.79
LYFCRPFREN	3.35
ISFPRPVKTT	2.30
IMFNRPKKKN	1.91
ARLKRPLKSL	2.07
YSPYRPYRCV	3.00
GLLVRPSRVR	3.41
PRLARPPRFY	2.85
PAILRPGRLD	2.48
QTLPRPMKTL	1.90
FFPPRPERVE	2.68
DDFYRPSREQ	2.15
ARLGRPGRSC	2.11
PSPARPARTQ	3.63
KDPRRPKRPR	3.57
LTLPRPVRAS	2.60
PPPPRPDRGP	2.93
FVPFRPRRSP	4.30
HTPGRPPRVT	3.29
GPPPRPGKPE	2.02
KYLCRPRRVE	2.41
TNPFRPQKVC	1.89
RSLHRPAKTS	2.23
PLLKRPVRTQ	3.22
IIISRPARLL	1.96
AKISRPKKRA	2.09
SVILRPSKSV	2.08
LDIQRPKRKR	3.33
RPLARPARPN	3.48
PDFSRPNRKY	3.08
PGFLRPQRAA	2.99
PLFVRPPKGS	2.33
TAPRRPQRYS	4.21
QVPSRPTRAP	3.94
KAFIRPFREH	4.07
FSIVRPRRLL	3.14
GIFTRPSKLT	2.08
QAPDRPSREL	3.52
QDLPRPLRSR	2.19
EGINRPRKGH	2.00
PEPPRPRRGS	3.55
PALLRPGRLD	3.31
WYPLRPKKIN	2.25
KKPSRPAKTR	2.04
EMLPRPPKVT	2.01
EQLPRPTRGR	2.94
LQFPRPVKLE	2.03
PNIGRPRKNV	2.13
VTLQRPTKEL	1.98
TGLSRPRRTA	2.87
VEFFRPPKEV	2.22
LEIVRPARRR	2.28
PPPSRPEKKK	1.90
AHLVRPQKCR	2.22
KILNRPKRYN	2.68
NCLTRPQKLS	2.08
QSIYRPSKNL	2.19
RLLYRPNKAG	1.30
EGPARPYRLK	3.10
SDPRRPNKVL	2.81
QRPGRPARVA	3.35
LSPLRPSRRH	3.95
YLLFRPFKGY	2.11
RFLPRPIRQQ	3.24
SALLRPGRID	3.21
ICFLRPTKEN	1.75
CEIERPERLT	2.09
AGPRRPHRCS	3.86
MPIARPARQT	3.98
GLPGRPGRPG	2.45
EDFVRPLREG	3.72
LPPPRPQRPA	2.40
PLILRPQRGV	2.54
KDLSRPQKPQ	2.03
GGPQRPGRVV	3.63
GSPRRPRRQR	3.92
ALLYRPVRQY	2.58
QILSRPERLL	2.99
TVPTRPEKGY	2.75
DFLQRPGRQP	2.32
HPLIRPFKTL	1.94
QIPSRPVRIP	3.95
ALPARPHRGL	3.78
PDIERPYKAE	1.88
RPPSRPSRPP	4.08
YNFIRPVKGK	2.28
RTFLRPPKVK	1.89
EALSRPARTA	2.64
SDPPRPHRIL	2.23
RDITRPSRPA	2.28
SQIERPIRIV	2.73
LRIARPPKQG	2.22
MILRRPSLQR	2.37
PGPKRPGNTL	1.72
EPPRRPRARS	0.62

LSDFPKKPPQPELG	1.81
IRLSPNRPPLGGSC	2.27
PRRPPPRPPCRLSM	4.44
QPGSPRKPPADAKA	1.56
TSAPPSRPPPPRAT	3.74
RPPRPSRPPPPTPR	3.86
KPPKPPKPPRPPKT	3.55
LASRPTRPPEPRHI	3.04
PGDSPAKPPEGSAG	1.72
PAPPPVKPPPSPVN	1.64
GEVPPPRPPPPEEK	1.66
RKPKPERPPSSSSS	1.82
PAAPPKKPPRPGAP	3.39
RPAPPQRPPPPSGA	3.30
DKNIPMKPPTPLLN	1.77
VEGSPFRPPSHSFS	1.71
PGPPPGRPPGPPPG	1.52
QELLPGKPPKSRVL	1.70
QPLGPAKPPAQQTG	1.72
TKTPPPRPPLPTQQ	2.85
CAVGPNRPPTVSQP	1.71
PPPKPNRPPNVDLT	2.50
PSQPPPRPPPDYLR	1.84
SLPSPSKPPIQQPT	1.74
GQDKPVKPPTKKSE	1.68
RLRLPPKPPPPLQP	2.95
EPPPPQKPPPPNSK	1.65
PGPVPPRPPAAEPP	1.70
ALPSPLRPPAPPLP	2.48
HPGKPERPPPQGGN	1.76
CLGLPSRPPSQLSG	1.63
VPKPPPKPPSPPAP	1.86
VAPRPARPPGDLGP	2.18
PPAPPLRPPGPPTG	2.55
FDGRPTKPPPNSYS	1.82
APQPPGRPPTPGPP	2.49
RPTVPARPPATHPR	3.91
DVVPPIKPPKEIED	1.64
PTRPPSRPPSRPSR	4.37
PGVPPARPPPLTKV	2.91
SGAPPTKPPTTPVG	1.62
YGLSPLRPPSVLPP	1.60
PPQPPQRPPPPTGL	3.14
SVIPPDRPPGASAG	1.73
EQPPPGRPPLRLED	1.53
EASGPERPPALLQE	1.72
PSLRPLKPPPPPRQ	2.94
DMAIPSKPPAASPE	1.60
VLALPGKPPFLTQL	1.76
RSRVPVRPPQQYSD	2.12
GPILPKKPPVKPPT	1.65
NTAKPTRPPSESSS	1.69
RQIPPYRPPQQGPP	2.23
APFLPPKPPPPIIV	1.55
ISTPPSRPPQVKTA	2.34
RPAKPHKPPGSMGL	2.21
KPASPKKPPPGERR	1.64
RQGPPQKPPGPAGP	1.67
FGDLPRKPPLPSSA	1.68
SPPSPDRPPHSQTS	1.75
VSGPPRKPPGTVAG	1.79
PSAPPAKPPSTQRA	1.73
KEMVPAKPPKGLVG	1.75
PKKPPVKPPTEPIG	1.58
LTPVPSKPPVTPAT	1.74
DDEPPARPPPPPPA	1.58
KANSPEKPPEAGAA	1.61
PPGKPERPPPQGGN	1.77
RLTSPLRPPEGVRL	2.37
FTGPPQKPPRLGAQ	3.43
VTDLPKKPPPPEVT	1.72
LGRLPSRPPLPGSG	2.43
ALHVPAKPPRAAAA	3.61
YQGGPPRPPMGMRP	1.94
PPARPPRPPYPPRR	4.20
RPALPGRPPRWLNR	4.21
VPQKPERPPLPPKP	3.42
RGGPPCKPPAPEDE	1.59
IKPFPSRPPVLTQE	1.68
ASQSPKKPPAKDPL	1.76
GGASPLRPPGAGPQ	1.81
LLPGPGRPPPPPTK	2.01
VPLPPARPPTRDNP	3.24
GVSKPRKPPMVMTP	1.77
PGPFPPRPPGPLGP	1.83
GPSSPQKPPTPEET	1.64
ESGRPIKPPRKDLP	3.95
ILGSPARPPFSFQP	1.52
VKKIPKKPPAPDPF	1.75
RGFLPYRPPRPAFF	3.86
KIPKPPKPPKPPRP	3.12
CLSLPDKPPPQPQQ	1.76
KIIPPKKPPTKVVP	1.59
ITVSPNKPPAVSPG	1.62
PPALPPKPPKPMTS	1.95
PSPSPARPPGTCPP	1.49
QPQGPPRPPQGGRP	1.77
PPPLPNKPPPEDYY	1.63
STAGPLRPPPPGAG	1.79
SSGPPPKPPVSSGS	1.91
KAFPPPRPPPPNWE	2.16
QPGSPRKPPMDEKS	1.78
SPVLPARPPPPPLP	2.85
DQIPPNKPPWFDQP	1.64
PPPIPKKPPKGKFP	1.81
RPVLPQRPPEERPP	2.40
LGLPPGKPPSSSPL	1.37
APEPPPRPPPALSP	2.98
PQPPPKPPRPQPP	2.71
TAPLPIRPPSGTKK	2.67
QVDPPERPPPPPSD	1.64
EIPKPIKPPTYQVL	1.72
DKCPPPRPPTSPYP	1.92
TGGGPGRPPDWTPH	1.64
LGASPAKPPSPGAP	1.79
PAVPPARPPTAQEV	1.96
KYCGPYKPPGILKQ	1.60
QAQLPPKPPAWAWA	1.59
AVAPPARPPRPPYP	3.62
PPPPPTKPPETEAQ	1.78
QGAGPSRPPTPGRN	3.01
KACRPIKPPGPPIN	1.68
CWGGPWRPPPPGFW	1.64
PDHRPPRPPQPPPP	2.74
AERPPSKPPPVELR	1.85
GQQLPPRPPQSSSV	1.82
LQLGPSRPPTGEAL	1.58
ETSGPERPPEKPRK	1.59
SLVLPCRPPIGLPP	1.61
FGTLPPRPPGQDTS	1.72
EARKPAKPPVSPKP	1.73
PREGPGRPPLDGRD	1.67
LPEVPEKPPAGECV	1.60
SNPVPPRPPPPCLA	2.16
SDFSPSKPPSGPGK	1.72
FVLVPLRPPSPARS	3.21
LGHPPAKPPLPPGP	1.81
SPAPPHKPPNTREN	1.68
KKPVPARPPPPTNF	2.18
QVIGPHRPPELHDR	1.82
HDDPPQKPPRSITL	3.92
KPQPPARPPPPVLP	3.18
SGSRPRKPPPRKHT	3.57
LGPVPHRPPFEPGS	1.81
GGGSPEKPPSRRRP	2.91
MDFSPPRPPYPSYP	1.26
GHTLPPKPPSPRGT	2.76
GLGGPQKPPPHTEP	1.60
CQPGPAKPPAHRPC	1.60
DRPKPPRPPTGRPS	2.76
EGNRPKKPPPPSAP	1.73
TRSPPPRPPPPAIN	3.15
PPALPPKPPKAKPA	2.09
CTPRPARPPSPTEQ	2.51
FDGSPEKPPEDDSD	1.56
VNIVPKKPPLLDRP	1.54
EDVKPFKPPLSDAE	1.62
TPTVPLKPPPPLTL	1.59
PPGLPPRPPLSSSS	2.64
TNFPPPRPPPPSRP	3.21
RGGPPPRPPPRQSL	4.25
PSDLPKKPPKPEFG	1.21
ESSRPVKPPKKDVP	1.72
QQPGPAKPPTQQVG	1.80
SQPGPAKPPAQPSG	1.89
KSQGPAKPPPPRKP	3.12
TTRRPPRPPGSSRK	3.50
RRNVPMKPPDEAKV	3.36
VTIRPLRPPQRARL	4.29
KGSKPVRPPAPGHG	1.81
PPPPPERPPPIPPD	1.46
VVPVPERPPEPRAM	1.20
PREFPFKPPSIYMI	1.51
PPSRPSRPPSHPSA	4.17
TIPKPPKPPDKPLM	1.77
YTCKIFRPPRASHC	3.25
RSQTPERPAKKIRE	2.45
LQGIFTRPSKLTRQ	2.51
NTFHILRPTKAPGF	2.29
LDNALIRPGRFDMQ	2.38
EQGGLLRPERDHVE	1.90
ESRDITRPSRPASY	2.73
TENAPCRPLRRRRK	4.28
IRGLLARPAKLQVQ	1.99
EREFLERPSRDHLY	2.52
SGVLLVRPSRLSSS	3.90
RPHAPGRPARGKVQ	3.59
PLRPILRPRKYPNR	3.32
SASEFDRPLRPIVV	2.12
VASGPSRPGREEHI	3.40
EKGQPSRPTKSKDA	1.91
NMNRFDRPDRNVRQ	3.56
FLAKIARPKKRAET	2.23

LNATKISPSAPSKD	1.72
EHLQKERPKPPNKG	2.87
EFLIKEDPDLPPQG	1.89
NAPPRIFPKAPIYG	3.93
NRKQRSIPLSPIVP	3.33
QSEEKEKPKGPMAV	1.94
EKEAKSEPQQPEDN	1.35
LNLSKDAPVPPAPE	2.01
KLNKRVLPSKPKKS	2.49
SETARKVPPIPTQI	1.86
KDFSKNAPYNPSEV	1.88
WFILKLFPHEPIEG	1.11
RAGNREPPSTPSTL	2.85
NNLKKRPPTAPQRK	3.36
LKDPKSFPPPPLKP	1.74
HEVPKRSPEKPSNP	1.96
LDKGKHVPSKPVSN	2.56
TMKNKPKPTPPSPP	2.46
YFVKKIRPGNPLTK	2.61
SYMDKIYPDLPNDK	2.34
LPEPRFYPLSPKNN	1.98
KKIIKDVPGTPSDK	3.58
LPEKKNSPTTPSVF	1.95
VEMKRKKPVRPVDI	3.21
EIPSKDHPYDPEKD	1.71
NPIPKSVPLHPKSG	2.35
PTVQKFSPHPPTTL	1.76
PPPPRRGPAPPPPP	3.64
SQKKKALPKQPKAS	3.03
KLVLRNVPILPILN	1.62
TYIWRYLPAPPVCI	1.81
TQMKRLKPVSPGLR	3.46
LAPKREAPKPPANT	2.25
KTSTKAQPAEPQAE	1.93
KVSSKYDPDNPNKD	2.10
PTANKYAPVSPTVQ	2.23
PIGSRYNPNLPVTE	2.12
VCHSKYIPLKPIQD	2.14
EKVFRGDPEHPAIS	4.24
LAAHKTKPSIPPKK	2.68
EKESRHYPIKPPSP	1.81
DLSLRYAPNLPRVI	2.55
LTSFKGRPKPPKTL	2.85
PQQNRPLPQLPNRN	3.57
HICDKMNPASPQYF	2.05
KEFFRLTPNQPVGL	2.13
HLLGKEGPSAPNPS	1.95
KDKARGNPEKPPYW	2.39
KVIRKGSPKSPAMV	2.66
YGDNKKNPESPESV	1.90
PCKKKSKPKVPAKN	2.58
CRHLKLVPSTPMDI	2.64
KQLMKNLPKIPLLN	2.77
AGQERFAPLAPMYY	1.53
FYKEKAKPLEPILD	1.91
RYCFKLLPPQPGSK	2.90
SKDKKTKPTPPPKP	2.55
REGPKSCPRGPLCP	3.59
YDLTRFLPNHPGGQ	1.77
AMESRVAPEIPGLI	1.92
TSWARDNPSKPDIA	1.92
KFDAKGRPIPPHPD	2.66
QLQEKLSPDLPDEE	1.69
NTIIKLFPYIPIPV	1.95
FYDFRSLPKVPTTQ	3.64
DSVLRQKPDKPRPI	3.09
VWGPKPVPFQPMDV	2.68
EYFPREYPKDPVSE	2.66
YVKERRPPPPPPLL	1.24
NEFAKFAPDLPVLK	1.39
KLPKKFPPPTPLLE	2.91
PIRLRKRPPPPPPV	3.81
GTGRRRSPSTPIMP	2.80
KQKKKPTPTRPYNY	1.53
LEFPKDYPLSPPKL	2.04
TLNDRQIPLSPDQM	1.78
AVVYKLKPYIPSLS	2.00
LNKTKLLPQSPILN	1.97
KDEVKLHPPEPKDL	2.01
EFTTREGPHQPIGN	2.43
KVNFRRIPTGPDSP	3.05
NSFSKNDPNFPILK	2.31
INIAKAHPNFPIAL	1.73
TSSLRKPPNGPSSV	1.47
FVSPRRAPKPPSYP	3.70
VVPERVKPAPPVSR	3.43
VNTPRSVPNSPNDG	1.46
LKKQKAIPSSPCGM	2.85
TNGPRYSPQNPRPF	1.48
GKDHRLAPNGPGMH	3.33
MGLNKDVPHCPESL	2.16
AFYYKYYPVEPNCN	2.62
SERRKALPKEPKKL	3.07
LPLPKLRPPRPTFA	3.59
RRKLRTVPGVPLIH	1.95
FQPQRTAPKPPISA	3.68
PPLPRAPPVPPATF	2.18
LSPAKARPSFPHND	2.91
FQDYRCSPWCPSRS	4.53
SILNRCVPKRPTID	2.98
SMDSKRKPSPPSQR	3.05
LQNAKNDPLPPVIE	2.08
STNAKKDPFPPPKD	1.52
KRKAKAPPPPPPPP	3.04
GDEERFLPNRPHYE	1.73
SFVTKYLPAAPVGS	1.43
RSRSKKDPGIPSNF	2.77
VLFMKGTPEFPKCG	2.18
VHFYKSLPQGPAPA	1.70
SYISRKYPRAPAFI	3.49
PQTMRQAPKRPDAD	2.54
DENSKWQPSDPFTM	1.96
KMSAKNTPKHPVDI	3.09
MAFIKILPNNPWPF	1.64
RHSKKPAPPPPGMQ	2.53
FEYSKYRPLTPSSE	1.87
GDHPKGPPPPPPPP	1.85
ALLRRTIPKRPFYH	3.68
DVNHRYPPMAPTTV	1.76
NIPTKSPPKSPKHK	2.87
EFDLKNIPMMPAGE	1.27
HEREKALPPIPFTT	1.74
PPSIRKQPIAPDVL	2.34
LASDKGLPMIPAFV	1.97
IAGQRTFPEDPNYP	1.65
KNLLREIPQVPAFQ	2.29
WLDEKRIPSTPIDY	1.06
VKKEKKPPKIPKKV	3.11
ILPPRIVPAIPKKA	2.36
QNGLRFNPDVPKLW	1.81
IKMSRSPPRPPSLK	3.12
MDKFRIPPKSPNIE	3.50
YVNAKLDPSLPSQY	1.85
FKVYRWNPDEPSAK	2.95
VEVDKKVPVLPMVA	2.03
NYHHRETPPPPPSN	1.77
SSKIRPTPRKPSRM	2.99
NKPKRKRPAPPKKK	2.80
EFLRKHSPKAPATG	2.79
YVYGRQDPKKPFFT	3.12
INTFKTLPTKPEMQ	1.57
KALPKRRPSPPLQS	3.25
AISKKLGPDTPIKV	2.67
LNPNRRAPRRPLST	3.69
ETTTRETPLPPIPA	1.93
ISADKGFPLIPSGY	1.48
TSISRISPQDPEAK	1.98
GHKKKNLPKFPKNK	2.71
NELIKQGPKRPTSA	2.66
EEKVKRTPPLPPVP	2.03
LNFPKNIPTIPFYV	1.97
CNYSKIFPGIPISL	1.61
TTNSRLCPSSPSSS	1.80
RFWTRARPIDPNAY	2.69
SLTLKTLPRVPTDS	1.88
GQIDRDKPSEPERT	1.18
LFDEKVKPQIPQLR	1.77
DEFNRNTPPQPSLL	1.87
INGEKACPFLPSSL	2.00
TTTIKNRPVPPPLP	1.94
NDNEKKKPSSPTAA	2.33
SIIPRIFPSTPGTI	2.25
KKVIRNYPGHPEGT	2.76
KQSKRARPSSPYTV	2.46
RPVYRLKPNAPFQY	1.86
GRHLKNIPRIPIFC	2.85
PSSKKNKPNPPPGS	2.76
TDTTKVTPIPVPVI	1.43
ENENKKLPPAPTVF	1.71
WLGNKYDPENPFND	1.82
ETGVRYGPEFPLCT	1.97
GEMVKLTPKNPFYK	2.18
VCTIRSTPSQPIHC	1.97
FHPEKLTPYTPMTG	1.98
NPMQKPVPFVPVLD	1.82
TLKKKKTPKKPIAA	3.18
TKTDKSYPMCPVDA	1.72
SLGERFMPMAPEVY	1.70
ITSIRGSPSLPKNK	2.62
HFPSRSQPSDPKLQ	1.96
DIGSKKLPTGPSKK	3.59
TTRKKKDPNAPKRA	2.62
PSAMKLVPSIPPAT	1.95
LLEERNNPDIPVDK	1.93
QTVIKTHPRIPKSE	2.33
LITDKEYPVRPAYT	2.14
DILKKESPDVPLFD	2.23
NLKTKSHPEVPRLD	2.63
IGSPRHAPSRPDSI	2.26
AVQLKRQPAQPRES	3.17
CWHPKTIPKQPMEI	1.42
QWSKRIMPYIPGPV	2.09
FVFFKSLPDLPKPQ	2.11

KLNKRVLPSKPKKS	0.18
SETARKVPPIPTQI	1.54
KDFSKNAPYNPSEV	1.73
WFILKLFPHEPIEG	1.49
RAGNREPPSTPSTL	1.27
NNLKKRPPTAPQRK	2.18
LKDPKSFPPPPLKP	1.66
HEVPKRSPEKPSNP	0.38
LDKGKHVPSKPVSN	1.10
TMKNKPKPTPPSPP	1.72
YFVKKIRPGNPLTK	1.77
SYMDKIYPDLPNDK	1.36
LPEPRFYPLSPKNN	1.67
KKIIKDVPGTPSDK	0.75
VEMKRKKPVRPVDI	1.68
EIPSKDHPYDPEKD	0.86
NPIPKSVPLHPKSG	0.90
PTVQKFSPHPPTTL	1.16
PPPPRRGPAPPPPP	1.30
SQKKKALPKQPKAS	1.47
KLVLRNVPILPILN	1.24
TYIWRYLPAPPVCI	1.37
TQMKRLKPVSPGLR	3.06
LAPKREAPKPPANT	1.45
KTSTKAQPAEPQAE	0.57
KVSSKYDPDNPNKD	1.38
PTANKYAPVSPTVQ	1.44
PIGSRYNPNLPVTE	2.17
VCHSKYIPLKPIQD	1.48
EKVFRGDPEHPAIS	1.18
LAAHKTKPSIPPKK	1.27
EKESRHYPIKPPSP	1.31
DLSLRYAPNLPRVI	1.27
LTSFKGRPKPPKTL	1.11
PQQNRPLPQLPNRN	0.61
HICDKMNPASPQYF	1.26
HLLGKEGPSAPNPS	1.34
KDKARGNPEKPPYW	1.18
YGDNKKNPESPESV	0.92
PCKKKSKPKVPAKN	1.52
CRHLKLVPSTPMDI	2.01
KQLMKNLPKIPLLN	0.99
AGQERFAPLAPMYY	1.48
FYKEKAKPLEPILD	1.44
RYCFKLLPPQPGSK	1.68
SKDKKTKPTPPPKP	1.61
REGPKSCPRGPLCP	1.08
YDLTRFLPNHPGGQ	1.66
TSWARDNPSKPDIA	1.31
KFDAKGRPIPPHPD	0.54
QLQEKLSPDLPDEE	1.45
NTIIKLFPYIPIPV	1.47
FYDFRSLPKVPTTQ	1.87
DSVLRQKPDKPRPI	1.01
VWGPKPVPFQPMDV	2.39
EYFPREYPKDPVSE	0.75
YVKERRPPPPPPLL	1.25
NEFAKFAPDLPVLK	1.09
PIRLRKRPPPPPPV	1.59
GTGRRRSPSTPIMP	1.72
KQKKKPTPTRPYNY	1.42
TLNDRQIPLSPDQM	1.44
AVVYKLKPYIPSLS	1.40
LNKTKLLPQSPILN	0.94
KDEVKLHPPEPKDL	0.38
YPNGRTLPPVPTQV	1.26
KVNFRRIPTGPDSP	1.39
NSFSKNDPNFPILK	1.53
AGPRKYIPRAPAQG	1.59
INIAKAHPNFPIAL	1.31
TSSLRKPPNGPSSV	1.24
FVSPRRAPKPPSYP	1.12
VVPERVKPAPPVSR	1.71
VNTPRSVPNSPNDG	1.45
LKKQKAIPSSPCGM	1.21
TNGPRYSPQNPRPF	1.19
VFVEKSKPTYPGTE	1.28
GKDHRLAPNGPGMH	0.67
MGLNKDVPHCPESL	1.70
AFYYKYYPVEPNCN	1.72
SERRKALPKEPKKL	1.64
LPLPKLRPPRPTFA	0.57
RRKLRTVPGVPLIH	1.30
FQPQRTAPKPPISA	1.29
PPLPRAPPVPPATF	1.12
LSPAKARPSFPHND	1.50
FQDYRCSPWCPSRS	2.50
SILNRCVPKRPTID	0.91
SMDSKRKPSPPSQR	2.04
LQNAKNDPLPPVIE	1.34
STNAKKDPFPPPKD	-0.05
KRKAKAPPPPPPPP	1.35
GDEERFLPNRPHYE	1.54
SFVTKYLPAAPVGS	1.28
RSRSKKDPGIPSNF	0.84
VLFMKGTPEFPKCG	2.03
VHFYKSLPQGPAPA	1.65
SYISRKYPRAPAFI	1.52
GLMSKLGPNLPASI	1.60
PQTMRQAPKRPDAD	2.87
DENSKWQPSDPFTM	1.43
KMSAKNTPKHPVDI	1.63
RHSKKPAPPPPGMQ	1.35
FEYSKYRPLTPSSE	2.04
GDHPKGPPPPPPPP	-0.52
ALLRRTIPKRPFYH	2.40
DVNHRYPPMAPTTV	1.53
NIPTKSPPKSPKHK	0.08
EFDLKNIPMMPAGE	0.96
HEREKALPPIPFTT	0.23
EKLIKITPDTPFGF	1.28
PPSIRKQPIAPDVL	1.13
LASDKGLPMIPAFV	2.13
KNLLREIPQVPAFQ	0.81
WLDEKRIPSTPIDY	2.98
AKTNKNVPVLPTPG	1.85
VKKEKKPPKIPKKV	0.95
ILPPRIVPAIPKKA	1.46
IKMSRSPPRPPSLK	2.62
MDKFRIPPKSPNIE	1.87
YVNAKLDPSLPSQY	1.03
FKVYRWNPDEPSAK	1.46
VEVDKKVPVLPMVA	1.50
NYHHRETPPPPPSN	1.77
SSKIRPTPRKPSRM	1.77
NKPKRKRPAPPKKK	1.36
EFLRKHSPKAPATG	1.57
YVYGRQDPKKPFFT	1.37
INTFKTLPTKPEMQ	1.06
KALPKRRPSPPLQS	1.48
AISKKLGPDTPIKV	0.52
PGGPKFEPLYPREE	1.22
LNPNRRAPRRPLST	2.70
ETTTRETPLPPIPA	1.64
ISADKGFPLIPSGY	1.23
GHKKKNLPKFPKNK	1.36
NELIKQGPKRPTSA	1.34
KALWKIQPYLPEFS	1.68
DRKNRAPPQRPRFN	1.38
EEKVKRTPPLPPVP	1.25
CNYSKIFPGIPISL	1.41
TTNSRLCPSSPSSS	1.44
RFWTRARPIDPNAY	3.09
SLTLKTLPRVPTDS	1.45
LNFKRLLPRDPSEK	1.76
GQIDRDKPSEPERT	1.32
LFDEKVKPQIPQLR	0.69
DEFNRNTPPQPSLL	1.53
INGEKACPFLPSSL	1.37
NDNEKKKPSSPTAA	1.79
SIIPRIFPSTPGTI	1.35
KKVIRNYPGHPEGT	1.39
KQSKRARPSSPYTV	1.08
RPVYRLKPNAPFQY	1.89
GRHLKNIPRIPIFC	0.79
PSSKKNKPNPPPGS	-0.10
ENENKKLPPAPTVF	1.11
WLGNKYDPENPFND	1.85
ETGVRYGPEFPLCT	1.37
GEMVKLTPKNPFYK	1.37
VCTIRSTPSQPIHC	1.59
FHPEKLTPYTPMTG	1.29
NPMQKPVPFVPVLD	1.70
TLKKKKTPKKPIAA	1.11
TKTDKSYPMCPVDA	1.29
SLGERFMPMAPEVY	1.71
ITSIRGSPSLPKNK	1.50
HFPSRSQPSDPKLQ	1.25
DIGSKKLPTGPSKK	1.54
TTRKKKDPNAPKRA	1.61
PSAMKLVPSIPPAT	1.33
LLEERNNPDIPVDK	1.29
QTVIKTHPRIPKSE	1.23
LITDKEYPVRPAYT	1.34
DILKKESPDVPLFD	1.24
NLKTKSHPEVPRLD	2.15
IGSPRHAPSRPDSI	1.45
AVQLKRQPAQPRES	1.15
CWHPKTIPKQPMEI	1.61
QWSKRIMPYIPGPV	2.16
FVFFKSLPDLPKPQ	0.93
RGWQRFLPTGPIAH	2.08
KVLIKTLPVLPEAR	0.73
QTGSKTVPQAPQAP	1.25
VHVLKYAPDEPNSL	1.47
SDNSKSEPLPPVIT	0.15
LDPGKNTPGLPEET	2.10
LPLSRVQPVQPSQT	1.43
ASFRKTKPPPPLDF	1.33
PKTGKPLPTQPSSS	0.99
QATGRRGPAPPPPP	1.05
LRISKPDPECPSKL	-0.22
EIKDRDQPAVPPSP	1.31

LSDFPKKPPQPELG	1.98
IRLSPNRPPLGGSC	3.04
PRRPPPRPPCRLSM	4.51
QPGSPRKPPADAKA	1.66
TSAPPSRPPPPRAT	4.15
RPPRPSRPPPPTPR	4.14
KPPKPPKPPRPPKT	4.23
LASRPTRPPEPRHI	4.36
PGDSPAKPPEGSAG	1.86
PAPPPVKPPPSPVN	2.26
GEVPPPRPPPPEEK	3.06
RKPKPERPPSSSSS	3.88
PAAPPKKPPRPGAP	2.90
RPAPPQRPPPPSGA	4.09
DKNIPMKPPTPLLN	2.38
VEGSPFRPPSHSFS	1.90
PGPPPGRPPGPPPG	2.97
QELLPGKPPKSRVL	2.19
QPLGPAKPPAQQTG	1.80
TKTPPPRPPLPTQQ	4.13
CAVGPNRPPTVSQP	2.99
PPPKPNRPPNVDLT	3.46
PSQPPPRPPPDYLR	3.25
SLPSPSKPPIQQPT	1.98
GQDKPVKPPTKKSE	2.37
RLRLPPKPPPPLQP	3.78
EPPPPQKPPPPNSK	3.35
PGPVPPRPPAAEPP	2.52
ALPSPLRPPAPPLP	3.29
HPGKPERPPPQGGN	2.90
CLGLPSRPPSQLSG	2.01
VPKPPPKPPSPPAP	2.55
VAPRPARPPGDLGP	2.80
PPAPPLRPPGPPTG	3.57
FDGRPTKPPPNSYS	2.55
APQPPGRPPTPGPP	2.91
RPTVPARPPATHPR	4.17
DVVPPIKPPKEIED	1.74
PTRPPSRPPSRPSR	4.30
PGVPPARPPPLTKV	4.00
SGAPPTKPPTTPVG	1.82
YGLSPLRPPSVLPP	2.07
PPQPPQRPPPPTGL	3.99
SVIPPDRPPGASAG	1.86
EQPPPGRPPLRLED	1.97
EASGPERPPALLQE	1.80
PSLRPLKPPPPPRQ	3.66
DMAIPSKPPAASPE	1.90
VLALPGKPPFLTQL	2.68
RSRVPVRPPQQYSD	2.92
GPILPKKPPVKPPT	3.26
NTAKPTRPPSESSS	2.15
RQIPPYRPPQQGPP	1.83
APFLPPKPPPPIIV	3.12
ISTPPSRPPQVKTA	3.29
RPAKPHKPPGSMGL	3.72
KPASPKKPPPGERR	2.89
RQGPPQKPPGPAGP	2.51
FGDLPRKPPLPSSA	3.00
SPPSPDRPPHSQTS	1.84
VSGPPRKPPGTVAG	1.80
PSAPPAKPPSTQRA	2.41
KEMVPAKPPKGLVG	2.44
PKKPPVKPPTEPIG	1.81
LTPVPSKPPVTPAT	1.57
DDEPPARPPPPPPA	1.73
KANSPEKPPEAGAA	1.68
PPGKPERPPPQGGN	2.88
RLTSPLRPPEGVRL	3.02
FTGPPQKPPRLGAQ	1.88
VTDLPKKPPPPEVT	1.77
LGRLPSRPPLPGSG	4.17
ALHVPAKPPRAAAA	2.72
YQGGPPRPPMGMRP	2.79
PPARPPRPPYPPRR	4.30
RPALPGRPPRWLNR	4.13
VPQKPERPPLPPKP	4.14
RGGPPCKPPAPEDE	1.53
IKPFPSRPPVLTQE	2.64
ASQSPKKPPAKDPL	1.78
GGASPLRPPGAGPQ	1.91
LLPGPGRPPPPPTK	3.51
VPLPPARPPTRDNP	2.77
GVSKPRKPPMVMTP	2.52
PGPFPPRPPGPLGP	3.28
GPSSPQKPPTPEET	1.76
ESGRPIKPPRKDLP	3.29
ILGSPARPPFSFQP	3.19
VKKIPKKPPAPDPF	3.44
RGFLPYRPPRPAFF	4.14
KIPKPPKPPKPPRP	4.00
CLSLPDKPPPQPQQ	1.78
KIIPPKKPPTKVVP	2.12
ITVSPNKPPAVSPG	1.77
PPALPPKPPKPMTS	3.40
PSPSPARPPGTCPP	1.83
QPQGPPRPPQGGRP	2.32
PPPLPNKPPPEDYY	1.97
STAGPLRPPPPGAG	3.27
SSGPPPKPPVSSGS	2.09
KAFPPPRPPPPNWE	4.13
QPGSPRKPPMDEKS	1.83
SPVLPARPPPPPLP	3.18
DQIPPNKPPWFDQP	1.67
PPPIPKKPPKGKFP	3.59
RPVLPQRPPEERPP	2.62
LGLPPGKPPSSSPL	1.66
APEPPPRPPPALSP	3.37
PQPPPKPPRPQPP	3.53
TAPLPIRPPSGTKK	3.04
QVDPPERPPPPPSD	2.44
EIPKPIKPPTYQVL	1.80
DKCPPPRPPTSPYP	3.66
TGGGPGRPPDWTPH	2.82
LGASPAKPPSPGAP	2.01
PAVPPARPPTAQEV	2.71
KYCGPYKPPGILKQ	1.68
QAQLPPKPPAWAWA	2.07
AVAPPARPPRPPYP	3.98
PPPPPTKPPETEAQ	1.80
QGAGPSRPPTPGRN	3.35
KACRPIKPPGPPIN	3.33
CWGGPWRPPPPGFW	3.11
PDHRPPRPPQPPPP	3.14
AERPPSKPPPVELR	1.83
GQQLPPRPPQSSSV	2.69
LQLGPSRPPTGEAL	1.80
ETSGPERPPEKPRK	2.97
SLVLPCRPPIGLPP	1.86
FGTLPPRPPGQDTS	1.89
EARKPAKPPVSPKP	3.22
PREGPGRPPLDGRD	1.77
LPEVPEKPPAGECV	1.76
SNPVPPRPPPPCLA	4.06
SDFSPSKPPSGPGK	1.80
FVLVPLRPPSPARS	4.24
LGHPPAKPPLPPGP	2.42
SPAPPHKPPNTREN	1.89
KKPVPARPPPPTNF	4.08
QVIGPHRPPELHDR	1.92
HDDPPQKPPRSITL	2.12
KPQPPARPPPPVLP	4.04
SGSRPRKPPPRKHT	3.57
LGPVPHRPPFEPGS	1.62
GGGSPEKPPSRRRP	2.70
MDFSPPRPPYPSYP	2.99
GHTLPPKPPSPRGT	3.67
GLGGPQKPPPHTEP	1.90
CQPGPAKPPAHRPC	2.25
DRPKPPRPPTGRPS	3.88
EGNRPKKPPPPSAP	3.33
TRSPPPRPPPPAIN	4.12
PPALPPKPPKAKPA	3.64
CTPRPARPPSPTEQ	3.98
FDGSPEKPPEDDSD	1.56
VNIVPKKPPLLDRP	2.06
EDVKPFKPPLSDAE	1.76
TPTVPLKPPPPLTL	2.96
PPGLPPRPPLSSSS	3.74
TNFPPPRPPPPSRP	4.05
RGGPPPRPPPRQSL	4.23
PSDLPKKPPKPEFG	2.41
ESSRPVKPPKKDVP	1.99
QQPGPAKPPTQQVG	1.48
SQPGPAKPPAQPSG	2.11
KSQGPAKPPPPRKP	3.89
TTRRPPRPPGSSRK	4.05
RRNVPMKPPDEAKV	2.17
VTIRPLRPPQRARL	4.06
KGSKPVRPPAPGHG	4.33
PPPPPERPPPIPPD	1.87
VVPVPERPPEPRAM	2.93
PREFPFKPPSIYMI	2.72
PPSRPSRPPSHPSA	4.31
TIPKPPKPPDKPLM	3.24
YTCKIFRPPRASHC	2.51
RSQTPERPAKKIRE	2.77
LQGIFTRPSKLTRQ	2.32
NTFHILRPTKAPGF	2.41
LDNALIRPGRFDMQ	1.91
EQGGLLRPERDHVE	1.83
ESRDITRPSRPASY	2.28
TENAPCRPLRRRRK	3.22
IRGLLARPAKLQVQ	1.73
EREFLERPSRDHLY	2.03
SGVLLVRPSRLSSS	2.32
RPHAPGRPARGKVQ	3.10
PLRPILRPRKYPNR	3.69
SASEFDRPLRPIVV	1.73
VASGPSRPGREEHI	2.63
EKGQPSRPTKSKDA	2.16
NMNRFDRPDRNVRQ	2.65
FLAKIARPKKRAET	2.28

ADDLKYIPPIPQDF	1.94
RLLFRSLPPHPACL	2.87
ISHNKLSPSEPILC	2.31
LIWHKSKPVKPTNK	2.55
LVGMRSFPLYPSGL	2.59
DDNVRPSPISPSIN	2.19
RRIRKEHPGEPNVI	2.49
PEWRRPHPNIPDAK	2.26
VDSFKYEPLAPGND	2.13
DKKKKKYPRKPKKN	2.73
GFQDRSLPHFPKNS	2.25
LNATKISPSAPSKD	2.06
EHLQKERPKPPNKG	2.56
EFLIKEDPDLPPQG	2.24
NAPPRIFPKAPIYG	2.23
NRKQRSIPLSPIVP	2.69
QSEEKEKPKGPMAV	2.30
EKEAKSEPQQPEDN	2.14
LNLSKDAPVPPAPE	2.21
KLNKRVLPSKPKKS	2.96
SETARKVPPIPTQI	2.62
KDFSKNAPYNPSEV	2.15
WFILKLFPHEPIEG	3.10
RAGNREPPSTPSTL	2.44
NNLKKRPPTAPQRK	2.62
LKDPKSFPPPPLKP	2.40
HEVPKRSPEKPSNP	2.23
LDKGKHVPSKPVSN	2.30
TMKNKPKPTPPSPP	2.96
YFVKKIRPGNPLTK	2.71
SYMDKIYPDLPNDK	1.92
LPEPRFYPLSPKNN	2.46
KKIIKDVPGTPSDK	2.21
LPEKKNSPTTPSVF	2.01
VEMKRKKPVRPVDI	2.67
EIPSKDHPYDPEKD	1.71
NPIPKSVPLHPKSG	2.56
PTVQKFSPHPPTTL	2.33
PPPPRRGPAPPPPP	4.18
SQKKKALPKQPKAS	2.69
KLVLRNVPILPILN	2.48
TYIWRYLPAPPVCI	2.65
TQMKRLKPVSPGLR	3.16
LAPKREAPKPPANT	5.06
KTSTKAQPAEPQAE	2.22
KVSSKYDPDNPNKD	2.64
PTANKYAPVSPTVQ	2.28
PIGSRYNPNLPVTE	2.27
VCHSKYIPLKPIQD	2.47
EKVFRGDPEHPAIS	1.95
LAAHKTKPSIPPKK	2.40
EKESRHYPIKPPSP	2.26
DLSLRYAPNLPRVI	2.40
LTSFKGRPKPPKTL	2.52
PQQNRPLPQLPNRN	2.27
HICDKMNPASPQYF	2.20
KEFFRLTPNQPVGL	1.64
HLLGKEGPSAPNPS	2.10
KDKARGNPEKPPYW	1.78
KVIRKGSPKSPAMV	2.70
YGDNKKNPESPESV	2.17
PCKKKSKPKVPAKN	2.76
CRHLKLVPSTPMDI	2.56
KQLMKNLPKIPLLN	2.76
AGQERFAPLAPMYY	2.00
FYKEKAKPLEPILD	1.94
RYCFKLLPPQPGSK	2.28
SKDKKTKPTPPPKP	2.25
REGPKSCPRGPLCP	2.28
YDLTRFLPNHPGGQ	1.91
AMESRVAPEIPGLI	2.63
TSWARDNPSKPDIA	1.91
KFDAKGRPIPPHPD	1.73
QLQEKLSPDLPDEE	2.33
NTIIKLFPYIPIPV	2.31
FYDFRSLPKVPTTQ	2.53
DSVLRQKPDKPRPI	2.13
VWGPKPVPFQPMDV	1.99
EYFPREYPKDPVSE	2.43
YVKERRPPPPPPLL	4.20
NEFAKFAPDLPVLK	2.13
KLPKKFPPPTPLLE	4.29
PIRLRKRPPPPPPV	4.78
GTGRRRSPSTPIMP	2.96
KQKKKPTPTRPYNY	2.71
LEFPKDYPLSPPKL	2.13
TLNDRQIPLSPDQM	1.87
AVVYKLKPYIPSLS	2.05
LNKTKLLPQSPILN	2.07
KDEVKLHPPEPKDL	2.21
EFTTREGPHQPIGN	1.90
YPNGRTLPPVPTQV	2.24
KVNFRRIPTGPDSP	2.70
NSFSKNDPNFPILK	2.21
AGPRKYIPRAPAQG	2.29
INIAKAHPNFPIAL	2.29
TSSLRKPPNGPSSV	2.16
FVSPRRAPKPPSYP	3.20
VVPERVKPAPPVSR	2.18
RDDVKRTPFIPESV	2.23
VNTPRSVPNSPNDG	1.81
LKKQKAIPSSPCGM	2.49
TNGPRYSPQNPRPF	2.20
VFVEKSKPTYPGTE	1.98
GKDHRLAPNGPGMH	2.34
MGLNKDVPHCPESL	2.00
AFYYKYYPVEPNCN	2.27
SERRKALPKEPKKL	2.30
LPLPKLRPPRPTFA	4.89
RRKLRTVPGVPLIH	3.30
FQPQRTAPKPPISA	4.95
PPLPRAPPVPPATF	4.56
LSPAKARPSFPHND	2.33
FQDYRCSPWCPSRS	2.42
SILNRCVPKRPTID	2.12
SMDSKRKPSPPSQR	2.71
LQNAKNDPLPPVIE	1.77
STNAKKDPFPPPKD	2.30
KRKAKAPPPPPPPP	3.60
GDEERFLPNRPHYE	2.10
SFVTKYLPAAPVGS	2.33
YTYSRNEPVIPTKP	2.32
RSRSKKDPGIPSNF	2.90
VLFMKGTPEFPKCG	2.33
VHFYKSLPQGPAPA	2.71
SYISRKYPRAPAFI	2.87
GLMSKLGPNLPASI	2.46
PQTMRQAPKRPDAD	3.51
DENSKWQPSDPFTM	2.46
KMSAKNTPKHPVDI	2.49
MAFIKILPNNPWPF	1.78
RHSKKPAPPPPGMQ	3.29
FEYSKYRPLTPSSE	2.16
GDHPKGPPPPPPPP	4.31
ALLRRTIPKRPFYH	2.96
DVNHRYPPMAPTTV	2.24
NIPTKSPPKSPKHK	4.10
EFDLKNIPMMPAGE	2.03
HEREKALPPIPFTT	2.33
EKLIKITPDTPFGF	2.31
PPSIRKQPIAPDVL	2.18
LASDKGLPMIPAFV	1.90
IAGQRTFPEDPNYP	2.23
KNLLREIPQVPAFQ	2.02
WLDEKRIPSTPIDY	2.21
AKTNKNVPVLPTPG	2.11
VKKEKKPPKIPKKV	2.73
ILPPRIVPAIPKKA	3.56
QNGLRFNPDVPKLW	2.42
IKMSRSPPRPPSLK	4.25
MDKFRIPPKSPNIE	2.35
YVNAKLDPSLPSQY	1.99
FKVYRWNPDEPSAK	2.00
VEVDKKVPVLPMVA	2.11
NYHHRETPPPPPSN	2.50
SSKIRPTPRKPSRM	2.83
NKPKRKRPAPPKKK	2.94
EFLRKHSPKAPATG	2.62
YVYGRQDPKKPFFT	2.54
INTFKTLPTKPEMQ	2.22
KALPKRRPSPPLQS	3.06
AISKKLGPDTPIKV	2.25
PGGPKFEPLYPREE	1.95
LNPNRRAPRRPLST	3.90
ETTTRETPLPPIPA	2.25
ISADKGFPLIPSGY	2.05
TSISRISPQDPEAK	2.29
GHKKKNLPKFPKNK	2.90
NELIKQGPKRPTSA	2.24
KALWKIQPYLPEFS	2.16
DRKNRAPPQRPRFN	3.39
EEKVKRTPPLPPVP	2.77
LNFPKNIPTIPFYV	2.41
CNYSKIFPGIPISL	1.69
TTNSRLCPSSPSSS	2.21
RFWTRARPIDPNAY	2.45
SLTLKTLPRVPTDS	2.33
LNFKRLLPRDPSEK	2.74
GQIDRDKPSEPERT	2.00
LFDEKVKPQIPQLR	1.95
DEFNRNTPPQPSLL	2.07
INGEKACPFLPSSL	2.26
TTTIKNRPVPPPLP	3.27
NDNEKKKPSSPTAA	1.99
SIIPRIFPSTPGTI	2.57
KKVIRNYPGHPEGT	2.62
KQSKRARPSSPYTV	2.43
RPVYRLKPNAPFQY	2.32
GRHLKNIPRIPIFC	3.02
PSSKKNKPNPPPGS	2.03
TDTTKVTPIPVPVI	1.68
ENENKKLPPAPTVF	1.83
WLGNKYDPENPFND	1.95

ADDLKYIPPIPQDF	1.84
RLLFRSLPPHPACL	2.77
ISHNKLSPSEPILC	1.68
LIWHKSKPVKPTNK	1.86
LVGMRSFPLYPSGL	2.59
DDNVRPSPISPSIN	1.24
RRIRKEHPGEPNVI	2.03
PEWRRPHPNIPDAK	1.97
VDSFKYEPLAPGND	1.55
DKKKKKYPRKPKKN	1.82
GFQDRSLPHFPKNS	2.38
LNATKISPSAPSKD	1.47
EHLQKERPKPPNKG	2.05
EFLIKEDPDLPPQG	2.13
NAPPRIFPKAPIYG	2.19
NRKQRSIPLSPIVP	2.49
QSEEKEKPKGPMAV	1.31
EKEAKSEPQQPEDN	1.29
LNLSKDAPVPPAPE	2.11
KLNKRVLPSKPKKS	2.91
SETARKVPPIPTQI	4.33
KDFSKNAPYNPSEV	1.80
WFILKLFPHEPIEG	1.64
RAGNREPPSTPSTL	1.89
NNLKKRPPTAPQRK	3.78
LKDPKSFPPPPLKP	1.89
HEVPKRSPEKPSNP	1.67
LDKGKHVPSKPVSN	1.78
TMKNKPKPTPPSPP	1.68
YFVKKIRPGNPLTK	2.34
SYMDKIYPDLPNDK	1.88
LPEPRFYPLSPKNN	1.69
KKIIKDVPGTPSDK	1.81
LPEKKNSPTTPSVF	1.83
VEMKRKKPVRPVDI	1.81
NPIPKSVPLHPKSG	0.86
PTVQKFSPHPPTTL	1.32
PPPPRRGPAPPPPP	1.76
SQKKKALPKQPKAS	2.27
KLVLRNVPILPILN	2.58
TYIWRYLPAPPVCI	2.55
TQMKRLKPVSPGLR	3.29
LAPKREAPKPPANT	1.84
KTSTKAQPAEPQAE	1.86
KVSSKYDPDNPNKD	1.98
PTANKYAPVSPTVQ	1.97
PIGSRYNPNLPVTE	1.98
VCHSKYIPLKPIQD	0.80
EKVFRGDPEHPAIS	1.82
LAAHKTKPSIPPKK	1.22
EKESRHYPIKPPSP	1.86
DLSLRYAPNLPRVI	1.62
LTSFKGRPKPPKTL	1.70
PQQNRPLPQLPNRN	4.73
HICDKMNPASPQYF	1.48
KEFFRLTPNQPVGL	2.24
HLLGKEGPSAPNPS	1.69
KDKARGNPEKPPYW	1.83
KVIRKGSPKSPAMV	2.30
YGDNKKNPESPESV	0.52
PCKKKSKPKVPAKN	1.56
CRHLKLVPSTPMDI	1.92
KQLMKNLPKIPLLN	2.11
AGQERFAPLAPMYY	2.01
FYKEKAKPLEPILD	1.40
RYCFKLLPPQPGSK	1.92
SKDKKTKPTPPPKP	1.85
REGPKSCPRGPLCP	1.48
YDLTRFLPNHPGGQ	1.31
AMESRVAPEIPGLI	1.78
TSWARDNPSKPDIA	1.54
KFDAKGRPIPPHPD	1.77
QLQEKLSPDLPDEE	1.77
NTIIKLFPYIPIPV	1.78
FYDFRSLPKVPTTQ	2.42
DSVLRQKPDKPRPI	1.64
VWGPKPVPFQPMDV	2.05
EYFPREYPKDPVSE	2.02
YVKERRPPPPPPLL	2.21
NEFAKFAPDLPVLK	1.84
KLPKKFPPPTPLLE	1.00
PIRLRKRPPPPPPV	2.46
GTGRRRSPSTPIMP	1.97
KQKKKPTPTRPYNY	1.50
LEFPKDYPLSPPKL	2.06
TLNDRQIPLSPDQM	2.08
AVVYKLKPYIPSLS	1.50
LNKTKLLPQSPILN	1.55
KDEVKLHPPEPKDL	1.51
EFTTREGPHQPIGN	1.47
YPNGRTLPPVPTQV	2.96
KVNFRRIPTGPDSP	2.20
NSFSKNDPNFPILK	1.92
AGPRKYIPRAPAQG	1.97
INIAKAHPNFPIAL	1.02
TSSLRKPPNGPSSV	2.66
FVSPRRAPKPPSYP	1.62
VVPERVKPAPPVSR	1.73
RDDVKRTPFIPESV	1.94
VNTPRSVPNSPNDG	1.77
LKKQKAIPSSPCGM	1.55
TNGPRYSPQNPRPF	1.93
VFVEKSKPTYPGTE	1.81
GKDHRLAPNGPGMH	0.49
MGLNKDVPHCPESL	2.40
AFYYKYYPVEPNCN	1.41
SERRKALPKEPKKL	1.94
LPLPKLRPPRPTFA	1.94
RRKLRTVPGVPLIH	3.80
FQPQRTAPKPPISA	2.17
PPLPRAPPVPPATF	1.25
LSPAKARPSFPHND	2.24
FQDYRCSPWCPSRS	2.76
SILNRCVPKRPTID	4.45
SMDSKRKPSPPSQR	2.57
LQNAKNDPLPPVIE	1.67
STNAKKDPFPPPKD	1.60
KRKAKAPPPPPPPP	1.49
GDEERFLPNRPHYE	1.03
SFVTKYLPAAPVGS	2.00
YTYSRNEPVIPTKP	1.84
RSRSKKDPGIPSNF	2.20
VLFMKGTPEFPKCG	2.43
VHFYKSLPQGPAPA	1.41
SYISRKYPRAPAFI	2.00
GLMSKLGPNLPASI	2.44
PQTMRQAPKRPDAD	3.57
DENSKWQPSDPFTM	1.48
KMSAKNTPKHPVDI	2.48
MAFIKILPNNPWPF	1.67
RHSKKPAPPPPGMQ	1.75
FEYSKYRPLTPSSE	2.64
GDHPKGPPPPPPPP	1.04
ALLRRTIPKRPFYH	3.27
DVNHRYPPMAPTTV	1.72
NIPTKSPPKSPKHK	1.81
EFDLKNIPMMPAGE	1.87
HEREKALPPIPFTT	1.63
EKLIKITPDTPFGF	1.67
PPSIRKQPIAPDVL	1.69
LASDKGLPMIPAFV	1.71
IAGQRTFPEDPNYP	1.46
KNLLREIPQVPAFQ	3.31
WLDEKRIPSTPIDY	2.95
AKTNKNVPVLPTPG	2.13
VKKEKKPPKIPKKV	1.85
ILPPRIVPAIPKKA	4.25
QNGLRFNPDVPKLW	1.34
IKMSRSPPRPPSLK	3.06
MDKFRIPPKSPNIE	2.29
YVNAKLDPSLPSQY	1.61
FKVYRWNPDEPSAK	2.00
VEVDKKVPVLPMVA	2.26
NYHHRETPPPPPSN	2.11
SSKIRPTPRKPSRM	3.90
NKPKRKRPAPPKKK	1.92
EFLRKHSPKAPATG	2.10
YVYGRQDPKKPFFT	2.30
INTFKTLPTKPEMQ	1.87
KALPKRRPSPPLQS	2.96
AISKKLGPDTPIKV	1.17
PGGPKFEPLYPREE	2.24
LNPNRRAPRRPLST	3.09
ETTTRETPLPPIPA	2.22
ISADKGFPLIPSGY	1.51
TSISRISPQDPEAK	0.93
GHKKKNLPKFPKNK	1.50
NELIKQGPKRPTSA	1.78
KALWKIQPYLPEFS	1.62
DRKNRAPPQRPRFN	2.52
EEKVKRTPPLPPVP	2.10
LNFPKNIPTIPFYV	1.72
CNYSKIFPGIPISL	1.93
TTNSRLCPSSPSSS	2.31
RFWTRARPIDPNAY	3.21
SLTLKTLPRVPTDS	2.21
LNFKRLLPRDPSEK	3.21
GQIDRDKPSEPERT	1.68
LFDEKVKPQIPQLR	1.82
DEFNRNTPPQPSLL	1.16
INGEKACPFLPSSL	1.70
TTTIKNRPVPPPLP	2.78
NDNEKKKPSSPTAA	1.91
SIIPRIFPSTPGTI	2.57
KKVIRNYPGHPEGT	1.46
KQSKRARPSSPYTV	2.01
RPVYRLKPNAPFQY	2.17
GRHLKNIPRIPIFC	2.25
PSSKKNKPNPPPGS	1.61
TDTTKVTPIPVPVI	1.39
ENENKKLPPAPTVF	1.34
WLGNKYDPENPFND	2.17
ETGVRYGPEFPLCT	1.08

ADDLKYIPPIPQDF	1.77
RLLFRSLPPHPACL	2.76
ISHNKLSPSEPILC	1.29
LIWHKSKPVKPTNK	2.03
DDNVRPSPISPSIN	1.37
RRIRKEHPGEPNVI	2.09
PEWRRPHPNIPDAK	1.00
VDSFKYEPLAPGND	1.34
DKKKKKYPRKPKKN	2.05
GFQDRSLPHFPKNS	3.71
LNATKISPSAPSKD	1.50
EHLQKERPKPPNKG	1.74
EFLIKEDPDLPPQG	1.82
NAPPRIFPKAPIYG	2.15
NRKQRSIPLSPIVP	2.26
QSEEKEKPKGPMAV	1.56
EKEAKSEPQQPEDN	1.73
LNLSKDAPVPPAPE	1.73
KLNKRVLPSKPKKS	3.14
SETARKVPPIPTQI	1.48
KDFSKNAPYNPSEV	1.62
WFILKLFPHEPIEG	1.50
RAGNREPPSTPSTL	2.13
NNLKKRPPTAPQRK	1.81
LKDPKSFPPPPLKP	1.90
HEVPKRSPEKPSNP	1.81
LDKGKHVPSKPVSN	1.88
TMKNKPKPTPPSPP	1.68
YFVKKIRPGNPLTK	2.14
SYMDKIYPDLPNDK	2.14
LPEPRFYPLSPKNN	1.52
KKIIKDVPGTPSDK	2.19
LPEKKNSPTTPSVF	1.74
VEMKRKKPVRPVDI	1.83
EIPSKDHPYDPEKD	1.49
NPIPKSVPLHPKSG	1.46
PTVQKFSPHPPTTL	1.65
PPPPRRGPAPPPPP	0.91
SQKKKALPKQPKAS	3.51
KLVLRNVPILPILN	1.38
TYIWRYLPAPPVCI	2.82
TQMKRLKPVSPGLR	2.62
LAPKREAPKPPANT	1.85
KTSTKAQPAEPQAE	0.81
KVSSKYDPDNPNKD	1.50
PTANKYAPVSPTVQ	1.58
PIGSRYNPNLPVTE	1.56
VCHSKYIPLKPIQD	1.40
EKVFRGDPEHPAIS	1.88
LAAHKTKPSIPPKK	1.88
EKESRHYPIKPPSP	1.65
DLSLRYAPNLPRVI	1.54
LTSFKGRPKPPKTL	2.28
PQQNRPLPQLPNRN	4.50
HICDKMNPASPQYF	2.06
KEFFRLTPNQPVGL	1.41
HLLGKEGPSAPNPS	1.84
KDKARGNPEKPPYW	1.70
KVIRKGSPKSPAMV	1.70
YGDNKKNPESPESV	1.40
PCKKKSKPKVPAKN	2.68
CRHLKLVPSTPMDI	2.35
KQLMKNLPKIPLLN	4.56
AGQERFAPLAPMYY	1.37
FYKEKAKPLEPILD	1.80
RYCFKLLPPQPGSK	4.19
SKDKKTKPTPPPKP	2.52
REGPKSCPRGPLCP	1.44
YDLTRFLPNHPGGQ	1.78
AMESRVAPEIPGLI	1.68
TSWARDNPSKPDIA	1.61
KFDAKGRPIPPHPD	1.43
QLQEKLSPDLPDEE	1.43
NTIIKLFPYIPIPV	1.63
FYDFRSLPKVPTTQ	4.48
DSVLRQKPDKPRPI	1.42
VWGPKPVPFQPMDV	1.38
EYFPREYPKDPVSE	1.49
YVKERRPPPPPPLL	1.80
NEFAKFAPDLPVLK	2.00
KLPKKFPPPTPLLE	1.84
PIRLRKRPPPPPPV	1.99
GTGRRRSPSTPIMP	1.72
KQKKKPTPTRPYNY	1.99
LEFPKDYPLSPPKL	1.90
TLNDRQIPLSPDQM	1.48
AVVYKLKPYIPSLS	1.97
LNKTKLLPQSPILN	3.28
KDEVKLHPPEPKDL	1.70
EFTTREGPHQPIGN	1.64
YPNGRTLPPVPTQV	4.24
KVNFRRIPTGPDSP	2.07
NSFSKNDPNFPILK	2.03
AGPRKYIPRAPAQG	1.80
INIAKAHPNFPIAL	1.63
TSSLRKPPNGPSSV	2.00
FVSPRRAPKPPSYP	1.98
VVPERVKPAPPVSR	1.63
RDDVKRTPFIPESV	1.66
VNTPRSVPNSPNDG	1.68
LKKQKAIPSSPCGM	2.31
TNGPRYSPQNPRPF	0.87
VFVEKSKPTYPGTE	1.91
GKDHRLAPNGPGMH	1.56
MGLNKDVPHCPESL	1.28
AFYYKYYPVEPNCN	1.71
SERRKALPKEPKKL	4.47
LPLPKLRPPRPTFA	1.88
RRKLRTVPGVPLIH	1.93
FQPQRTAPKPPISA	1.60
PPLPRAPPVPPATF	1.47
LSPAKARPSFPHND	1.73
FQDYRCSPWCPSRS	2.98
SILNRCVPKRPTID	1.98
SMDSKRKPSPPSQR	1.95
LQNAKNDPLPPVIE	-0.70
STNAKKDPFPPPKD	1.93
KRKAKAPPPPPPPP	1.24
GDEERFLPNRPHYE	1.58
SFVTKYLPAAPVGS	3.40
YTYSRNEPVIPTKP	1.65
RSRSKKDPGIPSNF	2.07
VLFMKGTPEFPKCG	1.71
VHFYKSLPQGPAPA	3.73
SYISRKYPRAPAFI	1.70
GLMSKLGPNLPASI	1.80
PQTMRQAPKRPDAD	2.08
DENSKWQPSDPFTM	1.44
KMSAKNTPKHPVDI	2.01
MAFIKILPNNPWPF	2.22
RHSKKPAPPPPGMQ	2.24
FEYSKYRPLTPSSE	2.06
GDHPKGPPPPPPPP	0.80
ALLRRTIPKRPFYH	1.54
DVNHRYPPMAPTTV	1.59
NIPTKSPPKSPKHK	1.72
EFDLKNIPMMPAGE	1.57
HEREKALPPIPFTT	4.76
EKLIKITPDTPFGF	1.57
PPSIRKQPIAPDVL	1.41
LASDKGLPMIPAFV	4.18
IAGQRTFPEDPNYP	1.64
KNLLREIPQVPAFQ	1.93
WLDEKRIPSTPIDY	1.91
AKTNKNVPVLPTPG	1.96
VKKEKKPPKIPKKV	1.85
ILPPRIVPAIPKKA	1.59
QNGLRFNPDVPKLW	1.58
IKMSRSPPRPPSLK	2.35
MDKFRIPPKSPNIE	1.54
YVNAKLDPSLPSQY	1.47
FKVYRWNPDEPSAK	1.88
VEVDKKVPVLPMVA	1.49
NYHHRETPPPPPSN	1.85
SSKIRPTPRKPSRM	2.16
NKPKRKRPAPPKKK	1.95
EFLRKHSPKAPATG	1.96
YVYGRQDPKKPFFT	2.07
INTFKTLPTKPEMQ	4.16
KALPKRRPSPPLQS	2.88
AISKKLGPDTPIKV	1.26
PGGPKFEPLYPREE	1.61
LNPNRRAPRRPLST	2.77
ETTTRETPLPPIPA	3.01
ISADKGFPLIPSGY	1.52
TSISRISPQDPEAK	1.46
GHKKKNLPKFPKNK	3.67
NELIKQGPKRPTSA	1.51
KALWKIQPYLPEFS	2.25
DRKNRAPPQRPRFN	2.13
EEKVKRTPPLPPVP	1.68
LNFPKNIPTIPFYV	2.12
CNYSKIFPGIPISL	1.44
TTNSRLCPSSPSSS	1.76
RFWTRARPIDPNAY	2.25
SLTLKTLPRVPTDS	4.50
LNFKRLLPRDPSEK	3.13
GQIDRDKPSEPERT	1.64
LFDEKVKPQIPQLR	1.27
DEFNRNTPPQPSLL	1.16
INGEKACPFLPSSL	1.28
TTTIKNRPVPPPLP	1.70
NDNEKKKPSSPTAA	1.60
SIIPRIFPSTPGTI	2.80
KKVIRNYPGHPEGT	1.81
KQSKRARPSSPYTV	2.00
RPVYRLKPNAPFQY	1.78
GRHLKNIPRIPIFC	2.30
PSSKKNKPNPPPGS	1.86
TDTTKVTPIPVPVI	1.48
ENENKKLPPAPTVF	3.55
WLGNKYDPENPFND	1.80
ETGVRYGPEFPLCT	1.36

EHLQKERPKPPNKG	2.92
EFLIKEDPDLPPQG	0.91
NAPPRIFPKAPIYG	3.32
NRKQRSIPLSPIVP	3.10
QSEEKEKPKGPMAV	2.04
KLNKRVLPSKPKKS	3.43
SETARKVPPIPTQI	4.29
WFILKLFPHEPIEG	3.13
LKDPKSFPPPPLKP	1.80
HEVPKRSPEKPSNP	2.17
TMKNKPKPTPPSPP	2.77
YFVKKIRPGNPLTK	2.39
SYMDKIYPDLPNDK	2.35
LPEPRFYPLSPKNN	2.31
KKIIKDVPGTPSDK	1.22
LPEKKNSPTTPSVF	1.06
VEMKRKKPVRPVDI	3.41
NPIPKSVPLHPKSG	1.94
PTVQKFSPHPPTTL	1.01
PPPPRRGPAPPPPP	2.33
SQKKKALPKQPKAS	2.49
KLVLRNVPILPILN	3.18
TYIWRYLPAPPVCI	2.72
LAPKREAPKPPANT	1.68
KTSTKAQPAEPQAE	2.21
KVSSKYDPDNPNKD	1.65
PTANKYAPVSPTVQ	0.00
PIGSRYNPNLPVTE	2.29
VCHSKYIPLKPIQD	1.54
EKVFRGDPEHPAIS	1.69
LAAHKTKPSIPPKK	3.24
EKESRHYPIKPPSP	1.50
DLSLRYAPNLPRVI	2.19
LTSFKGRPKPPKTL	2.33
PQQNRPLPQLPNRN	4.56
HICDKMNPASPQYF	2.21
KEFFRLTPNQPVGL	2.22
HLLGKEGPSAPNPS	1.16
KDKARGNPEKPPYW	1.80
KVIRKGSPKSPAMV	2.25
YGDNKKNPESPESV	1.12
PCKKKSKPKVPAKN	2.75
CRHLKLVPSTPMDI	2.74
KQLMKNLPKIPLLN	2.90
AGQERFAPLAPMYY	1.99
RYCFKLLPPQPGSK	2.55
SKDKKTKPTPPPKP	2.32
REGPKSCPRGPLCP	2.27
YDLTRFLPNHPGGQ	2.17
AMESRVAPEIPGLI	2.71
TSWARDNPSKPDIA	2.42
KFDAKGRPIPPHPD	2.35
NTIIKLFPYIPIPV	1.93
FYDFRSLPKVPTTQ	3.48
DSVLRQKPDKPRPI	2.06
VWGPKPVPFQPMDV	3.00
EYFPREYPKDPVSE	1.97
YVKERRPPPPPPLL	2.52
NEFAKFAPDLPVLK	2.12
KLPKKFPPPTPLLE	1.60
PIRLRKRPPPPPPV	2.47
GTGRRRSPSTPIMP	2.90
KQKKKPTPTRPYNY	2.83
LEFPKDYPLSPPKL	1.81
TLNDRQIPLSPDQM	1.78
AVVYKLKPYIPSLS	2.12
LNKTKLLPQSPILN	1.86
EFTTREGPHQPIGN	1.00
YPNGRTLPPVPTQV	3.17
KVNFRRIPTGPDSP	2.37
NSFSKNDPNFPILK	1.77
AGPRKYIPRAPAQG	2.37
TSSLRKPPNGPSSV	2.08
FVSPRRAPKPPSYP	2.92
VVPERVKPAPPVSR	2.68
VNTPRSVPNSPNDG	2.82
LKKQKAIPSSPCGM	2.41
TNGPRYSPQNPRPF	2.67
GKDHRLAPNGPGMH	1.47
MGLNKDVPHCPESL	2.86
SERRKALPKEPKKL	2.81
LPLPKLRPPRPTFA	4.22
RRKLRTVPGVPLIH	3.42
FQPQRTAPKPPISA	2.58
PPLPRAPPVPPATF	2.13
LSPAKARPSFPHND	3.37
FQDYRCSPWCPSRS	2.74
SMDSKRKPSPPSQR	2.92
LQNAKNDPLPPVIE	1.03
STNAKKDPFPPPKD	1.72
KRKAKAPPPPPPPP	1.29
GDEERFLPNRPHYE	3.11
SFVTKYLPAAPVGS	1.73
YTYSRNEPVIPTKP	2.38
RSRSKKDPGIPSNF	2.56
VLFMKGTPEFPKCG	2.82
VHFYKSLPQGPAPA	1.96
SYISRKYPRAPAFI	2.33
GLMSKLGPNLPASI	2.78
PQTMRQAPKRPDAD	4.20
DENSKWQPSDPFTM	1.11
KMSAKNTPKHPVDI	2.99
MAFIKILPNNPWPF	1.88
RHSKKPAPPPPGMQ	2.48
FEYSKYRPLTPSSE	2.69
GDHPKGPPPPPPPP	1.06
ALLRRTIPKRPFYH	3.85
DVNHRYPPMAPTTV	1.33
NIPTKSPPKSPKHK	2.71
EFDLKNIPMMPAGE	2.05
HEREKALPPIPFTT	2.20
EKLIKITPDTPFGF	1.79
PPSIRKQPIAPDVL	2.65
LASDKGLPMIPAFV	1.68
IAGQRTFPEDPNYP	1.95
KNLLREIPQVPAFQ	3.03
WLDEKRIPSTPIDY	3.22
AKTNKNVPVLPTPG	1.99
VKKEKKPPKIPKKV	2.89
ILPPRIVPAIPKKA	3.55
QNGLRFNPDVPKLW	1.94
IKMSRSPPRPPSLK	3.92
MDKFRIPPKSPNIE	3.32
YVNAKLDPSLPSQY	1.96
FKVYRWNPDEPSAK	2.19
VEVDKKVPVLPMVA	1.99
NYHHRETPPPPPSN	2.51
SSKIRPTPRKPSRM	3.99
NKPKRKRPAPPKKK	2.75
EFLRKHSPKAPATG	2.36
YVYGRQDPKKPFFT	2.20
INTFKTLPTKPEMQ	2.04
KALPKRRPSPPLQS	4.13
AISKKLGPDTPIKV	1.69
PGGPKFEPLYPREE	1.09
LNPNRRAPRRPLST	3.57
ETTTRETPLPPIPA	2.03
ISADKGFPLIPSGY	1.58
TSISRISPQDPEAK	1.31
GHKKKNLPKFPKNK	2.77
NELIKQGPKRPTSA	2.39
KALWKIQPYLPEFS	2.30
DRKNRAPPQRPRFN	3.57
EEKVKRTPPLPPVP	2.00
LNFPKNIPTIPFYV	1.43
CNYSKIFPGIPISL	2.05
TTNSRLCPSSPSSS	1.96
RFWTRARPIDPNAY	3.63
SLTLKTLPRVPTDS	2.27
LNFKRLLPRDPSEK	3.11
GQIDRDKPSEPERT	1.84
LFDEKVKPQIPQLR	1.63
DEFNRNTPPQPSLL	1.87
INGEKACPFLPSSL	1.08
TTTIKNRPVPPPLP	2.46
NDNEKKKPSSPTAA	1.57
SIIPRIFPSTPGTI	2.65
KKVIRNYPGHPEGT	1.54
KQSKRARPSSPYTV	2.67
RPVYRLKPNAPFQY	2.77
GRHLKNIPRIPIFC	2.98
PSSKKNKPNPPPGS	1.69
TDTTKVTPIPVPVI	1.07
ENENKKLPPAPTVF	1.21
WLGNKYDPENPFND	2.94
ETGVRYGPEFPLCT	0.86
GEMVKLTPKNPFYK	2.32
VCTIRSTPSQPIHC	2.25
FHPEKLTPYTPMTG	1.66
NPMQKPVPFVPVLD	3.39
TLKKKKTPKKPIAA	2.48
TKTDKSYPMCPVDA	1.70
SLGERFMPMAPEVY	1.20
ITSIRGSPSLPKNK	3.14
HFPSRSQPSDPKLQ	2.47
DIGSKKLPTGPSKK	1.70
TTRKKKDPNAPKRA	2.59
PSAMKLVPSIPPAT	2.40
LLEERNNPDIPVDK	1.59
QTVIKTHPRIPKSE	2.13
LITDKEYPVRPAYT	2.10
NLKTKSHPEVPRLD	2.51
IGSPRHAPSRPDSI	3.00
AVQLKRQPAQPRES	2.64
CWHPKTIPKQPMEI	3.11
QWSKRIMPYIPGPV	3.26
FVFFKSLPDLPKPQ	2.65
RGWQRFLPTGPIAH	3.32
KVLIKTLPVLPEAR	2.40
QTGSKTVPQAPQAP	0.82
VHVLKYAPDEPNSL	0.52
SDNSKSEPLPPVIT	1.33
LSFDRPPPLTPEKN	2.09

RLLFRSLPPHPACL	3.20
ISHNKLSPSEPILC	1.36
LIWHKSKPVKPTNK	1.86
LVGMRSFPLYPSGL	2.52
DDNVRPSPISPSIN	1.26
RRIRKEHPGEPNVI	1.78
PEWRRPHPNIPDAK	2.16
VDSFKYEPLAPGND	1.18
DKKKKKYPRKPKKN	1.20
GFQDRSLPHFPKNS	1.70
LNATKISPSAPSKD	0.92
EHLQKERPKPPNKG	1.74
EFLIKEDPDLPPQG	1.71
NAPPRIFPKAPIYG	2.27
NRKQRSIPLSPIVP	2.72
QSEEKEKPKGPMAV	1.26
EKEAKSEPQQPEDN	0.94
LNLSKDAPVPPAPE	1.91
KLNKRVLPSKPKKS	2.22
SETARKVPPIPTQI	3.91
KDFSKNAPYNPSEV	1.77
WFILKLFPHEPIEG	2.12
RAGNREPPSTPSTL	1.50
NNLKKRPPTAPQRK	3.63
LKDPKSFPPPPLKP	0.81
HEVPKRSPEKPSNP	1.79
LDKGKHVPSKPVSN	0.68
TMKNKPKPTPPSPP	2.34
YFVKKIRPGNPLTK	1.96
SYMDKIYPDLPNDK	1.57
LPEPRFYPLSPKNN	1.49
KKIIKDVPGTPSDK	1.30
LPEKKNSPTTPSVF	1.36
VEMKRKKPVRPVDI	2.18
EIPSKDHPYDPEKD	0.78
NPIPKSVPLHPKSG	2.67
PTVQKFSPHPPTTL	1.20
PPPPRRGPAPPPPP	1.60
SQKKKALPKQPKAS	1.79
KLVLRNVPILPILN	1.87
TYIWRYLPAPPVCI	2.24
TQMKRLKPVSPGLR	2.80
LAPKREAPKPPANT	1.47
KTSTKAQPAEPQAE	1.64
KVSSKYDPDNPNKD	1.51
PTANKYAPVSPTVQ	1.05
PIGSRYNPNLPVTE	1.94
VCHSKYIPLKPIQD	1.41
EKVFRGDPEHPAIS	1.83
LAAHKTKPSIPPKK	1.56
EKESRHYPIKPPSP	1.60
DLSLRYAPNLPRVI	1.29
LTSFKGRPKPPKTL	1.37
PQQNRPLPQLPNRN	4.70
HICDKMNPASPQYF	1.44
KEFFRLTPNQPVGL	1.17
HLLGKEGPSAPNPS	1.60
KDKARGNPEKPPYW	1.29
KVIRKGSPKSPAMV	0.94
YGDNKKNPESPESV	1.29
PCKKKSKPKVPAKN	1.79
CRHLKLVPSTPMDI	1.60
KQLMKNLPKIPLLN	1.98
AGQERFAPLAPMYY	1.20
FYKEKAKPLEPILD	1.76
RYCFKLLPPQPGSK	1.37
SKDKKTKPTPPPKP	1.36
REGPKSCPRGPLCP	1.56
YDLTRFLPNHPGGQ	1.35
AMESRVAPEIPGLI	1.66
TSWARDNPSKPDIA	1.69
KFDAKGRPIPPHPD	1.60
QLQEKLSPDLPDEE	1.21
NTIIKLFPYIPIPV	2.33
FYDFRSLPKVPTTQ	2.70
DSVLRQKPDKPRPI	0.96
VWGPKPVPFQPMDV	2.41
EYFPREYPKDPVSE	1.36
YVKERRPPPPPPLL	1.51
NEFAKFAPDLPVLK	0.90
KLPKKFPPPTPLLE	0.78
PIRLRKRPPPPPPV	1.41
GTGRRRSPSTPIMP	1.38
KQKKKPTPTRPYNY	1.65
LEFPKDYPLSPPKL	1.17
TLNDRQIPLSPDQM	1.63
AVVYKLKPYIPSLS	1.51
LNKTKLLPQSPILN	1.16
KDEVKLHPPEPKDL	1.28
EFTTREGPHQPIGN	1.54
YPNGRTLPPVPTQV	2.70
KVNFRRIPTGPDSP	1.65
NSFSKNDPNFPILK	1.76
AGPRKYIPRAPAQG	1.65
INIAKAHPNFPIAL	0.45
TSSLRKPPNGPSSV	1.58
FVSPRRAPKPPSYP	1.85
VVPERVKPAPPVSR	1.94
VNTPRSVPNSPNDG	2.32
LKKQKAIPSSPCGM	1.51
TNGPRYSPQNPRPF	1.64
VFVEKSKPTYPGTE	1.34
GKDHRLAPNGPGMH	1.39
MGLNKDVPHCPESL	2.38
AFYYKYYPVEPNCN	1.87
SERRKALPKEPKKL	1.69
LPLPKLRPPRPTFA	3.63
RRKLRTVPGVPLIH	2.89
FQPQRTAPKPPISA	1.32
PPLPRAPPVPPATF	1.03
LSPAKARPSFPHND	1.67
FQDYRCSPWCPSRS	2.34
SILNRCVPKRPTID	4.07
SMDSKRKPSPPSQR	2.23
LQNAKNDPLPPVIE	1.57
STNAKKDPFPPPKD	1.43
KRKAKAPPPPPPPP	1.06
GDEERFLPNRPHYE	2.81
SFVTKYLPAAPVGS	1.91
YTYSRNEPVIPTKP	1.20
RSRSKKDPGIPSNF	1.72
VLFMKGTPEFPKCG	2.11
VHFYKSLPQGPAPA	1.34
SYISRKYPRAPAFI	1.46
GLMSKLGPNLPASI	1.62
PQTMRQAPKRPDAD	3.79
DENSKWQPSDPFTM	1.00
KMSAKNTPKHPVDI	2.52
MAFIKILPNNPWPF	0.63
RHSKKPAPPPPGMQ	1.25
FEYSKYRPLTPSSE	2.46
GDHPKGPPPPPPPP	1.37
ALLRRTIPKRPFYH	2.99
DVNHRYPPMAPTTV	1.20
NIPTKSPPKSPKHK	2.30
EFDLKNIPMMPAGE	1.51
HEREKALPPIPFTT	1.18
EKLIKITPDTPFGF	1.54
PPSIRKQPIAPDVL	1.47
LASDKGLPMIPAFV	1.80
IAGQRTFPEDPNYP	1.20
KNLLREIPQVPAFQ	2.15
WLDEKRIPSTPIDY	2.25
AKTNKNVPVLPTPG	1.48
VKKEKKPPKIPKKV	1.52
ILPPRIVPAIPKKA	3.51
QNGLRFNPDVPKLW	0.92
IKMSRSPPRPPSLK	3.25
MDKFRIPPKSPNIE	2.67
YVNAKLDPSLPSQY	1.25
FKVYRWNPDEPSAK	1.47
VEVDKKVPVLPMVA	1.64
NYHHRETPPPPPSN	2.25
SSKIRPTPRKPSRM	2.84
NKPKRKRPAPPKKK	1.91
EFLRKHSPKAPATG	1.12
YVYGRQDPKKPFFT	1.52
INTFKTLPTKPEMQ	1.16
KALPKRRPSPPLQS	3.34
AISKKLGPDTPIKV	0.85
PGGPKFEPLYPREE	1.23
LNPNRRAPRRPLST	2.24
ETTTRETPLPPIPA	1.85
ISADKGFPLIPSGY	1.16
TSISRISPQDPEAK	1.28
GHKKKNLPKFPKNK	1.68
NELIKQGPKRPTSA	1.24
KALWKIQPYLPEFS	1.63
DRKNRAPPQRPRFN	2.21
EEKVKRTPPLPPVP	1.66
LNFPKNIPTIPFYV	1.36
CNYSKIFPGIPISL	1.78
TTNSRLCPSSPSSS	1.44
RFWTRARPIDPNAY	2.99
SLTLKTLPRVPTDS	1.45
LNFKRLLPRDPSEK	3.54
GQIDRDKPSEPERT	1.58
LFDEKVKPQIPQLR	1.20
DEFNRNTPPQPSLL	1.40
INGEKACPFLPSSL	1.52
TTTIKNRPVPPPLP	1.52
NDNEKKKPSSPTAA	1.31
SIIPRIFPSTPGTI	1.51
KKVIRNYPGHPEGT	1.15
KQSKRARPSSPYTV	1.82
RPVYRLKPNAPFQY	2.11
GRHLKNIPRIPIFC	1.84
PSSKKNKPNPPPGS	1.45
TDTTKVTPIPVPVI	0.81
ENENKKLPPAPTVF	1.39
WLGNKYDPENPFND	2.10
ETGVRYGPEFPLCT	1.61
GEMVKLTPKNPFYK	1.65

YSFRRPVWPA	1.44
RRLKRPRTQG	1.54
KKIKRPKFTV	1.63
LGPRRPMRPV	4.01
RGPKRPKVLQ	0.95
AAPRRPFSWE	1.45
EEPKRPPGIC	1.37
YDLRRPSFTR	2.15
LPPKRPELPP	1.49
PGPRRPERGP	4.13
AVLKRPSQLS	1.67
KWIRRPVGAS	1.61
HIFRRPKGLF	1.56
IPFKRPCTYG	1.55
TNPKRPLSSA	1.55
DSLRRPQLRL	1.17
TPLRRPGMLV	1.99
DVIRRPTFVP	1.36
AKLRRPMVIE	1.54
FGIRRPLDPL	1.56
HVLRRPIVVV	1.57
ETFRRPQIDP	1.36
EKIKRPRSSN	1.42
NLFKRPLPKS	1.40
SVPRRPGQRR	1.51
WDLRRPYEPI	1.65
LKLRRPVTVP	1.45
GQPRRPGQCP	1.31
GQLKRPRRNG	2.71
SAPRRPSSLQ	1.57
NAPKRPPLAF	1.51
PTFRRPKTLR	1.75
DIPKRPSYVA	1.68
FKLKRPLEDG	1.58
SCPRRPDYLK	1.38
QPPRRPWGNL	1.78
TELRRPDAVR	1.58
QTIKRPLNPL	0.95
RPIKRPAYWR	0.57
GSPRRPGPEP	1.65
LFPKRPALEP	1.61
NQPKRPPAAP	1.58
RHFRRPDPCR	1.37
ATLKRPEVIA	1.58
VNFRRPEDAR	1.24
PKPKRPAILT	1.52
CSPIRPPKYY	2.36
VEPKRPSRPI	3.67
STPKRPSTSQ	1.51
QPLRRPSSVG	1.57
MTPRRPGPTR	1.88
LLFRRPLPKK	1.54
ASFKRPVVIL	1.68
RPLKRPRLGP	1.49
VGIRRPFQIP	1.53
SILRRPYVTC	1.03
GQPRRPRNLA	1.82
GGPRRPERHL	3.52
KSLRRPETSY	1.59
FDIRRPSLEH	2.06
QSLRRPLWLS	1.82
FHPKRPMHIL	1.75
EELRRPEMKN	1.53
LAFRRPAEYD	1.46
SPPRRPQHNS	1.65
PSPRRPMSNL	1.88
VAPRRPRDAV	3.56
TSLRRPQDKD	1.43
VTPRRPFEKS	2.39
NAPKRPPSGF	1.30
NRLRRPGGSY	1.39
LEPKRPPPPR	1.46
NTLKRPKRPP	2.65
QGLRRPARPG	2.93
KGLRRPHCTV	1.65
TCLKRPLVVI	1.42
VNLKRPCPFW	1.68
LSPRRPVSPG	1.78
RPFRRPNANF	3.60
GHIKRPMNAF	1.68
EEPKRPLPRK	1.55
PLPRRPGHDD	1.57
MGIKRPAPQP	1.39
GRLRRPAHVP	1.32
DVLRRPSKDE	1.41
PELRRPRSEP	1.54
APPRRPRPPP	2.36
KGFRRPSEKN	1.52
KNIKRPPGDI	1.28
LDPRRPEIPT	1.42
VYLKRPAHSD	1.52
EVLKRPEYFG	1.64
PRLRRPMPWL	1.93
PELRRPGVQG	1.41
VILRRPERPM	1.50
WPLKRPGRIS	3.18
LKLRRPLYAH	1.50
KGPKRPQQPN	1.67
ATPRRPAGLL	1.68
HPFRRPKLSG	1.69
EKPKRPVSAM	1.59
LKIKRPYGRK	1.48
GKFKRPPLRR	1.55
SHIKRPMNAF	1.57
VTPRRPGRPD	3.72
PAPRRPASLA	1.57
VHIKRPEMTS	1.44
YFFKRPYPFV	1.81
RHFKRPAYAS	1.46
EILRRPIVAC	1.32
NGIRRPLQGV	1.24
RVPKRPIINT	1.73
HILKRPDRVL	2.94
VQLKRPKDAN	1.46
GEFRRPRLAS	1.65
YVLKRPHVDE	1.29
TPPKRPGGRR	1.84
LLIRRPECFG	1.19
AALKRPDDVP	1.58
LIFKRPSRQF	3.71
PAPKRPPDPF	1.71
PLPRRPSTPR	3.29
MYLRRPTEES	2.05
PAIRRPAEKF	1.56
LNIRRPLVKM	1.39
LLIKRPPFFH	1.53
RHPRRPPRSP	3.09
DRFRRPRDEG	1.66
NVLRRPFQAQ	1.50
FFIRRPTLDI	1.77
GRPKRPHARR	1.39
AFLKRPELGL	1.56
GPFRRPTTQL	2.53
MQLRRPSDRE	1.65
RSPKRPAWGS	1.67
EALKRPMGPV	1.06
QRIRRPFRPI	2.26
NFPRRPDSGD	1.48
QSPKRPRSPG	1.50
VPLKRPRLDK	1.66
ISLRRPNCFG	1.43
AGLRRPTYTP	1.50
KQPRRPHFLL	1.48
VILRRPLDYE	1.30
RQIRRPKNVV	1.20
KPPKRPRLQR	2.58
NELRRPPLCY	1.54
TPLRRPTETN	1.50
APPKRPAVAS	1.81
LELRRPMDSR	1.64
KWIRRPTGVS	1.63
KPIKRPVLDP	1.55
SFLKRPKSQV	1.57
LKIRRPHDYQ	1.57
LSLRRPPGPA	1.53
CAPRRPCAGD	1.37
IRLRRPPLRY	2.59
APPRRPRPPP	2.96
TALRRPSLRR	1.81
AFLKRPISNK	1.54
VELRRPGQSH	1.46
QHLRRPRGLS	1.50
LPPKRPLSSS	2.83
QEFRRPCTLS	1.59
LDLRRPPSDL	1.55
LYLRRPAADG	1.63
PRPRRPRQLT	1.86
KTLRRPRCLL	1.51
FDLRRPPQNH	1.80
QSIRRPSRQL	3.16
VHLRRPQSPG	1.50
GGLKRPSTRT	1.35
SRFKRPKPNL	1.57
CIPRRPNWNQ	2.21
QAPRRPLPQV	2.77
APLRRPDSSD	1.67
QALKRPDDTA	1.58
HELRRPVLQR	1.48
VSPRRPWSPS	2.35
LKLRRPSFRD	1.78
APIRRPISAE	3.00
VPFRRPSTFG	1.49
YMLRRPALED	1.90
ELPRRPLPPA	1.60
SMLRRPRTSS	2.06
RPLRRPRPVQ	2.92
LDLRRPMRTV	3.03
NQLRRPQYRP	1.11
SALKRPFEDG	1.44
RVLKRPPNSV	1.52
RSPKRPRLQL	1.27
MPLRRPKMKK	1.54
VEPKRPCRPI	3.94

SGTALLRPLRPKPP	2.17
KFLDPSRPYKCTVC	1.33
TMCCIYRPLKPRTN	1.99
SQQNFHRPVKVRKK	1.72
FNQALGRPEKIHNK	1.96
HGSFLARPSRKNQG	3.38
LCYCLYRPRKYLYV	2.14
RVPIPPRPVKPDYR	2.81
APVPLLRPHRPSGP	3.13
FSFSLPRPGRRGSR	3.04
LEQLLLRPERPGDT	1.84
ICTKLVRPKRKHGT	3.38
CDKAFCRPDKLRLH	2.07
XSQHFGRPRRADHE	2.66
NFGNLPRPGRGARG	2.98
KLDTPLRPARQQPT	3.34
KVHVPARPYRVLNN	3.61
ATCRFYRPPRCSHC	3.75
EASSPARPSKHSVG	2.36
IIHVISRPLKAPPA	1.92
LVIRLPRPPKVLRL	3.29
NSPTLERPGRKRKW	2.70
APANLERPVRYIRK	3.49
EMPHLARPIKVESP	2.16
RHWRPWRPDKTKKQ	2.76
RDFIISRPTKLKSL	2.03
GNANIQRPCRKSVN	3.51
HHGRIGRPPKYRKI	2.21
PRKYPNRPSKTPVH	2.04
QFPKLDRPTKQMRE	2.06
YAMGFNRPSKIQEN	2.38
AATQPLRPPRGTAA	4.08
VMARLSRPERPDLV	3.15
AVKVFHRPWRRGWH	2.90
ADVDPQRPPRPEVK	3.44
VVSSPPRPVRHGDM	3.47
EIGFPGRPGRPGMN	2.14
PAPSLPRPERKSIV	2.18
SQSFIIRPQKEGER	2.07
TPDLLPRPPRLLLL	2.92
EKIPFERPFRRPNA	2.98
LTKVFFRPGKFAEF	2.12
RYLAICRPLRARVL	3.12
TRYMFSRPFRKHGV	3.34
FVLRPLRPCRRYST	4.00
AEEPLHRPKKELSA	2.13
GSWCPMRPKKEAMK	2.23
HSFLINRPGKVKYM	2.31
SFAYPNRPDRPVLK	3.11
QQQLLQRPPRDAQQ	3.51
QPVNPPRPFKHSER	2.44
FNRYLCRPRRVEMA	2.53
DKADLIRPKRKYEK	2.62
HKAHLIRPRRLTPG	3.03
SSPEPPRPAKRPRR	2.79
QALYFCRPFRENVL	3.35
PLISFPRPVKTTWL	2.30
TKIMFNRPKKKNAI	1.91
TRYSPYRPYRCVHQ	3.00
GYGLLVRPSRVRRA	3.41
PRVDPYRPVRLPMQ	2.51
APPRLARPPRFYEA	2.85
IDPAILRPGRLDQL	2.48
RQQTLPRPMKTLQR	1.90
EVFFPPRPERVEHN	2.68
SEDDFYRPSREQPL	2.15
GAARLGRPGRSCLP	2.11
SAPSPARPARTQGP	3.63
AILTLPRPVRASLP	2.60
EKGELGRPGRKGRP	2.01
ALPPPPRPDRGPPG	2.93
EDFVPFRPRRSPRM	4.30
SAHTPGRPPRVTKD	3.29
SQGPPPRPGKPEGP	2.02
GSGKPSRPSKPIVA	2.10
FNKYLCRPRRVEIA	2.41
SSTNPFRPQKVCSF	1.89
RSRSLHRPAKTSEL	2.23
LLIIISRPARLLEC	1.96
FNKYLSRPKRFEVA	2.29
ILAKISRPKKRAKT	2.09
CVGRPTRPCRGPWR	3.98
DDSVILRPSKSVKL	2.08
GNLDIQRPKRKRKN	3.33
TPRPLARPARPNLS	3.48
LNPDFSRPNRKYTW	3.08
PDPGFLRPQRAALY	2.99
NEPLFVRPPKGSPQ	2.33
PPQVPSRPTRAPPS	3.94
PSPALPRPSRSSPG	2.89
VGKAFIRPFREHHI	4.07
RLFSIVRPRRLLYM	3.14
LKGIFTRPSKLTRK	2.08
GLLLLPRPGRPLDL	1.99
QLQAPDRPSRELLQ	3.52
YPQDLPRPLRSREF	2.19
LAEGINRPRKGHDA	2.00
DEPEPPRPRRGSLR	3.55
LDPALLRPGRLDRK	3.31
RKWYPLRPKKINLD	2.25
KRKKPSRPAKTRKR	2.04
EVEMLPRPPKVTKE	2.01
SGEQLPRPTRGRMR	2.94
RILQFPRPVKLEDL	2.03
YGPNIGRPRKNVIA	2.13
LTVTLQRPTKELHG	1.98
NETGLSRPRRTAMS	2.87
SIVEFFRPPKEVHL	2.22
DDLEIVRPARRRTP	2.28
DTPPPSRPEKKKAD	1.90
ELAHLVRPQKCRPY	2.22
SGMAFPRPKRPAPA	3.12
ANKILNRPKRYNSG	2.68
LSNCLTRPQKLSPV	2.08
MAQSIYRPSKNLDK	2.19
DGRLLYRPNKAGET	1.30
PRPSPARPKKSEES	2.01
SREGPARPYRLKPG	3.10
SCQRPGRPARVALP	3.35
DVLSPLRPSRRHFP	3.95
PATAPARPLRLPSG	4.05
ELYLLFRPFKGYEG	2.11
SVCFIGRPWRVVDG	2.05
SYRRLDRPTRIRFS	2.89
LIRFLPRPIRQQLL	3.24
LDSALLRPGRIDRK	3.21
KAICFLRPTKENVD	1.75
PECEIERPERLTAA	2.09
VPMPIARPARQTSR	3.98
EFGLPGRPGRPGLN	2.45
LREDFVRPLREGIL	3.72
AHLPPPRPQRPAET	2.40
ARRPLARPIKPNEI	1.96
APRYLGRPKKEKTI	0.85
PYPLILRPQRGVDL	2.54
EEEELARPPREQGQ	2.19
IKKDLSRPQKPQLV	2.03
QAGGPQRPGRVVSL	3.63
QASRFQRPGRRMSE	3.61
PAALLYRPVRQYVY	2.58
IRQILSRPERLLFI	2.99
FPTVPTRPEKGYIW	2.75
GRDFLQRPGRQPQA	2.32
KTHPLIRPFKTLTE	1.94
PPQIPSRPVRIPPG	3.95
APALPARPHRGLST	3.78
MTSSLQRPWRVPWL	2.70
LPPDIERPYKAEPL	1.88
PSRPPSRPSRPPSH	4.08
LPKGLGRPGKGHYW	2.32
FVYNFIRPVKGKVS	2.28
VPSLPIRPSRAPSR	4.33
KPSRPPRPSRPPPP	3.90
KNRTFLRPPKVKME	1.89
TREALSRPARTAPL	2.64
LGLRLCRPYRLAAG	3.22
LQSDPPRPHRILQA	2.23
ESRDITRPSRPADL	2.28
ISSQIERPIRIVAL	2.73
KSLRIARPPKQGLN	2.22
GTQELRRPAQLRLQ	1.68
ALLEPRRPPHDYMP	1.52
EVMILRRPSLQRRA	2.37
SSPGPKRPGNTLRK	1.72
EEEPPRRPRARSCE	0.62
IVYSFRRPVWPAVV	1.44
LERRLKRPRTQGVV	1.54
TAKKIKRPKFTVPQ	1.63
QKFSLRRPLQTAMI	1.81
VNRGPKRPKVLQRG	0.95
PAAAPRRPFSWEDV	1.45
VFRVLRRPQEFVNR	1.86
EGEEPKRPPGICER	1.37
WRYDLRRPSFTRTW	2.15
IEPQIRRPRSLSSP	2.05
VKLLIRRPECFGPA	1.60
GKLPPKRPELPPTL	1.49
KIVDPKRPLMPEEC	1.43
PVAVLKRPSQLSRK	1.67
PPKWIRRPVGASFS	1.61
YGHIFRRPKGLFIS	1.56
DKIPFKRPCTYGVP	1.55
QSTNPKRPLSSANQ	1.55
LGDSLRRPQLRLWS	1.17
VFTPLRRPGMLVPK	1.99
QKDVIRRPTFVPQW	1.36
EDAKLRRPMVIEII	1.54
LPENFRRPQTDQAV	1.31
KTFGIRRPLDPLDP	1.56
LAHVLRRPIVVVAD	1.57
LPETFRRPQIDPAV	1.36
NIEKIKRPRSSNAE	1.42
TVNLFKRPLPKSKP	1.40

RGWQRFLPTGPIAH	1.98
KVLIKTLPVLPEAR	2.15
QTGSKTVPQAPQAP	1.66
VHVLKYAPDEPNSL	2.02
LDPGKNTPGLPEET	1.58
LSFDRPPPLTPEKN	1.97
LPLSRVQPVQPSQT	1.50
ASFRKTKPPPPLDF	3.29
PKTGKPLPTQPSSS	2.78
QATGRRGPAPPPPP	2.01
TGESRKAPLIPLLK	0.92
LRISKPDPECPSKL	2.38
GDHPKGPPPPPPPD	1.49
NNSTRPIPAIPMDL	2.31
FEEDKMTPSTPVRS	1.08
YSYLRLLPWWPSLA	1.98
SFIRKRAPTPPAPS	3.51
FLQDRVIPIFPHYP	2.01
AEIVKLTPKNPFYK	2.19
NIFQKQLPKMPKEY	2.81
LTKIRRRPQQPLTD	3.49
IQQNKQGPKIPFYL	2.03
AVGGKWEPGNPIAI	1.61
IESFRERPLEPPRK	1.67
NSVIRGCPGLPDTL	2.36
TEVWRKVPMKPIFE	2.21
DFRGRAYPLSPHFN	3.70
NQIARQIPYQPWYL	2.02
ALSTKGYPHEPPLL	2.28
EEEAKSVPPIPADQ	2.06
SRYLKDHPNLPTPE	2.32
GSYIKRTPKIPEKD	2.11
FASEKGAPFFPNKT	1.78
YKNRKNLPTIPIRL	4.09
RLIKRLKPPAPKEP	2.57
NLSLRYAPNLPPVI	2.56
APVVKSEPSLPRMR	3.29
NDMAKPLPTPPTAE	1.85
VVAVKIIPKKPWNA	1.93
SSVSKLKPSLPNKL	2.52
YVNPRNLPSVPNGL	2.24
AFVPKRKPSLPQLA	3.44
PKSPKAYPKLPDIQ	2.61
GIMIKAKPSSPYTY	1.84
PVRCRVLPATPRQI	3.31
AKDYRFNPAIPLRI	3.15
TVRTRRRPPPPPIP	3.54
SEMHRAAPETPRSI	2.18
ITAEKSAPTSPEVY	1.97
KITKKLGPKLPKAS	2.30
KMAKKLLPYKPHNS	2.19
VTDIKLTPTDPLVG	1.85
KKHDKLHPNYPSVK	2.83
NPFVRINPNRPEAA	2.65
ALEERFDPKSPTKR	2.98
EFQSKSSPVPPASE	3.16
TSSSRRGPGRPRKD	2.62
DKIFKTDPNEPAND	1.82
KQRIRKIPGIPLMS	2.90
ALLIKTVPGFPLLE	1.89
FSKKKEAPEDPKNL	1.75
TDLFKPLPEPPTEL	1.51
LMSLKRFPQLPSNT	2.90
LSRSKPLPLTPNSK	2.81
ITRARYMPQDPNIV	3.35
FGTAKILPEEPSNT	1.55
PDLNKRPPREPIEF	2.06
NGMPRRIPDYPDAF	2.61
ILKNRMNPEKPYEL	2.51
VNIGRRSPYLPLQP	3.02
LDVRRIYPKGPLLV	3.67
HKFVKSSPVVPFDD	2.39
KHEPKKSPTTPALA	2.65
LNRQKNYPDAPAIF	1.85
KNILKVCPVLPSIL	2.48
YILPKTDPRSPYYI	2.35
DQLNRLLPNLPEEE	2.02
PFLIKFTPKFPQSI	1.81
GNNSRNIPPPPPPP	1.79
DRLSKIKPFLPQSI	2.91
QLINRFMPHFPSSP	2.44
AKIDKVSPEDPWVL	1.74
VLKIKPLPYKPRGR	3.20
PANPKILPKIPSGA	2.20
STDHRGTPANPGRV	1.42
WRWARDTPRAPGLH	1.67
LIWHKNKPTEPDNK	2.08
WFDLKGLPRQPEPL	1.89
TINSRFYPPIPISE	1.98
KKHDKLYPKYPSVK	2.64
KKNPKKSPMGPQGA	2.86
HLVRKVAPTKPMYC	2.45
WENFKEEPGEPLSR	2.02
LIDFKDYPDVPKVP	1.19
KQAKRAPPSSPYVV	2.67
LNQSKGQPFQPSPT	2.14
HTIPKSGPIVPYVL	1.77
AKINKVSPEDPWVL	2.07
RPKRRAPPPVPKKP	3.07
KNPTKSPPPPPSPS	1.56
NTLLKVVPQEPTAV	3.05
TKFTKPKPKQPVLP	2.47
QPPDRNLPSHPSSN	1.29
SVSEKTNPSSPSVS	2.09
EELARVTPLTPKEY	1.82
QIVNKPLPPLPVAG	1.75
TSGYKYFPQFPQLK	1.98
LDLSKYGPLPPSRA	3.21
RYPFRMIPELPPKR	4.15
DEPIRYAPGDPIEK	1.76
VKPTKLEPENPQKP	1.70
NSTVRRKPHSPNKK	2.68
PKHIRNEPVKPFRN	3.22
VKATKWPPKLPEIQ	2.23
RIRDKSKPSSPTAT	3.18
EFPGKKCPRLPHRN	3.04
AERAKKAPSLPEGQ	2.97
SKIPKSIPIIPYVL	2.18
AVLIRADPLNPKVV	1.18
LPFPRKNPKLPENT	2.46
RLLSKYRPSCPIIL	2.86
GTVTKTAPSFPQSS	2.16
GTTNRGPPPLPPRA	4.70
LAGIRLVPVLPILL	1.80
IPYEKRDPTSPAPL	1.64
LGSEKSCPLAPAVA	1.86
QVYEKTIPLFPPEI	1.04
FGSNKNNPVPPELT	1.77
LKDNRLLPSWPKRG	2.18
GKMVKWGPLRPFLS	1.81
EVLRKYIPGEPEFL	1.67
LQQRRYYPIFPGSI	3.79
QRLDRDKPHEPNGY	2.02
VVNEKEVPAEPETQ	2.11
TGKFRYMPFSPAGT	2.67
EFIVKTIPVPPRDF	1.18
REYIKSLPMYPAAP	2.17
LKHTKNKPKNPDTA	3.27
IVNEKRTPALPSNL	1.87
LNTMKKRPAPPSLP	1.99
YYYYKHYPNMPPIN	2.36
SNFKKRDPGLPDFQ	2.38
KTRNKPKPLWPQVK	3.08
AKVNKCPPDHPYIQ	2.63
ETKKKWQPLPPEPL	2.06
VLGPKYDPKCPEEH	1.46
NTINKSNPQGPSDN	2.49
TARNKKSPGGPRQR	2.44
SILRKSSPEEPFMS	2.12
PPQMRSNPQQPPQD	1.96
MYDRKVIPRMPWHD	3.89
TGGGRIVPGVPPGA	1.90
MLFMKGSPSEPKCG	1.49
FLLKKMFPAPPLPT	2.37
ATGKRKLPGWPSAD	2.10
HSTPKPLPPVPALS	0.04
KPAGRPIPPAPTHY	1.96
ELDIKQGPVRPFIV	1.94
LQESKTVPQSPKKP	1.36
PPNYKQQPNCPSSN	1.79
PIVKKNVPKPPPLV	2.42
RLVNKLLPPHPFAD	2.64
MYGSKFIPFGPKCQ	2.12
QGSNKYSPSSPLTT	1.54
PKCEKIEPLSPSFK	2.73
AGLYKLSPLYPFDV	2.03
VYRRKCIPFAPHQI	3.41
VLTVKMSPSLPDEI	2.24
GTRPKILPKIPTGA	2.17
WSQMRMNPTNPDWI	2.06
DNELKDEPEPPNLI	1.24
KAALKYHPDKPTGD	1.33
NFPQRMMPPPPGLV	2.36
LAYERPLPDLPSTI	1.62
MKAYRANPPLPIDS	2.12
PFIRRYTPLKPITK	2.72
YAVGRIVPDSPTYD	1.94
FSTKKSKPIRPFVF	2.43
IESLREIPYGPDFD	1.21
QHKPKVKPSKPEAK	3.41
KTKKKTVPVKPLQL	3.09
TELLKTLPFYPTPS	1.76
IGKSKSAPPIPEDT	1.87
KKNKKKVPLNPLAK	2.73
MTQPKKNPMEPKTD	1.58
ITDTKLFPGNPNLM	2.05
AGHIKTHPNSPTPG	1.80
KIVRRDYPSTPVIV	2.37
ETSLRVLPIAPKIF	2.63
SKRMKALPKGPAAL	3.24
ERDGRGVPLGPRNH	2.57
LWRPRIVPILPYIT	3.92
AKSNKVSPEDPAVQ	2.76

GDHPKGPPPPPPPD	0.23
NNSTRPIPAIPMDL	1.32
FEEDKMTPSTPVRS	0.90
YSYLRLLPWWPSLA	1.73
SFIRKRAPTPPAPS	1.49
FLQDRVIPIFPHYP	0.08
AEIVKLTPKNPFYK	1.48
NIFQKQLPKMPKEY	2.31
LTKIRRRPQQPLTD	1.93
IQQNKQGPKIPFYL	1.09
AVGGKWEPGNPIAI	1.15
IESFRERPLEPPRK	2.08
NSVIRGCPGLPDTL	1.54
TEVWRKVPMKPIFE	1.52
DFRGRAYPLSPHFN	3.14
NQIARQIPYQPWYL	1.58
ALSTKGYPHEPPLL	1.10
EEEAKSVPPIPADQ	0.65
GSYIKRTPKIPEKD	1.51
FASEKGAPFFPNKT	2.43
YKNRKNLPTIPIRL	1.69
RLIKRLKPPAPKEP	1.61
NLSLRYAPNLPPVI	1.40
APVVKSEPSLPRMR	1.25
NDMAKPLPTPPTAE	1.73
VVAVKIIPKKPWNA	1.49
SSVSKLKPSLPNKL	1.70
YVNPRNLPSVPNGL	1.77
AFVPKRKPSLPQLA	1.22
PKSPKAYPKLPDIQ	1.37
GIMIKAKPSSPYTY	1.73
PVRCRVLPATPRQI	1.56
AKDYRFNPAIPLRI	1.48
TVRTRRRPPPPPIP	1.10
SEMHRAAPETPRSI	1.46
KITKKLGPKLPKAS	1.32
VTDIKLTPTDPLVG	-1.00
KKHDKLHPNYPSVK	1.52
NPFVRINPNRPEAA	1.67
ALEERFDPKSPTKR	1.13
TSSSRRGPGRPRKD	1.37
DKIFKTDPNEPAND	1.42
KQRIRKIPGIPLMS	2.03
ALLIKTVPGFPLLE	1.68
FSKKKEAPEDPKNL	1.26
TDLFKPLPEPPTEL	1.36
LSKLRLSPENPSNT	1.52
TTKHKAPPPPPPTA	1.32
LMSLKRFPQLPSNT	2.47
LSRSKPLPLTPNSK	1.94
ITRARYMPQDPNIV	1.85
FGTAKILPEEPSNT	0.89
PDLNKRPPREPIEF	1.03
NGMPRRIPDYPDAF	2.50
ILKNRMNPEKPYEL	2.17
VNIGRRSPYLPLQP	1.28
LDVRRIYPKGPLLV	1.03
HKFVKSSPVVPFDD	0.86
YWGKKFEPERPFEF	2.43
KHEPKKSPTTPALA	1.56
LNRQKNYPDAPAIF	0.90
KNILKVCPVLPSIL	-0.10
YILPKTDPRSPYYI	1.65
DQLNRLLPNLPEEE	1.26
GNNSRNIPPPPPPP	1.15
DRLSKIKPFLPQSI	1.21
QLINRFMPHFPSSP	2.51
VLKIKPLPYKPRGR	1.54
PANPKILPKIPSGA	-0.05
WRWARDTPRAPGLH	3.57
LIWHKNKPTEPDNK	1.47
WFDLKGLPRQPEPL	2.61
TINSRFYPPIPISE	0.84
KKNPKKSPMGPQGA	1.82
HLVRKVAPTKPMYC	1.84
WENFKEEPGEPLSR	1.91
LIDFKDYPDVPKVP	1.68
KQAKRAPPSSPYVV	0.46
HTIPKSGPIVPYVL	1.52
AKINKVSPEDPWVL	1.11
RPKRRAPPPVPKKP	1.42
KNPTKSPPPPPSPS	1.22
NTLLKVVPQEPTAV	1.80
TKFTKPKPKQPVLP	0.79
QPPDRNLPSHPSSN	1.14
LLPTKLIPSIPGGK	0.54
SVSEKTNPSSPSVS	1.47
EELARVTPLTPKEY	1.62
QIVNKPLPPLPVAG	1.14
TSGYKYFPQFPQLK	1.52
LDLSKYGPLPPSRA	1.31
RYPFRMIPELPPKR	2.74
DEPIRYAPGDPIEK	0.00
VKPTKLEPENPQKP	0.94
NSTVRRKPHSPNKK	2.01
VKATKWPPKLPEIQ	-0.10
DYRPKCIPCPPNGI	0.78
RIRDKSKPSSPTAT	0.49
EFPGKKCPRLPHRN	1.63
AERAKKAPSLPEGQ	1.63
SKIPKSIPIIPYVL	1.41
LPFPRKNPKLPENT	1.58
RLLSKYRPSCPIIL	1.70
GTVTKTAPSFPQSS	2.13
GTTNRGPPPLPPRA	1.49
LAGIRLVPVLPILL	1.35
LGSEKSCPLAPAVA	1.72
QVYEKTIPLFPPEI	0.56
FGSNKNNPVPPELT	1.13
LKDNRLLPSWPKRG	1.34
GKMVKWGPLRPFLS	2.19
LQQRRYYPIFPGSI	2.67
QRLDRDKPHEPNGY	1.08
VVNEKEVPAEPETQ	1.53
TGKFRYMPFSPAGT	2.01
EFIVKTIPVPPRDF	1.14
REYIKSLPMYPAAP	1.92
LKHTKNKPKNPDTA	1.41
IVNEKRTPALPSNL	1.32
LNTMKKRPAPPSLP	1.86
YYYYKHYPNMPPIN	2.44
SNFKKRDPGLPDFQ	0.79
KTRNKPKPLWPQVK	1.64
AKVNKCPPDHPYIQ	1.31
ETKKKWQPLPPEPL	1.12
VLGPKYDPKCPEEH	0.28
NTINKSNPQGPSDN	1.49
TARNKKSPGGPRQR	1.66
SILRKSSPEEPFMS	1.63
PPQMRSNPQQPPQD	2.29
MYDRKVIPRMPWHD	2.60
TGGGRIVPGVPPGA	1.34
MLFMKGSPSEPKCG	2.11
FLLKKMFPAPPLPT	1.45
ATGKRKLPGWPSAD	0.60
HSTPKPLPPVPALS	1.45
ELDIKQGPVRPFIV	1.52
LQESKTVPQSPKKP	1.18
PPNYKQQPNCPSSN	1.24
PIVKKNVPKPPPLV	2.22
RLVNKLLPPHPFAD	1.45
MYGSKFIPFGPKCQ	2.51
QGSNKYSPSSPLTT	1.50
PKCEKIEPLSPSFK	0.89
VYRRKCIPFAPHQI	1.67
VLTVKMSPSLPDEI	1.45
GTRPKILPKIPTGA	1.43
WSQMRMNPTNPDWI	3.12
DNELKDEPEPPNLI	1.41
KAALKYHPDKPTGD	1.27
NFPQRMMPPPPGLV	1.45
LAYERPLPDLPSTI	1.16
MKAYRANPPLPIDS	2.12
PFIRRYTPLKPITK	1.83
YAVGRIVPDSPTYD	1.52
FSTKKSKPIRPFVF	1.49
IESLREIPYGPDFD	1.34
QHKPKVKPSKPEAK	1.61
KTKKKTVPVKPLQL	1.83
IGKSKSAPPIPEDT	1.77
KKNKKKVPLNPLAK	1.58
MTQPKKNPMEPKTD	1.89
ITDTKLFPGNPNLM	0.36
TSCTKEKPTPPTTT	-0.05
AGHIKTHPNSPTPG	0.95
KIVRRDYPSTPVIV	0.51
ETSLRVLPIAPKIF	1.68
SKRMKALPKGPAAL	1.82
ERDGRGVPLGPRNH	1.40
LWRPRIVPILPYIT	3.27
AKSNKVSPEDPAVQ	1.39
DLDWRKIPFLPKEI	1.42
GYDEKGNPIPPNNW	1.52
KQLKKQGPKRPSSA	1.46
SENGRLTPGLPRSY	1.49
LTSVKIVPQRPISD	1.74
TIEARVHPEPPNKP	1.32
LDSMKNLPLNPSKK	1.62
SLISKRNPCYPYGI	1.73
LYREREEPPLPKRI	1.26
SLKNRDGPITPSTI	1.83
LPKLKSNPSVPHLH	1.31
KGVLKEYPMRPYGS	1.15
SRSKRSKPLPPQLL	1.03
DGKLKHIPRRPYEI	0.54
MPNLKRLPIPPLQD	1.36
LSGTRLVPNCPLLW	1.47
PLRERIIPILPANS	1.20
ALWAKFFPEKPLNI	1.27
NAPERAVPILPPRN	1.92
SKVNKVAPDHPFIQ	0.98
SKYAKLDPSTPVSL	1.59
SVDPKNYPDAPLEG	1.39

SGTALLRPLRPKPP	2.18
KFLDPSRPYKCTVC	1.94
TMCCIYRPLKPRTN	2.08
SQQNFHRPVKVRKK	2.00
FNQALGRPEKIHNK	2.00
HGSFLARPSRKNQG	2.49
LCYCLYRPRKYLYV	2.46
RVPIPPRPVKPDYR	3.78
APVPLLRPHRPSGP	2.85
FSFSLPRPGRRGSR	3.32
LEQLLLRPERPGDT	2.17
ICTKLVRPKRKHGT	1.99
CDKAFCRPDKLRLH	1.74
XSQHFGRPRRADHE	1.85
NFGNLPRPGRGARG	3.13
KLDTPLRPARQQPT	3.14
KVHVPARPYRVLNN	3.24
ATCRFYRPPRCSHC	2.60
EASSPARPSKHSVG	2.33
IIHVISRPLKAPPA	1.86
LVIRLPRPPKVLRL	3.32
NSPTLERPGRKRKW	1.91
APANLERPVRYIRK	2.26
EMPHLARPIKVESP	2.20
RHWRPWRPDKTKKQ	2.75
RDFIISRPTKLKSL	2.26
GNANIQRPCRKSVN	2.22
HHGRIGRPPKYRKI	2.17
PRKYPNRPSKTPVH	1.76
QFPKLDRPTKQMRE	2.12
YAMGFNRPSKIQEN	2.39
AATQPLRPPRGTAA	3.17
VMARLSRPERPDLV	3.07
AVKVFHRPWRRGWH	1.59
ADVDPQRPPRPEVK	2.10
VVSSPPRPVRHGDM	3.31
EIGFPGRPGRPGMN	2.03
PAPSLPRPERKSIV	2.16
SQSFIIRPQKEGER	1.94
TPDLLPRPPRLLLL	1.97
EKIPFERPFRRPNA	2.09
LTKVFFRPGKFAEF	2.30
RYLAICRPLRARVL	2.97
TRYMFSRPFRKHGV	2.73
FVLRPLRPCRRYST	4.27
AEEPLHRPKKELSA	1.85
GSWCPMRPKKEAMK	3.27
HSFLINRPGKVKYM	2.07
SFAYPNRPDRPVLK	2.39
QQQLLQRPPRDAQQ	2.03
QPVNPPRPFKHSER	3.10
FNRYLCRPRRVEMA	2.31
DKADLIRPKRKYEK	1.81
HKAHLIRPRRLTPG	3.25
SSPEPPRPAKRPRR	2.26
QALYFCRPFRENVL	2.35
PLISFPRPVKTTWL	2.23
TKIMFNRPKKKNAI	2.05
TRYSPYRPYRCVHQ	2.96
GYGLLVRPSRVRRA	2.87
PRVDPYRPVRLPMQ	2.24
APPRLARPPRFYEA	2.31
IDPAILRPGRLDQL	1.94
RQQTLPRPMKTLQR	1.83
EVFFPPRPERVEHN	2.08
SEDDFYRPSREQPL	1.97
GAARLGRPGRSCLP	2.11
SAPSPARPARTQGP	3.66
AILTLPRPVRASLP	3.14
EKGELGRPGRKGRP	2.21
ALPPPPRPDRGPPG	3.65
EDFVPFRPRRSPRM	3.42
SAHTPGRPPRVTKD	2.43
SQGPPPRPGKPEGP	3.51
GSGKPSRPSKPIVA	3.13
FNKYLCRPRRVEIA	2.19
SSTNPFRPQKVCSF	3.13
RSRSLHRPAKTSEL	2.27
LLIIISRPARLLEC	1.94
FNKYLSRPKRFEVA	2.04
ILAKISRPKKRAKT	1.77
CVGRPTRPCRGPWR	3.77
DDSVILRPSKSVKL	2.22
GNLDIQRPKRKRKN	1.69
TPRPLARPARPNLS	2.79
LNPDFSRPNRKYTW	1.80
PDPGFLRPQRAALY	2.49
NEPLFVRPPKGSPQ	2.19
PPQVPSRPTRAPPS	4.10
PSPALPRPSRSSPG	3.31
VGKAFIRPFREHHI	2.59
RLFSIVRPRRLLYM	2.85
LKGIFTRPSKLTRK	2.28
GLLLLPRPGRPLDL	1.95
QLQAPDRPSRELLQ	2.58
YPQDLPRPLRSREF	1.98
LAEGINRPRKGHDA	2.30
DEPEPPRPRRGSLR	2.10
LDPALLRPGRLDRK	2.09
RKWYPLRPKKINLD	2.31
KRKKPSRPAKTRKR	2.24
EVEMLPRPPKVTKE	1.80
SGEQLPRPTRGRMR	2.32
RILQFPRPVKLEDL	2.08
YGPNIGRPRKNVIA	2.22
LTVTLQRPTKELHG	2.28
NETGLSRPRRTAMS	2.19
SIVEFFRPPKEVHL	1.99
DDLEIVRPARRRTP	1.83
DTPPPSRPEKKKAD	2.00
ELAHLVRPQKCRPY	1.94
SGMAFPRPKRPAPA	2.80
ANKILNRPKRYNSG	2.30
LSNCLTRPQKLSPV	2.02
MAQSIYRPSKNLDK	2.27
DGRLLYRPNKAGET	1.65
PRPSPARPKKSEES	2.42
SREGPARPYRLKPG	3.08
SCQRPGRPARVALP	4.13
DVLSPLRPSRRHFP	3.42
PATAPARPLRLPSG	3.89
ELYLLFRPFKGYEG	2.25
SVCFIGRPWRVVDG	1.89
SYRRLDRPTRIRFS	3.35
LIRFLPRPIRQQLL	3.57
LDSALLRPGRIDRK	2.07
KAICFLRPTKENVD	1.84
PECEIERPERLTAA	2.45
VPMPIARPARQTSR	3.23
EFGLPGRPGRPGLN	2.15
LREDFVRPLREGIL	2.32
AHLPPPRPQRPAET	2.38
ARRPLARPIKPNEI	2.13
APRYLGRPKKEKTI	1.84
PYPLILRPQRGVDL	2.45
EEEELARPPREQGQ	2.18
IKKDLSRPQKPQLV	1.99
QAGGPQRPGRVVSL	2.74
QASRFQRPGRRMSE	2.65
PAALLYRPVRQYVY	2.63
IRQILSRPERLLFI	2.60
FPTVPTRPEKGYIW	2.63
GRDFLQRPGRQPQA	2.13
KTHPLIRPFKTLTE	2.17
PPQIPSRPVRIPPG	4.57
APALPARPHRGLST	4.58
MTSSLQRPWRVPWL	2.36
LPPDIERPYKAEPL	1.98
PSRPPSRPSRPPSH	4.06
LPKGLGRPGKGHYW	2.02
FVYNFIRPVKGKVS	2.09
VPSLPIRPSRAPSR	4.39
KPSRPPRPSRPPPP	4.66
KNRTFLRPPKVKME	1.75
TREALSRPARTAPL	2.33
LGLRLCRPYRLAAG	2.50
LQSDPPRPHRILQA	2.26
ESRDITRPSRPADL	2.13
ISSQIERPIRIVAL	2.24
KSLRIARPPKQGLN	2.60
GTQELRRPAQLRLQ	1.66
ALLEPRRPPHDYMP	1.74
EVMILRRPSLQRRA	3.34
SSPGPKRPGNTLRK	2.79
EEEPPRRPRARSCE	1.42
IVYSFRRPVWPAVV	2.17
LERRLKRPRTQGVV	1.30
TAKKIKRPKFTVPQ	1.18
QKFSLRRPLQTAMI	1.87
VNRGPKRPKVLQRG	2.15
PAAAPRRPFSWEDV	1.70
VFRVLRRPQEFVNR	2.45
EGEEPKRPPGICER	1.33
WRYDLRRPSFTRTW	2.80
IEPQIRRPRSLSSP	2.56
VKLLIRRPECFGPA	2.16
GKLPPKRPELPPTL	2.31
KIVDPKRPLMPEEC	1.53
PVAVLKRPSQLSRK	1.86
PPKWIRRPVGASFS	2.81
YGHIFRRPKGLFIS	2.04
DKIPFKRPCTYGVP	1.83
QSTNPKRPLSSANQ	1.97
LGDSLRRPQLRLWS	0.78
VFTPLRRPGMLVPK	2.27
QKDVIRRPTFVPQW	1.96
EDAKLRRPMVIEII	1.87
LPENFRRPQTDQAV	1.61
KTFGIRRPLDPLDP	1.62
LAHVLRRPIVVVAD	1.58
LPETFRRPQIDPAV	1.25
NIEKIKRPRSSNAE	1.74
TVNLFKRPLPKSKP	1.78

ETGVRYGPEFPLCT	1.98
GEMVKLTPKNPFYK	2.61
VCTIRSTPSQPIHC	2.39
FHPEKLTPYTPMTG	1.98
NPMQKPVPFVPVLD	2.62
TLKKKKTPKKPIAA	2.73
TKTDKSYPMCPVDA	2.18
SLGERFMPMAPEVY	2.15
ITSIRGSPSLPKNK	2.57
HFPSRSQPSDPKLQ	2.41
DIGSKKLPTGPSKK	2.34
TTRKKKDPNAPKRA	2.44
PSAMKLVPSIPPAT	2.75
LLEERNNPDIPVDK	2.49
QTVIKTHPRIPKSE	2.27
LITDKEYPVRPAYT	2.13
DILKKESPDVPLFD	2.16
NLKTKSHPEVPRLD	2.56
IGSPRHAPSRPDSI	2.29
AVQLKRQPAQPRES	2.38
CWHPKTIPKQPMEI	2.54
QWSKRIMPYIPGPV	2.47
FVFFKSLPDLPKPQ	2.93
RGWQRFLPTGPIAH	2.90
KVLIKTLPVLPEAR	2.37
QTGSKTVPQAPQAP	1.80
VHVLKYAPDEPNSL	2.28
SDNSKSEPLPPVIT	2.12
LDPGKNTPGLPEET	2.19
LSFDRPPPLTPEKN	2.24
LPLSRVQPVQPSQT	2.09
ASFRKTKPPPPLDF	2.23
PKTGKPLPTQPSSS	2.29
QATGRRGPAPPPPP	2.78
TGESRKAPLIPLLK	2.33
LRISKPDPECPSKL	2.39
EIKDRDQPAVPPSP	2.03
GDHPKGPPPPPPPD	4.60
NNSTRPIPAIPMDL	2.39
FEEDKMTPSTPVRS	2.40
YSYLRLLPWWPSLA	2.27
SFIRKRAPTPPAPS	2.94
FLQDRVIPIFPHYP	2.42
AEIVKLTPKNPFYK	2.01
NIFQKQLPKMPKEY	2.44
LTKIRRRPQQPLTD	2.59
IQQNKQGPKIPFYL	1.94
AVGGKWEPGNPIAI	1.89
IESFRERPLEPPRK	1.99
NSVIRGCPGLPDTL	2.18
TEVWRKVPMKPIFE	2.39
DFRGRAYPLSPHFN	2.89
NQIARQIPYQPWYL	2.21
ALSTKGYPHEPPLL	2.14
EEEAKSVPPIPADQ	1.47
SRYLKDHPNLPTPE	1.84
GSYIKRTPKIPEKD	2.31
FASEKGAPFFPNKT	2.55
YKNRKNLPTIPIRL	2.98
RLIKRLKPPAPKEP	2.61
NLSLRYAPNLPPVI	2.89
APVVKSEPSLPRMR	2.38
NDMAKPLPTPPTAE	2.06
VVAVKIIPKKPWNA	2.06
SSVSKLKPSLPNKL	2.62
YVNPRNLPSVPNGL	2.25
AFVPKRKPSLPQLA	3.24
PKSPKAYPKLPDIQ	2.55
GIMIKAKPSSPYTY	2.37
PVRCRVLPATPRQI	3.17
AKDYRFNPAIPLRI	2.48
TVRTRRRPPPPPIP	4.66
SEMHRAAPETPRSI	2.51
ITAEKSAPTSPEVY	1.97
KITKKLGPKLPKAS	2.83
KMAKKLLPYKPHNS	2.85
VTDIKLTPTDPLVG	2.93
KKHDKLHPNYPSVK	2.61
TLDEKNLPRLPTPF	2.31
NPFVRINPNRPEAA	2.44
ALEERFDPKSPTKR	2.04
EFQSKSSPVPPASE	1.90
TSSSRRGPGRPRKD	2.79
DKIFKTDPNEPAND	2.00
KQRIRKIPGIPLMS	2.79
ALLIKTVPGFPLLE	2.16
FSKKKEAPEDPKNL	2.17
TDLFKPLPEPPTEL	1.37
LSKLRLSPENPSNT	2.20
TTKHKAPPPPPPTA	3.23
LMSLKRFPQLPSNT	2.54
LSRSKPLPLTPNSK	2.51
ITRARYMPQDPNIV	2.28
FGTAKILPEEPSNT	2.19
PDLNKRPPREPIEF	2.28
NGMPRRIPDYPDAF	2.35
ILKNRMNPEKPYEL	2.51
VNIGRRSPYLPLQP	2.68
LDVRRIYPKGPLLV	2.36
HKFVKSSPVVPFDD	2.18
YWGKKFEPERPFEF	2.59
KHEPKKSPTTPALA	2.86
LNRQKNYPDAPAIF	1.94
KNILKVCPVLPSIL	2.07
YILPKTDPRSPYYI	2.28
DQLNRLLPNLPEEE	1.68
PFLIKFTPKFPQSI	2.99
GNNSRNIPPPPPPP	4.01
DRLSKIKPFLPQSI	2.35
QLINRFMPHFPSSP	2.71
AKIDKVSPEDPWVL	2.39
VLKIKPLPYKPRGR	2.81
PANPKILPKIPSGA	2.22
STDHRGTPANPGRV	2.41
WRWARDTPRAPGLH	3.32
LIWHKNKPTEPDNK	2.19
WFDLKGLPRQPEPL	2.01
TINSRFYPPIPISE	1.88
KKHDKLYPKYPSVK	2.53
KKNPKKSPMGPQGA	3.00
HLVRKVAPTKPMYC	2.68
WENFKEEPGEPLSR	1.99
LIDFKDYPDVPKVP	2.19
KQAKRAPPSSPYVV	2.47
LNQSKGQPFQPSPT	2.41
HTIPKSGPIVPYVL	2.21
AKINKVSPEDPWVL	2.08
RPKRRAPPPVPKKP	4.83
KNPTKSPPPPPSPS	4.44
NTLLKVVPQEPTAV	2.19
TKFTKPKPKQPVLP	2.65
QPPDRNLPSHPSSN	2.19
LLPTKLIPSIPGGK	2.24
SVSEKTNPSSPSVS	2.10
EELARVTPLTPKEY	2.26
QIVNKPLPPLPVAG	2.40
TSGYKYFPQFPQLK	2.37
LDLSKYGPLPPSRA	2.19
RYPFRMIPELPPKR	3.55
DEPIRYAPGDPIEK	2.03
VKPTKLEPENPQKP	1.96
NSTVRRKPHSPNKK	2.98
PKHIRNEPVKPFRN	2.56
VKATKWPPKLPEIQ	1.76
DYRPKCIPCPPNGI	2.19
RIRDKSKPSSPTAT	2.74
EFPGKKCPRLPHRN	2.57
AERAKKAPSLPEGQ	2.59
SKIPKSIPIIPYVL	2.39
AVLIRADPLNPKVV	2.01
LPFPRKNPKLPENT	2.17
RLLSKYRPSCPIIL	2.53
GTVTKTAPSFPQSS	2.42
GTTNRGPPPLPPRA	4.60
LAGIRLVPVLPILL	2.18
IPYEKRDPTSPAPL	2.06
LGSEKSCPLAPAVA	2.09
QVYEKTIPLFPPEI	2.18
FGSNKNNPVPPELT	2.23
LKDNRLLPSWPKRG	2.71
GKMVKWGPLRPFLS	2.81
EVLRKYIPGEPEFL	2.23
LQQRRYYPIFPGSI	2.45
QRLDRDKPHEPNGY	2.34
VVNEKEVPAEPETQ	2.00
TGKFRYMPFSPAGT	2.74
EFIVKTIPVPPRDF	2.06
REYIKSLPMYPAAP	1.85
LKHTKNKPKNPDTA	2.61
IVNEKRTPALPSNL	2.01
LNTMKKRPAPPSLP	4.35
YYYYKHYPNMPPIN	2.46
SNFKKRDPGLPDFQ	2.21
KTRNKPKPLWPQVK	2.42
AKVNKCPPDHPYIQ	2.22
ETKKKWQPLPPEPL	2.41
VLGPKYDPKCPEEH	2.20
NTINKSNPQGPSDN	1.88
TARNKKSPGGPRQR	2.77
SILRKSSPEEPFMS	2.16
PPQMRSNPQQPPQD	2.43
MYDRKVIPRMPWHD	2.55
TGGGRIVPGVPPGA	2.07
MLFMKGSPSEPKCG	2.40
FLLKKMFPAPPLPT	4.38
ATGKRKLPGWPSAD	2.13
HSTPKPLPPVPALS	2.16
KPAGRPIPPAPTHY	2.28
ELDIKQGPVRPFIV	2.21
LQESKTVPQSPKKP	2.20
PPNYKQQPNCPSSN	2.17
PIVKKNVPKPPPLV	2.19
RLVNKLLPPHPFAD	2.46

GEMVKLTPKNPFYK	1.87
VCTIRSTPSQPIHC	1.84
FHPEKLTPYTPMTG	1.10
NPMQKPVPFVPVLD	2.45
TLKKKKTPKKPIAA	1.49
TKTDKSYPMCPVDA	2.02
SLGERFMPMAPEVY	1.52
ITSIRGSPSLPKNK	2.02
HFPSRSQPSDPKLQ	1.83
DIGSKKLPTGPSKK	1.58
TTRKKKDPNAPKRA	1.90
PSAMKLVPSIPPAT	1.66
LLEERNNPDIPVDK	1.58
QTVIKTHPRIPKSE	0.96
LITDKEYPVRPAYT	1.24
NLKTKSHPEVPRLD	3.03
IGSPRHAPSRPDSI	2.09
AVQLKRQPAQPRES	1.87
CWHPKTIPKQPMEI	1.96
QWSKRIMPYIPGPV	2.15
FVFFKSLPDLPKPQ	2.25
RGWQRFLPTGPIAH	2.75
KVLIKTLPVLPEAR	1.93
QTGSKTVPQAPQAP	1.75
VHVLKYAPDEPNSL	1.54
SDNSKSEPLPPVIT	1.74
LDPGKNTPGLPEET	1.52
LSFDRPPPLTPEKN	1.96
LPLSRVQPVQPSQT	1.29
ASFRKTKPPPPLDF	1.69
PKTGKPLPTQPSSS	1.81
QATGRRGPAPPPPP	1.48
TGESRKAPLIPLLK	2.00
LRISKPDPECPSKL	2.04
EIKDRDQPAVPPSP	1.83
GDHPKGPPPPPPPD	1.74
NNSTRPIPAIPMDL	4.77
FEEDKMTPSTPVRS	1.78
YSYLRLLPWWPSLA	2.06
SFIRKRAPTPPAPS	2.40
FLQDRVIPIFPHYP	1.86
AEIVKLTPKNPFYK	2.00
NIFQKQLPKMPKEY	2.74
LTKIRRRPQQPLTD	2.59
IQQNKQGPKIPFYL	1.37
AVGGKWEPGNPIAI	1.60
IESFRERPLEPPRK	2.21
NSVIRGCPGLPDTL	2.40
TEVWRKVPMKPIFE	2.72
DFRGRAYPLSPHFN	3.26
NQIARQIPYQPWYL	1.52
ALSTKGYPHEPPLL	1.80
EEEAKSVPPIPADQ	1.63
SRYLKDHPNLPTPE	2.02
GSYIKRTPKIPEKD	2.19
FASEKGAPFFPNKT	2.77
YKNRKNLPTIPIRL	3.44
RLIKRLKPPAPKEP	2.12
NLSLRYAPNLPPVI	2.34
APVVKSEPSLPRMR	1.85
NDMAKPLPTPPTAE	1.75
VVAVKIIPKKPWNA	1.68
SSVSKLKPSLPNKL	2.25
YVNPRNLPSVPNGL	2.69
AFVPKRKPSLPQLA	2.47
PKSPKAYPKLPDIQ	0.00
GIMIKAKPSSPYTY	2.39
PVRCRVLPATPRQI	4.02
AKDYRFNPAIPLRI	2.73
TVRTRRRPPPPPIP	2.82
SEMHRAAPETPRSI	2.32
ITAEKSAPTSPEVY	0.54
KITKKLGPKLPKAS	2.27
KMAKKLLPYKPHNS	1.58
VTDIKLTPTDPLVG	1.49
KKHDKLHPNYPSVK	2.62
TLDEKNLPRLPTPF	1.81
NPFVRINPNRPEAA	1.75
ALEERFDPKSPTKR	1.75
EFQSKSSPVPPASE	1.92
TSSSRRGPGRPRKD	2.43
DKIFKTDPNEPAND	1.68
KQRIRKIPGIPLMS	3.58
ALLIKTVPGFPLLE	1.07
FSKKKEAPEDPKNL	1.83
TDLFKPLPEPPTEL	1.27
LSKLRLSPENPSNT	1.57
TTKHKAPPPPPPTA	1.90
LMSLKRFPQLPSNT	3.13
LSRSKPLPLTPNSK	2.60
ITRARYMPQDPNIV	2.04
FGTAKILPEEPSNT	1.37
PDLNKRPPREPIEF	1.44
NGMPRRIPDYPDAF	2.53
ILKNRMNPEKPYEL	2.38
VNIGRRSPYLPLQP	1.88
LDVRRIYPKGPLLV	1.54
HKFVKSSPVVPFDD	2.19
YWGKKFEPERPFEF	2.54
KHEPKKSPTTPALA	1.48
LNRQKNYPDAPAIF	1.80
KNILKVCPVLPSIL	1.77
YILPKTDPRSPYYI	2.25
DQLNRLLPNLPEEE	2.83
PFLIKFTPKFPQSI	2.17
GNNSRNIPPPPPPP	1.70
DRLSKIKPFLPQSI	2.19
QLINRFMPHFPSSP	2.85
AKIDKVSPEDPWVL	1.20
VLKIKPLPYKPRGR	1.40
PANPKILPKIPSGA	1.54
STDHRGTPANPGRV	1.37
WRWARDTPRAPGLH	3.12
LIWHKNKPTEPDNK	2.01
WFDLKGLPRQPEPL	2.44
TINSRFYPPIPISE	2.11
KKHDKLYPKYPSVK	1.79
KKNPKKSPMGPQGA	2.15
HLVRKVAPTKPMYC	2.40
WENFKEEPGEPLSR	1.91
LIDFKDYPDVPKVP	1.45
KQAKRAPPSSPYVV	1.29
LNQSKGQPFQPSPT	2.04
HTIPKSGPIVPYVL	1.45
AKINKVSPEDPWVL	1.59
RPKRRAPPPVPKKP	3.02
KNPTKSPPPPPSPS	1.61
NTLLKVVPQEPTAV	1.80
TKFTKPKPKQPVLP	1.37
QPPDRNLPSHPSSN	1.68
LLPTKLIPSIPGGK	1.24
SVSEKTNPSSPSVS	0.30
EELARVTPLTPKEY	1.88
QIVNKPLPPLPVAG	1.62
TSGYKYFPQFPQLK	1.77
LDLSKYGPLPPSRA	2.32
RYPFRMIPELPPKR	4.17
DEPIRYAPGDPIEK	1.42
VKPTKLEPENPQKP	1.45
NSTVRRKPHSPNKK	2.86
PKHIRNEPVKPFRN	1.70
VKATKWPPKLPEIQ	1.00
DYRPKCIPCPPNGI	1.29
RIRDKSKPSSPTAT	1.75
EFPGKKCPRLPHRN	3.48
AERAKKAPSLPEGQ	0.60
SKIPKSIPIIPYVL	1.86
AVLIRADPLNPKVV	1.11
LPFPRKNPKLPENT	1.60
RLLSKYRPSCPIIL	3.05
GTVTKTAPSFPQSS	1.80
GTTNRGPPPLPPRA	4.94
LAGIRLVPVLPILL	4.11
IPYEKRDPTSPAPL	1.77
LGSEKSCPLAPAVA	1.81
QVYEKTIPLFPPEI	1.36
FGSNKNNPVPPELT	1.43
LKDNRLLPSWPKRG	2.16
GKMVKWGPLRPFLS	3.02
EVLRKYIPGEPEFL	0.84
LQQRRYYPIFPGSI	1.96
QRLDRDKPHEPNGY	1.72
VVNEKEVPAEPETQ	1.11
TGKFRYMPFSPAGT	2.63
EFIVKTIPVPPRDF	1.48
REYIKSLPMYPAAP	2.00
LKHTKNKPKNPDTA	1.60
IVNEKRTPALPSNL	1.71
LNTMKKRPAPPSLP	2.43
YYYYKHYPNMPPIN	2.49
SNFKKRDPGLPDFQ	1.95
KTRNKPKPLWPQVK	1.75
AKVNKCPPDHPYIQ	1.72
ETKKKWQPLPPEPL	1.45
VLGPKYDPKCPEEH	1.10
NTINKSNPQGPSDN	1.97
TARNKKSPGGPRQR	1.92
SILRKSSPEEPFMS	2.21
PPQMRSNPQQPPQD	2.49
MYDRKVIPRMPWHD	3.17
TGGGRIVPGVPPGA	2.49
MLFMKGSPSEPKCG	2.58
FLLKKMFPAPPLPT	1.72
ATGKRKLPGWPSAD	1.91
HSTPKPLPPVPALS	1.97
KPAGRPIPPAPTHY	3.35
ELDIKQGPVRPFIV	1.39
LQESKTVPQSPKKP	1.70
PPNYKQQPNCPSSN	0.00
PIVKKNVPKPPPLV	1.52
RLVNKLLPPHPFAD	2.16
MYGSKFIPFGPKCQ	2.57
QGSNKYSPSSPLTT	1.57

GEMVKLTPKNPFYK	1.78
VCTIRSTPSQPIHC	1.78
FHPEKLTPYTPMTG	1.40
NPMQKPVPFVPVLD	2.28
TLKKKKTPKKPIAA	1.81
TKTDKSYPMCPVDA	1.56
SLGERFMPMAPEVY	0.57
ITSIRGSPSLPKNK	2.04
HFPSRSQPSDPKLQ	2.51
DIGSKKLPTGPSKK	2.78
TTRKKKDPNAPKRA	2.03
PSAMKLVPSIPPAT	1.63
LLEERNNPDIPVDK	1.42
QTVIKTHPRIPKSE	1.83
LITDKEYPVRPAYT	0.89
DILKKESPDVPLFD	1.43
NLKTKSHPEVPRLD	2.02
IGSPRHAPSRPDSI	1.26
AVQLKRQPAQPRES	1.81
CWHPKTIPKQPMEI	2.27
QWSKRIMPYIPGPV	2.36
FVFFKSLPDLPKPQ	4.55
RGWQRFLPTGPIAH	2.68
KVLIKTLPVLPEAR	3.48
QTGSKTVPQAPQAP	1.58
VHVLKYAPDEPNSL	1.29
SDNSKSEPLPPVIT	1.38
LSFDRPPPLTPEKN	1.68
LPLSRVQPVQPSQT	1.52
ASFRKTKPPPPLDF	1.29
PKTGKPLPTQPSSS	4.34
QATGRRGPAPPPPP	1.80
TGESRKAPLIPLLK	1.38
LRISKPDPECPSKL	1.35
EIKDRDQPAVPPSP	1.31
GDHPKGPPPPPPPD	1.45
NNSTRPIPAIPMDL	3.25
FEEDKMTPSTPVRS	1.81
YSYLRLLPWWPSLA	2.14
SFIRKRAPTPPAPS	2.32
FLQDRVIPIFPHYP	0.69
AEIVKLTPKNPFYK	1.59
NIFQKQLPKMPKEY	4.22
LTKIRRRPQQPLTD	2.07
IQQNKQGPKIPFYL	1.30
NSVIRGCPGLPDTL	1.55
TEVWRKVPMKPIFE	1.77
DFRGRAYPLSPHFN	2.99
NQIARQIPYQPWYL	1.68
ALSTKGYPHEPPLL	1.55
EEEAKSVPPIPADQ	1.03
SRYLKDHPNLPTPE	1.97
GSYIKRTPKIPEKD	2.74
FASEKGAPFFPNKT	1.09
YKNRKNLPTIPIRL	4.45
RLIKRLKPPAPKEP	2.22
NLSLRYAPNLPPVI	1.61
APVVKSEPSLPRMR	1.70
NDMAKPLPTPPTAE	4.11
VVAVKIIPKKPWNA	1.83
SSVSKLKPSLPNKL	1.49
YVNPRNLPSVPNGL	3.37
AFVPKRKPSLPQLA	1.90
PKSPKAYPKLPDIQ	2.16
GIMIKAKPSSPYTY	1.79
PVRCRVLPATPRQI	4.45
AKDYRFNPAIPLRI	2.32
TVRTRRRPPPPPIP	1.98
SEMHRAAPETPRSI	1.98
ITAEKSAPTSPEVY	2.07
KITKKLGPKLPKAS	1.67
KMAKKLLPYKPHNS	2.72
VTDIKLTPTDPLVG	1.68
KKHDKLHPNYPSVK	1.86
TLDEKNLPRLPTPF	4.45
NPFVRINPNRPEAA	1.56
ALEERFDPKSPTKR	1.52
EFQSKSSPVPPASE	1.54
TSSSRRGPGRPRKD	2.62
DKIFKTDPNEPAND	1.22
KQRIRKIPGIPLMS	2.25
ALLIKTVPGFPLLE	1.34
FSKKKEAPEDPKNL	1.63
TDLFKPLPEPPTEL	4.17
LSKLRLSPENPSNT	1.74
TTKHKAPPPPPPTA	0.79
LMSLKRFPQLPSNT	2.82
LSRSKPLPLTPNSK	4.75
ITRARYMPQDPNIV	2.19
FGTAKILPEEPSNT	2.26
PDLNKRPPREPIEF	1.84
NGMPRRIPDYPDAF	1.46
ILKNRMNPEKPYEL	2.00
VNIGRRSPYLPLQP	1.97
LDVRRIYPKGPLLV	2.12
HKFVKSSPVVPFDD	1.73
YWGKKFEPERPFEF	2.66
KHEPKKSPTTPALA	1.33
LNRQKNYPDAPAIF	1.76
KNILKVCPVLPSIL	1.76
YILPKTDPRSPYYI	1.29
DQLNRLLPNLPEEE	4.08
PFLIKFTPKFPQSI	1.99
GNNSRNIPPPPPPP	1.45
DRLSKIKPFLPQSI	2.38
QLINRFMPHFPSSP	2.51
AKIDKVSPEDPWVL	1.41
VLKIKPLPYKPRGR	3.53
PANPKILPKIPSGA	4.21
STDHRGTPANPGRV	1.78
WRWARDTPRAPGLH	3.31
LIWHKNKPTEPDNK	1.91
WFDLKGLPRQPEPL	4.29
TINSRFYPPIPISE	1.94
KKHDKLYPKYPSVK	2.06
KKNPKKSPMGPQGA	2.13
HLVRKVAPTKPMYC	2.08
WENFKEEPGEPLSR	1.59
LIDFKDYPDVPKVP	1.56
KQAKRAPPSSPYVV	2.10
LNQSKGQPFQPSPT	1.39
HTIPKSGPIVPYVL	1.62
AKINKVSPEDPWVL	1.37
RPKRRAPPPVPKKP	3.72
NTLLKVVPQEPTAV	1.88
TKFTKPKPKQPVLP	1.76
QPPDRNLPSHPSSN	1.89
LLPTKLIPSIPGGK	1.23
SVSEKTNPSSPSVS	2.05
EELARVTPLTPKEY	1.74
QIVNKPLPPLPVAG	4.78
TSGYKYFPQFPQLK	1.56
LDLSKYGPLPPSRA	1.90
RYPFRMIPELPPKR	3.44
DEPIRYAPGDPIEK	1.74
VKPTKLEPENPQKP	1.38
NSTVRRKPHSPNKK	2.17
PKHIRNEPVKPFRN	1.67
VKATKWPPKLPEIQ	1.41
DYRPKCIPCPPNGI	1.37
RIRDKSKPSSPTAT	2.23
EFPGKKCPRLPHRN	1.85
AERAKKAPSLPEGQ	2.24
SKIPKSIPIIPYVL	1.88
AVLIRADPLNPKVV	1.14
LPFPRKNPKLPENT	2.17
RLLSKYRPSCPIIL	1.62
GTVTKTAPSFPQSS	1.41
GTTNRGPPPLPPRA	2.80
LAGIRLVPVLPILL	1.35
IPYEKRDPTSPAPL	1.47
LGSEKSCPLAPAVA	1.05
QVYEKTIPLFPPEI	1.50
FGSNKNNPVPPELT	1.49
LKDNRLLPSWPKRG	2.54
GKMVKWGPLRPFLS	2.17
EVLRKYIPGEPEFL	1.68
LQQRRYYPIFPGSI	1.59
QRLDRDKPHEPNGY	1.57
VVNEKEVPAEPETQ	1.45
TGKFRYMPFSPAGT	2.48
EFIVKTIPVPPRDF	1.76
REYIKSLPMYPAAP	3.71
LKHTKNKPKNPDTA	1.66
IVNEKRTPALPSNL	1.94
LNTMKKRPAPPSLP	2.57
YYYYKHYPNMPPIN	1.77
SNFKKRDPGLPDFQ	1.57
KTRNKPKPLWPQVK	2.06
AKVNKCPPDHPYIQ	1.52
ETKKKWQPLPPEPL	2.64
VLGPKYDPKCPEEH	1.66
NTINKSNPQGPSDN	1.76
TARNKKSPGGPRQR	2.04
SILRKSSPEEPFMS	1.74
PPQMRSNPQQPPQD	1.75
MYDRKVIPRMPWHD	2.76
TGGGRIVPGVPPGA	1.86
MLFMKGSPSEPKCG	1.78
FLLKKMFPAPPLPT	2.17
ATGKRKLPGWPSAD	1.72
HSTPKPLPPVPALS	4.33
KPAGRPIPPAPTHY	2.06
ELDIKQGPVRPFIV	1.45
LQESKTVPQSPKKP	1.58
PPNYKQQPNCPSSN	1.44
PIVKKNVPKPPPLV	1.70
RLVNKLLPPHPFAD	4.31
MYGSKFIPFGPKCQ	2.16
QGSNKYSPSSPLTT	1.65
PKCEKIEPLSPSFK	1.93
AGLYKLSPLYPFDV	1.42
VYRRKCIPFAPHQI	2.13

LPLSRVQPVQPSQT	2.21
ASFRKTKPPPPLDF	2.91
PKTGKPLPTQPSSS	1.95
QATGRRGPAPPPPP	1.70
TGESRKAPLIPLLK	2.21
LRISKPDPECPSKL	2.31
EIKDRDQPAVPPSP	0.94
NNSTRPIPAIPMDL	4.60
FEEDKMTPSTPVRS	4.04
YSYLRLLPWWPSLA	1.97
SFIRKRAPTPPAPS	3.18
FLQDRVIPIFPHYP	2.24
AEIVKLTPKNPFYK	0.30
NIFQKQLPKMPKEY	2.59
LTKIRRRPQQPLTD	2.97
IQQNKQGPKIPFYL	1.28
AVGGKWEPGNPIAI	1.24
IESFRERPLEPPRK	3.43
NSVIRGCPGLPDTL	2.38
DFRGRAYPLSPHFN	3.21
NQIARQIPYQPWYL	2.16
ALSTKGYPHEPPLL	1.43
SRYLKDHPNLPTPE	2.00
GSYIKRTPKIPEKD	2.64
FASEKGAPFFPNKT	2.42
YKNRKNLPTIPIRL	3.47
RLIKRLKPPAPKEP	2.47
NLSLRYAPNLPPVI	2.19
APVVKSEPSLPRMR	2.71
NDMAKPLPTPPTAE	2.85
VVAVKIIPKKPWNA	1.62
SSVSKLKPSLPNKL	3.14
YVNPRNLPSVPNGL	2.98
AFVPKRKPSLPQLA	2.86
PKSPKAYPKLPDIQ	1.92
PVRCRVLPATPRQI	4.20
AKDYRFNPAIPLRI	3.15
TVRTRRRPPPPPIP	2.95
SEMHRAAPETPRSI	2.96
ITAEKSAPTSPEVY	1.81
KITKKLGPKLPKAS	2.69
KMAKKLLPYKPHNS	2.26
VTDIKLTPTDPLVG	1.66
KKHDKLHPNYPSVK	2.21
TLDEKNLPRLPTPF	1.97
NPFVRINPNRPEAA	2.54
ALEERFDPKSPTKR	0.61
EFQSKSSPVPPASE	0.86
TSSSRRGPGRPRKD	2.99
KQRIRKIPGIPLMS	4.09
ALLIKTVPGFPLLE	1.60
FSKKKEAPEDPKNL	1.13
TDLFKPLPEPPTEL	2.11
LSKLRLSPENPSNT	1.43
TTKHKAPPPPPPTA	2.15
LMSLKRFPQLPSNT	3.38
LSRSKPLPLTPNSK	2.92
ITRARYMPQDPNIV	2.69
FGTAKILPEEPSNT	-1.00
PDLNKRPPREPIEF	2.43
NGMPRRIPDYPDAF	3.12
ILKNRMNPEKPYEL	2.54
VNIGRRSPYLPLQP	2.45
HKFVKSSPVVPFDD	2.09
YWGKKFEPERPFEF	3.06
KHEPKKSPTTPALA	1.29
LNRQKNYPDAPAIF	1.59
YILPKTDPRSPYYI	2.05
DQLNRLLPNLPEEE	4.09
PFLIKFTPKFPQSI	2.06
GNNSRNIPPPPPPP	1.79
DRLSKIKPFLPQSI	2.13
QLINRFMPHFPSSP	3.44
AKIDKVSPEDPWVL	1.53
VLKIKPLPYKPRGR	2.63
PANPKILPKIPSGA	1.97
STDHRGTPANPGRV	0.79
WRWARDTPRAPGLH	3.89
LIWHKNKPTEPDNK	2.77
WFDLKGLPRQPEPL	3.69
TINSRFYPPIPISE	2.48
KKHDKLYPKYPSVK	2.27
HLVRKVAPTKPMYC	2.98
WENFKEEPGEPLSR	3.45
LIDFKDYPDVPKVP	1.10
KQAKRAPPSSPYVV	2.18
LNQSKGQPFQPSPT	1.20
HTIPKSGPIVPYVL	1.68
AKINKVSPEDPWVL	1.17
RPKRRAPPPVPKKP	3.80
KNPTKSPPPPPSPS	1.87
NTLLKVVPQEPTAV	2.06
TKFTKPKPKQPVLP	-0.70
QPPDRNLPSHPSSN	1.69
LLPTKLIPSIPGGK	2.19
EELARVTPLTPKEY	1.40
QIVNKPLPPLPVAG	3.23
TSGYKYFPQFPQLK	2.13
LDLSKYGPLPPSRA	2.56
RYPFRMIPELPPKR	4.27
DEPIRYAPGDPIEK	-0.30
VKPTKLEPENPQKP	1.23
NSTVRRKPHSPNKK	2.91
PKHIRNEPVKPFRN	2.97
VKATKWPPKLPEIQ	1.64
DYRPKCIPCPPNGI	1.59
RIRDKSKPSSPTAT	2.77
EFPGKKCPRLPHRN	3.92
AERAKKAPSLPEGQ	1.65
SKIPKSIPIIPYVL	1.63
AVLIRADPLNPKVV	1.54
RLLSKYRPSCPIIL	2.72
GTVTKTAPSFPQSS	1.30
GTTNRGPPPLPPRA	4.64
LAGIRLVPVLPILL	3.39
IPYEKRDPTSPAPL	1.71
LGSEKSCPLAPAVA	1.33
QVYEKTIPLFPPEI	0.97
FGSNKNNPVPPELT	1.85
LKDNRLLPSWPKRG	3.56
GKMVKWGPLRPFLS	3.30
LQQRRYYPIFPGSI	2.68
QRLDRDKPHEPNGY	2.02
VVNEKEVPAEPETQ	1.52
TGKFRYMPFSPAGT	2.78
EFIVKTIPVPPRDF	2.31
REYIKSLPMYPAAP	2.01
LKHTKNKPKNPDTA	1.68
IVNEKRTPALPSNL	1.87
LNTMKKRPAPPSLP	3.16
YYYYKHYPNMPPIN	2.59
SNFKKRDPGLPDFQ	2.33
KTRNKPKPLWPQVK	2.25
AKVNKCPPDHPYIQ	1.60
ETKKKWQPLPPEPL	1.48
NTINKSNPQGPSDN	2.21
TARNKKSPGGPRQR	2.63
SILRKSSPEEPFMS	2.52
MYDRKVIPRMPWHD	3.20
TGGGRIVPGVPPGA	2.12
MLFMKGSPSEPKCG	3.04
FLLKKMFPAPPLPT	2.12
ATGKRKLPGWPSAD	3.56
HSTPKPLPPVPALS	2.67
KPAGRPIPPAPTHY	3.65
ELDIKQGPVRPFIV	1.61
LQESKTVPQSPKKP	1.29
PPNYKQQPNCPSSN	2.27
PIVKKNVPKPPPLV	1.81
RLVNKLLPPHPFAD	2.27
MYGSKFIPFGPKCQ	2.95
QGSNKYSPSSPLTT	1.87
VYRRKCIPFAPHQI	2.26
VLTVKMSPSLPDEI	2.65
GTRPKILPKIPTGA	2.64
WSQMRMNPTNPDWI	3.51
KAALKYHPDKPTGD	1.52
NFPQRMMPPPPGLV	2.83
LAYERPLPDLPSTI	3.65
MKAYRANPPLPIDS	2.97
PFIRRYTPLKPITK	2.80
YAVGRIVPDSPTYD	2.20
FSTKKSKPIRPFVF	2.94
IESLREIPYGPDFD	0.41
QHKPKVKPSKPEAK	1.11
KTKKKTVPVKPLQL	2.25
TELLKTLPFYPTPS	1.36
IGKSKSAPPIPEDT	1.63
KKNKKKVPLNPLAK	2.73
MTQPKKNPMEPKTD	2.61
ITDTKLFPGNPNLM	1.46
KIVRRDYPSTPVIV	2.43
ETSLRVLPIAPKIF	2.60
SKRMKALPKGPAAL	3.19
ERDGRGVPLGPRNH	2.81
AKSNKVSPEDPAVQ	2.15
DLDWRKIPFLPKEI	2.90
KQLKKQGPKRPSSA	2.88
SENGRLTPGLPRSY	2.38
LTSVKIVPQRPISD	3.19
TIEARVHPEPPNKP	1.65
LDSMKNLPLNPSKK	2.54
SLISKRNPCYPYGI	1.90
LYREREEPPLPKRI	4.07
SLKNRDGPITPSTI	2.21
LPKLKSNPSVPHLH	1.85
KGVLKEYPMRPYGS	3.06
SRSKRSKPLPPQLL	2.61
DGKLKHIPRRPYEI	2.50
MPNLKRLPIPPLQD	2.69
LSGTRLVPNCPLLW	3.15
ALWAKFFPEKPLNI	2.61
NAPERAVPILPPRN	4.39

VCTIRSTPSQPIHC	1.57
FHPEKLTPYTPMTG	1.62
NPMQKPVPFVPVLD	2.48
TLKKKKTPKKPIAA	1.50
TKTDKSYPMCPVDA	1.19
SLGERFMPMAPEVY	0.86
ITSIRGSPSLPKNK	1.65
HFPSRSQPSDPKLQ	1.51
DIGSKKLPTGPSKK	1.09
TTRKKKDPNAPKRA	1.73
PSAMKLVPSIPPAT	1.80
LLEERNNPDIPVDK	1.29
QTVIKTHPRIPKSE	1.80
LITDKEYPVRPAYT	1.29
DILKKESPDVPLFD	1.26
NLKTKSHPEVPRLD	3.44
IGSPRHAPSRPDSI	1.76
AVQLKRQPAQPRES	1.62
CWHPKTIPKQPMEI	1.90
QWSKRIMPYIPGPV	2.04
FVFFKSLPDLPKPQ	1.90
RGWQRFLPTGPIAH	2.78
QTGSKTVPQAPQAP	1.46
VHVLKYAPDEPNSL	1.41
SDNSKSEPLPPVIT	1.51
LDPGKNTPGLPEET	1.18
LSFDRPPPLTPEKN	0.87
LPLSRVQPVQPSQT	1.32
ASFRKTKPPPPLDF	2.48
PKTGKPLPTQPSSS	1.38
QATGRRGPAPPPPP	1.57
TGESRKAPLIPLLK	0.76
LRISKPDPECPSKL	1.87
GDHPKGPPPPPPPD	0.56
NNSTRPIPAIPMDL	4.64
FEEDKMTPSTPVRS	3.59
YSYLRLLPWWPSLA	2.00
SFIRKRAPTPPAPS	2.00
FLQDRVIPIFPHYP	1.42
AEIVKLTPKNPFYK	0.92
NIFQKQLPKMPKEY	3.70
LTKIRRRPQQPLTD	1.86
IQQNKQGPKIPFYL	0.70
AVGGKWEPGNPIAI	1.29
IESFRERPLEPPRK	1.94
NSVIRGCPGLPDTL	2.47
TEVWRKVPMKPIFE	2.90
DFRGRAYPLSPHFN	2.41
NQIARQIPYQPWYL	1.63
ALSTKGYPHEPPLL	1.18
EEEAKSVPPIPADQ	1.75
SRYLKDHPNLPTPE	1.37
GSYIKRTPKIPEKD	1.72
FASEKGAPFFPNKT	1.61
YKNRKNLPTIPIRL	2.73
RLIKRLKPPAPKEP	1.82
NLSLRYAPNLPPVI	2.13
APVVKSEPSLPRMR	1.80
NDMAKPLPTPPTAE	2.31
VVAVKIIPKKPWNA	1.17
SSVSKLKPSLPNKL	1.59
YVNPRNLPSVPNGL	2.88
AFVPKRKPSLPQLA	1.67
PKSPKAYPKLPDIQ	1.38
GIMIKAKPSSPYTY	2.30
PVRCRVLPATPRQI	3.14
AKDYRFNPAIPLRI	2.75
TVRTRRRPPPPPIP	1.96
SEMHRAAPETPRSI	2.14
ITAEKSAPTSPEVY	1.85
KITKKLGPKLPKAS	1.64
KMAKKLLPYKPHNS	1.57
VTDIKLTPTDPLVG	1.01
KKHDKLHPNYPSVK	1.10
TLDEKNLPRLPTPF	1.81
NPFVRINPNRPEAA	1.87
ALEERFDPKSPTKR	1.80
EFQSKSSPVPPASE	1.70
TSSSRRGPGRPRKD	1.81
DKIFKTDPNEPAND	1.15
KQRIRKIPGIPLMS	3.05
ALLIKTVPGFPLLE	0.88
FSKKKEAPEDPKNL	1.34
TDLFKPLPEPPTEL	1.50
LSKLRLSPENPSNT	0.90
TTKHKAPPPPPPTA	2.27
LMSLKRFPQLPSNT	2.74
LSRSKPLPLTPNSK	2.02
ITRARYMPQDPNIV	1.93
FGTAKILPEEPSNT	1.48
PDLNKRPPREPIEF	0.66
NGMPRRIPDYPDAF	2.93
ILKNRMNPEKPYEL	1.74
VNIGRRSPYLPLQP	1.29
LDVRRIYPKGPLLV	1.51
HKFVKSSPVVPFDD	1.61
YWGKKFEPERPFEF	2.51
KHEPKKSPTTPALA	1.43
LNRQKNYPDAPAIF	1.29
KNILKVCPVLPSIL	1.00
YILPKTDPRSPYYI	1.44
DQLNRLLPNLPEEE	4.37
PFLIKFTPKFPQSI	1.44
GNNSRNIPPPPPPP	1.20
DRLSKIKPFLPQSI	1.57
QLINRFMPHFPSSP	2.50
AKIDKVSPEDPWVL	1.12
VLKIKPLPYKPRGR	1.44
PANPKILPKIPSGA	0.71
STDHRGTPANPGRV	1.48
WRWARDTPRAPGLH	3.30
LIWHKNKPTEPDNK	1.98
WFDLKGLPRQPEPL	2.30
TINSRFYPPIPISE	1.91
KKHDKLYPKYPSVK	1.58
KKNPKKSPMGPQGA	1.85
HLVRKVAPTKPMYC	1.85
WENFKEEPGEPLSR	2.15
LIDFKDYPDVPKVP	0.30
KQAKRAPPSSPYVV	1.32
LNQSKGQPFQPSPT	1.42
HTIPKSGPIVPYVL	1.27
AKINKVSPEDPWVL	1.09
RPKRRAPPPVPKKP	2.06
KNPTKSPPPPPSPS	1.05
NTLLKVVPQEPTAV	2.49
TKFTKPKPKQPVLP	0.45
QPPDRNLPSHPSSN	1.29
LLPTKLIPSIPGGK	1.16
SVSEKTNPSSPSVS	1.10
EELARVTPLTPKEY	1.07
QIVNKPLPPLPVAG	1.46
TSGYKYFPQFPQLK	1.31
LDLSKYGPLPPSRA	2.41
RYPFRMIPELPPKR	3.87
DEPIRYAPGDPIEK	0.96
VKPTKLEPENPQKP	1.35
NSTVRRKPHSPNKK	3.26
PKHIRNEPVKPFRN	1.78
VKATKWPPKLPEIQ	1.70
DYRPKCIPCPPNGI	1.56
RIRDKSKPSSPTAT	1.67
EFPGKKCPRLPHRN	3.86
SKIPKSIPIIPYVL	1.52
AVLIRADPLNPKVV	0.98
LPFPRKNPKLPENT	1.65
RLLSKYRPSCPIIL	2.39
GTVTKTAPSFPQSS	1.66
GTTNRGPPPLPPRA	4.84
LAGIRLVPVLPILL	2.69
IPYEKRDPTSPAPL	1.43
LGSEKSCPLAPAVA	1.43
QVYEKTIPLFPPEI	0.73
FGSNKNNPVPPELT	1.57
LKDNRLLPSWPKRG	2.69
GKMVKWGPLRPFLS	2.99
EVLRKYIPGEPEFL	1.02
LQQRRYYPIFPGSI	1.65
QRLDRDKPHEPNGY	1.29
VVNEKEVPAEPETQ	1.10
TGKFRYMPFSPAGT	2.17
EFIVKTIPVPPRDF	1.65
REYIKSLPMYPAAP	1.68
LKHTKNKPKNPDTA	1.23
IVNEKRTPALPSNL	1.06
LNTMKKRPAPPSLP	2.31
YYYYKHYPNMPPIN	2.40
SNFKKRDPGLPDFQ	1.69
KTRNKPKPLWPQVK	1.54
AKVNKCPPDHPYIQ	1.27
ETKKKWQPLPPEPL	0.40
VLGPKYDPKCPEEH	1.36
NTINKSNPQGPSDN	1.67
TARNKKSPGGPRQR	1.46
SILRKSSPEEPFMS	1.34
PPQMRSNPQQPPQD	2.66
MYDRKVIPRMPWHD	2.87
TGGGRIVPGVPPGA	1.70
MLFMKGSPSEPKCG	2.42
FLLKKMFPAPPLPT	1.61
ATGKRKLPGWPSAD	1.96
HSTPKPLPPVPALS	1.48
KPAGRPIPPAPTHY	3.46
ELDIKQGPVRPFIV	1.25
LQESKTVPQSPKKP	0.96
PPNYKQQPNCPSSN	1.48
PIVKKNVPKPPPLV	1.43
RLVNKLLPPHPFAD	1.39
MYGSKFIPFGPKCQ	2.07
QGSNKYSPSSPLTT	2.16
PKCEKIEPLSPSFK	-1.00
AGLYKLSPLYPFDV	1.25
VYRRKCIPFAPHQI	1.85

AGFKRPVVLF	1.39
QGLRRPPSVL	1.81
KRFKRPQFSP	1.53
DPLKRPTFKQ	1.60
ISLRRPRLAS	1.75
FEPRRPPPLR	1.51
KTIKRPFGVK	1.35
SSFRRPRRSS	3.37
IQFKRPHTVA	1.55
REPKRPEIIF	1.49
IQPRRPRDPT	1.89
LQPKRPKTIK	1.37
GSPRRPCGRK	1.54
CAPRRPRRPR	3.69
DVIKRPLVKN	1.42
AGPKRPVFIL	0.98
GMFRRPSEGQ	1.64
RLPRRPARSG	3.95
PQLRRPFELL	1.53
PDFKRPACFG	1.49
APLRRPLAVL	2.50
RNPRRPPPSS	1.90
ALFKRPKANG	1.28
QPPRRPKSSN	2.15
PRIKRPRLQV	1.56
QELRRPKYTI	1.51
PGPPRPPKEP	1.87
PEPKRPGVSE	1.42
DPLKRPVVYV	1.33
NIFKRPLGSR	1.48
PRPKRPAPAQ	2.16
GSLRRPLSWK	1.64
PDLRRPPTPA	1.60
PGFRRPARPE	3.45
IFLKRPVVEG	1.51
IPFRRPSTYG	1.63
NAPKRPPSAF	1.60
SGLKRPSSSA	1.50
SAPRRPTATG	1.67
PAPKRPRGGG	1.59
GFLKRPVVVT	1.58
KKFRRPDIQY	1.39
TPPRRPHRCS	3.82
HWLRRPVYSL	1.70
QVPRRPHHTP	1.63
LPLRRPSYTL	1.71
SGIKRPMASE	1.25
DHIRRPMNAF	1.48
GDPRRPYPTD	1.08
NLIKRPDDEQ	1.21
GSPRRPTEAE	1.37
RRPKRPRTIL	2.26
LRLKRPKPER	1.63
RRPRRPGPSD	2.77
RRLKRPETPT	1.46
RLPRRPTVES	3.11
TYLRRPEASS	1.33
ASPRRPRSFK	1.76
TYPRRPGSSP	1.74
RDLRRPFSAP	1.73
PNPKRPGLDR	1.64
LEPRRPRYRV	1.55
VAIRRPPDLG	1.37
EAIKRPSPDE	1.63
PAPRRPTTDQ	1.56
RRPRRPRRPG	3.38
RAPRRPRSPP	1.97
KQPRRPRRWV	3.34
SGLKRPSSSA	1.51
PVPRRPSTWS	3.38
QEPKRPSQLP	1.72
KKFKRPPQRL	1.76
KALRRPKCAE	1.41
LRLRRPRVSS	1.83
KSPRRPSPTA	1.62
PSPKRPVKLL	1.87
FVIKRPELLT	1.45
SYLRRPAVVY	1.55
LCPRRPWPSL	2.00
GVLRRPRPTP	1.58
KELRRPPPRS	1.53
PRPRRPREAP	2.61
GPPKRPGGSS	1.70
RTPRRPRSAR	1.77
TQPKRPMSIP	1.37
WGLRRPIRLQ	2.97
IKPKRPFVES	2.08
ITLRRPGCEL	1.20
VSPRRPQATE	2.17
WDIRRPDRCE	3.36
GRLRRPVNVP	1.53
LLLKRPWDPA	1.48
FHIKRPAPRP	1.26
SAPRRPAKGA	2.25
SFPRRPLAVE	1.72
SVLKRPVTSE	1.58
SLLRRPGEKT	1.47
YQLRRPEDRP	1.16
PEFKRPRDIA	1.48
FLPPRPPKVL	3.06
AWPRRPRRDY	3.19
EEPRRPLRSD	3.28
IRLKRPLKME	1.34
KVIRRPRRGR	2.07
IEFKRPGKAA	2.09
KGIRRPWKGV	1.80
KIFRRPRKLF	1.94
EAPRRPSRKG	4.10
EKPKRPTRRR	4.36
RDPRRPARGX	3.14
GVLRRPRRRP	3.39
TSPRRPLRPT	3.55
YLPKRPAKDD	1.05
WGLRRPIRLQ	2.69
RSPPRPPRER	3.88
SVLKRPERSQ	1.95
PLPKRPRRGA	3.74
DSPRRPSRCT	4.05
QWPRRPGRRW	2.91
VYPKRPEKPV	1.63
QPLRRPFRPN	3.23
QQPKRPQKHI	2.01
GFFKRPLKKK	2.17
PGPRRPPWEG	1.40
PPPPRPPKQQ	3.00
APPRRPPQSA	3.52
QSPRRPPHPL	2.53
QSPKRPPEDF	1.62
KPPKRPPIEA	1.76
PPPRRPPQAP	2.87
LLPPRPPQGL	2.03
QPPSRPPRPP	3.83
NFPNKPPLTF	1.66
VVPLRPPPPP	2.70
SRPTRPPEPR	3.04
VPPPRPPPPE	1.66
GPPKRPPRKL	4.23
PKPERPPSSS	1.82
APPQRPPPPS	3.30
GSPFRPPSHS	1.71
LGPAKPPAQQ	1.72
QRPTRPPRQD	3.83
PSPSKPPIQQ	1.74
PPPQKPPPPN	1.65
GLPSRPPSQL	1.63
TVPARPPATH	3.91
RPPSRPPSRP	4.37
VPPARPPPLT	2.91
ALPGKPPFLT	1.76
KPPVKPPTEP	1.58
PVPSKPPVTP	1.74
EPPARPPPPP	1.58
TSPLRPPEGV	2.37
IRPPRPPKVL	4.09
QKPERPPLPP	3.42
RSPGRPPKSA	2.19
PGPGRPPPPP	2.01
GSPARPPFSF	1.52
PKPPKPPKPP	3.12
PSPARPPGPS	1.49
GPPPKPPVSS	1.91
FPPPRPPPPN	2.16
FKPSRPPRPS	4.26
CPPPRPPTSP	1.92
CGPYKPPGIL	1.60
APPARPPRPP	3.62
PPPTKPPETE	1.78
RPPSKPPPVE	1.85
QLPPRPPQSS	1.82
LGPSRPPTGE	1.58
TLPPRPPGQD	1.72
LVPLRPPSPA	3.21
QPPARPPPPV	3.18
FSPPRPPYPS	1.26
PKPPRPPTGR	2.76
NRPKKPPPPS	1.73
TVPLKPPPPL	1.59
PKPPRPPKTL	3.11
PGPAKPPTQQ	1.80
NVPMKPPDEA	3.36
PKPPKPPDKP	1.77
RDITRPSRPA	2.73
EFLERPSRDH	2.52
VLLVRPSRLS	3.90
NRFDRPDRNV	3.56
AKIARPKKRA	2.23
LDPSRPYKCT	1.33
CCIYRPLKPR	1.99
YCLYRPRKYL	2.14
KGPTRPPRPS	4.03
FSLPRPGRRG	3.04
QHFGRPRRAD	2.66
DTPLRPARQQ	3.34

PTSVPRRPGQRRPP	1.51
KFWDLRRPYEPINS	1.65
MKTSPRRPLILKRR	2.18
VHLKLRRPVTVPAG	1.45
DSGQPRRPGQCPLS	1.31
SSSAPRRPSSLQSL	1.57
DPNAPKRPPLAFFL	1.51
TSPTFRRPKTLRLR	1.75
RADIPKRPSYVAPL	1.68
IRFKLKRPLEDGGR	1.58
DESCPRRPDYLKGL	1.38
LSQPPRRPWGNLTA	1.78
FVTELRRPDAVRTK	1.58
KPQTIKRPLNPLAS	0.95
GKRPIKRPAYWRTE	0.57
SHGSPRRPGPEPGA	1.65
PQEHPKRPVLGLEC	1.31
ASLFPKRPALEPVR	1.61
ASNQPKRPPAAPEN	1.58
CGRHFRRPDPCREP	1.37
LRATLKRPEVIATC	1.58
AFVNFRRPEDARAA	1.24
TLLEPRRPRSESVT	1.47
GTPKPKRPAILTYH	1.52
KDEKPKRPEDESGR	1.48
LASTPKRPSTSQTV	1.51
RGDDLRRPATMKGK	1.48
LAQPLRRPSSVGEL	1.57
MNMTPRRPGPTRKH	1.88
HILLFRRPLPKKPK	1.54
REASFKRPVVILGP	1.68
EQRPLKRPRLGPPD	1.49
SVVGIRRPFQIPPG	1.53
LDSILRRPYVTCQP	1.03
PTGQPRRPRNLAAA	1.82
ITKSLRRPETSYRQ	1.59
FNFDIRRPSLEHRQ	2.06
QPQSLRRPLWLSHS	1.82
ISFHPKRPMHILLH	1.75
TEEELRRPEMKNFF	1.53
EGLAFRRPAEYDPK	1.46
DPSPPRRPQHNSSG	1.65
SEPHIKRPMNAFMV	1.51
GIPSPRRPMSNLWL	1.88
RPVAPRRPRDAVGQ	3.56
VETSLRRPQDKDAK	1.43
PGHSPRRPRPLGLS	1.65
TYVTPRRPFEKSRL	2.39
SSDWLRRPLGEDNQ	1.64
DPNAPKRPPSGFFL	1.30
RNPVLRRPPILEDY	1.46
DANRLRRPGGSYPA	1.39
QPLEPKRPPPPRPV	1.46
LLNTLKRPKRPPLK	2.65
SFSHLKRPQQGAGN	1.42
SGKGLRRPHCTVEE	1.65
WLTCLKRPLVVIHE	1.42
YKVNLKRPCPFWND	1.68
RHLSPRRPVSPGKD	1.78
FERPFRRPNANFTS	3.60
PSGHIKRPMNAFMV	1.68
EDEEPKRPLPRKQS	1.55
QQPLPRRPGHDDGQ	1.57
NSMGIKRPAPQPAS	1.39
SGGRLRRPAHVPLD	1.32
TNPELRRPRSEPSN	1.54
ASAPPRRPRPPPPS	2.36
CKKGFRRPSEKNQH	1.52
NYKNIKRPPGDIMF	1.28
ALLDPRRPEIPTEQ	1.42
PEVYLKRPAHSDDW	1.52
DPEVLKRPEYFGKF	1.64
EHPRLRRPMPWLLL	1.93
ARPELRRPGVQGIP	1.41
QLVILRRPERPMED	1.50
KSLKLKRPLYAHLA	1.50
TRKGPKRPQQPNPP	1.67
GWATPRRPAGLLML	1.68
PAHPFRRPKLSGRS	1.69
MAASLKRPDSGATG	1.43
GSEKPKRPVSAMFI	1.59
KSRTLKRPTVLEPI	1.47
VKLKIKRPYGRKSQ	1.48
LRGKFKRPPLRRVR	1.55
NSSHIKRPMNAFMV	1.57
GQKQPKRPRSESSC	1.71
PAPGLRRPLSPPAA	1.76
EPPAPRRPASLAQR	1.57
MKVHIKRPEMTSSS	1.44
NGYFFKRPYPFVLF	1.81
PLRHFKRPAYASSS	1.46
SQEILRRPIVACEL	1.32
FSESLKRPWEDDPP	1.77
NINGIRRPLQGVAN	1.24
SLRVPKRPIINTPV	1.73
DNGGFKRPLAPSGR	1.50
RGAQLRRPRHSHPT	1.73
LKVQLKRPKDANRP	1.46
LCNILRRPIIVISD	1.41
VESILKRPSSAITR	1.43
TPGEFRRPRLASFG	1.65
QVYVLKRPHVDEFL	1.29
IKTPPKRPGGRRRG	1.84
VRLLIRRPECFGPA	1.19
RLAALKRPDDVPLS	1.58
GSPAPKRPPDPFPG	1.71
PPPLPRRPSTPRQG	3.29
SWERPRRPNGDIVG	3.57
IFMYLRRPTEESVE	2.05
PDPAIRRPAEKFLE	1.56
DTLNIRRPLVKMVY	1.39
CCSGPKRPQDKVAV	1.70
GQKLPRRPQPGPGE	1.17
THLLIKRPPFFHYR	1.53
PDETLRRPGGPQYG	1.39
REDRFRRPRDEGGW	1.66
CFHSPRRPYASPLL	1.85
CRNVLRRPFQAQCG	1.50
SMFFIRRPTLDINW	1.77
SAGRPKRPHARRAL	1.39
HLAFLKRPELGLTL	1.56
LLGPFRRPTTQLRA	2.53
VSMQLRRPSDRELS	1.65
VARSPKRPAWGSRR	1.67
EREALKRPMGPVHY	1.06
SCNFPRRPDSGDVT	1.48
ALQSPKRPRSPGSN	1.50
CEVPLKRPRLDKNS	1.66
RRIHIKRPDDRAHL	1.32
EGISLRRPNCFGIA	1.43
PEDAPKRPKEAAPD	1.29
PGAGLRRPTYTPPS	1.50
ARKQPRRPHFLLEP	1.48
GQVILRRPLDYEKN	1.30
IIRQIRRPKNVVLR	1.20
TPKPPKRPRLQRPA	2.58
LANELRRPPLCYHN	1.54
PGFSLKRPRGLSRS	1.60
KTTPLRRPTETNPV	1.50
PAAPPKRPAVASAR	1.81
EVLELRRPMDSRLE	1.64
PPKWIRRPTGVSFA	1.63
SSKPIKRPVLDPFI	1.55
RVKYIKRPGAVLEA	1.43
FSSFLKRPKSQVSE	1.57
QSLKIRRPHDYQPL	1.57
TSLSLRRPPGPASQ	1.53
GQCAPRRPCAGDGA	1.37
LVIRLRRPPLRYPR	2.59
ASAPPRRPRPPPQS	2.96
SFTALRRPSLRREA	1.81
RDAFLKRPISNKYM	1.54
GTVELRRPGQSHAA	1.46
TYQHLRRPRGLSSL	1.50
PVLDLKRPFLSRES	1.67
PLLPPKRPLSSSHE	2.83
RGQEFRRPCTLSEL	1.59
PVLDLRRPPSDLYL	1.55
SGSKLKRPTPTFHS	1.62
ESPPPKRPRIAYLS	1.59
ARLYLRRPAADGAE	1.63
PRPRPRRPRQLTRA	1.86
ALTLPRRPFLHCST	3.18
LCKTLRRPRCLLET	1.51
IMFDLRRPPQNHTY	1.80
SPVHLRRPQSPGAS	1.50
LSGGLKRPSTRTPN	1.35
VRSRFKRPKPNLAR	1.57
KLGTLRRPEGPAEP	1.39
FLCIPRRPNWNQNT	2.21
SPQAPRRPLPQVAT	2.77
GPAPLRRPDSSDDR	1.67
MFQALKRPDDTAFQ	1.58
GHGEPRRPPCDPLA	1.31
PHHELRRPVLQRQT	1.48
SGDQLKRPFGAFRS	1.31
KPVSPRRPWSPSKE	2.35
VNLKLRRPSFRDDI	1.78
PMAPIRRPISAESA	3.00
EGVPFRRPSTFGIP	1.49
KQYMLRRPALEDYV	1.90
MSLLLRRPPGREAY	1.20
GSELPRRPLPPAAQ	1.60
EGSMLRRPRTSSQH	2.06
EERPLRRPRPVQRT	2.92
IKFVIKRPELLTHS	1.46
HLWQPKRPAVPKKA	1.24
GPLDLRRPMRTVPG	3.03
ARNQLRRPQYRPQY	1.11
GSSALKRPFEDGLG	1.44
KGRVLKRPPNSVVT	1.52
GARSPKRPRLQLPG	1.27
TQPVLKRPRHVHCH	1.67

DLDWRKIPFLPKEI	3.46
GYDEKGNPIPPNNW	1.85
KQLKKQGPKRPSSA	2.88
SENGRLTPGLPRSY	1.81
LTSVKIVPQRPISD	2.00
SLISKRNPCYPYGI	2.44
LYREREEPPLPKRI	3.78
SLKNRDGPITPSTI	1.78
LPKLKSNPSVPHLH	2.93
KGVLKEYPMRPYGS	2.40
SRSKRSKPLPPQLL	2.84
DGKLKHIPRRPYEI	3.27
MPNLKRLPIPPLQD	1.91
LSGTRLVPNCPLLW	1.87
PLRERIIPILPANS	2.53
ALWAKFFPEKPLNI	2.01
NAPERAVPILPPRN	3.52
NSGEKIYPQEPPIS	1.73
SKYAKLDPSTPVSL	2.00
SVDPKNYPDAPLEG	2.06
LQYHKKNPYSPLFS	2.65
MTVPRNRPMAPFGT	3.08
FANIKREPVHPEIR	1.98
TGKPRKTPRPPFPF	3.15
NAQLRKTPLEPGLE	2.19
MSWCKERPECPLCR	3.01
EGTTKVDPIIPVPE	1.76
PKKSRVLPPLPFPL	2.97
SEAKRKFPEAPEVP	1.82
PYMYKYLPRFPNLK	2.25
LVPSKAIPPLPQLD	1.79
FHDPKFIPNKPYRQ	2.71
EFDEKLPPKKPAGP	1.61
DTYARNNPDFPPQN	2.07
MAEFRRVPLPPMAE	1.78
TQSGRMGPSEPKHR	2.86
TYLTRPLPSTPNED	2.28
TLDEKECPSVPPTV	1.76
PSRSKSNPILPIMR	2.29
EEQVKEKPNEPEGK	1.41
INNTRLSPNSPTPP	1.77
LIWHKNKPTKPDNK	2.43
SVNIKCWPLKPSNF	1.88
KSYRKADPTNPQGW	1.89
NLYQRTTPAGPDPS	2.77
INVEREAPMFPVPY	1.88
ENTPRPNPTQPRKP	2.19
IMDAKFHPFKPQLF	2.08
TKAKKEKPKPPKPI	2.74
SRVTRKRPREPKST	2.90
ILFERILPILPVES	2.18
RFSSRRAPIAPSGK	3.31
RRLKKNLPSLPTPV	3.24
KTVDKGLPMIPTYV	1.16
GLVLRSTPVIPDMK	2.07
YSTNRGIPARPCKD	2.73
FFYRRDIPDVPITT	4.19
KYIIKTEPESPEED	2.00
HNAAKPIPHVPQAS	2.06
NKIPKHLPADPIFS	2.27
VTSVKLHPNLPIVF	2.10
TYGFRVRPDFPVFG	4.09
KISPSAPSKDSFTK	2.20
KTKPTPPPKPSHLK	2.19
CILPSTPTRPLSQS	1.65
HSEPAIPYRETFLS	1.92
LRSPNTPRRLRKTL	2.37
TSSPPLPPRQNVAT	1.57
RGTPANPGRVATLI	2.17
QGLPASPSRAALRY	3.23
RIPPTVPSKIIDVV	1.63
VFLPTAPSKHMDFA	2.00
IVNPFIPRRPYSNV	3.05
PTPPSPPAKRIPRS	2.54
PFIPKFPSRESVES	1.60
KKPPKIPKKVYTLE	1.75
NVQPRKPSRVHSRN	2.95
FLPPPKPFRHTETK	2.47
SASPYRPQRLPAKF	2.71
LQIPPLPLKLLTPV	2.05
EVTPEEPSKSIDNK	1.40
IFGPFGPMKEVKIL	1.80
RERPLEPPRKPMAS	2.41
IFNPQNPLKTGFEP	1.98
CNPPTDPGRIPMSE	1.37
DVPPQLPTRTKSGE	1.88
APLPPQPRKHDKTP	2.34
SPFPSAPLRKLEIL	1.70
LKYPYFPTKMSYLQ	1.96
VNTPREPVRSSSKI	1.88
EIPPPLPPKIMVHS	2.08
NANPFLPNKHPVTQ	1.45
TSKPSLPEKPQKLR	2.40
GIHPGFPIRMVQYA	1.84
ERDPNMPKRPTNAY	2.10
QGPPNLPARDKSLV	1.80
TDSPQLPSKDKSQE	2.07
RIVPAIPKKATVDE	1.97
MAAPPVPPKQSQED	1.93
AMPPTLPHRDWKDY	2.05
VILPQIPPKLTFIL	2.03
KKDPNAPKRRLSAY	2.28
LNSPDLPERTKLRN	3.79
DPHPEDPRRIYRIY	2.82
GVFPNSPVKQTTII	1.81
EVTPEEPSKKIRTS	1.72
ENYPCPPYKIIILD	2.20
SGKPKKPYRRWTPE	2.04
KKLPTGPSKKRQRK	2.13
ARDPILPYRLVKDR	2.51
PPLPPIPTRDDMSN	1.86
AKSPLLPFKLLSDR	1.93
QGQPLPPPRGQVIL	2.36
TPLPFAPNKSSKKT	2.26
PNYPDSPPKVTFIS	1.84
EFIPPDPTKVTEFE	1.99
DFRPRMPKRQRIPP	2.89
NSLPCSPKKQPARN	1.76
LMPPDSPPRMTENT	1.67
LVIPSTPSKKCKYS	1.98
EAAPQLPSRSSAAP	2.01
PQLPKSPSRYSLSR	4.16
ENKPFKPPRRVGSN	2.53
AKQPKEPKKRTTRR	2.29
LCIPDLPLRIHNQT	1.84
AAQPPLPSRNVASG	1.99
NLDPNPPVRGENLT	1.91
SSFPTIPLRASSGA	2.67
SAIPITPTKRIRRN	2.94
LPPPIIPLRKYGHN	4.31
LPVPPPPVRPSISF	1.58
NNAPTLPKRKNPKI	3.44
IIGPDEPEKCLILK	1.98
DHKPENPIRTFKDL	2.22
HLPPPPPPRAQQQQ	1.79
CGFPLLPSKRGVTA	2.00
GNIPVFPAKFGIQF	1.66
GYDPTDPSRPRINE	1.99
GSEPIDPSKLEFAR	1.76
RKRPAPPKKKAPST	2.28
EVSPVEPKRTENER	1.91
VSDPYVPTRNTFNY	2.24
RHRPQKPVRTVIYT	2.00
VVSPSHPFKQLQKI	1.78
KPKPPKPIKYHFED	2.19
QQKPAIPQKKSFLT	2.23
PLPPIPPTKSKTSP	1.91
LSSPCLPPKVNATN	2.05
SDKPLTPTKTSSTL	2.04
QKTPPSPAKPKQKT	2.52
RVLPSKPKKSVKEN	2.19
PMSPGPPSKKDSRH	2.11
GIKPQQPIKVDIKG	2.06
SMTPDSPVKSDLTS	1.93
RTEPSTPSRPVPPD	1.74
KNFPTLPSKINSRH	2.39
FVLPCLPRKNTYKV	1.98
AVDPENPKKLASKF	1.87
TNYPVNPEKHEMDK	1.91
PAFPEYPMRRDTLY	3.50
LKRPILPSKNMDHT	2.12
DSLPKLPFRSWGQP	2.25
ELEPQHPLRHKSSL	1.81
EMIPSSPQRKSVSN	1.77
LTLPFGPVKSIWLS	0.77
IEQPFGPPKITKDG	2.08
KLPPRLPHRAAQRR	4.30
IMPPPKPFRHGLDS	2.41
CILPAFPCKSSNEQ	2.12
AGHPMKPHRIRMAH	2.14
QAHPPKPKRREGDI	2.12
QRQPKRPKRYSLLL	2.07
DESPIDPSKAPKVP	2.04
KKCPRLPHRNKKVT	2.04
FCHPLSPLKSNFID	1.79
YSNPQTPLRSLAGL	1.91
PSAPKVPFKTLDSF	1.88
KYGPLPPSRACVGS	2.74
QTYPAQPPKIGNAF	1.63
SVLPQTPLKDVMGG	2.00
KNLPTIPIRLCGID	3.33
LGAPFCPRRKDKLV	2.15
FGTPVAPTRTLSRK	3.52
IEVPQSPTKNRVKV	2.11
FYEPLDPQKDKDMT	1.85
SHQPYLPIKNKNIV	1.91
IFFPNEPIRFVYPY	1.48
TRRPHHPLKQQSTN	1.94
LDHPNIPWKLIISG	1.88
WKPPDLPIRLRKRP	3.87
LVQPNVPEKHNMST	1.72
KFHPPEPYKGKGIY	1.98
SPLPVLPRRISTHT	3.95

LQYHKKNPYSPLFS	2.00
MTVPRNRPMAPFGT	1.74
FANIKREPVHPEIR	1.34
TGKPRKTPRPPFPF	1.44
NAQLRKTPLEPGLE	1.95
MSWCKERPECPLCR	2.02
EGTTKVDPIIPVPE	0.20
PKKSRVLPPLPFPL	1.21
SEAKRKFPEAPEVP	0.85
PYMYKYLPRFPNLK	3.00
LVPSKAIPPLPQLD	1.71
FHDPKFIPNKPYRQ	1.21
EFDEKLPPKKPAGP	0.30
DTYARNNPDFPPQN	1.50
MAEFRRVPLPPMAE	1.81
TQSGRMGPSEPKHR	1.54
TLDEKECPSVPPTV	1.19
PSRSKSNPILPIMR	1.40
EEQVKEKPNEPEGK	1.46
INNTRLSPNSPTPP	0.83
LIWHKNKPTKPDNK	1.45
NLYQRTTPAGPDPS	2.10
INVEREAPMFPVPY	0.11
IMDAKFHPFKPQLF	1.63
TKAKKEKPKPPKPI	1.05
SRVTRKRPREPKST	1.61
ILFERILPILPVES	1.21
RFSSRRAPIAPSGK	1.73
RRLKKNLPSLPTPV	1.61
KTVDKGLPMIPTYV	0.70
GLVLRSTPVIPDMK	2.10
KNIIKYVPWGPPLE	1.14
YSTNRGIPARPCKD	2.03
FFYRRDIPDVPITT	1.53
KYIIKTEPESPEED	1.60
HNAAKPIPHVPQAS	0.26
NKIPKHLPADPIFS	1.32
VTSVKLHPNLPIVF	1.02
TYGFRVRPDFPVFG	1.84
KISPSAPSKDSFTK	0.11
KTKPTPPPKPSHLK	1.85
CILPSTPTRPLSQS	1.28
HSEPAIPYRETFLS	1.16
LRSPNTPRRLRKTL	2.51
TSSPPLPPRQNVAT	1.48
RGTPANPGRVATLI	0.00
QGLPASPSRAALRY	1.76
RIPPTVPSKIIDVV	1.35
VFLPTAPSKHMDFA	1.67
IVNPFIPRRPYSNV	2.15
PTPPSPPAKRIPRS	2.13
PFIPKFPSRESVES	1.85
KKPPKIPKKVYTLE	1.34
NVQPRKPSRVHSRN	3.12
FLPPPKPFRHTETK	1.83
SASPYRPQRLPAKF	2.02
LQIPPLPLKLLTPV	1.70
IFGPFGPMKEVKIL	1.37
RERPLEPPRKPMAS	1.61
IFNPQNPLKTGFEP	1.76
CNPPTDPGRIPMSE	1.75
DVPPQLPTRTKSGE	1.79
APLPPQPRKHDKTP	1.76
SPFPSAPLRKLEIL	1.60
LKYPYFPTKMSYLQ	2.00
VNTPREPVRSSSKI	1.49
EIPPPLPPKIMVHS	1.19
NANPFLPNKHPVTQ	1.73
TSKPSLPEKPQKLR	1.28
GIHPGFPIRMVQYA	2.06
ERDPNMPKRPTNAY	1.50
QGPPNLPARDKSLV	1.36
RIVPAIPKKATVDE	1.44
MAAPPVPPKQSQED	1.61
AMPPTLPHRDWKDY	1.60
VILPQIPPKLTFIL	1.00
KKDPNAPKRRLSAY	1.67
LNSPDLPERTKLRN	1.49
DPHPEDPRRIYRIY	1.61
GVFPNSPVKQTTII	1.99
EVTPEEPSKKIRTS	1.30
ENYPCPPYKIIILD	1.54
SGKPKKPYRRWTPE	1.45
KKLPTGPSKKRQRK	1.51
ARDPILPYRLVKDR	1.67
PPLPPIPTRDDMSN	1.57
AKSPLLPFKLLSDR	1.66
QGQPLPPPRGQVIL	1.92
TPLPFAPNKSSKKT	1.70
PNYPDSPPKVTFIS	1.88
EFIPPDPTKVTEFE	1.60
DFRPRMPKRQRIPP	1.99
NSLPCSPKKQPARN	1.50
LMPPDSPPRMTENT	2.21
LVIPSTPSKKCKYS	1.57
EAAPQLPSRSSAAP	1.31
PQLPKSPSRYSLSR	2.89
ENKPFKPPRRVGSN	0.56
AKQPKEPKKRTTRR	2.02
LCIPDLPLRIHNQT	1.55
AAQPPLPSRNVASG	1.80
NLDPNPPVRGENLT	1.26
SSFPTIPLRASSGA	0.96
SAIPITPTKRIRRN	2.15
LPPPIIPLRKYGHN	2.55
LPVPPPPVRPSISF	1.71
NNAPTLPKRKNPKI	1.80
IIGPDEPEKCLILK	1.53
DHKPENPIRTFKDL	2.08
HLPPPPPPRAQQQQ	1.19
CGFPLLPSKRGVTA	1.34
GNIPVFPAKFGIQF	1.77
EEKPLLPTRPNKAE	1.74
GYDPTDPSRPRINE	1.54
GSEPIDPSKLEFAR	1.40
RKRPAPPKKKAPST	1.60
EVSPVEPKRTENER	1.62
VSDPYVPTRNTFNY	1.37
RHRPQKPVRTVIYT	1.80
VVSPSHPFKQLQKI	1.70
KPKPPKPIKYHFED	1.72
QQKPAIPQKKSFLT	1.86
PLPPIPPTKSKTSP	1.32
LSSPCLPPKVNATN	1.34
SDKPLTPTKTSSTL	1.73
QKTPPSPAKPKQKT	1.64
RVLPSKPKKSVKEN	1.25
PMSPGPPSKKDSRH	2.17
GIKPQQPIKVDIKG	1.61
SMTPDSPVKSDLTS	1.59
RTEPSTPSRPVPPD	0.91
KNFPTLPSKINSRH	1.41
FVLPCLPRKNTYKV	1.31
AVDPENPKKLASKF	1.73
TNYPVNPEKHEMDK	1.51
VTVPKLPFKLAVNK	1.84
PAFPEYPMRRDTLY	1.92
LKRPILPSKNMDHT	1.66
DSLPKLPFRSWGQP	1.41
ELEPQHPLRHKSSL	1.78
EMIPSSPQRKSVSN	1.57
LTLPFGPVKSIWLS	1.62
IEQPFGPPKITKDG	1.42
KLPPRLPHRAAQRR	2.34
IMPPPKPFRHGLDS	2.35
CILPAFPCKSSNEQ	1.40
AGHPMKPHRIRMAH	2.21
QAHPPKPKRREGDI	1.58
QRQPKRPKRYSLLL	2.29
DESPIDPSKAPKVP	1.07
KKCPRLPHRNKKVT	1.67
FCHPLSPLKSNFID	1.64
YSNPQTPLRSLAGL	2.02
PSAPKVPFKTLDSF	1.63
KYGPLPPSRACVGS	1.62
QTYPAQPPKIGNAF	2.49
SVLPQTPLKDVMGG	1.01
KNLPTIPIRLCGID	1.66
LGAPFCPRRKDKLV	1.40
FGTPVAPTRTLSRK	2.49
IEVPQSPTKNRVKV	1.28
FYEPLDPQKDKDMT	1.30
SHQPYLPIKNKNIV	1.93
IFFPNEPIRFVYPY	1.56
TRRPHHPLKQQSTN	1.42
LDHPNIPWKLIISG	1.55
WKPPDLPIRLRKRP	3.30
LVQPNVPEKHNMST	1.68
KFHPPEPYKGKGIY	1.58
SPLPVLPRRISTHT	1.61
NYIPPDPPKDIQLN	1.61
QFVPIAPQRLNMIF	1.84
NSPPVYPKRIRHFE	3.49
VPRPCPPIRRKSST	2.37
YSLPMHPGRWWVHT	2.20
VMVPHLPGKILLSN	1.56
NPPPVPPPRNVTAM	1.63
DLPPEPPTKLSDSF	1.49
KPDPECPSKLRHYE	1.77
GRHPDMPTRVKNAY	1.65
PVGPKVPAKEPSPK	0.76
KIMPPTPAKKVEFI	2.25
RAVPILPPRNNVEP	1.89
CNEPHLPPKWVQRS	0.99
ITSPPLPPRADCIE	1.55
AMTPFSPRKSHINN	2.95
EIAPLIPKKPALRK	1.96
QAAPRNPNKDATIA	1.02
AFVPTNPWRSSMSL	1.60
VETPPKPIKKDVRF	1.85
EESPVKPLKITQKA	1.60
QMNPKNPIRLGMAN	2.07
HSTPVTPRRLFTSQ	1.89

PTSVPRRPGQRRPP	2.94
KFWDLRRPYEPINS	1.79
MKTSPRRPLILKRR	3.46
VHLKLRRPVTVPAG	1.81
DSGQPRRPGQCPLS	1.91
SSSAPRRPSSLQSL	3.02
DPNAPKRPPLAFFL	3.93
TSPTFRRPKTLRLR	1.74
RADIPKRPSYVAPL	3.16
IRFKLKRPLEDGGR	1.60
DESCPRRPDYLKGL	1.45
LSQPPRRPWGNLTA	2.19
FVTELRRPDAVRTK	1.63
KPQTIKRPLNPLAS	1.72
GKRPIKRPAYWRTE	1.11
SHGSPRRPGPEPGA	1.49
PQEHPKRPVLGLEC	0.65
ASLFPKRPALEPVR	2.97
ASNQPKRPPAAPEN	1.71
CGRHFRRPDPCREP	1.18
LRATLKRPEVIATC	1.99
AFVNFRRPEDARAA	1.71
TLLEPRRPRSESVT	2.05
GTPKPKRPAILTYH	3.05
KDEKPKRPEDESGR	2.50
LASTPKRPSTSQTV	1.89
RGDDLRRPATMKGK	2.16
LAQPLRRPSSVGEL	1.66
MNMTPRRPGPTRKH	3.40
HILLFRRPLPKKPK	2.18
REASFKRPVVILGP	1.26
EQRPLKRPRLGPPD	2.65
SVVGIRRPFQIPPG	2.10
LDSILRRPYVTCQP	1.74
PTGQPRRPRNLAAA	2.41
ITKSLRRPETSYRQ	1.67
FNFDIRRPSLEHRQ	3.53
QPQSLRRPLWLSHS	3.14
ISFHPKRPMHILLH	2.72
TEEELRRPEMKNFF	1.46
EGLAFRRPAEYDPK	1.60
DPSPPRRPQHNSSG	2.38
SEPHIKRPMNAFMV	1.80
GIPSPRRPMSNLWL	2.81
RPVAPRRPRDAVGQ	3.19
VETSLRRPQDKDAK	1.64
PGHSPRRPRPLGLS	3.45
TYVTPRRPFEKSRL	2.43
SSDWLRRPLGEDNQ	1.57
DPNAPKRPPSGFFL	3.73
RNPVLRRPPILEDY	1.20
DANRLRRPGGSYPA	1.78
QPLEPKRPPPPRPV	2.09
LLNTLKRPKRPPLK	1.93
SFSHLKRPQQGAGN	1.51
SGKGLRRPHCTVEE	1.24
WLTCLKRPLVVIHE	2.51
YKVNLKRPCPFWND	1.46
RHLSPRRPVSPGKD	2.52
FERPFRRPNANFTS	2.53
PSGHIKRPMNAFMV	1.88
EDEEPKRPLPRKQS	1.19
QQPLPRRPGHDDGQ	2.02
NSMGIKRPAPQPAS	2.22
SGGRLRRPAHVPLD	1.39
TNPELRRPRSEPSN	1.61
ASAPPRRPRPPPPS	3.96
CKKGFRRPSEKNQH	1.51
NYKNIKRPPGDIMF	1.56
ALLDPRRPEIPTEQ	1.57
PEVYLKRPAHSDDW	1.45
DPEVLKRPEYFGKF	2.04
EHPRLRRPMPWLLL	2.89
ARPELRRPGVQGIP	1.87
QLVILRRPERPMED	1.22
KSLKLKRPLYAHLA	1.58
TRKGPKRPQQPNPP	3.36
GWATPRRPAGLLML	2.39
PAHPFRRPKLSGRS	1.81
MAASLKRPDSGATG	1.54
GSEKPKRPVSAMFI	3.40
KSRTLKRPTVLEPI	1.71
VKLKIKRPYGRKSQ	1.51
LRGKFKRPPLRRVR	1.70
NSSHIKRPMNAFMV	2.25
GQKQPKRPRSESSC	1.64
PAPGLRRPLSPPAA	2.02
EPPAPRRPASLAQR	2.92
MKVHIKRPEMTSSS	2.21
NGYFFKRPYPFVLF	2.03
PLRHFKRPAYASSS	0.98
SQEILRRPIVACEL	1.73
FSESLKRPWEDDPP	1.55
NINGIRRPLQGVAN	1.65
SLRVPKRPIINTPV	3.39
DNGGFKRPLAPSGR	1.99
RGAQLRRPRHSHPT	1.60
LKVQLKRPKDANRP	1.84
LCNILRRPIIVISD	1.93
VESILKRPSSAITR	1.50
TPGEFRRPRLASFG	2.86
QVYVLKRPHVDEFL	1.77
IKTPPKRPGGRRRG	2.80
VRLLIRRPECFGPA	2.73
RLAALKRPDDVPLS	1.69
GSPAPKRPPDPFPG	2.85
PPPLPRRPSTPRQG	3.98
SWERPRRPNGDIVG	3.44
IFMYLRRPTEESVE	2.22
PDPAIRRPAEKFLE	1.72
DTLNIRRPLVKMVY	1.78
CCSGPKRPQDKVAV	1.70
GQKLPRRPQPGPGE	1.63
THLLIKRPPFFHYR	3.31
PDETLRRPGGPQYG	1.09
REDRFRRPRDEGGW	2.06
CFHSPRRPYASPLL	3.04
CRNVLRRPFQAQCG	2.05
SMFFIRRPTLDINW	2.87
SAGRPKRPHARRAL	2.85
HLAFLKRPELGLTL	1.46
LLGPFRRPTTQLRA	2.79
VSMQLRRPSDRELS	2.81
VARSPKRPAWGSRR	3.02
EREALKRPMGPVHY	1.70
SCNFPRRPDSGDVT	1.83
ALQSPKRPRSPGSN	2.41
CEVPLKRPRLDKNS	1.82
RRIHIKRPDDRAHL	1.85
EGISLRRPNCFGIA	1.71
PEDAPKRPKEAAPD	1.37
PGAGLRRPTYTPPS	2.23
ARKQPRRPHFLLEP	1.50
GQVILRRPLDYEKN	1.32
IIRQIRRPKNVVLR	1.69
TPKPPKRPRLQRPA	3.92
LANELRRPPLCYHN	2.24
PGFSLKRPRGLSRS	1.62
KTTPLRRPTETNPV	1.76
PAAPPKRPAVASAR	3.34
EVLELRRPMDSRLE	1.54
PPKWIRRPTGVSFA	2.64
SSKPIKRPVLDPFI	2.28
RVKYIKRPGAVLEA	1.56
FSSFLKRPKSQVSE	1.71
QSLKIRRPHDYQPL	1.97
TSLSLRRPPGPASQ	1.43
GQCAPRRPCAGDGA	2.15
LVIRLRRPPLRYPR	3.79
ASAPPRRPRPPPQS	3.12
SFTALRRPSLRREA	1.44
RDAFLKRPISNKYM	2.40
GTVELRRPGQSHAA	1.63
TYQHLRRPRGLSSL	2.29
PVLDLKRPFLSRES	1.70
PLLPPKRPLSSSHE	4.02
RGQEFRRPCTLSEL	2.20
PVLDLRRPPSDLYL	1.80
SGSKLKRPTPTFHS	1.61
ESPPPKRPRIAYLS	2.52
ARLYLRRPAADGAE	1.96
PRPRPRRPRQLTRA	2.31
ALTLPRRPFLHCST	3.88
LCKTLRRPRCLLET	1.72
IMFDLRRPPQNHTY	2.36
SPVHLRRPQSPGAS	1.75
LSGGLKRPSTRTPN	1.43
VRSRFKRPKPNLAR	1.63
KLGTLRRPEGPAEP	1.02
FLCIPRRPNWNQNT	3.09
SPQAPRRPLPQVAT	2.64
GPAPLRRPDSSDDR	1.66
MFQALKRPDDTAFQ	1.74
GHGEPRRPPCDPLA	1.81
PHHELRRPVLQRQT	0.61
SGDQLKRPFGAFRS	1.88
KPVSPRRPWSPSKE	3.25
VNLKLRRPSFRDDI	1.78
PMAPIRRPISAESA	2.67
EGVPFRRPSTFGIP	2.05
KQYMLRRPALEDYV	2.17
MSLLLRRPPGREAY	1.80
GSELPRRPLPPAAQ	1.85
EGSMLRRPRTSSQH	2.65
EERPLRRPRPVQRT	2.83
IKFVIKRPELLTHS	1.80
HLWQPKRPAVPKKA	2.91
GPLDLRRPMRTVPG	2.99
ARNQLRRPQYRPQY	1.43
GSSALKRPFEDGLG	1.60
KGRVLKRPPNSVVT	1.40
GARSPKRPRLQLPG	3.08
TQPVLKRPRHVHCH	1.78

MYGSKFIPFGPKCQ	2.46
QGSNKYSPSSPLTT	2.23
PKCEKIEPLSPSFK	2.07
AGLYKLSPLYPFDV	2.12
VYRRKCIPFAPHQI	2.58
VLTVKMSPSLPDEI	2.36
GTRPKILPKIPTGA	2.89
WSQMRMNPTNPDWI	1.91
DNELKDEPEPPNLI	2.25
KAALKYHPDKPTGD	2.19
NFPQRMMPPPPGLV	2.79
LAYERPLPDLPSTI	2.12
MKAYRANPPLPIDS	2.09
PFIRRYTPLKPITK	2.81
YAVGRIVPDSPTYD	2.28
FSTKKSKPIRPFVF	2.58
IESLREIPYGPDFD	2.06
QHKPKVKPSKPEAK	2.98
KTKKKTVPVKPLQL	2.55
TELLKTLPFYPTPS	1.89
IGKSKSAPPIPEDT	2.39
KKNKKKVPLNPLAK	2.72
MTQPKKNPMEPKTD	2.17
ITDTKLFPGNPNLM	1.98
TSCTKEKPTPPTTT	2.07
AGHIKTHPNSPTPG	1.78
KIVRRDYPSTPVIV	2.56
ETSLRVLPIAPKIF	2.74
SKRMKALPKGPAAL	2.91
ERDGRGVPLGPRNH	2.08
LWRPRIVPILPYIT	3.02
AKSNKVSPEDPAVQ	2.57
DLDWRKIPFLPKEI	2.40
GYDEKGNPIPPNNW	1.98
KQLKKQGPKRPSSA	2.67
SENGRLTPGLPRSY	2.23
LTSVKIVPQRPISD	1.95
TIEARVHPEPPNKP	2.57
LDSMKNLPLNPSKK	2.43
SLISKRNPCYPYGI	2.68
LYREREEPPLPKRI	2.05
SLKNRDGPITPSTI	2.33
LPKLKSNPSVPHLH	2.29
KGVLKEYPMRPYGS	2.07
SRSKRSKPLPPQLL	2.95
DGKLKHIPRRPYEI	2.49
MPNLKRLPIPPLQD	2.49
LSGTRLVPNCPLLW	2.64
PLRERIIPILPANS	2.24
ALWAKFFPEKPLNI	2.19
NAPERAVPILPPRN	2.87
SKVNKVAPDHPFIQ	1.86
NSGEKIYPQEPPIS	2.05
SKYAKLDPSTPVSL	2.66
SVDPKNYPDAPLEG	1.96
LQYHKKNPYSPLFS	2.50
MTVPRNRPMAPFGT	2.77
FANIKREPVHPEIR	2.27
TGKPRKTPRPPFPF	3.08
NAQLRKTPLEPGLE	2.25
MSWCKERPECPLCR	2.43
EGTTKVDPIIPVPE	2.26
PKKSRVLPPLPFPL	3.55
SEAKRKFPEAPEVP	2.59
PYMYKYLPRFPNLK	2.58
LVPSKAIPPLPQLD	2.75
FHDPKFIPNKPYRQ	2.48
EFDEKLPPKKPAGP	1.94
DTYARNNPDFPPQN	2.14
MAEFRRVPLPPMAE	2.65
TQSGRMGPSEPKHR	2.66
TYLTRPLPSTPNED	2.00
TLDEKECPSVPPTV	2.33
PSRSKSNPILPIMR	2.72
EEQVKEKPNEPEGK	2.13
INNTRLSPNSPTPP	2.14
LIWHKNKPTKPDNK	2.48
SVNIKCWPLKPSNF	1.75
KSYRKADPTNPQGW	2.01
NLYQRTTPAGPDPS	2.06
INVEREAPMFPVPY	2.17
ENTPRPNPTQPRKP	2.14
IMDAKFHPFKPQLF	2.39
TKAKKEKPKPPKPI	2.20
SRVTRKRPREPKST	2.92
ILFERILPILPVES	2.53
RFSSRRAPIAPSGK	2.75
RRLKKNLPSLPTPV	2.92
KTVDKGLPMIPTYV	1.89
GLVLRSTPVIPDMK	2.32
KNIIKYVPWGPPLE	2.22
YSTNRGIPARPCKD	2.43
FFYRRDIPDVPITT	2.14
KYIIKTEPESPEED	2.39
HNAAKPIPHVPQAS	1.79
NKIPKHLPADPIFS	2.44
VTSVKLHPNLPIVF	2.14
TYGFRVRPDFPVFG	2.55
KAVHLYFPILPHCG	2.10
YPEKFQKPYNPLAM	2.20
GEYDLIMPIYPYRL	2.38
EFDMLLLPFIPSID	2.40
MFNLLQKPEQPHQN	2.31
IFQSLLHPELPIII	2.38
KLAPLNCPSLPTTN	2.33
VLLPLRLPEIPPKG	3.03
REASLDIPYKPIIA	2.25
PFIDYEFPHLPNEL	2.00
PFGTFTLPADPEYK	2.23
LASPFHDPILPRRT	2.46
AAMNLETPSNPDSA	1.93
PEGVFCIPAPPPNV	1.92
RLFDLVKPKNPKFS	2.64
PPPDFNDPTAPLSS	2.18
KPPVYRLPPLPRLK	3.36
AEAYLVTPEEPAKN	1.60
EAYRLNYPELPRLT	2.10
SPKFLNSPDLPERT	2.26
KLPDLKLPPSPVPS	2.36
MLNEHGVPVVPSRE	2.30
RERPLEPPRKPMAS	2.99
VAGYFVIPHTPMPS	2.25
QLRSLFKPFGPILN	2.57
VFKFLFHPVTPSHF	2.75
TEDGFVIPALPAAV	2.46
FFFFHLSPSIPFWF	3.81
SPLSWSGPLTPPTF	2.23
EADGLVQPVVPDKV	1.88
FFQLMQSPSLPLLM	2.92
SANNYPFPQQPNGM	1.90
KFRQMFKPLAPNTR	3.09
LLAEHNGPKLPHIP	2.30
DFIKLMNPTLPSLK	2.18
ILTLLKMPEVPNSV	2.24
SLVKFSCPMTPLLE	2.40
KPMLHNVPHDPSLP	2.62
VLTKMNAPQKPEDI	2.47
KFVIHNEPIDPSDK	1.67
VPIIFEIPFNPIYK	2.33
VAWCHDSPKPPSDR	1.49
HLFHLTTPQDPRLL	2.39
RPPSHSAPYIPRSS	4.68
NPFPFPIPEIPSTQ	1.94
QPQHLPPPPPPRAQ	4.88
HAEAFEDPSIPRIS	2.51
WETWLYKPGMPPRP	2.41
LLAKLPLPRDPRVA	2.54
SLCALVGPVIPHTI	1.45
KPKMLPIPSKPTLF	3.13
TAPIHSTPKPPPND	1.89
PLTTLPPPRLPSIQ	3.45
KPSLWEAPQNPPYS	2.18
FEMVFRHPEQPHMI	2.49
IEEGHLDPEAPDKL	1.93
NFPSLIDPHVPTYG	1.99
VELHFPKPEDPACI	1.17
GPSQFAPPPPPPGV	4.31
YEGRFWNPENPYKF	2.21
KEVDLTRPSAPSEP	2.12
VLKNLPTPGEPFNF	2.17
KWTIMPHPNRPNAA	2.63
GLLLLFVPDSPYDP	1.33
SPVPLGSPRGPLPV	2.13
KPKVLFHPHWPLYI	2.29
GFVFFIAPHLPVDL	2.71
ELFTFDMPTDPSSF	1.65
EAFKLPKPWSPNSH	2.15
DFNKFERPNIPVEE	2.33
VPPNFNAPPNPMAF	3.20
SWRSLTKPKIPLYK	2.55
KLAILTCPFEPPKP	2.24
GPIIHPGPEPPRYD	2.31
ILVPHLDPIDPWEA	2.22
GLRELFLPGMPGLM	2.20
REDAHEDPVSPKAG	2.16
YENCHSLPLDPQSP	2.09
PEPTMGSPDAPPLV	2.03
ALYHLSNPNVPKLW	2.29
KASSMPAPPPPPPP	4.02
ILQILFEPMPPFNS	2.30
ALDNFTLPGTPGFP	2.26
EFSPHSTPVTPRRL	2.06
IEVPMEYPFKPPKM	2.58
QPAQFMPPQDPAAA	2.13
FPKKMGPPQWPFST	2.45
FEESMNNPLPPLPP	2.23
TLRSMIPPFTPQLD	2.62
MLKALFTPLIPVSF	2.41
KELNLTGPKVPEDI	2.01
ELAEFTDPSCPVSE	2.28
IEITHSEPAIPYRE	2.06
HATLYWAPVSPLKS	2.18
AADWYEVPKVPLSA	2.44

PKCEKIEPLSPSFK	1.65
AGLYKLSPLYPFDV	1.25
VYRRKCIPFAPHQI	2.14
VLTVKMSPSLPDEI	0.64
GTRPKILPKIPTGA	1.34
WSQMRMNPTNPDWI	2.95
DNELKDEPEPPNLI	1.40
KAALKYHPDKPTGD	1.93
NFPQRMMPPPPGLV	1.83
LAYERPLPDLPSTI	3.55
MKAYRANPPLPIDS	2.16
PFIRRYTPLKPITK	2.77
YAVGRIVPDSPTYD	2.10
FSTKKSKPIRPFVF	2.36
IESLREIPYGPDFD	1.17
QHKPKVKPSKPEAK	1.85
KTKKKTVPVKPLQL	1.65
TELLKTLPFYPTPS	1.76
IGKSKSAPPIPEDT	1.41
KKNKKKVPLNPLAK	1.81
MTQPKKNPMEPKTD	2.26
ITDTKLFPGNPNLM	1.83
TSCTKEKPTPPTTT	1.54
AGHIKTHPNSPTPG	1.33
KIVRRDYPSTPVIV	2.31
ETSLRVLPIAPKIF	2.88
SKRMKALPKGPAAL	2.31
ERDGRGVPLGPRNH	1.55
LWRPRIVPILPYIT	2.80
AKSNKVSPEDPAVQ	2.20
DLDWRKIPFLPKEI	2.25
GYDEKGNPIPPNNW	1.66
KQLKKQGPKRPSSA	1.91
SENGRLTPGLPRSY	2.37
LTSVKIVPQRPISD	2.39
TIEARVHPEPPNKP	1.42
LDSMKNLPLNPSKK	1.86
SLISKRNPCYPYGI	1.66
LYREREEPPLPKRI	3.44
SLKNRDGPITPSTI	2.37
LPKLKSNPSVPHLH	1.72
KGVLKEYPMRPYGS	1.58
SRSKRSKPLPPQLL	2.20
DGKLKHIPRRPYEI	1.84
MPNLKRLPIPPLQD	1.82
LSGTRLVPNCPLLW	3.16
PLRERIIPILPANS	2.42
ALWAKFFPEKPLNI	1.71
NAPERAVPILPPRN	4.22
SKVNKVAPDHPFIQ	1.11
NSGEKIYPQEPPIS	1.64
SKYAKLDPSTPVSL	1.63
SVDPKNYPDAPLEG	1.25
LQYHKKNPYSPLFS	2.32
MTVPRNRPMAPFGT	2.27
FANIKREPVHPEIR	1.60
TGKPRKTPRPPFPF	3.56
NAQLRKTPLEPGLE	2.06
MSWCKERPECPLCR	2.38
EGTTKVDPIIPVPE	1.73
PKKSRVLPPLPFPL	4.28
SEAKRKFPEAPEVP	2.07
PYMYKYLPRFPNLK	2.96
LVPSKAIPPLPQLD	1.46
FHDPKFIPNKPYRQ	1.46
EFDEKLPPKKPAGP	1.51
DTYARNNPDFPPQN	1.67
MAEFRRVPLPPMAE	4.48
TQSGRMGPSEPKHR	2.53
TYLTRPLPSTPNED	4.07
TLDEKECPSVPPTV	1.93
PSRSKSNPILPIMR	2.39
EEQVKEKPNEPEGK	1.36
INNTRLSPNSPTPP	1.50
LIWHKNKPTKPDNK	1.93
SVNIKCWPLKPSNF	1.79
KSYRKADPTNPQGW	1.75
NLYQRTTPAGPDPS	2.54
INVEREAPMFPVPY	1.57
ENTPRPNPTQPRKP	1.73
IMDAKFHPFKPQLF	2.33
TKAKKEKPKPPKPI	1.51
SRVTRKRPREPKST	2.21
ILFERILPILPVES	2.66
RFSSRRAPIAPSGK	2.89
RRLKKNLPSLPTPV	2.45
KTVDKGLPMIPTYV	1.38
GLVLRSTPVIPDMK	3.72
KNIIKYVPWGPPLE	1.21
YSTNRGIPARPCKD	3.93
FFYRRDIPDVPITT	2.40
KYIIKTEPESPEED	1.53
HNAAKPIPHVPQAS	1.31
NKIPKHLPADPIFS	1.83
VTSVKLHPNLPIVF	1.87
TYGFRVRPDFPVFG	2.56
KISPSAPSKDSFTK	1.90
KTKPTPPPKPSHLK	0.91
CILPSTPTRPLSQS	2.29
HSEPAIPYRETFLS	1.55
LRSPNTPRRLRKTL	3.41
TSSPPLPPRQNVAT	4.22
RGTPANPGRVATLI	1.69
QGLPASPSRAALRY	1.80
RIPPTVPSKIIDVV	1.65
VFLPTAPSKHMDFA	2.32
IVNPFIPRRPYSNV	2.81
PTPPSPPAKRIPRS	1.77
PFIPKFPSRESVES	2.70
KKPPKIPKKVYTLE	1.52
NVQPRKPSRVHSRN	3.28
FLPPPKPFRHTETK	3.08
SASPYRPQRLPAKF	1.83
LQIPPLPLKLLTPV	1.89
EVTPEEPSKSIDNK	1.29
IFGPFGPMKEVKIL	1.75
RERPLEPPRKPMAS	2.78
IFNPQNPLKTGFEP	1.79
CNPPTDPGRIPMSE	1.61
DVPPQLPTRTKSGE	4.37
APLPPQPRKHDKTP	1.72
SPFPSAPLRKLEIL	1.47
LKYPYFPTKMSYLQ	1.59
VNTPREPVRSSSKI	1.84
EIPPPLPPKIMVHS	2.13
NANPFLPNKHPVTQ	1.91
TSKPSLPEKPQKLR	1.80
GIHPGFPIRMVQYA	1.39
ERDPNMPKRPTNAY	1.81
QGPPNLPARDKSLV	4.27
TDSPQLPSKDKSQE	1.98
RIVPAIPKKATVDE	4.04
MAAPPVPPKQSQED	1.72
AMPPTLPHRDWKDY	3.42
VILPQIPPKLTFIL	1.00
KKDPNAPKRRLSAY	2.18
LNSPDLPERTKLRN	4.37
DPHPEDPRRIYRIY	1.15
GVFPNSPVKQTTII	0.60
EVTPEEPSKKIRTS	1.19
ENYPCPPYKIIILD	1.55
SGKPKKPYRRWTPE	2.29
KKLPTGPSKKRQRK	0.96
ARDPILPYRLVKDR	2.64
PPLPPIPTRDDMSN	2.09
AKSPLLPFKLLSDR	1.65
QGQPLPPPRGQVIL	3.92
TPLPFAPNKSSKKT	1.46
PNYPDSPPKVTFIS	1.10
EFIPPDPTKVTEFE	1.21
DFRPRMPKRQRIPP	1.94
NSLPCSPKKQPARN	2.32
LMPPDSPPRMTENT	0.08
LVIPSTPSKKCKYS	1.51
EAAPQLPSRSSAAP	4.36
PQLPKSPSRYSLSR	2.59
ENKPFKPPRRVGSN	2.36
AKQPKEPKKRTTRR	2.11
LCIPDLPLRIHNQT	3.43
AAQPPLPSRNVASG	4.78
NLDPNPPVRGENLT	1.42
SSFPTIPLRASSGA	2.90
SAIPITPTKRIRRN	1.86
LPPPIIPLRKYGHN	3.28
LPVPPPPVRPSISF	2.89
NNAPTLPKRKNPKI	4.30
IIGPDEPEKCLILK	1.37
DHKPENPIRTFKDL	2.08
HLPPPPPPRAQQQQ	3.28
CGFPLLPSKRGVTA	2.55
GNIPVFPAKFGIQF	1.70
EEKPLLPTRPNKAE	3.81
GYDPTDPSRPRINE	1.85
GSEPIDPSKLEFAR	1.52
RKRPAPPKKKAPST	1.77
EVSPVEPKRTENER	1.67
VSDPYVPTRNTFNY	1.78
RHRPQKPVRTVIYT	1.68
VVSPSHPFKQLQKI	1.75
KPKPPKPIKYHFED	0.83
QQKPAIPQKKSFLT	1.63
PLPPIPPTKSKTSP	1.48
LSSPCLPPKVNATN	1.54
SDKPLTPTKTSSTL	2.00
QKTPPSPAKPKQKT	2.19
RVLPSKPKKSVKEN	1.63
PMSPGPPSKKDSRH	2.14
GIKPQQPIKVDIKG	1.59
SMTPDSPVKSDLTS	1.50
RTEPSTPSRPVPPD	1.20
KNFPTLPSKINSRH	1.55
FVLPCLPRKNTYKV	1.21
AVDPENPKKLASKF	0.72

VLTVKMSPSLPDEI	1.69
GTRPKILPKIPTGA	4.07
WSQMRMNPTNPDWI	2.12
DNELKDEPEPPNLI	1.51
KAALKYHPDKPTGD	1.78
NFPQRMMPPPPGLV	1.87
LAYERPLPDLPSTI	4.81
MKAYRANPPLPIDS	2.36
PFIRRYTPLKPITK	1.77
YAVGRIVPDSPTYD	1.60
FSTKKSKPIRPFVF	2.26
IESLREIPYGPDFD	1.68
QHKPKVKPSKPEAK	1.95
KTKKKTVPVKPLQL	1.53
TELLKTLPFYPTPS	1.73
IGKSKSAPPIPEDT	0.83
KKNKKKVPLNPLAK	2.01
MTQPKKNPMEPKTD	1.20
ITDTKLFPGNPNLM	1.64
TSCTKEKPTPPTTT	1.32
AGHIKTHPNSPTPG	2.21
KIVRRDYPSTPVIV	1.53
ETSLRVLPIAPKIF	3.46
SKRMKALPKGPAAL	3.80
ERDGRGVPLGPRNH	1.58
LWRPRIVPILPYIT	2.05
AKSNKVSPEDPAVQ	1.64
DLDWRKIPFLPKEI	3.03
GYDEKGNPIPPNNW	1.82
KQLKKQGPKRPSSA	2.18
SENGRLTPGLPRSY	1.55
LTSVKIVPQRPISD	1.18
TIEARVHPEPPNKP	1.51
LDSMKNLPLNPSKK	3.90
SLISKRNPCYPYGI	2.20
LYREREEPPLPKRI	2.28
SLKNRDGPITPSTI	1.41
LPKLKSNPSVPHLH	2.06
KGVLKEYPMRPYGS	1.89
SRSKRSKPLPPQLL	3.24
DGKLKHIPRRPYEI	1.69
MPNLKRLPIPPLQD	4.39
LSGTRLVPNCPLLW	1.97
PLRERIIPILPANS	2.98
ALWAKFFPEKPLNI	2.06
NAPERAVPILPPRN	1.72
NSGEKIYPQEPPIS	1.41
SKYAKLDPSTPVSL	1.51
SVDPKNYPDAPLEG	1.45
LQYHKKNPYSPLFS	1.80
MTVPRNRPMAPFGT	2.27
FANIKREPVHPEIR	1.88
TGKPRKTPRPPFPF	2.08
NAQLRKTPLEPGLE	1.69
MSWCKERPECPLCR	2.19
EGTTKVDPIIPVPE	1.85
PKKSRVLPPLPFPL	4.40
SEAKRKFPEAPEVP	0.58
PYMYKYLPRFPNLK	3.20
LVPSKAIPPLPQLD	2.01
FHDPKFIPNKPYRQ	2.09
EFDEKLPPKKPAGP	1.59
DTYARNNPDFPPQN	0.69
MAEFRRVPLPPMAE	1.76
TQSGRMGPSEPKHR	1.89
TYLTRPLPSTPNED	4.15
TLDEKECPSVPPTV	1.51
PSRSKSNPILPIMR	1.99
EEQVKEKPNEPEGK	1.49
INNTRLSPNSPTPP	1.63
LIWHKNKPTKPDNK	2.03
SVNIKCWPLKPSNF	1.70
KSYRKADPTNPQGW	1.46
NLYQRTTPAGPDPS	1.78
INVEREAPMFPVPY	1.69
ENTPRPNPTQPRKP	1.12
IMDAKFHPFKPQLF	1.97
TKAKKEKPKPPKPI	1.75
SRVTRKRPREPKST	1.98
ILFERILPILPVES	3.75
RFSSRRAPIAPSGK	2.09
RRLKKNLPSLPTPV	4.43
KTVDKGLPMIPTYV	3.92
GLVLRSTPVIPDMK	1.78
KNIIKYVPWGPPLE	1.52
YSTNRGIPARPCKD	2.25
FFYRRDIPDVPITT	2.92
KYIIKTEPESPEED	1.74
HNAAKPIPHVPQAS	1.91
NKIPKHLPADPIFS	1.83
VTSVKLHPNLPIVF	1.76
TYGFRVRPDFPVFG	1.85
MVRPAPPRRADPEG	2.78
QPAPRIPSKDAIIS	2.70
FPDPNNPSKVQIRM	2.22
ENFPIVPLKSVAQD	1.66
KIMPPTPAKKVEFI	3.09
TTRPAPPSKAKDVA	2.88
GNIPVFPAKFGIQF	2.33
ATVPSAPYRKQIIS	2.08
KISPSAPSKDSFTK	1.90
LSKPSKPLKEIRFD	2.68
IAYPGDPLRFHSHL	2.24
DYQPLPPSRELDPD	1.88
ATRPSSPMRHKSIS	2.60
ACLPVGPRKNGMNV	2.33
LSLPRLPNKHHWPL	3.52
APLPPQPRKHDKTP	2.18
KPDPECPSKLRHYE	2.34
CGFPLLPSKRGVTA	2.81
QSNPTSPTKQTFTI	1.59
PNEPGAPSRLSFEN	1.93
DRPPDVPRKIIVGR	2.68
QLTPLLPDKYFDSA	1.76
DHKPENPIRTFKDL	2.23
DDTPSIPSKISSKS	2.32
IINPDTPHRRIFNQ	2.24
LRRPIFPGRDYRHQ	2.05
DSIPVDPEKVVAIV	1.96
GPPPAMPARPTATT	1.95
ELPPIKPPRPTSTT	2.01
DLQPLEPKKAAAST	2.34
APAPLDPFKTGGNN	2.83
SNSPQIPKKKVKDQ	1.83
SLPPTPPYKAHKKK	2.74
GVYPSRPEKTYDYS	2.04
PFIPKFPSRESVES	2.21
QGLPASPSRAALRY	2.15
HNSPVAPNRYAANL	2.01
VTVPKLPFKLAVNK	2.11
EYSPLVPSKMCADK	2.26
DSLPFAPLRKVYEL	1.99
VPPPNVPKKDKREV	4.05
ALKPKTPSKAPRKR	2.92
PPEPATPSKKSLTT	1.66
ENKPFKPPRRVGSN	2.22
TDYPFKPPKISFTT	2.25
AKQPKEPKKRTTRR	2.01
KKDPNAPKRRLSAY	2.18
EMIPSSPQRKSVSN	2.21
FDFPLSPVKHENPT	1.44
QKTPPSPAKPKQKT	2.31
KSFPPPPLKPGQKT	1.93
QGQPLPPPRGQVIL	2.20
GGQPPVPVRMQPQP	2.08
IMPPPKPFRHGLDS	3.94
RAVPILPPRNNVEP	2.10
FLPPPKPFRHTETK	2.68
FQPPPKPFRRSQSS	2.95
APTPPAPSRSEKLH	2.41
NAPPYVPQRSKQPD	2.16
ITLPRRPAKKIQLG	2.45
DSLPKLPFRSWGQP	2.52
GAKPKIPSKLYQSV	2.16
TNYPVNPEKHEMDK	1.47
KSSPWSPYKVEKVL	2.14
VDAPINPTKILTFI	2.24
IIFPYDPLKLGTHA	2.05
SSIPPNPGKTAKPI	2.08
DFTPVEPSKMREYI	1.96
TPAPNLPEKTRLAL	1.78
NYIPPDPPKDIQLN	1.58
KYDPDNPNKDKLGT	2.20
NIQPDLPPRKIMIP	2.29
PKDPCSPQRDEILT	2.12
LMPPDSPPRMTENT	2.08
IAHPVRPHRAITMT	2.05
LPVPQLPPKLLVYP	1.21
TPPPVEPLKTKDLA	2.11
VLEPSKPYKKNPFM	2.08
VHGPGIPMKSLEVA	2.30
AKSPLLPFKLLSDR	2.20
MVTPSKPPKKSVGF	2.87
NSLPCSPKKQPARN	2.30
IPTPSKPLKKRKKR	1.94
EILPVKPTKKSSKK	2.45
QRHPKSPVKKPMTS	2.28
LIPPGFPTKNYPPG	1.90
MALPRTPLKTSTEE	3.04
DQAPSLPPKPNTQL	2.25
QNTPPLPPKPKSPH	3.93
AVDPENPKKLASKF	1.92
VFLPTAPSKHMDFA	2.53
HSSPIAPSKAKRQR	2.30
DPLPYSPLKLYDQK	2.16
GRIPQLPNRDEMTA	1.59
LCIPDLPLRIHNQT	2.01
FSKPQEPVKAEEAE	2.41
AAQPPLPSRNVASG	1.91
EIAPSLPSRNSIPA	2.21
EAAPQLPSRSSAAP	1.94
CREPEVPQRLLTLI	2.02
DVLPAGPVRSLEGA	2.06

SKVNKVAPDHPFIQ	1.13
NSGEKIYPQEPPIS	1.36
SKYAKLDPSTPVSL	1.42
SVDPKNYPDAPLEG	1.68
LQYHKKNPYSPLFS	1.87
FANIKREPVHPEIR	1.87
TGKPRKTPRPPFPF	3.80
NAQLRKTPLEPGLE	2.57
MSWCKERPECPLCR	3.24
PKKSRVLPPLPFPL	4.11
SEAKRKFPEAPEVP	1.27
PYMYKYLPRFPNLK	3.06
LVPSKAIPPLPQLD	2.35
FHDPKFIPNKPYRQ	2.70
EFDEKLPPKKPAGP	1.86
DTYARNNPDFPPQN	1.24
MAEFRRVPLPPMAE	4.48
TQSGRMGPSEPKHR	2.80
TYLTRPLPSTPNED	4.30
TLDEKECPSVPPTV	1.43
PSRSKSNPILPIMR	3.10
INNTRLSPNSPTPP	1.77
LIWHKNKPTKPDNK	2.94
SVNIKCWPLKPSNF	1.77
KSYRKADPTNPQGW	1.98
NLYQRTTPAGPDPS	2.59
INVEREAPMFPVPY	1.39
ENTPRPNPTQPRKP	2.31
IMDAKFHPFKPQLF	2.91
TKAKKEKPKPPKPI	1.98
SRVTRKRPREPKST	2.83
ILFERILPILPVES	3.27
RRLKKNLPSLPTPV	2.60
KTVDKGLPMIPTYV	1.08
GLVLRSTPVIPDMK	3.38
KNIIKYVPWGPPLE	0.11
YSTNRGIPARPCKD	4.24
FFYRRDIPDVPITT	2.92
KYIIKTEPESPEED	0.84
HNAAKPIPHVPQAS	0.93
NKIPKHLPADPIFS	2.92
VTSVKLHPNLPIVF	1.97
TYGFRVRPDFPVFG	2.95
KISPSAPSKDSFTK	2.25
CILPSTPTRPLSQS	4.31
HSEPAIPYRETFLS	1.97
LRSPNTPRRLRKTL	4.32
TSSPPLPPRQNVAT	4.40
RGTPANPGRVATLI	2.17
QGLPASPSRAALRY	2.72
RIPPTVPSKIIDVV	2.79
VFLPTAPSKHMDFA	2.38
IVNPFIPRRPYSNV	3.89
PTPPSPPAKRIPRS	3.94
PFIPKFPSRESVES	3.41
KKPPKIPKKVYTLE	3.11
NVQPRKPSRVHSRN	4.30
FLPPPKPFRHTETK	4.56
SASPYRPQRLPAKF	3.38
LQIPPLPLKLLTPV	3.22
IFGPFGPMKEVKIL	2.29
RERPLEPPRKPMAS	4.13
IFNPQNPLKTGFEP	2.23
CNPPTDPGRIPMSE	1.36
DVPPQLPTRTKSGE	4.45
APLPPQPRKHDKTP	3.06
SPFPSAPLRKLEIL	1.93
LKYPYFPTKMSYLQ	2.47
VNTPREPVRSSSKI	2.96
EIPPPLPPKIMVHS	4.62
NANPFLPNKHPVTQ	3.15
TSKPSLPEKPQKLR	4.07
GIHPGFPIRMVQYA	2.52
ERDPNMPKRPTNAY	3.21
QGPPNLPARDKSLV	4.56
TDSPQLPSKDKSQE	3.64
RIVPAIPKKATVDE	4.12
MAAPPVPPKQSQED	3.66
AMPPTLPHRDWKDY	4.16
VILPQIPPKLTFIL	2.91
KKDPNAPKRRLSAY	2.38
LNSPDLPERTKLRN	4.55
DPHPEDPRRIYRIY	2.21
GVFPNSPVKQTTII	2.43
EVTPEEPSKKIRTS	1.18
SGKPKKPYRRWTPE	3.60
KKLPTGPSKKRQRK	2.91
ARDPILPYRLVKDR	4.23
PPLPPIPTRDDMSN	3.31
AKSPLLPFKLLSDR	3.63
QGQPLPPPRGQVIL	4.36
TPLPFAPNKSSKKT	2.50
PNYPDSPPKVTFIS	1.94
EFIPPDPTKVTEFE	1.43
DFRPRMPKRQRIPP	2.92
NSLPCSPKKQPARN	2.52
LMPPDSPPRMTENT	3.18
LVIPSTPSKKCKYS	2.80
EAAPQLPSRSSAAP	4.74
PQLPKSPSRYSLSR	3.21
ENKPFKPPRRVGSN	4.36
AKQPKEPKKRTTRR	2.93
LCIPDLPLRIHNQT	4.41
AAQPPLPSRNVASG	4.67
NLDPNPPVRGENLT	2.07
SSFPTIPLRASSGA	3.50
SAIPITPTKRIRRN	3.43
LPPPIIPLRKYGHN	4.20
LPVPPPPVRPSISF	4.06
NNAPTLPKRKNPKI	4.61
IIGPDEPEKCLILK	1.93
DHKPENPIRTFKDL	2.38
HLPPPPPPRAQQQQ	3.81
CGFPLLPSKRGVTA	3.62
GNIPVFPAKFGIQF	3.24
EEKPLLPTRPNKAE	4.67
GSEPIDPSKLEFAR	1.73
RKRPAPPKKKAPST	2.92
EVSPVEPKRTENER	1.86
VSDPYVPTRNTFNY	2.91
RHRPQKPVRTVIYT	3.52
VVSPSHPFKQLQKI	1.85
KPKPPKPIKYHFED	2.40
QQKPAIPQKKSFLT	3.59
PLPPIPPTKSKTSP	2.70
LSSPCLPPKVNATN	3.51
SDKPLTPTKTSSTL	2.18
QKTPPSPAKPKQKT	2.56
RVLPSKPKKSVKEN	3.42
PMSPGPPSKKDSRH	3.00
GIKPQQPIKVDIKG	2.01
SMTPDSPVKSDLTS	2.61
RTEPSTPSRPVPPD	3.84
KNFPTLPSKINSRH	4.00
FVLPCLPRKNTYKV	3.63
VTVPKLPFKLAVNK	3.53
PAFPEYPMRRDTLY	3.70
LKRPILPSKNMDHT	3.79
DSLPKLPFRSWGQP	4.59
ELEPQHPLRHKSSL	2.04
EMIPSSPQRKSVSN	3.33
LTLPFGPVKSIWLS	1.56
KLPPRLPHRAAQRR	4.49
IMPPPKPFRHGLDS	4.49
CILPAFPCKSSNEQ	2.64
AGHPMKPHRIRMAH	3.81
QAHPPKPKRREGDI	3.97
QRQPKRPKRYSLLL	3.73
KKCPRLPHRNKKVT	3.85
FCHPLSPLKSNFID	2.34
YSNPQTPLRSLAGL	3.43
PSAPKVPFKTLDSF	2.45
KYGPLPPSRACVGS	3.30
QTYPAQPPKIGNAF	2.07
SVLPQTPLKDVMGG	2.25
KNLPTIPIRLCGID	4.03
LGAPFCPRRKDKLV	3.18
FGTPVAPTRTLSRK	3.71
IEVPQSPTKNRVKV	2.25
FYEPLDPQKDKDMT	2.08
SHQPYLPIKNKNIV	3.13
LDHPNIPWKLIISG	0.90
WKPPDLPIRLRKRP	4.68
LVQPNVPEKHNMST	1.98
KFHPPEPYKGKGIY	1.66
SPLPVLPRRISTHT	4.55
NYIPPDPPKDIQLN	2.40
QFVPIAPQRLNMIF	3.20
NSPPVYPKRIRHFE	4.36
VPRPCPPIRRKSST	4.36
YSLPMHPGRWWVHT	2.85
VMVPHLPGKILLSN	2.62
NPPPVPPPRNVTAM	4.15
DLPPEPPTKLSDSF	1.75
KPDPECPSKLRHYE	1.62
PVGPKVPAKEPSPK	2.58
KIMPPTPAKKVEFI	3.48
RAVPILPPRNNVEP	4.58
CNEPHLPPKWVQRS	3.44
ITSPPLPPRADCIE	4.59
AMTPFSPRKSHINN	3.27
EIAPLIPKKPALRK	3.71
QAAPRNPNKDATIA	0.91
AFVPTNPWRSSMSL	2.92
VETPPKPIKKDVRF	2.83
EESPVKPLKITQKA	2.46
QMNPKNPIRLGMAN	2.94
HSTPVTPRRLFTSQ	4.22
ELPPTHPIRLGLAL	2.25
DERPFRPKRTSRSA	3.87
GAPPTTPSRPRTTT	4.14
GRKPKDPSKPRQML	2.43
RVHPEPPNKPGITT	2.43

VLTVKMSPSLPDEI	1.31
GTRPKILPKIPTGA	1.50
WSQMRMNPTNPDWI	3.10
DNELKDEPEPPNLI	1.21
KAALKYHPDKPTGD	1.27
NFPQRMMPPPPGLV	1.98
LAYERPLPDLPSTI	3.93
MKAYRANPPLPIDS	2.79
PFIRRYTPLKPITK	1.96
YAVGRIVPDSPTYD	2.41
FSTKKSKPIRPFVF	1.82
IESLREIPYGPDFD	0.28
QHKPKVKPSKPEAK	0.99
KTKKKTVPVKPLQL	1.58
TELLKTLPFYPTPS	1.03
IGKSKSAPPIPEDT	1.10
KKNKKKVPLNPLAK	2.25
MTQPKKNPMEPKTD	1.78
ITDTKLFPGNPNLM	1.19
TSCTKEKPTPPTTT	1.47
AGHIKTHPNSPTPG	1.16
KIVRRDYPSTPVIV	1.24
ETSLRVLPIAPKIF	1.74
SKRMKALPKGPAAL	2.05
ERDGRGVPLGPRNH	1.70
LWRPRIVPILPYIT	2.52
AKSNKVSPEDPAVQ	1.10
DLDWRKIPFLPKEI	2.19
GYDEKGNPIPPNNW	0.97
KQLKKQGPKRPSSA	2.04
SENGRLTPGLPRSY	1.72
LTSVKIVPQRPISD	1.54
TIEARVHPEPPNKP	0.00
LDSMKNLPLNPSKK	1.90
SLISKRNPCYPYGI	1.00
LYREREEPPLPKRI	4.21
SLKNRDGPITPSTI	1.15
LPKLKSNPSVPHLH	0.72
KGVLKEYPMRPYGS	2.13
SRSKRSKPLPPQLL	1.02
DGKLKHIPRRPYEI	1.69
MPNLKRLPIPPLQD	1.92
LSGTRLVPNCPLLW	1.90
PLRERIIPILPANS	1.93
ALWAKFFPEKPLNI	1.88
NAPERAVPILPPRN	4.56
SKVNKVAPDHPFIQ	0.85
NSGEKIYPQEPPIS	1.22
SKYAKLDPSTPVSL	1.69
SVDPKNYPDAPLEG	1.66
LQYHKKNPYSPLFS	1.90
MTVPRNRPMAPFGT	2.33
FANIKREPVHPEIR	2.06
TGKPRKTPRPPFPF	3.07
NAQLRKTPLEPGLE	1.90
MSWCKERPECPLCR	2.23
PKKSRVLPPLPFPL	3.56
SEAKRKFPEAPEVP	1.29
PYMYKYLPRFPNLK	2.64
LVPSKAIPPLPQLD	1.41
FHDPKFIPNKPYRQ	2.05
EFDEKLPPKKPAGP	1.21
DTYARNNPDFPPQN	1.59
MAEFRRVPLPPMAE	3.86
TQSGRMGPSEPKHR	2.04
TYLTRPLPSTPNED	4.67
TLDEKECPSVPPTV	1.76
PSRSKSNPILPIMR	1.85
EEQVKEKPNEPEGK	1.37
INNTRLSPNSPTPP	0.85
LIWHKNKPTKPDNK	1.85
SVNIKCWPLKPSNF	1.35
KSYRKADPTNPQGW	0.98
NLYQRTTPAGPDPS	3.06
INVEREAPMFPVPY	1.29
ENTPRPNPTQPRKP	1.74
IMDAKFHPFKPQLF	1.93
TKAKKEKPKPPKPI	1.33
SRVTRKRPREPKST	1.69
ILFERILPILPVES	3.17
RFSSRRAPIAPSGK	2.26
RRLKKNLPSLPTPV	1.70
KTVDKGLPMIPTYV	1.30
GLVLRSTPVIPDMK	2.48
KNIIKYVPWGPPLE	1.42
YSTNRGIPARPCKD	3.71
FFYRRDIPDVPITT	2.56
KYIIKTEPESPEED	1.53
HNAAKPIPHVPQAS	1.77
NKIPKHLPADPIFS	1.96
VTSVKLHPNLPIVF	0.91
TYGFRVRPDFPVFG	2.13
KISPSAPSKDSFTK	1.97
KTKPTPPPKPSHLK	2.37
CILPSTPTRPLSQS	4.31
HSEPAIPYRETFLS	2.80
LRSPNTPRRLRKTL	3.51
TSSPPLPPRQNVAT	4.67
RGTPANPGRVATLI	2.30
QGLPASPSRAALRY	2.02
RIPPTVPSKIIDVV	2.20
VFLPTAPSKHMDFA	1.65
IVNPFIPRRPYSNV	3.22
PFIPKFPSRESVES	3.15
KKPPKIPKKVYTLE	2.05
NVQPRKPSRVHSRN	3.72
FLPPPKPFRHTETK	4.40
SASPYRPQRLPAKF	2.73
LQIPPLPLKLLTPV	2.29
EVTPEEPSKSIDNK	2.02
IFGPFGPMKEVKIL	2.41
RERPLEPPRKPMAS	3.26
IFNPQNPLKTGFEP	2.09
CNPPTDPGRIPMSE	2.41
DVPPQLPTRTKSGE	4.86
APLPPQPRKHDKTP	2.57
SPFPSAPLRKLEIL	2.31
LKYPYFPTKMSYLQ	2.26
VNTPREPVRSSSKI	2.00
EIPPPLPPKIMVHS	4.16
NANPFLPNKHPVTQ	2.55
TSKPSLPEKPQKLR	2.50
GIHPGFPIRMVQYA	2.19
ERDPNMPKRPTNAY	2.53
QGPPNLPARDKSLV	4.81
TDSPQLPSKDKSQE	1.98
RIVPAIPKKATVDE	3.52
MAAPPVPPKQSQED	2.45
AMPPTLPHRDWKDY	4.41
VILPQIPPKLTFIL	2.20
KKDPNAPKRRLSAY	2.46
LNSPDLPERTKLRN	4.77
DPHPEDPRRIYRIY	1.88
GVFPNSPVKQTTII	2.06
EVTPEEPSKKIRTS	2.09
ENYPCPPYKIIILD	1.94
SGKPKKPYRRWTPE	2.61
KKLPTGPSKKRQRK	1.93
ARDPILPYRLVKDR	3.63
PPLPPIPTRDDMSN	2.74
AKSPLLPFKLLSDR	2.36
QGQPLPPPRGQVIL	4.20
TPLPFAPNKSSKKT	2.00
PNYPDSPPKVTFIS	2.42
EFIPPDPTKVTEFE	1.73
DFRPRMPKRQRIPP	2.32
NSLPCSPKKQPARN	2.30
LMPPDSPPRMTENT	2.69
LVIPSTPSKKCKYS	1.98
EAAPQLPSRSSAAP	4.97
PQLPKSPSRYSLSR	2.59
ENKPFKPPRRVGSN	3.92
AKQPKEPKKRTTRR	1.89
LCIPDLPLRIHNQT	4.49
AAQPPLPSRNVASG	5.03
NLDPNPPVRGENLT	2.50
SSFPTIPLRASSGA	2.89
SAIPITPTKRIRRN	1.50
LPPPIIPLRKYGHN	4.27
LPVPPPPVRPSISF	3.53
NNAPTLPKRKNPKI	4.66
IIGPDEPEKCLILK	1.80
DHKPENPIRTFKDL	2.24
HLPPPPPPRAQQQQ	3.21
CGFPLLPSKRGVTA	2.50
GNIPVFPAKFGIQF	2.33
EEKPLLPTRPNKAE	4.80
GYDPTDPSRPRINE	2.41
GSEPIDPSKLEFAR	1.58
RKRPAPPKKKAPST	1.66
EVSPVEPKRTENER	1.92
VSDPYVPTRNTFNY	2.43
RHRPQKPVRTVIYT	1.96
VVSPSHPFKQLQKI	2.24
KPKPPKPIKYHFED	2.10
QQKPAIPQKKSFLT	2.00
PLPPIPPTKSKTSP	2.15
LSSPCLPPKVNATN	1.96
SDKPLTPTKTSSTL	2.58
QKTPPSPAKPKQKT	2.24
RVLPSKPKKSVKEN	2.29
PMSPGPPSKKDSRH	2.25
GIKPQQPIKVDIKG	2.19
SMTPDSPVKSDLTS	2.73
RTEPSTPSRPVPPD	2.64
KNFPTLPSKINSRH	2.18
FVLPCLPRKNTYKV	1.88
AVDPENPKKLASKF	1.63
TNYPVNPEKHEMDK	2.13
VTVPKLPFKLAVNK	2.36
PAFPEYPMRRDTLY	3.28
LKRPILPSKNMDHT	2.40
DSLPKLPFRSWGQP	4.78

CRFYRPPRCS	3.75
SSPPRPVRHG	3.47
DLLPRPPRLL	2.92
IPFERPFRRP	2.98
FLINRPGKVK	2.31
VDPYRPVRLP	2.51
GELGRPGRKG	2.01
GKPSRPSKPI	2.10
KYLSRPKRFE	2.29
GRPTRPCRGP	3.98
PALPRPSRSS	2.89
LLLPRPGRPL	1.99
DDPRRPSRSP	3.34
QELRRPSKSM	1.69
MAFPRPKRPA	3.12
PSPARPKKSE	2.01
IRPPRPPKVL	3.95
TAPARPLRLP	4.05
CFIGRPWRVV	2.05
RRLDRPTRIR	2.89
RPLARPIKPN	1.96
RYLGRPKKEK	0.85
EELARPPREQ	2.19
SRFQRPGRRM	3.61
SSLQRPWRVP	2.70
ALLKRPLREI	2.94
KGLGRPGKGH	2.32
SLPIRPSRAP	4.33
SRPPRPSRPP	3.90
LRLCRPYRLA	3.22
PPPKRPVRAS	4.15
QELRRPAQLR	1.68
LEPRRPPHDY	1.52
FSLRRPLQTA	1.81
RVLRRPQEFV	1.86
PQIRRPRSLS	2.05
LLIRRPECFG	1.60
VDPKRPLMPE	1.43
ENFRRPQTDQ	1.31
ASLRRPKRII	2.87
TSPRRPLILK	2.18
SGLRRPEKPP	1.76
EHPKRPVLGL	1.31
LEPRRPRSES	1.47
EKPKRPEDES	1.48
DDLRRPATMK	1.48
PHIKRPMNAF	1.51
NFLRRPQRMP	3.09
HSPRRPRPLG	1.65
DWLRRPLGED	1.64
PVLRRPPILE	1.46
SHLKRPQQGA	1.42
ASLKRPDSGA	1.43
RTLKRPTVLE	1.47
KQPKRPRSES	1.71
PGLRRPLSPP	1.76
ESLKRPWEDD	1.77
GGFKRPLAPS	1.50
AQLRRPRHSH	1.73
NILRRPIIVI	1.41
SILKRPSSAI	1.43
ERPRRPNGDI	3.57
SGPKRPQDKV	1.70
KLPRRPQPGP	1.17
ETLRRPGGPQ	1.39
HSPRRPYASP	1.85
APPPRPPKPS	3.48
IHIKRPDDRA	1.32
DAPKRPKEAA	1.29
WLPRRPARSV	3.92
FSLKRPRGLS	1.60
KYIKRPGAVL	1.43
MVIKRPGRKR	3.21
LDLKRPFLSR	1.67
NTLKRPKRPP	2.61
SKLKRPTPTF	1.62
PPPKRPRIAY	1.59
TLPRRPFLHC	3.18
GTLRRPEGPA	1.39
GEPRRPPCDP	1.31
ARLRRPPRPS	3.44
DQLKRPFGAF	1.31
LLLRRPPGRE	1.20
PALRRPGRFD	2.82
FVIKRPELLT	1.46
WQPKRPAVPK	1.24
PVLKRPRHVH	1.67
AELRRPPPAP	1.42
GKFKRPIIAG	1.63
KSPKRPASPS	1.51
PWIRRPFFPF	1.25
ELLRRPMDSL	1.48
PTPKRPRGRP	1.56
EPPRRPAFLP	1.51
GPPKRPGRPP	3.71
FIIKRPFLEP	1.63
LLFKRPFGTC	1.61
DIPKRPFSQC	2.00
MTFRRPAQAF	1.43
EKLRRPEGAS	1.61
HRPRRPRGAL	1.43
GEPRRPAEIY	1.28
CVLRRPGANH	1.66
SKLRRPLKRN	2.04
EPPKRPTSRL	1.56
PLPKRPALEK	2.20
TIPKRPWLSA	1.55
TLPRRPYKVL	2.04
TDIRRPRLYL	1.56
PTPRRPASVN	2.23
NDLKRPMQAP	1.52
TPPRRPVPVA	2.50
NSLKRPDTTE	1.39
LKLRRPVVGS	1.52
GPLKRPIVEF	1.43
RLPRRPNDIY	1.59
PSLRRPSREL	2.65

LQAELRRPPPAPAQ	1.42
AKMPLRRPKMKKTP	1.54
REAGFKRPVVLFGP	1.39
SAGKFKRPIIAGME	1.63
KLKSPKRPASPSSP	1.51
TWQGLRRPPSVLGH	1.81
RTKRFKRPQFSPTG	1.53
DADPLKRPTFKQIV	1.60
HHPWIRRPFFPFHS	1.25
QSISLRRPRLASQS	1.75
TSFEPRRPPPLRPG	1.51
FTKTIKRPFGVKYN	1.35
VEELLRRPMDSLDS	1.48
RGIQFKRPHTVAPW	1.55
EHREPKRPEIIFLP	1.49
GWIQPRRPRDPTPS	1.89
ANLQPKRPKTIKQR	1.37
LGGSPRRPCGRKGS	1.54
CPCAPRRPRRPRRP	3.69
YVDVIKRPLVKNPV	1.42
GPAGPKRPVFILGE	0.98
GEGMFRRPSEGQSL	1.64
VQPQLRRPFELLIA	1.53
EVPTPKRPRGRPKG	1.56
ILPDFKRPACFGTY	1.49
PPAPLRRPLAVLHA	2.50
NSRNPRRPPPSSVN	1.90
RFALFKRPKANGVK	1.28
ALQPPRRPKSSNPF	2.15
HAPRIKRPRLQVVK	1.56
PREPPRRPAFLPVG	1.51
IIQELRRPKYTIYF	1.51
GSPEPKRPGVSEAA	1.42
SEDPLKRPVVYVKG	1.33
ISFIIKRPFLEPKK	1.63
LTLLFKRPFGTCDP	1.61
MKDIPKRPFSQCLS	2.00
SRNIFKRPLGSRIE	1.48
AFPRPKRPAPAQEA	2.16
AEMTFRRPAQAFPV	1.43
FEGSLRRPLSWKSL	1.64
SGPDLRRPPTPAPW	1.60
PEIFLKRPVVEGNR	1.51
QVEKLRRPEGASNE	1.61
EGIPFRRPSTYGIP	1.63
DPNAPKRPPSAFFL	1.60
DLSGLKRPSSSAST	1.50
KYSAPRRPTATGDY	1.67
PGPAPKRPRGGGAG	1.59
MWGFLKRPVVVTAD	1.58
KSKKFRRPDIQYPD	1.39
EGHRPRRPRGALPP	1.43
LPHWLRRPVYSLYI	1.70
LPGEPRRPAEIYHC	1.28
PSQVPRRPHHTPFQ	1.63
KSLPLRRPSYTLGM	1.71
SASGIKRPMASEVP	1.25
EKDHIRRPMNAFMI	1.48
PICVLRRPGANHEG	1.66
VPGDPRRPYPTDLE	1.08
LFNLIKRPDDEQRQ	1.21
GWGSPRRPTEAEAR	1.37
DPRRPKRPRTILTT	2.26
LYLRLKRPKPERDL	1.63
RPRRPRRPGPSDPA	2.77
SVEPPKRPTSRLIM	1.56
LQRRLKRPETPTHD	1.46
LPPLPKRPALEKTN	2.20
KGTIPKRPWLSATP	1.55
SPRLPRRPTVESHH	3.11
PKTYLRRPEASSPA	1.33
PPASPRRPRSFKSK	1.76
EGTYPRRPGSSPKV	1.74
FYRDLRRPFSAPDA	1.73
YHPNPKRPGLDREQ	1.64
SVTDIRRPRLYLLQ	1.56
DELEPRRPRYRVPD	1.55
LTVAIRRPPDLGGP	1.37
PVEAIKRPSPDEPM	1.63
QGPAPRRPTTDQIS	1.56
APRRPRRPRRPGPS	3.38
APRAPRRPRSPPSS	1.97
PPPTPRRPASVNGS	2.23
GLSGLKRPSSSAST	1.51
LRPVPRRPSTWSRR	3.38
SPQEPKRPSQLPSP	1.72
KRKKFKRPPQRLFD	1.76
GRNDLKRPMQAPPG	1.52
PKTPPRRPVPVADC	2.50
DTKALRRPKCAELH	1.41
SGLRLRRPRVSSTE	1.83
RSKSPRRPSPTARG	1.62
IQFVIKRPELLTEG	1.45
SGNSLKRPDTTESL	1.39
ARSYLRRPAVVYIG	1.55
PVLCPRRPWPSLCW	2.00
GFGVLRRPRPTPGD	1.58
SAKELRRPPPRSPQ	1.53
LQPRPRRPREAPDT	2.61
AEGPPKRPGGSSPL	1.70
DPRTPRRPRSARAG	1.77
LATQPKRPMSIPII	1.37
VLLKLRRPVVGSSE	1.52
IFGPLKRPIVEFSL	1.43
SPIKPKRPFVESNV	2.08
PSRLPRRPNDIYVN	1.59
LAITLRRPGCELEA	1.20
KQVSPRRPQATEAV	2.17
QLWDIRRPDRCERM	3.36
SGGRLRRPVNVPLD	1.53
LSLLLKRPWDPAVG	1.48
PSFHIKRPAPRPLA	1.26
STSFPRRPLAVEGG	1.72
PKSVLKRPVTSEEL	1.58
NLSLLRRPGEKTYT	1.47
GTYQLRRPEDRPAG	1.16
ICPEFKRPRDIAVD	1.48
YRLPPTRPPASVSP	2.17
PQQFPSRPPPPQPK	2.51
APALPPKPPKPTTV	2.30
WGALPPRPPLLLLL	1.63
LSPRPEKPPEEPGQ	1.66
LFIIPPKPPGRPQG	2.66
GRTPPARPPQDPAE	1.84
VCARPEKPPGQVTR	1.63
LDPLPRRPPAGRRG	3.04
GGASPGRPPQPEQP	1.58
PEAVPGKPPFAEPD	1.85
PRLRPMRPPPPPPA	3.27
SFQSPTRPPLASPT	1.44
AGSSPGKPPRLVGG	2.43
CGPPPLRPPLPAAA	1.77
PRPGPCRPPPVPAP	2.14
ATDLPKKPPPPEVT	1.83
PPWVPWRPPSSAAV	2.92
SCTPPPRPPVLEEW	1.47
QRQRPPRPPPRGTP	3.94
AETRPHRPPGARDS	2.34
GPGKPERPPPQGGD	1.53
RHPKPLKPPWHLSM	1.88
CAGLPGKPPAIQAC	1.63
TTPPPGRPPAPSSE	1.39
RPPPPAKPPPDEEE	1.51
CARGPLRPPVRCPA	3.58
SNTPPPRPPKPSHL	3.33
FLLLPPRPPPARPE	1.63
KAGRPSKPPSPKQN	1.45
MHNFPLRPPPPPPP	1.84
VRPPPARPPSGPGL	2.98
TEGIPPKPPPRLVW	3.44
YDTFPEKPPPSPGD	1.52
PPHLPARPPGGWSR	3.11
LPPKPARPPAVSPA	2.33
SPTVPIKPPCIPNG	1.82
TQSLPTRPPTFPPT	1.90
MVPGPARPPGSCWD	1.66
RAEVPTKPPLPPAR	3.91
SQQPPSRPPHLPVE	1.65
PTTRPTRPPPPLIP	3.18
EYHRPAKPPPPPTG	1.64
GPAPPPRPPQLERH	3.00
SRSPPKKPPKRTSS	2.37
PTRPPSRPPSRPQS	3.85
VNEGPPKPPTELEP	1.30
LGKIPLKPPPLELL	1.86
PSPGPCRPPHGTQA	1.67
LPPLPHRPPQNPFV	2.42
RQPRPPRPPLGDRP	3.17
RCCFPVRPPTASTI	2.22
RAWIPGKPPCPPRG	3.29
LEIPPPKPPAPEMN	1.50
APLLPPRPPPLPPV	3.15
AGNLPSRPPPLSSP	1.78
LVPVPARPPLTRGQ	3.47
DLHPPPRPPSPTEI	1.81
EMTPPPKPPRLYLP	3.26
MAGLPFRPPWGCLS	1.86
EYALPQRPPQGPED	1.59
SSHSPYRPPTNYRN	1.72
QLRPPPKPPLPAPD	2.92
EPGLPVRPPGGGQS	2.57
KAVLPLRPPWPLWG	2.00
LPLPPPRPPLLSAR	3.36
FLGGPARPPSPSPS	1.91
SAPPPPRPPEEPAP	1.93
TACPPARPPAQVGL	1.54
RRQPPARPPGPSTD	3.17
PGPSPARPPAKPPE	1.28
LARIPNKPPPEYPG	1.48
VPQKPERPPLPPEP	2.33
ASSRPGRPPKRSLG	3.84
ALSLPEKPPRGEQG	2.86
SPCVPAKPPLAEFE	1.68

NYIPPDPPKDIQLN	2.26
QFVPIAPQRLNMIF	2.63
NSPPVYPKRIRHFE	3.94
VPRPCPPIRRKSST	2.69
YSLPMHPGRWWVHT	1.90
VMVPHLPGKILLSN	2.40
NPPPVPPPRNVTAM	2.26
DLPPEPPTKLSDSF	1.88
KPDPECPSKLRHYE	1.84
GRHPDMPTRVKNAY	2.02
PVGPKVPAKEPSPK	1.94
KIMPPTPAKKVEFI	1.51
RAVPILPPRNNVEP	1.70
CNEPHLPPKWVQRS	2.08
ITSPPLPPRADCIE	2.26
AMTPFSPRKSHINN	2.81
EIAPLIPKKPALRK	2.04
QAAPRNPNKDATIA	1.89
AFVPTNPWRSSMSL	2.47
VETPPKPIKKDVRF	1.81
EESPVKPLKITQKA	1.78
QMNPKNPIRLGMAN	2.83
HSTPVTPRRLFTSQ	3.36
ELPPTHPIRLGLAL	1.89
DERPFRPKRTSRSA	2.80
GRKPKDPSKPRQML	2.17
RVHPEPPNKPGITT	2.09
KDHPYDPEKDSVLK	1.71
RFDPKSPTKRSIAI	2.11
NQAPLEPLKPTATG	2.06
VIRPGVPTKFTKKQ	2.08
NHIPPFPLRFSFAQ	2.08
TEPPMNPLKNREKM	1.94
QGEPLDPKRPSTAS	1.72
DMVPKHPTKRYVPK	2.20
KSVPIPPRKNATLD	3.06
NKVPTNPKKFKKAP	2.10
IAAPKKPRKPRQTK	1.70
SSQPRPPPRPQQNP	1.84
QYIPPPPPKYINSK	2.08
TTAPRGPKKAQPAQ	2.14
DKLPFSPIKEKKLK	2.67
SMSPLPPARANKSD	2.08
ANLPNRPKRGEGIN	2.16
VTTPLKPKRLAIPI	2.09
AGSPIRPYKQRYLL	2.87
MLIPNFPNKKLKLN	1.54
DSLPFAPLRKVYEL	1.79
SNVPTTPSRELNGT	1.52
KERPKPPNKGDVGR	2.23
PIQPQEPFRHNTTI	2.02
FREPDLPRKGLLED	2.12
SSLPSPPAKKSATS	1.82
DQKPLVPIRIYGAI	2.26
SLPPTPPYKAHKKK	2.06
SSTPTTPERPKRKS	2.91
EGKPLFPGKDHVHQ	1.95
YMQPIIPGKTECFE	1.80
VHGPVIPMKALEIA	1.75
FWNPENPYKFVHPR	2.23
MEYPFKPPKMQFDT	1.75
FPYPFKPSRVGVKY	2.25
FVIPGVPSKCYSLF	1.97
RPSPWLPSKPNCYH	2.01
LVQPVVPDKVFSFR	2.09
VHGPGVPVKTLEIA	2.06
IYTPYIPFRNSRTW	3.74
YKIPVVPPRETYFA	2.14
FYYPESPTKNLRPR	2.94
NEGPEYPTKIEKNQ	2.10
VTQPITPVKKRRSE	2.65
EDTPDLPQKTGIRK	1.39
NWSPNIPLRYSDFA	3.03
TTRPAPPSKAKDVA	1.79
SASPTLPTRRSRRL	4.67
WNLPWSPFKITTIM	2.16
FQPPPKPFRRSQSS	2.90
LRKPRAPMRNVIIT	2.29
IAYPGDPLRFHSHL	1.08
LWQPVDPSREDLVI	1.69
IIIPPIPSKHSLLK	1.97
DKLPEGPVKSLEGA	1.55
HNSPVAPNRYAANL	2.20
CREPEVPQRLLTLI	1.78
NLKPERPSKVSFEM	2.01
FKAPTNPRKVYCKR	1.91
MVRPAPPRRADPEG	3.56
MVTPSKPPKKSVGF	2.17
DRPPDVPRKIIVGR	1.98
FLQPVDPVKLDIPF	1.97
RAPPPVPKKPSSRI	2.11
LGEPFDPNKHEATF	1.99
NIQPDLPPRKIMIP	3.18
MTDPDAPSRTEHKW	2.09
LDDPFTPPRWLHHI	2.63
VMKPKSPDKEFFDA	1.95
IRYPVTPNKASLDR	1.94
KNSPLAPIKKNVEL	2.26
MKAPKMPDKHRDNY	2.21
SGKPLFPGRDYHHQ	1.53
PVDPTTPVKLGPGI	1.61
TNEPTKPIRRLSTP	2.05
FFDPESPNKGKLIL	1.80
LDRPQLPPRQVVNA	2.16
ALVPFAPFKLLKDR	1.22
FDFPLSPVKHENPT	1.85
KDAPIEPPKYMGMG	2.08
QSTPTPPAKRLFLI	1.76
ELLPVVPEKFLIAC	1.96
NYAPRLPRRETSSS	3.59
FVPPNLPMRRFKEI	4.04
FFQPEYPVRMEPDL	1.76
DVIPKKPLKSAADK	2.15
GAIPRGPKKDDASF	1.97
HSSPIAPSKAKRQR	2.83
AVLPIEPMKIKGTT	1.99
MSEPEVPFKVVAQF	1.87
RMIPELPPKRIGSQ	2.56
LRSPQNPNRMTAVI	1.75
PSNPALPKKLLNVP	1.46
LRLPEIPPKGDFDV	0.18
HQIPSAPQRLVSII	1.94
YRYPQTPMRLEIAV	2.32
GGPPLLPQKSAAFQ	2.11
KYDPDNPNKDKLGT	2.16
DELPSPPLRMVHID	2.11
DSIPLEPKKAKHLM	2.59
QSQPPRPPRPAANL	2.76
LAPPLSPTKPSPLH	1.91
TLRPGIPLKPMLAK	2.02
EEHPPLPARRKSEE	2.79
YRIPDDPRKSQNKS	1.98
WSYPYEPLRVLQVG	2.51
ACLPVGPRKNGMNV	2.20
EYFPISPEKKEEME	2.08
VYSPIQPRRRKDSS	2.76
LLAPNSPLKNTKSV	1.76
TKKPRPPVKSKPKH	1.79
TPPPVEPLKTKDLA	1.97
DQAPSLPPKPNTQL	1.74
PLIPDNPGRLLSSK	1.98
NNGPKIPQKNTIDS	1.65
NSPPTTPSKDECGP	1.64
MVLPPAPPKNSDPS	1.73
SKVPKAPTRETHIP	1.37
LSCPNVPGKPQVAY	1.83
FHDPILPRRTSTFL	3.99
VGNPEGPEKPNKKF	2.41
LPAPPPPPRRGPAP	3.47
SPSPLNPYRPHHNM	1.80
NIPPNSPGKLQLNT	1.57
GVYPSRPEKTYDYS	2.05
GGQPPVPVRMQPQP	1.93
GPAPPPPPRASRPT	4.01
LSLPRLPNKHHWPL	2.29
PPPPPPPPKPPLND	1.78
RGIPARPCKDDVMK	1.74
GPPPAMPARPTATT	1.94
PYPPSLPSRDLYEV	1.56
LPPPRSPNRPTLSD	4.73
VDPPFMPFRDAGYS	1.85
VKEPSFPSRSAFSP	1.65
IAYPFYPFKKLGRQ	1.88
WISPDTPLKFPLNE	1.93
ISTPEVPKKKIKTG	2.44
QTSPGQPRKGQNGS	2.20
WNDPSDPTKGFQYL	1.48
IAHPVRPHRAITMT	3.52
SPSPSTPTKSGKMR	2.44
GLEPNTPLKSIKVD	1.74
GNVPHSPVRFADTV	2.06
ASMPRVPLKIVLKD	2.01
PPVPKAPSRRSSSL	3.09
TDGPIDPFKEPKTS	2.14
SNSPISPHKLIYKN	1.85
RNEPVKPFRNIDLK	2.16
KLEPENPQKPSNTH	1.88
HLDPEAPDKLLENS	2.21
VSPPFFPEKELNNR	1.61
PNSPTPPLKSKVSR	2.23
VCPPSFPSRRCETC	2.85
TRRPTNPFRVISVS	1.54
IINPDTPHRRIFNQ	2.50
EYSPLVPSKMCADK	2.01
FKMPKKPQRSKEEV	1.99
TGRPDKPFRALLVD	2.04
IIFPTSPTKLTFSN	1.66
PIMPTLPPRPYITI	1.50
RLIPTSPQKTNINT	1.72
ATRPSSPMRHKSIS	2.22
EILPVKPTKKSSKK	2.56
FRFPRLPQKKKEEL	2.02
LRIPETPVKKSPLV	1.83

ELPPTHPIRLGLAL	1.66
DERPFRPKRTSRSA	2.23
GAPPTTPSRPRTTT	1.89
GRKPKDPSKPRQML	1.63
RVHPEPPNKPGITT	1.88
KDHPYDPEKDSVLK	0.57
RFDPKSPTKRSIAI	1.79
NQAPLEPLKPTATG	1.16
VIRPGVPTKFTKKQ	1.80
NHIPPFPLRFSFAQ	1.55
TEPPMNPLKNREKM	1.74
QGEPLDPKRPSTAS	1.36
DMVPKHPTKRYVPK	2.06
KSVPIPPRKNATLD	2.16
NKVPTNPKKFKKAP	1.85
IAAPKKPRKPRQTK	1.73
SSQPRPPPRPQQNP	0.76
QYIPPPPPKYINSK	1.66
TTAPRGPKKAQPAQ	1.64
DKLPFSPIKEKKLK	1.60
SMSPLPPARANKSD	2.66
ANLPNRPKRGEGIN	1.45
VTTPLKPKRLAIPI	1.31
AGSPIRPYKQRYLL	2.55
MLIPNFPNKKLKLN	1.78
DSLPFAPLRKVYEL	1.54
SNVPTTPSRELNGT	1.85
KERPKPPNKGDVGR	1.38
PIQPQEPFRHNTTI	0.90
FREPDLPRKGLLED	1.62
SSLPSPPAKKSATS	1.22
DQKPLVPIRIYGAI	1.95
SLPPTPPYKAHKKK	1.36
SSTPTTPERPKRKS	1.57
EGKPLFPGKDHVHQ	1.04
YMQPIIPGKTECFE	2.41
VHGPVIPMKALEIA	1.44
FWNPENPYKFVHPR	1.75
MEYPFKPPKMQFDT	1.74
FPYPFKPSRVGVKY	1.91
FVIPGVPSKCYSLF	1.68
RPSPWLPSKPNCYH	1.57
LVQPVVPDKVFSFR	1.72
VHGPGVPVKTLEIA	1.75
IYTPYIPFRNSRTW	3.16
YKIPVVPPRETYFA	1.42
FYYPESPTKNLRPR	1.64
NEGPEYPTKIEKNQ	1.53
VTQPITPVKKRRSE	1.95
EDTPDLPQKTGIRK	1.26
NWSPNIPLRYSDFA	2.76
TTRPAPPSKAKDVA	1.86
SASPTLPTRRSRRL	4.00
WNLPWSPFKITTIM	3.43
FQPPPKPFRRSQSS	2.49
LRKPRAPMRNVIIT	1.96
IAYPGDPLRFHSHL	2.16
LWQPVDPSREDLVI	1.71
IIIPPIPSKHSLLK	1.70
DKLPEGPVKSLEGA	1.87
HNSPVAPNRYAANL	1.53
CREPEVPQRLLTLI	1.83
NLKPERPSKVSFEM	1.72
FKAPTNPRKVYCKR	1.59
MVRPAPPRRADPEG	3.12
MVTPSKPPKKSVGF	1.93
DRPPDVPRKIIVGR	1.79
FLQPVDPVKLDIPF	1.39
RAPPPVPKKPSSRI	1.62
LGEPFDPNKHEATF	1.87
NIQPDLPPRKIMIP	2.04
MTDPDAPSRTEHKW	1.54
LDDPFTPPRWLHHI	1.59
VMKPKSPDKEFFDA	2.13
IRYPVTPNKASLDR	2.22
KNSPLAPIKKNVEL	1.53
MKAPKMPDKHRDNY	2.21
SGKPLFPGRDYHHQ	1.74
PVDPTTPVKLGPGI	1.33
FFDPESPNKGKLIL	2.17
LDRPQLPPRQVVNA	1.50
ALVPFAPFKLLKDR	1.61
FDFPLSPVKHENPT	0.38
KDAPIEPPKYMGMG	1.87
QSTPTPPAKRLFLI	1.69
ELLPVVPEKFLIAC	1.13
NYAPRLPRRETSSS	3.67
FVPPNLPMRRFKEI	2.20
FFQPEYPVRMEPDL	1.88
DVIPKKPLKSAADK	1.56
GAIPRGPKKDDASF	0.76
HSSPIAPSKAKRQR	2.23
AVLPIEPMKIKGTT	1.57
MSEPEVPFKVVAQF	1.81
RMIPELPPKRIGSQ	2.30
LRSPQNPNRMTAVI	1.69
PSNPALPKKLLNVP	1.90
LRLPEIPPKGDFDV	1.89
HQIPSAPQRLVSII	1.27
YRYPQTPMRLEIAV	2.16
GGPPLLPQKSAAFQ	0.30
KYDPDNPNKDKLGT	1.82
DELPSPPLRMVHID	1.45
DSIPLEPKKAKHLM	1.42
QSQPPRPPRPAANL	1.20
LAPPLSPTKPSPLH	0.81
TLRPGIPLKPMLAK	1.70
EEHPPLPARRKSEE	1.73
YRIPDDPRKSQNKS	1.86
WSYPYEPLRVLQVG	3.48
ACLPVGPRKNGMNV	1.94
EYFPISPEKKEEME	1.18
VYSPIQPRRRKDSS	3.07
LLAPNSPLKNTKSV	1.01
TKKPRPPVKSKPKH	1.40
TPPPVEPLKTKDLA	1.64
DQAPSLPPKPNTQL	1.86
PLIPDNPGRLLSSK	1.69
NNGPKIPQKNTIDS	1.44
NSPPTTPSKDECGP	1.48
MVLPPAPPKNSDPS	1.54
SKVPKAPTRETHIP	1.56
LSCPNVPGKPQVAY	1.63
FHDPILPRRTSTFL	1.94
VGNPEGPEKPNKKF	1.70
LPAPPPPPRRGPAP	1.75
SPSPLNPYRPHHNM	1.73
NIPPNSPGKLQLNT	1.97
GVYPSRPEKTYDYS	1.67
GGQPPVPVRMQPQP	1.95
GPAPPPPPRASRPT	1.75
LSLPRLPNKHHWPL	1.65
PPPPPPPPKPPLND	1.76
RGIPARPCKDDVMK	1.17
GPPPAMPARPTATT	2.15
PYPPSLPSRDLYEV	1.49
LPPPRSPNRPTLSD	4.69
VDPPFMPFRDAGYS	1.58
VKEPSFPSRSAFSP	1.62
IAYPFYPFKKLGRQ	1.51
WISPDTPLKFPLNE	2.36
ISTPEVPKKKIKTG	1.38
QTSPGQPRKGQNGS	1.35
WNDPSDPTKGFQYL	1.67
IAHPVRPHRAITMT	2.16
SPSPSTPTKSGKMR	1.72
GLEPNTPLKSIKVD	1.77
GNVPHSPVRFADTV	1.47
ASMPRVPLKIVLKD	2.45
PPVPKAPSRRSSSL	2.88
TDGPIDPFKEPKTS	1.77
RNEPVKPFRNIDLK	1.67
KLEPENPQKPSNTH	1.96
HLDPEAPDKLLENS	1.80
VSPPFFPEKELNNR	1.41
PNSPTPPLKSKVSR	1.60
VCPPSFPSRRCETC	2.07
TRRPTNPFRVISVS	1.69
YYGPNSPLKDEEN	1.09
IINPDTPHRRIFNQ	1.86
EYSPLVPSKMCADK	1.75
FKMPKKPQRSKEEV	2.39
TGRPDKPFRALLVD	1.67
IIFPTSPTKLTFSN	1.14
PIMPTLPPRPYITI	1.94
RLIPTSPQKTNINT	1.48
ATRPSSPMRHKSIS	2.48
EILPVKPTKKSSKK	1.33
FRFPRLPQKKKEEL	2.24
LRIPETPVKKSPLV	1.62
RPNPTQPRKPLDCK	1.79
YTLPLLPGKGGFRI	1.31
LIPPLHPLKKQHPK	1.25
DLQPLEPKKAAAST	2.02
ILSPGYPNKSFNNK	1.84
ALRPTGPDRGDGFD	0.91
SNSPQIPKKKVKDQ	1.81
SNDPIPPPRKVSDV	1.12
QDSPDMPEKECVYV	1.63
TNPPIDPIREANVM	1.74
EYHPIKPLRSSSST	1.48
ISTPPPPPKMAIPA	1.54
ELPPIKPPRPTSTT	1.96
PIVPREPLRNEPTD	4.52
ITLPRRPAKKIQLG	2.03
KLKPSLPNKLVGKY	1.53
QPKPGNPLRNARLN	1.34
DVYPHEPAKNGEGS	1.58
LRKPSAPKRMSSIS	1.89
GKVPVQPRKRKSIT	1.93
ELDPICPLKVPLTA	1.66
ERDPNAPKKPLTVF	1.54
NDDPVIPYKLSKDT	1.65

LQAELRRPPPAPAQ	1.32
AKMPLRRPKMKKTP	2.30
REAGFKRPVVLFGP	1.38
SAGKFKRPIIAGME	1.66
KLKSPKRPASPSSP	2.90
TWQGLRRPPSVLGH	2.54
RTKRFKRPQFSPTG	1.10
DADPLKRPTFKQIV	1.30
HHPWIRRPFFPFHS	3.27
QSISLRRPRLASQS	2.32
TSFEPRRPPPLRPG	2.32
FTKTIKRPFGVKYN	1.79
VEELLRRPMDSLDS	1.66
RGIQFKRPHTVAPW	1.46
EHREPKRPEIIFLP	1.64
GWIQPRRPRDPTPS	2.92
ANLQPKRPKTIKQR	1.74
LGGSPRRPCGRKGS	2.08
CPCAPRRPRRPRRP	3.24
YVDVIKRPLVKNPV	1.47
GPAGPKRPVFILGE	2.41
GEGMFRRPSEGQSL	1.84
VQPQLRRPFELLIA	1.61
EVPTPKRPRGRPKG	1.62
ILPDFKRPACFGTY	1.36
PPAPLRRPLAVLHA	2.66
NSRNPRRPPPSSVN	2.67
RFALFKRPKANGVK	1.13
ALQPPRRPKSSNPF	4.35
HAPRIKRPRLQVVK	1.52
PREPPRRPAFLPVG	3.04
IIQELRRPKYTIYF	2.13
GSPEPKRPGVSEAA	1.25
SEDPLKRPVVYVKG	1.77
ISFIIKRPFLEPKK	2.11
LTLLFKRPFGTCDP	1.64
MKDIPKRPFSQCLS	3.45
SRNIFKRPLGSRIE	-0.05
AFPRPKRPAPAQEA	2.19
AEMTFRRPAQAFPV	2.06
FEGSLRRPLSWKSL	1.83
SGPDLRRPPTPAPW	1.59
PEIFLKRPVVEGNR	1.98
QVEKLRRPEGASNE	1.81
EGIPFRRPSTYGIP	1.61
DPNAPKRPPSAFFL	3.56
DLSGLKRPSSSAST	1.45
KYSAPRRPTATGDY	1.57
PGPAPKRPRGGGAG	2.54
MWGFLKRPVVVTAD	2.08
KSKKFRRPDIQYPD	1.61
EGHRPRRPRGALPP	3.31
LPHWLRRPVYSLYI	2.32
LPGEPRRPAEIYHC	1.65
PSQVPRRPHHTPFQ	3.22
KSLPLRRPSYTLGM	2.51
SASGIKRPMASEVP	1.91
EKDHIRRPMNAFMI	1.83
PICVLRRPGANHEG	1.96
VPGDPRRPYPTDLE	1.60
LFNLIKRPDDEQRQ	1.53
GWGSPRRPTEAEAR	2.29
DPRRPKRPRTILTT	2.98
LYLRLKRPKPERDL	1.85
RPRRPRRPGPSDPA	3.13
SVEPPKRPTSRLIM	2.68
LQRRLKRPETPTHD	1.62
LPPLPKRPALEKTN	3.36
KGTIPKRPWLSATP	2.71
SPRLPRRPTVESHH	3.68
PKTYLRRPEASSPA	1.78
PPASPRRPRSFKSK	2.43
EGTYPRRPGSSPKV	1.86
FYRDLRRPFSAPDA	2.24
YHPNPKRPGLDREQ	1.53
SVTDIRRPRLYLLQ	1.98
DELEPRRPRYRVPD	1.42
LTVAIRRPPDLGGP	1.25
PVEAIKRPSPDEPM	2.01
QGPAPRRPTTDQIS	3.82
APRRPRRPRRPGPS	3.18
APRAPRRPRSPPSS	2.41
PPPTPRRPASVNGS	3.01
GLSGLKRPSSSAST	1.54
LRPVPRRPSTWSRR	3.68
SPQEPKRPSQLPSP	1.52
KRKKFKRPPQRLFD	1.35
GRNDLKRPMQAPPG	1.60
PKTPPRRPVPVADC	1.79
DTKALRRPKCAELH	1.80
SGLRLRRPRVSSTE	1.65
RSKSPRRPSPTARG	2.08
IQFVIKRPELLTEG	1.47
SGNSLKRPDTTESL	1.28
ARSYLRRPAVVYIG	1.78
PVLCPRRPWPSLCW	2.04
GFGVLRRPRPTPGD	1.87
SAKELRRPPPRSPQ	2.06
LQPRPRRPREAPDT	2.35
AEGPPKRPGGSSPL	2.46
DPRTPRRPRSARAG	2.31
LATQPKRPMSIPII	2.82
VLLKLRRPVVGSSE	2.37
IFGPLKRPIVEFSL	1.79
SPIKPKRPFVESNV	3.33
PSRLPRRPNDIYVN	1.62
LAITLRRPGCELEA	1.45
KQVSPRRPQATEAV	2.10
QLWDIRRPDRCERM	2.84
SGGRLRRPVNVPLD	1.64
LSLLLKRPWDPAVG	1.59
PSFHIKRPAPRPLA	1.79
STSFPRRPLAVEGG	2.12
PKSVLKRPVTSEEL	1.69
NLSLLRRPGEKTYT	1.37
GTYQLRRPEDRPAG	1.34
ICPEFKRPRDIAVD	1.17
YRLPPTRPPASVSP	3.18
PQQFPSRPPPPQPK	3.72
APALPPKPPKPTTV	3.39
WGALPPRPPLLLLL	3.53
LSPRPEKPPEEPGQ	1.60
LFIIPPKPPGRPQG	2.73
GRTPPARPPQDPAE	2.11
VCARPEKPPGQVTR	2.00
LDPLPRRPPAGRRG	3.66
GGASPGRPPQPEQP	2.93
PEAVPGKPPFAEPD	1.80
PRLRPMRPPPPPPA	3.67
SFQSPTRPPLASPT	2.21
AGSSPGKPPRLVGG	2.01
CGPPPLRPPLPAAA	3.67
PRPGPCRPPPVPAP	3.28
ATDLPKKPPPPEVT	1.67
PPWVPWRPPSSAAV	2.92
SCTPPPRPPVLEEW	2.04
QRQRPPRPPPRGTP	4.04
AETRPHRPPGARDS	3.16
GPGKPERPPPQGGD	2.29
RHPKPLKPPWHLSM	3.51
CAGLPGKPPAIQAC	1.70
TTPPPGRPPAPSSE	3.69
RPPPPAKPPPDEEE	1.95
CARGPLRPPVRCPA	3.29
SNTPPPRPPKPSHL	4.25
FLLLPPRPPPARPE	3.31
KAGRPSKPPSPKQN	3.50
MHNFPLRPPPPPPP	2.33
VRPPPARPPSGPGL	4.11
TEGIPPKPPPRLVW	3.10
YDTFPEKPPPSPGD	1.99
PPHLPARPPGGWSR	3.38
LPPKPARPPAVSPA	3.63
SPTVPIKPPCIPNG	2.05
TQSLPTRPPTFPPT	3.08
MVPGPARPPGSCWD	1.53
RAEVPTKPPLPPAR	3.39
SQQPPSRPPHLPVE	2.89
PTTRPTRPPPPLIP	3.65
EYHRPAKPPPPPTG	1.93
GPAPPPRPPQLERH	4.03
SRSPPKKPPKRTSS	3.34
PTRPPSRPPSRPQS	3.77
VNEGPPKPPTELEP	0.53
LGKIPLKPPPLELL	1.79
PSPGPCRPPHGTQA	2.11
LPPLPHRPPQNPFV	1.90
RQPRPPRPPLGDRP	3.60
RCCFPVRPPTASTI	3.50
RAWIPGKPPCPPRG	3.68
LEIPPPKPPAPEMN	1.64
APLLPPRPPPLPPV	3.60
AGNLPSRPPPLSSP	3.03
LVPVPARPPLTRGQ	3.53
DLHPPPRPPSPTEI	3.42
EMTPPPKPPRLYLP	2.78
MAGLPFRPPWGCLS	3.64
EYALPQRPPQGPED	1.56
SSHSPYRPPTNYRN	2.83
QLRPPPKPPLPAPD	3.61
EPGLPVRPPGGGQS	1.77
KAVLPLRPPWPLWG	4.06
LPLPPPRPPLLSAR	3.93
FLGGPARPPSPSPS	3.94
SAPPPPRPPEEPAP	1.82
TACPPARPPAQVGL	2.56
RRQPPARPPGPSTD	3.98
PGPSPARPPAKPPE	2.29
LARIPNKPPPEYPG	1.31
VPQKPERPPLPPEP	3.67
ASSRPGRPPKRSLG	4.31
ALSLPEKPPRGEQG	2.05
SPCVPAKPPLAEFE	1.83

GPLLLTVPELPEAD	2.21
YEVTFDGPNDPLHP	2.04
KPLNLSKPISPPPS	2.41
HLDSFSPPFSPSTT	2.08
GWSTYIPPFNPLEI	2.06
LPQQHLSPLIPIAI	1.66
LPTALLSPLRPSLC	2.06
APPALSAPSVPPVP	3.82
VPGEHFAPMGPNGQ	1.81
DFVTLHVPATPETE	2.28
GLIGMNDPPRPNVK	2.06
HEHNYAIPYTPATF	2.11
APLLYSCPSEPMAY	1.92
KAPYLVQPNVPEKH	2.12
VPSAFTAPPVPTKK	2.55
QFQKLVPPTLPENV	2.19
KPSGYYGPNSPLKL	2.20
KAGSFGAPSSPTSG	2.09
MASFLPVPEKPNLI	2.21
IAGMLMVPESPRFL	2.41
SELYYEGPETPTPS	1.70
FLTVYILPKNPFRV	3.17
SFRELWQPVDPSRE	1.99
RLNSFAPPPNPYAT	2.44
TELNLSCPNVPGKP	2.36
RLSLLRSPKAPDAH	2.65
ALGIFFAPESPWWL	2.36
KFTDMLDPEKPNVI	2.42
RFHVFCEPILPSVA	2.20
TPYQLLSPILPVLL	2.46
AEFDLTDPEVPSFY	2.22
PPKLLVYPESPETN	1.96
KFFKHLYPAIPILD	2.34
RLQKLDDPKSPYQC	2.10
FESFLRPPSTPDLP	1.85
HFLCLDPPIDPNNL	2.09
RAAGMDVPIIPGIM	1.99
TPTPHPHPHTPHPH	2.20
SPNDHSDPSCPCNR	1.88
YWKLFFQPEYPVRM	2.16
VAKSYGFPVPPKVN	2.41
NLHGYGHPNPPNEK	2.34
KPLKFLRPTYPDLC	2.17
NPQSLARPPPPKRI	2.39
DPNQLSTPPTPKSA	2.22
KLALHQYPNFPSEG	2.11
SPSQYQYPSQPALS	1.81
RLLALFIPALPFII	2.42
FLSRYPVPDLPIYL	3.13
DEFTLDLPRIPSLE	2.26
SAFPYVFPLPPWIP	4.11
ELEDFAFPDTPTIV	2.41
KLFVLLPPIDPKDG	2.41
NETKLKFPTLPEVP	2.41
HLVVWNPPILPQHE	2.07
KELLLIPPGFPTKN	2.09
GEFFHCSPNIPHYA	2.43
VAPGLWFPNSPPPL	3.68
ELRTLFVPLIPSVT	2.47
GARFLPAPHRPEKY	2.18
LPAVFSAPIRPDIV	2.30
HPIHLQPPVPPVYK	2.50
VLEAYNVPTPPRLE	1.76
TAWDMVPPYSPLSE	2.10
TWKMHLWPVSPPQL	2.35
FLSYMSEPKYPSNE	2.15
HPEYLSLPFEPYCF	2.33
KLVKFKLPFLPISD	2.99
HEYNLALPKFPILF	2.17
NALKLIHPFMPFIS	2.23
SWAVLPVPGGPVMY	1.84
QEISLIDPSTPDKA	2.19
VPDSYKPPKNPIVF	2.92
KAIVWRLPKMPKIK	2.71
TLHELGVPITPIAF	2.07
ALRKFGYPFSPNDP	2.22
FEKRYYVPEDPSQD	2.08
PEHLFNNPQNPMDF	2.20
YLTEFSRPIPPTPS	2.23
NLAPFTQPDFPFQT	1.91
SASTMAAPVHPQQQ	2.22
FLRTMAQPTDPKDQ	2.41
AAMMLGNPMAPAVQ	2.41
TLFRFIVPFNPYFS	2.95
IPVGYVTPKFPSLY	1.33
TASQLQNPPPPPST	2.23
PLPTFSAPSLPQQS	4.67
APKKWKAPKGPKPT	3.00
QLVTLISPYDPNFK	2.25
SLQQFYNPESPIQP	2.12
FEDHHITPCKPGGQ	1.83
TPMDFPEPVGPLAI	2.24
LLQSLRLPTTPTER	2.10
KLKSLNLPQLPPPK	2.83
TLATLTEPKLPKSY	1.26
QFSGLLEPSLPNNM	1.93
VFSCLRDPVIPIKL	2.12
KLDPLGEPFDPNKH	2.44
EPEPLMSPVPPSSA	2.33
TPTHHHIPHIPNQN	2.17
NEHGMNAPSVPEFI	2.54
IPTSHAPPLPPTAP	4.64
APPALSAPSIPPVP	4.03
YFKELPPPSDPSSI	2.22
APQPHLLPWLPPTD	2.07
IFFKLIHPFLPILH	2.70
YASQYSRPDGPILL	2.05
DLSVHTSPHGPIGS	2.27
LAIRLCWPPAPVTL	2.19
MEEPFIWPEEPRPD	1.90
FARTLKIPNEPSPG	2.16
TPINFVFPNLPLEH	1.91
PPMAYNRPVYPPPQ	3.83
YPAKLRIPETPVKK	2.34
VFGTYLGPDAPLSK	2.12
SAENLIQPTAPIRD	1.75
LALLMTDPDAPSRT	2.25
IAKPYVLPFFPLYA	2.39
AARILANPDHPFSR	2.22
NEAPMLSPQRPYST	2.05
GLIAHQGPQFPVMG	2.38
TEEILPTPVQPGGH	2.08
YEADYVIPVLPGSH	2.10
DLIHLDCPQQPNGY	2.34
LLYLMTYPQQPMVK	2.21
ILVAYGLPVSPDEK	2.22
HAVLLNPPVAPTAE	2.36
KPAVLHYPPIPASA	2.26
EAGDFEQPKGPAGA	1.70
FESTLPIPTYPREG	2.09
AEVYYGPPTDPRKQ	1.92
KFTDMQVPKGPVCP	2.29
FLYPYDDPNAPIDE	1.74
SPKKHVSPSRPPSV	3.15
PPEGFPEPLNPTNF	1.93
ILTCLPVPPPPVRP	2.79
QPQAYHLPGNPYST	2.27
TLIEYMGPAPPKGS	2.12
LWILYMSPDVPSAS	2.40
HEKLYHNPSLPAQS	1.89
PLNQLPNPAMPLLH	2.02
FENLLMEPNLPFFT	2.31
ILIFLWIPDDPSKA	3.14
LLKGYIPPYKPIVK	2.23
KWKSLFPPFFPCDI	2.38
SWLTMLPPMAPLLS	2.70
HLARYIFPPNPVFH	2.45
ILPVLNIPLPPQEK	1.33
NEFLHFDPHIPQPA	2.19
ELRKLVQPITPIIK	2.23
KPPSYPSPVQPPQS	4.22
SLTSLPPPPPPQFS	3.86
AELLLGQPMFPGES	2.24
PFSSMKEPFIPQSS	2.61
RPQWLSYPEQPLNS	2.38
TFRDMDLPQHPVIH	2.27
MAKSFADPRVPQPM	2.24
REFEFIIPVFPVIC	2.21
WLSKLVLPPPPVSI	3.62
PEVHHHCPGVPCLV	2.52
TLKKFVLPCLPRKN	2.70
AELFLGIPIFPGAS	1.56
NLLSLENPVVPSHY	1.88
SPRHYNVPSSPIAP	2.37
RPSKFFHPPIPHIP	2.89
NPLYWLLPSHPYTI	2.22
VPGGMSIPGAPQGA	2.14
SPSDFNKPDLPELI	2.50
KLPQLPPPPPPPPP	4.73
IPLPHQPPPLPIYS	4.73
EARPYSEPSEPEDS	2.32
DARPFLKPVDPIAL	2.10
ALKSLSNPSSPWQQ	1.48
ILHQLGAPSIPENL	2.24
PEVKHFCPSTPIML	1.97
PFYTMPDPANPKYS	2.16
LWKPLVYPTCPSEH	2.27
GETQWRIPYLPLDP	2.03
IARPLSVPGSPKVR	2.58
MANILYYPQKPLGT	2.18
KFLIHNEPIDPNDR	2.09
VLGVLNVPLPPQTR	2.37
PPQTHQSPPPPPQQ	2.35
NFLKHYLPSSPGDI	2.24
ALNNWILPSTPHIQ	2.23
KELPFTGPSIPFEN	2.53
SLLIHSLPRKPLLM	2.50
AANAHISPPVPGPA	1.46
AAYDHAEPVQPEDA	2.16
GETRWRIPYLPLDP	2.07
NAIEMALPNDPYPA	1.94
SPFTFLFPFLPTII	2.56
QPTNFQYPPPPFGQ	2.35

TNYPVNPEKHEMDK	1.79
VTVPKLPFKLAVNK	1.70
PAFPEYPMRRDTLY	1.76
LKRPILPSKNMDHT	2.15
DSLPKLPFRSWGQP	4.21
ELEPQHPLRHKSSL	1.58
EMIPSSPQRKSVSN	2.34
LTLPFGPVKSIWLS	1.24
IEQPFGPPKITKDG	1.45
KLPPRLPHRAAQRR	4.04
IMPPPKPFRHGLDS	3.19
CILPAFPCKSSNEQ	1.83
AGHPMKPHRIRMAH	2.45
QAHPPKPKRREGDI	1.61
QRQPKRPKRYSLLL	2.02
KKCPRLPHRNKKVT	3.42
FCHPLSPLKSNFID	1.96
YSNPQTPLRSLAGL	2.42
PSAPKVPFKTLDSF	1.57
KYGPLPPSRACVGS	1.82
QTYPAQPPKIGNAF	1.53
SVLPQTPLKDVMGG	1.65
KNLPTIPIRLCGID	4.46
LGAPFCPRRKDKLV	1.77
FGTPVAPTRTLSRK	2.60
IEVPQSPTKNRVKV	1.43
FYEPLDPQKDKDMT	1.15
SHQPYLPIKNKNIV	1.70
IFFPNEPIRFVYPY	1.82
TRRPHHPLKQQSTN	1.85
LDHPNIPWKLIISG	0.88
WKPPDLPIRLRKRP	4.11
LVQPNVPEKHNMST	1.64
KFHPPEPYKGKGIY	1.73
SPLPVLPRRISTHT	4.28
NYIPPDPPKDIQLN	2.25
QFVPIAPQRLNMIF	1.95
NSPPVYPKRIRHFE	3.56
VPRPCPPIRRKSST	3.51
YSLPMHPGRWWVHT	1.89
VMVPHLPGKILLSN	1.76
NPPPVPPPRNVTAM	3.08
DLPPEPPTKLSDSF	1.49
KPDPECPSKLRHYE	1.36
GRHPDMPTRVKNAY	2.17
PVGPKVPAKEPSPK	1.49
KIMPPTPAKKVEFI	2.25
RAVPILPPRNNVEP	4.77
CNEPHLPPKWVQRS	0.61
ITSPPLPPRADCIE	4.50
AMTPFSPRKSHINN	2.63
EIAPLIPKKPALRK	1.90
QAAPRNPNKDATIA	1.61
AFVPTNPWRSSMSL	1.76
VETPPKPIKKDVRF	1.94
EESPVKPLKITQKA	1.71
QMNPKNPIRLGMAN	1.41
HSTPVTPRRLFTSQ	2.45
ELPPTHPIRLGLAL	1.39
DERPFRPKRTSRSA	2.05
GAPPTTPSRPRTTT	2.56
GRKPKDPSKPRQML	1.50
RVHPEPPNKPGITT	1.74
KDHPYDPEKDSVLK	1.34
RFDPKSPTKRSIAI	1.60
NQAPLEPLKPTATG	1.58
VIRPGVPTKFTKKQ	1.76
NHIPPFPLRFSFAQ	2.03
TEPPMNPLKNREKM	2.17
QGEPLDPKRPSTAS	1.66
DMVPKHPTKRYVPK	1.97
KSVPIPPRKNATLD	4.08
NKVPTNPKKFKKAP	1.31
IAAPKKPRKPRQTK	1.98
SSQPRPPPRPQQNP	2.98
QYIPPPPPKYINSK	2.05
TTAPRGPKKAQPAQ	1.95
DKLPFSPIKEKKLK	2.07
SMSPLPPARANKSD	3.42
ANLPNRPKRGEGIN	1.60
VTTPLKPKRLAIPI	1.25
AGSPIRPYKQRYLL	2.54
MLIPNFPNKKLKLN	1.85
DSLPFAPLRKVYEL	2.19
SNVPTTPSRELNGT	1.57
KERPKPPNKGDVGR	1.03
PIQPQEPFRHNTTI	1.31
FREPDLPRKGLLED	1.52
SSLPSPPAKKSATS	1.61
DQKPLVPIRIYGAI	2.06
SLPPTPPYKAHKKK	1.47
SSTPTTPERPKRKS	2.76
EGKPLFPGKDHVHQ	1.59
YMQPIIPGKTECFE	2.70
VHGPVIPMKALEIA	1.44
FWNPENPYKFVHPR	1.83
MEYPFKPPKMQFDT	1.84
FPYPFKPSRVGVKY	2.07
RPSPWLPSKPNCYH	2.15
LVQPVVPDKVFSFR	1.80
VHGPGVPVKTLEIA	1.72
IYTPYIPFRNSRTW	2.68
YKIPVVPPRETYFA	2.59
FYYPESPTKNLRPR	1.46
NEGPEYPTKIEKNQ	1.32
VTQPITPVKKRRSE	1.75
EDTPDLPQKTGIRK	1.16
NWSPNIPLRYSDFA	2.28
TTRPAPPSKAKDVA	2.10
SASPTLPTRRSRRL	4.89
WNLPWSPFKITTIM	2.94
FQPPPKPFRRSQSS	3.93
LRKPRAPMRNVIIT	2.36
IAYPGDPLRFHSHL	1.26
LWQPVDPSREDLVI	-0.10
IIIPPIPSKHSLLK	1.35
DKLPEGPVKSLEGA	1.75
HNSPVAPNRYAANL	1.94
CREPEVPQRLLTLI	1.63
NLKPERPSKVSFEM	2.91
FKAPTNPRKVYCKR	1.53
MVRPAPPRRADPEG	4.16
MVTPSKPPKKSVGF	2.06
DRPPDVPRKIIVGR	1.97
FLQPVDPVKLDIPF	1.75
RAPPPVPKKPSSRI	1.46
LGEPFDPNKHEATF	1.03
NIQPDLPPRKIMIP	4.52
MTDPDAPSRTEHKW	1.36
LDDPFTPPRWLHHI	1.95
VMKPKSPDKEFFDA	1.68
IRYPVTPNKASLDR	1.61
KNSPLAPIKKNVEL	1.43
MKAPKMPDKHRDNY	2.52
SGKPLFPGRDYHHQ	2.02
PVDPTTPVKLGPGI	1.28
TNEPTKPIRRLSTP	1.67
FFDPESPNKGKLIL	1.79
LDRPQLPPRQVVNA	4.41
ALVPFAPFKLLKDR	2.13
FDFPLSPVKHENPT	1.46
KDAPIEPPKYMGMG	1.60
QSTPTPPAKRLFLI	1.55
ELLPVVPEKFLIAC	1.18
NYAPRLPRRETSSS	4.45
FVPPNLPMRRFKEI	4.07
DVIPKKPLKSAADK	0.99
GAIPRGPKKDDASF	1.68
HSSPIAPSKAKRQR	2.75
AVLPIEPMKIKGTT	1.59
MSEPEVPFKVVAQF	1.30
RMIPELPPKRIGSQ	3.69
LRSPQNPNRMTAVI	1.32
PSNPALPKKLLNVP	1.45
LRLPEIPPKGDFDV	1.46
HQIPSAPQRLVSII	1.20
YRYPQTPMRLEIAV	1.96
GGPPLLPQKSAAFQ	1.45
KYDPDNPNKDKLGT	1.68
DELPSPPLRMVHID	2.09
DSIPLEPKKAKHLM	1.53
QSQPPRPPRPAANL	2.02
LAPPLSPTKPSPLH	1.49
TLRPGIPLKPMLAK	2.07
EEHPPLPARRKSEE	4.42
YRIPDDPRKSQNKS	1.33
WSYPYEPLRVLQVG	3.04
ACLPVGPRKNGMNV	2.23
EYFPISPEKKEEME	1.51
VYSPIQPRRRKDSS	2.76
LLAPNSPLKNTKSV	1.42
TKKPRPPVKSKPKH	1.74
TPPPVEPLKTKDLA	1.75
DQAPSLPPKPNTQL	1.51
PLIPDNPGRLLSSK	1.63
NNGPKIPQKNTIDS	1.42
NSPPTTPSKDECGP	1.60
MVLPPAPPKNSDPS	1.90
SKVPKAPTRETHIP	1.67
LSCPNVPGKPQVAY	0.18
FHDPILPRRTSTFL	3.83
VGNPEGPEKPNKKF	1.54
LPAPPPPPRRGPAP	3.75
SPSPLNPYRPHHNM	1.64
GVYPSRPEKTYDYS	1.11
GGQPPVPVRMQPQP	2.90
GPAPPPPPRASRPT	3.49
LSLPRLPNKHHWPL	2.40
PPPPPPPPKPPLND	1.56
RGIPARPCKDDVMK	3.03
GPPPAMPARPTATT	1.92
PYPPSLPSRDLYEV	2.97
LPPPRSPNRPTLSD	4.18

KWEPSTPQRNGPIL	2.24
AIEPGVPSRYSDET	2.10
GNTPDAPKRGFLGY	1.66
LNSPDLPERTKLRN	2.04
IFGPSFPFRSHIDV	2.26
QLLPNVPDKVLVPG	1.67
NNNPVPPQKINQPP	2.28
SHQPYLPIKNKNIV	1.82
TDSPQLPSKDKSQE	1.91
EYHPIKPLRSSSST	2.02
VIRPGVPTKFTKKQ	2.71
TLRPGIPLKPMLAK	2.66
SASPTLPTRRSRRL	2.33
QLFPVRPVRVRHED	1.98
PPPPPPPSRKCGTP	2.42
LPVPPPPVRPSISF	2.24
LVTPEEPAKNKSER	2.01
VVIPPVPSRYSDER	1.76
SSMPNSPKKLKKLN	1.80
TLIPPIPEKQSIKN	1.94
VSTPPVPKKFQEIF	2.37
DSGPQRPKRTRAKG	2.31
NEYPSKPPKVKFPA	2.52
NLDPNPPVRGENLT	2.31
SASPYRPQRLPAKF	2.07
MKAPKMPDKHRDNY	2.26
TEPPMNPLKNREKM	2.14
SMYPGLPSRLEKEL	2.52
KNLPLNPSKKLEGK	2.31
VSDPYVPTRNTFNY	2.59
ERDPNMPKRRTNAY	1.92
YGPPTDPRKQKQAK	2.30
KRTPKIPEKDVKSD	3.60
HPDPADPAKELELN	2.30
TDYPFKPRVNFTT	1.87
NSPPVYPKRIRHFE	2.25
EIAPLIPKKPALRK	2.26
VETPPKPIKKDVRF	3.93
GYDPTDPSRPRINE	2.08
ELPPTHPIRLGLAL	2.16
DYLPPHPHRTSSLP	1.94
LSTPSAPAKINNYS	1.83
LDDPFTPPRWLHHI	1.97
IIFPTSPTKLTFSN	2.10
RPSPWLPSKPNCYH	3.11
QGYPRQPQRPQRYH	2.29
LTSPKNPIKSVTKF	2.17
ERDPNAPKKPLTVF	2.20
MEYPFKPPKMQFDT	2.21
PFLPVLPQKPGGVR	3.14
NKVPTNPKKFKKAP	2.46
QIFPKKPKRFVMS	2.73
GRHPDMPTRVKNAY	2.28
SKGPRTPAKEILEF	3.21
QYIPPPPPKYINSK	2.48
WNLPWSPFKITTIM	3.09
SNSPISPHKLIYKN	1.94
RRKPHSPNKKKPKE	2.24
TSAPDIPPRSPNRN	2.03
RRGPGRPRKDASTS	3.00
IFPPLNPARTSLNS	2.01
DYLPPVPEKNAGKQ	2.35
RVHPEPPNKPGITT	2.17
SLAPVYPLKSLLRI	1.96
RPNPTQPRKPLDCK	2.00
VKRPLSPTRINGKS	2.20
YMQPIIPGKTECFE	2.22
CILPAFPCKSSNEQ	1.87
IVNPFIPRRPYSNV	2.72
WSYPYEPLRVLQVG	2.95
SHLPLIPEKIKSLK	2.24
YSNPQTPLRSLAGL	2.34
NHIPPFPLRFSFAQ	2.26
IIIPPIPSKHSLLK	2.55
LAPPLSPTKPSPLH	2.03
IFGPFGPMKEVKIL	2.40
DLPPKFPEKPVHLV	1.82
EILPQAPAKKVFES	2.12
RERPLEPPRKPMAS	2.74
TEVPSKPKKPSKTE	1.90
SGKPKKPYRRWTPE	2.37
VQQPEDPLRVLGEY	2.11
KKYPRKPKKNLNLG	2.35
NMVPLNPNRIIPDE	2.11
INQPSTPKKLKRKS	2.15
EVSPVEPKRTENER	2.26
FTAPPVPTKKKSKN	3.64
STSPPRPKRIVVAI	2.06
LRSPNTPRRLRKTL	2.35
LRSPNTPRRLRKTL	2.11
LRSPNTPRRLRKTL	2.16
NLKPERPSKVSFEM	2.53
GGDPTKPVRTWNDL	2.05
GITPVGPGKFCRSA	2.19
PVGPKVPAKEPSPK	3.76
LSSPCLPPKVNATN	1.94
YQYPQTPSKVDISI	2.29
VSIPFDPSKDTFDH	1.98
PLIPDNPGRLLSSK	1.92
FWNPENPYKFVHPR	2.11
SSFPTIPLRASSGA	2.32
HGVPVVPSREVTIN	2.14
CQRPGCPGRYKLVR	2.25
ELEPQHPLRHKSSL	2.01
MSLPVTPVKSKVCS	2.45
RPPPLTPEKNLYLT	2.07
SEEPTSPTRQVNPE	2.20
LVQPVVPDKVFSFR	1.66
FFHPAIPLKNAAPF	2.21
TKTPTQPIRLQTVS	1.60
DVYPHEPAKNGEGS	2.12
SNFPDFPLKKYLKN	2.06
IHSPRIPSKDEFIA	1.98
YPSPAQPPKSPLLN	2.11
RPTPRKPSRMATDH	3.16
PPVPKAPSRRSSSL	2.16
ETVPTEPTRYNYQN	1.52
LHGPFKPVKSSNGE	1.79
LRKPRAPMRNVIIT	2.27
SDKPLTPTKTSSTL	2.13
DELPSPPLRMVHID	2.35
LPPPRSPNRPTLSD	1.96
RGTPANPGRVATLI	2.09
KKLPTGPSKKRQRK	2.31
PNSPTPPLKSKVSR	2.16
EANPDNPKKLDLKA	2.12
GYDPTDPNRPKVTK	2.20
SSRPTSPVRKARST	2.01
ELPPTHPIRLGLAL	2.25
QDSPDMPEKECVYV	2.07
LRSPNTPRRLRKTL	2.27
KLEPENPQKPSNTH	1.97
MVLPPAPPKNSDPS	2.15
LRLPEIPPKGDFDV	2.99
DGRPHSPLRARSAT	1.57
RKRPAPPKKKAPST	2.32
ISTPPPPPKMAIPA	1.75
NVQPRKPRSVHSRN	1.92
QTSPGQPRKGQNGS	1.79
KLRPDLPVKNFVVK	3.32
YTLPLLPGKGGFRI	2.71
VTLPISPSRAIGSF	2.08
ILKPDIPLRNMLLR	2.20
LLKPETPSKRLVIR	2.46
TKVPSKPDRNSKIL	2.12
PSNPALPKKLLNVP	2.25
SAFPQSPIRAYGST	1.88
LRDPVIPIKLAAEK	2.09
VKHPMKPFRLMLTD	2.84
TSTPPLPRRRATTS	2.56
FCHPLSPLKSNFID	2.21
DERPFRPKRTSRSA	2.26
GSSPVSPSDKRPSWK	1.78
AMPPTLPHRDWKDY	2.51
ILLPPLPERAYIMD	2.38
FPYPFKPSRVGVKY	2.08
FRFPRLPQKKKEEL	2.89
ECHPELPNKDVKLD	1.73
NVDPASPRKWHELLS	2.01
PNYPDSPPKVTFIS	2.27
KNLPTIPIRLCGID	4.50
NKIPPKPRKVLQLL	2.18
AGSPIRPYKQRYLL	1.94
ILSPGYPNKSFNNK	2.11
PIMPTLPPRPYITI	2.36
SALPILPKREVVTR	2.15
QGEPLDPKRPSTAS	2.09
IAAPKKPRKPRQTK	2.51
GLEPNTPLKSIKVD	2.14
HSTPVTPRRLFTSQ	2.38
QYYPEEPTKPVTKT	2.05
KFIPNKPYRQFNTW	1.78
KDHPYDPEKDSVLK	1.41
AAWPLDPARERWTR	2.14
SGKPLFPGRDYHHQ	2.01
FVIPGVPSKCYSLF	2.45
FISPHLPMKGRLVL	2.64
NANPFLPNKHPVTQ	2.32
GSNPDLPNKSNLKF	2.15
LIDPSTPDKAAKKD	2.01
PLQPRPPVKPLSKQ	1.93
IQHPFLPRKIPIIT	2.22
TTAPRGPKKAQPAQ	1.66
SNDPIPPPRKVSDV	2.03
PVYPSLPERSKVIL	2.32
DESPIDPSKAPKVP	1.99
SSYPMNPPKISFMQ	1.87
KKDPFPPPKDLIAS	1.81
RLIPTSPQKTNINT	1.93
LPAPHRPEKYKERF	2.24
ATTPAIPTKSDEAE	2.23
IAYPFYPFKKLGRQ	1.77
ELDPICPLKVPLTA	2.11

RFDPKSPTKRSIAI	2.51
NQAPLEPLKPTATG	1.40
VIRPGVPTKFTKKQ	2.30
NHIPPFPLRFSFAQ	3.84
TEPPMNPLKNREKM	2.81
QGEPLDPKRPSTAS	2.64
DMVPKHPTKRYVPK	3.25
KSVPIPPRKNATLD	4.40
NKVPTNPKKFKKAP	2.25
IAAPKKPRKPRQTK	3.50
SSQPRPPPRPQQNP	3.94
QYIPPPPPKYINSK	2.59
TTAPRGPKKAQPAQ	2.23
DKLPFSPIKEKKLK	1.81
SMSPLPPARANKSD	4.22
ANLPNRPKRGEGIN	3.80
VTTPLKPKRLAIPI	3.28
AGSPIRPYKQRYLL	3.25
MLIPNFPNKKLKLN	3.04
DSLPFAPLRKVYEL	2.51
SNVPTTPSRELNGT	2.43
KERPKPPNKGDVGR	2.51
PIQPQEPFRHNTTI	1.78
FREPDLPRKGLLED	3.13
SSLPSPPAKKSATS	2.48
DQKPLVPIRIYGAI	3.71
SLPPTPPYKAHKKK	2.88
SSTPTTPERPKRKS	4.76
EGKPLFPGKDHVHQ	0.72
YMQPIIPGKTECFE	2.78
FWNPENPYKFVHPR	2.72
MEYPFKPPKMQFDT	2.68
FPYPFKPSRVGVKY	3.45
FVIPGVPSKCYSLF	1.65
RPSPWLPSKPNCYH	3.80
LVQPVVPDKVFSFR	2.49
VHGPGVPVKTLEIA	1.64
IYTPYIPFRNSRTW	3.52
YKIPVVPPRETYFA	3.52
FYYPESPTKNLRPR	2.47
NEGPEYPTKIEKNQ	2.10
VTQPITPVKKRRSE	3.42
EDTPDLPQKTGIRK	3.75
NWSPNIPLRYSDFA	3.57
TTRPAPPSKAKDVA	2.88
SASPTLPTRRSRRL	4.69
WNLPWSPFKITTIM	3.81
FQPPPKPFRRSQSS	4.65
LRKPRAPMRNVIIT	3.52
IAYPGDPLRFHSHL	1.97
LWQPVDPSREDLVI	2.25
IIIPPIPSKHSLLK	3.50
DKLPEGPVKSLEGA	1.94
HNSPVAPNRYAANL	2.19
CREPEVPQRLLTLI	3.06
NLKPERPSKVSFEM	3.61
FKAPTNPRKVYCKR	2.43
MVRPAPPRRADPEG	4.28
MVTPSKPPKKSVGF	3.42
DRPPDVPRKIIVGR	3.47
RAPPPVPKKPSSRI	4.46
LGEPFDPNKHEATF	1.83
NIQPDLPPRKIMIP	4.44
MTDPDAPSRTEHKW	2.08
LDDPFTPPRWLHHI	3.53
VMKPKSPDKEFFDA	2.93
IRYPVTPNKASLDR	1.98
KNSPLAPIKKNVEL	2.40
MKAPKMPDKHRDNY	3.25
SGKPLFPGRDYHHQ	3.31
TNEPTKPIRRLSTP	3.70
FFDPESPNKGKLIL	1.83
LDRPQLPPRQVVNA	4.16
ALVPFAPFKLLKDR	2.08
FDFPLSPVKHENPT	1.72
KDAPIEPPKYMGMG	1.64
QSTPTPPAKRLFLI	3.15
ELLPVVPEKFLIAC	1.14
NYAPRLPRRETSSS	4.46
FVPPNLPMRRFKEI	4.41
FFQPEYPVRMEPDL	3.02
DVIPKKPLKSAADK	2.56
GAIPRGPKKDDASF	2.35
HSSPIAPSKAKRQR	3.13
AVLPIEPMKIKGTT	2.78
MSEPEVPFKVVAQF	1.69
RMIPELPPKRIGSQ	4.54
LRSPQNPNRMTAVI	3.08
PSNPALPKKLLNVP	3.01
LRLPEIPPKGDFDV	2.51
HQIPSAPQRLVSII	2.76
GGPPLLPQKSAAFQ	3.85
KYDPDNPNKDKLGT	1.32
DELPSPPLRMVHID	3.87
DSIPLEPKKAKHLM	2.15
QSQPPRPPRPAANL	4.44
LAPPLSPTKPSPLH	2.05
TLRPGIPLKPMLAK	2.48
EEHPPLPARRKSEE	4.49
YRIPDDPRKSQNKS	2.55
WSYPYEPLRVLQVG	3.37
VYSPIQPRRRKDSS	4.12
LLAPNSPLKNTKSV	1.98
TKKPRPPVKSKPKH	2.54
TPPPVEPLKTKDLA	1.56
DQAPSLPPKPNTQL	4.24
PLIPDNPGRLLSSK	2.30
NNGPKIPQKNTIDS	2.63
NSPPTTPSKDECGP	2.00
MVLPPAPPKNSDPS	2.95
SKVPKAPTRETHIP	3.11
LSCPNVPGKPQVAY	2.30
FHDPILPRRTSTFL	4.65
VGNPEGPEKPNKKF	2.27
LPAPPPPPRRGPAP	3.80
SPSPLNPYRPHHNM	2.54
NIPPNSPGKLQLNT	1.93
GVYPSRPEKTYDYS	2.67
GGQPPVPVRMQPQP	3.78
GPAPPPPPRASRPT	3.80
LSLPRLPNKHHWPL	4.43
PPPPPPPPKPPLND	3.01
RGIPARPCKDDVMK	3.86
GPPPAMPARPTATT	4.06
PYPPSLPSRDLYEV	3.92
LPPPRSPNRPTLSD	4.53
VDPPFMPFRDAGYS	2.76
VKEPSFPSRSAFSP	3.04
IAYPFYPFKKLGRQ	2.91
WISPDTPLKFPLNE	3.77
ISTPEVPKKKIKTG	3.70
QTSPGQPRKGQNGS	2.07
WNDPSDPTKGFQYL	3.11
IAHPVRPHRAITMT	3.42
GLEPNTPLKSIKVD	1.83
GNVPHSPVRFADTV	-0.10
ASMPRVPLKIVLKD	3.84
PPVPKAPSRRSSSL	4.47
RNEPVKPFRNIDLK	3.78
KLEPENPQKPSNTH	1.06
HLDPEAPDKLLENS	1.55
VSPPFFPEKELNNR	2.51
PNSPTPPLKSKVSR	2.94
VCPPSFPSRRCETC	4.61
TRRPTNPFRVISVS	3.28
IINPDTPHRRIFNQ	3.22
EYSPLVPSKMCADK	2.57
FKMPKKPQRSKEEV	3.90
TGRPDKPFRALLVD	3.75
IIFPTSPTKLTFSN	2.50
PIMPTLPPRPYITI	4.42
RLIPTSPQKTNINT	2.74
ATRPSSPMRHKSIS	3.79
EILPVKPTKKSSKK	3.43
FRFPRLPQKKKEEL	4.29
LRIPETPVKKSPLV	2.21
RPNPTQPRKPLDCK	2.43
YTLPLLPGKGGFRI	2.68
LIPPLHPLKKQHPK	3.15
DLQPLEPKKAAAST	1.58
ILSPGYPNKSFNNK	2.47
VVIPPVPSRYSDER	3.65
ALRPTGPDRGDGFD	2.16
SNSPQIPKKKVKDQ	3.26
SNDPIPPPRKVSDV	3.91
QDSPDMPEKECVYV	1.43
TNPPIDPIREANVM	1.17
EYHPIKPLRSSSST	3.50
ISTPPPPPKMAIPA	2.52
ELPPIKPPRPTSTT	4.76
PIVPREPLRNEPTD	3.87
ITLPRRPAKKIQLG	4.69
DVYPHEPAKNGEGS	2.10
LRKPSAPKRMSSIS	3.47
GKVPVQPRKRKSIT	3.15
ELDPICPLKVPLTA	1.63
ERDPNAPKKPLTVF	1.97
NDDPVIPYKLSKDT	2.30
NSRPQQPERRRFNS	4.61
VQTPHVPDRPPSQL	3.62
GNTPDAPKRGFLGY	2.82
KRPPTAPQRKISLP	3.94
RRKPHSPNKKKPKE	2.84
SNNPSKPRKYKTKS	2.49
RSIPRGPDKNSLRV	2.58
IQHPFLPRKIPIIT	3.05
SGPPLLPPRNTMKN	4.52
NAPPYVPQRSKQPD	4.07
QRLPGEPGREQAPN	1.79
GRIPQLPNRDEMTA	4.06
MRAPPSPRKSKSGH	3.09
LIPPGFPTKNYPPG	1.93
KLGPDTPIKVAGPL	2.29

ELEPQHPLRHKSSL	2.52
EMIPSSPQRKSVSN	2.51
LTLPFGPVKSIWLS	2.47
IEQPFGPPKITKDG	1.71
KLPPRLPHRAAQRR	4.34
IMPPPKPFRHGLDS	4.55
CILPAFPCKSSNEQ	2.03
AGHPMKPHRIRMAH	2.95
QAHPPKPKRREGDI	2.87
QRQPKRPKRYSLLL	2.79
DESPIDPSKAPKVP	2.08
KKCPRLPHRNKKVT	3.25
YSNPQTPLRSLAGL	3.20
PSAPKVPFKTLDSF	2.25
KYGPLPPSRACVGS	2.69
QTYPAQPPKIGNAF	2.42
SVLPQTPLKDVMGG	2.10
KNLPTIPIRLCGID	3.58
LGAPFCPRRKDKLV	2.40
FGTPVAPTRTLSRK	2.43
IEVPQSPTKNRVKV	2.30
FYEPLDPQKDKDMT	2.11
SHQPYLPIKNKNIV	2.00
IFFPNEPIRFVYPY	2.38
TRRPHHPLKQQSTN	2.19
LDHPNIPWKLIISG	2.18
WKPPDLPIRLRKRP	4.39
LVQPNVPEKHNMST	2.03
KFHPPEPYKGKGIY	2.06
SPLPVLPRRISTHT	4.47
NYIPPDPPKDIQLN	1.90
QFVPIAPQRLNMIF	2.78
NSPPVYPKRIRHFE	4.34
VPRPCPPIRRKSST	3.59
YSLPMHPGRWWVHT	2.49
VMVPHLPGKILLSN	2.59
NPPPVPPPRNVTAM	4.08
DLPPEPPTKLSDSF	2.02
KPDPECPSKLRHYE	2.38
GRHPDMPTRVKNAY	2.18
PVGPKVPAKEPSPK	1.69
KIMPPTPAKKVEFI	2.83
RAVPILPPRNNVEP	4.53
CNEPHLPPKWVQRS	1.92
ITSPPLPPRADCIE	4.97
AMTPFSPRKSHINN	2.41
QAAPRNPNKDATIA	1.67
AFVPTNPWRSSMSL	1.77
VETPPKPIKKDVRF	2.57
EESPVKPLKITQKA	1.71
QMNPKNPIRLGMAN	2.26
HSTPVTPRRLFTSQ	3.81
ELPPTHPIRLGLAL	2.31
DERPFRPKRTSRSA	3.00
GAPPTTPSRPRTTT	4.00
GRKPKDPSKPRQML	2.20
RVHPEPPNKPGITT	2.40
RFDPKSPTKRSIAI	1.86
NQAPLEPLKPTATG	2.32
VIRPGVPTKFTKKQ	2.10
NHIPPFPLRFSFAQ	3.41
TEPPMNPLKNREKM	2.10
QGEPLDPKRPSTAS	2.62
DMVPKHPTKRYVPK	2.66
KSVPIPPRKNATLD	3.00
NKVPTNPKKFKKAP	2.32
IAAPKKPRKPRQTK	2.10
SSQPRPPPRPQQNP	3.31
QYIPPPPPKYINSK	1.91
TTAPRGPKKAQPAQ	2.42
DKLPFSPIKEKKLK	2.19
SMSPLPPARANKSD	4.23
ANLPNRPKRGEGIN	3.09
VTTPLKPKRLAIPI	2.56
AGSPIRPYKQRYLL	2.25
MLIPNFPNKKLKLN	2.63
DSLPFAPLRKVYEL	2.30
SNVPTTPSRELNGT	2.32
KERPKPPNKGDVGR	1.78
PIQPQEPFRHNTTI	1.72
FREPDLPRKGLLED	2.07
SSLPSPPAKKSATS	1.79
DQKPLVPIRIYGAI	3.11
SLPPTPPYKAHKKK	2.03
SSTPTTPERPKRKS	4.50
EGKPLFPGKDHVHQ	1.96
YMQPIIPGKTECFE	2.44
VHGPVIPMKALEIA	1.97
FWNPENPYKFVHPR	2.39
MEYPFKPPKMQFDT	2.25
FPYPFKPSRVGVKY	2.42
FVIPGVPSKCYSLF	1.82
RPSPWLPSKPNCYH	2.76
LVQPVVPDKVFSFR	2.57
VHGPGVPVKTLEIA	2.06
IYTPYIPFRNSRTW	2.83
YKIPVVPPRETYFA	3.24
FYYPESPTKNLRPR	1.56
NEGPEYPTKIEKNQ	2.04
VTQPITPVKKRRSE	2.11
EDTPDLPQKTGIRK	2.00
NWSPNIPLRYSDFA	3.15
TTRPAPPSKAKDVA	2.40
SASPTLPTRRSRRL	4.99
WNLPWSPFKITTIM	3.02
FQPPPKPFRRSQSS	4.71
LRKPRAPMRNVIIT	2.41
IAYPGDPLRFHSHL	2.29
LWQPVDPSREDLVI	2.14
IIIPPIPSKHSLLK	2.31
DKLPEGPVKSLEGA	1.95
HNSPVAPNRYAANL	2.39
CREPEVPQRLLTLI	2.25
NLKPERPSKVSFEM	3.13
FKAPTNPRKVYCKR	2.15
MVRPAPPRRADPEG	3.89
MVTPSKPPKKSVGF	2.56
DRPPDVPRKIIVGR	2.55
FLQPVDPVKLDIPF	2.07
RAPPPVPKKPSSRI	2.70
LGEPFDPNKHEATF	2.21
NIQPDLPPRKIMIP	4.75
MTDPDAPSRTEHKW	2.38
LDDPFTPPRWLHHI	2.85
VMKPKSPDKEFFDA	2.45
IRYPVTPNKASLDR	2.32
KNSPLAPIKKNVEL	1.20
MKAPKMPDKHRDNY	2.60
SGKPLFPGRDYHHQ	2.95
PVDPTTPVKLGPGI	2.25
TNEPTKPIRRLSTP	3.11
FFDPESPNKGKLIL	2.11
LDRPQLPPRQVVNA	4.46
ALVPFAPFKLLKDR	2.09
FDFPLSPVKHENPT	2.13
KDAPIEPPKYMGMG	2.00
QSTPTPPAKRLFLI	2.16
ELLPVVPEKFLIAC	2.17
NYAPRLPRRETSSS	4.70
FVPPNLPMRRFKEI	4.37
FFQPEYPVRMEPDL	2.32
DVIPKKPLKSAADK	1.57
GAIPRGPKKDDASF	2.37
HSSPIAPSKAKRQR	2.15
AVLPIEPMKIKGTT	2.28
MSEPEVPFKVVAQF	2.02
RMIPELPPKRIGSQ	4.28
LRSPQNPNRMTAVI	2.19
PSNPALPKKLLNVP	1.94
LRLPEIPPKGDFDV	2.09
HQIPSAPQRLVSII	2.33
YRYPQTPMRLEIAV	2.56
GGPPLLPQKSAAFQ	2.07
KYDPDNPNKDKLGT	2.42
DELPSPPLRMVHID	3.83
DSIPLEPKKAKHLM	2.31
QSQPPRPPRPAANL	4.04
LAPPLSPTKPSPLH	1.89
TLRPGIPLKPMLAK	2.36
EEHPPLPARRKSEE	4.86
YRIPDDPRKSQNKS	1.91
WSYPYEPLRVLQVG	2.90
ACLPVGPRKNGMNV	2.22
EYFPISPEKKEEME	2.38
VYSPIQPRRRKDSS	3.35
LLAPNSPLKNTKSV	1.73
TKKPRPPVKSKPKH	2.11
TPPPVEPLKTKDLA	2.16
DQAPSLPPKPNTQL	3.67
PLIPDNPGRLLSSK	2.31
NNGPKIPQKNTIDS	1.87
NSPPTTPSKDECGP	2.47
MVLPPAPPKNSDPS	2.44
SKVPKAPTRETHIP	2.34
LSCPNVPGKPQVAY	1.89
FHDPILPRRTSTFL	4.62
VGNPEGPEKPNKKF	2.12
LPAPPPPPRRGPAP	3.10
SPSPLNPYRPHHNM	2.72
NIPPNSPGKLQLNT	1.89
GVYPSRPEKTYDYS	2.05
GGQPPVPVRMQPQP	3.29
GPAPPPPPRASRPT	3.37
LSLPRLPNKHHWPL	3.30
PPPPPPPPKPPLND	2.00
RGIPARPCKDDVMK	2.94
GPPPAMPARPTATT	3.03
PYPPSLPSRDLYEV	4.20
LPPPRSPNRPTLSD	4.08
VKEPSFPSRSAFSP	2.39
IAYPFYPFKKLGRQ	2.36
WISPDTPLKFPLNE	2.56
ISTPEVPKKKIKTG	2.35

TTVRPAKPPVVDRS	1.54
GTGSPARPPTPAQV	1.93
AAVPPPRPPIPEER	2.62
TRPPPIRPPFSHPI	2.91
TRPPPIRPPFSHPL	2.75
KRGRPGRPPSTNKK	1.78
PPWGPFRPPYSIFQ	1.75
ERPPPFKPPPPPIK	1.96
AAALPAKPPNLDAA	1.73
KPGSPYRPPVPRAP	2.30
PDQGPQRPPPEGLL	1.39
GSSSPSRPPGLSSN	1.86
EIMSPRKPPLFLTK	2.13
LLYLPLKPPNMFPS	1.45
VGTLPSKPPFRFGQ	2.23
PARPPAKPPEDEPD	1.75
QAGPPSRPPRYSSS	4.13
MEHSPQRPPRTEPT	4.16
SMGPPSKPPDEEHR	1.70
GLRRPEKPPYSYIA	1.54
HHHLPERPPFIPEG	1.26
QAVFPFKPPQRIEA	1.90
LRPVPGKPPGVDPL	1.82
EGNKPQRPPPPRRP	3.75
VPPRPPRPPETART	3.71
EEKVPEKPPTPKES	1.40
QSCPPQRPPAPACP	1.70
KPASPKKPPPGERS	1.75
LGPVPVRPPPGPEL	1.52
SPGPPYRPPSQRLR	2.74
NRWGPGRPPFRKAQ	1.83
RHASPARPPAARSQ	2.64
LPLPPARPPVLHSS	3.20
DIPGPTRPPGQVLS	1.88
RPALPGRPPQVAEQ	1.74
ALLVPVKPPTAWEG	0.38
YEEPPQKPPRFSQL	3.52
YKVSPGRPPGSIKA	1.69
LEIPPPKPPAPLLH	1.56
IRLPPARPPTPGRS	3.70
SKTPPQRPPPQGCL	2.29
GIRRPEKPPYSYIA	1.73
QDLRPLRPPAQTPQ	2.43
SISFPTRPPLQAQI	1.79
PPGKPERPPPQGGD	1.79
TPAPPLRPPDSLSR	2.65
PRRGPPRPPGLPYP	3.04
LLLPPPRPPHSDIT	2.94
MNGLPPRPPLPESL	1.76
GIKSPSKPPGPNRI	1.73
CCGRPGRPPGPAAA	1.86
WGLLPFRPPRPLPV	3.65
FSSVPSKPPMSLEP	1.48
PPPRPPRPPSPPPE	3.72
RRRSPPRPPSRPAR	4.33
ENESPLKPPGICMD	1.70
PRPLPPRPPAAQPR	3.44
QPDGPARPPGMAPS	1.80
PRPLPPRPPAAQPP	3.19
PLRLPPRPPGPRLA	3.69
APPLPVRPPKTSPG	3.65
FTDLPKKPPPPEVT	1.94
AAPPPAKPPPPPQP	1.62
NSSRPGRPPKRSLG	3.60
PGSLPSKPPPGPVP	1.68
TRPKPLKPPCDLSM	1.96
VTQLPARPPPALYT	1.59
EEVVPPRPPLPRSY	2.04
GDAPPPRPPPPVRP	3.10
LHGRPYRPPSPPPP	2.34
HLSSPARPPDNLSP	1.98
DTTSPSRPPGGPII	1.56
AAQPPQKPPASYFA	1.66
VYTPPPRPPPPWGS	2.26
LAELPGRPPIFWPG	1.73
TPLSPLKPPRMDTA	2.52
LSERPPRPPPPPPP	2.53
ASHPPPRPPKHPSV	2.93
PNLLPHRPPLLVFP	1.85
PPSGPLRPPATISP	2.02
QILSPPRPPTSPCF	1.88
GLSGPCRPPPPPQA	1.89
PSNPPPRPPAEARK	2.26
AKLFPGRPPVKKLL	1.97
RAPPPSRPPPRPSV	3.64
WCQSPQRPPPSTTH	1.87
AASSPARPPGLQRS	2.13
EEVPPHKPPTLPLD	1.46
SPVLPARPPPPPLS	2.60
LTWLPPKPPTAFDG	1.38
EHRPPEKPPRPPRP	3.99
HKGLPPRPPSHNRP	2.51
ASAPPQRPPSGPEG	1.33
PRPPPGKPPTRPGV	2.35
PRAVPARPPTRPRF	3.81
SPPVPPRPPSSGPP	3.10
QVDPPERPPSPPSD	1.96
YLQLPPRPPGTRAS	2.37
QVVGPHRPPALDDR	1.78
SHHPPDRPPDPFST	1.48
PPALPPKPPKPTTV	1.89
QPQGPPRPPQGDKP	1.92
KGPGPQKPPTEADK	1.78
PSLLPPRPPPALSL	1.92
LLPFPLRPPSPHCP	2.25
RMKRPFRPPPAHFQ	2.74
NVLVPSRPPRNPYD	3.67
TKDSPGKPPMAPPS	1.96
ALPRPLRPPLPPDP	2.74
PKISPQKPPRKDME	3.46
AGLRPPKPPRPASL	3.38
SSTLPQKPPYSFID	1.75
QDSPPPKPPRTRSC	4.31
ALTSPGRPPMGFTT	1.71
AAPFPDRPPAHLVS	1.68
AWARPPKPPTFGEF	1.34
SVSRPERPPESAAL	1.93
LRKRPLKPPAKKIM	1.96
GPPPPPRPPFGRPP	3.14
SKTPPQRPPPQGGP	2.10
RLAGPPRPPGHPRE	2.21
PPPLPQRPPPRLPP	3.34
IPERPLRPPSEKRA	4.04
VPPPPARPPPRSAR	4.17
VSYPPSKPPTYEEQ	1.75
QKTRPIKPPPDSQE	1.62
APPPPSRPPQPRPG	2.93
VTSKPRKPPTPPEK	2.20
HGRLPARPPFYAPA	1.53
RSSGPERPPHGLPL	1.60
GAGSPARPPSPRLD	2.53
NSFSPQKPPGHCSR	1.64
MLLLPPRPPHPRSS	3.48
TRGRPTRPPTRPPT	2.96
PPPPPPRPPSRPKL	4.14
GYTGPPRPPPYGYQ	1.79
VLPPPPRPPQRRCR	4.36
DKCPPPRPPSSPYP	1.76
VYKPPLKPPKGTRQ	1.91
PPEKPPRPPRPLHL	4.16
VTDLPKKPPPPEAT	1.71
PPPPPARPPARRAS	4.06
CVRGPLRPPVRCPA	3.70
LAYFPPRPPLPPSV	1.69
PRKLPSRPPPHYPG	2.81
GGQPPPRPPPGMPH	1.63
DVIIPAKPPVSFFS	1.20
QGGRPHRPPQGQPP	1.57
YLGKPSRPPHLLLE	1.64
HVQSPEKPPTSPRE	1.70
GSGSPVRPPVSTWG	1.51
AGPGPARPPVPPRP	3.75
GLGSPERPPKTSPG	2.09
FNDSPYKPPVTRCN	1.81
LAELPQKPPPGDLP	1.80
IIRPPVRPPDPPCR	3.51
VAEPPPRPPPPPPP	1.88
TRWPPSRPPPPCPF	2.56
PPPPPLRPPPPPPP	2.45
LYRFPGKPPTYVTD	1.59
TPAPPLRPPDSPSR	2.45
SGGRPLRPPHPVAK	2.86
GGPSPSRPPPSLFS	1.97
QEDSPRKPPNFPVP	1.99
TTTPPTKPPSFNLH	1.73
CLLSPLRPPGQRGL	2.60
PPPVPLRPPEHFIN	2.28
IEEVPPRPPSPPKK	2.35
DLLSPRRPPDDPWA	1.31
PPVRPARPPGPLVT	3.87
DFPKPKKPPPPAKA	1.52
YTVPPARPPPPLTK	2.32
KDPGPPRPPAGATQ	1.64
GSQAPARPPKPRPR	2.63
RQHLPERPRRCPLC	3.78
XSQHFGRPRREDHL	2.78
SPKCPGRPWKSVSE	2.36
VRYPISRPRRRCSP	2.95
DTALLPRPERRCSL	2.05
IAEALSRPGREACL	2.97
EALEIPRPLRALDQ	0.00
VNRPLVRPQKRLSS	2.40
GFPAPFRPSRKRSG	3.98
TPVSLGRPERYRRH	2.81
RQCQLQRPLRGFTW	3.08
NAALFPRPERYNPQ	2.84
RPEDPTRPSRFQQQ	3.26
RYLGITRPHKMLTT	2.01
RTPTPDRPPRSAWG	3.31
XSQHFGRPRRVDHE	2.57
GPSPPYRPEKLSSP	2.39
SCKNIYRPDKFLQC	1.53
XSQHFGRPRRVDHL	2.95
RPNPTQPRKPLDCK	2.32
YTLPLLPGKGGFRI	2.03
LIPPLHPLKKQHPK	1.03
DLQPLEPKKAAAST	2.08
ILSPGYPNKSFNNK	1.97
VVIPPVPSRYSDER	1.70
ALRPTGPDRGDGFD	2.12
SNSPQIPKKKVKDQ	2.84
SNDPIPPPRKVSDV	1.88
QDSPDMPEKECVYV	1.71
TNPPIDPIREANVM	2.14
EYHPIKPLRSSSST	1.75
ISTPPPPPKMAIPA	2.04
ELPPIKPPRPTSTT	1.74
PIVPREPLRNEPTD	2.80
ITLPRRPAKKIQLG	2.17
KLKPSLPNKLVGKY	0.96
QPKPGNPLRNARLN	2.24
DVYPHEPAKNGEGS	1.99
LRKPSAPKRMSSIS	2.08
GKVPVQPRKRKSIT	2.38
ELDPICPLKVPLTA	1.75
ERDPNAPKKPLTVF	1.88
NDDPVIPYKLSKDT	1.97
NSRPQQPERRRFNS	3.85
PPEPATPSKKSLTT	1.66
VQTPHVPDRPPSQL	1.73
GNTPDAPKRGFLGY	1.94
KRPPTAPQRKISLP	2.46
RRKPHSPNKKKPKE	2.17
SNNPSKPRKYKTKS	1.87
RSIPRGPDKNSLRV	2.36
IQHPFLPRKIPIIT	2.51
SGPPLLPPRNTMKN	2.76
NAPPYVPQRSKQPD	2.75
QRLPGEPGREQAPN	2.01
GRIPQLPNRDEMTA	1.89
MRAPPSPRKSKSGH	1.91
LIPPGFPTKNYPPG	1.60
KLGPDTPIKVAGPL	1.25
PNEPGAPSRLSFEN	2.02
SSLPLGPIKERSKQ	2.08
YILPKNPFRVPSEM	3.93
QNTPPLPPKPKSPH	2.01
DYLPPVPEKNAGKQ	1.68
VAPPPLPNRQLPNL	3.17
QIFPKKPLRRTVMS	2.36
IFGPSFPFRSHIDV	2.14
FPFPTPPDRVALSK	1.72
DEPPSFPKRWGWLK	1.89
RDKPSEPERTKDGS	1.87
SSTPIVPNRLKTVL	1.75
VTLPTGPAKIIWEK	1.98
LSKPSKPLKEIRFD	2.37
MALPRTPLKTSTEE	2.00
TDLPVNPPRDDKEW	1.94
HPDPADPAKELELN	1.86
NEYPSKPPKVKFPA	1.82
LSTPSAPAKINNYS	1.26
NAKPPLPIKAVTRP	3.31
QLLPNNPDKNNNGT	2.17
EVYPIEPPKVVCLK	1.69
LTCPFEPPKPKTKH	2.27
THAPTTPNRTSPNR	1.87
LENPSTPDKTEEDW	1.70
GTPPTTPLRTAQGI	1.99
EKFPKLPHKLVHML	1.75
SRKPPIPAKQHVKK	1.71
QLTPLLPDKYFDSA	1.70
IFPPLNPARTSLNS	1.86
YGPPTDPRKQKQAK	2.19
QLLPNVPDKVLVPG	1.94
VPEPSKPCRLLLVR	1.79
HGLPYKPSKLIRIF	1.90
TLIPPIPEKQSIKN	1.91
AMNPESPTRDQIFL	1.54
VPPPNVPKKDKREV	2.47
ALKPKTPSKAPRKR	2.39
VAKPKKPARPTMST	1.85
DSPPQSPRRSSNSS	2.49
SKDPNRPTKFAFES	1.67
TTVPTKPKKPHDIS	1.91
SNFPDFPLKKYLKN	1.77
SLSPKDPERARLLS	1.90
DSIPVDPEKVVAIV	1.70
YQYPQTPSKVDISI	1.56
SMYPGLPSRLEKEL	1.89
VQQPEDPLRVLGEY	1.39
LHVPRNPSKSRRGS	2.18
YDFPTVPARKFVQP	3.17
FVSPRIPSRIVKNG	2.95
LTSPKNPIKSVTKF	2.74
YTNPRAPGKLQDSH	2.18
NSMPNVPVRLKLHT	3.72
DYQPLSPPRELDPD	2.08
VTLPISPSRASISF	1.72
LRRPIFPGRDYRHQ	2.92
GSSPVSPDKRPSWK	1.84
SQSPKRPKKYPKKA	1.67
QGYPRQPQRPQRYH	3.87
GYDPTDPNRPKVTK	2.03
KLRPDLPVKNFVVK	2.38
KALPKEPKKLLPNS	2.32
ISRPVQPFKVGSIG	1.78
KHAPFIPVKPALEK	1.96
KFIPNKPYRQFNTW	1.49
SMEPHSPKKAKSVE	1.77
QMRPYTPLRSPNSR	3.46
SHLPLIPEKIKSLK	2.09
VSTPPVPKKFQEIF	1.79
ISNPSSPAKTKRDI	1.96
NKIPPKPRKVLQLL	1.80
TGFPPTPLKREGVG	0.88
EKLPETPDRSKCAC	1.84
VPSPGLPEKVNEKS	1.90
DLNPDAPPKPQLIP	1.98
SKFPELPSKVHNAV	2.02
LRDPVIPIKLAAEK	2.30
EIAPSLPSRNSIPA	1.97
VHDPNIPLRPKEII	1.36
SSRPTSPVRKARST	2.57
ATVPSAPYRKQIIS	1.48
KGAPFFPNKTFRKL	2.15
DLKPAYPGRSAHVV	1.32
SEEPTSPTRQVNPE	1.51
ETVPTEPTRYNYQN	2.05
FTAPPVPTKKKSKN	2.54
NMVPLNPNRIIPDE	1.50
QETPIIPKRIIHYS	3.68
LPAPHRPEKYKERF	1.95
RYKPTPPLKKRYDY	1.41
ASKPSVPPRNYFSK	3.05
TATPSSPKRMHSSI	1.71
NNNPVPPQKINQPP	1.33
VDFPIFPRRFAKVE	3.31
ESLPRTPTKGRSTQ	2.66
DFTPVEPSKMREYI	1.81
LPLPTLPDRQLFQT	2.10
MTAPLWPNKNEKNH	1.52
TQAPLAPSRNQENE	1.50
VTKPVVPKKTKKSA	2.22
FFHPAIPLKNAAPF	2.19
IFDPQDPYRRAQQF	1.73
QSPPRSPGRSPTRR	4.52
DYNPPPPSRNSPIR	2.29
KKDPNAPKRALSAY	1.76
RPPPLTPEKNLYLT	1.81
DLAPKWPKKTVSNE	1.68
SSYPMNPPKISFMQ	1.23
EKGPRMPSRGRPRP	2.60
TEAPMNPLKNREQM	0.28
QILPTKPVKIIEQN	1.48
EFCPTYPSKLFVPR	1.73
DYNPTIPPRSKDRP	4.55
YLDPFHPKRKFIGV	2.25
TSAPDIPPRSPNRN	4.82
AARPTRPDKARRLG	2.24
KTKPSIPPKKVELN	1.66
EANPDNPKKLDLKA	1.90
CIIPPLPDKKVFQY	1.60
DPLPYSPLKLYDQK	1.77
AGTPNVPTRRPILK	3.62
KSFPPPPLKPGQKT	2.05
RCSPWCPSRSHGAV	2.17
VHGPGIPMKSLEVA	1.65
SSNPDDPIRVTTPM	1.41
VLEPSKPYKKNPFM	2.07
KKDPFPPPKDLLVQ	2.01
LDYPKAPIKLTMKS	1.68
DVLPAGPVRSLEGA	1.39
DSDPNTPKKLSSPR	2.03
LQNPLGPERTFLIM	2.08
AIPPPVPNRPGGTT	1.00
PKDPCSPQRDEILT	2.14
DSGPQRPKRTRAKG	2.66
DVLPAGPVRSLEGV	1.45
VYVPINPPKELDQR	1.51
SLDPDRPEKDCVEE	1.92
TLQPGLPHKPLPSD	1.92
SAFPQSPIRAYGST	2.38
MPTPMTPVKVGALA	1.13
DGRPHSPLRARSAT	3.23
STSPKLPPRGKQRE	3.72
NINPKQPPKTSGEK	1.39
STPPTLPPRRIEDP	2.29
NTVPSTPSRSNSVL	1.66
INQPSTPKKLKRKS	2.18
HEHPCGPLKKLFSD	0.20
SSSPFIPKKRKPRS	1.88
SELPTRPSKSKTYN	2.14
HGVPVVPSREVTIN	2.15
GWKPLRPIKLISWD	1.77
RPTPRKPSRMATDH	1.90
NSRPQQPERRRFNS	3.39
PPEPATPSKKSLTT	1.42
VQTPHVPDRPPSQL	1.75
GNTPDAPKRGFLGY	1.44
KRPPTAPQRKISLP	1.71
RRKPHSPNKKKPKE	2.30
SNNPSKPRKYKTKS	1.55
RSIPRGPDKNSLRV	1.88
IQHPFLPRKIPIIT	2.28
SGPPLLPPRNTMKN	1.82
NAPPYVPQRSKQPD	2.30
QRLPGEPGREQAPN	1.15
GRIPQLPNRDEMTA	1.73
MRAPPSPRKSKSGH	2.23
LIPPGFPTKNYPPG	1.61
KLGPDTPIKVAGPL	1.23
PNEPGAPSRLSFEN	1.85
SSLPLGPIKERSKQ	1.29
YILPKNPFRVPSEM	2.58
QNTPPLPPKPKSPH	1.66
DYLPPVPEKNAGKQ	1.96
VAPPPLPNRQLPNL	1.86
QIFPKKPLRRTVMS	2.45
IFGPSFPFRSHIDV	1.50
FPFPTPPDRVALSK	2.01
DEPPSFPKRWGWLK	2.05
RDKPSEPERTKDGS	1.44
SSTPIVPNRLKTVL	1.03
VTLPTGPAKIIWEK	1.29
LSKPSKPLKEIRFD	1.80
MALPRTPLKTSTEE	2.00
TDLPVNPPRDDKEW	1.66
HPDPADPAKELELN	1.09
NEYPSKPPKVKFPA	1.74
LSTPSAPAKINNYS	1.58
NAKPPLPIKAVTRP	1.69
QLLPNNPDKNNNGT	1.72
EVYPIEPPKVVCLK	1.42
LTCPFEPPKPKTKH	1.53
THAPTTPNRTSPNR	1.16
LENPSTPDKTEEDW	1.72
GTPPTTPLRTAQGI	1.56
EKFPKLPHKLVHML	1.92
QLTPLLPDKYFDSA	1.30
IFPPLNPARTSLNS	1.50
YGPPTDPRKQKQAK	1.58
QLLPNVPDKVLVPG	1.69
VPEPSKPCRLLLVR	1.23
HGLPYKPSKLIRIF	1.78
TLIPPIPEKQSIKN	1.57
AMNPESPTRDQIFL	1.59
VPPPNVPKKDKREV	1.25
ALKPKTPSKAPRKR	2.01
VAKPKKPARPTMST	1.89
DSPPQSPRRSSNSS	1.19
SKDPNRPTKFAFES	1.58
TTVPTKPKKPHDIS	1.36
MTDPDAPSKTDHKW	1.54
SNFPDFPLKKYLKN	1.19
SLSPKDPERARLLS	1.58
DSIPVDPEKVVAIV	1.35
YQYPQTPSKVDISI	1.56
SMYPGLPSRLEKEL	2.07
VQQPEDPLRVLGEY	1.22
LHVPRNPSKSRRGS	2.37
YDFPTVPARKFVQP	0.92
FVSPRIPSRIVKNG	3.03
LTSPKNPIKSVTKF	1.41
YTNPRAPGKLQDSH	1.42
NSMPNVPVRLKLHT	3.44
DYQPLSPPRELDPD	0.94
VTLPISPSRASISF	1.52
LRRPIFPGRDYRHQ	2.37
GSSPVSPDKRPSWK	1.27
SQSPKRPKKYPKKA	1.56
QGYPRQPQRPQRYH	2.58
GYDPTDPNRPKVTK	1.72
KLRPDLPVKNFVVK	1.18
KALPKEPKKLLPNS	1.80
ISRPVQPFKVGSIG	1.90
KHAPFIPVKPALEK	1.44
KFIPNKPYRQFNTW	1.75
SMEPHSPKKAKSVE	1.86
QMRPYTPLRSPNSR	3.15
SHLPLIPEKIKSLK	1.75
VSTPPVPKKFQEIF	1.55
ISNPSSPAKTKRDI	1.69
NKIPPKPRKVLQLL	2.00
TGFPPTPLKREGVG	1.73
EKLPETPDRSKCAC	1.84
VPSPGLPEKVNEKS	1.41
DLNPDAPPKPQLIP	1.62
SKFPELPSKVHNAV	1.51
LRDPVIPIKLAAEK	1.46
EIAPSLPSRNSIPA	1.71
VHDPNIPLRPKEII	1.76
SSRPTSPVRKARST	1.86
ATVPSAPYRKQIIS	1.73
KGAPFFPNKTFRKL	1.81
DLKPAYPGRSAHVV	1.89
SEEPTSPTRQVNPE	1.42
ETVPTEPTRYNYQN	1.74
FTAPPVPTKKKSKN	1.65
NMVPLNPNRIIPDE	2.15
QETPIIPKRIIHYS	2.39
LPAPHRPEKYKERF	1.55
RYKPTPPLKKRYDY	1.92
ASKPSVPPRNYFSK	1.65
TATPSSPKRMHSSI	1.56
NNNPVPPQKINQPP	1.64
VDFPIFPRRFAKVE	2.13
ESLPRTPTKGRSTQ	1.42
DFTPVEPSKMREYI	1.78
DLPPKFPEKPVHLV	1.41
LPLPTLPDRQLFQT	1.69
MTAPLWPNKNEKNH	1.41
TQAPLAPSRNQENE	0.61
VTKPVVPKKTKKSA	1.28
FFHPAIPLKNAAPF	0.97
IFDPQDPYRRAQQF	1.71
QSPPRSPGRSPTRR	3.54
DYNPPPPSRNSPIR	1.49
KKDPNAPKRALSAY	1.29
RPPPLTPEKNLYLT	1.88
DLAPKWPKKTVSNE	1.16
SSYPMNPPKISFMQ	2.05
EKGPRMPSRGRPRP	2.21
TEAPMNPLKNREQM	1.75
QILPTKPVKIIEQN	1.56
EFCPTYPSKLFVPR	1.52
DYNPTIPPRSKDRP	4.05
YLDPFHPKRKFIGV	1.77
TSAPDIPPRSPNRN	4.56
AARPTRPDKARRLG	2.30
KTKPSIPPKKVELN	1.54
EANPDNPKKLDLKA	0.88
CIIPPLPDKKVFQY	1.69
DPLPYSPLKLYDQK	2.13
AGTPNVPTRRPILK	1.43
KSFPPPPLKPGQKT	2.26
RCSPWCPSRSHGAV	1.97
VHGPGIPMKSLEVA	1.70
SSNPDDPIRVTTPM	1.88
VLEPSKPYKKNPFM	1.92
KKDPFPPPKDLLVQ	1.69
LDYPKAPIKLTMKS	1.11
DVLPAGPVRSLEGA	1.66
DSDPNTPKKLSSPR	1.36
LQNPLGPERTFLIM	1.52
AIPPPVPNRPGGTT	1.38
PKDPCSPQRDEILT	2.19
DSGPQRPKRTRAKG	1.96
DVLPAGPVRSLEGV	1.64
VYVPINPPKELDQR	1.24
SLDPDRPEKDCVEE	1.66
TLQPGLPHKPLPSD	1.73
SAFPQSPIRAYGST	1.57
MPTPMTPVKVGALA	1.74
DGRPHSPLRARSAT	1.34
STSPKLPPRGKQRE	1.96
NINPKQPPKTSGEK	0.76
STPPTLPPRRIEDP	1.65
NTVPSTPSRSNSVL	1.67
INQPSTPKKLKRKS	1.35
SSSPFIPKKRKPRS	1.96
SELPTRPSKSKTYN	1.60
HGVPVVPSREVTIN	1.53
GWKPLRPIKLISWD	2.37
RPTPRKPSRMATDH	1.87
KWEPSTPQRKGPIL	1.63
APAPLDPFKTGGNN	1.83
RFNPAIPLRIYLKT	1.58
MSLPVTPVKSKVCS	1.58
PKEPRLPKRKVAVM	1.45
GVPPNFPQRMMPPP	1.93
QQLPLEPLKPTATG	1.43
SGPPPRPKKPSTLK	1.93
TAAPATPPRHICNN	1.62
SELPKSPYRQKPTQ	1.57
ILYPDIPGKNYFLT	1.67
AIEPGVPSRYSDET	1.36
LHDPLVPIKSYGLT	1.13
TPAPNLPEKTRLAL	1.58
GILPLAPLRPLDPL	1.49
QPLPTSPSKRFSSL	1.97
VKHPMKPFRLMLTD	2.31
SQKPQKPKKSAITK	1.87
QEKPLTPLKYTKLI	1.46
LIDPSTPDKAAKKD	1.30
DIHPSLPVRTRFAP	1.48
REEPPLPKRIRISK	1.82
LPVPQLPPKLLVYP	1.76
PPPPPVPAKLFGES	1.58
TTVRPAKPPVVDRS	2.91
GTGSPARPPTPAQV	3.38
AAVPPPRPPIPEER	3.63
TRPPPIRPPFSHPI	4.02
TRPPPIRPPFSHPL	3.94
KRGRPGRPPSTNKK	3.40
PPWGPFRPPYSIFQ	3.40
ERPPPFKPPPPPIK	3.43
AAALPAKPPNLDAA	1.86
KPGSPYRPPVPRAP	3.26
PDQGPQRPPPEGLL	1.79
GSSSPSRPPGLSSN	2.50
EIMSPRKPPLFLTK	3.23
LLYLPLKPPNMFPS	1.12
VGTLPSKPPFRFGQ	2.70
PARPPAKPPEDEPD	1.66
QAGPPSRPPRYSSS	3.88
MEHSPQRPPRTEPT	3.04
SMGPPSKPPDEEHR	1.65
GLRRPEKPPYSYIA	2.45
HHHLPERPPFIPEG	1.35
QAVFPFKPPQRIEA	1.80
LRPVPGKPPGVDPL	1.51
EGNKPQRPPPPRRP	4.26
VPPRPPRPPETART	3.76
EEKVPEKPPTPKES	1.33
QSCPPQRPPAPACP	3.60
KPASPKKPPPGERS	2.87
LGPVPVRPPPGPEL	1.91
SPGPPYRPPSQRLR	3.77
NRWGPGRPPFRKAQ	3.81
RHASPARPPAARSQ	3.67
LPLPPARPPVLHSS	3.56
DIPGPTRPPGQVLS	1.93
RPALPGRPPQVAEQ	2.70
ALLVPVKPPTAWEG	1.74
YEEPPQKPPRFSQL	2.12
YKVSPGRPPGSIKA	2.74
LEIPPPKPPAPLLH	2.85
IRLPPARPPTPGRS	4.30
SKTPPQRPPPQGCL	3.38
GIRRPEKPPYSYIA	2.94
QDLRPLRPPAQTPQ	2.19
SISFPTRPPLQAQI	2.21
PPGKPERPPPQGGD	2.49
TPAPPLRPPDSLSR	2.81
PRRGPPRPPGLPYP	3.90
LLLPPPRPPHSDIT	3.15
MNGLPPRPPLPESL	3.95
GIKSPSKPPGPNRI	3.86
CCGRPGRPPGPAAA	3.25
WGLLPFRPPRPLPV	3.56
FSSVPSKPPMSLEP	1.56
PPPRPPRPPSPPPE	3.97
RRRSPPRPPSRPAR	3.98
ENESPLKPPGICMD	1.35
PRPLPPRPPAAQPR	3.88
QPDGPARPPGMAPS	1.60
PRPLPPRPPAAQPP	3.49
PLRLPPRPPGPRLA	4.27
APPLPVRPPKTSPG	3.36
FTDLPKKPPPPEVT	1.77
AAPPPAKPPPPPQP	1.89
NSSRPGRPPKRSLG	4.27
PGSLPSKPPPGPVP	1.36
TRPKPLKPPCDLSM	2.65
VTQLPARPPPALYT	3.15
EEVVPPRPPLPRSY	3.92
GDAPPPRPPPPVRP	4.17
LHGRPYRPPSPPPP	2.39
HLSSPARPPDNLSP	1.89
DTTSPSRPPGGPII	1.86
AAQPPQKPPASYFA	2.10
VYTPPPRPPPPWGS	3.62
LAELPGRPPIFWPG	1.99
TPLSPLKPPRMDTA	1.75
LSERPPRPPPPPPP	2.97
ASHPPPRPPKHPSV	3.73
PNLLPHRPPLLVFP	2.28
PPSGPLRPPATISP	2.10
QILSPPRPPTSPCF	3.68
GLSGPCRPPPPPQA	3.47
PSNPPPRPPAEARK	3.00
AKLFPGRPPVKKLL	4.13
RAPPPSRPPPRPSV	4.04
WCQSPQRPPPSTTH	2.75
AASSPARPPGLQRS	3.60
EEVPPHKPPTLPLD	1.78
SPVLPARPPPPPLS	3.61
LTWLPPKPPTAFDG	1.86
EHRPPEKPPRPPRP	3.99
HKGLPPRPPSHNRP	3.70
ASAPPQRPPSGPEG	2.64
PRPPPGKPPTRPGV	3.19
PRAVPARPPTRPRF	4.25
SPPVPPRPPSSGPP	3.27
QVDPPERPPSPPSD	2.51
YLQLPPRPPGTRAS	3.09
QVVGPHRPPALDDR	1.63
SHHPPDRPPDPFST	3.14
PPALPPKPPKPTTV	3.54
QPQGPPRPPQGDKP	1.97
KGPGPQKPPTEADK	1.92
PSLLPPRPPPALSL	3.76
LLPFPLRPPSPHCP	3.99
RMKRPFRPPPAHFQ	3.71
NVLVPSRPPRNPYD	3.47
TKDSPGKPPMAPPS	1.91
ALPRPLRPPLPPDP	3.54
PKISPQKPPRKDME	2.69
AGLRPPKPPRPASL	4.09
SSTLPQKPPYSFID	1.44
QDSPPPKPPRTRSC	3.46
ALTSPGRPPMGFTT	2.42
AAPFPDRPPAHLVS	1.96
AWARPPKPPTFGEF	2.85
SVSRPERPPESAAL	2.01
LRKRPLKPPAKKIM	3.79
GPPPPPRPPFGRPP	3.73
SKTPPQRPPPQGGP	3.16
RLAGPPRPPGHPRE	3.43
PPPLPQRPPPRLPP	3.44
IPERPLRPPSEKRA	3.88
VPPPPARPPPRSAR	4.07
VSYPPSKPPTYEEQ	1.63
QKTRPIKPPPDSQE	1.56
APPPPSRPPQPRPG	3.59
VTSKPRKPPTPPEK	3.19
HGRLPARPPFYAPA	2.89
RSSGPERPPHGLPL	2.88
GAGSPARPPSPRLD	4.18
NSFSPQKPPGHCSR	2.70
MLLLPPRPPHPRSS	4.06
TRGRPTRPPTRPPT	3.92
PPPPPPRPPSRPKL	4.13
GYTGPPRPPPYGYQ	2.76
VLPPPPRPPQRRCR	4.34
DKCPPPRPPSSPYP	3.61
VYKPPLKPPKGTRQ	2.89
PPEKPPRPPRPLHL	4.47
VTDLPKKPPPPEAT	1.76
PPPPPARPPARRAS	4.12
CVRGPLRPPVRCPA	3.74
LAYFPPRPPLPPSV	3.34
PRKLPSRPPPHYPG	3.37
GGQPPPRPPPGMPH	2.73
DVIIPAKPPVSFFS	1.73
QGGRPHRPPQGQPP	1.71
YLGKPSRPPHLLLE	3.25
HVQSPEKPPTSPRE	1.70
GSGSPVRPPVSTWG	1.22
AGPGPARPPVPPRP	3.53
GLGSPERPPKTSPG	2.71
FNDSPYKPPVTRCN	1.97
LAELPQKPPPGDLP	1.57
IIRPPVRPPDPPCR	3.54
VAEPPPRPPPPPPP	2.78
TRWPPSRPPPPCPF	3.89
PPPPPLRPPPPPPP	3.06
LYRFPGKPPTYVTD	2.19
TPAPPLRPPDSPSR	3.31
SGGRPLRPPHPVAK	3.79
GGPSPSRPPPSLFS	3.13
QEDSPRKPPNFPVP	2.15
TTTPPTKPPSFNLH	2.81
CLLSPLRPPGQRGL	2.51
PPPVPLRPPEHFIN	2.64
IEEVPPRPPSPPKK	3.63
DLLSPRRPPDDPWA	1.92
PPVRPARPPGPLVT	3.82
DFPKPKKPPPPAKA	3.92
YTVPPARPPPPLTK	3.96
KDPGPPRPPAGATQ	2.36
GSQAPARPPKPRPR	4.80
RQHLPERPRRCPLC	4.04
XSQHFGRPRREDHL	2.44
SPKCPGRPWKSVSE	2.07
VRYPISRPRRRCSP	2.98
DTALLPRPERRCSL	1.82
IAEALSRPGREACL	2.15
VNRPLVRPQKRLSS	3.80
GFPAPFRPSRKRSG	4.01
TPVSLGRPERYRRH	2.60
RQCQLQRPLRGFTW	2.37
NAALFPRPERYNPQ	1.80
RPEDPTRPSRFQQQ	2.17
RYLGITRPHKMLTT	2.36
RTPTPDRPPRSAWG	2.60
XSQHFGRPRRVDHE	2.09
GPSPPYRPEKLSSP	2.83
SCKNIYRPDKFLQC	1.80
XSQHFGRPRRVDHL	2.31
CAGPPSRPQRPFCA	3.38
IEFDLSKPFTPFQQ	2.13
KELIYKQPSGPGLV	1.90
LEDALHVPTNPEIL	2.28
ELRDLDIPLVPSPK	1.88
SLISMETPEQPHQQ	2.17
YLNNLAEPLIPLSL	2.35
QASMLRRPSLPHID	3.04
PPPPLPPPLFPSSS	4.59
SPKKLASPRTPNAN	2.44
VLKTLAFPINPSDI	2.19
SFTPFNRPSDPSNG	2.32
QFSPMDDPKSPTPM	2.08
HFLAYTNPRAPGKL	2.05
TPSNMGSPGSPVNV	2.19
VPLPFGIPPFPMFP	4.06
HPYMMVYPMSPPPP	2.75
YFKNMSNPKIPATK	2.11
SFVGFPKPLLPRNH	2.31
IERVWNGPVEPSDE	2.26
GAPPLPVPNGPAVG	2.35
SPVNLLKPFNPTLP	2.09
VLKGFYSPSAPSYD	2.46
GFDGYMQPIIPGKT	2.20
VLFNLDHPMVPENL	2.34
NEKLHETPHLPSPV	2.04
AFHFWRPPIAPAEM	3.29
AAPKHAPPPVPNET	5.04
FFNRYPNPYSPHVL	2.14
NASAMPAPYMPYYY	2.35
SPFSLFIPTIPATT	2.18
DPNAYMQPQVPVQM	2.21
FASQLRKPRAPMRN	2.66
SENRLRTPLKPLVN	2.44
DPSLYQIPEPPRGG	1.99
SAYYLGLPVTPEDM	2.33
DFSSYGPPGTPGYD	2.04
ILQELSLPSRPVMP	1.72
EAIKLGVPKGPLFA	2.12
VFSLMNCPAYPDVQ	2.19
LFKRLPVPAPPMDS	2.16
TLPPLSSPMNPNGH	2.40
FFPIMSLPVTPVKS	2.09
EETEMLAPKLPEEN	1.91
NEGDLYPPEQPGVD	1.90
QELRFLVPLVPLLV	2.08
FWKIHLSPPLPSFK	2.98
NASLLRDPSAPYAH	2.13
REVDLPIPAQPGPP	2.06
EPIAHLFPPGPKNP	2.55
HPSGLNSPKLPPLP	2.66
FPFYLSFPFCPSFP	2.88
LELLLSLPTGPESK	2.16
DLKEFNGPEPPIKR	2.10
SFRKLASPSNPNFF	2.32
EPPSWKPPDLPIRL	4.94
HPHPHPHPHTPTHT	2.38
IEPDFIEPDAPLAA	1.79
PPLDFEQPRLPSTA	2.28
RLTNFEVPSLPTFH	2.50
VAVPFTLPVIPYDL	2.38
QELPMEVPKEPAPA	2.14
QLIGFGNPKNPFAL	1.66
IAECLGVPWEPVDL	2.16
DELLLMSPELPADL	1.20
SLFPLKTPISPGTS	2.38
MASGLLPPIQPGFD	2.35
TFSIYSIPSKPGEC	2.36
VEVIMHSPSDPEYA	2.18
GWPVYNYPMQPTTE	2.23
FAYSLGLPGAPKIK	1.88
GLRWYRSPIYPYLY	2.67
RLWLYAAPKRPKTF	2.93
SENYLVKPTIPGTT	1.79
GEIMFPAPPLPIAN	3.44
TEGKYAPPSQPSMA	2.35
KLEDLNEPLSPWIS	1.85
FFTIWFIPLVPVFW	2.60
GLLTYKGPKVPYQK	2.18
AELFLRRPIFPGRD	2.33
VPNRWATPLDPSIL	2.10
KFPLMDKPSIPTLR	2.64
SFVHLTEPSQPLSD	2.00
ALGFYRAPGSPAPA	2.00
AFHELGIPVIPNPS	2.29
LEFSHYAPTAPHVQ	2.01
KLNCLQIPHLPPSV	2.54
SFTRMPLPTPPIEG	2.46
IATMYPIPAYPDLL	1.99
HFNDLQSPAQPQQS	1.97
DLMALKYPYFPTKM	2.14
YEKALHFPAGPKST	2.17
KEQNWKHPLLPLLK	2.51
LPEELMRPKVPELL	2.16
SPSTHAAPVTPIIS	1.73
HAWDMRSPHRPFYS	2.91
EWPGLDPPYIPLDF	1.80
GLHDYTSPASPKQT	2.11
SELTFIAPTQPVST	2.04
TAAELQAPPPPPSS	2.50
AAVRLDQPILPQND	2.02
GWEPFSLPKDPKVV	1.88
SETSHHSPSAPFQR	2.41
NPGSMSMPGVPTSM	2.28
YLLELPDPLIPNDV	2.21
SFDYLFHPFVPCTI	1.36
SPKPFSKPQEPVKA	2.56
SLAVYNIPLFPDDP	1.96
NAIAMALPNEPYPA	1.99
PLFGLSIPKTPTLK	2.09
DFRLHYYPKDPSNV	1.89
YLPHMSRPLGPDEV	2.24
GLDALNIPHKPKQL	2.22
DLLGLSTPQSPTNS	1.96
WLLPYSLPMHPGRW	2.85
KESKFGTPEKPLSD	2.40
GWVVYPPPPKPSYN	2.66
DARPFLQPVDPVKL	2.25
LLLPLLLPPLPSVK	3.81
DLSYYLRPIKPFNT	2.21
VEPEWYLPILPMIL	2.37
FLALFSDPARPFLT	2.65
KLWDLSKPSKPLKE	2.37
MFELLCHPKVPVGM	2.43
PPPPMGQPKDPKFE	2.39
TLEWLLTPYTPGDD	2.21
VLIKFVIPVYPSLP	2.75
SPSTMDFPKLPSFQ	2.67
NALGLDPPPSPEDP	2.38
EPATLPLPGTPTLA	1.79
LPTGYYEPMKPSSL	2.22
HFYEYVYPSLPFIE	2.18
GAGGLGCPALPYLA	2.04
ALDYLIPPPMPKNQ	2.12
PEDFLPPPPKPHFA	2.10
KPSVMKLPPSPKQP	2.58
LLISHLFPTNPEFS	2.62
VFLRLISPTRPSLE	3.21
VARPLSVPGSPRDL	2.27
QPAQLQEPQEPLST	2.10
PEVLMDQPYSPLSD	1.90
YLNSLSEPLVPLAL	2.12
MLFSWPYPEAPIEG	2.10
KPPSYPSPAQPPKS	4.08
ELLCFLSPGKPSKG	1.76
GPEFWISPDTPLKF	1.94
SATGWLKPAVPIQW	1.98
NEIELLRPRSPVQK	2.10
ILCAYNSPKVPADI	1.91
DFPLYKSPAFPSSC	2.24
AELMLRIPYLPGQN	2.37
HLTDLSPPGTPPTM	2.42
NEDTFLPPPPPPPS	4.31
LAKDLIVPRRPEWN	5.07
APKYYVPPGIPTND	4.02
LPFMYLLPRFPSHI	2.66
QENPYIIPLPPSST	2.38
VPAILMTPICPRSL	2.14
SPRCLPTPILPSMS	2.29
VPDQLLTPLQPYTI	2.23
EEMGMEEPQAPEAF	2.11
IARSFTIPFLPKIP	2.31
SLDNFYNPLGPEDR	2.15
ALKEMTHPIKPSAQ	2.42
MFTYFQEPPFPDLL	2.38
KLPKLNVPLNPKGK	2.48
GESHYKPPSTPKIN	2.86
PETRMDLPFSPEVN	2.11
FLPVFNLPSNPALF	2.19
PLAPLPPPPPPSVA	3.68
KEFRFIFPLQPLFI	2.87
YEQTMKSPKYPSTY	2.14
KLGRFTNPQQPRED	2.36
VLSKFIKPKNPSLV	2.50
AADSHAIPVDPPSY	1.58
PPPPPPPPPPVPA	4.98
TPPPPPLPSVLSS	4.41
PAPPPPPPPPPPP	4.73
LPAPPPPPRRGPA	4.64
APAPPPPPQMPAT	3.89
VDPPFMPFRDAGYS	1.37
VKEPSFPSRSAFSP	1.77
IAYPFYPFKKLGRQ	1.21
WISPDTPLKFPLNE	2.23
QTSPGQPRKGQNGS	1.81
WNDPSDPTKGFQYL	1.69
IAHPVRPHRAITMT	1.94
SPSPSTPTKSGKMR	1.55
GLEPNTPLKSIKVD	1.77
GNVPHSPVRFADTV	1.56
ASMPRVPLKIVLKD	2.76
PPVPKAPSRRSSSL	3.22
TDGPIDPFKEPKTS	1.53
SNSPISPHKLIYKN	1.71
RNEPVKPFRNIDLK	1.59
KLEPENPQKPSNTH	1.20
HLDPEAPDKLLENS	1.46
VSPPFFPEKELNNR	1.51
PNSPTPPLKSKVSR	1.40
VCPPSFPSRRCETC	3.24
TRRPTNPFRVISVS	2.34
YYGPNSPLKLDEEN	1.70
IINPDTPHRRIFNQ	2.20
EYSPLVPSKMCADK	2.31
FKMPKKPQRSKEEV	2.22
TGRPDKPFRALLVD	2.90
IIFPTSPTKLTFSN	1.93
PIMPTLPPRPYITI	3.74
RLIPTSPQKTNINT	1.87
ATRPSSPMRHKSIS	3.07
EILPVKPTKKSSKK	1.81
FRFPRLPQKKKEEL	3.08
LRIPETPVKKSPLV	0.20
RPNPTQPRKPLDCK	2.21
YTLPLLPGKGGFRI	1.45
LIPPLHPLKKQHPK	1.55
DLQPLEPKKAAAST	1.48
ILSPGYPNKSFNNK	2.00
VVIPPVPSRYSDER	3.27
ALRPTGPDRGDGFD	1.78
SNSPQIPKKKVKDQ	1.54
SNDPIPPPRKVSDV	2.21
QDSPDMPEKECVYV	0.67
TNPPIDPIREANVM	1.44
EYHPIKPLRSSSST	1.68
ISTPPPPPKMAIPA	1.42
ELPPIKPPRPTSTT	3.28
PIVPREPLRNEPTD	4.54
ITLPRRPAKKIQLG	4.14
KLKPSLPNKLVGKY	1.66
QPKPGNPLRNARLN	1.69
DVYPHEPAKNGEGS	1.65
LRKPSAPKRMSSIS	2.44
GKVPVQPRKRKSIT	1.86
ELDPICPLKVPLTA	1.60
ERDPNAPKKPLTVF	1.78
NDDPVIPYKLSKDT	1.74
NSRPQQPERRRFNS	3.59
PPEPATPSKKSLTT	1.38
VQTPHVPDRPPSQL	1.72
GNTPDAPKRGFLGY	1.57
KRPPTAPQRKISLP	2.78
RRKPHSPNKKKPKE	1.59
SNNPSKPRKYKTKS	1.63
RSIPRGPDKNSLRV	2.47
IQHPFLPRKIPIIT	1.54
SGPPLLPPRNTMKN	4.41
NAPPYVPQRSKQPD	4.16
QRLPGEPGREQAPN	0.32
GRIPQLPNRDEMTA	4.08
MRAPPSPRKSKSGH	2.42
KLGPDTPIKVAGPL	1.82
PNEPGAPSRLSFEN	1.72
SSLPLGPIKERSKQ	1.19
YILPKNPFRVPSEM	2.30
QNTPPLPPKPKSPH	3.02
DYLPPVPEKNAGKQ	1.63
VAPPPLPNRQLPNL	4.51
QIFPKKPLRRTVMS	2.87
IFGPSFPFRSHIDV	2.59
FPFPTPPDRVALSK	2.06
DEPPSFPKRWGWLK	1.80
RDKPSEPERTKDGS	1.38
SSTPIVPNRLKTVL	3.17
VTLPTGPAKIIWEK	1.38
LSKPSKPLKEIRFD	1.92
MALPRTPLKTSTEE	2.53
TDLPVNPPRDDKEW	1.75
HPDPADPAKELELN	1.70
NEYPSKPPKVKFPA	1.78
LSTPSAPAKINNYS	1.49
NAKPPLPIKAVTRP	1.52
EVYPIEPPKVVCLK	0.95
LTCPFEPPKPKTKH	0.91
THAPTTPNRTSPNR	2.74
LENPSTPDKTEEDW	1.44
GTPPTTPLRTAQGI	2.29
EKFPKLPHKLVHML	1.52
SRKPPIPAKQHVKK	1.17
QLTPLLPDKYFDSA	2.02
IFPPLNPARTSLNS	2.13
YGPPTDPRKQKQAK	1.80
QLLPNVPDKVLVPG	1.83
VPEPSKPCRLLLVR	1.36
HGLPYKPSKLIRIF	1.83
TLIPPIPEKQSIKN	1.58
AMNPESPTRDQIFL	1.80
VPPPNVPKKDKREV	2.09
ALKPKTPSKAPRKR	2.48
VAKPKKPARPTMST	1.94
DSPPQSPRRSSNSS	1.62
SKDPNRPTKFAFES	1.58
TTVPTKPKKPHDIS	1.26
MTDPDAPSKTDHKW	1.51
SNFPDFPLKKYLKN	1.25
SLSPKDPERARLLS	1.90
DSIPVDPEKVVAIV	1.58
YQYPQTPSKVDISI	1.87
SMYPGLPSRLEKEL	2.91
VQQPEDPLRVLGEY	1.57
LHVPRNPSKSRRGS	2.11
YDFPTVPARKFVQP	2.40
FVSPRIPSRIVKNG	4.01
LTSPKNPIKSVTKF	1.98
YTNPRAPGKLQDSH	1.81
NSMPNVPVRLKLHT	3.39
DYQPLSPPRELDPD	2.51
VTLPISPSRASISF	1.41
LRRPIFPGRDYRHQ	2.98
GSSPVSPDKRPSWK	1.70
SQSPKRPKKYPKKA	1.76
QGYPRQPQRPQRYH	2.79
GYDPTDPNRPKVTK	1.55
KLRPDLPVKNFVVK	1.94
KALPKEPKKLLPNS	1.65
ISRPVQPFKVGSIG	1.36
KHAPFIPVKPALEK	1.34
KFIPNKPYRQFNTW	1.71
SMEPHSPKKAKSVE	1.93
QMRPYTPLRSPNSR	3.06
SHLPLIPEKIKSLK	1.70
VSTPPVPKKFQEIF	1.73
ISNPSSPAKTKRDI	1.25
NKIPPKPRKVLQLL	2.27
TGFPPTPLKREGVG	1.76
EKLPETPDRSKCAC	1.83
DLNPDAPPKPQLIP	1.04
SKFPELPSKVHNAV	1.31
LRDPVIPIKLAAEK	0.34
EIAPSLPSRNSIPA	4.27
VHDPNIPLRPKEII	1.71
SSRPTSPVRKARST	1.99
ATVPSAPYRKQIIS	2.16
KGAPFFPNKTFRKL	1.92
DLKPAYPGRSAHVV	1.50
SEEPTSPTRQVNPE	1.61
ETVPTEPTRYNYQN	1.43
FTAPPVPTKKKSKN	1.94
NMVPLNPNRIIPDE	2.53
QETPIIPKRIIHYS	2.80
LPAPHRPEKYKERF	1.46
RYKPTPPLKKRYDY	1.71
ASKPSVPPRNYFSK	3.59
TATPSSPKRMHSSI	1.91
NNNPVPPQKINQPP	1.67
VDFPIFPRRFAKVE	2.71
ESLPRTPTKGRSTQ	1.54
DFTPVEPSKMREYI	2.44
DLPPKFPEKPVHLV	1.96
LPLPTLPDRQLFQT	3.59
MTAPLWPNKNEKNH	1.74
TQAPLAPSRNQENE	1.58
VTKPVVPKKTKKSA	0.00
FFHPAIPLKNAAPF	1.35
IFDPQDPYRRAQQF	1.72
QSPPRSPGRSPTRR	2.96
DYNPPPPSRNSPIR	1.00
KKDPNAPKRALSAY	1.40
RPPPLTPEKNLYLT	1.71
DLAPKWPKKTVSNE	0.86
SSYPMNPPKISFMQ	1.82
EKGPRMPSRGRPRP	1.86
TEAPMNPLKNREQM	1.33
QILPTKPVKIIEQN	1.16
EFCPTYPSKLFVPR	1.84
DYNPTIPPRSKDRP	3.49
YLDPFHPKRKFIGV	1.76
TSAPDIPPRSPNRN	4.43
AARPTRPDKARRLG	2.04
KTKPSIPPKKVELN	1.44
EANPDNPKKLDLKA	1.66
CIIPPLPDKKVFQY	1.35
DPLPYSPLKLYDQK	1.80
YKIPVVPPRETYFA	2.29
GAPPTTPSRPRTTT	1.47
VHDPNIPLRPKEII	2.11
RIPPTVPSKIIDVV	2.72
LQIPPLPLKLLTPV	1.68
VFSPTNPLRIKIAR	2.01
LTLPFGPVKSIWLS	1.99
LPPPIIPLRKYGHN	2.04
FPYPGNPLKIINYE	2.33
EYFPISPEKKEEME	1.75
SKFPELPSKVHNAV	2.09
QSQPPRPPRPAANL	2.11
SSQPRPPPRPQQNP	1.92
VHGPGVPVKTLEIA	1.94
YILPKNPFRVPSEM	2.46
LRSPNTPRRLRKTL	2.48
ALVPFAPFKLLKDR	2.11
ARDPILPYRLVKDR	2.07
FHDPILPRRTSTFL	2.44
SKIPMLPSRRTMAG	3.05
QEKPLTPLKYTKLI	2.16
NIQPNIPLRSYRYL	2.07
THAPTTPNRTSPNR	1.78
PLPPIPPTKSKTSP	2.95
LVQPNVPEKHNMST	2.26
VTLPTGPAKIIWEK	2.13
RNEPVKPFRNIDLK	1.94
IFPPLNPARTSLNS	1.87
YSLPMHPGRWWVHT	2.66
MLIPNFPNKKLKLN	2.62
ENAPANPSKALKVK	1.84
DSIPLEPKKAKHLM	2.12
TEAPMNPLKNREQM	2.12
KFHPPEPYKGKGIY	2.38
LLPPTDPFRRKHEQ	2.19
IFDPQDPYRRAQQF	1.69
SSTPIVPNRLKTVL	2.21
SDLPSSPIKAPNVE	2.28
NDDPVIPYKLSKDT	2.30
ENVPLYPSRRNIWE	2.25
SSTPTTPERPKRKS	2.33
SELPKSPYRQKPTQ	2.30
LSLPRLPNKHHWPL	3.27
LRSPNTPRRLRKTL	2.33
LIMPIYPYRLVSTI	2.53
AFVPTNPWRSSMSL	2.79
LDYPKAPIKLTMKS	2.24
WKPPDLPIRLRKRP	3.56
EIPPPLPPKIMVHS	3.71
SRKPPIPAKQHVKK	3.00
TRRPHHPLKQQSTN	2.25
IEVPQSPTKNRVKV	2.15
VVSPSHPFKQLQKI	2.17
SNNPSKPRKYKTKS	2.52
VENPFLPAKEERTG	2.20
PNEPGAPSRLSFEN	1.54
SSLPLGPIKERSKQ	1.89
YILPKNPFRVPSEM	2.91
QNTPPLPPKPKSPH	4.59
DYLPPVPEKNAGKQ	2.83
VAPPPLPNRQLPNL	4.51
QIFPKKPLRRTVMS	4.55
IFGPSFPFRSHIDV	3.98
FPFPTPPDRVALSK	2.92
DEPPSFPKRWGWLK	3.98
RDKPSEPERTKDGS	2.05
SSTPIVPNRLKTVL	3.82
VTLPTGPAKIIWEK	1.58
LSKPSKPLKEIRFD	2.77
MALPRTPLKTSTEE	3.05
TDLPVNPPRDDKEW	1.98
NEYPSKPPKVKFPA	2.62
LSTPSAPAKINNYS	2.24
NAKPPLPIKAVTRP	3.27
QLLPNNPDKNNNGT	1.70
LTCPFEPPKPKTKH	1.91
THAPTTPNRTSPNR	4.17
LENPSTPDKTEEDW	1.79
GTPPTTPLRTAQGI	3.76
EKFPKLPHKLVHML	3.90
SRKPPIPAKQHVKK	3.69
QLTPLLPDKYFDSA	2.71
IFPPLNPARTSLNS	3.71
YGPPTDPRKQKQAK	2.24
QLLPNVPDKVLVPG	1.84
VPEPSKPCRLLLVR	3.08
HGLPYKPSKLIRIF	2.87
TLIPPIPEKQSIKN	2.41
AMNPESPTRDQIFL	2.81
VPPPNVPKKDKREV	2.99
ALKPKTPSKAPRKR	3.31
VAKPKKPARPTMST	3.96
DSPPQSPRRSSNSS	4.20
SKDPNRPTKFAFES	2.21
TTVPTKPKKPHDIS	3.19
MTDPDAPSKTDHKW	1.75
SNFPDFPLKKYLKN	2.58
SLSPKDPERARLLS	1.99
YQYPQTPSKVDISI	2.15
SMYPGLPSRLEKEL	3.79
VQQPEDPLRVLGEY	1.66
LHVPRNPSKSRRGS	3.10
YDFPTVPARKFVQP	2.91
FVSPRIPSRIVKNG	4.53
LTSPKNPIKSVTKF	2.36
YTNPRAPGKLQDSH	2.68
NSMPNVPVRLKLHT	4.31
DYQPLSPPRELDPD	2.70
LRRPIFPGRDYRHQ	3.90
GSSPVSPDKRPSWK	2.35
SQSPKRPKKYPKKA	2.80
QGYPRQPQRPQRYH	4.12
KLRPDLPVKNFVVK	4.03
KALPKEPKKLLPNS	2.45
ISRPVQPFKVGSIG	3.05
KHAPFIPVKPALEK	2.96
KFIPNKPYRQFNTW	3.09
SMEPHSPKKAKSVE	3.09
QMRPYTPLRSPNSR	3.65
SHLPLIPEKIKSLK	2.89
VSTPPVPKKFQEIF	2.84
ISNPSSPAKTKRDI	2.03
NKIPPKPRKVLQLL	3.24
TGFPPTPLKREGVG	3.24
EKLPETPDRSKCAC	4.09
VPSPGLPEKVNEKS	2.15
DLNPDAPPKPQLIP	1.95
SKFPELPSKVHNAV	3.07
LRDPVIPIKLAAEK	1.83
EIAPSLPSRNSIPA	4.55
VHDPNIPLRPKEII	2.75
SSRPTSPVRKARST	3.25
ATVPSAPYRKQIIS	3.28
KGAPFFPNKTFRKL	3.00
DLKPAYPGRSAHVV	3.95
ETVPTEPTRYNYQN	1.40
FTAPPVPTKKKSKN	3.96
NMVPLNPNRIIPDE	3.04
QETPIIPKRIIHYS	4.18
LPAPHRPEKYKERF	3.09
RYKPTPPLKKRYDY	3.33
ASKPSVPPRNYFSK	4.30
TATPSSPKRMHSSI	2.73
NNNPVPPQKINQPP	2.33
VDFPIFPRRFAKVE	4.21
ESLPRTPTKGRSTQ	3.34
DFTPVEPSKMREYI	1.18
DLPPKFPEKPVHLV	4.01
LPLPTLPDRQLFQT	4.18
MTAPLWPNKNEKNH	2.04
TQAPLAPSRNQENE	3.08
VTKPVVPKKTKKSA	2.93
FFHPAIPLKNAAPF	2.47
IFDPQDPYRRAQQF	1.78
QSPPRSPGRSPTRR	3.28
DYNPPPPSRNSPIR	2.94
KKDPNAPKRALSAY	2.46
RPPPLTPEKNLYLT	2.58
DLAPKWPKKTVSNE	3.20
SSYPMNPPKISFMQ	2.64
EKGPRMPSRGRPRP	3.71
TEAPMNPLKNREQM	2.60
QILPTKPVKIIEQN	2.26
EFCPTYPSKLFVPR	2.90
DYNPTIPPRSKDRP	3.83
YLDPFHPKRKFIGV	1.95
TSAPDIPPRSPNRN	4.16
AARPTRPDKARRLG	3.70
KTKPSIPPKKVELN	3.28
EANPDNPKKLDLKA	2.41
CIIPPLPDKKVFQY	3.84
DPLPYSPLKLYDQK	2.07
AGTPNVPTRRPILK	4.39
KSFPPPPLKPGQKT	2.34
RCSPWCPSRSHGAV	3.37
VHGPGIPMKSLEVA	1.52
SSNPDDPIRVTTPM	1.93
VLEPSKPYKKNPFM	2.38
KKDPFPPPKDLLVQ	2.52
LDYPKAPIKLTMKS	2.73
DVLPAGPVRSLEGA	2.14
DSDPNTPKKLSSPR	1.45
LQNPLGPERTFLIM	2.29
AIPPPVPNRPGGTT	4.05
PKDPCSPQRDEILT	0.79
DSGPQRPKRTRAKG	4.59
DVLPAGPVRSLEGV	0.60
VYVPINPPKELDQR	2.47
SLDPDRPEKDCVEE	2.10
TLQPGLPHKPLPSD	2.47
SAFPQSPIRAYGST	2.27
MPTPMTPVKVGALA	2.46
DGRPHSPLRARSAT	3.18
STSPKLPPRGKQRE	4.31
NINPKQPPKTSGEK	2.32
STPPTLPPRRIEDP	4.44
NTVPSTPSRSNSVL	4.42
INQPSTPKKLKRKS	3.39
HEHPCGPLKKLFSD	2.22
SSSPFIPKKRKPRS	3.29
SELPTRPSKSKTYN	3.66
HGVPVVPSREVTIN	2.74
GWKPLRPIKLISWD	4.08
RPTPRKPSRMATDH	4.22
KWEPSTPQRKGPIL	3.39
APAPLDPFKTGGNN	1.55
RFNPAIPLRIYLKT	3.48
MSLPVTPVKSKVCS	3.33
PKEPRLPKRKVAVM	4.58
GVPPNFPQRMMPPP	3.27
QQLPLEPLKPTATG	2.41
SGPPPRPKKPSTLK	3.42
TAAPATPPRHICNN	4.48
SELPKSPYRQKPTQ	2.64
ILYPDIPGKNYFLT	2.06
AIEPGVPSRYSDET	2.18
LHDPLVPIKSYGLT	2.10
TPAPNLPEKTRLAL	3.78
GILPLAPLRPLDPL	3.22
QPLPTSPSKRFSSL	2.22
VKHPMKPFRLMLTD	3.60
SQKPQKPKKSAITK	2.81
QEKPLTPLKYTKLI	2.97
DIHPSLPVRTRFAP	4.39
REEPPLPKRIRISK	4.69
LPVPQLPPKLLVYP	3.64
PPPPPVPAKLFGES	3.47
PACPTPPYRSRETE	2.97
KKYPRKPKKNLNLG	2.17
VENPFLPAKEERTG	1.92
NEVPTTPVRLILGQ	3.40
TKTPTQPIRLQTVS	3.13
DSIPSRPQRRKSVL	4.44
STSPPRPKRIVVAI	3.60
IELPTQPVRKNPEA	2.74
DETPFFPDKIQKSF	2.21
QRHPKSPVKKPMTS	2.83
APTPPAPSRSEKLH	3.67
TDYPFKPPKISFTT	2.57
EAPPAKPSRSDIID	3.53
ECHPELPNKDVKLD	2.14
VIMPEGPRKSVSMA	3.05
RRGPGRPRKDASTS	2.95
YTTPKTPPRPKTIL	4.46
PQGPGIPPRTYEAS	3.42
IIKPYLPFKISKWL	2.94
GAKPKIPSKLYQSV	2.50
TDYPFKPPKVNFTT	2.76
QTSPGQPRKGQNGS	2.25
WNDPSDPTKGFQYL	1.41
IAHPVRPHRAITMT	2.35
SPSPSTPTKSGKMR	2.17
GLEPNTPLKSIKVD	1.68
GNVPHSPVRFADTV	1.69
ASMPRVPLKIVLKD	2.84
PPVPKAPSRRSSSL	3.79
SNSPISPHKLIYKN	2.16
RNEPVKPFRNIDLK	2.92
KLEPENPQKPSNTH	1.98
HLDPEAPDKLLENS	2.52
VSPPFFPEKELNNR	1.99
PNSPTPPLKSKVSR	2.21
VCPPSFPSRRCETC	4.62
TRRPTNPFRVISVS	2.75
YYGPNSPLKLDEEN	1.56
IINPDTPHRRIFNQ	2.72
EYSPLVPSKMCADK	2.25
FKMPKKPQRSKEEV	3.42
TGRPDKPFRALLVD	2.82
PIMPTLPPRPYITI	4.40
RLIPTSPQKTNINT	2.29
ATRPSSPMRHKSIS	3.24
EILPVKPTKKSSKK	1.54
FRFPRLPQKKKEEL	3.14
LRIPETPVKKSPLV	1.57
RPNPTQPRKPLDCK	2.02
YTLPLLPGKGGFRI	1.98
LIPPLHPLKKQHPK	2.56
ILSPGYPNKSFNNK	2.45
VVIPPVPSRYSDER	3.56
ALRPTGPDRGDGFD	2.25
SNSPQIPKKKVKDQ	2.18
SNDPIPPPRKVSDV	3.42
TNPPIDPIREANVM	2.04
EYHPIKPLRSSSST	2.88
ISTPPPPPKMAIPA	2.28
ELPPIKPPRPTSTT	4.63
PIVPREPLRNEPTD	3.71
ITLPRRPAKKIQLG	4.57
QPKPGNPLRNARLN	2.23
DVYPHEPAKNGEGS	2.35
LRKPSAPKRMSSIS	2.43
GKVPVQPRKRKSIT	2.47
ELDPICPLKVPLTA	2.20
ERDPNAPKKPLTVF	2.44
NDDPVIPYKLSKDT	2.37
NSRPQQPERRRFNS	4.16
PPEPATPSKKSLTT	1.82
VQTPHVPDRPPSQL	2.93
GNTPDAPKRGFLGY	2.36
KRPPTAPQRKISLP	3.26
RRKPHSPNKKKPKE	2.05
SNNPSKPRKYKTKS	1.79
RSIPRGPDKNSLRV	2.16
IQHPFLPRKIPIIT	2.40
SGPPLLPPRNTMKN	4.48
NAPPYVPQRSKQPD	3.90
QRLPGEPGREQAPN	2.07
GRIPQLPNRDEMTA	4.32
MRAPPSPRKSKSGH	3.04
LIPPGFPTKNYPPG	2.51
KLGPDTPIKVAGPL	2.02
PNEPGAPSRLSFEN	2.41
YILPKNPFRVPSEM	2.09
QNTPPLPPKPKSPH	4.26
DYLPPVPEKNAGKQ	2.30
VAPPPLPNRQLPNL	4.44
QIFPKKPLRRTVMS	3.93
IFGPSFPFRSHIDV	3.82
FPFPTPPDRVALSK	2.55
DEPPSFPKRWGWLK	3.38
RDKPSEPERTKDGS	2.08
SSTPIVPNRLKTVL	3.29
VTLPTGPAKIIWEK	2.16
LSKPSKPLKEIRFD	2.36
MALPRTPLKTSTEE	2.45
TDLPVNPPRDDKEW	2.08
HPDPADPAKELELN	2.21
NEYPSKPPKVKFPA	1.74
LSTPSAPAKINNYS	2.16
NAKPPLPIKAVTRP	2.46
QLLPNNPDKNNNGT	2.11
EVYPIEPPKVVCLK	1.93
LTCPFEPPKPKTKH	2.17
THAPTTPNRTSPNR	3.61
LENPSTPDKTEEDW	1.84
GTPPTTPLRTAQGI	3.44
EKFPKLPHKLVHML	2.14
SRKPPIPAKQHVKK	1.55
QLTPLLPDKYFDSA	2.32
IFPPLNPARTSLNS	2.92
YGPPTDPRKQKQAK	2.32
QLLPNVPDKVLVPG	1.96
VPEPSKPCRLLLVR	2.63
HGLPYKPSKLIRIF	2.35
TLIPPIPEKQSIKN	2.51
AMNPESPTRDQIFL	2.10
VPPPNVPKKDKREV	2.31
ALKPKTPSKAPRKR	2.53
VAKPKKPARPTMST	3.69
DSPPQSPRRSSNSS	3.53
SKDPNRPTKFAFES	2.37
TTVPTKPKKPHDIS	2.06
MTDPDAPSKTDHKW	2.04
SNFPDFPLKKYLKN	2.30
DSIPVDPEKVVAIV	0.00
YQYPQTPSKVDISI	1.87
SMYPGLPSRLEKEL	3.57
VQQPEDPLRVLGEY	1.82
LHVPRNPSKSRRGS	2.32
YDFPTVPARKFVQP	2.41
FVSPRIPSRIVKNG	3.99
LTSPKNPIKSVTKF	1.61
YTNPRAPGKLQDSH	2.38
NSMPNVPVRLKLHT	4.04
DYQPLSPPRELDPD	2.50
VTLPISPSRASISF	1.90
LRRPIFPGRDYRHQ	3.04
GSSPVSPDKRPSWK	2.69
SQSPKRPKKYPKKA	2.33
QGYPRQPQRPQRYH	3.93
GYDPTDPNRPKVTK	1.84
KLRPDLPVKNFVVK	1.98
KALPKEPKKLLPNS	3.45
ISRPVQPFKVGSIG	2.27
KHAPFIPVKPALEK	1.81
KFIPNKPYRQFNTW	2.62
SMEPHSPKKAKSVE	2.12
QMRPYTPLRSPNSR	3.16
SHLPLIPEKIKSLK	2.13
VSTPPVPKKFQEIF	1.74
ISNPSSPAKTKRDI	2.11
NKIPPKPRKVLQLL	2.24
TGFPPTPLKREGVG	2.63
EKLPETPDRSKCAC	3.75
VPSPGLPEKVNEKS	2.34
DLNPDAPPKPQLIP	1.82
SKFPELPSKVHNAV	2.12
EIAPSLPSRNSIPA	4.61
VHDPNIPLRPKEII	2.04
SSRPTSPVRKARST	2.50
ATVPSAPYRKQIIS	2.86
KGAPFFPNKTFRKL	2.90
DLKPAYPGRSAHVV	3.14
SEEPTSPTRQVNPE	2.48
ETVPTEPTRYNYQN	1.73
FTAPPVPTKKKSKN	2.33
NMVPLNPNRIIPDE	2.70
QETPIIPKRIIHYS	3.89
LPAPHRPEKYKERF	2.23
RYKPTPPLKKRYDY	2.17
ASKPSVPPRNYFSK	3.97
TATPSSPKRMHSSI	2.39
NNNPVPPQKINQPP	1.31
VDFPIFPRRFAKVE	3.23
ESLPRTPTKGRSTQ	2.32
DFTPVEPSKMREYI	2.25
DLPPKFPEKPVHLV	2.26
LPLPTLPDRQLFQT	3.83
MTAPLWPNKNEKNH	2.41
TQAPLAPSRNQENE	2.80
VTKPVVPKKTKKSA	2.13
FFHPAIPLKNAAPF	2.46
IFDPQDPYRRAQQF	2.50
QSPPRSPGRSPTRR	2.61
DYNPPPPSRNSPIR	2.51
KKDPNAPKRALSAY	2.21
DLAPKWPKKTVSNE	2.04
SSYPMNPPKISFMQ	2.33
EKGPRMPSRGRPRP	3.27
TEAPMNPLKNREQM	2.50
QILPTKPVKIIEQN	1.87
EFCPTYPSKLFVPR	2.15
DYNPTIPPRSKDRP	3.38
YLDPFHPKRKFIGV	1.54
TSAPDIPPRSPNRN	4.17
AARPTRPDKARRLG	2.29
KTKPSIPPKKVELN	2.12
EANPDNPKKLDLKA	2.39
CIIPPLPDKKVFQY	2.24
DPLPYSPLKLYDQK	2.33
AGTPNVPTRRPILK	3.64
KSFPPPPLKPGQKT	2.36
RCSPWCPSRSHGAV	3.04
VHGPGIPMKSLEVA	2.58
SSNPDDPIRVTTPM	2.45
VLEPSKPYKKNPFM	2.10
KKDPFPPPKDLLVQ	2.04
LDYPKAPIKLTMKS	2.54
DVLPAGPVRSLEGA	2.34
DSDPNTPKKLSSPR	2.19

CAGPPSRPQRPFCA	3.63
PNIKISRPLKVLLT	1.96
ISMKFLRPLRVLSQ	4.12
VCPGPIRPIKQISD	2.10
TSPAPHRPPKRVKA	0.00
LPIAFIRPFREHHI	3.83
RKMEIYRPHKANAE	1.95
TRTDPTRPIRTPVI	2.75
TRDDPYRPAKPVPT	0.68
GKGRPDRPARRPSP	3.43
DNIPLSRPKRNLSR	3.29
LTLKPIRPRKYPNR	2.68
CRLTLLRPKKDGAA	2.30
VGTELLRPLRGLLS	2.50
SWQILARPWRAFFK	3.01
RELDLSRPSRSISL	3.50
IHCDPLRPRRETCH	3.74
PRRRPGRPRKLPRL	1.51
RVIGLLRPLKDVTV	2.31
HPEFLSRPIKPSPP	1.88
YCTGPPRPGRLFGC	2.50
DKKSPTRPKRHAKP	3.62
LVLEFLRPDRQGVT	2.86
PGEHPGRPGRGGSV	2.62
LLLLLYRPPRYQIA	2.20
YIFSLVRPARYQRV	3.35
RLIAISRPLRYVTV	3.65
RKFSLERPFRPSVT	2.90
SKASLSRPGRRPLG	2.35
GHQEFARPSRGYLA	3.37
FRRLPTRPRKGGPE	2.53
EEAQIFRPLKFNTT	1.92
GPRPPPRPARHRSH	4.12
ETNIPQRPSRPARR	4.32
KPPALPRPIRNLEV	2.07
NGTQILRPPKVLSP	0.70
ENDNIERPVRRRHS	1.95
LVNNFLRPLRMAAS	4.08
PCYHILRPCRNQDL	2.61
GLSTLQRPLKDVTD	2.03
IDPAILRPGRLDKT	2.50
EDALPTRPKKRARS	2.83
AMLSIPRPSKRFWM	2.41
QEALPMRPAREGEL	3.46
ATAFLQRPSRAGSL	4.20
DKKSPTRPKRQAKP	3.58
KAPPPTRPKRNDST	3.93
ACLPPPRPQRPAET	2.32
FKDVLLRPKRSSLK	3.54
PTILPCRPQRSACH	4.24
HSTGINRPKRTGSL	3.46
SRLHFGRPRRADYL	2.93
DGRTPLRPCRNTKQ	4.17
SPDSLSRPRRTVFT	2.68
EDPPPTRPSRPWRD	3.83
PLPRFPRPPRALPA	3.28
RVHRLPRPLKQRML	2.22
GDEPLPRPPRGAPH	2.19
LDPALIRPGRIDRK	3.66
RVPIPPRPTRPHVQ	3.98
HLHILHRPWKCGLC	2.34
RMLVPGRPVKDATP	2.55
EVITLERPLRCSSC	2.82
PTCSPHRPHRLPLA	2.39
WTDRPERPCKPLLT	3.50
XSQHFGRPRRADHL	2.92
TLVWPHRPARPRPV	2.79
RKMEIYRPHKATAE	1.21
YHPPIFRPMKRITW	2.16
PAVPPGRPRRGSLP	3.32
CLLRPWRPLRARGC	3.84
FRRVLLRPSRRVRS	3.76
SWPLLERPWKAFLN	1.09
TPPAFIRPTRKLDD	2.24
SFLTLTRPDRALVR	2.95
GEGGPQRPSRSQRR	4.00
LRPPPMRPVRPVVQ	3.86
RRKAPPRPPRPAAP	3.16
DKLSPDRPIRDTRH	3.55
SQAFIYRPIRQRVY	2.79
DSPSFCRPSKYSPG	1.49
NDRFPFRPSRGRPT	3.41
TLLSPPRPGRLPDL	2.65
ILGFPLRPNRGLYQ	3.93
IDEALLRPGRLEVK	2.57
FDNTLSRPCRVPLG	3.09
LKVWPPRPGRWPCF	3.42
AVPPPSRPQRLSSL	4.19
LHESFPRPKRVCKD	3.54
LWPPLYRPRRDAFE	3.10
PGQGPQRPRRPAKD	3.67
EEPKILRPPRRPRK	4.10
KISWLCRPRRVCLC	3.08
EVQPPVRPGKGGSV	2.16
LPLPFCRPQKIYHF	2.63
LYGLLQRPVRLALG	3.10
TFGIPLRPNRGRGG	3.91
APEALPRPRREGEL	2.85
DVPAFVRPARVXPT	3.39
ARVPLGRPRRRPRA	2.62
EVITLERPLRCNCC	2.58
CCGCPDRPGRRGRR	3.51
TPRKILRPTKETSE	2.24
GEQAPARPPRNVQA	3.54
NNPLIGRPNKVTTV	1.66
RSWWPQRPGRWLSR	3.50
GTLPPPRPRREGRS	3.74
GQLQPSRPTRARGT	3.68
GASCIARPFRRDSQ	2.83
ALPLPGRPARGVFY	2.78
PASAFDRPARTRGQ	3.30
RAPSPARPRRPHAC	3.82
PPTPPPRPQKTHSR	3.95
PPQVPSRPNRAPPG	4.04
HGKGFSRPARPANR	3.51
CCTFLQRPIRKPSK	3.67
QALYFCRPFREKVL	3.03
ITLTPVRPDRTPRP	3.83
GALHPPRPGRGLGQ	2.64
YKPPLSRPLRPGPW	3.08
PRQLLTRPHKAEDF	2.02
SAPPPQRPFRVCDH	3.66
SPRALWRPPRRRTQ	3.00
QYRSLPRPAKSSSM	2.34
RPAVLPRPEKPQLP	2.29
RLNWLGRPSKVLSL	2.33
ALSRLARPQRLPVA	3.13
LNRGPCRPRRQAGR	3.83
DRPIIDRPIRGRGG	2.51
LMIIIARPARLLEC	2.53
EQVDLIRPWRRLLE	2.12
GAASICRPTRAVPS	2.98
LAEELIRPERNTLV	1.29
GLPAPLRPERLAPV	3.38
CLTRIPRPGRQRRD	2.91
VVVALSRPVRPQDL	2.67
ARGSPARPRRLPLL	3.83
LGLELGRPVRFLYE	2.34
KTLRLWRPPRYPRK	3.51
RYWAISRPFRYKRK	3.03
VLAELPRPRRPPLQ	2.78
SDREPTRPPRPILQ	3.59
SRLTLLRPEKDGEA	2.33
VGASLTRPCRKLRW	3.86
ARLPPPRPQRPAET	2.83
GLGELLRPYRCASC	2.56
LFPPLFRPQRPHSA	3.76
CGVRFHRPYRLLMH	3.17
IFDGIQRPLKDINE	1.91
MKRKPGRPRRSPPS	2.14
VLAEIPRPLKRRGS	2.14
ALPRPIRPHRNISA	3.89
RVLCLLRPWRPLRA	3.16
QGSAPLRPFRSGEG	3.85
ILLSISRPYKTKPT	2.24
LYRGLSRPIRRTTQ	2.90
HQEKPERPLRGGKS	4.00
PKDYPLRPPKMKFI	1.91
ETISPLRPQRPKSQ	3.61
PRAPLHRPQKPREG	1.99
NSAGISRPSRVSDA	2.56
VVPQPQRPDKCPLP	1.81
VLPKFPRPVRTQEQ	2.68
LVSIPERPLRPPSE	3.55
VEHVPGRPVRNPQC	2.80
TIPPFERPFKDHQV	0.00
PSGPPARPSRSMWD	3.52
FLNRLVRPRRSAPK	2.86
DGVSLCRPGRTADC	2.30
YSSSLERPRKLRAV	1.89
YLTVLGRPDRPRDL	2.59
ARRPPPRPHRRAET	3.31
EQYIPSRPLKTQEL	1.51
PCPAPPRPARVPAH	3.30
SQIPPQRPQRDQRV	4.15
NSDTPPRPDRLPLD	2.10
RGERLPRPDKCPCE	0.51
YHPPIFRPMKRINL	2.22
CISKPERPMKFLRE	2.78
PGTGPWRPGRQRGA	2.82
TLLGPGRPYRAIDF	2.27
EVDGLSRPQRPRPR	2.13
SGPGPARPARARPR	4.17
ALLTPHRPGRALSF	3.74
GTRAPQRPKRESRG	4.06
GSVHPVRPMRLESF	4.00
HVFQFTRPPRETPT	3.69
KAGSICRPAKDECD	2.22
VATDLGRPARSATG	1.26
TSVPLARPLRTSEE	2.21
IGVSPGRPGKSVLF	2.12
NFGDLGRPGRGGRG	2.95
RLLPPQRPGKCQRV	3.44
KWEPSTPQRKGPIL	3.27
APAPLDPFKTGGNN	2.09
RFNPAIPLRIYLKT	1.82
MSLPVTPVKSKVCS	-0.22
PKEPRLPKRKVAVM	2.36
GVPPNFPQRMMPPP	1.76
QQLPLEPLKPTATG	2.16
SGPPPRPKKPSTLK	2.14
TAAPATPPRHICNN	2.44
SELPKSPYRQKPTQ	3.22
ILYPDIPGKNYFLT	2.07
AIEPGVPSRYSDET	1.80
TPAPNLPEKTRLAL	1.91
GILPLAPLRPLDPL	2.06
QPLPTSPSKRFSSL	1.73
VKHPMKPFRLMLTD	2.01
SQKPQKPKKSAITK	1.94
QEKPLTPLKYTKLI	1.89
LIDPSTPDKAAKKD	1.39
DIHPSLPVRTRFAP	3.83
REEPPLPKRIRISK	3.36
LPVPQLPPKLLVYP	2.57
PPPPPVPAKLFGES	1.91
PACPTPPYRSRETE	1.71
KKYPRKPKKNLNLG	2.26
VENPFLPAKEERTG	0.88
NEVPTTPVRLILGQ	1.88
TKTPTQPIRLQTVS	2.14
QVPPKSPEKSGWLY	1.08
DSIPSRPQRRKSVL	3.18
STSPPRPKRIVVAI	2.55
IELPTQPVRKNPEA	1.62
DETPFFPDKIQKSF	1.58
QRHPKSPVKKPMTS	2.09
APTPPAPSRSEKLH	2.18
TDYPFKPPKISFTT	1.74
EAPPAKPSRSDIID	1.85
ECHPELPNKDVKLD	2.11
VIMPEGPRKSVSMA	1.88
RRGPGRPRKDASTS	2.51
YTTPKTPPRPKTIL	1.63
PQGPGIPPRTYEAS	1.94
IIKPYLPFKISKWL	1.58
GAKPKIPSKLYQSV	1.84
TDYPFKPPKVNFTT	1.77
SHAPTKPTKTYKKW	2.05
KWEPSTPQRNGPIL	2.16
ENFPIVPLKSVAQD	1.97
HTPPLNPSKKCASI	2.03
KMFPFNPAKIKKDD	2.05
FLSPGKPSKGTSLH	1.98
ALRPTPPERGSFPL	2.36
SDLPSSPIKAPNVE	1.62
EEHPSSPGKKQITY	1.92
PLQPRPPVKPLSKQ	1.96
RNEPVIPTKPSSSQ	1.79
PFLPKIPQKPGGVS	1.91
LWIPDDPSKARFLS	1.80
GHTPQFPTKDEVSS	1.40
FISPHLPMKGRLVL	2.36
MQGPKIPWRCGRVD	3.46
RLLPSWPKRGFSLL	1.84
GGDPTKPVRTWNDL	1.49
IPTPSKPLKKRKKR	1.99
SKPPQLPPKCSSLR	1.87
VPSPIVPNKMRKQL	2.72
NIQPNIPLRSYRYL	4.12
SSRPMPPIRGPKRR	3.04
ILLPPLPERAYIMD	1.10
SFVPFPPFKLLTFD	1.88
SENPLLPEKLSQSK	1.74
PRRPNEPGKGMKWR	1.96
NSNPIDPLRCDRSV	2.44
CDFPYDPFKVMRQN	1.62
TEVPSKPKKPSKTE	2.56
IELPHDPHKHFHRD	2.10
EVTPKVPERPSRRK	3.71
LIMPIYPYRLVSTI	1.98
EVIPSWPRRGKVEE	1.71
SSPPPLPTRRDHIK	4.75
PTTPGYPSKDSDTE	1.61
QSNPTSPTKQTFTI	1.79
TSTPPLPRRRATTS	4.21
DYLPQHPHRTSSLP	1.25
VSIPFDPSKDTFDH	2.02
GITPVGPGKFCRSA	1.33
FENPTIPKKSYSEF	1.89
SPPPVSPSKMKTTK	2.02
EKLPAIPRKFIVTE	1.43
SKGPRTPAKEILEF	2.24
AAWPLDPARERWTR	2.74
TLQPGLPHKPLASN	1.96
VPDPSTPSKPNLEV	1.21
CQRPGCPGRYKLVR	2.09
ILKPDIPLRNMLLR	4.17
AGQPEIPLKSNNKS	1.84
IKYPGSPQRSSSFS	2.02
LLKPETPSKRLVIR	2.36
VKKPARPWRAKPGQ	1.96
KYDPEDPNRRKLAR	2.49
QNNPPKPQKPVPLN	1.15
SSIPPNPGKTAKPI	1.85
VERPSSPTKTITTE	1.60
TGLPKKPLKTIRLV	1.76
KELPEFPIKKYHKR	2.07
RPLPQLPNRNNRPV	3.79
PVYPSLPERSKVIL	1.60
NLKPFHPPKEKSMN	2.04
SNSPSPPEKINNKR	1.79
ILTPDFPERRRLMK	3.94
KRTPKIPEKDVKSD	2.18
FPDPNNPSKVQIRM	2.01
VFSPTNPLRIKIAR	2.04
EHKPVSPDKIGTFT	1.77
LYKPGMPPRPHFIT	1.41
QLFPVRPVRVRHED	4.53
ALEPGIPDRFSPEM	2.01
SKIPMLPSRRTMAG	3.42
IHSPRIPSKDEFIA	1.95
KDYPLSPPKLTFTP	1.86
SALPILPKREVVTR	1.86
CLGPLDPLKEISDL	2.01
SRPPRPPPKPLHLR	2.00
SHSPISPLKIETAP	2.29
GAPPPPPHKDHLCD	2.02
LEQPAEPSKLAKKL	1.94
QLEPLAPYKFFDII	1.93
PFLPVLPQKPGGVR	1.70
NVDPASPWRHELLS	1.86
ENVPLYPSRRNIWE	2.88
PRGPLCPKKHVLPI	2.04
HRGPRRPQKNRYNT	0.99
KSSPWSPYKVEKVL	2.07
DDAPATPPRPLKRK	2.38
TDPPPQPRRLSDIV	1.84
FLLPNLPMRTFKEI	2.34
PPPPPPPSRKCGTP	2.75
YWAPVSPLKSINLF	2.02
SQFPSSPYRTVDPY	1.86
KNLPLNPSKKLEGK	2.49
KSKPKVPAKNPELE	2.22
CDFPYDPFRVMRLK	2.52
SSMPNSPKKPVDSL	1.90
EILPQAPAKKVFES	1.54
SQQPTAPMKKTLLK	2.54
YPSPAQPPKSPLLN	1.97
KMTPSTPVRSISPI	2.26
LLPPTDPFRRKHEQ	1.75
LFGPSFPNKTTVEI	1.96
VDAPINPTKILTFI	1.72
LARPPPPKRIRTDS	2.72
QYYPEEPTKPTVKT	1.74
RGPPPLPPRANVQP	3.48
SFAPFFPTKRRGSW	2.53
QPAPRIPSKDAIIS	1.76
IIFPYDPLKLGTHA	1.35
ASYPLTPRRYVPED	3.45
FNGPEPPIKRLKKA	2.41
GSNPDLPNKSNLKF	2.21
SLAPVYPLKSLLRI	3.88
NRKPAEPTKPNNLD	1.71
NYQPKPPIREGNGP	1.64
SDTPASPSKVQSFD	1.97
VVLPKFPDKSFVLD	1.97
KTVPQSPKKPLSLL	2.00
FPYPGNPLKIINYE	2.10
LVTPEEPAKNKSER	2.10
MVTPREPKKRTTRK	2.27
PTAPNSPIKLGRRK	2.13
NNTPIVPEKFSNDL	1.84
ATTPAIPTKSDEAE	1.85
STRPQMPSRTRETA	2.49
FSKPQEPVKAEEAE	1.88
AQRPRGPQRGKDIK	2.08
DDTPSIPSKISSKS	1.80
ENAPANPSKALKVK	1.33
VKRPLSPTRINGKS	2.32
LHYPYGPARNPSYI	2.16
LIQPTAPIRDPTRF	4.48
LHGPFKPVKSSNGE	2.01
ENLPPSPVRAVNPL	1.93
TKVPSKPDRNSKIL	2.18
NSCPMSPLKEKYVL	1.63
PACPTPPYRSRETE	1.49
KKYPRKPKKNLNLG	1.83
VENPFLPAKEERTG	1.20
NEVPTTPVRLILGQ	1.53
TKTPTQPIRLQTVS	1.78
QVPPKSPEKSGWLY	1.36
DSIPSRPQRRKSVL	2.52
STSPPRPKRIVVAI	1.90
IELPTQPVRKNPEA	1.30
DETPFFPDKIQKSF	1.22
QRHPKSPVKKPMTS	1.96
APTPPAPSRSEKLH	1.23
TDYPFKPPKISFTT	1.95
EAPPAKPSRSDIID	1.74
ECHPELPNKDVKLD	1.34
VIMPEGPRKSVSMA	2.36
RRGPGRPRKDASTS	1.73
YTTPKTPPRPKTIL	1.73
PQGPGIPPRTYEAS	1.78
IIKPYLPFKISKWL	1.60
GAKPKIPSKLYQSV	1.88
TDYPFKPPKVNFTT	1.82
SHAPTKPTKTYKKW	1.34
KWEPSTPQRNGPIL	1.50
ENFPIVPLKSVAQD	0.98
HTPPLNPSKKCASI	1.91
KMFPFNPAKIKKDD	2.53
FLSPGKPSKGTSLH	1.54
ALRPTPPERGSFPL	1.44
SDLPSSPIKAPNVE	1.78
EEHPSSPGKKQITY	1.25
PLQPRPPVKPLSKQ	1.54
RNEPVIPTKPSSSQ	1.23
PFLPKIPQKPGGVS	1.66
LWIPDDPSKARFLS	1.40
GHTPQFPTKDEVSS	1.50
FISPHLPMKGRLVL	1.31
MQGPKIPWRCGRVD	2.81
RLLPSWPKRGFSLL	2.40
GGDPTKPVRTWNDL	1.63
IPTPSKPLKKRKKR	1.70
SKPPQLPPKCSSLR	0.82
VPSPIVPNKMRKQL	2.10
NIQPNIPLRSYRYL	2.88
SSRPMPPIRGPKRR	2.57
ILLPPLPERAYIMD	1.59
SFVPFPPFKLLTFD	1.46
SENPLLPEKLSQSK	1.59
PRRPNEPGKGMKWR	1.69
NSNPIDPLRCDRSV	1.66
CDFPYDPFKVMRQN	1.60
TEVPSKPKKPSKTE	1.60
IELPHDPHKHFHRD	1.37
EVTPKVPERPSRRK	2.69
LIMPIYPYRLVSTI	2.75
EVIPSWPRRGKVEE	1.41
SSPPPLPTRRDHIK	2.56
PTTPGYPSKDSDTE	1.43
QSNPTSPTKQTFTI	1.82
TSTPPLPRRRATTS	3.27
DYLPQHPHRTSSLP	1.52
VSIPFDPSKDTFDH	1.53
GITPVGPGKFCRSA	1.75
FENPTIPKKSYSEF	1.74
SPPPVSPSKMKTTK	1.66
EKLPAIPRKFIVTE	1.24
SKGPRTPAKEILEF	1.65
AAWPLDPARERWTR	1.72
TLQPGLPHKPLASN	1.80
VPDPSTPSKPNLEV	1.09
CQRPGCPGRYKLVR	1.71
ILKPDIPLRNMLLR	1.78
AGQPEIPLKSNNKS	1.53
IKYPGSPQRSSSFS	1.58
LLKPETPSKRLVIR	1.12
VKKPARPWRAKPGQ	1.95
KYDPEDPNRRKLAR	1.20
SSIPPNPGKTAKPI	1.63
VERPSSPTKTITTE	1.66
TGLPKKPLKTIRLV	1.87
KELPEFPIKKYHKR	1.34
RPLPQLPNRNNRPV	1.53
PVYPSLPERSKVIL	1.53
NLKPFHPPKEKSMN	1.86
SNSPSPPEKINNKR	1.71
ILTPDFPERRRLMK	2.00
KRTPKIPEKDVKSD	1.68
FPDPNNPSKVQIRM	1.33
VFSPTNPLRIKIAR	1.21
EHKPVSPDKIGTFT	1.33
LYKPGMPPRPHFIT	1.78
QLFPVRPVRVRHED	3.77
ALEPGIPDRFSPEM	1.49
SKIPMLPSRRTMAG	3.67
IHSPRIPSKDEFIA	1.62
KDYPLSPPKLTFTP	1.53
SALPILPKREVVTR	1.52
CLGPLDPLKEISDL	1.63
SRPPRPPPKPLHLR	2.34
SHSPISPLKIETAP	1.67
GAPPPPPHKDHLCD	1.24
LEQPAEPSKLAKKL	1.57
QLEPLAPYKFFDII	1.38
PFLPVLPQKPGGVR	1.55
NVDPASPWRHELLS	0.93
ENVPLYPSRRNIWE	1.28
PRGPLCPKKHVLPI	0.26
HRGPRRPQKNRYNT	1.79
KSSPWSPYKVEKVL	2.09
DDAPATPPRPLKRK	2.00
TDPPPQPRRLSDIV	1.56
FLLPNLPMRTFKEI	1.82
PPPPPPPSRKCGTP	1.70
YWAPVSPLKSINLF	2.36
SQFPSSPYRTVDPY	0.95
KNLPLNPSKKLEGK	1.74
KSKPKVPAKNPELE	1.66
CDFPYDPFRVMRLK	1.53
SSMPNSPKKPVDSL	1.96
EILPQAPAKKVFES	1.33
SQQPTAPMKKTLLK	0.36
YPSPAQPPKSPLLN	1.56
KMTPSTPVRSISPI	1.85
LLPPTDPFRRKHEQ	1.18
LFGPSFPNKTTVEI	1.48
VDAPINPTKILTFI	1.84
LARPPPPKRIRTDS	1.85
QYYPEEPTKPTVKT	1.57
RGPPPLPPRANVQP	1.16
SFAPFFPTKRRGSW	2.54
QPAPRIPSKDAIIS	1.37
IIFPYDPLKLGTHA	1.10
ASYPLTPRRYVPED	4.37
FNGPEPPIKRLKKA	1.06
GSNPDLPNKSNLKF	1.59
SLAPVYPLKSLLRI	1.73
NRKPAEPTKPNNLD	1.98
NYQPKPPIREGNGP	1.73
SDTPASPSKVQSFD	1.89
VVLPKFPDKSFVLD	1.53
KTVPQSPKKPLSLL	1.73
FPYPGNPLKIINYE	1.59
LVTPEEPAKNKSER	1.87
MVTPREPKKRTTRK	1.95
PTAPNSPIKLGRRK	1.83
NNTPIVPEKFSNDL	1.45
ATTPAIPTKSDEAE	1.63
STRPQMPSRTRETA	1.64
FSKPQEPVKAEEAE	1.38
AQRPRGPQRGKDIK	1.86
DDTPSIPSKISSKS	2.06
ENAPANPSKALKVK	1.54
VKRPLSPTRINGKS	1.69
LHYPYGPARNPSYI	1.50
LIQPTAPIRDPTRF	1.84
LHGPFKPVKSSNGE	1.89
ENLPPSPVRAVNPL	1.63
TKVPSKPDRNSKIL	1.44
NSCPMSPLKEKYVL	1.81
PNIKISRPLKVLLT	2.19
ISMKFLRPLRVLSQ	2.97
VCPGPIRPIKQISD	2.54
TSPAPHRPPKRVKA	0.62
LPIAFIRPFREHHI	3.02
RKMEIYRPHKANAE	1.90
TRTDPTRPIRTPVI	1.98
TRDDPYRPAKPVPT	1.83
GKGRPDRPARRPSP	3.94
DNIPLSRPKRNLSR	2.14
LTLKPIRPRKYPNR	2.01
CRLTLLRPKKDGAA	2.20
VGTELLRPLRGLLS	2.05
SWQILARPWRAFFK	1.87
RELDLSRPSRSISL	1.99
IHCDPLRPRRETCH	3.13
PRRRPGRPRKLPRL	1.98
RVIGLLRPLKDVTV	2.42
HPEFLSRPIKPSPP	1.66
YCTGPPRPGRLFGC	2.56
DKKSPTRPKRHAKP	2.82
LVLEFLRPDRQGVT	1.70
PGEHPGRPGRGGSV	1.50
LLLLLYRPPRYQIA	1.95
YIFSLVRPARYQRV	3.25
RLIAISRPLRYVTV	2.82
RKFSLERPFRPSVT	2.02
SKASLSRPGRRPLG	1.73
GHQEFARPSRGYLA	2.33
FRRLPTRPRKGGPE	3.19
EEAQIFRPLKFNTT	1.88
GPRPPPRPARHRSH	4.46
ETNIPQRPSRPARR	3.26
KPPALPRPIRNLEV	2.98
NGTQILRPPKVLSP	2.18
ENDNIERPVRRRHS	1.77
LVNNFLRPLRMAAS	3.10
PCYHILRPCRNQDL	2.63
GLSTLQRPLKDVTD	1.96
IDPAILRPGRLDKT	2.35
EDALPTRPKKRARS	2.46
AMLSIPRPSKRFWM	1.30
QEALPMRPAREGEL	1.85
ATAFLQRPSRAGSL	2.47
DKKSPTRPKRQAKP	2.70
KAPPPTRPKRNDST	3.50
ACLPPPRPQRPAET	1.91
FKDVLLRPKRSSLK	2.75
PTILPCRPQRSACH	3.99
HSTGINRPKRTGSL	2.35
SRLHFGRPRRADYL	2.30
DGRTPLRPCRNTKQ	2.46
SPDSLSRPRRTVFT	1.96
EDPPPTRPSRPWRD	2.58
PLPRFPRPPRALPA	3.54
RVHRLPRPLKQRML	2.08
GDEPLPRPPRGAPH	2.40
LDPALIRPGRIDRK	1.88
RVPIPPRPTRPHVQ	4.29
HLHILHRPWKCGLC	1.45
RMLVPGRPVKDATP	3.47
EVITLERPLRCSSC	2.01
PTCSPHRPHRLPLA	2.22
WTDRPERPCKPLLT	2.71
XSQHFGRPRRADHL	2.26
TLVWPHRPARPRPV	3.35
RKMEIYRPHKATAE	2.25
YHPPIFRPMKRITW	2.47
PAVPPGRPRRGSLP	3.87
CLLRPWRPLRARGC	3.38
FRRVLLRPSRRVRS	2.95
SWPLLERPWKAFLN	2.25
TPPAFIRPTRKLDD	2.16
SFLTLTRPDRALVR	2.32
GEGGPQRPSRSQRR	3.20
LRPPPMRPVRPVVQ	4.21
RRKAPPRPPRPAAP	3.98
DKLSPDRPIRDTRH	2.90
SQAFIYRPIRQRVY	2.01
DSPSFCRPSKYSPG	1.76
NDRFPFRPSRGRPT	3.65
TLLSPPRPGRLPDL	2.28
ILGFPLRPNRGLYQ	3.50
FDNTLSRPCRVPLG	2.23
LKVWPPRPGRWPCF	3.04
AVPPPSRPQRLSSL	3.87
LHESFPRPKRVCKD	2.59
LWPPLYRPRRDAFE	3.59
PGQGPQRPRRPAKD	3.77
EEPKILRPPRRPRK	2.71
KISWLCRPRRVCLC	2.69
EVQPPVRPGKGGSV	2.14
LPLPFCRPQKIYHF	2.83
LYGLLQRPVRLALG	3.04
TFGIPLRPNRGRGG	2.77
APEALPRPRREGEL	2.50
ARVPLGRPRRRPRA	2.30
EVITLERPLRCNCC	2.08
CCGCPDRPGRRGRR	2.18
TPRKILRPTKETSE	1.49
GEQAPARPPRNVQA	2.72
NNPLIGRPNKVTTV	2.43
RSWWPQRPGRWLSR	2.75
GTLPPPRPRREGRS	3.57
GQLQPSRPTRARGT	2.04
GASCIARPFRRDSQ	2.08
ALPLPGRPARGVFY	3.11
PASAFDRPARTRGQ	1.99
RAPSPARPRRPHAC	3.33
PPTPPPRPQKTHSR	4.61
PPQVPSRPNRAPPG	4.29
HGKGFSRPARPANR	0.72
CCTFLQRPIRKPSK	2.53
QALYFCRPFREKVL	2.35
ITLTPVRPDRTPRP	3.79
GALHPPRPGRGLGQ	1.90
YKPPLSRPLRPGPW	2.42
PRQLLTRPHKAEDF	2.30
SAPPPQRPFRVCDH	3.72
SPRALWRPPRRRTQ	3.08
QYRSLPRPAKSSSM	2.88
RPAVLPRPEKPQLP	2.33
RLNWLGRPSKVLSL	2.43
ALSRLARPQRLPVA	2.69
LNRGPCRPRRQAGR	2.32
DRPIIDRPIRGRGG	2.28
LMIIIARPARLLEC	2.22
EQVDLIRPWRRLLE	2.17
GAASICRPTRAVPS	2.31
GLPAPLRPERLAPV	3.28
CLTRIPRPGRQRRD	2.47
ARGSPARPRRLPLL	3.29
LGLELGRPVRFLYE	2.39
KTLRLWRPPRYPRK	2.35
RYWAISRPFRYKRK	2.62
VLAELPRPRRPPLQ	2.39
SDREPTRPPRPILQ	1.72
SRLTLLRPEKDGEA	2.27
VGASLTRPCRKLRW	2.43
ARLPPPRPQRPAET	3.50
GLGELLRPYRCASC	2.05
LFPPLFRPQRPHSA	4.01
CGVRFHRPYRLLMH	2.30
IFDGIQRPLKDINE	2.10
MKRKPGRPRRSPPS	3.00
VLAEIPRPLKRRGS	2.31
ALPRPIRPHRNISA	3.74
RVLCLLRPWRPLRA	2.56
QGSAPLRPFRSGEG	3.27
ILLSISRPYKTKPT	2.12
LYRGLSRPIRRTTQ	2.37
HQEKPERPLRGGKS	3.31
PKDYPLRPPKMKFI	3.27
ETISPLRPQRPKSQ	2.34
PRAPLHRPQKPREG	2.29
NSAGISRPSRVSDA	1.98
VVPQPQRPDKCPLP	2.45
VLPKFPRPVRTQEQ	2.48
LVSIPERPLRPPSE	2.49
VEHVPGRPVRNPQC	1.70
TIPPFERPFKDHQV	2.10
PSGPPARPSRSMWD	3.44
FLNRLVRPRRSAPK	3.30
DGVSLCRPGRTADC	2.05
YSSSLERPRKLRAV	2.14
YLTVLGRPDRPRDL	2.10
ARRPPPRPHRRAET	3.40
EQYIPSRPLKTQEL	2.20
PCPAPPRPARVPAH	3.49
SQIPPQRPQRDQRV	4.18
RGERLPRPDKCPCE	2.07
YHPPIFRPMKRINL	2.40
CISKPERPMKFLRE	3.95
PGTGPWRPGRQRGA	2.19
TLLGPGRPYRAIDF	2.69
EVDGLSRPQRPRPR	2.45
SGPGPARPARARPR	4.24
ALLTPHRPGRALSF	2.69
GTRAPQRPKRESRG	3.15
GSVHPVRPMRLESF	3.39
HVFQFTRPPRETPT	2.23
KAGSICRPAKDECD	2.10
VATDLGRPARSATG	1.95
TSVPLARPLRTSEE	2.27
IGVSPGRPGKSVLF	2.11
NFGDLGRPGRGGRG	2.95
RLLPPQRPGKCQRV	4.40
NCEHFHRPLRRCEA	2.30
KLMEPHRPERQDEK	2.16
EDKALPRPIRPHRN	1.36
VHGDIFRPPRKGML	1.99
PPPPPPRPSKPTPS	4.16
QVIHPPRPPKVLGL	2.63
AGTPNVPTRRPILK	2.65
KSFPPPPLKPGQKT	1.76
RCSPWCPSRSHGAV	2.07
VHGPGIPMKSLEVA	1.64
SSNPDDPIRVTTPM	1.32
VLEPSKPYKKNPFM	0.76
KKDPFPPPKDLLVQ	1.09
LDYPKAPIKLTMKS	0.77
DVLPAGPVRSLEGA	0.00
DSDPNTPKKLSSPR	0.00
LQNPLGPERTFLIM	1.44
AIPPPVPNRPGGTT	2.27
PKDPCSPQRDEILT	1.73
DSGPQRPKRTRAKG	2.34
DVLPAGPVRSLEGV	1.51
VYVPINPPKELDQR	1.23
SLDPDRPEKDCVEE	1.31
TLQPGLPHKPLPSD	1.87
SAFPQSPIRAYGST	1.69
MPTPMTPVKVGALA	1.04
DGRPHSPLRARSAT	1.88
STSPKLPPRGKQRE	4.76
NINPKQPPKTSGEK	0.99
STPPTLPPRRIEDP	4.39
NTVPSTPSRSNSVL	2.59
INQPSTPKKLKRKS	1.80
HEHPCGPLKKLFSD	1.12
SSSPFIPKKRKPRS	1.63
SELPTRPSKSKTYN	1.31
HGVPVVPSREVTIN	1.91
GWKPLRPIKLISWD	2.46
RPTPRKPSRMATDH	3.32
KWEPSTPQRKGPIL	1.71
APAPLDPFKTGGNN	1.68
RFNPAIPLRIYLKT	2.07
MSLPVTPVKSKVCS	2.05
PKEPRLPKRKVAVM	3.74
GVPPNFPQRMMPPP	1.77
QQLPLEPLKPTATG	1.66
SGPPPRPKKPSTLK	1.46
TAAPATPPRHICNN	4.12
SELPKSPYRQKPTQ	1.20
ILYPDIPGKNYFLT	1.51
AIEPGVPSRYSDET	1.56
LHDPLVPIKSYGLT	1.68
TPAPNLPEKTRLAL	1.82
GILPLAPLRPLDPL	1.76
QPLPTSPSKRFSSL	1.51
VKHPMKPFRLMLTD	2.55
SQKPQKPKKSAITK	1.43
QEKPLTPLKYTKLI	2.00
LIDPSTPDKAAKKD	1.45
DIHPSLPVRTRFAP	2.97
REEPPLPKRIRISK	4.07
LPVPQLPPKLLVYP	1.81
PPPPPVPAKLFGES	1.61
PACPTPPYRSRETE	1.12
KKYPRKPKKNLNLG	0.98
VENPFLPAKEERTG	0.00
NEVPTTPVRLILGQ	1.69
TKTPTQPIRLQTVS	2.02
QVPPKSPEKSGWLY	1.37
DSIPSRPQRRKSVL	2.81
STSPPRPKRIVVAI	2.26
IELPTQPVRKNPEA	1.67
DETPFFPDKIQKSF	1.37
QRHPKSPVKKPMTS	2.28
APTPPAPSRSEKLH	1.69
TDYPFKPPKISFTT	1.64
EAPPAKPSRSDIID	1.50
ECHPELPNKDVKLD	1.40
VIMPEGPRKSVSMA	2.25
RRGPGRPRKDASTS	2.33
YTTPKTPPRPKTIL	3.02
PQGPGIPPRTYEAS	2.52
IIKPYLPFKISKWL	1.84
GAKPKIPSKLYQSV	1.63
TDYPFKPPKVNFTT	1.97
SHAPTKPTKTYKKW	1.00
KWEPSTPQRNGPIL	1.49
ENFPIVPLKSVAQD	2.10
HTPPLNPSKKCASI	1.45
KMFPFNPAKIKKDD	2.63
FLSPGKPSKGTSLH	1.77
ALRPTPPERGSFPL	2.65
SDLPSSPIKAPNVE	1.44
EEHPSSPGKKQITY	0.76
PLQPRPPVKPLSKQ	1.76
RNEPVIPTKPSSSQ	0.69
PFLPKIPQKPGGVS	1.70
LWIPDDPSKARFLS	1.56
GHTPQFPTKDEVSS	1.34
FISPHLPMKGRLVL	1.83
MQGPKIPWRCGRVD	3.10
RLLPSWPKRGFSLL	2.86
GGDPTKPVRTWNDL	1.21
IPTPSKPLKKRKKR	1.93
SKPPQLPPKCSSLR	2.15
VPSPIVPNKMRKQL	2.31
NIQPNIPLRSYRYL	2.96
SSRPMPPIRGPKRR	2.89
ILLPPLPERAYIMD	4.17
SFVPFPPFKLLTFD	1.83
SENPLLPEKLSQSK	1.40
PRRPNEPGKGMKWR	1.27
NSNPIDPLRCDRSV	1.36
CDFPYDPFKVMRQN	1.07
TEVPSKPKKPSKTE	1.88
IELPHDPHKHFHRD	1.31
EVTPKVPERPSRRK	3.32
LIMPIYPYRLVSTI	2.28
EVIPSWPRRGKVEE	1.54
SSPPPLPTRRDHIK	4.53
PTTPGYPSKDSDTE	1.66
QSNPTSPTKQTFTI	1.78
TSTPPLPRRRATTS	4.75
DYLPQHPHRTSSLP	2.04
VSIPFDPSKDTFDH	1.86
GITPVGPGKFCRSA	1.51
FENPTIPKKSYSEF	1.67
SPPPVSPSKMKTTK	1.40
EKLPAIPRKFIVTE	1.62
SKGPRTPAKEILEF	1.73
AAWPLDPARERWTR	1.41
TLQPGLPHKPLASN	1.21
VPDPSTPSKPNLEV	1.29
CQRPGCPGRYKLVR	1.97
ILKPDIPLRNMLLR	3.21
AGQPEIPLKSNNKS	0.00
IKYPGSPQRSSSFS	1.36
LLKPETPSKRLVIR	1.41
VKKPARPWRAKPGQ	1.70
KYDPEDPNRRKLAR	1.49
QNNPPKPQKPVPLN	1.37
SSIPPNPGKTAKPI	1.75
VERPSSPTKTITTE	1.39
TGLPKKPLKTIRLV	1.89
KELPEFPIKKYHKR	1.48
RPLPQLPNRNNRPV	4.40
PVYPSLPERSKVIL	3.60
NLKPFHPPKEKSMN	2.11
SNSPSPPEKINNKR	1.67
ILTPDFPERRRLMK	2.39
KRTPKIPEKDVKSD	2.38
FPDPNNPSKVQIRM	1.45
VFSPTNPLRIKIAR	0.26
EHKPVSPDKIGTFT	1.77
LYKPGMPPRPHFIT	1.54
QLFPVRPVRVRHED	4.06
ALEPGIPDRFSPEM	1.75
SKIPMLPSRRTMAG	4.77
IHSPRIPSKDEFIA	1.86
KDYPLSPPKLTFTP	1.41
SALPILPKREVVTR	4.30
CLGPLDPLKEISDL	0.92
SRPPRPPPKPLHLR	2.75
SHSPISPLKIETAP	1.31
GAPPPPPHKDHLCD	1.88
LEQPAEPSKLAKKL	0.92
QLEPLAPYKFFDII	1.80
PFLPVLPQKPGGVR	1.30
NVDPASPWRHELLS	1.52
ENVPLYPSRRNIWE	1.95
PRGPLCPKKHVLPI	1.47
HRGPRRPQKNRYNT	1.78
KSSPWSPYKVEKVL	2.19
DDAPATPPRPLKRK	3.65
TDPPPQPRRLSDIV	2.09
FLLPNLPMRTFKEI	3.91
PPPPPPPSRKCGTP	3.18
YWAPVSPLKSINLF	1.40
SQFPSSPYRTVDPY	1.57
KNLPLNPSKKLEGK	1.83
KSKPKVPAKNPELE	1.71
CDFPYDPFRVMRLK	1.62
SSMPNSPKKPVDSL	2.52
EILPQAPAKKVFES	1.81
SQQPTAPMKKTLLK	1.73
YPSPAQPPKSPLLN	1.26
KMTPSTPVRSISPI	2.45
LLPPTDPFRRKHEQ	1.69
LFGPSFPNKTTVEI	1.30
VDAPINPTKILTFI	1.83
LARPPPPKRIRTDS	3.10
QYYPEEPTKPTVKT	0.81
RGPPPLPPRANVQP	4.26
SFAPFFPTKRRGSW	2.37
QPAPRIPSKDAIIS	2.23
IIFPYDPLKLGTHA	1.92
ASYPLTPRRYVPED	3.14
FNGPEPPIKRLKKA	1.85
GSNPDLPNKSNLKF	1.42
SLAPVYPLKSLLRI	1.66
SHAPTKPTKTYKKW	3.56
KWEPSTPQRNGPIL	3.53
ENFPIVPLKSVAQD	2.58
HTPPLNPSKKCASI	1.18
KMFPFNPAKIKKDD	3.40
FLSPGKPSKGTSLH	2.64
ALRPTPPERGSFPL	3.29
SDLPSSPIKAPNVE	0.92
EEHPSSPGKKQITY	1.65
PLQPRPPVKPLSKQ	2.93
RNEPVIPTKPSSSQ	2.71
PFLPKIPQKPGGVS	3.34
LWIPDDPSKARFLS	2.75
GHTPQFPTKDEVSS	1.30
FISPHLPMKGRLVL	3.19
RLLPSWPKRGFSLL	4.03
GGDPTKPVRTWNDL	2.31
IPTPSKPLKKRKKR	3.20
SKPPQLPPKCSSLR	4.65
VPSPIVPNKMRKQL	3.42
NIQPNIPLRSYRYL	4.29
SSRPMPPIRGPKRR	4.06
ILLPPLPERAYIMD	4.42
SFVPFPPFKLLTFD	2.51
PRRPNEPGKGMKWR	2.34
NSNPIDPLRCDRSV	2.48
CDFPYDPFKVMRQN	1.52
TEVPSKPKKPSKTE	3.52
EVTPKVPERPSRRK	4.77
LIMPIYPYRLVSTI	3.49
EVIPSWPRRGKVEE	2.97
SSPPPLPTRRDHIK	4.55
PTTPGYPSKDSDTE	2.14
QSNPTSPTKQTFTI	2.17
TSTPPLPRRRATTS	4.56
DYLPQHPHRTSSLP	2.31
GITPVGPGKFCRSA	1.33
FENPTIPKKSYSEF	1.70
SPPPVSPSKMKTTK	1.36
EKLPAIPRKFIVTE	2.82
SKGPRTPAKEILEF	2.58
AAWPLDPARERWTR	2.48
TLQPGLPHKPLASN	0.59
VPDPSTPSKPNLEV	2.13
CQRPGCPGRYKLVR	2.80
ILKPDIPLRNMLLR	4.11
AGQPEIPLKSNNKS	3.10
IKYPGSPQRSSSFS	2.59
LLKPETPSKRLVIR	3.17
VKKPARPWRAKPGQ	3.73
KYDPEDPNRRKLAR	2.35
QNNPPKPQKPVPLN	2.06
SSIPPNPGKTAKPI	2.13
TGLPKKPLKTIRLV	2.87
KELPEFPIKKYHKR	3.03
RPLPQLPNRNNRPV	4.29
PVYPSLPERSKVIL	4.38
NLKPFHPPKEKSMN	2.62
SNSPSPPEKINNKR	2.07
ILTPDFPERRRLMK	4.14
KRTPKIPEKDVKSD	2.53
FPDPNNPSKVQIRM	1.99
VFSPTNPLRIKIAR	2.81
EHKPVSPDKIGTFT	1.78
LYKPGMPPRPHFIT	2.95
QLFPVRPVRVRHED	4.46
ALEPGIPDRFSPEM	1.99
SKIPMLPSRRTMAG	4.65
IHSPRIPSKDEFIA	2.35
KDYPLSPPKLTFTP	1.20
CLGPLDPLKEISDL	1.13
SRPPRPPPKPLHLR	4.41
SHSPISPLKIETAP	1.59
GAPPPPPHKDHLCD	1.67
LEQPAEPSKLAKKL	1.29
QLEPLAPYKFFDII	1.85
PFLPVLPQKPGGVR	4.11
NVDPASPWRHELLS	1.80
PRGPLCPKKHVLPI	2.46
HRGPRRPQKNRYNT	3.23
KSSPWSPYKVEKVL	2.40
DDAPATPPRPLKRK	4.42
TDPPPQPRRLSDIV	3.74
FLLPNLPMRTFKEI	4.19
PPPPPPPSRKCGTP	3.84
YWAPVSPLKSINLF	3.20
SQFPSSPYRTVDPY	2.06
KNLPLNPSKKLEGK	1.99
KSKPKVPAKNPELE	2.60
CDFPYDPFRVMRLK	2.36
SSMPNSPKKPVDSL	2.96
EILPQAPAKKVFES	2.39
SQQPTAPMKKTLLK	1.77
YPSPAQPPKSPLLN	2.72
KMTPSTPVRSISPI	4.13
LFGPSFPNKTTVEI	1.95
LARPPPPKRIRTDS	4.19
QYYPEEPTKPTVKT	1.70
RGPPPLPPRANVQP	4.41
SFAPFFPTKRRGSW	4.19
QPAPRIPSKDAIIS	3.21
IIFPYDPLKLGTHA	1.24
ASYPLTPRRYVPED	3.84
FNGPEPPIKRLKKA	2.80
GSNPDLPNKSNLKF	2.80
SLAPVYPLKSLLRI	2.89
NRKPAEPTKPNNLD	2.47
NYQPKPPIREGNGP	2.06
SDTPASPSKVQSFD	1.97
VVLPKFPDKSFVLD	2.73
KTVPQSPKKPLSLL	2.45
FPYPGNPLKIINYE	1.69
LVTPEEPAKNKSER	1.69
MVTPREPKKRTTRK	2.79
PTAPNSPIKLGRRK	2.74
NNTPIVPEKFSNDL	2.29
ATTPAIPTKSDEAE	2.22
STRPQMPSRTRETA	3.61
FSKPQEPVKAEEAE	2.44
AQRPRGPQRGKDIK	2.76
DDTPSIPSKISSKS	2.09
ENAPANPSKALKVK	1.54
VKRPLSPTRINGKS	3.36
LHYPYGPARNPSYI	2.44
LIQPTAPIRDPTRF	2.73
LHGPFKPVKSSNGE	2.46
ENLPPSPVRAVNPL	2.19
TKVPSKPDRNSKIL	3.64
NSCPMSPLKEKYVL	2.45
LQNPLGPERTFLIM	1.69
AIPPPVPNRPGGTT	3.78
DSGPQRPKRTRAKG	3.62
VYVPINPPKELDQR	2.12
SLDPDRPEKDCVEE	1.97
TLQPGLPHKPLPSD	2.55
SAFPQSPIRAYGST	2.19
MPTPMTPVKVGALA	1.82
DGRPHSPLRARSAT	2.29
STSPKLPPRGKQRE	4.86
NINPKQPPKTSGEK	2.35
STPPTLPPRRIEDP	4.69
NTVPSTPSRSNSVL	3.97
INQPSTPKKLKRKS	2.10
SSSPFIPKKRKPRS	2.41
SELPTRPSKSKTYN	2.57
HGVPVVPSREVTIN	2.88
GWKPLRPIKLISWD	2.61
RPTPRKPSRMATDH	3.42
KWEPSTPQRKGPIL	2.78
APAPLDPFKTGGNN	1.36
RFNPAIPLRIYLKT	2.93
MSLPVTPVKSKVCS	2.41
PKEPRLPKRKVAVM	4.59
GVPPNFPQRMMPPP	2.45
QQLPLEPLKPTATG	2.08
SGPPPRPKKPSTLK	2.48
TAAPATPPRHICNN	4.77
ILYPDIPGKNYFLT	2.06
AIEPGVPSRYSDET	2.18
LHDPLVPIKSYGLT	2.36
TPAPNLPEKTRLAL	2.34
GILPLAPLRPLDPL	2.50
QPLPTSPSKRFSSL	2.40
VKHPMKPFRLMLTD	3.49
SQKPQKPKKSAITK	1.91
QEKPLTPLKYTKLI	0.85
LIDPSTPDKAAKKD	2.48
DIHPSLPVRTRFAP	3.71
REEPPLPKRIRISK	4.70
LPVPQLPPKLLVYP	2.29
PPPPPVPAKLFGES	1.80
PACPTPPYRSRETE	2.86
KKYPRKPKKNLNLG	2.61
VENPFLPAKEERTG	2.27
NEVPTTPVRLILGQ	3.32
TKTPTQPIRLQTVS	2.37
QVPPKSPEKSGWLY	2.07
DSIPSRPQRRKSVL	3.63
STSPPRPKRIVVAI	3.13
IELPTQPVRKNPEA	2.05
QRHPKSPVKKPMTS	2.23
APTPPAPSRSEKLH	2.65
TDYPFKPPKISFTT	2.06
EAPPAKPSRSDIID	3.30
ECHPELPNKDVKLD	1.88
VIMPEGPRKSVSMA	2.55
RRGPGRPRKDASTS	1.76
YTTPKTPPRPKTIL	4.44
PQGPGIPPRTYEAS	3.23
IIKPYLPFKISKWL	2.23
TDYPFKPPKVNFTT	2.37
SHAPTKPTKTYKKW	2.01
KWEPSTPQRNGPIL	3.21
ENFPIVPLKSVAQD	2.00
HTPPLNPSKKCASI	1.96
KMFPFNPAKIKKDD	2.61
FLSPGKPSKGTSLH	2.39
ALRPTPPERGSFPL	2.96
SDLPSSPIKAPNVE	1.71
EEHPSSPGKKQITY	1.39
PLQPRPPVKPLSKQ	2.11
RNEPVIPTKPSSSQ	2.06
PFLPKIPQKPGGVS	2.24
LWIPDDPSKARFLS	2.30
GHTPQFPTKDEVSS	2.42
FISPHLPMKGRLVL	2.48
MQGPKIPWRCGRVD	4.14
RLLPSWPKRGFSLL	3.05
GGDPTKPVRTWNDL	2.23
IPTPSKPLKKRKKR	2.33
SKPPQLPPKCSSLR	3.46
VPSPIVPNKMRKQL	2.28
NIQPNIPLRSYRYL	3.77
SSRPMPPIRGPKRR	3.28
ILLPPLPERAYIMD	4.70
SFVPFPPFKLLTFD	2.46
SENPLLPEKLSQSK	2.20
PRRPNEPGKGMKWR	2.26
NSNPIDPLRCDRSV	1.83
CDFPYDPFKVMRQN	2.08
TEVPSKPKKPSKTE	1.83
IELPHDPHKHFHRD	1.89
EVTPKVPERPSRRK	4.27
LIMPIYPYRLVSTI	2.99
EVIPSWPRRGKVEE	2.14
SSPPPLPTRRDHIK	5.07
PTTPGYPSKDSDTE	1.40
QSNPTSPTKQTFTI	2.26
TSTPPLPRRRATTS	5.09
DYLPQHPHRTSSLP	2.24
VSIPFDPSKDTFDH	1.85
GITPVGPGKFCRSA	1.91
FENPTIPKKSYSEF	1.64
SPPPVSPSKMKTTK	2.39
EKLPAIPRKFIVTE	2.32
AAWPLDPARERWTR	2.13
TLQPGLPHKPLASN	1.94
VPDPSTPSKPNLEV	2.19
CQRPGCPGRYKLVR	2.31
ILKPDIPLRNMLLR	3.38
AGQPEIPLKSNNKS	2.01
IKYPGSPQRSSSFS	2.30
LLKPETPSKRLVIR	1.78
VKKPARPWRAKPGQ	2.50
KYDPEDPNRRKLAR	2.24
QNNPPKPQKPVPLN	2.17
SSIPPNPGKTAKPI	2.63
VERPSSPTKTITTE	2.20
TGLPKKPLKTIRLV	2.16
KELPEFPIKKYHKR	0.79
RPLPQLPNRNNRPV	3.96
PVYPSLPERSKVIL	4.32
NLKPFHPPKEKSMN	2.89
SNSPSPPEKINNKR	2.14
ILTPDFPERRRLMK	3.25
KRTPKIPEKDVKSD	2.27
FPDPNNPSKVQIRM	2.08
VFSPTNPLRIKIAR	2.18
EHKPVSPDKIGTFT	2.26
LYKPGMPPRPHFIT	2.51
QLFPVRPVRVRHED	4.14
ALEPGIPDRFSPEM	1.48
SKIPMLPSRRTMAG	5.01
IHSPRIPSKDEFIA	2.27
KDYPLSPPKLTFTP	1.03
SALPILPKREVVTR	4.64
CLGPLDPLKEISDL	2.31
SRPPRPPPKPLHLR	3.01
SHSPISPLKIETAP	1.66
GAPPPPPHKDHLCD	2.19
LEQPAEPSKLAKKL	1.98
QLEPLAPYKFFDII	2.52
PFLPVLPQKPGGVR	2.53
NVDPASPWRHELLS	2.43
ENVPLYPSRRNIWE	3.65
PRGPLCPKKHVLPI	2.31
HRGPRRPQKNRYNT	2.21
KSSPWSPYKVEKVL	2.51
DDAPATPPRPLKRK	4.13
TDPPPQPRRLSDIV	3.25
FLLPNLPMRTFKEI	3.91
PPPPPPPSRKCGTP	3.39
YWAPVSPLKSINLF	2.23
SQFPSSPYRTVDPY	2.52
KNLPLNPSKKLEGK	1.79
KSKPKVPAKNPELE	2.32
CDFPYDPFRVMRLK	2.39
SSMPNSPKKPVDSL	2.03
EILPQAPAKKVFES	1.10
YPSPAQPPKSPLLN	1.35
KMTPSTPVRSISPI	3.47
LLPPTDPFRRKHEQ	2.35
LFGPSFPNKTTVEI	2.24
VDAPINPTKILTFI	1.53
LARPPPPKRIRTDS	3.51
QYYPEEPTKPTVKT	1.93
RGPPPLPPRANVQP	4.52
SFAPFFPTKRRGSW	3.00
QPAPRIPSKDAIIS	1.98
IIFPYDPLKLGTHA	1.75
ASYPLTPRRYVPED	3.61
FNGPEPPIKRLKKA	1.63
GSNPDLPNKSNLKF	2.25
SLAPVYPLKSLLRI	2.24
NRKPAEPTKPNNLD	2.37
NYQPKPPIREGNGP	2.19
SDTPASPSKVQSFD	1.67
VVLPKFPDKSFVLD	2.22
KTVPQSPKKPLSLL	2.28
LVTPEEPAKNKSER	1.84
MVTPREPKKRTTRK	2.89
PTAPNSPIKLGRRK	2.24
NNTPIVPEKFSNDL	2.43
STRPQMPSRTRETA	1.57
FSKPQEPVKAEEAE	2.89
AQRPRGPQRGKDIK	2.05
DDTPSIPSKISSKS	2.27
ENAPANPSKALKVK	2.32
VKRPLSPTRINGKS	2.70
LHYPYGPARNPSYI	2.15
LIQPTAPIRDPTRF	2.46
LHGPFKPVKSSNGE	2.18

NCEHFHRPLRRCEA	2.71
KLMEPHRPERQDEK	2.69
EDKALPRPIRPHRN	2.68
VHGDIFRPPRKGML	3.60
PPPPPPRPSKPTPS	3.11
QVIHPPRPPKVLGL	2.07
PASALPRPPRLSGS	3.65
GDPAPCRPLRAHFL	3.96
LDPALLRPGRFDRQ	2.91
PCKEILRPRRTLQK	3.02
SLCFIARPGKVCQL	2.18
NPALFPRPERYNPQ	2.50
AGPRLPRPPREGST	3.35
QDAYLSRPGRALAH	2.22
IYTELCRPSKRRMQ	1.93
TRRVLLRPEKRPPV	2.25
QLFTPNRPKKREIY	2.22
EWACLLRPLRGREP	2.49
SCRAPPRPAKTTDP	2.58
LDGALMRPGRLDRH	3.19
ACLPPPRPQRPVTK	4.00
VLLPPPRPTRKRSL	3.92
TLLALQRPQRLHHH	3.51
PWRGFCRPSRFFSS	3.48
DDILINRPAKKHLE	0.88
SQGPPPRPGKPEGS	2.01
GINAFARPCKMPYV	2.02
MLLRLARPAKLSEL	2.54
LEYVLFRPNRRYNQ	2.65
PERLLLRPARIRAD	3.01
PPLLPGRPARDRGP	3.79
SINLLSRPERGWEG	0.00
CRLTLLRPEKDGTA	2.38
DRWDPTRPPRATDT	2.56
ILPCLSRPARCDQA	3.12
PGSGPPRPSRRLLD	2.87
PSESLPRPRRPLPT	2.42
PSMDFNRPGKRAPW	1.89
GMEKIWRPNRVFSL	3.05
SSRDLCRPPKKGEL	2.08
WIPVLQRPSRTAAV	3.38
KNLDISRPDKKSKK	2.51
PEPELCRPIRHHPD	1.99
HAQSLSRPLKQTPI	1.77
LDPALMRPGRFHRQ	3.46
GWRSLQRPARRSGR	3.41
EADSIPRPYRCQQC	1.02
GAFQPGRPLRVVAT	3.10
SEGSLTRPKKAIPP	0.00
REAALERPRRTKRE	3.40
TYTAPPRPPKKREY	2.28
LHVQLNRPNKRNAM	2.28
KTEKPERPSRERDR	3.96
PRGVLPRPCRVLVL	3.27
CCELIERPSKKDGF	2.08
PLMHPARPFRVSNH	3.90
CRLTLLRPEKDGAA	2.47
LPVCLTRPPKLVLH	2.08
QGIEFLRPLKTTTP	1.50
EAQFFLRPLRCYGD	3.50
SSRRPVRPGRRGLG	3.86
GACLFNRPTKLFEP	1.77
PPTRPSRPWRDLPE	3.63
YRHGISRPLRPLCS	3.18
PPPPIPRPAKLQAA	1.96
LNKYLSRPKRFEVA	1.86
LLSQLGRPFREYEL	2.31
EEIALERPEKEIRN	2.09
AHMAISRPLKASQM	2.14
LRWGFSRPRKDKGL	2.41
GKNRPLRPSRRRQL	3.87
GHRPPGRPRRCPKA	3.01
GTRQLGRPKRCPRS	2.17
TRKELERPIKVPVV	0.00
WGCLPARPRRPTPT	3.67
TPPDPPRPKRRSHS	3.37
ESSGPPRPSRGPAA	3.43
LTHVLCRPRRGCCG	3.47
HCDYLNRPHKSIHR	2.06
ELYPICRPLKKTWE	0.00
DGEMFMRPGKLFDY	2.07
IPREPGRPGKGNYW	1.77
LIRTFQRPDRVELM	3.79
TPNPLERPIKMGWL	1.97
AALRPPRPARGSRL	4.23
RPPFLSRPARGLYS	2.89
LDPALMRPGRLDRK	3.67
SLLDFIRPLKISSD	2.34
IDPAILRPGRPDQL	2.34
FNKYISRPRRVELA	2.38
KFLDPARPYKCTVC	1.31
RIIAFSRPVKYEDV	2.05
PPLEPARPNRHPES	2.45
KDPGPPRPHRPEAT	3.06
SQGPLQRPSRLVFT	3.14
CSKEFNRPDKLKAH	2.12
LSADPERPGRFRLE	1.57
KLMKFSRPKKTRSG	0.00
KSQYFWRPKRMELS	2.87
GREAPGRPPRAVPK	3.33
KTKTLLRPERSRFD	2.38
NIQTLDRPIKKGPV	1.21
PPYGPSRPDKKQRM	2.29
LLEPPERPYRDREP	2.27
ALKQLDRPEKGLSE	1.63
VCLALVRPCKMGLC	1.73
TPSELERPCRMVSR	2.89
GDSKLFRPRRPRSS	2.99
DGKFLLRPRKEQGT	2.63
HTKNLLRPTKLNLQ	2.04
ACEIIERPSKKDGF	2.11
WICQICRPRKKGRK	2.28
ALRGLVRPHKKRKK	1.74
HALHPARPARDHLD	3.66
VRLPFHRPPRAGAW	3.45
VGASLTRPNRRLRW	3.29
MLQAPIRPPKPQSS	3.02
VCPGPIRPIRQISD	3.11
CEGSLTRPKKAIPA	1.88
AALLFLRPPRVRGH	3.90
HYLVPVRPRRQIVY	3.71
QAVHLPRPPRVLGL	3.28
GGHGPERPKRPGSL	3.89
AQPQPLRPPRRVSS	4.40
MTTTPERPRRGIPG	3.98
PHADPGRPERLVFK	2.23
PGNIPQRPDRLPMS	3.33
QHYTLGRPGRAPRR	2.75
PRKYPNRPSKTPLH	1.90
EQKRPLRPRRKGRK	4.07
CELRLSRPPKAYGS	1.56
ASPSLERPEKGAGK	1.18
DEAQLSRPKKSCDR	1.71
RKPELERPIKVNLA	2.01
EKPILDRPTRISQP	1.76
RRQPPVRPGRVHLP	3.62
IGRNIARPWKLIYY	2.31
PHSAPLRPPRELHA	3.97
ELTIIERPVRRSLK	2.85
LKAPPARPVKGAYR	2.38
LTCGPARPWKSVAL	2.15
PPQVPSRPTRAPPR	3.80
CGGRPARPDRGPEP	3.24
SFSELVRPFKSNKS	1.52
PAGELCRPKKDMCD	2.02
NSHLLPRPLRHLAC	3.21
TSPAPHRPPKRGPL	2.34
GSECLSRPWRARAK	2.39
ARRLLVRPARLRRH	3.13
DGSFLARPSKSNPG	1.98
HLLLFHRPLRASGA	3.23
SPGFPARPGRGRPY	2.75
VCRDITRPPKTVAT	2.08
AKTPLSRPVKRNNA	1.72
QNRPLIRPKKRLIV	1.64
ICHLPCRPVKPNII	1.99
MDGFLCRPCRPLLQ	2.47
VQSIINRPRKSPLP	2.04
KDVLFIRPRKYIHC	1.82
TRESIVRPSRRGGI	3.12
RHQRLHRPGRPVPG	1.07
YRRRFCRPSRARDI	2.67
ASATLGRPPRIPEP	2.14
EVPDPNRPQRLGLH	2.30
WAGTLQRPVKLLPW	2.04
LGQKPQRPRRPASP	4.23
ARRPLARPIKRGDI	2.36
AAVAPSRPGREPQA	3.40
GWGSPGRPKKKHTG	2.13
SGERLPRPDKGKMR	1.96
LNCSFSRPDRPASV	3.61
IDPALLRPGRLDKC	2.79
LMIHPPRPPKVLGL	2.65
RTEGFWRPLRGVNV	2.98
SSVRPPRPKKEPQT	3.37
VKRRPGRPRKCPLQ	2.22
WSLKLCRPSKDYLL	3.05
SVIKPPRPDRGWFV	3.18
DNLKLFRPKKLLPK	1.62
WSEVLHRPWRNHPI	2.43
RAPRPTRPDKARRL	2.34
ATCHFYRPPRCSHC	3.92
PVVLPWRPARAHSC	3.93
LWRRFGRPAKCSTP	2.02
DPSGLPRPQKIPIS	2.17
SQGPPPRPGKPEGQ	2.08
MRKYPNRPSKTPPH	2.35
LLEQILRPCKFLTY	1.48
LVNGFLRPLRMAAS	3.69
GHQEFARPSRGDLA	3.20
LPEELPRPRRPPPE	2.01
ASFQPARPHRARHP	3.50
RVEELSRPKRFYLE	2.35
PASALPRPPRLSGS	1.97
GDPAPCRPLRAHFL	3.70
LDPALLRPGRFDRQ	2.14
PCKEILRPRRTLQK	2.26
SLCFIARPGKVCQL	2.17
NPALFPRPERYNPQ	2.01
AGPRLPRPPREGST	3.05
QDAYLSRPGRALAH	2.34
IYTELCRPSKRRMQ	1.78
TRRVLLRPEKRPPV	2.09
QLFTPNRPKKREIY	2.84
EWACLLRPLRGREP	2.23
SCRAPPRPAKTTDP	2.45
LDGALMRPGRLDRH	3.20
ACLPPPRPQRPVTK	3.77
VLLPPPRPTRKRSL	3.87
TLLALQRPQRLHHH	2.94
PWRGFCRPSRFFSS	2.34
DDILINRPAKKHLE	2.10
SQGPPPRPGKPEGS	2.12
GINAFARPCKMPYV	1.99
MLLRLARPAKLSEL	2.53
LEYVLFRPNRRYNQ	1.74
PERLLLRPARIRAD	2.03
PPLLPGRPARDRGP	4.43
SINLLSRPERGWEG	1.99
CRLTLLRPEKDGTA	2.05
DRWDPTRPPRATDT	1.79
ILPCLSRPARCDQA	2.56
PGSGPPRPSRRLLD	3.36
PSESLPRPRRPLPT	2.61
PSMDFNRPGKRAPW	2.66
GMEKIWRPNRVFSL	2.24
SSRDLCRPPKKGEL	2.04
WIPVLQRPSRTAAV	3.12
KNLDISRPDKKSKK	2.53
PEPELCRPIRHHPD	2.07
HAQSLSRPLKQTPI	2.19
LDPALMRPGRFHRQ	2.49
GWRSLQRPARRSGR	1.59
GAFQPGRPLRVVAT	2.17
SEGSLTRPKKAIPP	1.84
REAALERPRRTKRE	2.52
TYTAPPRPPKKREY	2.22
LHVQLNRPNKRNAM	2.11
KTEKPERPSRERDR	2.60
PRGVLPRPCRVLVL	2.71
CCELIERPSKKDGF	1.85
PLMHPARPFRVSNH	4.20
CRLTLLRPEKDGAA	1.85
LPVCLTRPPKLVLH	2.43
QGIEFLRPLKTTTP	1.92
EAQFFLRPLRCYGD	2.02
SSRRPVRPGRRGLG	3.27
GACLFNRPTKLFEP	1.82
PPTRPSRPWRDLPE	4.16
YRHGISRPLRPLCS	2.94
PPPPIPRPAKLQAA	2.01
LNKYLSRPKRFEVA	2.33
AHMAISRPLKASQM	2.62
LRWGFSRPRKDKGL	2.01
GKNRPLRPSRRRQL	3.27
GHRPPGRPRRCPKA	2.24
GTRQLGRPKRCPRS	2.18
TRKELERPIKVPVV	2.51
WGCLPARPRRPTPT	3.86
TPPDPPRPKRRSHS	2.31
ESSGPPRPSRGPAA	2.47
LTHVLCRPRRGCCG	2.23
HCDYLNRPHKSIHR	1.95
DGEMFMRPGKLFDY	1.86
IPREPGRPGKGNYW	1.52
LIRTFQRPDRVELM	2.35
TPNPLERPIKMGWL	1.77
AALRPPRPARGSRL	4.38
RPPFLSRPARGLYS	3.16
LDPALMRPGRLDRK	2.54
IDPAILRPGRPDQL	2.23
FNKYISRPRRVELA	2.59
KFLDPARPYKCTVC	1.64
RIIAFSRPVKYEDV	1.90
KDPGPPRPHRPEAT	2.61
SQGPLQRPSRLVFT	1.84
CSKEFNRPDKLKAH	2.08
LSADPERPGRFRLE	2.08
KLMKFSRPKKTRSG	0.23
KSQYFWRPKRMELS	2.37
GREAPGRPPRAVPK	4.29
KTKTLLRPERSRFD	1.91
NIQTLDRPIKKGPV	1.77
PPYGPSRPDKKQRM	2.60
LLEPPERPYRDREP	1.91
ALKQLDRPEKGLSE	2.47
VCLALVRPCKMGLC	0.98
TPSELERPCRMVSR	1.46
GDSKLFRPRRPRSS	2.37
DGKFLLRPRKEQGT	2.12
HTKNLLRPTKLNLQ	1.79
ACEIIERPSKKDGF	-0.40
WICQICRPRKKGRK	2.66
ALRGLVRPHKKRKK	2.03
HALHPARPARDHLD	3.10
VRLPFHRPPRAGAW	2.66
VGASLTRPNRRLRW	2.25
MLQAPIRPPKPQSS	3.44
VCPGPIRPIRQISD	2.41
CEGSLTRPKKAIPA	2.11
AALLFLRPPRVRGH	1.92
HYLVPVRPRRQIVY	3.50
QAVHLPRPPRVLGL	2.25
GGHGPERPKRPGSL	3.12
AQPQPLRPPRRVSS	3.06
MTTTPERPRRGIPG	3.31
PHADPGRPERLVFK	0.92
PGNIPQRPDRLPMS	2.56
QHYTLGRPGRAPRR	1.86
PRKYPNRPSKTPLH	1.71
EQKRPLRPRRKGRK	3.00
CELRLSRPPKAYGS	1.12
ASPSLERPEKGAGK	2.24
DEAQLSRPKKSCDR	1.88
RKPELERPIKVNLA	2.09
EKPILDRPTRISQP	-0.22
RRQPPVRPGRVHLP	3.87
IGRNIARPWKLIYY	2.50
PHSAPLRPPRELHA	4.11
ELTIIERPVRRSLK	1.79
LKAPPARPVKGAYR	3.56
LTCGPARPWKSVAL	2.63
PPQVPSRPTRAPPR	4.04
CGGRPARPDRGPEP	3.40
SFSELVRPFKSNKS	2.03
PAGELCRPKKDMCD	2.29
NSHLLPRPLRHLAC	3.36
TSPAPHRPPKRGPL	3.60
GSECLSRPWRARAK	1.96
ARRLLVRPARLRRH	1.83
DGSFLARPSKSNPG	1.56
HLLLFHRPLRASGA	1.53
SPGFPARPGRGRPY	2.88
VCRDITRPPKTVAT	2.28
AKTPLSRPVKRNNA	2.17
QNRPLIRPKKRLIV	3.18
ICHLPCRPVKPNII	2.08
MDGFLCRPCRPLLQ	2.37
VQSIINRPRKSPLP	1.93
KDVLFIRPRKYIHC	1.94
TRESIVRPSRRGGI	1.95
RHQRLHRPGRPVPG	1.81
YRRRFCRPSRARDI	1.93
ASATLGRPPRIPEP	1.82
EVPDPNRPQRLGLH	2.36
WAGTLQRPVKLLPW	2.52
LGQKPQRPRRPASP	4.54
ARRPLARPIKRGDI	2.42
AAVAPSRPGREPQA	2.62
GWGSPGRPKKKHTG	2.50
SGERLPRPDKGKMR	1.68
LNCSFSRPDRPASV	2.09
LMIHPPRPPKVLGL	3.01
RTEGFWRPLRGVNV	2.03
SSVRPPRPKKEPQT	3.02
VKRRPGRPRKCPLQ	2.03
WSLKLCRPSKDYLL	3.36
SVIKPPRPDRGWFV	3.69
DNLKLFRPKKLLPK	1.85
WSEVLHRPWRNHPI	2.56
RAPRPTRPDKARRL	3.33
ATCHFYRPPRCSHC	2.34
PVVLPWRPARAHSC	3.33
LWRRFGRPAKCSTP	2.05
DPSGLPRPQKIPIS	2.05
SQGPPPRPGKPEGQ	2.01
MRKYPNRPSKTPPH	3.20
LLEQILRPCKFLTY	0.84
LVNGFLRPLRMAAS	2.25
GHQEFARPSRGDLA	1.72
LPEELPRPRRPPPE	2.17
ASFQPARPHRARHP	2.81
RVEELSRPKRFYLE	2.32
FVCPPNRPFRCKND	2.35
LRAMPCRPPRLLAT	3.85
DVPAFVRPARVEPT	1.86
ADAEPGRPKKRAHS	1.68
LRRHPSRPMRQSRR	2.61
VTLLIYRPERNTKL	1.46
MFEDPTRPGKQQQL	2.08
ARRGPGRPRKRKHS	2.36
DAQEIIRPRRCKYF	2.22
FHKIFIRPLRYRSR	3.47
LCHPLARPRRDAEP	2.61
SVIKPTRPDRGWFV	3.50
KKDCFERPRRVGAK	2.02
NRKPAEPTKPNNLD	1.96
NYQPKPPIREGNGP	1.90
SDTPASPSKVQSFD	1.71
VVLPKFPDKSFVLD	1.35
KTVPQSPKKPLSLL	1.25
FPYPGNPLKIINYE	1.94
LVTPEEPAKNKSER	1.90
MVTPREPKKRTTRK	2.21
PTAPNSPIKLGRRK	1.06
NNTPIVPEKFSNDL	0.58
ATTPAIPTKSDEAE	1.94
STRPQMPSRTRETA	1.87
FSKPQEPVKAEEAE	1.43
AQRPRGPQRGKDIK	1.90
DDTPSIPSKISSKS	2.39
ENAPANPSKALKVK	1.83
VKRPLSPTRINGKS	2.65
LHYPYGPARNPSYI	1.75
LIQPTAPIRDPTRF	1.88
LHGPFKPVKSSNGE	2.03
ENLPPSPVRAVNPL	1.46
TKVPSKPDRNSKIL	1.64
NSCPMSPLKEKYVL	1.84
TKVPSKPDRNSKIL	2.62
NSCPMSPLKEKYVL	2.65

FVCPPNRPFRCKND	3.57
LDPALLRPGRFDKL	2.97
LRAMPCRPPRLLAT	3.71
DVPAFVRPARVEPT	3.66
ADAEPGRPKKRAHS	2.08
LRRHPSRPMRQSRR	3.54
VTLLIYRPERNTKL	2.56
MFEDPTRPGKQQQL	1.27
ARRGPGRPRKRKHS	2.08
DAQEIIRPRRCKYF	2.86
FHKIFIRPLRYRSR	4.20
LCHPLARPRRDAEP	2.99
SVIKPTRPDRGWFV	3.70
KKDCFERPRRVGAK	3.41
RRPALHRPIKVNLL	2.05
GEDVLARPQRFAQL	2.29
ATDAPSRPGRLAGR	3.74
PEVGPWRPKRLSGV	3.67
LCQDLIRPIRKTGT	3.26
RLYSIHRPCKQCLN	1.57
GGDALGRPPRGARS	3.33
RHLAPNRPSKSRTD	2.72
KDLALLRPSKRQPP	1.82
GCVRPARPERRLRS	4.10
PRSVFLRPQRNLES	3.58
PLLHLTRPSRLEPG	3.25
KKPDIPRPIKINLL	2.20
EGQQLVRPKKLPLI	1.94
IKILPARPIKESEY	2.07
IPQRPSRPARRPLG	4.44
FGLVPERPLKQELG	2.81
TNSRLARPTRLSLG	3.25
CEGTLERPQRSRGR	2.97
SESRIRRPMNAFMV	0.00
FEEGIKRPYFHVKP	1.69
CEMLIKRPCKVKTI	1.80
LGLSFRRPNVITLL	1.58
TKIPFKRPRGHNFS	1.73
YGLTFRRPRGLFIT	1.37
DESRIRRPMNAFMV	1.84
ATGTLKRPTSLSRH	1.52
VDNTIKRPNPAPGT	0.87
LRRLLKRPELEEIF	1.56
AWVSPRRPDEEQRP	1.76
RGAQLRRPRHSHLT	1.40
GNIELKRPLIKAPS	1.41
VLGVLRRPLEAPPE	0.04
SLEVIRRPETKVLI	1.34
AAAVPRRPTQQGTV	1.59
MLAMPRRPDPVFLN	1.76
PFRALKRPLQFLGL	1.45
ARAALRRPLSGQAL	1.94
STQSLKRPGTSQAG	1.62
HRCHLRRPAAPPSP	1.76
PAPRLRRPGRVRPH	1.85
EGTEPRRPRAGPEE	1.56
WRKIPKRPGSVHRT	1.69
AVKSLKRPLEATFD	1.37
DPEDLKRPDPASLK	1.21
AREEFRRPRESLLN	1.30
PRPTPRRPRPRLAR	2.97
IDPALRRPGRFDRE	2.50
CTQGLRRPFGVAVM	0.95
HTVELRRPCSDMEG	1.70
LEDSPKRPKEAENP	1.10
LQRQLKRPRPVILD	1.62
GAPGPRRPFQQKPL	0.80
LLLHPRRPRLHPGT	1.94
ITGTLKRPSLPEEE	1.56
AVQFLRRPKTITRV	1.18
EPEEPKRPVPAERG	2.02
GKLKLKRPEQLQEV	1.51
MQFSIRRPKLPSSE	2.07
QIRILKRPTSNGVV	0.81
FCDFLKRPIALLKM	1.34
CEHNPKRPVTCEQG	1.52
AGSDPKRPEMPSKK	1.37
LLDTIKRPALQGLC	1.62
QVYVLKRPYVDEFL	0.82
YYQIIKRPMDLSII	1.89
VSVLPKRPRPAEFK	1.73
LSKWLKRPGALEKQ	1.32
PGLAPKRPLETGPR	1.50
MLLSPRRPTVEKRA	2.54
PAASPRRPAPRTGP	1.32
AAPLLRRPHGGAGP	1.41
ICQKIKRPANSGEL	1.66
QRCHFKRPRELEDA	1.81
EQGCLKRPVSRKVG	1.67
LRGKFKRPTLRRVR	1.37
LQPRPRRPREAADT	2.57
VSAALRRPGPSLVL	1.53
ALPLPRRPSALPPK	2.18
TPPQPKRPRPGVSK	2.54
GGVRLRRPSVNGEP	1.55
TTVLIRRPDLRYQL	1.47
FGDKFKRPIGSWEC	1.53
PCLTPRRPSSTRAR	2.94
FGAVLRRPHDPKTV	1.26
RVSWIRRPPGKALE	1.18
RRLALRRPEILVQP	0.97
SCGGFRRPEELGRV	1.62
FLDYLKRPGLTYLQ	1.17
LGEVPRRPASRGVP	1.35
EEASFRRPHPDGDV	1.60
VSPEPRRPAPAVSP	2.37
TPPLPRRPRKGSSE	3.46
SEAAPRRPEATAAP	1.42
LTVAIRRPTDDSNQ	1.47
VVELPRRPAPPAAV	1.55
NCQAPRRPWLASHM	1.95
GYPAFKRPHMTAKD	1.49
YPARPRRPASTAGS	3.61
EGHALRRPPPRWLL	1.82
ANLALRRPQGSTCR	1.10
SLKRPKRPLFNFAL	2.23
SDSKPKRPQPSREI	1.13
TLQAPRRPPTPGPD	1.55
SIWDPRRPLNTTEG	1.04
GPAGLRRPPDKQPS	1.44
NQSVPKRPAGSVQN	1.37
KEQEPKRPHIKKPL	1.59
SFLCLRRPWNWIAH	1.76
SSYTFKRPVTRITP	1.27
EDVLLRRPKCTPDM	1.59
ARARPRRPREETLT	1.63
AAMHPRRPDGFDGL	1.73
KVFLLRRPPQAPLP	1.32
PNATPRRPLQELRR	2.61
EKPKLRRPCDRSNG	1.00
AVMALRRPGERQYR	1.59
VPPGLRRPGPGTLY	1.62
SPARPRRPHACRDG	4.14
KRNSLKRPRDVDHR	1.45
CQKAFKRPSDLRQH	1.79
AVSAPKRPRVAPLQ	1.77
NEPHIKRPMNAFMV	1.66
MENNLRRPNLEAFN	1.43
PGGCPRRPPALAGP	1.67
NSSSFKRPDGEMNI	0.86
RPTFLRRPINQVVL	2.23
APPELRRPSHLHTS	1.52
MTVAIRRPGVPGAP	1.74
LYWLLRRPSGVVLC	2.25
PSPNFKRPYQRFQW	1.64
REVSLKRPLGNSPG	1.60
FRGCFRRPDNLSLA	1.87
ETSMLKRPVLLHLH	1.90
IFSTLKRPQVETKT	1.60
ETSMLKRPVLLHLQ	1.21
LEQAFRRPNVNLTS	1.63
FGRRPRRPVDGRVG	1.55
VEGILKRPPDEQRL	1.51
SRKNLKRPIAHLPC	1.78
YQRLPRRPGEPREV	2.06
MLANPRRPIAQWHT	2.03
DIKPLKRPRDEDDA	1.58
IGYDFRRPLAQLRE	1.62
PAQLPRRPAQRPGE	1.47
IYVCLKRPSWMVDN	1.76
VFLSFRRPFWREEH	1.11
WRRCLRRPDCRGRF	1.98
PTGSLRRPLGPHAP	1.20
VKMQLRRPSDQEVS	1.91
PFFAPKRPFFPPRY	2.33
PPQSPRRPELPDLA	1.71
AKREPRRPCWSPRL	1.67
SPDKFKRPTPPPSP	1.20
QGPAPRRPWERGDG	1.55
HTAEFRRPMATGDT	1.66
SYTRIKRPQDQLRF	1.59
GRPRPRRPAQYPAR	1.86
GKSHPKRPNSQSQH	1.66
AARTLRRPKALDCG	0.88
LEFPPRRPFARRRM	2.38
VLTALRRPARSSWH	3.27
MSTPLRRPSACETV	1.26
APPQIRRPNREVRP	3.51
AGPRPRRPGSLPGW	2.31
RSGNLKRPKIGKRF	1.13
ISFHPKRPVHILLH	1.44
PSDHIKRPMNAFMV	1.21
LAARLRRPSPPEPW	1.89
AVWSPRRPTATRDP	1.56
SLHGPRRPQPGASG	1.53
QKQDLRRPKIHGAV	1.75
FVTELRRPVAVRAK	1.60
EESLPKRPLGKHSK	1.51
EERALRRPGHLYLL	1.58
TDNKFRRPWTRVSA	1.61
AKINLKRPPTAGSQ	0.00
LTSFLRRPFQSPTS	1.85
PEYRPKRPANQFVP	1.39
RRPALHRPIKVNLL	2.04
GEDVLARPQRFAQL	0.88
ATDAPSRPGRLAGR	2.17
PEVGPWRPKRLSGV	3.38
LCQDLIRPIRKTGT	2.44
RLYSIHRPCKQCLN	2.46
GGDALGRPPRGARS	2.87
RHLAPNRPSKSRTD	2.41
KDLALLRPSKRQPP	2.35
GCVRPARPERRLRS	4.27
PRSVFLRPQRNLES	2.32
PLLHLTRPSRLEPG	3.09
KKPDIPRPIKINLL	2.49
EGQQLVRPKKLPLI	1.85
IKILPARPIKESEY	2.66
IPQRPSRPARRPLG	4.93
FGLVPERPLKQELG	2.45
TNSRLARPTRLSLG	3.28
CEGTLERPQRSRGR	2.10
SESRIRRPMNAFMV	1.52
FEEGIKRPYFHVKP	2.17
CEMLIKRPCKVKTI	1.68
LGLSFRRPNVITLL	2.48
TKIPFKRPRGHNFS	1.34
YGLTFRRPRGLFIT	2.54
DESRIRRPMNAFMV	1.97
ATGTLKRPTSLSRH	1.37
VDNTIKRPNPAPGT	1.71
LRRLLKRPELEEIF	1.77
AWVSPRRPDEEQRP	2.60
RGAQLRRPRHSHLT	1.73
GNIELKRPLIKAPS	1.46
VLGVLRRPLEAPPE	0.74
SLEVIRRPETKVLI	1.82
AAAVPRRPTQQGTV	2.26
MLAMPRRPDPVFLN	2.33
PFRALKRPLQFLGL	1.80
ARAALRRPLSGQAL	1.93
STQSLKRPGTSQAG	0.75
HRCHLRRPAAPPSP	1.74
PAPRLRRPGRVRPH	1.85
EGTEPRRPRAGPEE	0.69
WRKIPKRPGSVHRT	2.76
AVKSLKRPLEATFD	1.31
DPEDLKRPDPASLK	1.92
AREEFRRPRESLLN	1.44
PRPTPRRPRPRLAR	3.29
IDPALRRPGRFDRE	2.03
CTQGLRRPFGVAVM	1.43
HTVELRRPCSDMEG	1.44
LEDSPKRPKEAENP	0.41
LQRQLKRPRPVILD	2.29
GAPGPRRPFQQKPL	3.30
LLLHPRRPRLHPGT	3.19
ITGTLKRPSLPEEE	1.27
AVQFLRRPKTITRV	2.07
EPEEPKRPVPAERG	1.60
GKLKLKRPEQLQEV	1.55
MQFSIRRPKLPSSE	2.73
QIRILKRPTSNGVV	2.17
FCDFLKRPIALLKM	2.60
CEHNPKRPVTCEQG	1.29
AGSDPKRPEMPSKK	1.82
LLDTIKRPALQGLC	1.67
QVYVLKRPYVDEFL	1.45
YYQIIKRPMDLSII	1.93
VSVLPKRPRPAEFK	3.77
LSKWLKRPGALEKQ	1.83
PGLAPKRPLETGPR	3.13
MLLSPRRPTVEKRA	3.92
PAASPRRPAPRTGP	1.69
AAPLLRRPHGGAGP	2.01
ICQKIKRPANSGEL	1.38
QRCHFKRPRELEDA	1.47
EQGCLKRPVSRKVG	1.94
LRGKFKRPTLRRVR	1.58
LQPRPRRPREAADT	2.52
ALPLPRRPSALPPK	3.76
TPPQPKRPRPGVSK	2.40
GGVRLRRPSVNGEP	1.56
TTVLIRRPDLRYQL	1.21
FGDKFKRPIGSWEC	1.66
PCLTPRRPSSTRAR	3.14
FGAVLRRPHDPKTV	1.71
RVSWIRRPPGKALE	2.51
RRLALRRPEILVQP	1.50
SCGGFRRPEELGRV	1.06
FLDYLKRPGLTYLQ	0.90
LGEVPRRPASRGVP	3.02
EEASFRRPHPDGDV	-0.15
VSPEPRRPAPAVSP	1.02
TPPLPRRPRKGSSE	3.37
SEAAPRRPEATAAP	1.21
LTVAIRRPTDDSNQ	2.20
VVELPRRPAPPAAV	1.78
NCQAPRRPWLASHM	3.84
GYPAFKRPHMTAKD	1.99
YPARPRRPASTAGS	3.30
ANLALRRPQGSTCR	2.51
SLKRPKRPLFNFAL	3.34
SDSKPKRPQPSREI	1.65
TLQAPRRPPTPGPD	3.28
SIWDPRRPLNTTEG	1.62
GPAGLRRPPDKQPS	2.58
NQSVPKRPAGSVQN	1.42
KEQEPKRPHIKKPL	1.81
SFLCLRRPWNWIAH	2.70
SSYTFKRPVTRITP	1.95
EDVLLRRPKCTPDM	1.21
ARARPRRPREETLT	2.26
AAMHPRRPDGFDGL	1.15
KVFLLRRPPQAPLP	3.00
PNATPRRPLQELRR	3.20
EKPKLRRPCDRSNG	1.91
AVMALRRPGERQYR	2.31
VPPGLRRPGPGTLY	1.64
SPARPRRPHACRDG	3.94
CQKAFKRPSDLRQH	1.28
AVSAPKRPRVAPLQ	2.38
MENNLRRPNLEAFN	1.17
PGGCPRRPPALAGP	2.38
NSSSFKRPDGEMNI	1.60
RPTFLRRPINQVVL	2.76
APPELRRPSHLHTS	2.16
MTVAIRRPGVPGAP	1.90
LYWLLRRPSGVVLC	2.20
PSPNFKRPYQRFQW	1.85
REVSLKRPLGNSPG	1.75
FRGCFRRPDNLSLA	2.04
ETSMLKRPVLLHLH	2.64
IFSTLKRPQVETKT	1.57
ETSMLKRPVLLHLQ	2.26
LEQAFRRPNVNLTS	1.63
FGRRPRRPVDGRVG	1.92
VEGILKRPPDEQRL	1.31
SRKNLKRPIAHLPC	1.53
YQRLPRRPGEPREV	2.15
MLANPRRPIAQWHT	2.09
IGYDFRRPLAQLRE	2.31
PAQLPRRPAQRPGE	1.45
IYVCLKRPSWMVDN	1.67
VFLSFRRPFWREEH	1.14
WRRCLRRPDCRGRF	2.34
PTGSLRRPLGPHAP	1.54
VKMQLRRPSDQEVS	2.58
PFFAPKRPFFPPRY	3.49
PPQSPRRPELPDLA	2.21
AKREPRRPCWSPRL	2.29
SPDKFKRPTPPPSP	1.56
HTAEFRRPMATGDT	1.27
SYTRIKRPQDQLRF	2.11
GRPRPRRPAQYPAR	2.81
LEFPPRRPFARRRM	3.48
VLTALRRPARSSWH	2.60
MSTPLRRPSACETV	2.36
APPQIRRPNREVRP	2.80
AGPRPRRPGSLPGW	2.91
RSGNLKRPKIGKRF	1.70
ISFHPKRPVHILLH	2.24
PSDHIKRPMNAFMV	1.93
LAARLRRPSPPEPW	3.07
AVWSPRRPTATRDP	1.99
SLHGPRRPQPGASG	2.41
QKQDLRRPKIHGAV	1.93
FVTELRRPVAVRAK	2.07
EESLPKRPLGKHSK	2.30
EERALRRPGHLYLL	1.17
TDNKFRRPWTRVSA	1.31
AKINLKRPPTAGSQ	1.77
LTSFLRRPFQSPTS	3.19
PEYRPKRPANQFVP	2.38
SSNTIRRPRNYSVG	0.87
SPPELKRPRLDENK	1.89
DGDPLKRPLLGIVQ	1.62
TNFAFRRPCFESLK	2.62
RCWVLRRPMLAAAG	2.61
PGRAPRRPLLEGGG	2.85
TRVQPRRPTKGTHR	2.14
TPRGPRRPSGPAEL	1.48
RRLQPRRPGYPPRP	2.51
SGLAPKRPGPAGEL	1.05
ASGHIKRPMNAFMV	2.17
SLALPRRPQNRDSI	2.94
SSVALKRPPPAPPV	1.33
GYDRLRRPGCRCKH	2.15
PCSRPRRPAVYTSV	2.41
CGKAFRRPSDLFRH	1.43
EHRPLKRPRPRLGP	1.91
ELEQLRRPPGTAER	2.10
GVISLKRPFINLTA	1.55
KQASLKRPPTAGAQ	1.74
EGAAPRRPGFWGSL	3.19
PIQNIKRPYPGEVS	2.04

SSNTIRRPRNYSVG	1.50
SPPELKRPRLDENK	1.71
DGDPLKRPLLGIVQ	1.27
TNFAFRRPCFESLK	1.66
RCWVLRRPMLAAAG	1.28
PGRAPRRPLLEGGG	1.65
TRVQPRRPTKGTHR	1.58
TPRGPRRPSGPAEL	1.50
RRLQPRRPGYPPRP	1.76
SGLAPKRPGPAGEL	1.36
ASGHIKRPMNAFMV	1.69
SLALPRRPQNRDSI	1.98
SSVALKRPPPAPPV	1.39
GYDRLRRPGCRCKH	1.49
PCSRPRRPAVYTSV	1.60
CGKAFRRPSDLFRH	1.45
EHRPLKRPRPRLGP	1.34
ELEQLRRPPGTAER	1.20
GVISLKRPFINLTA	1.21
KQASLKRPPTAGAQ	0.72
EGAAPRRPGFWGSL	1.47
PIQNIKRPYPGEVS	1.69
CDGNPRRPSPVLRH	1.78
PSRFIRRPSTTICP	1.62
RQLRLRRPDDGSRE	1.60
RLPELKRPSGDQVQ	1.57
LLGKPKRPRSAYNV	1.66
TTVLIKRPDLKYQL	1.48
SITQLRRPSQLLYP	0.96
HILLFRRPLPKDQQ	1.08
SLMTPRRPLSTSEK	1.85
LTLNIRRPSPFDAG	0.91
QVNHLKRPVSGVGD	1.63
SLASPRRPLLCDFA	1.90
RYLILKRPGFQEHD	1.38
RHPRPRRPGGPALR	1.96
LFQPPRRPGLGTVG	3.18
RPNPLRRPLNRKRA	2.19
GKPNPRRPKDKMSA	1.13
VNVVFRRPLRPADP	2.89
SALRPKRPETPKQT	2.77
GESRIRRPMNAFMV	1.67
QQFRLRRPSGITWP	1.79
KPQRPRRPASPIST	3.41
PPSAPRRPPVYYKF	1.85
DPNAPKRPLSGFFL	1.61
AEEQLRRPRIVHVG	1.39
GVPVPKRPAEGGGG	1.24
TEDTPRRPPEPKPI	1.23
QAATPRRPAAISRP	2.25
LSFHPKRPWILTSL	1.37
PMRLPRRPLRAFES	3.72
FLGHIKRPWGICVG	1.53
ATPLPRRPQPRDLG	2.12
AAVTLKRPPPYQWD	1.62
EDSIPKRPVSSIPL	1.53
VPRDLRRPASLIGQ	1.41
PQTELRRPGQLIDE	1.44
MLDMFKRPTDPPEY	1.54
PDVWLRRPSTHTSG	1.78
VEPPPRRPMSQKSE	2.39
DPNAPKRPMSAYML	1.62
RLIAPRRPWTLSHV	3.05
TSWMPRRPSCPLKE	1.56
CALCLRRPHCGWCA	1.43
SCKIFRRPSLPCIS	1.09
LLCWIKRPSQRALR	1.46
GYDSLRRPGCRCKR	1.49
LAHILRRPIIVYGV	1.66
QRKNLRRPPCIKGW	1.40
RAITFRRPSRVHYY	2.85
PCAKPRRPQDKSQG	1.58
PQPPPRRPWASRVL	1.90
HPLVPKRPDACFMA	2.10
VCRLPKRPEPCPPL	1.48
PAALLRRPGLGRLV	0.69
RHLILKRPGFQEHE	1.83
SGGRLRRPVNVSLD	1.76
GEPSPKRPRGRPKG	1.37
AAPEPKRPHIAQVP	0.88
IRSALKRPVEQPLG	1.42
SVSGPKRPQDRVAV	1.59
ALPHFRRPFFEEAA	1.64
PRANPRRPPRAIVP	2.57
RAASLRRPRRAASR	1.90
GEQTFKRPAEDTLY	1.18
RRGCPRRPRLSSCS	1.61
GAGQPRRPEAEPGQ	1.02
SGAEIKRPGASVKV	1.54
TQISLKRPRHRSVG	0.81
EQFKIKRPAAWVDL	0.63
LSSPLRRPWDQAQH	2.00
EIAKLKRPTALKKV	1.21
YKVNLKRPCPFWAE	1.49
LVIRLRRPPLRCPR	1.96
NLFGPKRPKALKLL	2.05
SVNHIKRPHSVPSS	1.22
FQEFLKRPDHKQDF	1.52
WLGSLRRPSLVHGY	2.94
ALCHFRRPDVSVQG	1.29
EHPEPKRPSVDSLV	1.70
LIQELRRPKYSIYF	1.67
CPPIPRRPAEPPWP	1.52
GSVELRRPPAHQVM	1.74
PPRKIKRPKRKMYR	1.24
AWQQLRRPEASETL	1.47
YERTLRRPVPLPHS	1.57
TYSLLRRPFRQVLA	2.84
YQEYLKRPPESPPI	1.53
QQNLPKRPTSLPLN	1.68
VKPGLRRPPASPAL	1.52
NKVPIKRPSTGLLM	0.82
MCRVFKRPFSEPSA	1.95
WVTMPRRPPPSGGL	1.49
CGATLKRPMEFEAA	1.50
SAVVLKRPEILTDE	1.63
YENKPRRPYILKRD	1.71
RLHRLRRPHSSSDA	1.10
FNFDFRRPGPEPPR	1.42
FLTRLRRPIGKMTI	1.15
GESRLKRPSVLISA	1.50
PGTPPRRPASPSAL	2.35
AVAVPRRPRAGAER	2.02
KLEDPRRPDTSFLW	1.66
FSHLLRRPPPEAPR	2.23
LPARPRRPTPTMVP	4.03
ISRRLRRPPHVGRA	1.75
LDMALRRPPRLRLC	3.62
TSGSFRRPESPRPR	1.76
DPQAPRRPPSSFLL	2.03
LTIELRRPENEFWV	1.78
YPNKIKRPCLEDVT	1.40
SQNEPKRPMSGEYG	1.23
SGGKIRRPANVPID	0.00
VGIQLRRPLFQKVT	1.56
CKAYPKRPLLGLVL	1.10
ETMYLRRPEGIERQ	1.31
RPSFLRRPVNQVVL	1.64
SGGRIRRPVNVPID	1.46
SLPRPRRPLPTQQQ	2.71
DLPGPRRPAAAAAM	1.52
IQVAFRRPETPSSG	1.75
LLYTLRRPYFRKME	1.61
GHRDPRRPRPAQLP	1.11
ASPRLRRPKSPKLP	2.04
EALKLRRPPDASGA	1.56
RVLHLRRPGGPPHV	1.33
LETSLKRPKRPLFN	3.15
EGAVPRRPEDALLL	1.86
PLPAPRRPKVNSIL	3.67
LCGLIKRPGDLPEV	1.34
SPSVPKRPKLPAPT	3.25
THCIIKRPDNQHVI	1.59
AKDLLRRPFDLMLV	1.50
MLANPRRPIAQWHS	1.53
PQSSLRRPDPGRLP	1.60
KLFRPRRPRSSSDA	1.67
KKPPLRRPHSVIGG	1.20
NNRVLRRPSERGRA	2.18
SGGIPKRPSSPVIS	1.66
LTSGLRRPFGVAVM	1.55
GPPSPRRPLVPAGP	1.32
DRFPLKRPRYEDSD	2.11
DVTDPKRPLSFGVT	0.23
LVSRLRRPVSEVSG	2.05
MLLRPRRPPPLAPP	3.42
NCFIPKRPIPTIKD	1.69
KSPIPRRPTQSFVK	1.39
GQRRLRRPLILGLG	1.63
QPALLKRPQLELYV	1.45
KRIKLRRPRVCKQR	1.09
PSNPIRRPISADSA	1.55
ISELIKRPNVSPKV	1.49
EWIVFRRPVPISYH	1.98
SSGILKRPQTLAIP	1.85
DEVWFKRPDGSKSK	1.48
ERDFLKRPLESYVK	1.63
LHLVIRRPLETHPG	1.69
AEERPRRPSQEQSA	1.24
IFVAPKRPCPLPRA	3.62
WALFFRRPKASLPR	2.29
EEAALKRPEIISTV	1.26
DMQSFRRPSAASID	1.22
PNLIPRRPSPYEKT	2.12
LLMVPRRPGYGAMG	2.26
DEGSLRRPGRLTST	1.91
LGKVIRRPPLTEKL	0.67
RRARPRRPREAPAP	1.55
PTKSIKRPSPAEKS	0.87
GRAWLRRPHPRDVG	0.32
AASGIKRPMASEVL	1.69
TLLFPKRPFSWIRH	2.09
LLFCLRRPSSTVTM	1.74

CDGNPRRPSPVLRH	2.76
PSRFIRRPSTTICP	2.56
RQLRLRRPDDGSRE	1.79
RLPELKRPSGDQVQ	1.54
LLGKPKRPRSAYNV	2.43
TTVLIKRPDLKYQL	1.87
SITQLRRPSQLLYP	1.56
HILLFRRPLPKDQQ	1.60
SLMTPRRPLSTSEK	3.17
LTLNIRRPSPFDAG	2.20
SLASPRRPLLCDFA	3.23
RYLILKRPGFQEHD	2.36
RHPRPRRPGGPALR	2.43
LFQPPRRPGLGTVG	3.26
RPNPLRRPLNRKRA	2.30
GKPNPRRPKDKMSA	1.94
VNVVFRRPLRPADP	2.42
SALRPKRPETPKQT	2.86
GESRIRRPMNAFMV	2.14
QQFRLRRPSGITWP	2.07
KPQRPRRPASPIST	3.96
PPSAPRRPPVYYKF	3.44
DPNAPKRPLSGFFL	3.23
AEEQLRRPRIVHVG	1.55
GVPVPKRPAEGGGG	1.95
TEDTPRRPPEPKPI	1.23
QAATPRRPAAISRP	3.14
LSFHPKRPWILTSL	2.08
PMRLPRRPLRAFES	3.08
FLGHIKRPWGICVG	2.07
ATPLPRRPQPRDLG	3.78
AAVTLKRPPPYQWD	1.79
EDSIPKRPVSSIPL	1.75
VPRDLRRPASLIGQ	1.29
PQTELRRPGQLIDE	1.64
MLDMFKRPTDPPEY	1.75
PDVWLRRPSTHTSG	2.38
VEPPPRRPMSQKSE	2.41
DPNAPKRPMSAYML	1.92
RLIAPRRPWTLSHV	3.35
TSWMPRRPSCPLKE	2.84
CALCLRRPHCGWCA	1.68
SCKIFRRPSLPCIS	1.61
LLCWIKRPSQRALR	2.75
GYDSLRRPGCRCKR	2.15
LAHILRRPIIVYGV	2.80
QRKNLRRPPCIKGW	1.76
RAITFRRPSRVHYY	1.53
PCAKPRRPQDKSQG	1.89
PQPPPRRPWASRVL	2.71
HPLVPKRPDACFMA	3.29
VCRLPKRPEPCPPL	3.56
PAALLRRPGLGRLV	1.91
RHLILKRPGFQEHE	2.11
SGGRLRRPVNVSLD	1.74
GEPSPKRPRGRPKG	1.77
AAPEPKRPHIAQVP	2.04
SVSGPKRPQDRVAV	1.83
ALPHFRRPFFEEAA	1.98
PRANPRRPPRAIVP	1.97
RAASLRRPRRAASR	1.86
GEQTFKRPAEDTLY	1.20
RRGCPRRPRLSSCS	1.05
SGAEIKRPGASVKV	1.51
TQISLKRPRHRSVG	1.44
EQFKIKRPAAWVDL	0.00
EIAKLKRPTALKKV	1.93
YKVNLKRPCPFWAE	1.63
LVIRLRRPPLRCPR	4.11
NLFGPKRPKALKLL	3.29
SVNHIKRPHSVPSS	2.13
FQEFLKRPDHKQDF	1.52
WLGSLRRPSLVHGY	3.43
ALCHFRRPDVSVQG	1.70
LIQELRRPKYSIYF	2.23
CPPIPRRPAEPPWP	3.14
GSVELRRPPAHQVM	1.72
PPRKIKRPKRKMYR	1.48
AWQQLRRPEASETL	1.20
YERTLRRPVPLPHS	2.47
TYSLLRRPFRQVLA	2.85
YQEYLKRPPESPPI	1.63
QQNLPKRPTSLPLN	2.72
VKPGLRRPPASPAL	1.54
NKVPIKRPSTGLLM	2.56
MCRVFKRPFSEPSA	2.80
WVTMPRRPPPSGGL	3.38
SAVVLKRPEILTDE	1.58
YENKPRRPYILKRD	2.32
RLHRLRRPHSSSDA	0.98
FNFDFRRPGPEPPR	2.00
FLTRLRRPIGKMTI	2.58
PGTPPRRPASPSAL	3.57
AVAVPRRPRAGAER	2.41
KLEDPRRPDTSFLW	1.35
FSHLLRRPPPEAPR	3.72
LPARPRRPTPTMVP	3.84
ISRRLRRPPHVGRA	0.77
LDMALRRPPRLRLC	3.08
TSGSFRRPESPRPR	1.83
DPQAPRRPPSSFLL	3.08
LTIELRRPENEFWV	1.72
YPNKIKRPCLEDVT	1.98
SQNEPKRPMSGEYG	1.33
SGGKIRRPANVPID	1.98
VGIQLRRPLFQKVT	1.89
CKAYPKRPLLGLVL	2.59
ETMYLRRPEGIERQ	2.11
RPSFLRRPVNQVVL	2.23
SGGRIRRPVNVPID	2.02
SLPRPRRPLPTQQQ	3.49
DLPGPRRPAAAAAM	1.82
IQVAFRRPETPSSG	0.75
LLYTLRRPYFRKME	2.00
GHRDPRRPRPAQLP	2.62
ASPRLRRPKSPKLP	1.54
EALKLRRPPDASGA	1.63
RVLHLRRPGGPPHV	1.74
LETSLKRPKRPLFN	1.97
PLPAPRRPKVNSIL	2.87
LCGLIKRPGDLPEV	1.69
SPSVPKRPKLPAPT	4.34
THCIIKRPDNQHVI	1.75
AKDLLRRPFDLMLV	1.85
MLANPRRPIAQWHS	2.74
PQSSLRRPDPGRLP	1.58
KLFRPRRPRSSSDA	2.50
KKPPLRRPHSVIGG	1.91
NNRVLRRPSERGRA	2.81
SGGIPKRPSSPVIS	3.03
LTSGLRRPFGVAVM	1.63
GPPSPRRPLVPAGP	3.45
DRFPLKRPRYEDSD	2.16
DVTDPKRPLSFGVT	1.57
LVSRLRRPVSEVSG	1.99
MLLRPRRPPPLAPP	3.72
NCFIPKRPIPTIKD	2.74
KSPIPRRPTQSFVK	2.76
GQRRLRRPLILGLG	1.97
QPALLKRPQLELYV	1.72
KRIKLRRPRVCKQR	1.32
PSNPIRRPISADSA	1.68
ISELIKRPNVSPKV	1.17
EWIVFRRPVPISYH	2.75
SSGILKRPQTLAIP	1.02
DEVWFKRPDGSKSK	1.78
ERDFLKRPLESYVK	0.65
LHLVIRRPLETHPG	2.78
AEERPRRPSQEQSA	2.95
IFVAPKRPCPLPRA	3.86
WALFFRRPKASLPR	3.23
EEAALKRPEIISTV	1.18
DMQSFRRPSAASID	1.75
PNLIPRRPSPYEKT	2.60
LLMVPRRPGYGAMG	3.51
DEGSLRRPGRLTST	1.45
LGKVIRRPPLTEKL	2.44
RRARPRRPREAPAP	2.16
PTKSIKRPSPAEKS	1.88
GRAWLRRPHPRDVG	1.74
AASGIKRPMASEVL	2.07
TLLFPKRPFSWIRH	3.20
LLFCLRRPSSTVTM	2.73
DPNTFKRPQTPPKI	1.60
PMEGIRRPDQALLP	1.82
RPPPPRRPQGPPPP	3.58
PMVPPRRPWTLVNW	2.81
RLRHLRRPRVARLL	1.39
ATPLPRRPQPRQFF	4.30
VFQAPRRPGIGTVG	2.86
GPERPKRPGSLLAP	4.09
RWKEPKRPRPLKLS	2.62
NQKQLKRPAQGKRQ	1.84
AARGLRRPEDSSRK	0.56
DFYEPRRPEDDDPT	1.70
AVGPPKRPQPAALE	2.59
ETGALRRPQLLPLL	2.10
PAVILRRPNQSQPL	2.04
APELLKRPKEYTVR	1.47
ASSSPKRPSVERSN	2.28
PRKAPRRPGPGMCG	2.34
EASIFRRPYYVSHF	2.32
FAPEFRRPKNIHLR	2.31
NEDYPRRPLTRARS	2.84
GGPRPRRPLLLPNL	3.47
LLLGIRRPEFLSRP	3.13
ALRWLRRPEDRAFS	2.55
CNKGFRRPSEKNQH	1.75
PSIIFRRPEEAALS	1.68
IKILLRRPNEKKSK	1.91
HPDLLRRPGARSTS	-0.70
SLRKLKRPPPPPRR	2.30
SFLRLRRPWNWIAH	2.55

DPNTFKRPQTPPKI	1.44
PMEGIRRPDQALLP	1.56
RPPPPRRPQGPPPP	1.55
PMVPPRRPWTLVNW	3.46
RLRHLRRPRVARLL	1.18
ATPLPRRPQPRQFF	2.18
VFQAPRRPGIGTVG	2.19
GPERPKRPGSLLAP	3.39
RWKEPKRPRPLKLS	1.76
NQKQLKRPAQGKRQ	0.00
AARGLRRPEDSSRK	1.26
DFYEPRRPEDDDPT	0.00
AVGPPKRPQPAALE	1.50
ETGALRRPQLLPLL	1.60
PAVILRRPNQSQPL	1.22
APELLKRPKEYTVR	1.45
ASSSPKRPSVERSN	1.09
PRKAPRRPGPGMCG	1.67
EASIFRRPYYVSHF	1.77
FAPEFRRPKNIHLR	1.62
NEDYPRRPLTRARS	2.59
GGPRPRRPLLLPNL	1.61
LLLGIRRPEFLSRP	1.71
ALRWLRRPEDRAFS	1.68
CNKGFRRPSEKNQH	1.64
PSIIFRRPEEAALS	1.53
IKILLRRPNEKKSK	1.57
HPDLLRRPGARSTS	1.48
SLRKLKRPPPPPRR	1.89
SFLRLRRPWNWIAH	2.08
FVNEIRRPNGENYA	1.38
SPEAPRRPKQRPAP	1.79
TELLPKRPQHLPGH	1.54
SAPGPRRPAPALLA	2.61
RLKTLRRPRVRDRL	1.55
LLMVPRRPGYGTMG	2.91
ILDTLRRPKPVVDP	1.42
HGRAPRRPSGPGPP	1.55
KGAPFRRPLYTNVP	1.56
RRSPPRRPAWTLGI	1.95
LQFRIRRPRPPARG	1.93
ISHRLRRPGAPPST	0.99
VMQSLRRPSCCGCS	1.66
SGGRLKRPVNVPFD	1.06
VLQHLRRPVALQLD	1.64
PHKRPRRPHQIADL	1.88
LELALKRPRPPVLS	1.47
TAVPPKRPYGTQRT	1.58
YHLPLRRPSPGFSW	2.13
KPRQIKRPLEDAIE	1.76
RPSTLRRPAYRDNQ	1.65
ATFCLRRPWTEAGA	1.32
LPKHLRRPEPSKPQ	1.20
PKERLRRPETSGIS	1.59
PKRGPRRPKYSPPR	1.08
NKVHIKRPTTGLLM	1.51
GPLGLRRPPCASSD	1.06
GYQAFRRPCYSWGV	1.54
FIKIPRRPDQPGKG	2.07
LDPALRRPSQKVYR	1.58
ILSPPRRPRHSQPA	2.54
YSLLFRRPPPSPSP	2.31
PGTSPRRPSVVPCR	1.55
VSHLIRRPISVNPN	1.57
GAKGPRRPGEEAEL	0.00
GGRTPRRPGLQSGG	1.45
LKEILKRPVDPIRA	0.76
SGTPIRRPMGQMKP	0.52
DIPEPRRPVVEQAL	1.57
APPVLKRPILQSLL	1.59
GSALPRRPLLCIPA	1.56
AQQLLKRPRGPSPL	1.74
DSPLPRRPAFSGTS	1.06
VTCRFKRPVNVPRD	1.52
LGLLFRRPEALQVG	1.09
PEVQLRRPNWSKLV	1.55
PNYDLRRPHQYLHP	1.39
PWQRPKRPGHGHGW	1.63
TPLSLKRPPPYDAV	1.28
RAVDLRRPLLHELL	2.06
QGASIRRPSSGNAS	1.53
RRCRLRRPAVRPTD	1.62
PAARPRRPPTTLPP	2.04
AGSSPRRPQGGSDA	1.32
CEEHPRRPDHGRRG	1.24
DTYRPKRPTTLNLF	1.88
LFFELRRPPQHGVL	1.10
WTEDPRRPLPWALS	1.58
RTLGLRRPVHSSCI	1.31
ASPTPKRPSENMLI	1.64
FMCSPRRPRSRGRQ	3.05
SCFIPKRPIPTIKD	1.25
ETLTFKRPEGMHRE	1.47
IYSTLKRPQVETKI	1.30
RASSPKRPGPWSEP	1.69
VAWHPKRPLLAFAC	1.52
RPPNLKRPEDYEAV	1.08
TEGRLRRPQQQQER	1.48
PAPRIKRPRLQVVK	1.85
PFHSLKRPRPPFAR	1.82
KPEGLRRPPSKTSM	1.51
GLLSLRRPAPRRLE	1.12
DPNPPKRPLIFDTN	1.31
IVKQIRRPKHVVVK	1.11
LPFYFKRPPINVAI	1.21
RTPAPRRPLQRRRP	2.90
GLEVPRRPGARTGP	1.47

FVNEIRRPNGENYA	1.11
SPEAPRRPKQRPAP	2.50
TELLPKRPQHLPGH	1.34
SAPGPRRPAPALLA	3.53
RLKTLRRPRVRDRL	1.51
LLMVPRRPGYGTMG	3.11
HGRAPRRPSGPGPP	4.05
KGAPFRRPLYTNVP	2.22
RRSPPRRPAWTLGI	3.20
LQFRIRRPRPPARG	1.69
ISHRLRRPGAPPST	2.08
VMQSLRRPSCCGCS	3.07
SGGRLKRPVNVPFD	1.58
VLQHLRRPVALQLD	1.77
PHKRPRRPHQIADL	1.76
LELALKRPRPPVLS	2.11
TAVPPKRPYGTQRT	2.60
YHLPLRRPSPGFSW	3.10
KPRQIKRPLEDAIE	1.49
RPSTLRRPAYRDNQ	2.05
ATFCLRRPWTEAGA	1.79
LPKHLRRPEPSKPQ	1.11
PKERLRRPETSGIS	1.51
PKRGPRRPKYSPPR	1.92
NKVHIKRPTTGLLM	2.10
GPLGLRRPPCASSD	2.06
GYQAFRRPCYSWGV	2.36
FIKIPRRPDQPGKG	2.68
LDPALRRPSQKVYR	2.03
ILSPPRRPRHSQPA	2.90
YSLLFRRPPPSPSP	2.24
PGTSPRRPSVVPCR	2.77
VSHLIRRPISVNPN	2.58
GAKGPRRPGEEAEL	1.60
GGRTPRRPGLQSGG	1.86
LKEILKRPVDPIRA	1.74
SGTPIRRPMGQMKP	2.17
DIPEPRRPVVEQAL	1.62
APPVLKRPILQSLL	2.49
GSALPRRPLLCIPA	3.33
AQQLLKRPRGPSPL	1.88
DSPLPRRPAFSGTS	2.99
VTCRFKRPVNVPRD	1.52
LGLLFRRPEALQVG	1.79
PEVQLRRPNWSKLV	2.18
PWQRPKRPGHGHGW	2.31
TPLSLKRPPPYDAV	1.83
RAVDLRRPLLHELL	1.83
QGASIRRPSSGNAS	2.09
RRCRLRRPAVRPTD	1.45
PAARPRRPPTTLPP	3.86
AGSSPRRPQGGSDA	1.71
CEEHPRRPDHGRRG	1.74
DTYRPKRPTTLNLF	3.19
LFFELRRPPQHGVL	2.23
WTEDPRRPLPWALS	2.04
RTLGLRRPVHSSCI	1.69
ASPTPKRPSENMLI	1.64
FMCSPRRPRSRGRQ	3.06
SCFIPKRPIPTIKD	2.71
IYSTLKRPQVETKI	2.06
RASSPKRPGPWSEP	1.82
VAWHPKRPLLAFAC	2.43
RPPNLKRPEDYEAV	1.83
TEGRLRRPQQQQER	0.00
PFHSLKRPRPPFAR	2.36
KPEGLRRPPSKTSM	1.61
GLLSLRRPAPRRLE	2.70
DPNPPKRPLIFDTN	1.52
IVKQIRRPKHVVVK	1.64
LPFYFKRPPINVAI	1.86
RTPAPRRPLQRRRP	4.08
GLEVPRRPGARTGP	2.80

Table S2. Sequences and affinities of 7 PDZ-binding peptide panels (Vouilleme L, et al. *Angew Chem Int Ed Engl 2010,* 49, 9912-9916; Boisguerin P, et al. *Chem Biol* 2004, 11, 449-459)

Human CALP PDZ (1/1) [80]

Peptide	LogBLU
QAEEDWTCTIL	5.16
KRNTLYFSTDV	4.90
TIKVIKAKKKT	5.03
QKVKEKLKIDS	4.89
GSPIHSLETSL	4.87
RVAFKLRINKT	5.01
SNVKIVKVKKE	5.06
KRQSILFSTEV	4.86
LFLVLASRTQL	4.97
NVISERNSTTV	4.99
FGFDVGPVCFL	4.85
FGVDVGPVCFL	4.84
FGVEIGPVCFV	5.00
SSIKVVLRKEV	4.99
LQDSRVYVSSL	5.03
LADEMICITTL	5.09
NNPANRRTTPV	5.03
ETEEEVQDTRL	5.01
EQSDVRFSSAV	5.09
KGGALQSCTII	4.92
KINLSQKETSI	4.93
NSRPHTNETSL	5.37
SFHSIKQSTAV	5.18
SNQLAWFDTDL	4.92
QVNKVWDQSSV	5.00
RPRDHVKKTIL	4.98
IGTMFLRETSL	4.83
DMMLEMLCTKI	4.94
WGMGSQSCTLL	5.01
TENFPLKLCRI	4.92
KELKTVTFSKL	5.01
QSLAGSPSSKL	4.93
RELLKELRTQV	4.87
FRTSLPKSCDL	5.07
SVKYRLKFRFG	5.07
GPGWLLSWCSL	4.85
FREKKFFCTIL	5.39
PFRPQKVCSFL	5.11
LRLCIPSTGPL	5.21
HSEASGMVTQL	4.87
EQQRAEDITRL	5.19
RLPEAPKLTHL	4.85
VACRLAQLTRV	5.28
GGKGGLCQSSL	4.88
RHSRSSLNTVV	4.87
GSSSGHTSTTL	5.18
LRTLLLQQSNV	5.00
RRVRKLPSTTL	5.03
RGMKRLTSTRL	4.90
RGIKKITSTAL	4.88
SQKVAVYSTCL	4.82
TLDSQIQETSI	4.90
WCIQFNRGSRL	4.87
FVPWIKSVTKL	4.89
DWAKEKQHTRL	5.11
ALPAHHNATRL	5.00
EWAKERKLTRL	4.99
NALEEPKGTRL	5.46
HHHHTLPGSVQ	5.17
ARLVKGERTNL	4.90
NWIKRRPQTKL	5.27
IAKADAQESRL	4.90
ENLIEFIRSRL	4.89
DEDQHTQITKV	4.84
TERRSKSCTIL	5.02
SSTCILQETSL	5.33
AANGLMQTSKL	4.88
IHQNPGEFCVL	4.82
IHFTRRAITDL	4.93
DGQEEQVTTRL	5.31
QISEEYMQTVL	4.99
QSFLQTETSVI	4.83
RVVTRPCQCQV	4.84
SYRRPPSATCV	5.14
FIPTLFCLCRL	4.86
NFIFDEFRTVL	4.85
PWSPRLESSKI	5.09
SRLERIESTEI	4.95
CGKGFSYSSVL	5.07
DGYDWGPATDL	4.89

Human NHERF1 PDZ (1/2) [77]

Peptide	LogBLU
DEAEAGEGGEN	5.07
AQWVDS--TSL	5.56
CSLAPNIISQL	5.19
EWLVETLKSRQ	5.06
GDFREDDDTAL	5.86
GSPLHSLETSL	5.84
GRNCSTNDSLL	5.49
GPHSFVTSS-L	5.33
EMGAIKNLTSF	5.52
NRVGFAEAARL	5.40
LQDSRV-VSSL	5.44
ESENATSLTTF	5.37
ETEEEVQDTRL	5.95
SLQERGGASLK	4.99
PKNPATKQQKQ	5.29
SGPASETLDCS	5.26
GPQAQAENTAF	5.85
NSRPHTNETSL	5.36
SSKARSDDLSV	5.24
NKAKHDELT-F	5.53
WGMGSQSCTLL	5.88
DTISFTQTSQF	5.47
VEKRKANFKDQ	5.06
NISSTSEPKEE	5.64
VSLAPVVTTCA	5.33
LSSMPSASKTG	4.99
SIVAWTGMLIA	5.59
KMHIFFTTFAL	5.90
WNLSIKKEWTE	5.02
RSLSNINSEML	5.39
LPWIKRTMKRL	5.66
SDSEEELPTRL	5.93
SLEEARKIFRF	5.02
ALEEARKIFRF	5.54
NIAVAG-STRL	5.79
HSEASGMVTQL	5.64
LNTTDKEST-F	5.52
AKDHHTDLTTL	5.40
VRGLLLLKTRL	5.75
RRVRKLPSTTL	5.10
RGMKRLTSTRL	5.94
RGIKKITSTAL	5.50
LSRRRSAQMRV	5.01
SAAGPGFSLKF	4.99
FGQGTKVQIKR	5.09
FGQGTRLEIKR	5.92
LSMDHVCLGH-	5.12
FKSREDCCTKF	5.95
LTVTGKTVRLL	5.28
SLQASSEKTQQ	5.11
REVVDSTTSSL	5.13
REVLDSTTSSL	5.25
SVKVEAEASRQ	5.15
NNRSSGCCSGC	5.06
GRRLTTSTSTF	5.31
FIMLTRLVLNS	5.60
LHSCKTSVGKE	5.00
VVLTSVTTVLP	5.45
DSSDLVEDSFL	5.21
IAKADAQESRL	5.77
DSESQEENTQL	5.72
ENLIEFIRSRL	5.11
KTMVTRFNEAQ	5.01
SDEDMGFGLFD	5.26
TERRSKSCTIL	5.33
DNITLLQSVSN	5.50
QHVTEAFQFHF	5.04
SSTCILQETSL	5.81
MGRGNIFQKRR	5.38
DRN-VPGKVRQ	5.16
H-KTHLVTKNL	5.51
PGNIPPPPPTN	5.13
EKSSTMRIS-L	5.26
KRIPLTRTTTF	5.42
AANGLMQTSKL	5.61
FAPSRKLNTEI	5.29
GLA-LEETKPL	5.11

Human NHERF1 PDZ (2/2) [80]

Peptide	LogBLU
QAEEDWTCTIL	5.10
EQELLDFTNWF	5.14
GDFREDDDTAL	5.47
GSPLHSLETSL	5.63
GSPIHSLETSL	5.43
SSCNLAKETLL	4.94
ETLHSLQTAFT	5.11
FKLLDQMETPL	5.35
LADEMICITTL	5.06
ESENATSLTTF	5.04
TSSCDKSDTCF	5.16
ETEEEVQDTRL	5.65
LCLDTSRETDL	5.24
VEAETTTTFSP	5.19
GPQAQAENTAF	5.41
NSRPHTNETSL	5.22
SNQLAWFDTDL	5.22
IGTMFLRETSL	4.93
DTISFTQTSQF	5.16
MENGSLDTFLR	5.03
FREKKFFCTIL	5.19
AWLQEKLSFFR	5.02
NIKRKLDT-LQ	5.07
SDSEEELPTRL	5.35
NIAVAG-STRL	5.18
HSEASGMVTQL	5.59
LNTTDKEST-F	5.62
RIQEAPKLTHL	5.37
EQQRAEDITRL	5.78
RLPEAPKLTHL	5.10
TSSSVNVSSNL	5.05
GSSSGHTSTTL	5.57
GVEISCWSVEL	4.96
RRVRKLPSTTL	4.95
RGMKRLTSTRL	5.23
RGIKKITSTAL	5.44
SQKVAV-STCL	5.01
LRNGLVKDKRF	4.99
FKSREDCCTKF	5.00
ETHREVKFTSL	5.20
REVVDSTTSSL	5.07
LGARVSKETPL	5.44
FGCAEPANTFL	5.57
AGIENVNSTKF	4.99
PSTLTIFETAL	5.38
GRRLTTSTSTF	5.26
DWAKEKQHTRL	5.66
ALPAHHNATRL	5.17
EWAKERKLTRL	5.27
NALEEPKGTRL	5.79
GLIAGEKETHL	4.94
IAAITEKETHF	4.94
ERPTILKSTAF	5.18
HHHHTLPGSVQ	5.34
DMIQLMNSTHL	5.19
AGSENTKDIRL	5.00
PEFKQNGDTSL	5.53
APRAEAEDSFL	5.07
DSSDLVEDSFL	5.02
DIPGKEFDTPL	5.33
IAKADAQESRL	5.26
DSESQEENTQL	5.44
VKKPGKKCTVF	5.15
LERGLESATSL	5.17
TERRSKSCTIL	5.12
DGGRDQQETNL	5.52
SSTCILQETSL	5.53
NGTCTSRITTL	5.38
KRIPLTRTTTF	5.25
KINKTEICSQL	5.37
PLLEACEFLRK	5.11
QNQELPSCSSR	5.65
MEGAWSMSASH	4.92
NLGHTPFVDEL	5.11
LRWVRKTPW-Q	5.04
VREIGTVT-LM	4.92
SRLERIESTEI	5.33
LT-HQRIHNVT	5.13
KEGSRS-CTDS	5.30
TGEKP-SCKVC	5.46

Human NHERF2 PDZ (1/2) [80]

Peptide	LogBLU
QAEEDWTCTIL	5.35
AQWVDS--TSL	4.78
GDFREDDDTAL	4.81
GSPLHSLETSL	5.11
GSPIHSLETSL	5.01
SSCNLAKETLL	4.87
GRNCSTNDSLL	4.92
GPHSFVTSS-L	5.04
LQDSRV-VSSL	5.01
LADEMICITTL	4.93
ETEEEVQDTRL	5.83
GPQAQAENTAF	4.94
NSRPHTNETSL	4.97
IGTMFLRETSL	4.76
NKAKHDELT-F	4.92
MEAQGCSCTLL	5.50
WGMGSQSCTLL	5.31
GGAPVSAGTIF	4.85
IGKIGTVMTFL	5.48
DTISFTQTSQF	5.02
MENGSLDTFLR	4.85
LQDWASVGAFL	4.84
FREKKFFCALL	4.89
FREKKFFCTIL	5.44
PFRPQKVCSFL	4.94
KSKSCHDLSVL	4.90
FFNVI-WSI-L	4.93
SDSEEELPTRL	5.14
NIAVAG-STRL	5.37
HSEASGMVTQL	5.03
LNTTDKEST-F	5.26
EQQRAEDITRL	5.39
RGMKRLTSTRL	5.29
RGIKKITSTAL	4.88
FGGGTQLTVLR	4.85
FKSREDCCTKF	5.52
NFTSLTWISTL	4.90
FGCAEPANTFL	5.23
AGIENVNSTKF	5.25
PSTLTIFETAL	5.65
WCIQFNRGSRL	4.83
DWAKEKQHTRL	5.39
ALPAHHNATRL	5.45
EWAKERKLTRL	5.29
NALEEPKGTRL	5.67
VLKDDELKTKL	4.80
ERPTILKSTAF	4.76
DMIQLMNSTHL	4.77
DPEIVVQATVL	5.07
APRAEAEDSFL	5.24
DSSDLVEDSFL	5.31
NWIKRRPQTKL	5.00
DIPGKEFDTPL	5.42
IAKADAQESRL	5.36
DSESQEENTQL	5.15
ENLIEFIRSRL	5.07
VKKPGKKCTVF	4.80
GGACGG-CSVL	4.83
RFTTKRPNTFF	5.14
VWRSKRTDTLL	4.98
TERRSKSCTIL	4.81
SSTCILQETSL	4.95
RPRRKTKTLL	5.00
QRDR-SHWTKL	5.02
NGTCTSRITTL	5.00
EKSSTMRIS-L	5.17
KRIPLTRTTTF	5.28
KINKTEICSQL	4.98
LDQTLLELNNM	5.32
SMEPNQEETNR	5.58
IVSM-L-CNLQ	4.83
LFGSGTTLVVT	5.24
RDRTVQSPQSK	4.91
GTMF-WSRIE-	4.94
SEF-PRDSAKH	4.77
QPLRKDKDKKN	5.50
TDDIPLLKEA	5.34
SAPEAEQGGAE	5.13
FSLWSHEQTHN	5.34
IMRHHKEVGLP	4.79

Human NHERF2 PDZ (2/2) [80]

Peptide	LogBLU
QAEEDWTCTIL	5.45
PFIAIHAESKL	5.45
GDFREDDDTAL	5.66
GSPLHSLETSL	5.72
GSPIHSLETSL	5.67
SSCNLAKETLL	5.34
GRNCSTNDSLL	5.46
EMGAIKNLTSF	5.47
FKLLDQMETPL	5.76
LADEMICITTL	5.32
ESENATSLTTF	5.71
ARQHVVADTEL	5.58
TSSCDKSDTCF	5.44
ETEEEVQDTRL	5.82
LCLDTSRETDL	5.83
STMDHDLHDAL	5.52
VEAETTTTFSP	5.45
KINLSQKETSI	5.37
GPQAQAENTAF	5.29
NSRPHTNETSL	5.56
SNQLAWFDTDL	5.89
PFIFHEKTSEF	5.47
IGTMFLRETSL	5.50
DKNKDDELTGF	5.74
IGKIGTVMTFL	5.32
DTISFTQTSQF	5.51
DSAQGSESHSL	5.30
KSTKIPESEDL	5.35
RIQEAPKLTHL	5.40
EQQRAEDITRL	5.31
RLPEAPKLTHL	5.60
AKDHHTDLTTL	5.60
GGKGGLCQSSL	5.38
TSSSVNVSSNL	5.72
GSSSGHTSTTL	5.90
SANVPTEGFQF	5.41
RQLAEIAESGL	5.38
SQKVAV-STCL	5.37
FKSREDCCTKF	5.80
ETHREVKFTSL	5.43
REVVDSTTSSL	5.47
REVLDSTTSSL	5.57
LGARVSKETPL	5.68
FGCAEPANTFL	5.73
AGIENVNSTKF	5.80
PSTLTIFETAL	5.63
SQMMPHVQTHF	5.47
DWAKEKQHTRL	5.30
ALPAHHNATRL	5.39
VQPSSLEMSAL	5.29
GLIAGEKETHL	5.59
ERPTILKSTAF	5.34
DMIQLMNSTHL	5.48
NGGELMLESTF	5.54
PEFKQNGDTSL	5.68
CSNSTNMESSL	5.69
APRAEAEDSFL	5.59
TVLLKERSTEL	5.52
DIPGKEFDTPL	5.56
IAKADAQESRL	5.62
DSESQEENTQL	5.75
QANLSLEALFQ	5.31
LERGLESATSL	5.66
KLVPVHLDSIL	5.40
TERRSKSCTIL	5.35
APELESKESVL	5.49
DGGRDQQETNL	5.61
SSTCILQETSL	5.75
AANGLMQTSKL	5.80
KINKTEICSQL	5.39
QLKFFEENMNF	5.45
LAGAPMPLTHL	5.40
EPLPEKTQESL	5.28
DGQEEQVTTRL	5.83
HFGDGTRLSIL	5.35
RLQDLAPDTAL	5.71
LNRFQQDSQKF	5.51
MNDPAWDETNL	5.62
SEEELFSRTHL	5.31
NMPNKQPESSL	5.47

Human SYNA1 PDZ (1/1) [56]

Peptide	LogBLU
GDKTEEQVS-V	1.92
SSVVSERISSV	2.50
AD-SNLRETDI	1.92
ILCRGDRKRIV	1.32
SGKSIRTVVKI	1.36
SSLREMETFVS	2.56
VPKHGDRFFTI	1.65
GSPIHSLETSL	2.45
SSCNLAKETLL	2.52
CRPGFASESKV	2.45
EPPDGDFQTEV	2.39
DVQEGDFQTEV	2.41
NVISERNSTTV	2.40
DL-SGLNQRRI	1.48
CSNAKAVETDV	2.17
LCLDTSRETDL	2.03
TVRPGVKESLV	2.29
KINLSQKETSI	2.34
SLNGGSRETGL	2.38
IGTMFLRETSL	2.56
TIQISELETNV	2.43
LHLVLQETISP	2.46
DENDEVKITVI	2.38
ALSKENLESMV	2.69
HGQNMTVSVVI	2.38
IEPAKETTTNV	2.52
GKQVVPFSSSV	2.41
FDVFGQGTLVT	2.36
SSKVPAVTSLV	2.40
QRRQRKSRRTI	1.20
KEKMRGMRGKI	1.23
EPRPLRRESEI	2.29
DNIS-RRESAI	2.29
GSALSVRISNV	2.49
LRTLLLQQSNV	2.61
KFNKS-NGTVD	1.74
VDPGARMTALQ	2.64
FGGGTRVTVLG	2.11
FGTGTKVTVLR	2.40
FGGGTRVTVLS	2.36
TLDSQIQETSI	2.11
NCNTVTVETVV	2.37
VNASRTRITFV	2.24
QSVTSERETNI	2.43
AQCVTPVQTVV	2.64
PRNGRRSAGFI	1.53
RQFTLRHWLKV	1.34
ETPEEKQTTIA	2.38
ELAL-LRRSDV	1.77
QLNGTFESQVQ	2.42
QSQGTCRTSII	2.17
NGTCTSRITTL	2.41
RTACCHSETVV	2.32
FGTGTRLQVTL	2.52
TFGSGTRLTVV	2.72
ILQTSRVKMGD	2.50

Human PSD95 PDZ (1/1) [6068]

Peptide	LogBLU
FREKKFFCTIL	5.39
NSRPHTNETSL	5.37
SSTCILQETSL	5.33
DGQEEQVTTRL	5.31
VACRLAQLTRV	5.28
NWIKRRPQTKL	5.27
LRLCIPSTGPL	5.21
EQQRAEDITRL	5.19
SFHSIKQSTAV	5.18
GSSSGHTSTTL	5.18
HHHHTLPGSVQ	5.17
QAEEDWTCTIL	5.16
SYRRPPSATCV	5.14
DWAKEKQHTRL	5.11
PFRPQKVCSFL	5.11
LADEMICITTL	5.09
EQSDVRFSSAV	5.09
PWSPRLESSKI	5.09
CGKGFSYSSVL	5.07
SVKYRLKFRFG	5.07
FRTSLPKSCDL	5.07
SNVKIVKVKKE	5.06
NNPANRRTTPV	5.03
TIKVIKAKKKT	5.03
RRVRKLPSTTL	5.03
LQDSRVYVSSL	5.03
TERRSKSCTIL	5.02
ETEEEVQDTRL	5.01
RVAFKLRINKT	5.01
KELKTVTFSKL	5.01
WGMGSQSCTLL	5.01
LRTLLLQQSNV	5.00
QVNKVWDQSSV	5.00
FGVEIGPVCFV	5.00
ALPAHHNATRL	5.00
NVISERNSTTV	4.99
QISEEYMQTVL	4.99
EWAKERKLTRL	4.99
SSIKVVLRKEV	4.99
RPRDHVKKTIL	4.98
LFLVLASRTQL	4.97
SRLERIESTEI	4.95
DMMLEMLCTKI	4.94
KINLSQKETSI	4.93
IHFTRRAITDL	4.93
QSLAGSPSSKL	4.93
SNQLAWFDTDL	4.92
KGGALQSCTII	4.92
TENFPLKLCRI	4.92
KRNTLYFSTDV	4.90
IAKADAQESRL	4.90
TLDSQIQETSI	4.90
ARLVKGERTNL	4.90
RGMKRLTSTRL	4.90
DGYDWGPATDL	4.89
FVPWIKSVTKL	4.89
ENLIEFIRSRL	4.89
QKVKEKLKIDS	4.89
GGKGGLCQSSL	4.88
RGIKKITSTAL	4.88
AANGLMQTSKL	4.88
RHSRSSLNTVV	4.87
GSPIHSLETSL	4.87
RELLKELRTQV	4.87
WCIQFNRGSRL	4.87
HSEASGMVTQL	4.87
KRQSILFSTEV	4.86
FIPTLFCLCRL	4.86
FGFDVGPVCFL	4.85
RLPEAPKLTHL	4.85
GPGWLLSWCSL	4.85
NFIFDEFRTVL	4.85
RVVTRPCQCQV	4.84
DEDQHTQITKV	4.84
FGVDVGPVCFL	4.84
IGTMFLRETSL	4.83
QSFLQTETSVI	4.83
SQKVAVYSTCL	4.82
IHQNPGEFCVL	4.82
AKRIRERCCIL	4.80
NFTSLTWISTL	4.80
WEIPVQIVSHL	4.80
SDSEEELPTRL	4.79
SSKVPAVTSLV	4.79
KILLKICGGND	4.79
RHSGSYLVTSV	4.78
FGVDIGPVCFL	4.78
TPIGKPATAKL	4.43
IIKGTVRTFLS	4.43
GSGGFPTGRLA	4.43
PHRFQKTCSPI	4.42
KLDKESFLTWL	4.42
DGDFKIKVRGL	4.42
DLYSGLNQRRI	4.42
NRVGFAEAARL	4.42
VQSFRHLRRVK	4.42
WGQGTLVTVSL	4.42
LGRVYQSCWQV	4.42
LAKKAQAQPVM	4.41
FYELASPTSPI	4.41
NLKMKIQVLLH	4.41
SINKIRIFKGR	4.41
FGGGTTLTVLS	4.41
SEEELFSRTHL	4.41
GKVEFKLKIEK	4.41
NQPGTPTRTAV	4.41
GMGGGMGGGMF	4.41
QKLGRAMREIV	4.41
LGEKRACCISL	4.41
SVDKVTSPTKV	4.41
APPPQVEKKGQ	4.41
VIGHEDLETAV	4.41
TVTVKVPQKNS	4.41
PEWQSCFRRDL	4.41
ELLKIERRTEL	4.41
CCVPICCQCKI	4.41
RSLSNINSEML	4.41
LEQVVASYCNL	4.41
FGTGTKVTVLG	4.41
EQPVYTQLTRP	4.41
GSALSVRISNV	4.41
LLVSLYLHDNT	4.40
ADYSNLRETDI	4.40
LMSNDSKALCG	4.40
ALRTPIPKSNL	4.40
EQETEAAEKSA	4.40
TVTIKVPQKNS	4.40
PARGKLDEAEV	4.40
ETKSFFTSQQL	4.40
DCHQLADSFRE	4.40
WRRISSLESEV	4.40
FGGGTKVTVLG	4.40
DSSLQSLETSV	4.40
KFSGLTSKLAT	4.40

EFLLRFSQDNA	4.40
PPTYQIRFLPR	4.40
ELTKLNVSKSV	4.40
IATLTSEEREL	4.40
SAFKQCLLKKC	4.40
SDGVNEKVSCV	4.40
REVVDSTTSSL	4.40
FLRNLVPRTES	4.40
ARFWYRRSTTD	4.40
ERDSRESWRAA	4.40
REVLDSTTSSL	4.40
CCRNQSFCNKI	4.40
PQSPANIYYKV	4.40
LCVNVACTGCL	4.40
VGSLRRVTDLY	4.40
EQERSFRITFD	4.39
TNLKKGLRRKD	4.39
FLRIMKKTSLY	4.39
TRCLRMRPPRS	4.39
TEKPIDSETKM	4.39
GRLPRTYRLFR	4.39
PAVQSFRRIVE	4.39
HYKTHLVTKNL	4.39
LEMTRKRVLHA	4.39
IAVAATPIPKL	4.39
VSFTYCCTRVD	4.39
LCTEDPCSRLI	4.39
MEMRQLQRGPA	4.39
ATLFVKISLQD	4.39
IVRVLTARKTV	4.39
VLAKVKNSATP	4.39
SPCIAVQVVRI	4.39
ESSPIENDSSP	4.39
CCVPVCYQCKI	4.39
RSMKKRTSTGL	4.39
RSKGPLNGCHI	4.39
RGVPLSVNPVC	4.39
ENLEAETAPLP	4.39
FAWTIKLTGVK	4.39
AQCVTPVQTVV	4.39
EPPDGDFQTEV	4.39
PLNIIEQESSA	4.37
LTKFNVSYLKR	4.37
SVALKFKPRKH	4.37
SKPIHTTILNP	4.37
VPTSYLRVTLN	4.37
RRTRVNGDNRL	4.37
SPKDYSTKYRY	4.37
PAARAAAYDRH	4.37
GQKRPLKNDEL	4.37
CLQAEAFNPPS	4.37
EQYLSMVFSIV	4.37
WLTCLLRHERI	4.37
GWRPASLRSWG	4.36
TSMNERETGML	4.36
KLEKARPGQFS	4.36
GDYLKKPGGGG	4.36
LSRYQAQHGAL	4.36
LDLSPWASVKN	4.36
NGGELMLESTF	4.36
GRNCSTNDSLL	4.36
YVGIERETDVL	4.36
LQAKVRESYQK	4.36
GSRFSCRKMIL	4.36
KAEASVACSLC	4.36
RSDRGGGRSRY	4.36
NCNTVTVETVV	4.36
DDKKSKTCLML	4.36
DQVLSVLKGEE	4.36
MEAVAAQGKAK	4.36
RTCANCQSSSP	4.36
YDTTPFQAKTQ	4.36
NSYLSWKAHRL	4.36
VCALSRKIGRT	4.36
GNSDSKQKLND	4.36
CQLLVGERSLT	4.36
LQDPPNKKPKV	4.36
YRGGYNRFAPY	4.36
AAGNPGGESVP	4.36
SGPSIVHRKCF	4.36
ALNLASWREAS	4.36
QKSKEYFSKQK	4.36
GQKEQKETTKN	4.36
PRDASKYKAKQ	4.36
FLAQPPSGQRR	4.36
SGKSIRTVLTF	4.36
KKRLLLREQAL	4.36
PQMITPLVTKG	4.36
EDQGKVKQAKK	4.36
VLRDYIYNWRS	4.36
PPIPPPPPHQQ	4.36
QCRSVEGSCGF	4.36
LPEAPWIHKLL	4.36
LVVVDSRLKKQ	4.36
AWRISRAFRRR	4.36
TQDIVFGEVDR	4.36
EAFSTPQQLEK	4.36
PSSSASKKQYV	4.36
PQSPPNIYYKV	4.36
SSRGGKCSSSK	4.36
CQAGYDSEDVI	4.36
TKRTRRPQPLT	4.36
EDYETHQLRQA	4.36
RWSRRWQESRR	4.36
FLGRVVNPTLL	4.36
KGKDRKPGNLV	4.36
HEKPVSRPQNT	4.36
ITRKRSGEAAV	4.36
PPRAKQDSAPL	4.36
ISGRLYRLCCR	4.36
RGLAERASQQS	4.36
LWLVSVKYRAF	4.36
LRDIDGMNKPV	4.36
GFEVGPVCFLG	4.36
NVDLPPKESSI	4.36
AVNSATGVPTV	4.36
FGGGTKLTVLS	4.36
QKVGAVAAATS	4.36
CSNSTNMESSL	4.36
TRPLQTVPLWD	4.36
SYGNQEFKKKE	4.36
REESDWTIVLS	4.36
FRRPLPKDQQK	4.36
TKAVTKYTSSK	4.36
FLYVSIYWWGS	4.36
SESIGAYYKGG	4.36
GQVNHTMEVSC	4.36
GDFREDDDTAL	4.36
KATRPSQGHQP	4.36
DFRKLLVALLH	4.35
KDNPNQYHYVA	4.35
QVPEAPGGSAV	4.35
WFLLLPYISLV	4.35
YIVTRPRERPM	4.35
KEAIVPMEVWT	4.35
KSGDGKNSVWI	4.35
RRPEYTPIHLS	4.35
NEIISRIKVTT	4.35

LDRHQTMEVSC	4.35
AGCSLSFRSEL	4.35
PAPAQQKTKQK	4.35
FMSGANSQKQM	4.35
SELYHPDDHVL	4.35
RQERKPKDRRK	4.35
STWINQVIAYN	4.35
PENCKPWSVRV	4.35
FKRQTSYQGVL	4.35
HGYPYSDLDFE	4.35
EKDECVIATEV	4.35
RVHGMEALGSF	4.35
DESSLVRHRWK	4.35
KQFQPYFIPIN	4.35
IVPLMRQYRHH	4.35
VSRCQVCMRRT	4.35
ESFSFSSNVLS	4.35
EPPADQWKWPI	4.35
PCPGRIRHFKV	4.35
VEKRKANFKDQ	4.35
ATQTKENESKA	4.35
IFHTIKQHALN	4.35
QLCCKTCTFQG	4.35
MAQFSVLLPRH	4.35
PPAALPESTHM	4.35
RILRQRVENGN	4.35
FIAKYILKHTS	4.35
DNISYRRESAI	4.35
VSHRLCAVPQV	4.35
QTYVTYTNHAI	4.35
CSQYETLSDSE	4.35
GEIFEKFHPIR	4.35
IHLLLLQQGDE	4.35
DDDLGGLGLGP	4.35
NISSTSEPKEE	4.35
PFKDKKTCIIL	4.35
SELKYVPKAEQ	4.35
IEKTYLYLCYV	4.35
AMLILIGSLEL	4.35
FRRPCTLSELE	4.35
GASGLDKRDYV	4.35
SGSYLVQHQRI	4.35
IMFATFTLYLT	4.35
PELGDPPAAPQ	4.35
SPPPETSCVLA	4.35
MYLERLKEEKG	4.35
IEELIRKTKGS	4.35
AAPATGNKKTH	4.35
GDKPSLSARYV	4.35
KTVEDSKLQGF	4.35
GGLSPLNSHGL	4.35
PPHHGLQTSVQ	4.35
CLPRLPIGRFT	4.35
DGLWLRVEPLG	4.35
YLFERLTLKHD	4.35
DHVARLTWGGC	4.35
FQKIVPFRKAS	4.35
CCEHFQMQLRS	4.35
CRFYNHTICAQ	4.35
QLVHDKVVQNW	4.35
LDPPHQMELTQ	4.35
PRGACTALTPD	4.35
VMAMMYTVVTP	4.35
DDLDEIEKIAN	4.35
PRTQHIQQPRK	4.35
LSSNATRETLY	4.35
DIVFKRISKRI	4.35
ALCQFSSKEIH	4.35
LAHNVSKDNRQ	4.35
DVPHGKEEQIC	4.35
LADMYGGGEED	4.35
VIVDTSDLPDP	4.35
PRTYLYVWPYK	4.35
RSRRIRPTHPA	4.35
KLPCVEEVVEK	4.35
PRIKPRQRSLA	4.35
AIIKNAYKKGE	4.35
IYKGPGSEAGP	4.35
SSRARSDDLTV	4.35
FQVQSIRRIQQ	4.35
FTVTPVIEEDE	4.35
WKYREPKDRSE	4.35
MYSGHGKLEFV	4.35
PAGNRSEQRGF	4.35
EIDIPVVSHTT	4.35
FGPEVVAPQRL	4.35
VDEYNEMPMPV	4.35
THSGYRLNDYV	4.35
KTPFVVEIADW	4.35
SKSKAPHLHST	4.35
PFIWIPSKEKL	4.35
SSTSDTLTTSH	4.35
EPAVQRTLLEK	4.35
GEEIPTQKPDQ	4.35
ERPTILKSTAF	4.35
ALMYRLYMAED	4.35
CQPCFNKLFPL	4.35
QALVDILNEEN	4.35
NLKASVVFNQL	4.35
YLAWIREHTVS	4.35
NMFTNQATVIR	4.35
MAHTVRKDYRS	4.35
LVLDCLETRQE	4.35
CGGPRPGYETL	4.35
THGSRFYFWTK	4.35
RKPRRVETLPT	4.35
LLAPTKTHVEI	4.35
SESSGLQQPPR	4.35
AFVKKILGEDS	4.35
TIGVLARVALI	4.35
KDYLAHSSMDF	4.35
TVPRALMCLSQ	4.35
PKKPGLRRRQT	4.35
QPITQNGQHPT	4.35
GRVLWRPFQRP	4.35
NNRVGLAPAVP	4.35
ILNPNKMATLM	4.35
ELKAWLLEKGF	4.35
PGSGDPSHLFL	4.35
SKKMLKRRSNL	4.35
RQFTLRHWLKV	4.35
AVFSLIRWKSS	4.35
FVERGGGDGIA	4.35
ATFSPRTIEIC	4.35
PFVPTNYYTLK	4.35
RNDLSSTSTHS	4.35
SETVRSPNSPF	4.35
HYIWVPSPEKL	4.35
EAEAEKCSCCQ	4.35
TVHRQPSHFPR	4.35
ELVLHLLRSQS	4.35
DVQEGDFQTEV	4.35
KGKNVRQRNSS	4.35
YEEFVRVLVSK	4.35
NLQVQIYNTCQ	4.35
EYNGQITGASL	4.35
SRELPIAFCQR	4.35
LKRTEMKIRRA	4.35

PARFTHFHGEI	4.35
PNAQRAFADDP	4.35
APEVITSSPLK	4.35
AVYKQSVTSSA	4.35
HVTRRTPDYFL	4.35
EGCIPRRSTAS	4.35
FGEALVRHDEF	4.35
PSDGPGGGAAA	4.35
SETAIQPSWYF	4.35
ILLIAVFLVLT	4.35
DESFPACKIEF	4.35
RDEYDEVAMPV	4.35
PYIWVPARERL	4.35
VKNKGRRIAYL	4.35
IVLIIGLSVGK	4.35
GTPSTIETTPR	4.35
LEAGDKKPDAK	4.35
GSQETLIPPRL	4.35
IAAITEKETHF	4.35
DTRPASFWETS	4.35
ATSSTSRALIL	4.35
SSRPRPDDLEI	4.35
PEEEEGFEGGD	4.35
FRTLAKTCIKK	4.35
DWLSNQLRNQK	4.35
SKLFAGCAVKS	4.35
TKHSTVQHSYS	4.35
MASLQMAGNPG	4.35
VWRRPGAARAP	4.35
PLLSEIWDVHE	4.35
EANEALVKALE	4.35
HIRHHAKYLNA	4.35
EGLAIRNASLT	4.35
VYQAYETILKN	4.35
CITLGAYLGHK	4.35
SGPGNFFFQFG	4.35
DRLVLQYAPSA	4.35
CCGFYNNTVCA	4.35
AVGIFNTVINP	4.35
LLTAQEQPVYI	4.35
PNATSGSLENN	4.35
PMNPGQLCEVW	4.35
YLRVKTVTFEY	4.35
KPSCIENSVTI	4.35
LSPPSYCKLAS	4.35
VNLLGEEKKST	4.35
LKRFFEVRRVV	4.35
SQIQSIRRIQQ	4.35
QLPCCPLQAMV	4.35
KHTLGDSDNES	4.35
DEPEGGDGAAP	4.35
QNGCINCCKVL	4.35
GDKEPGLPPPR	4.35
PVSIGPVGKPK	4.35
PLAERWKNSLT	4.35
REIAEEEESMM	4.35
HVVGAYQNQEK	4.35
YHSEPVEDEKP	4.35
ALRMRLQIQLY	4.35
LKSKELGVATN	4.35
FLSGEPSQADV	4.35
VYQKLLERMKH	4.35
ANLIKGVGGCS	4.35
TVSIRYSGHSL	4.35
MKDDDYDDQLC	4.35
IPTDNQIKARK	4.35
KGTERERLLNF	4.35
DGSYQKPSYIL	4.35
FIKTTVKELIS	4.35
KQTALGWNSPT	4.35
QNQGNNLTGSF	4.35
KEPERVVHYEI	4.35
FKKLIRCREHT	4.35
DWLANQLKNKK	4.35
GNPCEFFVDIM	4.35
GRGDDFSWEQW	4.35
GSHYIANNKKM	4.35
TLFGLSSNSKK	4.35
DGEKDTYSYLA	4.35
KAVEAEGDEGS	4.35
RNMVVRSCGCH	4.35
IQNISHNWNRK	4.35
KGASDKCSCCA	4.35
SVSTDTSAEAL	4.35
WWKMMSETLNF	4.35
NPEDSSCTSEI	4.35
TFSKEAGDEEI	4.35
LASVQWKEVDG	4.35
DVTAALAKQGL	4.35
SLQRNQVPTVN	4.35
EQPVYTTLSRP	4.35
LNELERLGQDL	4.35
GSKDDFSWQQW	4.35
KMNHQKCCSEA	4.35
KINNNDDNEKI	4.35
WDFYTEETLAE	4.35
NEIQRDSALTK	4.35
ATTMMIRPADF	4.35
KEAQMPAVIRF	4.35
YKFGRTEELWT	4.35
HDDSDEDLLHI	4.35
SCWWIRYTRTP	4.35
RPGVLLRAPFP	4.35
LESLKGGKKAK	4.35
CNTLSSPNQYQ	4.35
LTRCFYLFPGH	4.35
VLSVLMLKQAL	4.35
HYKLVPQQLAH	4.35
KAIQMASRTGK	4.35
KLADMYGGGDD	4.35
EKVLRSKVDSS	4.35
SHLAAREKANL	4.35
KITPFTPNGFH	4.35
FGGGTNLTVLG	4.35
GVGVFNTVINP	4.35
HWRLILPRPWN	4.35
RPWINQILQAN	4.35
CVPNGLSLSSD	4.35
FARRRKRSVSL	4.35
SSRMVVHESEE	4.35
PNKHLQKTNFN	4.35
QRLDGASWGVS	4.35
LAPCSSRTRTP	4.35
YMNPEKKCRVW	4.35
IIFDLPNPQGS	4.35
PQFSYSASIRE	4.35
DFESLLDHKHR	4.35
GFSHEELDDSW	4.35
SLLAVILAPSL	4.35
LVVNPNYLLED	4.35
DTHWVPVSWVR	4.35
TPSEEAASPHS	4.35
ISYVLPEHMSM	4.35
TTDLESTRREV	4.35
ALAKVSRKPSI	4.35
LESSIQGLRIM	4.35
FDPPGSDTNSP	4.35
GRFDVKIEVKS	4.35

SLAEPRVSREG	4.35
SFFRENQQITV	4.35
MVSVQAGTKRQ	4.35
GIRAHEWMFPV	4.35
LSRKNPSKECF	4.35
VRVWQVTIGTR	4.35
TLSATETTFEY	4.35
FFNGDITAGLS	4.35
HLKHERLRKYH	4.35
LERLTAAWLSA	4.35
DILQGHMVAPL	4.35
NTMDADSLVSR	4.35
DASTGPLEGCR	4.35
TKQRIDEFESM	4.35
TQITVDIGTPS	4.35
LAPKIEDEEGS	4.35
RCQEQFRILSD	4.35
GTAEYRRTGDS	4.35
QHFEVPVLRCH	4.35
SEELLAFKVHP	4.35
MQYGLTRDSPP	4.35
HATPLPVTDTS	4.35
DEDDAEINPKV	4.35
CSKGDDDPLPT	4.35
LETIILSKLSQ	4.35
SAQSLAEDDVE	4.35
KDQSGEVLSSV	4.35
PEPTVLSLLWG	4.35
TDTNFSDMFWS	4.35
DNDDSDDDDDE	4.35
ASREEPIEWEF	4.35
YPIKNLEMSQQ	4.35
RLLDKHFKRLT	4.35
EAPGIRDHESL	4.35
AGIENVNSTKF	4.35
PQKDLEIFKNF	4.35
FQTQNFDRFDN	4.35
SGADGTVRLFT	4.35
HKLQDWAGSRQ	4.35
PPGHQGPPGPD	4.35
GSTVTENVVNS	4.35
TVTAKEDEEED	4.35
SRLPLRAQINL	4.35
LCWLRIDAHLL	4.35
PITISSIPPQT	4.35
QDMVVEGCGCR	4.35
WNSDASTSEAS	4.35
TIPKEGPGPAP	4.35
APYLRQLLDQF	4.35
VASFVKLILGD	4.35
DLALILCAGDN	4.35
AVYPYCPTGRK	4.35
PWEVNKPGEVC	4.35
NPHPYNEGYVY	4.35
GEKLYLVKHLS	4.35
TLRQGDSPGSQ	4.35
GVESFDEGSSH	4.35
LRAQDMQEGAD	4.35
YREVSRAFHLN	4.35
SSFSGFLVAPM	4.35
LGILYILQTLE	4.35
DLDPGAGSLEI	4.35
RPFLKPSYAFS	4.35
NVVVSLEGKPL	4.35
FQKKGAEKEEL	4.35
YEAFVRHILSG	4.35
EKEPPPPYLPA	4.34
AERRREKCRCV	4.34
TPQQRRSLPAI	4.34
DHVYNRLARYR	4.34
VVLVAPPLRVG	4.34
HTLKSNSWLNC	4.34
RDIGTKGLNEE	4.34
EPFEPAQPDAY	4.34
FSPPSYSKLTS	4.34
QENVDYVILKH	4.34
FHHLTYLPETT	4.34
CLRVANEVTLN	4.34
LEEDQKGETSG	4.34
KDQASWRFQVL	4.34
NNAMKISFAKK	4.34
DKFSFVCKFKN	4.34
AKIKEEEAAKD	4.34
HNVEATSMFQL	4.34
SGFLLFPDMEA	4.34
EEHCTFGDPIV	4.34
EEDDVDFAMFN	4.34
GCLREWCDAFL	4.34
VDTMAQKTQAV	4.34
FPEEDYGLDEI	4.34
YGSKDTFDDDS	4.34
CTPTDVRDVDI	4.34
PEGASNESERD	4.34
CGEGEEAAETE	4.34
LQVFVLEYLHL	4.34
LGTAMNPCSRT	4.34
AEHKRTCRRKR	4.34
CPRCLFQEGAP	4.34
AMEEEKPEGHV	4.34
SSATGSFSYPE	4.34
DSNGSNSSNIQ	4.34
ITVKVSDWILP	4.34
GCTFPEKPDFH	4.34
AKMSESQDIKL	4.34
MTEEEKNIKWG	4.34
RRQKFNQQYKA	4.34
VLKLMGRGTKF	4.34
DRGFRYNWPQR	4.34
IRAEPLNSVAH	4.34
EKKPGRQAGKQ	4.34
EPILCPCFMPN	4.34
DGSTSPRSQEP	4.34
PPPPKRPRPLT	4.34
LIPASASAAGQ	4.34
RKIKTAVRRRK	4.34
EEEPEADQEHA	4.34
STTISPLLLIP	4.34
KSQSSKKPYSD	4.34
IPENSAVDEEA	4.34
NELPYICKFKV	4.34
PGVTQRPRTTE	4.34
MPPSASAGQNL	4.34
IPWIENIISDF	4.34
APPSGSAPHFL	4.34
KSQCLENSPFT	4.34
AFGRRRRDLQA	4.34
ILHLVVMNFVG	4.34
AGRKFRNPKAK	4.34
GPLGIGPLHGR	4.34
FTGFLLYHDTN	4.34
VFQMFFGVEGQ	4.34
LDIHKKMVVDV	4.34
FYVALERVSHE	4.34
NNLYIFQDKVN	4.34
AFVSTWKHLPS	4.34
SAEQSPPPYSP	4.34
DSDHSSEHAHL	4.34
MCHYKNIGATR	4.34

EGGGGPAYNGM	4.34
DDSSEGSEVLM	4.34
NTHLPETKPSP	4.34
ENHGTGNHTAQ	4.34
GKVKPIYFHTQ	4.34
LEDLKSFVKSQ	4.34
AKSRVAFFEEL	4.34
SIEPLREKDKH	4.34
SMARPAAPFPS	4.34
PLHESMALEHF	4.34
LTFEVELVDID	4.34
VALPEYHRKAV	4.34
HYRQYSAGKAA	4.34
VVMAEVDGTCY	4.34
AILFLPMSAKS	4.34
EAALREEEEGV	4.34
SPEAPPAEAAE	4.34
TRYIMVSFVDP	4.34
DRILTLTTNEI	4.34
ELFEKQKFKSQ	4.34
PNMTVESCACR	4.34
GMGAHSPGQNQ	4.34
PAPPSSGGSRT	4.34
TDEDGWTTVRR	4.34
YEAFLKHILSV	4.34
GDPALPTHGDL	4.34
APGPGPPPAAR	4.34
DVTKPVPHLRL	4.34
PSKQFDKNAYV	4.34
KLIYDSWSSGK	4.34
VLSQPHLPFFR	4.34
SDSEDSETGPA	4.34
CGGTAEEILDV	4.34
FLSKFYVGHDP	4.34
LVAASQAALGL	4.34
NTEDDDDDDDD	4.34
YEELVRMVLNG	4.34
KANGGFQPDEK	4.34
GKIPCRSIRIS	4.34
GAFAYEGASFY	4.34
ATAGTRKQAHP	4.34
MVTNSSTENEA	4.34
VKTTTHSWVSG	4.34
CTKCFSTPKGR	4.34
KAKKMFLKPQD	4.34
RAPQSGLEGVF	4.34
KGPGFGVQAGL	4.34
LELGSMVNGVS	4.34
TSYNMDRFINK	4.34
GHAMIVEAYPK	4.34
LAFFAIIIAYF	4.34
KALLLLCGEDD	4.34
KWHKRGRFPSS	4.34
PGGTGGPAPQY	4.34
KKFSFVCKFKN	4.34
EAALLEEEEGV	4.34
PFPFKWWNMVP	4.34
EKLNPSMLRCE	4.34
KPEKYELSVIM	4.34
EENVLQSLYPC	4.34
LEIVLCSEPPM	4.34
VNKQGDSILCR	4.34
TSHLAVCEFPI	4.34
TAQLSLEHRDS	4.34
RRRGTLQSYLD	4.34
RNLQERLRRKE	4.34
NANAYFQSLIK	4.34
LGGLCDLSSRY	4.34
ALWTAAQALTL	4.34
EAGATVESTAC	4.34
LLKNQEFKGVS	4.34
HVRTERTPQRL	4.34
SVEQTPRKRLR	4.34
TQDDRQVELPV	4.34
RLKMLGQTRPH	4.34
GCYNTQALEQQ	4.34
QIRSNMCWLRD	4.34
GRWELMRIKVE	4.34
RILCSIQGFKD	4.34
LNTIKKEKKMT	4.34
KQIIAVLAQAF	4.34
ANPWIKFKSVS	4.34
RTPDGNLDQCK	4.34
HPLALASGTLY	4.34
NDWINSVIANN	4.34
DQGEKYIDLRH	4.34
QVIPLQRDDED	4.34
DTHLPTHSGSL	4.34
APLPALAPSSS	4.34
REEVLLTLTPC	4.34
VLYISTQKRRM	4.34
SQMMPHVQTHF	4.34
GKKDKKEEDKK	4.34
REGRGASCCRP	4.34
DSARPHFMSQF	4.34
RISVKKKQEQP	4.34
SYCACRSKTIK	4.34
FGSIKSDWLGC	4.34
AGRNFRNPLAK	4.34
EPQLKAPTPHQ	4.34
SSSSTTVKTSY	4.34
KKAERQKFSPY	4.34
PSTSTSNNSLP	4.34
FKSREDCCTKF	4.34
STLMWSDHLER	4.34
EGHQFLCSIEA	4.34
KQKRMKYSAVH	4.34
EFDQQQAESWS	4.34
CPQLLEPHRST	4.34
RVLEKLGVTVR	4.34
PLAGNPVSPTS	4.34
ISRCQVCMKRT	4.34
MPEDVLAERAL	4.34
VTYDILQCPED	4.34
SGHHLLPQEVF	4.34
LQSYLETSGTS	4.34
LSDRVIRAAFQ	4.34
FLPAAGTADCR	4.34
EQRIRMTVPCL	4.34
DGARSIHRKTF	4.34
MRPVSEMALLY	4.34
LQKRIAYLSGY	4.34
LESEREARRLR	4.34
MGAEKVGLVLQ	4.34
PEVRPTSAVAA	4.34
LQKRIEYLSSY	4.34
QISGVQQIQFS	4.34
ALYNASQPKNK	4.34
GDIHLLEVRSW	4.34
HELDSASSEVN	4.34
RHLCELLAQQF	4.34
MCASLITTLAR	4.34
SFGPEDLQKTQ	4.34
SLIENKMLKWA	4.34
WLVSGSTHRLL	4.34
IDWIEETIASH	4.34
LIDRMVNLHQA	4.34
RCCRAFLEKAH	4.34

CKEVVSRGDDY	4.34
TTTVAVTPPTA	4.34
IPCTAHWHFGQ	4.34
SDVTANTLLAS	4.34
TYYKRPVQEWV	4.34
AKEVAPVPKIH	4.34
KASCNCSNSIY	4.34
YLDQKSPTPKP	4.34
LLKHPVFRTTW	4.34
EEVLGEEQEGV	4.34
IKEEFTEAEIH	4.34
LRHPYGYTGPR	4.34
EVNNFPPQSWQ	4.34
ASWTPVSPPSM	4.34
VLLLECKRSRK	4.34
LEERRSQQGPP	4.34
SSVQGFGGWRS	4.34
MNTSGHDFEEL	4.34
NSDKKKKAKRD	4.34
QSMDVAAFNKI	4.34
CLRRLPFICSY	4.34
LVAKLKEIGRI	4.34
ERLMTPEKQSS	4.34
VAEFVCKYKLL	4.34
EKVLVEGGPAP	4.34
TIVKSKTDARS	4.34
IKQVFGDQGGH	4.34
SPQSLPRGTKA	4.34
MNEKDKKKEKK	4.34
SLLPALLRFGP	4.34
FPWEVPKKLKY	4.34
DHSPYTFQPNK	4.34
KMVGDYDRQKT	4.34
FIGKILDPRGP	4.34
PDLPPFSYHGF	4.34
TSCNSFLLKCL	4.34
GGKPVNKSKTT	4.34
YLVEWEGATAS	4.34
PVHAQMPSLCR	4.34
NSHQGTEDSTD	4.34
RPNLTLQNHAF	4.34
MSLVNSRCQEA	4.34
EEAKELVGRAS	4.34
TKSAQKAQKAK	4.34
DRQIIFWKLQY	4.34
ILKHGAKDKHD	4.34
YRHVADGEDHA	4.34
QDLQHPPSHGT	4.34
EMPKQRDPKSN	4.34
KDSRRRSGMKL	4.34
SGLPPSESGPW	4.34
DGPFKPDHYRY	4.34
RKMLRQQKKKR	4.34
LKRKQPVRKRQ	4.34
ATLRKDYLVEP	4.34
GKAFNQGKIFK	4.34
CIENSFMTASK	4.34
LEKHGYKMETS	4.34
LAEERRQKKET	4.34
CEERPRGSSSN	4.34
EGGEAPQEPQS	4.34
CTSWYCKVKMS	4.34
PRKGKGKSSKK	4.34
SFIIGRHKVRT	4.34
QITEDTVEFGS	4.34
KYKFEVYEKND	4.34
AQELEEDRAEA	4.34
VHECRRLSTPR	4.34
NTLSYFQSLIK	4.34
AANGAGPLQNW	4.34
DDFADDISLLK	4.34
KQKRMKYSAGI	4.34
SMKIRPFFPQQ	4.34
EGLLLRFPYAA	4.34
DAKPQPVDSWV	4.34
ITAGGIGDAKE	4.34
PEASPSSPPAP	4.34
DIADGCIYDND	4.34
LDYTLREVDTV	4.34
NFVEAVSKDFA	4.34
PKFEVIEKPQA	4.34
KQLNAITGSAF	4.34
NPAVAAASANL	4.34
QKPDKQEEFYA	4.34
KENCTGVQVAE	4.34
MTLSKSTCQDA	4.34
YVGIEREMEIP	4.34
SLWAVIFVLYQ	4.34
SPTEGKRKIII	4.34
AGVDGGCGGRH	4.34
ESLKKKLGLTQ	4.34
ALALYPLSVLF	4.34
FMGRVANPCVK	4.34
STLSYQEPLRT	4.34
DRRLPGKETMA	4.34
SNPLLPDQKVC	4.34
EDSIRSHFSVA	4.34
TIMEHTLNHPY	4.34
GTEKLTNKQIG	4.34
HVDFPVILYEV	4.34
LRNGLVKDKRF	4.34
KLVTLSNYLNK	4.34
VKKPGKKCTVF	4.34
LDQREEVPLTR	4.34
KDTMEKSESLM	4.34
DSDDEKMPDLE	4.34
DAGTKMSGEKI	4.34
GLGCNSFRYRR	4.34
NFDEASKNEAN	4.34
GPSEYPTKNYV	4.34
AGKVLPALLPS	4.34
TGRESPDHLDQ	4.34
LDWLSNELSKR	4.34
GTEIRSYGNDP	4.34
KSAQKAQKAGK	4.34
ATGDYDKKNYV	4.34
LAADVLRAERE	4.34
DKAFTPFSGPK	4.34
WTNAKECFSKM	4.34
GGVVTSNPLGF	4.34
YLPSAAAGKEA	4.34
NKAYKRLFLSG	4.34
RHKETLKSKTQ	4.34
DALHMQALPPR	4.34
EELGIPPDDED	4.34
ELLPSVSSVLT	4.34
VHIVQMFINTS	4.34
MATYKEKKASV	4.34
EGIWKLKPEYC	4.34
YEAFVKHIMSS	4.34
HERLGKKKEEL	4.34
PALPTAGNCTS	4.34
IMENKMCDLTV	4.34
DGQQPLVNGVP	4.34
LMKDLNAITTA	4.34
KLDYSDFQIVK	4.34
FVELGTQPATQ	4.34
PGAESGPIDST	4.34

SQLLKHNCVQM	4.34
LQSPITTSPSC	4.34
SLALMLLWLLS	4.34
FETKLGMNSPF	4.34
SVFASFVRADK	4.34
KEKLEFILAAH	4.34
LPIRNKTLSGL	4.34
SLPDPSKVQPQ	4.34
CTLATTPSPSP	4.34
VFLHSCPGTES	4.34
VSQPRGPNEKR	4.34
GVHFIFNKENF	4.34
GQTLLAVAKSQ	4.34
LFLPPFLAGMI	4.34
HTFEEPVYMKS	4.34
PHDKTKCFKFI	4.34
LVIEKDLPHYF	4.34
VRAQAKKFAPS	4.34
QCLRCQEPPQA	4.34
RTPKKTKMDPP	4.34
SSIRRLSTRRR	4.34
MHSILSNTSAE	4.34
VTEDYVQTGEH	4.34
ILFLPLPVSSD	4.34
EDSGSDPEDTY	4.34
CIRKRRLVAPC	4.34
LKKLFREGRFN	4.34
KQNPSGFGTQA	4.34
SVLSKARSWTF	4.34
RLYVGPEVDLW	4.34
EWLVETLKSRQ	4.34
HQDTMQRPQMM	4.34
LLFLLAMLLTL	4.34
FLVSLLHIFFK	4.34
TLTHYKSAVKA	4.34
NVYQVDSLQST	4.34
TVPLCGAHGSH	4.34
YTSCAQNWIYE	4.34
IQQEKECVQTS	4.34
EQVQMLAPLES	4.34
SNILDVMDEES	4.34
DENSGEFGIIL	4.34
EGTGDLDEFDF	4.34
RAQKRSVGSDE	4.34
ERKEREEWAKK	4.34
LQMNQTLPVQV	4.34
ENRAGNKREDL	4.34
LNNSTQTYGLQ	4.34
AVLSSGLTAAR	4.34
LHTTPCFIPYH	4.34
NVFYWAYYMYF	4.34
SSKELPSDFQL	4.34
QNVTSLLGCTH	4.34
SGDVWDIDNEF	4.34
RRSAEEYEYPS	4.34
NNSTHNLKDYV	4.34
IAFFLMTFLAS	4.34
KRAGWQGLCDR	4.34
ELIAREIVFDK	4.34
LQEYRKKHREE	4.34
EKPAVTAAPKK	4.34
TEPQYQPGENL	4.34
NGPFKPNYYRY	4.34
GEVFTKPQLWP	4.34
PFIFHEKTSEF	4.34
HTKSSGEMEVD	4.34
REPEDEGEDDD	4.34
DQFHVLDEVRR	4.34
AAYDISEDEED	4.34
IIYFRKPCSGD	4.34
LEWMMSRLKIR	4.34
TPQVAASGGQS	4.34
RGGYGKLAQNQ	4.34
VHIITHGEEKD	4.34
HQSKEENLISS	4.34
GHTRPFCPQNR	4.34
SDLNTQRPYYK	4.34
AEAAALRVYFQ	4.34
FLDFDFVAGGC	4.34
FFVDIGPVCFK	4.34
TSSCDKSDTCF	4.34
SKEGSQNPQSQ	4.34
EDMVVDECGCR	4.34
ILALKQRISQM	4.34
GIASAASYPTV	4.34
NLFPLNTLEST	4.34
ASVLSPSLISQ	4.34
EAQGEAAKSES	4.34
LLKKIEHARTC	4.34
AGADALAKVFV	4.34
KGMLTSTTEDE	4.34
QFDNFLVEATR	4.34
REEKPTSAPSS	4.34
DEAEAGEGGEN	4.34
VVLLGSLFSRK	4.34
KGFGRTPRPRW	4.34
CVANPENLHWL	4.34
AAAAQAKWPAQ	4.34
RLSALLTGICA	4.34
EAGITMSPGPQ	4.34
DAETKPKSPCT	4.34
EMGEMHRELNA	4.34
NEASRELESYC	4.34
DPQSSSQVTTS	4.34
WNLTIKKDWKD	4.34
NQGSSSNPLIH	4.34
HKLEQLRNSCA	4.34
RDVKNSEGDCA	4.34
NTIAKHIDAEY	4.34
FLKVYEKHSAQ	4.34
KLTVGQCDGAT	4.34
QAAKAGAPGKA	4.34
RHELASRGKNV	4.34
LNRFQQDSQKF	4.34
SIGKDGEKQDL	4.34
CTHTRVLHECL	4.34
DTDSTSTDDMW	4.34
SRSASNRLKAS	4.34
KVEELKKKYGI	4.34
LISKTRAALGV	4.34
HPRGGVQCQTS	4.34
KNRYICKQQLI	4.34
GQHGLEEDFML	4.34
EVNNLPVESWQ	4.34
FSYAPPTDSFL	4.34
YETLKHEKPPQ	4.34
GVQFGAGIRFL	4.34
GVYSRPIMNSS	4.34
PLLQEIYRDMY	4.34
EEFLTASQEAL	4.34
HKPIDYLNPPK	4.34
LGDEMACVHAL	4.34
AESRDETVSGA	4.34
GANPNPIYQVL	4.34
PRGMITKQAKK	4.34
GPIIPKCRSGV	4.34
LEEYTKKLNTQ	4.34
TTSLELDSRRH	4.34

GIASFPSYPEI	4.34
GVEAKRIFKKD	4.34
RRNTLQLHRYR	4.34
PNSSADKSGTM	4.34
YDDFLQGTMAI	4.34
TEEIIPLGNQE	4.34
TSDGAFELAPT	4.34
AERKQQKEQLE	4.34
SVTVVAPELSA	4.34
GHPLYPFNDPY	4.34
ERSINLQFLDR	4.34
PVVLSPGPQKP	4.34
DEDASRMEEVD	4.34
CSNAKAVETDV	4.34
VRMKIRPLVTQ	4.34
HLSKMFKEENE	4.34
SLQNEEKEENK	4.34
MEKRYGGFMRF	4.34
TVPRRGPDEGS	4.34
KLEDIRTRYAF	4.34
SGIGKKFGLLK	4.34
ISRCQVCMKNL	4.34
NSPELNPRLFK	4.34
EFIHQFLCNPL	4.34
PFSFKWWNMVP	4.34
KDETSAEPATD	4.34
QILIENEDVLF	4.34
QNQELPSCSSR	4.34
VDRVAEQDTQA	4.34
KLEENNHKTEA	4.34
AQNASPLVLPP	4.34
ENSPLLSGQQV	4.34
PEPLLASSGHC	4.34
LARECTIEATA	4.34
TPRNSDRCLEH	4.34
QDIQIMKDGKC	4.34
VRSAIQLGNYK	4.34
SENKIPATQKS	4.34
FMGRVANPSRS	4.34
RGLRRDGIIPT	4.34
SKVAGLSGCHF	4.34
LPGPLQDMLKK	4.34
RRDPPESNYIW	4.34
PKNPATKQQKQ	4.34
SDHYPVEVMLK	4.34
CLPPPESESKV	4.34
LGLTDPNGGLA	4.34
ANSPGQKQQGQ	4.34
GRALSNRQHAS	4.34
GLGPGGPGIFF	4.34
APKAASAPTDL	4.34
DLSPLWTLAKP	4.34
IRMRDAVLFEK	4.34
KNKRRRGCPIL	4.34
TIEQERQAGES	4.34
VSSQKVWLPAT	4.34
TKMGRVSKKAE	4.34
CVKKIYELYAV	4.34
IVLLLQCDIQK	4.34
QFLNAEDLCSA	4.34
LLPEGPPAIAN	4.34
YVAQAGLELLA	4.34
PLLQEIYKDLY	4.34
PSYCSFGEMKE	4.34
SQQFQPVVLAN	4.34
LRDCPDPCIGW	4.34
QVQLKMLPPAQ	4.34
FPANYVAPMTR	4.34
ISFQKQPEDRQ	4.34
VKAPLEVAQEH	4.34
NKPKVQCCQNI	4.34
GAEKPSWLEEQ	4.34
SHKDYPFHEEF	4.34
QRKKYRSLEKP	4.34
NLVYWVGYLYL	4.34
GQKDRETATIA	4.34
NDFFQGCPDRP	4.34
DKKATVYEVIY	4.34
YLDWIRDNMRP	4.34
PEDGPGLSPLL	4.34
LLLPLLLLQTP	4.34
DSLWEEKRGVS	4.34
LDQTLNELNCI	4.34
NETSQHHDDLE	4.34
SDPVELLVAES	4.34
TLKDLYSNKSV	4.34
SDKKKSGEKSS	4.34
VACRIREAGQR	4.34
VGESSSKGPRY	4.34
RGPRNQFITHD	4.34
SRSRSYSPRRY	4.34
QSTLRKRLYLQ	4.34
LHGLPIPASTS	4.34
HDVPVRYRIRP	4.34
CIKIAASLKGI	4.34
TYHLPPPDPFP	4.34
GVDAPSWLEEQ	4.34
SRTVLEQVLPI	4.34
KSYTPSKIRHA	4.34
AGAPSRGIIDS	4.34
PGETEFLNSSA	4.34
ILGYTRRATSW	4.34
PDMSQYWPRLQ	4.34
SGSNGFYSYFK	4.34
DDDEEEEEEEE	4.34
ELGELRKEPSL	4.34
GVSTLHGIRAW	4.34
INFVQNPNNNR	4.34
PEASLQDKEGA	4.34
GRGGAESHTFK	4.34
WGLCICQDWKD	4.34
SGSTTKNRFVV	4.34
YELLITGGTYA	4.34
GIANLASFPKM	4.34
PPESPQTRDKT	4.34
IEAYMTMKIRN	4.34
LTYHQRIHNVT	4.34
QGPGGLQGAST	4.34
SPMPVKEEFLP	4.34
YLVEWGGGGIF	4.34
LSSRGPGPATG	4.34
LQQGLLPHSSC	4.34
APGYPSYQQYH	4.34
LEGGAMSSQDG	4.34
SGFTIVSPLDI	4.34
FSSVSLQLSQD	4.34
TESMVVFGCPN	4.34
LREELQHSSLG	4.34
LLGCNIIPLQR	4.34
GVTGYFTFTLY	4.34
IQQIKEGTLQS	4.33
HPFFDLLKKSI	4.33
PAMVVDRCGCS	4.33
PPDFNPRDSYS	4.33
HKVGLGFELEA	4.33
GQTLVWCLHKE	4.33
QANLSLEALFQ	4.33
CVNKSKLLTDV	4.33

KDPVKGMTADD	4.33
KLWDSSRDISR	4.33
PFKEKGGCLIS	4.33
LQLNLKEYNAV	4.33
RKHSAKRCGCI	4.33
DQTNLCRLRSR	4.33
FVQELRKRGSP	4.33
TKKPAPCFEVE	4.33
KPAPSSGKDYV	4.33
RQAKKQKTRRT	4.33
DQINRLIRRMN	4.33
RQLGHQSTHRD	4.33
YQLMQEPPRCL	4.33
CTHCGCRGCSG	4.33
REETEEEKQKE	4.33
RAKASAESCSC	4.33
NPHPKPNPKNN	4.33
CDGSRVHLLYK	4.33
YVTMSSFYQNQ	4.33
WNKILMGTKEH	4.33
QPQSSDGTTSS	4.33
PQEGNKSRGAR	4.33
SPSRHRSTWNR	4.33
EGCQGETEDVL	4.33
APSSQSQPSGS	4.33
STGEQEISVGL	4.33
PIFSRLSISDD	4.33
TSKYAMFYPRN	4.33
IRKALQPHGCC	4.33
LNTTDKESTYF	4.33
GALHVYSLGSD	4.33
TLLLLETATAP	4.33
LGIFMFVYFSL	4.33
PGLCQHKVGAR	4.33
DKTIKLWKSDC	4.33
LEQWLQMTMWG	4.33
LMFTSPRSGIF	4.33
SVGASRHQGLL	4.33
ANSTMGSKEEL	4.33
AKAALVKSSMF	4.33
RYSLWFKQIMN	4.33
GYMYFYRRIPS	4.33
KIREMSGVSPF	4.33
HSDSNYTTQTT	4.33
CDADRLQEREL	4.33
TLPLILLLSLW	4.33
LKELPPVTSEQ	4.33
LFQWLGKALRH	4.33
GVANALAHKYH	4.33
LDQTLLDLNEM	4.33
VNVIYWAAYAM	4.33
VKFLLKENWFE	4.33
GDYGSNYLYDN	4.33
KQKIKMLKHDD	4.33
HLHSLSPGPSK	4.33
KLKHYCARIAL	4.33
VVSSVLTEKYR	4.33
VAAGPNPTSVM	4.33
SESLFVSNHAY	4.33
DPLGSPTLLAL	4.33
KDTMEVSSSVE	4.33
EDEEEEDEDEE	4.33
HQDTLRTMYFA	4.33
NKETAAACVEK	4.33
LQSINISGNGF	4.33
DYQYSDQGIDY	4.33
CVAHKLFNNLK	4.33
PRRRPRAMDFF	4.33
KIIRHEDVHKK	4.33
IKSNLVPPHTN	4.33
EICAYAACTGC	4.33
AKKRASTEMVT	4.33
DLHEKYSGSTP	4.33
LCYRFLFNSNT	4.33
HIHRTGKKYDA	4.33
PAKGKVGGRWK	4.33
RADELASQDGR	4.33
GLPALEFPNEK	4.33
MASIPLPDKAS	4.33
DTLAHALSAIG	4.33
LGLLLWGPGQL	4.33
CKVIKKRGNKH	4.33
FSDLKYECRDI	4.33
LASLTYWDKVP	4.33
PLSNFAFSYFP	4.33
SLLQATDFMSL	4.33
PQVHPNYRISA	4.33
ELSAWKFTPDR	4.33
VSPHLSYALTC	4.33
DSLQKRKMEVE	4.33
IMGTYVSSVPR	4.33
CLASEVSFSQS	4.33
TEMIDQEEGIS	4.33
TSGTLLRLPET	4.33
EGMPSSDFTTE	4.33
PRALRQKIVIK	4.33
MLHLHGTGIQV	4.33
FTLALSLSSSS	4.33
FLKKSKKKSDS	4.33
THQEDDGEKSD	4.33
KYSGSKTDSSN	4.33
KMAQWGNKPVC	4.33
VENFTARISNC	4.33
VGRADAPVALV	4.33
EAAEQDVEKKK	4.33
YIQEQRSQYRP	4.33
NKADYSYSGTP	4.33
KKDEEEDMSLD	4.33
HVISALVSEYR	4.33
TPPRNSAKAKK	4.33
PPCGPGLWRPW	4.33
HCVGSWSPAFW	4.33
FARKGKKGKRD	4.33
FNWPYMSIQCS	4.33
PLRDPALRQLL	4.33
DTELMDADSDF	4.33
TETSANVKTMG	4.33
SDDTVVYYCAR	4.33
REKSENADKTA	4.33
LTRDFYLFPGN	4.33
KATCLCENKIY	4.33
MAACTSREAPF	4.33
LLSATLTLTFQ	4.33
IRKFLSVLERQ	4.33
NTSKLNVFQIP	4.33
PPQGDKSRSPR	4.33
LPPGLAVKELK	4.33
IEGFLQALSLA	4.33
IEEVPGTKGSP	4.33
WGQGVLVTVSS	4.33
IDVPPEEECEF	4.33
PVLLGRLPFPH	4.33
SQHQQLEQARP	4.33
SGLGSGPKCCH	4.33
NRLVNHFMEEF	4.33
AIRRLKELKDQ	4.33
AFNWRNWISGN	4.33
NLESPEKHLQN	4.33

HTGGKILGFFF	4.33
QHSQNPRFYHK	4.33
SGATGKMILLL	4.33
DDEDDSGTEES	4.33
TLEVAGRMLGG	4.33
MDVWGQGTPVT	4.33
TLLLLGTATAP	4.33
APPPSGRMRPY	4.33
AQEAISVLALA	4.33
ALEEARKIFRF	4.33
PGIFPPPPPQP	4.33
LAHTNLRLFHH	4.33
PPVPSGFSGSM	4.33
LRKEFVDIVQP	4.33
VKEAETDSDSD	4.33
ELFLNSCPKGK	4.33
IVELKKVFEII	4.33
AATSQMAGGYA	4.33
SILFCGRFSSP	4.33
YAKDIGFIKLD	4.33
QETQRSEHKTH	4.33
TEFHAALGAYV	4.33
SDLPYHEMETL	4.33
VKLLNKVWKTD	4.33
GTMFYWSRIEY	4.33
VKSMKEPKTEA	4.33
FFNVIYWSIYL	4.33
IEYARQLEMIV	4.33
CSPPEDGLCPH	4.33
GPKKKKMKLGK	4.33
TDLPPELPKAC	4.33
KFLDALISLLS	4.33
CTILLLLYAFH	4.33
EVLRHPRDWVR	4.33
GISQESSEEEQ	4.33
KSQRSRQKKTT	4.33
KRGQSETFNIC	4.33
EQLHNHLQPGR	4.33
QQNLKKEKGLF	4.33
DSRKGGEPAGY	4.33
RKKRSKDFSGQ	4.33
QGGDLFQLLMP	4.33
SYLPLSYWQQP	4.33
DKRTTTTRVGS	4.33
AWEAYLSRLGV	4.33
SNMIVRSCKCS	4.33
ALAARLLPPAL	4.33
QAYLLFYERIL	4.33
EVRELGTVEEN	4.33
GIRKQSSVHTV	4.33
GLLCATTEALD	4.33
RDRTVQSPQSK	4.33
FVRTGKKGKRD	4.33
QLKYPNLVLLD	4.33
EEGKSDLDSDS	4.33
SPPSSEFMDVN	4.33
YKIDCNLLEFK	4.33
AQSLWPRPLFC	4.33
ANAIIHLFCFS	4.33
ITKMTLTQVST	4.33
QQVPPHQDCAC	4.33
LLIEMLQAKQT	4.33
DIQSYTTDFSF	4.33
NIFSDILADFK	4.33
TATERQYELQP	4.33
AVAIALAHKYH	4.33
NLKIKLAQAKL	4.33
KKDEQEHEFYK	4.33
SARQSTLDKEL	4.33
DSLLSAQGMNM	4.33
YMRRRSYQNIP	4.33
MTRLQTNKEAV	4.33
GEEDTAEKDEL	4.33
NIIQYFYHCLF	4.33
IEKMLNSDKSN	4.33
PPRPSHQPAPQ	4.33
SKDPADETEAD	4.33
ASEGPYAAQRD	4.33
VLLTVLLAWLF	4.33
PKKTESHHKTK	4.33
TPNSRNPAEPN	4.33
GGYVQDYEDFM	4.33
VTWIEGMMRNN	4.33
ARPGASPTPAC	4.33
EPRPPPTQEAA	4.33
RSAMQNYERRR	4.33
ESRHRQESGFR	4.33
PSKKPVADYFL	4.33
KASPVYLDILG	4.33
YRMTNQKIRMD	4.33
QRTQAPAVATT	4.33
GIEILLEKLST	4.33
LGRVANPLSTA	4.33
IDAFSDYANFK	4.33
VMFSRNKRQKQ	4.33
EANFSDLVFTY	4.33
AKYYTLFGRSY	4.33
ETFFGVQWVRP	4.33
ESDYEKYSMLQ	4.33
LKLTTGKCVKQ	4.33
ASQGKSSCSVM	4.33
ILIIIIVYFSS	4.33
GITEKAPEEKK	4.33
PSTYVEEEGIQ	4.33
AKPKKAAAKKK	4.33
PNCQIYFNVQN	4.33
SMLTKELYFSQ	4.33
PQRPSPSEPGR	4.33
KKSKKKEKVES	4.33
ESENATSLTTF	4.33
LALWFRKRCFC	4.33
VTDAIGADHRR	4.33
AEMELQAKLTA	4.33
PIMYPPDYCVF	4.33
GQSLRKKGRKP	4.33
SKRPEHLRMNL	4.33
PCQPKCPPKSK	4.33
TEKRKEKCHCI	4.33
EYHRDPVYSRH	4.33
VVMFLVVQYLT	4.33
EKQKVDLSTDC	4.33
PGCMSCKCVLS	4.33
VLMAMVKRKDF	4.33
RVKKAHKSKTH	4.33
VSFKTNKAHKS	4.33
SMVLYCQIGNK	4.33
ALLQEEWETVM	4.33
EQSATLQGDQT	4.33
PSNYDFVYDCN	4.33
GKKKLHGEYKN	4.33
KKVINKLKTTS	4.33
HVVSTIPESLQ	4.33
SVIGHLQTKGS	4.33
SRPLFSLIKVG	4.33
NGVHELVKNGI	4.33
CKVTEWVNVCK	4.33
LQNPQPMTPPW	4.33
RGSAGNKNYRM	4.33

KEKSKGSLKRK	4.33
VRSRSEGPDLW	4.33
EQLGMTNRLPK	4.33
LEEIAETPATI	4.33
EPTPQQVAQQQ	4.33
KEVLRKHLPAS	4.33
TPPLLQQNCSC	4.33
DDDHFKSTDAE	4.33
HAREWTQKYAM	4.33
PSEELELHLCC	4.33
HPLVQAQQWTW	4.33
CLMGKGLNRIK	4.33
LEALERRVHKM	4.33
GIGRLPLLNPI	4.33
RRDCGVIARVP	4.33
SVTSSDSECDV	4.33
DHGEQSVVTAP	4.33
SEQPQTAAARS	4.33
PPQYSHQTHRY	4.33
VNLPINGNGKQ	4.33
TDATPTLTNSS	4.33
NQPTEEKVDCI	4.33
PHENGEGNSET	4.33
PRFQEADSPTL	4.33
LRAVTEIAETW	4.33
RPAPEGPRAPA	4.33
STTMMIRPLDF	4.33
TEVLKTHGLLV	4.33
NSVKQYSGKFF	4.33
IAYEEIFVKNM	4.33
SGYRVAGYETQ	4.33
LSSFDGKLACE	4.33
PSTGPIIEEVD	4.33
VQQTFRTEIKI	4.33
EEFLSEKLERI	4.33
SNMVVKSCKCS	4.33
ERQGSPAGGSG	4.33
DLQGACTKKTA	4.33
VPQLYSFLKRP	4.33
VTWIEGVMRNN	4.33
YSYHLRRYSTP	4.33
PQGGRPSRPPQ	4.33
EDDEEDSSSPE	4.33
LFPANYVKLLE	4.33
PATGPTIEEVD	4.33
ESKRPSSLGCC	4.33
SRIRKIRKKQF	4.33
WKHIKHKYENK	4.33
FGANANRKFLD	4.33
ETLLPPLSQNS	4.33
CGEKRLVVCEF	4.33
TVDREHFKSAD	4.33
LVTVGAFFASK	4.33
KGLPDHPSRSM	4.33
VPRGPPSPGNP	4.33
EAFLPEAKAIA	4.33
KDVEEGDEKFE	4.33
STGEHELSAGF	4.33
EALLSQGPSCA	4.33
GAPVYMAAVLE	4.33
STCKDSKKKAE	4.33
PSQNTSELNTA	4.33
LQSSLRALRQM	4.33
LQRMKQQMQDQ	4.33
LNPKKKDSPML	4.33
LMQELFGITGS	4.33
KGFRNVIIGPA	4.33
TGTHKLQCVIL	4.33
WRDGWKDGSCD	4.33
KTGDKEEKHRK	4.33
YPGEEDGGNGG	4.33
FKWLREVRGGV	4.33
GTGHLPGLNPL	4.33
CEDLAGNAASP	4.33
WKTDASDVKPC	4.33
PGVKKSPKGSC	4.33
VFSKMAVWGNK	4.33
EVNVRKATSKK	4.33
LDVARQLNDAH	4.33
GLKTTKDCPQW	4.33
IFSKAIGDAEW	4.33
PPYKHFWTAES	4.33
VSRAVLLPQSC	4.33
ILKIDDVVNTR	4.33
LDPACFTHSFH	4.33
AQHHIYRLVKE	4.33
LKEKPQTEQVE	4.33
LCLDTSRETDL	4.33
QCHTFDSSNVE	4.33
YLGQHVHLGGR	4.33
GLQNFPEKPHH	4.33
YKCEECGKAFN	4.33
QGAPTSFSNQM	4.33
SLSMSSSSSPA	4.33
DDDFNENDEDD	4.33
PSTVFFGAFAL	4.33
GTQEQEEESHA	4.33
FPPLFLEVFED	4.33
AEEKDEDLLFE	4.33
AEVVSKGKGKK	4.33
LNKHQRLHPGI	4.33
MKKMNKEQGKE	4.33
GSPLVSMPASA	4.33
KRRLIFSKRKP	4.33
PVTKSFNRGEC	4.33
LVLEVFGNEIS	4.33
EKYGINTDPPK	4.33
FNYHSFLTEHQ	4.33
TESESSSFHSS	4.33
ALGDSTVDSKP	4.33
DDPCCSACNIQ	4.33
LHDDYFYHDEL	4.33
YSLENFMHSLK	4.33
SKSRQTMDCAT	4.33
GSTQKAEAACA	4.33
HSGLQPTGLLS	4.33
TEYSALEQNAK	4.33
MTQCLQEERYR	4.33
RNAECRGKKGK	4.33
RMALIRKTTKK	4.33
YTEDNDDDLYG	4.33
KEGKKQEKMLD	4.33
IPEDGSLNTTK	4.33
DSGTTLSSPPV	4.33
VVEQMSEISST	4.33
ISSFQQCYIFD	4.33
DSYEDEDEGEE	4.33
MDKTKRGGLFS	4.33
WGQGTLVTISS	4.33
GDKPLSDPAPF	4.33
AGKLKYFDKLN	4.33
GKTFFGAVMVG	4.33
GGLAERRHPPH	4.33
SSLEINIGPFL	4.33
DEAIALFQKML	4.33
FPALLKTLLNP	4.33
IRQRSQKGLLH	4.33
PLSLEPSKSWN	4.33

PCHGDLTESQS	4.33
GMGTVENWTDG	4.33
TLCGEVPLYYI	4.33
PYINGKFKKDN	4.33
EKETPETSGDL	4.33
AGRYITYRLVQ	4.33
ENLVPATVWDG	4.33
PLGDWSYAYMQ	4.33
ELVECEETFGR	4.33
RESTQEKKSIK	4.33
RPRYRPRCRRH	4.33
TPSLQLPGARS	4.33
LQPDHTNDEKE	4.33
SDYDLHGAQRL	4.33
CGAKKCRKFLN	4.33
WRSTQMQTACM	4.33
FRNLEGRRKRA	4.33
KVVAIVLHPFS	4.33
SGLSKLVSAQA	4.33
QNCLAVLNGDT	4.33
LRQALSRFPVM	4.33
SSQDEEDDDDI	4.33
EEGYQDYEPEA	4.33
SFEEENEGEEF	4.33
SLTKHQRTHTG	4.33
EPPPPLPPLPK	4.33
LAAGLLAWYYM	4.33
KLRKMNSDRFT	4.33
PLLQPNNYQFC	4.33
LHLEEASEDKP	4.33
TVTLKEDPGKV	4.33
KKKKKTAEQTV	4.33
DWIYRQMKANG	4.33
GKKQSSGPEMA	4.33
NEKNSVNMDLD	4.33
KSSAKRAGKKK	4.33
GSRSSDTPMES	4.33
RLVLDSVKLEA	4.33
GRGGINRGNIN	4.33
ALHAEHQKIHF	4.33
QGPEESDEFQS	4.33
KNFFWKTFTSC	4.33
QFVDHEKIYRM	4.33
KVASCSGCAKS	4.33
RQSETCSPGSD	4.33
ALDNYEGFSLA	4.33
SHQRVHKKDPR	4.33
QHQRVHTGKKP	4.33
LLSSLIYVSQN	4.33
YWLGCPAPGHL	4.33
RGHRQESVQLE	4.33
GELDIEQIEKN	4.33
QELSGGPGDGP	4.33
FHAGKKSNTCN	4.33
SRDSQSRSVSR	4.33
EVTAPQGATDR	4.33
SLFEDKRKSRT	4.33
AGEWQALTHVF	4.33
DKKRLLLGLDR	4.33
DEMPMNVADLI	4.33
GCVKIKKCIIM	4.33
PEAEGSRAAEE	4.33
YYCSVGYGFSP	4.33
SGEDSEAKAGE	4.33
APLATCDETSI	4.33
TSVSLCGRKAI	4.33
FISTQMASSSQ	4.33
YEKQLSHQSPE	4.33
RGKKKSGCLVL	4.33
FDTPVDKLPQL	4.33
TLETTVSESGC	4.33
GWRLFFQQNAL	4.33
DALVTFCEELG	4.33
KVAVGTLQEAK	4.33
QHRRKYARRPV	4.33
KEKASAYITLT	4.33
RARSESAQSKM	4.33
AAKRASRIYNT	4.33
VGATDLPGQEW	4.33
KQEVQWPPKHK	4.33
MTGYGMPRQIL	4.33
LHQHHRLHHGD	4.33
SGKDVPECKTQ	4.33
SYLGPEASPSP	4.33
FFKDGLEMEKC	4.33
SLLPPLGSHGA	4.33
DEEPMEEEPPL	4.33
QEHKDPKATPP	4.33
LYSNTPLAKRP	4.33
DEEESEAQGPK	4.33
PLLEACEFLRK	4.33
EDLRSRLQRGP	4.33
QIDKTSQTIGL	4.33
FKPLQEAECTF	4.33
NPPRSSMCAVQ	4.33
QRQQQFPGTSM	4.33
LAQQAVKRTAH	4.33
GETIELRLVSW	4.33
LKSIEERQLLK	4.33
ALVCPSVPGAT	4.33
LFGIESKSSDS	4.33
ISETGKAKYKA	4.33
RKAFDEANYGS	4.33
KRIHNGQKLHE	4.33
QTVRQGGYMPQ	4.33
DDDDDDDYDNL	4.33
GFGRGRGQPPQ	4.33
ELFPSPDPSGL	4.33
GKEQLLRLDNK	4.33
TQPTRNQCCSN	4.33
VWRSKDSEAQA	4.33
DGYEPPVQESV	4.33
LPPLFLEVFED	4.33
RHEIIHTGEKP	4.33
TASASRRSARD	4.33
KGFVMSRAMYV	4.33
RIFFGPVPCPN	4.33
NHQRTHTGEKP	4.33
IWKEIEASKRS	4.33
NMETNGVVPGM	4.33
IRSMPEQTGEK	4.33
KLCFSTAQHAS	4.33
SSSSFSQGQSS	4.33
KPETVIDSLLQ	4.33
HASSDVERMIL	4.33
PKSTEKPPGKH	4.33
PGVETLEDSVF	4.33
KKIHAGEKLYK	4.33
GQLEHVQPWSV	4.33
NMGPQYVTTYA	4.33
GSSSFSQGQCS	4.33
PRQLPPPPERD	4.33
SPEASPSSVTS	4.33
GCTKRSLARFC	4.33
YIWMEKRHRGP	4.33
GKKLCELETIN	4.33
AYAEKRMLTEV	4.33
QARLRQHYQTI	4.33

LEDGDRCKAKM	4.33
PQPTPLGQEQS	4.33
VGRPFISQYNV	4.33
SFEGVDEDEWD	4.33
TGEKPYKCKKM	4.33
VINMVKPLHNS	4.33
GLGPSHLDMNY	4.33
GSNYSEGWHIS	4.33
IMRHHKEVGLP	4.33
IYIYFFVIIIF	4.33
LEKVRKQIESI	4.33
KAAHPPSGIHS	4.33
RPRSWTCRYVF	4.33
YQDATAEGEGV	4.33
NHSSNLTKHNS	4.33
GESPDDILNVQ	4.33
HDNDKESDVEI	4.33
ERRHADERLSA	4.33
VHQRTHTGEKP	4.33
EGYEAGLAPQR	4.33
PQHVWALELKQ	4.33
KDPRANPSAFL	4.33
IDKVTGHLKLY	4.33
QHQRIHTGENP	4.33
AAEELKPRNKK	4.33
VHFDASVEDST	4.33
DEAGEKEAKSD	4.33
TGLFELKQPLR	4.33
QLKFFEENMNF	4.33
ETEAKKIGLVK	4.33
NARNWTLEGSC	4.33
AGQVAAANKKH	4.33
FLRAQERAAES	4.33
SLIKHQRIHSR	4.33
AEEPMEQEPAL	4.33
VATVAVGLYAM	4.33
KWIKDTIAANP	4.33
NEDRTALPVLE	4.33
PTAPECPPPAE	4.33
HQKIHTVAELV	4.33
GAHGSTRDQSI	4.33
DFVELLGELEE	4.33
EEISDPEEDDE	4.33
PFKCEECGKEF	4.33
RDKGLVNRGRG	4.33
KATCKDLVMFI	4.33
PAPTPACRGAE	4.33
QHQRVHIGEKP	4.33
RHQRTHSEKTS	4.33
GSPSDSDDKCQ	4.33
PCGTSQKGCCN	4.33
LVSESSDVLPK	4.33
SPDATTGQTFG	4.33
WGEGTLVTVSS	4.33
TCVTPIVHHVA	4.33
HSGEKRYKASD	4.33
WGFPKHKDVPL	4.32
EKRRNATGEVA	4.32
SSSSYGSGRRF	4.32
ACLLSAARLVP	4.32
PRTTSRGCYVM	4.32
QHQRVHTGERP	4.32
MPDMNVTKFSN	4.32
DVIRNFTLDMI	4.32
VAIKDKQEGIE	4.32
HCSGAPVAPLQ	4.32
PTEQDQFSLTP	4.32
SLQERGGASLK	4.32
HSLHSYEKRLF	4.32
GAAAEGTLRPR	4.32
SDGKAQMQSPA	4.32
KVSAPRVGGKR	4.32
DPYSSAHATAM	4.32
GDFKIKCVAFD	4.32
SSMETSPDFFF	4.32
FLDELDAVQMD	4.32
GSKKGKGKKKK	4.32
THSACKTRKQK	4.32
GVMNTEWIIES	4.32
PPAMPQPVPTA	4.32
GSRCINCCLIT	4.32
GNTDINTDLKD	4.32
IHCGKCYEKLF	4.32
QSKETTAAGDS	4.32
SANVPTEGFQF	4.32
SDSGVQPPASS	4.32
RHERTHNAEKP	4.32
QHQGVHTGDKP	4.32
LNANAAAWISP	4.32
QLCTAIDSIIF	4.32
KLPMEDLKETS	4.32
ACGPGGGDGVA	4.32
GAGAGQAPEAG	4.32
DELERVAKSNR	4.32
HLDKKTQTPKL	4.32
DIILSLFLNDI	4.32
GFSYFGEDLMP	4.32
KNLQTVNVDEN	4.32
DILCPDCGKDI	4.32
LIPSASSWNGE	4.32
IHEQMERDLKT	4.32
KKDRIAKEEGA	4.32
EAPPKEDLSEA	4.32
ANQDPESIMFN	4.32
GLPPNFNEWYV	4.32
SLEKQLGCCIE	4.32
HLKKLAVSSAC	4.32
VRKENQWCEEK	4.32
KIIHTGEKLQI	4.32
RPAFRVVDTEF	4.32
EKLLNYAPLEK	4.32
VPPPLPQFGKK	4.32
LALHRKRHMLV	4.32
SEFEPAMQIDG	4.32
FYQDTYGQQWK	4.32
GRGKNRRQSIF	4.32
TAATTAATAAQ	4.32
AMGVPEKPHSD	4.32
RRREACLVSPN	4.32
AQQTTLSLAIF	4.32
PHHYKPSPERY	4.32
QAIDDLMPAQK	4.32
QCEAPMEGFQL	4.32
CSLYQLENYCN	4.32
HEIQSDVLTIN	4.32
YESSTASALVA	4.32
VLILEEKEQTN	4.32
GLAAGQRCGAS	4.32
NPASRSQCCSN	4.32
ASMLFDIREST	4.32
PQNLWNPTYRS	4.32
DKRLAAKQSSG	4.32
GGPAPPPYSEV	4.32
MHQRTHVDDKH	4.32
PSSEDSHRKTR	4.32
MIGVLARVALI	4.32
MPCSPCPQGRY	4.32
ATHSSRFIPLK	4.32

SSSKSGMCLVS	4.32
LPAGPSACAHR	4.32
GVTIKPTVDDD	4.32
FSCVAPNVPLS	4.32
ASEQHMPQLGC	4.32
PGGDCSSDIWI	4.32
VIGRAFNADFH	4.32
KTSEELQQDFV	4.32
DIVFAASLYTQ	4.32
LTALCELRLSI	4.32
SYTNPEFVINV	4.32
HHKPGLGEGTP	4.32
RSASEPSLHRT	4.32
LALIYNEALKG	4.32
DNITLLQSVSN	4.32
FAQLRLGDVKN	4.32
GMLPANYVEAI	4.32
RFAQIVQKGGQ	4.32
VHYRTHSGEKP	4.32
RELLSNSEQLN	4.32
ILANTVKPLLY	4.32
PKDKVSLIKDE	4.32
VQDGAPGTFNF	4.32
QMLFRGRRASQ	4.32
TKKLNYRFPNI	4.32
FGQGTKVEVKG	4.32
QTTQAPSLQKR	4.32
AHQRGHTGEKP	4.32
LLEALTKHFQD	4.32
TATEGQYQPQP	4.32
FLKAVPSQKRT	4.32
AASLLCLLSKC	4.32
SIIMLEALERV	4.32
MEMLEAPHQLA	4.32
IIGTTTLNKRR	4.32
PRRSASPERMD	4.32
EGEEPGEDEDS	4.32
HLDKQTQTPKT	4.32
ARCGPCNTFGY	4.32
QTLAGHGGRRL	4.32
SPHYFSPFRPY	4.32
PCPQQPPQQQL	4.32
NAHYRPNDKKT	4.32
SEVNETDDTKM	4.32
RILTLPRSNPS	4.32
QSIDDMIPAQK	4.32
FTNTMRVVPVP	4.32
GTNMDIVEPTA	4.32
DRAPAPESDPR	4.32
EMEDNEMSCSP	4.32
NQRATKMLGSG	4.32
EEEEEDGEMRE	4.32
TSPKGYQVPAY	4.32
SKPVFSESLSD	4.32
GSSVDLGNLGQ	4.32
YNVYGTESVKI	4.32
PQQILQCSPAN	4.32
HIPINNIIVGG	4.32
AGSEGAAELEP	4.32
AAKAGLLGQPR	4.32
ITEDEAVLVLG	4.32
NPLLLSTEESV	4.32
RAFPMPGFDEH	4.32
CVGLEPPQDVT	4.32
KSRKALIPFIF	4.32
DVDLGDGYIMR	4.32
LSRRRSAQMRV	4.32
ALLQIVEPAVH	4.32
YDQGQYGNYQQ	4.32
TQQDLLTLCPY	4.32
EDSWDWVQKNQ	4.32
KGKETSASQGH	4.32
YVEFTRSLFVN	4.32
RIPTPVIHTKH	4.32
EKEQEDVLQTL	4.32
DEVVQIFDKEG	4.32
NSEFLKPEVKS	4.32
SEVGSMGALLF	4.32
ADFENSVRQGP	4.32
IASKIANELKL	4.32
FGQGTRLEIKR	4.32
VIESDSEGDAI	4.32
HLNNDRNPLFT	4.32
QQKTENGAGDQ	4.32
SHQRVHTGERP	4.32
MSWINKVIRSN	4.32
QRTSRRKERGT	4.32
KEKCEKFCKFT	4.32
AKIPGAKRHAE	4.32
DSALLPAVRRA	4.32
GLLNFVARKFS	4.32
WSYINSKSNDD	4.32
AYFHQKRKLGV	4.32
EPEPEPEPEQL	4.32
PAPKSSTCCIL	4.78
SNTANRRTTPV	4.78
GGACGGYCSVL	4.78
RRHWRRTKLGL	4.77
PSTLTIFETAL	4.77
PINSNPPNTFV	4.77
TVLLKERSTEL	4.77
RIQEAPKLTHL	4.76
KNFLAGRFSHL	4.75
KRLAILENTVV	4.75
GSPLHSLETSL	4.75
LQDEKVKESYV	4.74
TRQQKRACSLL	4.74
KPRKRANSCSI	4.73
MNDPAWDETNL	4.73
VWRSKRTDTLL	4.73
VSMLNRRTTPV	4.72
LDNALNDITSL	4.72
GPAVERPSTEL	4.72
SSKVAQATCKL	4.70
AGHNFRNPSVL	4.70
LERGLESATSL	4.70
GKQVVPFSSSV	4.70
QSQGTCRTSII	4.69
SSVVSERISSV	4.69
DPEIVVQATVL	4.69
NGTCTSRITTL	4.68
SGKSIRTVVKI	4.68
GSDMSLTACKV	4.67
VLKDDELKTKL	4.67
TVRPGVKESLV	4.66
TSVIIFSKSRV	4.65
FVLRYKIRAER	4.65
LEHIKTHELHL	4.64
VNASRTRITFV	4.64
FGGGTYVTVLR	4.64
CRPGFASESKV	4.64
VDIRLSVKLLK	4.64
QPGGCHVTCLV	4.63
RLVAFSCHCQV	4.63
SNFRNEIQSLV	4.63
FHKLIRFKCTS	4.63
IEPAKETTTNV	4.63
RQWATSSLSWL	4.63

SSSFQAEVSLV	4.63
EPQLTKRTHNV	4.62
PPPYRHRNTVV	4.62
TGLYFSVKTNI	4.62
PSTSTPVPSSI	4.61
LAQRARSPSDV	4.61
MKALGERVSIL	4.61
GDKTEEQVSYV	4.61
VRGLLLLKTRL	4.60
CSLAPNIISQL	4.60
GPHSFVTSSYL	4.59
SPFSEQKRASS	4.58
ETHREVKFTSL	4.58
EDYHSLYQSHL	4.58
RTACCHSETVV	4.58
SFHSIKQTAAV	4.58
SKRDQIVTVSV	4.58
KKSFKERCCLL	4.57
NIAVAGYSTRL	4.57
DGGRDQQETNL	4.57
KKVVGRVVFSV	4.56
FKLLDQMETPL	4.56
GRVMFKITARA	4.56
PSPDRDRESIV	4.56
GMMYLILRKRK	4.56
YSLVEAQKSKV	4.56
FGGGTRVTVLG	4.56
IRDYTQSSSSL	4.56
TMLSKTSTSYV	4.56
SSCNLAKETLL	4.55
AKDHHTDLTTL	4.55
GSWDHTLRVWA	4.55
MEAQGCSCTLL	4.55
TAKMYAVDTRV	4.54
DSESQEENTQL	4.54
PFIAIHAESKL	4.54
CVCTLTIKRGR	4.54
EKSSTMRISYL	4.54
NGSSRLQDSRI	4.54
FGGGTQLTVLR	4.54
RLRIYVSQYPF	4.53
RIRVMLYPSRI	4.53
MIKLETKKTKL	4.53
VSLAPVVTTCA	4.53
LIQHQRVHSAE	4.39
GEVVHTPETSV	4.39
NNPYAKSYTLV	4.39
VYIVIEIARGN	4.39
FGGGTKLTVLR	4.39
SHRRSQSSLTI	4.39
GAQAVEEPSIC	4.39
ENPAFMLLDLK	4.39
TCGASTTGSRF	4.39
ILCRGDRKRIV	4.39
QPGNSPSGTVV	4.39
WRMLTGRRSLA	4.39
FTVMEKKCEDA	4.39
AGPSIVHRKCF	4.39
LNAKSIRCCLH	4.39
GSLKAITAGSK	4.39
ELALYLRRSDV	4.39
TMKVTHFLPRL	4.38
GRRLTTSTSTF	4.38
GEHYPLDHFDK	4.38
TIQISELETNV	4.38
RRLLGKGREVG	4.38
KSKSCHDLSVL	4.38
MLIAMYFYTML	4.38
FGCAEPANTFL	4.38
AQQHQARQAAS	4.38
VAYLKLNTKVR	4.38
ASSSRLIYHVL	4.38
IKVVEELTRIH	4.38
WGQGILVTVSS	4.38
PSFPVSTLKLA	4.38
SLVARKLSRPL	4.38
LPQTDKCMTEQ	4.38
ATKLSRTKEEL	4.38
KRLMTRGQLPR	4.38
RPWRKHANIPL	4.38
WQIFLGRRSLT	4.38
LPASEDIKLQT	4.38
SHAMKITYAKK	4.38
PYWPHQPIENL	4.38
KESRISVQERQ	4.38
DIPGKEFDTPL	4.38
APELESKESVL	4.38
IYQRLNPCHTH	4.38
KKLSRMPPSHL	4.38
HHIRHQKIHTH	4.38
ALILQNVDLPN	4.38
VVNFLSRKSKL	4.38
ILLQVPVRPAC	4.38
SLNQAVVSKLA	4.38
LFHPLGADSQV	4.38
RFLDFIGHILT	4.38
DGLYEKLKTEL	4.38
GIYQAQSWYLG	4.38
ASSYSSSYLGD	4.38
QFFILITITKY	4.38
PNDSFTEATNV	4.38
LTFARTPSVCK	4.38
QEPEKKKKRRE	4.38
IQDLWQWRKSL	4.38
RTGRRGPTVIT	4.38
VLGERRQRAPN	4.38
KMHIFFTTFAL	4.38
RIALRLELLGC	4.38
STVSSTQVGPN	4.38
CKTSETVPRPA	4.38
VFGGTRLTVLG	4.38
NGKQDEPKNEQ	4.38
SKTETSQVAPA	4.38
IWSQEEARTRK	4.38
ELQPSEEVTWK	4.38
KYLEKPITMLL	4.38
AAENSLSPSSN	4.38
QLQLCSRHRES	4.38
TLRKKLILSYT	4.38
PINGCCSLDAE	4.38
PRSTHSRWRKR	4.38
LVWPLMAHLMT	4.38
MFRPGQKVTLA	4.38
TVFHKALYCEL	4.38
PFREPRSCALL	4.38
EGLPKPLTLRW	4.38
REEGASPLDFD	4.38
KAFQKLVRCRC	4.38
SHLGGNWARNK	4.38
VDWILEKTQAV	4.38
YIVKKRLFPFL	4.38
ISRCQVCVKYS	4.37
GRTQSSDSAYV	4.36
VETCEEALLDL	4.36
PYRSPPPYVPP	4.36
LLLILRLLRIL	4.36
SMEPNQEETNR	4.36
IQLSMDQKSDK	4.36

FGTGTMVTVLG	4.36
RLTVFVGAYTF	4.36
KELTIIKRKLT	4.36
RLSSEKGMGCS	4.36
VIHQKKTFFSL	4.36
TPGAPHHLSSL	4.36
KECMRALGFKI	4.36
LIGLHFKIKPI	4.36
EKWLYRTPLQM	4.36
VTVRVSDWTLP	4.36
FGAGTKLTVLR	4.36
GPPGHTMEVSC	4.36
SKGRPSTPLSP	4.36
RKKKVSSTKRH	4.36
EPKNLAKEVAM	4.36
LSSETGGMGSS	4.36
DPETEQVNGLF	4.36
FIMLTRLVLNS	4.36
LSRRSHDLHTL	4.36
HGQNMTVSVVI	4.36
PPYPTADHMLA	4.36
RIALQPSGSLL	4.36
AALGPQDPAPA	4.36
NQDEEEEEDED	4.36
INWSQFIPELQ	4.36
LQNSSGIEKYN	4.36
ELHELAQYGII	4.36
DLLIFRACCLL	4.36
LDFETFLMDSE	4.36
LGRVYRPCWQV	4.36
MGGYYAFYTSP	4.36
LIQFLKSSRLI	4.36
TCYRGKAKCCK	4.36
FLVVRHQLLKT	4.36
SGQNAITGFPS	4.36
KLKSSQMEQFT	4.36
YNSQGVDWWGN	4.36
SNVRINLQKEI	4.36
AFISRLAPTVP	4.36
LFSAVTLLLLC	4.36
FNQIPGNSLKN	4.36
YVEAKSSVLED	4.36
FSTALYGESDL	4.36
ILSCHCEECNS	4.36
SLRLSGRSNPI	4.36
IPAQSKDGSIV	4.36
YGRLAADYFSL	4.36
DEKKDAKKKGK	4.36
GRDTHRLPRAL	4.36
DDSKEYFSKHN	4.36
LNLVREVKIKT	4.36
EAEAEPEPELA	4.36
TVTIKLGDKNP	4.36
LSASDPGTATF	4.36
TVTVKIPQKNS	4.36
IGVLYLVLRKK	4.36
KKKFHKASQED	4.36
CRVAIYLSERL	4.36
PNMIVEECGCA	4.36
MRKAGIFQSVK	4.36
GGLWLRVEPLS	4.36
PFKDKKPCIIL	4.36
RSPRNRKPRRE	4.36
ANFLGKASGID	4.36
DLESSREQEDL	4.36
QEEKNFYLCPV	4.36
AGRNFRNPRIN	4.36
TSVTSVASACE	4.36
AGDSILTVDVA	4.36
YIDSTPCRYFT	4.36
LSHLETYRRPE	4.36
ITFSPLQPGEF	4.36
AASPSSAVFSL	4.36
SAGPKSMEVSC	4.36
HANSPNKKFYQ	4.36
PHHIGKGEPRP	4.36
IYDPACLTIPA	4.36
IEEIERLGQDL	4.36
VSQNPLASGRI	4.36
IEETTISSQKI	4.36
RSSRHSGLSHF	4.36
NSIGEFCLSGL	4.36
GPQDPTEGCRK	4.35
ACDAEATFSQR	4.35
RNFWRSSGNRF	4.35
LEEPLTQVAAS	4.35
AAAKEATKNNH	4.35
TTNEIQSNIYT	4.35
DLPLAQGIKFQ	4.35
ARLEEAVWRPY	4.35
GYLPNQLFRTF	4.35
AIQGPLTTASL	4.35
SNRKLMEIIGK	4.35
PPLYQLCFIPV	4.35
YSLGWASFPRN	4.35
ICSLHSLPPQS	4.35
DEQAEALAVER	4.35
ETASKERVIGV	4.35
YPPSPAKMTQH	4.35
SYHHPDQDHWC	4.35
KRLERIKSSRK	4.35
SGFGDPPETRT	4.35
EADRDHASDQL	4.35
GQLQSIRRVQH	4.35
LKHHHAGYEQF	4.35
NMEETQTSLLH	4.35
FRTLAEACMKR	4.35
KKAKEVELVSE	4.35
GVRAANPQKVG	4.35
FLSSSGPRVSV	4.35
SVTSSTATVVS	4.35
SLDTSIEKNSE	4.35
HRSPSESSRYS	4.35
PGAISDMIYIR	4.35
LLRHCCTRVAL	4.35
QAVRLLLLGPF	4.35
ECKLEVKVIAQ	4.35
TLFHVIRSDEL	4.35
KFQKKKAGSQS	4.35
LYGMIYVLVSS	4.35
NPVIRYKRRSK	4.35
DVVCTRIYVRE	4.35
RPESPRPPFDL	4.35
FGGGTNVTVVG	4.35
RDKQISASPST	4.35
VQVNSIKFDSE	4.35
YCIVSAFGLSI	4.35
LKREPLNYLPL	4.35
EIQLVIKVFIA	4.35
RSPRISYLHFF	4.35
NMPNKQPESSL	4.35
ISDIDDAVRKL	4.35
GQGAGALVHAQ	4.35
EAVAAQGKAKK	4.35
GRVTPKEWRNQ	4.35
LPNPCEDKDQL	4.35
KVQFYFNKENF	4.35
GATTSKSCCIQ	4.35

AGICLSIQSYY	4.35
CSVLINKKVDS	4.35
HGDNREHPNEQ	4.35
AKVFCVQRPDR	4.35
HLQPPTAEEGR	4.35
FDFTADFPSSC	4.35
RERRLQIWYRR	4.35
GEPVELNTQAL	4.35
VEDPRVLLLDL	4.35
AGSLRRVVDLY	4.35
TDFLEKEEEEA	4.35
EGYEVVTVFPK	4.35
TQSDASKQADL	4.35
PKATGVWGPWM	4.35
SRKARFGSEKP	4.35
SIQQSIERLLV	4.35
FDALEGKSIKS	4.35
PPPPPPPPSRK	4.35
VQSYPSKHDYV	4.35
RWELSSDQPYL	4.35
GQFVVRRFFKS	4.35
AVKTSRRVDMD	4.35
IVPLMRQNRTR	4.35
ARQYVLQRFRH	4.35
PAFGILVYLEF	4.35
YTGEACRTGDR	4.35
EIFFPETAHEA	4.35
EEIQKYCCSRK	4.35
ALKDLYCHKSD	4.35
IEIINKTILSF	4.35
PAGTRLPRKGD	4.35
VASVFYTVVIP	4.35
AKILQERGSRM	4.35
LQASAPNLTPI	4.35
ETPCYITGWGR	4.35
EILDYLRRALN	4.35
IYQGRLWAFCC	4.35
DGKTRLILKAR	4.35
EEFIAIMTGDI	4.35
KEKAVYAKMFA	4.35
MEKLLCDMFKN	4.35
DQDDNDCQDGY	4.35
KQNELLEPNSF	4.35
LPAPSSPPALP	4.35
IDKIFMDTLPF	4.35
ARVITDLSSGI	4.35
LCSESTATSPV	4.35
LCPRPAGRVHG	4.35
SHFIKQCFSLP	4.35
GSQRTWAPEPR	4.35
IGYPMPTGYSQ	4.35
TGFTSFGLLKL	4.35
DLGQLLPGGSA	4.35
KITIADCGQLE	4.35
QPSTPTHAAGV	4.35
GNHKNVLTFYS	4.35
IFDPQNPDENE	4.35
QFVSEKRVEQW	4.35
TQEAAVLNGVS	4.35
ESIPKKCLWSH	4.35
KFNTHFKTSGH	4.35
VQNLQAKETCQ	4.35
ELAEYAEIRVK	4.35
NRYFRKRRGAK	4.35
SPSNTSSTEPA	4.35
GGREGTIPSKV	4.35
QKNKDSWLDHV	4.35
NTTADGDNAGQ	4.35
PRGGRGRGVPY	4.35
QELLLKLLPDD	4.35
KHKRKKFRQKQ	4.35
KVYNEAGVTFT	4.35
SSIIYHCKHKL	4.35
KSGAHVDFYDK	4.35
AAKEAIKNSSR	4.35
SSCTRRNATRG	4.35
RGRTVGVSKKK	4.35
YELMKQFFHLN	4.35
KLEYSNFSIRY	4.35
SEATQQQHEVL	4.35
KVYSEAGVTFT	4.35
GEGEPFFPKGQ	4.35
EPLPPSYVACS	4.35
RQARDKNCIVA	4.35
ETVDPASLWEY	4.35
QDEYNELHMPV	4.35
FFNLIYWSVFS	4.35
MNTSSLRRNRR	4.35
NLYWICNKPYK	4.35
ASNYKELGFQG	4.35
PGSWWDKNMYY	4.35
FPVEVTLKFHR	4.35
MEISTFRIQLR	4.35
PRLVCGDKDQG	4.35
GDRESWSSGED	4.35
RALENVLSGKA	4.35
VGSLRRVMDLY	4.35
SLIVAFEGCPV	4.35
ANVGWNNSTFA	4.35
KPTHIIVETMM	4.35
SVFSGFLLFPD	4.35
LADMYGGGEDD	4.35
PPSYQICFIPV	4.35
VSRCQVCMKSL	4.35
KLAPGELTIIL	4.35
SHLKKESDKPL	4.35
SSPYTSVDEYS	4.35
LPVDIILASVG	4.35
AWREAQAEGST	4.35
RNMVVKACGCH	4.35
YEEFVQMMTAK	4.35
VAAVLVKAEYY	4.35
DLVSLIHCLHM	4.35
LLGFLLGSEED	4.35
CCFRDLCNSEL	4.35
MVSKDIGMNCL	4.35
APQWVPVSWVY	4.35
SGRARPEDLAI	4.35
GPPNYPISDEY	4.35
KMSKEKCPKGD	4.35
VSGAHSTAVHQ	4.35
HSPQNESRPSK	4.35
AEEEAATRIPA	4.35
FRLKPSLLHMP	4.35
PIFKQFFKDWK	4.35
MYRDPFGNPFA	4.35
MLVPGGEGPEV	4.35
SFHVTSLTDIY	4.35
LAPLVLCLSLL	4.35
RTGRTAPTVIT	4.35
ELVQEGDEGLW	4.35
YPLDQNLSDED	4.35
IEIISGAAALD	4.35
SLLNPPETLNL	4.35
YLEDPEDERRK	4.35
IMTPQSQSAGR	4.35
EKRKKFEKKTE	4.35
MSPYLVSLWRG	4.35

TFGQGTRLEIK	4.35
TLQIHVMECIE	4.35
GNAKPLYFHRK	4.35
QEWIPPSTPYK	4.35
EEVTGSCYFLL	4.35
EVSYVTDHGPM	4.35
LYSMQSSYFAS	4.35
IENTQGFDGVD	4.35
DKTALKETLGA	4.35
VCVQQSPVASS	4.35
RLPEVEVPQHL	4.35
SIDERMKKLGK	4.35
AGVHSNDHSVV	4.35
CFVNLFSVLIS	4.35
QQHSTFSPTIK	4.35
RNHMHEEMSQK	4.35
SIDHKPVLQMV	4.35
LYYRKMYGDMA	4.35
FPYPSSILSVK	4.35
KGGSDKCSCCP	4.35
ILCRPWTQTAW	4.35
SPPLLPLGTPC	4.35
QWVPVINLPER	4.35
AKLPCKILQRI	4.35
APFNTALKLLK	4.35
LNPDIITGFSS	4.35
TNNNVVKVDSV	4.35
LLSGGLHLLLK	4.35
SGSSSDSSDSE	4.35
RVYEVPVETKF	4.35
LDVQIQRRPSM	4.35
FPVPIDQCIDG	4.35
CERTVERYVCK	4.35
QRLCELLSAQF	4.35
IPRTDQYWEKI	4.35
APPRDFAAYRS	4.35
LDLSYVIEDKN	4.35
TTLGTLEEIAC	4.35
KLNTQSTYSEA	4.35
LQIPPPNIPRD	4.35
PGPPPNPGAVY	4.35
LAPGGSDPAGG	4.35
HELSARECACV	4.35
DYRKPEPACSP	4.35
DQVQDCLLLQA	4.35
QGDVTLSYVQI	4.35
EWLQKGLDWTE	4.35
MNRGMDSCRLW	4.35
EGKRDKNDADE	4.35
RLFPKANAQRT	4.35
MSTFASVGTHC	4.35
PAGELKFEPHI	4.35
EGRPPCLKINK	4.35
DAFGAFLKSCV	4.35
RRCPNCGATFA	4.35
MYGGVDSDKDS	4.35
WESCMDAEPEH	4.35
SVAQKAAASED	4.35
SGGGDAGPPPS	4.35
FKELKGGCVIS	4.35
EAAAGPLGCCR	4.35
EGRTELTPAYF	4.35
DRLAKEGKLKP	4.35
WGQGTLVTVSS	4.35
VHKLLTLIKEL	4.35
GAIRLGEAPAS	4.35
LNREPVLGVSP	4.35
KEKMRGMRGKI	4.35
PSHTSRTLILS	4.35
AKPRSKVVSCP	4.35
FGTRKEEWHYL	4.35
RAPFSRSSHPM	4.35
GGAPVSAGTIF	4.35
LQDWASVGAFL	4.35
KDNYRVYNTMP	4.35
VRLPYVCKFTD	4.35
QDDHVLEVSVT	4.35
MVSVQAGTQNL	4.35
DPDPKKGSRNV	4.35
YFGQGRGGGGY	4.35
KPSLVASKLAG	4.35
SVNGSSATSSG	4.35
VLEWLQLTMYA	4.35
KENPNQYNYIA	4.35
SWLDEVDHAQF	4.35
RSQQQKRQGTS	4.35
QNPKRARQDPT	4.35
SLHQALELDFL	4.35
FGGGTKLSVLG	4.35
RAGSTPVTSGP	4.35
KQSLNGPYMRR	4.35
LPISPFMLMIL	4.35
IDWKTWIWWKT	4.35
MTEYDCEFANV	4.35
IALSKSEISEA	4.35
LCKCLAMKSQA	4.35
QADAHSTLAKI	4.35
RAAGASRQAQD	4.35
NPQPACTLSPV	4.35
YSYYVYIMNHL	4.35
LYCKRSMQEWV	4.35
PHFSDEDKDPE	4.35
AKMVESQDVKI	4.35
KGSSEKCRCCA	4.35
WWMFILGAFGA	4.35
EAVELMKTGKW	4.35
SSPTHAKSAHV	4.35
NLCQELFLGRK	4.35
PATPSSPMYVD	4.35
SKKDTDEVFSS	4.35
VVLQWFTENSK	4.35
KWSVGEHPGQE	4.35
RPHVSMPQNAN	4.35
SPPEETELQAM	4.35
ITSLEVDKAGR	4.35
VNPLPTGYEDE	4.35
YEAFVKHIMSI	4.35
QCEARLEVRGE	4.35
AKPGVISVMGT	4.35
SLEYGVARMTC	4.35
SLAAPQRFGKK	4.35
KRSSFFRCVLL	4.35
PDHPAGARDGR	4.35
DPLAQDGAGVS	4.35
ILRRRPWAGRK	4.35
VNPLPTGYEHP	4.35
KMMIRPKHFKP	4.35
KDSKMKSKEAL	4.35
DEQDHQEVSYA	4.35
AMMPLLQEICS	4.35
AVPRELSPLDL	4.35
GIIGAKHSHLG	4.35
GPQAQAENTAF	4.35
FYGMARDAGLA	4.35
YHSETPYLSSG	4.35
TSAAPVPSDNH	4.35
VSISSPAHVAT	4.35
RLGSQLEASAH	4.35

SSKVVVVESVM	4.35
FAAHSASLTVR	4.35
PRLNLSAWRGT	4.35
KKRALRALRRL	4.35
QLVAAGVLDVA	4.35
ERYVTHNRAHW	4.35
KSRKLAYRPPK	4.35
EKLLSFRVDAQ	4.35
YSKLKKERPDF	4.35
TGSVSKPRSQK	4.35
CATVPPAIRSY	4.35
ASWGELKDFDV	4.35
VACKKACMLGQ	4.35
LSMDHVCLGHY	4.35
CDRKPDRTNSN	4.35
VTTLGSPMHSN	4.35
ATVMQQLSQNC	4.35
KALLALCGGED	4.35
LLTGQEQPVYL	4.35
GELEFKNDETV	4.34
ILPAKKQGGRP	4.34
AGSSTSKEAYI	4.34
RAACASASINV	4.34
LTCVQKNQAGS	4.34
SLVLLAMAEDY	4.34
GVGAGLAYLIR	4.34
LPREHRVDVET	4.34
LKRIEQIAQQL	4.34
YLYEKRTVSNH	4.34
IPGSPYRVVVP	4.34
DAESKDAVPTE	4.34
DHCPITLYLAL	4.34
RFTERRAQEAD	4.34
SLANMVEPCLY	4.34
WVCKNVNAKKK	4.34
MVRKVLELTGK	4.34
LPDLYKVFERC	4.34
ERKIRQAGVID	4.34
SGQSIRTVLTF	4.34
STPEELGLDKV	4.34
ESAIKGMIRKQ	4.34
DADFNGTKASE	4.34
VCLFGNYRPHL	4.34
LKTIKNEDETS	4.34
DPQEMLRLLGH	4.34
VSSTSSSFLEV	4.34
GIATAASYPNV	4.34
HRFFKKVPEIQ	4.34
IENGENEKTVS	4.34
DGSRAIHRKTF	4.34
SEERDDHLLPM	4.34
IYQVYDSILKN	4.34
YKLVTTGSPIS	4.34
VGGCEPEPQVC	4.34
FLQPLMAREDA	4.34
GPVSLNGCPDQ	4.34
VMVSEGIIFLI	4.34
EENLTRKSASK	4.34
VFTNAAFDPSP	4.34
LQDSHKACLGL	4.34
KGLGESRKDKK	4.34
PDQKSSGGRDS	4.34
EDAWSSSDEEG	4.34
SRSPVATATSC	4.34
VSSQPLPSQPW	4.34
SVKVEAEASRQ	4.34
LALRGGGGLRQ	4.34
STHEQVLRTKN	4.34
ADLIHFLLLKY	4.34
GNIEQNEHSVI	4.34
FPWEVPRKLVF	4.34
FLHMKEKEKSD	4.34
KSLASSPSHLQ	4.34
RGVPQSTAASS	4.34
KTAHFLPMAIT	4.34
GCFNTPSIEKP	4.34
GPEDEIGDPLA	4.34
ESPVAEKSAST	4.34
EYYCLLYYGGA	4.34
SVVKSSSWIGC	4.34
GSDESLITCKA	4.34
SSYSDANNFIR	4.34
GNIKKLLFHQK	4.34
PPLITPGSAQS	4.34
ETARFQPGYRS	4.34
VHRPPRQRDIT	4.34
SSSRHLSRTQT	4.34
GTYKWVNPHKL	4.34
ELLRHMFVHYH	4.34
EPIIDYFDVQD	4.34
KDQNPAECKQQ	4.34
KLTSPGALLFQ	4.34
VFSGFLIFPSA	4.34
ETDEDDDYMDI	4.34
IDWINSVIANN	4.34
MENGSLDTFLR	4.34
KAMFARFIEME	4.34
FGQGTDLTVLG	4.34
TGVSCPVGIKK	4.34
IGAKQKMHDEE	4.34
SMSIAPRMMSV	4.34
LFLPPWLAGMI	4.34
ITLLKICGGDD	4.34
KKYVSYNNLVI	4.34
QTYSSILKHLN	4.34
VASAEYHRKAL	4.34
LIFDVELLNLE	4.34
DYVHNLNLLQC	4.34
TLGLQENMRTS	4.34
DNSLDKLKTDC	4.34
IKSKTSKRKIR	4.34
EFSGPPARNQT	4.34
SLAGLGLWLLH	4.34
RESAKSELTES	4.34
DGDEELLRFSN	4.34
IMSSGNVNSSS	4.34
KLQSCLEAVAT	4.34
LRLELFGCDIY	4.34
AMLVGKDLKVD	4.34
DPVLIENSISI	4.34
AEFAPNKRRRY	4.34
LKQKRPVTEMP	4.34
KALLYLCGGDD	4.34
LKDVNVIPATA	4.34
RQDISVHKLKL	4.34
YVAGTGGLGPA	4.34
SVVPQEQEHAM	4.34
MEQTALSPEVR	4.34
EGGPKEEESPV	4.34
KSLQAKLERLH	4.34
PFAELVLPPQQ	4.34
NLIAEGLKSTE	4.34
REWFFNLLESG	4.34
EKILVALCGGN	4.34
NGSQNSSYSQS	4.34
GLTLQWILTSR	4.34
VLKGKKLSLPA	4.34
WVKPKDMLGPK	4.34

HPPEMNEAAVW	4.34
FYSMAKDAGLV	4.34
MIKPVEVTESA	4.34
EFNSYSLTGYV	4.34
WGQGTLVSVTS	4.34
LHEWALREGEE	4.34
PPPSPGHSLQH	4.34
GVTSTRVYERA	4.34
LPSAEVALQAK	4.34
LSFLKRQRKGL	4.34
IIGVQHQADAL	4.34
QAPVTFKGFRE	4.34
IPYSQETLVVD	4.34
LNNNDWYSQRY	4.34
MKFLNWATPNL	4.34
YLAHAIHQVTK	4.34
SLYSLQFAGGN	4.34
SISRSVSTNMD	4.34
VDAESIQQMAP	4.34
EVQKIQTKVDQ	4.34
KKNKEGPMNVN	4.34
SAFKNFFSRQH	4.34
KSNMPLAPGQF	4.34
PGSWWDQELYY	4.34
AVFQQFCALQA	4.34
LLGPELHSPGF	4.34
ITMNPGYAGNS	4.34
FAHYGQGTLVT	4.34
LLIEMLHAKRA	4.34
INEDIEETNIA	4.34
PRPFQLCLRPR	4.34
PMNPPHKCEVW	4.34
PSMSRDLFHYR	4.34
GQFLEELRNPF	4.34
MEALENRLRYR	4.34
EVQYFFKILDN	4.34
QHLIKLYSPEN	4.34
LLDSLQPERGK	4.34
PYNDYGDSKEI	4.34
PGGWVEKETYY	4.34
ENKGGTESLDP	4.34
PTQNARETEAP	4.34
TPEHTWSSRKT	4.34
IKNLRMGSEQL	4.34
DIEFKYKFKHL	4.34
SSHLDTLGSKS	4.34
EPKDESARKND	4.34
KADGAEAKPAE	4.34
KANLENPIRLA	4.34
KKREKLILTLT	4.34
CLYRSLKLEHY	4.34
SINSRPSQKLQ	4.34
RTLKEVLTCSW	4.34
GTLLAHFFIKF	4.34
QKKLAGDESAD	4.34
SCSTLLYGRTW	4.34
TISLSENGEEV	4.34
QCDQKGSPVPP	4.34
GVHRKEPQGAT	4.34
RVTQQHNTVFS	4.34
KRMRLDTWTLK	4.34
KCKSLRLPSDS	4.34
ALLDKTRYTEC	4.34
TWQSRRLMKKT	4.34
GFEVGPACFMG	4.34
AAEPAPSQHLW	4.34
KEAEEESSEDD	4.34
CVPADDRILCR	4.34
TTVEKKVMIHD	4.34
LKNLRNSPKNC	4.34
LHSPITTSPSC	4.34
QYPHINGSVKT	4.34
AGSQSQVETEA	4.34
VDQTVKVWECR	4.34
FRIACSFPRRD	4.34
LYGKRSNSRKK	4.34
AANIQADYLYR	4.34
KKAARGGKKRR	4.34
MTGISSLYYSS	4.34
CLAETERNART	4.34
FNLVYWLYYVN	4.34
EAKLKKLSRFA	4.34
HRSDQGKKAHS	4.34
YDTTKQRPDEW	4.34
GDDEFFDLDDY	4.34
EEEGKVRSTPM	4.34
PIQMIVLTYFP	4.34
KLEQQMAKIAA	4.34
LQARQASPAWK	4.34
KMPEEREEKAE	4.34
TRSKERLNAYT	4.34
LMFKTEGPDSD	4.34
EDEDVIQIVKK	4.34
AYEKYIKSIEE	4.34
GKLDRTFHLSY	4.34
CFIVARKKPGP	4.34
ATVASEKESVM	4.34
HKESNDCSCGG	4.34
QPFGPKGGYSY	4.34
KKSKRSMNDPY	4.34
DSRTPVTERVS	4.34
KGASEKCSCCA	4.34
TQVWEAMVLYT	4.34
PELYKDILSQS	4.34
LALNIDCDLLG	4.34
KRMRLSEGWAT	4.34
CKDAASAASDA	4.34
TSIHEAVTKIQ	4.34
WIEQWIKDHNS	4.34
DKNKDDELTGF	4.34
LIGARYISGIP	4.34
GDVQLDSVRIF	4.34
AASCARSCGSC	4.34
SPAVVMPTMGR	4.34
IFQDKVNSDMH	4.34
MARKPSRAYKI	4.34
IWEALSVMGAV	4.34
TTELPELTSTS	4.34
LASVPALPSDS	4.34
DESENQARVRI	4.34
MLSQTSSSLSF	4.34
TGAAGKFKRGK	4.34
RRRKRKPKPGP	4.34
TLGKIAEKCDR	4.34
PKPPGSLEIEQ	4.34
RQRLPAPDEAI	4.34
EQPESESEPKG	4.34
KRSKKALANAL	4.34
PRNYTMSFLPR	4.34
CHDKSLNKKSG	4.34
LIYDSSLCDLF	4.34
TSSMTTTETTS	4.34
ANSDQTVTVKE	4.34
HKPSYFWKDMV	4.34
ADLFQVPKAYS	4.34
KFKWRKDPQDK	4.34
VAPPEYHRKAV	4.34
MSGLPADNLAT	4.34

DGTGSPRLNDR	4.34
KPHINFMAAKL	4.34
SDQQQNKLRSS	4.34
GGDIQLTHVQT	4.34
DCVFGGQRLTL	4.34
RRKSLKQKKKR	4.34
FYRLAQESGLV	4.34
PISPTQPLTPS	4.34
EELMQELSSLE	4.34
PCEDTSLQPTS	4.34
SLGYSDLFCLS	4.34
LAIMWFGTNTN	4.34
QAEGTDKSDLP	4.34
DVLHEPFLKVP	4.34
LCLRLASLIEI	4.34
RLQKKGGKDKT	4.34
QSKATEECTST	4.34
PLAPVHNPISV	4.34
LFLEMLEAKVC	4.34
PFKEKGSCVIS	4.34
HILKSNSWLHC	4.34
HTSDGDALLLL	4.34
STPTDVRDIDL	4.34
STFNWPYGSGQ	4.34
RTGDGVLVPGS	4.34
KLKHHCIRVAL	4.34
QTPEIAAWSRA	4.34
AEDYLDDEDSD	4.34
CEMLSLLKQYC	4.34
IIQSKEDDSKA	4.34
QEMVVEGCGCR	4.34
SSPFGDYRQYQ	4.34
IIAILSQRPRK	4.34
ATLPGFRTIHC	4.34
GLSSDPADLDP	4.34
LLYRLAEELLG	4.34
RLQYFTGNPLF	4.34
KRTGDGNYKSG	4.34
KKKGEALFPAS	4.34
LCQKTMAPVAS	4.34
VPRSTHTAYIK	4.34
VHGGEAARGPE	4.34
EHRVHVQMEPV	4.34
TSTEYSEVRTQ	4.34
QQSEELLEVSN	4.34
DMFRLALHNFG	4.34
PGSYVPWNVPI	4.34
DINSRVEMKPK	4.34
EHEDVIQIVKK	4.34
YQKAPTKEFYA	4.34
ILCGCNGMNLG	4.34
SVAFPDETMPN	4.34
QRSDLVKHQRI	4.34
LVLYDELKKVI	4.34
LYGVYCFRAYN	4.34
CIFPSAPDVKA	4.34
EDPLPTAASGK	4.34
SWREALYGCHA	4.34
SKTFEKKRGKK	4.34
IGRSGIYETRC	4.34
VPEMTEILKKK	4.34
AASVSGLASVV	4.34
GNNRVGFATAA	4.34
DVRQYVQGCGV	4.34
FLCEPDSPKCD	4.34
LGKFISTVHPI	4.34
GRISRARHNEL	4.34
VAGYLRTATGD	4.34
LSSLAYSGKDA	4.34
QGTLVTVSSGS	4.34
VKRMRMDAWVT	4.34
RFRPDGLPKKY	4.34
SPSMEIMQVRK	4.34
LKQLLMLQSLE	4.34
SSLPYAEGFAY	4.34
TGEAEGFPATV	4.34
IREMLMARRAQ	4.34
PGKKPTPSLLI	4.34
PDMKPRVSCPP	4.34
CDFGDLTPLDF	4.34
LDEQIYQEDEC	4.34
NVLYWLVCLNL	4.34
NQYIEWLKENL	4.34
DYIADKKDVYE	4.34
SAEEEEEKQAE	4.34
RQENGNVHAIA	4.34
MQQMHGRMVPV	4.34
ADAEEDDEVKV	4.34
YAYIYCLCAAV	4.34
DAFSGLFWQPS	4.34
IEKMLKNGKSN	4.34
NKHCRGSTPRC	4.34
GPGSVHSDTSN	4.34
DLKRKMILQQF	4.34
STDSGAFSIGH	4.34
IQDDFDWDSIV	4.34
GSDESLIACKA	4.34
ALVLTGGEKKP	4.34
KNPGPSQGKSM	4.34
LIPTQPSQGAP	4.34
TPEEGEKQSQC	4.34
FPRSYVQPVHL	4.34
TNLQKGLRRKD	4.34
DDDQKAVKDEL	4.34
ATSGSPPAGRN	4.34
VQHEGLPKPLT	4.34
NYEKNNVMLQW	4.34
PVLLSSNSQKS	4.34
SFPVLATEPPK	4.34
PKKKKKAQEDL	4.34
DTLGIKPGPRV	4.34
LVLYDEIKKYV	4.34
PDQRDEAPLTR	4.34
LKASAPAPGHH	4.34
PDLTMFPPFSE	4.34
VNPIPVTDEVV	4.34
LTTIDLQHLNY	4.34
WEDSAGHWLYE	4.34
RRRTLHQNGNV	4.34
QLKSRQILGMR	4.34
SGHFHTVCLLV	4.34
CFNEESPKIGN	4.34
RRAIEQLAAMD	4.34
AIMEHVRDHPY	4.34
SAKDPAQAPQA	4.34
GLVTPHKTELV	4.34
RLPTPSAPLHS	4.34
SILFLGRFSSP	4.34
INLQKRLKSKE	4.34
EALKDEEKAGV	4.34
CVNIKKIFTDV	4.34
KVQLVNGMVPL	4.34
TPTDPKGLAQL	4.34
AWTRLYAMNNI	4.34
KFCGFEGLKLT	4.34
PLLQEIYKDMY	4.34
LSDHIEQMATE	4.34
GVLLGPVCFMG	4.34

CELQEENQYLI	4.34
PGPFSPDLQKT	4.34
CHCSTCYYHKS	4.34
ETSVSNDYKMR	4.34
DGEGNPDDGAK	4.34
PAGEASPGCTP	4.34
DDDTSRMEEVD	4.34
GGKSMSHNEST	4.34
YEQGLASELDQ	4.34
FRKAFLKILSC	4.34
GPGEVDPKVAL	4.34
TDRSSHKDSMN	4.34
GILCSALQKIK	4.34
VRKARGKKRVP	4.34
PSKNYVMAVSS	4.34
PPMKTMKGPFG	4.34
VHLDQSIFRRP	4.34
IENSFMTDARK	4.34
YDRDPEGKVQP	4.34
EHRQERRDRPY	4.34
QIALLVKDMSP	4.34
TQDPACKKPLG	4.34
DWIKKEMEEED	4.34
SVSTVLTSKYR	4.34
ESPDFPEELEK	4.34
GPAAGGSVAAS	4.34
VELDDLGKDEL	4.34
GVYSRPVLNTS	4.34
YGGGSGKSSHS	4.34
EMGAIKNLTSF	4.34
LLQRKASPPTV	4.34
ARLRFMKQFYK	4.34
EEVSSPQMKDQ	4.34
LPSDGVQNVNQ	4.34
LQLNLREFNLV	4.34
RIRSGMFWLRF	4.34
EECDAAEGAEN	4.34
TMNGAPVEPCT	4.34
PLKGRTVRASF	4.34
LDQQNPDEDFS	4.34
DDHDGGEGNNN	4.34
RLQSTNFALAE	4.34
TGGVLNPTVTQ	4.34
KLPLVEDFMCS	4.34
RFYTIEILKVE	4.34
PPAAATAYDRH	4.34
APRRTPARFGL	4.34
SYTTISTLGPG	4.34
LASPALVSWNW	4.34
GWPRQADAANS	4.34
DFGRRSAEDEN	4.34
QIDELLRQSLS	4.34
KLDEQEPFPHS	4.34
PVPPSPAPGGS	4.34
DVVAVRHYEKA	4.34
PLHRHGSDPGS	4.34
AGGNGSSNVHV	4.34
LPPNDHVNSNN	4.34
FRKNFRSILRC	4.34
DDLMDVALIGN	4.34
DTAFPPLHYEL	4.34
SSFSGFLLCPT	4.34
STPTDVTAIHL	4.34
TKRPAVCAAVL	4.34
SDVWGQGTLVS	4.34
GQGAPLLEPAP	4.34
LRWVRKTPWYQ	4.34
MLKCDPSDQNP	4.34
GMEGRKKKFES	4.34
PHPTIKMEMAV	4.34
APAAEPVPAST	4.34
FDMADGVKTQC	4.34
ACLMGRGMKRV	4.34
GWQPAPPPPPC	4.34
WGQGTLVTVST	4.34
SETLNLSEKCI	4.34
VPKLYEQLSGK	4.34
KQCSENVHIST	4.34
QYQYFPNILCS	4.34
IRRRTDVRITG	4.34
AAPTDPADGPV	4.34
MFDAAKSPTSQ	4.34
SALNRMKELQQ	4.34
NHVSSNSTGSS	4.34
TIEQEKQAGES	4.34
VPKPGDGSLTI	4.34
HWINPHMLAPH	4.34
NFFWKTFSSCK	4.34
RLRAILQNQGN	4.34
TDKEKQWSRWK	4.34
LPRSLAPAGKD	4.34
FIKQQREARVQ	4.34
DEFLKMMEGVQ	4.34
NHHAHNYYNSA	4.34
FIKAIVDLTGL	4.34
GKKYDSDSDDD	4.34
PRNLAKSVTVE	4.34
GKVLKLVPEKD	4.34
FAHWGQGTLVT	4.34
PCSTDTEHGNV	4.34
NQENVLTFYCQ	4.34
SKNYNMMTVSG	4.34
VDSRDGTLSGE	4.34
EEEEEEEEEET	4.34
LGSKEDTVISL	4.34
FFPPEDDQNKG	4.34
YPPYYQEEAGY	4.34
AAEAQDGPQEA	4.34
RRRTGGYRVQV	4.34
AVSVLGTEPNS	4.34
CLYSRLTICEF	4.34
PIGEDEESESD	4.34
ELLIKNEDILF	4.34
DPGEMSPPIDL	4.34
RAKNKVHPSST	4.34
FRDFDYIADWC	4.34
SVMKVKAELCL	4.34
YEMKFPDLCVY	4.34
PVYLRSHDEVS	4.34
GQYGQSKPWTF	4.34
NLHRNEDSVLF	4.34
LEKNKKKGKIF	4.34
PIEIQQVIVSE	4.34
KMRRRKACTIS	4.34
SSCPRGNAPRE	4.34
RPEDGHCSWPL	4.34
WNRKQLTYKNN	4.34
HPEDQGRREGL	4.34
SRRQTQAKYGF	4.34
GKKYLRYTPQP	4.34
MTDTPGNPAAP	4.34
GIADSVSSIFV	4.34
ILVPLSHLAQN	4.34
AANEPLKTHRE	4.34
RAEVYSSKLQD	4.34
SASYKAKKEIK	4.34
TSPINPAVALE	4.34
SQLLPQENRLS	4.34

TADSMWRPWRN	4.34
RRPTRSSTLER	4.34
LRHKALETLKH	4.34
YEVALEYLNSG	4.34
LGVPDAGMKPS	4.34
LRFNAIALSAA	4.34
AIRTGAAIFTQ	4.34
MQKKLLGKDAN	4.34
EVSPHVKTGTK	4.34
VLANLAHPPAE	4.34
SIRNLASWFER	4.34
QAADGAKVLCL	4.34
TSKPPGTTEWE	4.34
LEDLRTLEHNV	4.34
AQLGGRSCKIR	4.34
LTEYYKSLEKA	4.34
ESSDSESSFSD	4.34
PNNIFGNRNLL	4.34
EDMIATKCTCR	4.34
RQGRTLYGFGG	4.34
VFFAWAQKVGL	4.34
ESNPDPVHSEF	4.34
RRSSPQAMPCT	4.34
PPLDSKGANSV	4.34
GDLLRALDQAT	4.34
LSNESNKVNGN	4.34
HELCKSYRRLQ	4.34
LVAERAGTDES	4.34
RQKASGSHKRS	4.34
NLQNVDMKIGV	4.34
LDHLKSLIKED	4.34
LLLICIIVMLL	4.34
ELYLQHLTALH	4.34
SDKKLPEMDID	4.34
GSVRGTSAADV	4.34
FAKGFRDTGRN	4.34
EWQDPGRPLES	4.34
QGIAVIIQSTA	4.34
PATKMETEAHA	4.34
QVLGQPHKKSL	4.34
FSISKEHLECR	4.34
EGEGGAGGEPG	4.34
MKLRNLRPPPE	4.34
IAADLSPLLKH	4.34
PKFAVATLPPA	4.34
ANCVSVLIDHF	4.34
EHRQDRRERPY	4.34
PGGGALVLLEA	4.34
STPYLLFYKKL	4.34
DRIGSMSGLGC	4.34
PDHWTGLGLNC	4.34
LRQLITCRAAA	4.34
SVAPRRPQEPR	4.34
PEQQAAILSLV	4.34
LSSFDSKLACE	4.34
EEDMNLGLVRL	4.34
QKEKLRCENNA	4.34
DKMSALAAYFV	4.34
PTEVQEPIITA	4.34
AYSDVAKRLTK	4.34
TAGAIMETNLK	4.34
TLQQNAESRFN	4.34
LPWIKRTMKRL	4.34
CTEIVDQFVCK	4.34
ITGIQAFTASK	4.34
LIGARYISGVP	4.34
GALHVYSFGSD	4.34
YEISIGAEEKT	4.34
ADEERTESKGT	4.34
DCSPQNGGPGH	4.34
RNKDDSTKNKA	4.34
SPAKLKATAGH	4.34
VKKRKRKCLLL	4.34
GALHPARCSLM	4.34
EHIAKRAMENM	4.34
HKLNLRGKAWG	4.34
IWTDRPCVVFS	4.34
QLLPQHQVPAY	4.34
AVAVFLWGFYA	4.34
YQEQTGGHSTV	4.34
MCGHELTYEKM	4.34
HVHPLFKRFRK	4.34
KIVSKLKDTVS	4.34
VETATELLLSN	4.33
EGMIAESCTCR	4.33
PASALHSVAVH	4.33
VSDIEAVRASW	4.33
NWIIMTIKGAV	4.33
KFNKSYNGTVD	4.33
SSKFNNDDSVA	4.33
AEESKFLNMMT	4.33
SPCQYAVERPV	4.33
RHRARINTDFE	4.33
TAKLQLVEQQA	4.33
FWPFNQEATQQ	4.33
SLGPRVTDVAT	4.33
EAPTEDDHDEL	4.33
EKRLVPQVVTD	4.33
CILFCGRFSSP	4.33
AGKGRVGGRWK	4.33
EEEEEAAIVEP	4.33
EYNGGLVTVSS	4.33
NQGRKKQVVQF	4.33
AQAVRGLITKA	4.33
AGTGCCLCYPN	4.33
SAIVEQSWRDC	4.33
APKNADWTTAT	4.33
SCCGCSGAAPE	4.33
LQENLKDIMLQ	4.33
TSSSSRKSYKH	4.33
WDIAHMISGFE	4.33
APAPKASGKKA	4.33
LDQTLLELNNM	4.33
KAEEAWAQMEE	4.33
LQGPSQSSSEA	4.33
FDPLIYGYFSL	4.33
EEMPMNVADLI	4.33
KQEGTPEGLYL	4.33
RSGASILQAGC	4.33
SYFIKKKLSKA	4.33
DCVDTEIKFFK	4.33
LPLHDFLCQLS	4.33
CCAIRNSRDVI	4.33
IKNNLKECGLY	4.33
QGACVTPASGC	4.33
TNLGNLEESSE	4.33
DEDLPEERPDD	4.33
KSNAAERRGPL	4.33
PPRTANPPKKR	4.33
GILFCGRFSSP	4.33
RFTTKRPNTFF	4.33
DWIKDTIAANS	4.33
PAAVREKGVQN	4.33
LLVAHVVVGWN	4.33
PDLPYAPCIQP	4.33
VYKGQLQSAPS	4.33
RQTLLQKMSSF	4.33
WFYAHIIPFLG	4.33

LVPGPAAGPLP	4.33
GVLNNPGPGPA	4.33
MKKMNKEKTDK	4.33
FVWKQDVTDTP	4.33
KKRKRKRLKPT	4.33
ISNLTNPPAPK	4.33
SRAWTESSINT	4.33
QLMLTQQLQTF	4.33
GGGRMGTWSTR	4.33
QAGVRGLGHQS	4.33
TSTTVVAPLSS	4.33
STTEKSLLKEG	4.33
TLRRVEAKGRP	4.33
LEGSEGDGDTD	4.33
KEGSRSYCTDS	4.33
SVKEISDIVQR	4.33
AAASSEDIERS	4.33
AQAANTASASW	4.33
VVEVKDPVGTK	4.33
TPHVNHHMPPH	4.33
HVTTNGLQDHS	4.33
RHEDIHHQQQD	4.33
WGEGDLVTVSS	4.33
ASGLNACQYNS	4.33
DENDEVKITVI	4.33
AYKSRDTAIKT	4.33
LNNSIYKKLLT	4.33
STTAVVTNPKE	4.33
RSRDRERSGRF	4.33
KGQVALCKDCL	4.33
LALTVARPRWR	4.33
SWIRKVTGRSA	4.33
SLHHSQVPTVN	4.33
SFQATNEGTRS	4.33
LADSHGDIITA	4.33
PRWARGCSTGN	4.33
DHHEEDMDMSD	4.33
LPSLLLSLLPW	4.33
LQCDPSSASQF	4.33
GLMKFVDSKSG	4.33
CIVLALQRLCR	4.33
QNSPEACDYGL	4.33
CTWQSLRSQIA	4.33
SNQQFQPVMAN	4.33
RGKAKVTGRWK	4.33
EFTSDDMVIVG	4.33
SAPEAEQGGAE	4.33
AVLFMGQINKP	4.33
SLTEQRLTVES	4.33
PRGPPPAGLLH	4.33
EYEGPNKKPRF	4.33
DGKNTGSNTTE	4.33
NLLMTLRLWSS	4.33
DVISEGRGKTH	4.33
YERKKYGFKKR	4.33
RYIYYLDWMWS	4.33
ILAYLKQFKTK	4.33
KEEDESDDDNM	4.33
PVDFSDLPWPL	4.33
TTTVQDKDESE	4.33
PKFSISPDSLS	4.33
IEVAHKWPLKT	4.33
MYREYIRNRYL	4.33
KIVRREDVHNQ	4.33
AKKSFLRRLFD	4.33
LFGSGTTLVVT	4.33
LCGIPGSLSAG	4.33
AQEALTVLSLA	4.33
IANNLRGCGLY	4.33
HSGLQPTGFLS	4.33
GYIYFYQRVAS	4.33
SCKTCNISVGR	4.33
MLACLLAGFLR	4.33
LAQQRRKSAMF	4.33
TKNLCLLETAQ	4.33
AGYGMASYQTQ	4.33
EQKIEAETRAV	4.33
HSGLQPRGFLS	4.33
GDPRPYLPSPD	4.33
EMRKKEMLGKF	4.33
NVLLKPENLYN	4.33
LDQTLLELNNL	4.33
DIQQAFRNLED	4.33
FNDLDNQIKKM	4.33
EPKKSRRCVLL	4.33
LHDNLKQLMLQ	4.33
SPTSSLGEERN	4.33
GAIYDAILARK	4.33
NTMQMSLKDLL	4.33
LMEPPAVLLLQ	4.33
QTSVPFDHLGK	4.33
LSPFTFAATDD	4.33
KMRPGEATLSC	4.33
AHDGKEHRFMV	4.33
IRQGVLEHILS	4.33
DDISSRIFTAD	4.33
AILEKLEKKGL	4.33
KLMPWVLAMIR	4.33
RIGLLPLLNPT	4.33
GMNFKTPRGPV	4.33
SGYILFYQSRD	4.33
ETQPKRKCCIF	4.33
LIEEETGQKKI	4.33
EVAEEAQSGGD	4.33
DFSALLSQISS	4.33
HYNNLSASKVL	4.33
NILFLGKVNRP	4.33
HKIQKQMKENY	4.33
VTETMEKGFSK	4.33
DTLLSLFLNDT	4.33
ALEYLEGLESR	4.33
DSVYWKVPKTR	4.33
TSVGFFNLRHY	4.33
LPQALPTSPPW	4.33
ISVVCFFFLVH	4.33
PAPCPTTYAQH	4.33
EKNMSIKKLWK	4.33
PSLCHRNSNGL	4.33
AENKEKETAKS	4.33
EGADEAAEAAG	4.33
GSVLTIKKESE	4.33
DSEFDEVDWLE	4.33
YVNITKMLTDV	4.33
GKKATQASQEY	4.33
LKCRIIHNNNC	4.33
TPSEEVASPHS	4.33
GLIVALILSTK	4.33
KNGVYIKIVSR	4.33
TKKYGEKRPVD	4.33
NLEMVGEGTRQ	4.33
AYRLLKSKLYL	4.33
HSGLPPTGFLS	4.33
DEDEEEDDDDE	4.33
EKDSESGGGKD	4.33
TNGIYPHKLVF	4.33
QFAFSKALPRS	4.33
VFTKMAVWGNK	4.33
KTESHHKAKGK	4.33

YVQEMAKLDAN	4.33
IPAGLPSPRSE	4.33
LQYLKIMAGSK	4.33
NDFKHTKSFII	4.33
QWQQKGRLHSY	4.33
RIQEKLDRFLQ	4.33
VDWIHKVMRLG	4.33
EMAEDDDDSFP	4.33
RTKRLVVFDAR	4.33
SNMIVKSCKCS	4.33
RFSAVALCKAA	4.33
SLEEARKIFRF	4.33
AKKNFNTNLLE	4.33
KASPTLDFTER	4.33
SRVSQGKSEKA	4.33
IVSGNLRILDH	4.33
IMAAPSAWFLE	4.33
PHPSSMVTAMG	4.33
RKRSRDPEEEL	4.33
QGGQQAGGGCC	4.33
EILKMFLSGLS	4.33
RPPMRRWSSVS	4.33
ALQHAFIQEKI	4.33
SGQNLLAVESQ	4.33
TKNSKRKKRCC	4.33
APKTLPWGPKR	4.33
DPQYFEFEFEA	4.33
LRMMDGDLSSP	4.33
EELLRALDQVN	4.33
RHQPSWKPLMN	4.33
LQLNLKEYNLV	4.33
LVMSIRRSALM	4.33
TPKDWSKRGKW	4.33
KHQRTHTGQKP	4.33
PRSVLEEIGLT	4.33
SHIVRGEHTFH	4.33
EVKRARINPDS	4.33
KSSDKEEKHRK	4.33
PIPVDRSCGGL	4.33
GLEVVIKNDSN	4.33
RDIVFAASLYL	4.33
SRRFEELKKKT	4.33
LFGKSATWNSK	4.33
ASSGPTIEEVD	4.33
IARCLNVDWIP	4.33
LPEEERNIQWD	4.33
SKSIKFIPVST	4.33
NCFLNLSPRKP	4.33
SHIKRTSHESA	4.33
MKVQMLRESPF	4.33
VKSEEDRSRWA	4.33
YVQEMAKLDAK	4.33
LTEMSQDAPLA	4.33
DVKKEEKKEKK	4.33
SGQVYFGIIAL	4.33
SKDSGPPADGP	4.33
LPGVLCRSHPK	4.33
PQPTITKQEVI	4.33
RAEGINCNNQY	4.33
AEGQFYNYFPN	4.33
QPELFGLPSYE	4.33
SMAAAGGAFRP	4.33
TSILIQKSGPC	4.33
YDVALEYLESS	4.33
DMQNAFRSLED	4.33
CTGDCWPFRWS	4.33
VSTDQLNKIMP	4.33
LSETTNPMGHM	4.33
NSRLQRERANM	4.33
KEQNKIGVKLS	4.33
AQKAEGAGDAK	4.33
KQKDIDKDLVI	4.33
KRRTGQPLCIC	4.33
AKPDRSRSASP	4.33
CNECGNRWKFC	4.33
GGTVSSSSYNA	4.33
TTVVNPKYEGK	4.33
ILQTSRVKMGD	4.33
SSQDPQTEGTR	4.33
LEGDDLILEIE	4.33
LRARHLRDYPD	4.33
RVQKLSRVINM	4.33
YTDDIPLLKEA	4.33
PNMSVDTCACR	4.33
VDLMAHMASKE	4.33
PACKDPEEEEL	4.33
KGPRDPPSEWT	4.33
IYEWTKINGMI	4.33
PALEEGLTREE	4.33
YQTNHIRENAY	4.33
DHPDYPFSDEY	4.33
WGQGSLVTVSS	4.33
DKEAPQKSWAP	4.33
ETIPMASRGGA	4.33
YTHSQNLSQEG	4.33
ILTHAKAKNNQ	4.33
ETIWYLKYHWP	4.33
TTAKEGKSDSE	4.33
LTGKYPVELEW	4.33
KTMVTRFNEAQ	4.33
ISSVEDFTEFV	4.33
FKESKGMMNDE	4.33
ACSTSLAPVFP	4.33
LNVEWAKPSTN	4.33
YRPTEDSKEFS	4.33
VTTSELEGQGS	4.33
SFYDLEADQYC	4.33
NLISKLFCSGT	4.33
ALDAADRAKKK	4.33
PCSLGTFVLVQ	4.33
ISREWTQKYAM	4.33
AITYAKGFGML	4.33
DPKPAGNPTKA	4.33
DAHLVALAERD	4.33
EYLGSFDHYAT	4.33
RRSRSPDRRRR	4.33
IAGDSDLDSDE	4.33
LRENTSRLYSL	4.33
STLTPHKTIHI	4.33
CGSNSYVLVPV	4.33
DGSVCVLDLRK	4.33
GAAAVFEYPGN	4.33
EPPKAKDPTVS	4.33
QPASQGSGPTA	4.33
VEVPYVRYTIR	4.33
AVVQFEQHKRH	4.33
SMLTKELYFYH	4.33
ASKEYFQKVNQ	4.33
TDEEDEDELIL	4.33
SMRDSLIDSLT	4.33
RKYQEMTGQVW	4.33
DLSMTQKDKFM	4.33
AAVLWRKKSSD	4.33
ETSSQESAEED	4.33
MDQLAFHQFYI	4.33
TTFGKTNGYIA	4.33
DSLESQDSMEF	4.33
KDPSSEKSQLH	4.33

KRLQPTEINRS	4.33
AARTQGGKSQK	4.33
GMVKPLLFHKK	4.33
KKLKEAEKLFV	4.33
SPGGLSSAPSP	4.33
STFTNITYRGT	4.33
LAAWLLGRRNL	4.33
EKLEATINELV	4.33
KDLASPLIGRS	4.33
PNTWFQEILLL	4.33
RRKGGRRGRRL	4.33
PQPPEQLGLQA	4.33
TGYGRPRQIIS	4.33
PQPSTNIFGRY	4.33
LVEALKRKQQC	4.33
TKKLHFFPKSN	4.33
PDKSIINATDP	4.33
LERSVVRLGFG	4.33
ELSCSTDYRMR	4.33
KTLEKMEKHRK	4.33
WGQGTKVAVSS	4.33
QLDPIYVAYNM	4.33
GKLEYPTCAKR	4.33
PPTTATAFDHL	4.33
EPQPQREGCGC	4.33
RKYDTPKTKKN	4.33
AWNEKRRVYEE	4.33
GDEDEDDEWDD	4.33
RSQAKGRFTCG	4.33
IRVINGRAVEA	4.33
DHLALHMKRHI	4.33
IALPPTPAPTQ	4.33
GKRMQSLSLNK	4.33
DVDKWFHHQKN	4.33
SETNDTKVLRH	4.33
CQRLVELHTCR	4.33
EPYSESDDDWC	4.33
TNLQKILRRKD	4.33
GNHGHSMETSP	4.33
EDFGEEAEEEA	4.33
DEGPPGYPDRN	4.33
QHQSVHSEGKS	4.33
LLETTSGALSL	4.33
PAEKADEPMEH	4.33
FCFITFNQEEP	4.33
PKQEFLDIEDP	4.33
GPPYKCMWESL	4.33
GGRDSEEEMLA	4.33
PRIASNAGSIA	4.33
GDDEACSMDTN	4.33
PTYKYLEQMQI	4.33
AAENGFQEHEV	4.33
PSVPKVPKGRK	4.33
TTISQVAPGED	4.33
DEQSEYSDIRR	4.33
FEEEEEEEEEG	4.33
RRKRKTIQRHK	4.33
MFPRDPKRLTP	4.33
LSEAHNPAGHM	4.33
LIQHQRVHYRE	4.33
LLKKSDLQKDF	4.33
KKKSKTKCVIM	4.33
RGTKTVQEKEN	4.33
HDHHDEFCLMP	4.33
ELLDEHLKSAK	4.33
AVSKEMSKRSP	4.33
CTKEANITTPR	4.33
QNRIIFDSVGK	4.33
KKTCSVSEWII	4.33
RSDPNHSLQGI	4.33
ADCASLVPQCA	4.33
TYHENAALTGK	4.33
DASVKEWTITY	4.33
EKLQDEDLGFL	4.33
NKDDDIDIDAI	4.33
TELSPTLPHQL	4.33
KEELEEVIKDI	4.33
SGGNYRDNYDN	4.33
DFHILERASSQ	4.33
TPQLLQWLKEE	4.33
DSAQGSESHSL	4.33
SRDGKWRSSRH	4.33
KTLSKEEETKK	4.33
KFLPSELRDEH	4.33
ECKCSPRKCSK	4.33
QRNMTKLQLAL	4.33
LAGVGVPGLPV	4.33
ATADLPPTEVT	4.33
KRAAVAASSSS	4.33
KTFKRILHIRS	4.33
AEDTAVYYCAK	4.33
GQVVSSHKREI	4.33
QETGKKETIEE	4.33
MEKIAQSSLCN	4.33
LQQSMPPCEPP	4.33
ITAAVPEDCDT	4.33
PEQYSLAFAEV	4.33
LLKTMQSNNSF	4.33
LVLTYQPERKD	4.33
DARNPPVVSKM	4.33
KLKKQKLMARE	4.33
HRASHYQAHYT	4.33
QSTPCSSSSTA	4.33
KRQKTENGASA	4.33
SWRCTMKKASH	4.33
SDTEQSEDNNE	4.33
TLESTTVGSSV	4.33
SLAPSMETHNP	4.33
GHYELVMPQAN	4.33
GTQTPSPPSEK	4.33
NAEKLLNVVKV	4.33
LDKKLEDIALP	4.33
PFLAAAWSLHP	4.33
FVKPLTTSTNK	4.33
LQDVANDRGSH	4.33
VGMVPEWSDNS	4.33
GENLEVRWSKY	4.33
KGQQYDMVLDV	4.33
DHLALHMKRHF	4.33
RVMFARYKELD	4.33
SEKGTVQQADE	4.33
LAKYEARHGPL	4.33
EDAVHSGALND	4.33
VHQRMHTGEKP	4.33
CQPGAPSITFA	4.33
HSGEKSESISV	4.33
QDWVQKTIAEN	4.33
QEEYQEYEPEA	4.33
LMRHHRTHLVE	4.33
FGKGTTVTVSS	4.33
EGSVDPEGQGS	4.33
AISPDDSDEEN	4.33
IQAKTSWDPGF	4.33
EQLHGARPRRV	4.33
AYTDYIVPDIF	4.33
EDVHCYSMQSK	4.33
KWIEDTIAENS	4.33
PVVPVHLDRII	4.33

SYAAWIDSVLA	4.33
TATESQYQQQP	4.33
NKFGEDMPHPH	4.33
IFLLNKKFYGK	4.33
REEPSGDGELP	4.33
EQPVSEGGCSC	4.33
DPYPAAYAAAL	4.33
ARKTAGLSDLY	4.33
DILLSLFLNDT	4.33
INDKMHFSLKE	4.33
MLQIIQEKRGY	4.33
EVRAVLEKLHS	4.33
LQEELESLLEK	4.33
VSMFFTHTFPK	4.33
ELSRSAPAVEA	4.33
APNPDDMEEDP	4.33
SKRLTSLAQSY	4.33
LMTPNGPEVHG	4.33
PGRPNGGPRAL	4.33
SEEKEKGACGC	4.33
ITDFQILENQA	4.33
KKEKAKSCSIM	4.33
RRITQGFCVVT	4.33
NYVEILVALPH	4.33
MIEFKINPSRR	4.33
FFPKSVEDCHY	4.33
VPSYWKRPQMS	4.33
FENQDIHSASA	4.33
GINVKKPHYFT	4.33
TTNQTEFERVF	4.33
PGPSDTPILPQ	4.33
EEEKREEKLEQ	4.33
MDTLASHQLYI	4.33
QRGQGTMNPPH	4.33
FSSSLPYLMFL	4.33
PDTCRCRKLRR	4.33
TNLQERLRRKE	4.33
KPTMQSSRRCC	4.33
LIGILASLAER	4.33
WGQGTTVHVSS	4.33
PMKPNTQATPP	4.33
MGDASNHMGQM	4.33
NFLLSQNFDDE	4.33
LLNDLQANSLK	4.33
LVLAAGAYSPQ	4.33
STLCAIDLQFP	4.33
KDKKGCHCVIF	4.33
PPEAPKGKKKK	4.33
VSKYETKYGPL	4.33
CPNVPSRPQAM	4.33
TATEAETTRTR	4.33
LPLISSLQSSE	4.33
CQVVKADTYCA	4.33
CRRSSMKPGFV	4.33
GKEDAGKAFAS	4.33
ELCYAPTKDPK	4.33
PQQVWALELNQ	4.33
NFLLQQNFDED	4.33
PGEAGLGGQER	4.33
WVLPKPAQAVY	4.33
LDNIAYIMPGL	4.33
YKEETIEKMQE	4.33
TEGCSFRRKQH	4.33
QRYNSFISLTV	4.33
INADGVGDAKD	4.33
IDRVTSYLNAS	4.33
QQYNSFVSLSV	4.33
SPTSPVPVPVM	4.33
VDPGARMTALQ	4.33
EFEEEAEEEVA	4.33
VGFCEQEFEQT	4.33
PICVPCPGGRF	4.33
DSSYFSSDESD	4.33
TVLQNEDTKSG	4.33
AGRKPLRGMSI	4.33
FSGGTSLWQNI	4.33
SDSFPYEFFQV	4.33
SSREQVHQTTR	4.33
AAMRKAAAKKD	4.33
GKKDWDDDQND	4.33
YCDGRSERNLL	4.33
AAQGENAGEDP	4.33
EFRSKQAEEEF	4.33
ELGRPGEEDLD	4.33
GEFKQTSSFLV	4.33
FGQGTKVDLKR	4.33
LITVTGSSFLV	4.33
GKLVYPSCEEK	4.33
EGFPDKQPRKK	4.33
CRMDHKAEVAS	4.33
PPPPGMRPPRP	4.33
AMGHYVLAERE	4.32
PGLDPTPKVLE	4.32
DYRNDQRNRPY	4.32
GAGAGDDEEED	4.32
AGQPSDSLYSL	4.32
AHQRSHTREKL	4.32
PDDNSGWDIDF	4.32
FIGPTPCPSCG	4.32
RHSLLQTLYKV	4.32
LTYCFNKPEDK	4.32
ADGVEVKRPKY	4.32
AGATLSICSPK	4.32
GVEVEVTIADA	4.32
REGIVEYPRCE	4.32
HLLVDFLQSLS	4.32
HGLHSYEKRLY	4.32
SQQNKSQCCSN	4.32
RLRNYYYDVVN	4.32
TTEETSKPKDD	4.32
LVKRTRNRSKE	4.32
EEGLVQMLDPS	4.32
MEPTAARTRVP	4.32
AVDGPMDKKEE	4.32
HKDRAKSCNLM	4.32
LFQHWEGSIPT	4.32
KHQKLHTRDKS	4.32
SHLNKHKKIHT	4.32
APAAPHTGPEG	4.32
VVCKQVFKKVQ	4.32
TENSFTLDADF	4.32
ERTCRCDKPRR	4.32
ECNQCGKAFAQ	4.32
SPQKREKDRTK	4.32
HGSALHALSVP	4.32
ADGASQHVGME	4.32
RRVHARKEPSH	4.32
THLPTIPEMVP	4.32
DSRVQQALDGL	4.32
REIDVRIDRNA	4.32
ENRDECLMCGS	4.32
AYNDFFLEDNK	4.32
QHQKLHTAWMQ	4.32
GGASGLASPGC	4.32
VDWIQETMKNN	4.32
GQGKGGLKSPA	4.32
NHQRTHTVKKS	4.32
SDEDMGFGLFD	4.32

EEVDAMLAVKK	4.32
LGTPPGQGPQS	4.32
SSREHVQSRPL	4.32
LALKERGGRKK	4.32
HSLLLYKAFLK	4.32
TSEGQSKRCKP	4.32
VHTDYIVPNLF	4.32
GCTKRSLAKYC	4.32
DDIQTKRSFQY	4.32
KLMDVGLIAIR	4.32
HFRPAGLPEKY	4.32
ALEPGRPNGLL	4.32
FGQGTKLQITR	4.32
DVYKENLVDGF	4.32
AFSAAIDYYLR	4.32
PNARLRSEENE	4.32
FGQGTKVQIKR	4.32
SDTEQVEADLE	4.32
IGFSNLSKTLK	4.32
CHKYFEKAGLK	4.32
LYPLRKYAVKA	4.32
GSGGYGGRSRY	4.32
KKGGGCPCVLL	4.32
QRTHIGEKPYE	4.32
GPWPMHPAGMQ	4.32
VIMMVLIAHLE	4.32
AWRRLPQAFRP	4.32
IGDDDEDIDDI	4.32
REHERTHTINR	4.32
AWHRVHVYVSP	4.32
VDWIRMIMRNN	4.32
EGEGEGEEEEE	4.32
KSVGGACVLVA	4.32
RHKRNHMGEKS	4.32
LSQLTQHKKIH	4.32
IKSTERKVRQL	4.32
LTTPQTFSNIK	4.32
MVTKFYFQEDE	4.32
GVEISCWSVEL	4.32
TDTRLTFHFQF	4.32
QSKIHSGGKNL	4.32
KNKWFFQKLRF	4.32
EDMLLELLPDD	4.32
APDLKESGAAV	4.32
QKIAEKFSQRG	4.32
VDLTSLKAELG	4.32
RSRSRSVDSGN	4.32
VSVGPTYMRVS	4.32
DPGQLETVLEK	4.32
QKILDSGNKKN	4.32
TQENVDTILVE	4.32
PRSTNFFGFLS	4.32
PEGKHHSLSFS	4.32
KGQLTSVHSSS	4.32
TNWIQKTIQAK	4.32
FGGGTTVDIKR	4.32
LLKSGPFGKKY	4.32
LYKGKKERPRS	4.32
ELEDLQLSEDD	4.32
YNVYGIESVKI	4.32
YSGQNSMGGYD	4.32
ERRAAALAKIK	4.32
RDGDILGKYVD	4.32
DKKRKGQVIQF	4.32
RNFSLGSKPRN	4.32
EQVLNASRAKK	4.32
QAAIQELLNSA	4.32
VDVLRDHIHVH	4.32
LRSKDRDLGSS	4.32
SRSRSRSHSRS	4.32
ANQDPDSILFN	4.32
DEIDETTELSS	4.32
AAQNAFKGNQI	4.32
KRTQAPTKASE	4.32
TAPHCGHRWTE	4.32
ANARAKKLIDS	4.32
LNVPPLLISIR	4.32
TKELRKLKNQA	4.32
ECFLMELEQPA	4.32
NQAKKCECPSN	4.32
GGGGGYGGSSF	4.32
KLDTRIKTRKN	4.32
EFIVETKICQE	4.32
AEQPGVSPSGS	4.32
TTACHEFFEHE	4.32
EAMGEEPSRAE	4.32
PRCGPCNSFVR	4.32
GAAPRPPPKPM	4.32
GLRRGLPLLQP	4.32
LGASVIITNGR	4.32
AAGTKKQFQKF	4.32
GARARYQKSYR	4.32
HKKSHEESHKE	4.32
CQGIGMTNPNL	4.32
PSSIADLVFTY	4.32
TNESKIEIQKI	4.32
DVNFEFPEFQL	4.32
YVDGVEVHNAK	4.32
SSQPLLVHDDV	4.32
SMQLPPALPPQ	4.32
DWIKKIIGSKG	4.32
THEFERAPRSV	4.32
LVTVSGNETME	4.32
KSPEKNERHTC	4.32
TGEKPYSCKVC	4.32
RTQSPQNCSIM	4.32
NIKRKLDTYLQ	4.32
SRCGPCNTFGY	4.32
GGDVDDLLDMI	4.32
AKADGIVSKNF	4.32
KTIQGDEEDLR	4.32
KWINDTMKKHR	4.32
VYYCQQRSNWP	4.32
QILLQRNPATR	4.32
VKGIAPYSYAG	4.32
SQLSVQEDVKI	4.32
NEKCGKEFSEF	4.32
RQLAEIAESGL	4.32
QYHGRKGTVKQ	4.32
ASSSTCPLGGP	4.32
VVLRNPLIAGK	4.32
TEKCAKEFGEF	4.32
LWDIQKDLKDL	4.32
EAFLPEAKAIS	4.32
IFLSDQTKEKE	4.32
VQNLAPAETHT	4.32
SQSHSSSMISR	4.32
GHPFLRRNSGC	4.32
AVNGTVPLTHI	4.32
VTSWQTRDQYY	4.32
FVSTTYSGVTR	4.32
SLFPLPGLPDF	4.32
HKYLDSEEDEE	4.32
NKFAVETLICS	4.32
TTSSSQHSSTK	4.32
SDITQARVKSC	4.32
SILFYGRFSSP	4.32
KFFPYSSADAS	4.32

PRNRRHHPPDT	4.32
FGEGTRLEIKR	4.32
EGMDFQGPGES	4.32
EQLTNDKTPEA	4.32
EEEDEYSGGLC	4.32
YLDWIQEIMEN	4.32
LDKASQEPPLL	4.32
MGRGNIFQKRR	4.32
KHETYGHKTPY	4.32
PHHQDFQSYPK	4.32
ITKWNRNESYS	4.32
QYNEDRNPIST	4.32
VEIENPETSDQ	4.32
SAIAAVIARFY	4.32
PDFASSFKIVL	4.32
PDFLSTFHIRI	4.32
KDKDVAGQPQP	4.32
KIRRLSACKQQ	4.32
FDETTYEETED	4.32
DVFQQQTGGVY	4.32
KSPEEEGAVSS	4.32
KEAEVQAKQQA	4.32
QQEAPMDGFQL	4.32
FGQGTKLEIKR	4.32
MLHIWGKYTRS	4.32
RVDQIRDNMAG	4.32
FGQGTKLDIKR	4.32
SNSPEKPKVIQ	4.32
SAAGPGFSLKF	4.32
TFGGGTKVDNK	4.32
VEKTVAPTECS	4.32
HGAAPCSGGSQ	4.32
ESSISNLVFTY	4.32
ISRKPLAAKNR	4.32
GKYDCGHVQKK	4.32
GRGRGRGGPRR	4.32
QCTLTHAAGYP	4.32
GDDLTLHSFLP	4.32
YALNMLLQRCN	4.32
VPSPCPVFLQD	4.32
TQQQKWLKGPQ	4.32
FGQGTRLEIKR	4.32
AFNVMFSAPHI	4.32
YGQEGPLEGKI	4.32
LFPLHQETAAS	4.32
YTNIMRVLSIS	4.32
NEKCAKEFSEF	4.32
TSSSVNVSSNL	4.53
NVDFPPKESSL	4.53
PRYKLCVIPRS	4.53
QEVLPAATSIL	4.53
DMIQLMNSTHL	4.53
FGGGTRVTVLS	4.53
YKHLKSLQSKL	4.53
LGARVSKETPL	4.52
GSDVSLTACKV	4.52
SSKARSDDLSV	4.52
SHSATSVHTSV	4.52
QFVHPILQSAV	4.52
LRDYKQSSSTL	4.52
SPSVYSRSQYV	4.52
DTPPSTNCTHV	4.51
TVTVKISQKNS	4.51
GVLYPREGTEV	4.50
KLIWVRKIHSP	4.50
IPFRSRSPSDV	4.50
AQWVDSYYTSL	4.50
HSEDAGVTCTL	4.50
QFTTLLTSLAP	4.50
RTAFIKDQSAL	4.50
KINKTEICSQL	4.50
GPGKKPEDSRV	4.49
TFGSGTRLTVV	4.49
VREIGTVTYLM	4.49
KYRLIYLYCSF	4.49
FGTGTKVTVLR	4.49
FGGGTRLTVLS	4.49
PLPRRSTRLKT	4.48
YRPRRKTKTLL	4.48
STTWCRTCWCP	4.48
HFGDGTRLSIL	4.48
ESSAIQSISHV	4.48
HVYIKNRVSMI	4.48
VMGINHRFCCL	4.48
CDLFDVPVLNL	4.47
RRELCSRLQDQ	4.47
DLISWLCFSVL	4.47
PRPAPPSSNKV	4.47
TLYHRALYCEL	4.47
GIIYIHLRKRE	4.47
SSASVKLSFTS	4.47
RLQDLAPDTAL	4.47
LGPGDPFSTYV	4.47
GYLQAASVTLL	4.47
GLIAGEKETHL	4.47
YSTTVTLFKVK	4.46
IMTVTHFLPRI	4.46
IGKRGIIGYDV	4.46
QRDRYSHWTKL	4.46
HLRVSFSKSTI	4.46
LTVPFEKHSTL	4.46
QSFCTVLVSSI	4.46
LVAQFQKNSKL	4.46
YKDGAYENCQL	4.45
GVIYVILRKRE	4.45
VSTSRVRLFYV	4.45
MIQREDQETAV	4.45
AEMDRRPATVV	4.45
VEAHQSWRTDV	4.45
QAKKRMVESPL	4.45
VLVKILKKCSV	4.45
KLKFNFKTSLW	4.45
IGKIGTVMTFL	4.45
DDANRNYRSHL	4.44
TVFGKTLYCEL	4.44
VVLTSVTTVLP	4.44
VPFRSRSPSEV	4.44
PGGRSNITSLS	4.44
MLLLLLLNYNM	4.44
TTFSGYLLYRT	4.44
LFPENFTRRLD	4.44
PKTSAHHATFV	4.44
HYSKQDRCSDL	4.44
EMKFLGATETV	4.44
SHLQGNQLRRN	4.44
AARSAAASNYV	4.44
AKEKSPIQSQI	4.44
QLARRIRGERA	4.44
PPLDSQQHTEV	4.43
QLNGTFESQVQ	4.43
KEQIQKSTGAP	4.43
ARQHVVADTEL	4.43
SYIWVPAKEKL	4.43
YRRLLVQILQK	4.43
DNQARLSGSCL	4.43
FAPSRKLNTEI	4.37
DSSDLVEDSFL	4.37
TLKDICSPKTD	4.37

RFTEWIQSFLH	4.37
RMKVAWWRWTS	4.37
TVLFMGQVMEP	4.37
VQPSSLEMSAL	4.37
KVMDSDEDDDY	4.37
GPGAAAGPGID	4.37
GKRVTNQLSLS	4.37
KWTFEACRQIN	4.37
PAFSHGGFSHN	4.37
LAGAPMPLTHL	4.37
AVVVDITEHCH	4.37
FGTGTRLQVTL	4.37
IVLLGLSALLQ	4.37
LFRRRRRGFRQ	4.37
EKGILQPTLKV	4.37
SQRSCSQPSTA	4.37
QLHQLHSRLIS	4.37
TLRKKLVFPSD	4.37
PSLLERHCAYL	4.37
EHVQARRFSIN	4.37
TNAIKDALAAT	4.37
LLSDTEEDDKC	4.37
RFHAKFTLEYS	4.37
EPGPPGDPGLT	4.37
VRTRRFSKHLL	4.37
LRRRKRFQRHQ	4.37
SFGKYGRNAYV	4.37
LIGKKGQQKTV	4.37
TDSEKVGLVLQ	4.37
WARVGWIQLPG	4.37
CYDALRKMINY	4.37
NVRIFVSEYWF	4.37
PEFKQNGDTSL	4.37
IRRRPSAYQAL	4.37
HKPQGKKTKFE	4.37
QFCCRTCLLAG	4.37
KKIIKCKFCRQ	4.37
SKSLEPICNIL	4.37
VPRGTKMEATD	4.37
KLTAKWQRQGR	4.37
KATEDKAAKGK	4.37
LPVLGSQTGKI	4.37
LGTLVTMGLLT	4.37
PPSFRVMVSGL	4.37
FPAPPGEEPPA	4.37
FYSLGLSFASL	4.37
APRAEAEDSFL	4.37
SSCSTPRADRL	4.37
LPGVDALSNIL	4.37
NGFIVEETLPL	4.37
CAGGGRGAETQ	4.37
LCLVLSTRPHS	4.37
LQEYGTQGCQV	4.37
GPIPPAVNARL	4.37
LKTKRVTKKAQ	4.37
QSVTSERETNI	4.37
PVVLRVKSSDQ	4.37
KIVITDCGQLS	4.37
KLYWQGNSKLP	4.37
ASSPLKLNELN	4.37
TIKGTVRTFLS	4.37
VEKVKEFLSTL	4.37
KADKLAEEHSS	4.37
RFRSLVCFIPP	4.37
VNWIKEKTKLT	4.37
AVLWLRTHLGS	4.37
KLVTTVTEIAG	4.37
SGIEMSEVNAL	4.37
TTFLVDKYEIL	4.37
YRYYTSESKSS	4.37
IIKLGPPPKKA	4.37
MIQYYQNDIPY	4.37
MTKAGKFQVRT	4.37
SECCRFILNLL	4.37
FRVQKRNRTPQ	4.37
PRFHPSGGKTR	4.37
STSSLRSVCFK	4.37
KRLSTPSASTY	4.37
MEIATFRLRLG	4.37
EVSSVSSVSPA	4.37
KQDWSSCPDIF	4.37
FRDKRLFCVLL	4.37
ATAGSGVQPAQ	4.37
FMNKSLSGPGQ	4.37
LTLSTMDSSTC	4.37
VGHPTERCFVF	4.36
ELLDYWGSEVP	4.36
SLRKTPLKTAN	4.36
LTSKENLPYVL	4.36
PGGRANITSLG	4.36
MCPLLTFRAIN	4.36
PSPYELCAVPR	4.36
TGKTVSYLGLE	4.36
CQCYIGWCPFW	4.36
APHTEEEGAED	4.36
EPKNTSTYRVT	4.36
EGSHSVTASAL	4.36
FKKMIFCSRYW	4.36
FVAMMTSAWQL	4.36
CILFFGRFSSP	4.36
AACASYPIPLV	4.36
SNLSQSTLDIH	4.36
IRRFINSQFVL	4.36
SFIEQVAVSMT	4.36
GLAYLEETKPL	4.36
CVEMGDVESAF	4.36
RRRRRFTRQTG	4.36
KTLKSNSWFGC	4.36
KAERQLVRNKP	4.36
HLSSKRYTEEA	4.36
VEKFLKRAENS	4.36
PTDNSETVENT	4.36
LAVASFPKKQE	4.36
TEVGDKWIWLK	4.36
SYNSISSVVSR	4.36
TLHLVLRLRGG	4.36
ILPGVEALSNM	4.36
LLSDMYKSSDI	4.36
RLTIIVGPFSF	4.36
FGGGTKLTVLG	4.36
TASLTIWKKMG	4.36
PVVPVHLDTTI	4.36
QGFLNPLEFSA	4.36
STLLTMFGLPQ	4.36
EASATLNSIVA	4.36
ANQWIKFKSVS	4.36
VGEGDLLVELE	4.36
IVIDLPTPRNS	4.36
ISILNYKCRKV	4.36
RFLAEDALNTV	4.36
LAASFGKSINF	4.36
GVVCTRIYEKV	4.36
GSAPVDAGGYP	4.36
LMVAFLILFAM	4.36
KDQSAWKFQIL	4.36
YYVSKGLENID	4.36
ALLQSHPRAKL	4.36
GICHDAGRSKQ	4.36

LKKVEVPKLLQ	4.36
IDHSPKATPDN	4.36
GAAAGSTTSAP	4.36
QFGSEVELRHS	4.36
YILHAFSIYYH	4.36
LLIGASSRRSQ	4.36
KREMYSRLKKL	4.36
KSVRKRRVRKD	4.36
TSPLLTFRAIN	4.36
EEDYPQFGSPK	4.36
GQAVEVVTLEQ	4.36
EKLQNPNQSVV	4.36
KTHLPTFSQED	4.36
ITKYSTVQYSK	4.36
SLQWFLVCIRK	4.36
PNTYICMQRTV	4.36
RQFQLQHWLAI	4.36
YKRIQRKFAAC	4.36
QVRALLQAQQA	4.36
FLKYISCILIN	4.36
TLLNGDLQTSI	4.36
TILFYGRLCSP	4.36
SELDKSSAHSY	4.36
PFRRQLLQGRV	4.36
KSVLATEHAQT	4.36
ETLSVLKFTKS	4.36
SHMPSHLGTYY	4.36
PKSTQHSKKAH	4.36
LGIKQGEKHSA	4.36
VVSLSLLHLYS	4.36
ESTKRKLASAV	4.36
PGGRSNITSLG	4.35
VQISQSPTAMA	4.35
SEIDAELKNIL	4.35
SYRVLRHLAQP	4.35
LDHALNDMTSI	4.35
IAGHLLRSYRS	4.35
VTDQVNCPKLS	4.35
VGICLSIQSYY	4.35
GGLTQQVEKKE	4.35
DQVAILLFKSG	4.35
PPFYQLCFIPV	4.35
GTKLTVLSQPK	4.35
PCPPLQPVSVM	4.35
LTVTGKTVRLL	4.35
NNRSSGCCSGC	4.35
FRKAFLKILHC	4.35
PAPPTQQSSAE	4.35
YRRLLLAIVGQ	4.35
VAHQLQALRRG	4.35
IGLRISPLQFR	4.35
ESQTFFGLYKL	4.35
AESRSKVVSCP	4.35
EDVKPAKTVNI	4.35
YYLAYPLLSTA	4.35
ALLPRHRGRTA	4.35
SSMIYHCKHKL	4.35
NLLDNTRHGPA	4.35
ERLRRESVRPV	4.35
RLFNDIHKFRD	4.35
WEVLKPKLLQR	4.35
RNMVVRACGCH	4.35
GGHHSPSSLDG	4.35
HIDGREESFRI	4.35
FREKKFFCALL	4.35
VWVFRLYPSTS	4.35
CVLSRKAVRRA	4.35
TGTEETKQDDD	4.35
VCTLLSRTGRA	4.35
DWIQRVTGVRN	4.35
WGETAQAQFPG	4.35
EQRPYRRESEI	4.35
SSSDTSDSDSG	4.35
TKGRYSLDVWS	4.35
YCQNCFLKLFC	4.35
KAIIDFRNKQT	4.35
FPLILTLLLIT	4.35
DQPVYTTLTRP	4.35
KKHIFACTGFK	4.35
AMEIISEKPEF	4.35
LLLSLFSLACL	4.35
VLAGVALLLQI	4.35
GCSVKQAEDRF	4.35
DTISFTQTSQF	4.35
RELAEDDSILK	4.35
SHPNYPYSDEY	4.35
VQALSLYDGLV	4.35
SVIANQDPIAV	4.35
QPSSGRESPRH	4.35
KYVTTLLYKPI	4.35
AILGVQPDTED	4.35
QDDVLYALKDE	4.35
TSSQKCEFIHQ	4.35
LAEKSDRCSAC	4.35
ASLSFLFKALL	4.35
AMLDVKPDADE	4.35
FDIIKYLYDFL	4.35
IPCGDIRLNAV	4.35
FKTRLPRRARP	4.35
YVSVLPTTADF	4.35
QRAEGTFPGKI	4.35
LGPDGLPKPAA	4.35
ITYEQLKRLQI	4.35
RWNKDFGDTTV	4.35
YLKLLFLKKYS	4.35
ASTTTTASKAQ	4.35
LVMSDTAGTCY	4.35
YVASYVLEHLY	4.35
VALSVVIAARN	4.35
LYCQRSLLDKV	4.35
HGVKDIKWRPR	4.35
RKRSHAGYQTI	4.35
KKRRQINEDEP	4.35
SWNKRTRVSTK	4.35
PFPDLTSSGFL	4.35
RKEIFQLFWRN	4.35
PTVVSIGGGKG	4.35
EKTKRVSTKEI	4.35
DTRRICELLFS	4.35
IIFAIKKTLNI	4.35
SFAKKQQQQQS	4.35
NPEYLGLDVPV	4.35
RYCSGKSKKPV	4.35
LSPGPSPAVAR	4.35
KGTSDKCSCCA	4.35
EPPGNRSGAVW	4.35
IVSMYLYCNLQ	4.35
SPLVAAQPSDT	4.35
SSRSSSVDYIL	4.35
RQPDTPTELRA	4.35
VQRAVSVNPGK	4.35
FITLAEACTKR	4.35
YSFPISSILEW	4.35
LYSVGESDKET	4.35
CCAIRRNREMV	4.35
ANGAGGATNLQ	4.35
LLQFETQVLCH	4.35
AVFSFLFRAIV	4.35

YVRSLARNGMD	4.35
KGRVVKASFRA	4.35
IIRPAEPSLLD	4.35
LHLVLQETISP	4.35
VPKNHQAVRNP	4.35
CRHNPVFGVMS	4.35
YNFQSEHPYFG	4.35
CSCHEDGDGTP	4.35
SSSHMSVEEGS	4.35
RTKYRSLSSHS	4.35
PNAYSGELFDA	4.35
YHSDTPYYPSG	4.35
SYKIGKEMQNA	4.35
GSLEKRSQPGP	4.35
AKRQGHAEPQP	4.35
VGSLRRVIDFS	4.35
DVVYNAFSLRV	4.35
ELELKTTRGKM	4.35
LDRLEETVQAK	4.35
ARALEAAALAH	4.35
EKCLEKKQQSV	4.35
GRILGKVERID	4.35
PFSYNGTYRPV	4.35
RSLVKSRCQES	4.35
WGQGTPVTVSS	4.35
SREVHTKISAE	4.35
EWTDDDLVESL	4.35
EVEKPFAIAKE	4.35
IYLQEMMHSNQ	4.35
PQKLIYLRHYR	4.35
SFWLFRMPTSA	4.35
AALYGRLYERD	4.35
DPPLIENSVSI	4.35
HTRNYIPDLKK	4.35
LRMPNYKKKAT	4.35
RRFGPPGPEAW	4.35
SPEEIDVTSDD	4.35
LPLLVGGVASR	4.35
IVYTELPNAEP	4.35
LSSMPSASKTG	4.35
ERSLKSILNKN	4.35
GQQKEFGVGYI	4.35
SEEEGTVELSA	4.35
DVNETQPPQSE	4.35
VLYKYKLLPRS	4.35
WLLQKNPQLGH	4.35
GNPSEFFVDVM	4.35
AKESTAEKDEL	4.35
LYCKRSLQEWV	4.35
PNMVATECGCR	4.35
CRLEVKASAAH	4.35
DSIEDLGEVKK	4.35
TAYSCLPGKPK	4.35
DLKQMEEPCRF	4.35
RNELKKKASLF	4.35
EVLGCEAQDLY	4.35
RGGAGGGPSGD	4.35
TVYASVTLPES	4.35
PACCHCPDPHA	4.35
GGYSHDSRHRL	4.35
HFIYSSLFPVP	4.35
TPEMILSMMDR	4.35
FERESSGEEEE	4.35
FSSALYGESDL	4.35
TVKTDTSCHDL	4.35
SDVLFRYKVKS	4.35
ALSEFKAMDSI	4.35
GNHQSVLTFYS	4.35
FVVHMKQKGKK	4.35
IAPREPGAVSY	4.35
TSIRPCAAETQ	4.35
SCFSPYSFVSV	4.35
PPKGEDAEAHK	4.35
PEDRKVYTYVA	4.35
NVFRKALRACC	4.35
RPSVVENSVAI	4.35
KDSSEASDSAS	4.35
IPFDCWEPVQE	4.35
LTSEVTGMGTL	4.35
KFQCGFGLTIG	4.35
ASVAPGGQSGI	4.35
VNTMASSAVVD	4.35
SAVISLEGKPL	4.35
RSRHNGAISSK	4.35
GQVAILLFKSG	4.35
PSLANTTTPSP	4.35
YCGLQFRQHHH	4.35
KRRKGSKGPEG	4.35
ESAHDPGVVGT	4.35
GICRDFLESQQ	4.35
KDKKEVEIREE	4.35
NKAKHDELTYF	4.35
IATILETLYDL	4.35
ANSQSRMSTNM	4.35
AECKLEVRVPQ	4.35
PKSKKPDVKKN	4.35
KQASSQSWVPG	4.35
FGTGTKVIVLG	4.35
RTEQVPEKTEE	4.35
MSKQRGSRSGK	4.35
TMQENSTPRED	4.35
SRSSADSSRKH	4.35
FTNFFQNKPND	4.35
SPDRIPNSVAI	4.35
VRAFPDGPGLL	4.35
GLWWNPAPYQP	4.35
QQKQAPTWPSK	4.35
PRNGRRSAGFI	4.35
DINLDEDILDD	4.35
VLALLLASTLA	4.35
QGPSLEWLKKL	4.35
IVTNFLAGFEA	4.35
QHLRQQGSVEV	4.35
QARRRLAQFPG	4.35
LQARRQHTRKK	4.35
GKHYGNKKYRK	4.35
LQKLFSKRISS	4.35
LLTGQETPLYI	4.35
NQYLSILAAQK	4.35
FSCCPEDDNWT	4.35
DESFPACKIEL	4.35
KESAFEFLSSA	4.35
DGDGGALGHHV	4.35
LIQFLQNSRLI	4.35
SYSFSSSSIGH	4.35
QPPYTDYVSTR	4.35
SFKRNSIKKIV	4.35
PTGSSSTNPFL	4.35
LLRNVTLPEIF	4.35
WDYRREPPRPA	4.35
LRGEEVQEHAE	4.35
KGASEKCSCCD	4.35
SIKQAVRGIQV	4.35
LNIGSQGSTDY	4.35
EKIIGKVEKVD	4.35
FRRVLGQLAAA	4.35
IEYDFLEHPEC	4.35
CLGKACGRKRK	4.35

AQYETLSDSDD	4.35
DKARFRGIFSH	4.35
ERPSAKKRKTT	4.35
LHSCKTSVGKE	4.35
MAAKEAMKSNR	4.35
ISLSRLDLGSG	4.35
KVLLVLCGGDD	4.35
KTITGKTFSSR	4.35
EEALVTVPAHQ	4.35
IPPRTTTPYFL	4.35
EDEHNQNGNLD	4.35
GPPNYPFSDEY	4.35
FLAAKELYTKN	4.35
SRGRGGNESAM	4.35
ERRGESTMSAH	4.35
ALCRAVGGKFS	4.35
KKIGKTREELG	4.35
HSSDVLSPQMM	4.35
KAKAKKGKGKD	4.35
FKNSGKKSADH	4.35
TTYISYSNHSI	4.35
QPPGGSKVILF	4.35
ALNHTLSVEGF	4.35
ARRSPRHLGSG	4.35
GLDPLGYEIQL	4.35
SLEAYCHIRGF	4.35
TEIIYSEVKKQ	4.35
EEAMTQIIRVS	4.35
YEAFVKHIMSG	4.35
REVHTKIISEE	4.35
HAIVKEVTQSD	4.35
VICKRKNPFLL	4.35
SPSQTTLTNLF	4.35
KANSWFNCRKN	4.35
DIILSLFLNDM	4.35
IEKILNKGSTN	4.35
PVNDEPIVIED	4.35
NALMDGASGLM	4.35
PVPFFSLLQYE	4.35
YGSDPREELLY	4.35
LFNLIYWSIFS	4.35
WGQGTTVIVSS	4.35
LHERALREGEE	4.35
PSAGADSETHC	4.35
EPKDMTTFRSA	4.35
LLRAQRKFRPY	4.35
ALSSNFNEWYV	4.35
MIGVLVGVALI	4.35
SRQISSKKRPQ	4.35
LLADLPDLQDP	4.35
NTLGVDCIERK	4.35
LRTGNLFHRPA	4.35
LQPLLGNTGKS	4.35
PPHHGVQTPVQ	4.35
VKTDKWDFYCQ	4.35
KKQRKYGAEEC	4.35
EVELSVSTKKE	4.35
RDRTVQSPKSK	4.35
LLNGRDEEQYI	4.35
SLFLARVMNPV	4.35
YPKSNSSKEYV	4.35
VNAFSARTLVM	4.35
IFHKRAPEQAL	4.35
TVLSSMGLAAM	4.35
VFTSAQATTEA	4.35
TPGKPMREDTM	4.35
MPVPAAAGAQQ	4.35
IPNPFIQQQDA	4.35
NMGMDGQWHYM	4.35
QEAYRRFYGPV	4.35
RIVAEEFLKNF	4.35
QQMTDKCTTDE	4.35
ICEMTAFRFPD	4.35
DSRSGSPMARR	4.35
YRSPTDFMRHL	4.35
ILKHGAKDKDD	4.35
EQVSEEKTNEA	4.35
GSVHRTPSRQC	4.35
LLETDRVTAHF	4.35
QSTPCSTSSMA	4.35
KSTKIPESEDL	4.35
HNIKIIRNGPC	4.35
REDTLLTLTPC	4.35
SQQELSPVSSF	4.35
IDKLFLDTLPF	4.35
RVMPANSILWC	4.35
PCHLYDYRFQG	4.35
VTLGLWMVTAR	4.35
AGEEQAAKKKD	4.35
HPRRSRPSTAH	4.35
SLLEACTFRRP	4.35
FQQLQELTKTP	4.35
PGGIDRFVSAM	4.35
SMVAYKIPVND	4.35
NAPCSKDLGNA	4.34
LCHILGDFQRE	4.34
LESLNKDKKHH	4.34
EGHKLPVPGYN	4.34
SELDVFDAYLE	4.34
QTINYGRDDEK	4.34
NLQLQPGNAKL	4.34
PAPGGGADDPV	4.34
EVIERTALKDE	4.34
GAVSINSCPAA	4.34
RMQRKVLYRLK	4.34
DVEDDPDEMAV	4.34
AAAENKVAVKG	4.34
NSERKKRGARR	4.34
HTGAGEGGLWG	4.34
ALEEPDDQNRI	4.34
VCKQQPSRVSC	4.34
GEKLYLVRALQ	4.34
KSKIFDNESAA	4.34
AAKDPSPSVQG	4.34
NIGAAGFKMSP	4.34
GPTPASAEQNE	4.34
AWAFMRYRQQL	4.34
EGQYSGFKSPY	4.34
LTSVRQWVRKT	4.34
ACSCRVLSLNC	4.34
PPAKKAISELP	4.34
WILPFSPIIQK	4.34
VENRKSATKKN	4.34
LEGWGRGVYIR	4.34
AVSDELIKLYL	4.34
KVGEKVFPIWR	4.34
HSHYSDSDEAS	4.34
EEESYNPDKVT	4.34
DSYDLGDLLIN	4.34
PVSQPSLVGSK	4.34
LKPKRLGAHTP	4.34
LNIGDVQLEKP	4.34
ESLPPPPAELL	4.34
RGGSGGFGDEC	4.34
TADERAPLIRT	4.34
SVSAINLFVAL	4.34
CSVDPQCLQEL	4.34
ASLGACGFSYG	4.34

SDDNKINNLLQ	4.34
DEVLYEDSSTA	4.34
NKIINLMRKYQ	4.34
NGRVLYYATCK	4.34
NIIFFGKFCSP	4.34
RATSSSSSQPM	4.34
PDSRDLDLSDC	4.34
SSFSGYLLYPM	4.34
MREVNTFVKNL	4.34
FYSFLSSYAAG	4.34
NMGMEGQWHYM	4.34
PQPDSTSSGWF	4.34
CYRFYSTRGTL	4.34
RIQYQKRQGSS	4.34
LQQQDSRISLR	4.34
QRNRPWPKDSY	4.34
NGEDIDVTSND	4.34
ALLVAIFLIFL	4.34
AARKEALQQKQ	4.34
QSRKKLRMEAS	4.34
TVNGAPVPPLT	4.34
ASIHSFELDLL	4.34
QNGNDNENNRK	4.34
GVSHRARPPPA	4.34
LLEPYKPPSAQ	4.34
EEALDTVPAHY	4.34
PEPETQAPPRS	4.34
SGKSIRTILMF	4.34
KDQVNTVGIPI	4.34
DHLALHMKRHL	4.34
DQTSSWKFQVL	4.34
MEVVPPEEDPE	4.34
VQSQGTGTAQD	4.34
RPKGDKMRDEL	4.34
TATEGQYQQQP	4.34
ARFQPGVLEAP	4.34
HCRRLGRPALS	4.34
YKKIIKKLLES	4.34
VESRDGTVSGA	4.34
CECRLEVRVPQ	4.34
SQQEAAAKKFF	4.34
QEGKDEKAKGK	4.34
PGGGGSGGDPT	4.34
AYASGCTISPY	4.34
KAAKEAAAATS	4.34
LGLQAGATAPG	4.34
LYGEIESERTT	4.34
FKRSLLEMATF	4.34
LLHPFFKDMSL	4.34
DTAKFQGLGLN	4.34
WLGPILGYDTD	4.34
LRPHPLGIPTN	4.34
VTSLLQGSPHL	4.34
LVRGPPTLEYG	4.34
PQKCSEPQSSK	4.34
LCWCLREDDPQ	4.34
ENMGSKFDNIA	4.34
TRALRASESGI	4.34
SRQNWPKGENQ	4.34
QAPAQDPGHME	4.34
CLAETERNARS	4.34
VVAFFCQLQWS	4.34
PAATLLRRSKT	4.34
LQCDPSSRSQF	4.34
KVIKTEPEDSR	4.34
YKGEDCSPCTA	4.34
YACGDEDLEPL	4.34
INNRVVRASFK	4.34
QASADEYSYVA	4.34
LHEWVLREGEE	4.34
GSGGFGSTGKN	4.34
ENLPIPQRNTN	4.34
FPPPVSPRLNL	4.34
PRDPDPASRTH	4.34
PSPQDLLLVEH	4.34
PAVVQKTASGQ	4.34
WPKEAMNVQTV	4.34
NRFGTFVAALT	4.34
TENDSPTNVQQ	4.34
KCGKLQSVDEE	4.34
NALLSLVGSDP	4.34
ETLHSLQTAFT	4.34
VAPQSSEFIGA	4.34
ALRHALELEYL	4.34
DGDSTASPSSE	4.34
LHQHRKTVCVC	4.34
HIANLGVISQN	4.34
LAHKRGLLVRV	4.34
DKGKDIRESKK	4.34
ESASSPPVITE	4.34
ATDKTKQEEVD	4.34
WHQRGAFHPSN	4.34
GELSQGVQKMT	4.34
AGSENTKDIRL	4.34
LESALKDLKIK	4.34
CGKVSCVTPNF	4.34
DHPQLSGLLFL	4.34
NAASAQKATAE	4.34
LIPVHIGNANK	4.34
ERKKTMELTTR	4.34
SRSLSQSNVAS	4.34
SKAKASSSSHA	4.34
NSVKGCGNCSC	4.34
AKPCESQLCPL	4.34
GEQSSETSDSD	4.34
TTYFLNGGPSS	4.34
LLLNVVVMLAV	4.34
SSMSEGDEDEK	4.34
VFQFLREEETF	4.34
GFPYANYKRKI	4.34
TLGMAEEDEEE	4.34
RLAIQFGNYKK	4.34
LCCGIRNSSVY	4.34
GQGAGALVHSE	4.34
QSVDIAAFNKI	4.34
FSEDEAQDMEL	4.34
KGKKLSLPMPI	4.34
AKPPHKQKKAQ	4.34
NLGRGSSAPSQ	4.34
AAAATASSLLI	4.34
KLKSFPNIGSL	4.34
SGLPAHPEVPD	4.34
MMILRLVLLLL	4.34
RKQGAGAPTAP	4.34
GLTWLTSNYKS	4.34
PSGGADKPLKG	4.34
VEQKMSIRFLH	4.34
LIAYLKKATNE	4.34
GSCGMACFVPQ	4.34
SLEAYCHIKGF	4.34
LAYPVYAQQRR	4.34
AKEQIKWSLLR	4.34
LCQYRNIGTAR	4.34
PQFSYSASGRE	4.34
QPKIVKWDRDM	4.34
YVITHGEEKEE	4.34
LGERKGPPGLL	4.34
LPGPLQDMFKK	4.34

SLLSPQLCPAA	4.34
SLGPALLWAGP	4.34
EYDQRQLQAWP	4.34
NFRSSNKYSSS	4.34
HSFVKVCTDEE	4.34
QKPLMDMAPQQ	4.34
PGSGAVEMESV	4.34
PTETHSLSWDN	4.34
ILEYKRRNGLE	4.34
MLWQLDLSPGC	4.34
IFGSGTRLSIR	4.34
SGLGCKVLRRH	4.34
FKKLMKQGGAA	4.34
NAALQNGIFCN	4.34
RLQYFSGNPLL	4.34
NRGEKVILFQY	4.34
PRTRLEDPAMW	4.34
CVRKSQETAFE	4.34
LRKKYRDIERL	4.34
RLWEASRIPLL	4.34
EDENEGEDYAE	4.34
EASAGPLGCCR	4.34
SAVWGQGTLVT	4.34
LEAVRSPSFEK	4.34
NESSLSAKSEL	4.34
LLNPSSRCQLW	4.34
LLTHHFAQLMY	4.34
LYVEERAHKGS	4.34
GAEEFQEMVHS	4.34
DYSPQLQKPKF	4.34
LLGERGSSTRG	4.34
VDPSLLLHMLE	4.34
RGNKWLNCRYG	4.34
TRNHSTAYLTK	4.34
MEMLETPLQIT	4.34
ETQSKNGPVPV	4.34
SSLLHQPKARP	4.34
HSEDYSKVPKY	4.34
VTSGDPELPQV	4.34
DADLSQEPHLF	4.34
LHEWAFREGEE	4.34
SIGYSDLFCLS	4.34
VVLREQALALY	4.34
EPQWVPVSWVY	4.34
CGAIPCNTTRG	4.34
KPKPATSQATP	4.34
CDLFDVQILNY	4.34
DSLNSPTLLAL	4.34
PGGTGSSWGGQ	4.34
TVMGLKRAHQH	4.34
MFLKCIVETST	4.34
SVGASRHQGPL	4.34
YQMGDCCKEEI	4.34
VITFKQKIFMS	4.34
LYPERALAGHP	4.34
LSEMLPQHAAK	4.34
YHRLQSKVTAK	4.34
LLSTMDSPAST	4.34
KVGLQFLQRQC	4.34
KKGEDFVKTLK	4.34
RQRISELGARA	4.34
SHGCESHPITF	4.34
GDTLEKFVEGL	4.34
GSSFLAGEHPG	4.34
AQLANGGLKRR	4.34
HLSALLDMVDI	4.34
KEYLLKMATEE	4.34
LQMILKDYAKL	4.34
FSGSVEVIEVS	4.34
TREEFLTLIRS	4.34
MCFRLLQSVVI	4.34
LDWLSHELSKR	4.34
IQVIEEADEEE	4.34
EFAEDIFLNGC	4.34
SILFFGRFSSP	4.34
PYILKRDSYYY	4.34
NRLQNMEVTDA	4.34
MVTYDIKLSKM	4.34
TLLLLGASAAP	4.34
ICIEGRSNVGK	4.34
KEHTANISWAM	4.34
TKQRIDEFEAL	4.34
EEVSPPQTKDQ	4.34
ENVDLQRPNGL	4.34
DSELTSGLLAT	4.34
SAQGSDVSLTA	4.34
VVHQETEIADE	4.34
SSLREMETFVS	4.34
EVPGRRPLLHS	4.34
SAHFLPMVVHS	4.34
DIVSMISEDKK	4.34
YKSCAHDWVYE	4.34
KRTSSPHKEES	4.34
GNIAESLESLV	4.34
EKPPQTEHIEL	4.34
AGVQPALGVGL	4.34
MKSEEITALTN	4.34
STDKTKREEVD	4.34
TIPVLWTLLGS	4.34
SIVAWTGMLIA	4.34
LALMLQLWLLG	4.34
VISNDKRSFCH	4.34
ASSGLQSVVHR	4.34
MYEELNAFFHN	4.34
GDEGDAGEGEN	4.34
NYKILQADQEL	4.34
LFKWLQLTMFA	4.34
KIFEMGPVFTL	4.34
GHSTYPSHQYQ	4.34
MSAQLNAIFHD	4.34
NGHSMKQEMAM	4.34
SQPVRAAASQD	4.34
PARLDGQGLAS	4.34
EDVPGQAKDEL	4.34
SVEEEPWKREK	4.34
GVYRPPPKVKN	4.34
STHMDQQFFQT	4.34
LMGEQFLVSWC	4.34
LQILWEAARHL	4.34
GSDESLIASKA	4.34
KWDYEKNEWKK	4.34
LDDSDSLGDSM	4.34
TKAVTKYTSAK	4.34
FSESTHLVQHW	4.34
TANLVKEDSEV	4.34
YSRTEGSLDGT	4.34
EALQDVEDENQ	4.34
ALHQARGPAGQ	4.34
DKKATVYEVVY	4.34
VAREHIDKYKN	4.34
FLLFGADVVYK	4.34
IPWVQKILAAN	4.34
DPLEILEPDVQ	4.34
HRSKCGMCCKT	4.34
PQKSYLVGFSV	4.34
KLFLEMLEAKV	4.34
HAHVHKCCVVQ	4.34
YDSIWCNMKSN	4.34

STAPTPSTAAV	4.34
NLGHTPFVDEL	4.34
ARQSIDVGKTS	4.34
ALHHALELEYL	4.34
IRELTANLTFS	4.34
LCHHPIVEEPY	4.34
QVTRDLIVAEA	4.34
SLNGGSRETGL	4.34
SPQWVPVSWVY	4.34
WKKENNKDKLP	4.34
ESLDLGRSDGL	4.34
LAVTGKKTTRP	4.34
FMSKVTNPKQA	4.34
GLSVDRLVNGE	4.34
EDALREEGEGV	4.34
CSVDFECLQNN	4.34
ALGAAQATAKA	4.34
LKNRQIKASFK	4.34
QELQSIRKRHR	4.34
DKGASANQEKG	4.34
SLQASSEKTQQ	4.34
ALHQALELEYL	4.34
VTCTRIYEKVE	4.34
PGFASGLERYL	4.34
KYYPGWVAGSW	4.34
ETPEEKQTTIA	4.34
CAEGSQEECDK	4.34
QLNIELEAAHH	4.34
PYNGQSYPGSG	4.34
SGPASETLDCS	4.34
THVPSHLGSYY	4.34
FHPLGMSGAGS	4.34
QADKKIRECNL	4.34
HPKYKHDYVSH	4.34
DEFLEFMKGVE	4.34
KATCLCDTEIK	4.34
VHKHSRLLRQN	4.34
ITRGEHRFHMS	4.34
ELQGQDPTHLV	4.34
TICPVGGQGPD	4.34
VYSVDPYNPAS	4.34
FNIVYWLYYVN	4.34
YKKIIKEHLES	4.34
QTNEENSRVLP	4.34
VLLIENADDLQ	4.34
NGGKVVNQDST	4.34
GSWDSFLKIWN	4.34
RRFFKAKKLIE	4.34
YLEESDEDDLF	4.34
EQELLDFTNWF	4.34
NFVAAQERDQK	4.34
CQEQLDILRDK	4.34
GLYSRSLLNAS	4.34
FIDFCTMAECS	4.34
ELHELAQFGII	4.34
PPPLDQEEMES	4.34
SSFPFGSGVQT	4.34
HLGGAKQAGDV	4.34
YQTRGIAAWFT	4.34
IQNNLKYIGLC	4.34
TRYIAVSFVDP	4.34
RLHVDGVPHGR	4.34
SPPAETELQAM	4.34
QAGANTRPCPS	4.34
SILLLLLGPLF	4.34
CPGRKVQTQAC	4.34
EEMPLNVADLI	4.34
YISYLNHVLDV	4.34
KLAHVKHKEQI	4.34
LGTGYDRKDYV	4.34
EKERKQEEGES	4.34
SYLHKDEGNPE	4.34
HLKPLGPGSGK	4.34
AKAKELATKLG	4.34
FIGKVVNPTQK	4.34
QRVNVQPELVS	4.34
CHLCGDAVPRR	4.34
SRCQVCMKKRH	4.34
YRPAEVAETGA	4.34
HLGDVLNFLPL	4.34
RGGRQGYHHEQ	4.34
ELEYLGPDEND	4.34
EVNAILKALPQ	4.34
DQQDEEAGEGN	4.34
KNLDTKKYCQS	4.34
SDPAESMESLV	4.34
AGQFEDADVDH	4.34
GYRQQQSQTAY	4.34
DGGQHSSIHPA	4.34
EQIISLVSSDS	4.34
KQKRMKFSATY	4.34
AEPQYQPGDQT	4.34
WGRGTPVTVSS	4.34
FTLRFGVDRPS	4.34
AKTKEAGDGQP	4.34
SGSGPTIEEVD	4.34
KGPTISLTQIV	4.34
NSVVLYSTPPI	4.34
LKEQIMAFASK	4.34
AMIAKMNRQRT	4.34
IQEWLQLTMYS	4.34
DSVWTPSNKPI	4.34
GLARKIYKRRK	4.34
PNLLTQHCACI	4.34
PDMVVEACGCS	4.34
FNKEFLSKPKA	4.34
PIKRGRSCILL	4.34
FMEDGLEGMMF	4.34
LVAHECPNYFR	4.34
EKVKLGMTNSH	4.34
FDVWGQGTKVS	4.34
ARLWGGTLLWT	4.34
LVLYDEIKKYT	4.34
STMDHDLHDAL	4.34
EDMVVESCGCR	4.34
GYVIIKPLVWV	4.34
WGKGTTVTVSS	4.34
RDFYDSAGKQH	4.34
TRYIAVSFIDP	4.34
SVCQDIKPCVM	4.34
EMEGCIPQKQD	4.34
GSNINLEADES	4.34
GGENNEISETR	4.34
KLVPVHLDSIL	4.34
GQPQPGFGYSM	4.34
RILSTMDSPST	4.34
QPLRKDKDKKN	4.34
MLRNLILPKRR	4.34
GVIQHKEKCNQ	4.34
VTYQDIKPCVM	4.34
PVSTTAVEGAE	4.34
LVMLWSIWQYA	4.34
EHTKIEEEDVE	4.34
VPKHGDRFFTI	4.34
LLRRQAQQAGK	4.34
HTTPITSRVAR	4.34
DHPEYPFHDEY	4.34
YSYNVQDKRFI	4.34

TLRKKTKSKRS	4.34
YNAISAKTLKL	4.34
KLITEPAEIMA	4.34
FLRKKLFFKTS	4.34
RRLQHRPWCIH	4.34
GHGKEKPENSS	4.34
FQKLIQLLLSP	4.34
ICKAVAMLWKL	4.34
LAAYRPAVHPR	4.34
GSVNMREKPFC	4.34
LFLEMLEAMMD	4.34
DSGIHIILRTE	4.34
VAYSGENTFGF	4.34
CLRNVHKVTTC	4.34
ATEIEKSISAR	4.34
STAEPELSIVF	4.34
NFGAIGFFWVE	4.34
TPVKQSGGGCC	4.34
CTATQSFSTFR	4.34
PQGPPQGQSPQ	4.34
FKFGRTEDLWQ	4.34
SEDDDKKDDKN	4.34
NKENSAVPVEE	4.34
IEGDKSYFNAG	4.34
HFMQAEEGNPF	4.34
QTVRSIAAWFT	4.34
IVNVFAIAIVL	4.34
KRKKNDIYGED	4.34
LTQALANGPEA	4.34
FKHRLQCMTSQ	4.34
APLVFDFDGHE	4.34
AVCTRTYEKEA	4.34
NDWVPSITLPE	4.34
RSAAKVLTSMC	4.34
EYNVQKAAGLI	4.34
QYTKVWQCNCK	4.34
LISLKQAPLVH	4.34
MDAFMHEKNED	4.34
LSWYSFILLVL	4.34
RSFHTSRNPSR	4.34
PTSSEELVTLR	4.34
PALKMWLHSYH	4.34
VQDLAKAVAKV	4.34
LIYSEMKYMVH	4.34
FMGKVVNPTQK	4.34
RRIRGLEEGLG	4.34
GYILQAKRRGS	4.34
YIATAYIVSST	4.34
CGAKRCRRFLN	4.34
FEHIREGKFEE	4.34
KEIENTYANVA	4.34
PGDLPLDNQRT	4.34
EYDSLYPEDDL	4.34
AGRLIELNQQG	4.34
YSHMSASVPPQ	4.34
SSFNWPYMAIQ	4.34
LVFDVELLKLE	4.34
TYLEKAIKIHN	4.34
LARKLAVLTPG	4.34
RGYRSNYLYDN	4.34
PFTFKWWHMVP	4.34
CFRKQYEDQLS	4.34
PRNFRSPAGGG	4.34
GEEEEVAAVAE	4.34
SGHDPRAQGTL	4.34
FDVFGQGTLVT	4.34
SACQYAVDRPV	4.34
VTGHFTFTLYR	4.34
EGLDFNFEPDP	4.34
INGESGQAKRN	4.34
FNVVYWLYYVH	4.34
RKEILRHGNSQ	4.34
NNTLSFIVIEA	4.34
MALLATVLGRF	4.34
FPEEEEGGCEL	4.34
YVRTVYNIFRP	4.34
PPMKEFPDQHH	4.34
LIIKKVERKNF	4.34
TQLHAAESDEF	4.34
KIFQERLRRKE	4.34
LRRARGRKRKT	4.34
ESKLSFATCKS	4.34
KQNDVFGEAEQ	4.34
IVITKGHFAMV	4.34
ATASLQTEKDN	4.34
RTHYSQLRKKS	4.34
KWIKNTIAANS	4.34
QGQAPSVFSVC	4.34
EVVTFPGNPNT	4.34
LTDYYRSLEKN	4.34
KSTMGKPQRLY	4.34
QRMHLRQYELL	4.34
KPRGETKGMIY	4.34
ELYLQSLTAEH	4.34
PEIHENYRING	4.34
WGQGTWSPSLQ	4.34
DDDEEEEDEEE	4.34
ALPSDFKTILD	4.34
LKKAELKAKLL	4.34
AQPLGERDGDF	4.34
NFLQSRLLPRV	4.34
SEFYPRDSAKH	4.34
YLIPQIPHSHY	4.34
TYSKQVTSQEP	4.34
DSYDSYATHNE	4.34
QAFSICLSSFN	4.34
FIIALALGSRK	4.34
PTYKFFEQMQN	4.34
MEILHPGKCLA	4.34
VYEDLEGWFQA	4.34
HKLGLGLEFQA	4.34
AIDMADEDYEF	4.34
PIFIADAFNVR	4.34
CSERTEMYTCK	4.34
NLVYWVSYLYL	4.34
GTGGQVACAES	4.34
ALNLEKDSDLE	4.34
QKAAAEFGAHY	4.34
GPGSAILEMKK	4.34
RNYVTPVNRNV	4.34
PAWDPMGALAE	4.34
EPVIKGAASPK	4.34
IKNNLKDCGLF	4.34
SGKKPIDWKEL	4.34
GGQRPGGKRRK	4.34
DVQADREDSRE	4.34
LSTCAAAAGTQ	4.34
AKASSAGVLVS	4.34
FAFEGIGDEDL	4.34
LTVTFKEYVTV	4.34
KATPTHFSHWP	4.34
YLLYYRRVDLL	4.34
ILEVVPEEDKL	4.34
VWYMDYDAFLG	4.34
LEKALNKRFKM	4.34
HHPSSMVTAMG	4.33
QLLLGVKGHAF	4.33
CESMTDVHTCK	4.33

DYLSSISESIQ	4.33
YLDLKSDEWEY	4.33
TQKSLSLSLGK	4.33
KESKQEHKDVM	4.33
SLEESRKIFRF	4.33
PRPAQGQQPPQ	4.33
TFCQSIISTLT	4.33
LQSCIDLFKNN	4.33
AVLIVAKKCPS	4.33
SGAFVYDCSKF	4.33
PGMVVDRCGCS	4.33
TNLQKRLRRKD	4.33
HLLLVDPEGVV	4.33
IIVAVKCMLLN	4.33
EALRSLQQLQH	4.33
WNLSIKKEWTE	4.33
TIEQEKRSEIS	4.33
KPGPALKTNLF	4.33
FSDLKYECRDP	4.33
GQDIIDLQTEV	4.33
FKVAQTLCFPS	4.33
EGLVLQAYGNS	4.33
KQRLATFFPRK	4.33
RRITITECGQI	4.33
WIQKVIDQFGE	4.33
EHQAKAGSDKL	4.33
GGQGEALLNTT	4.33
TGLIHPSVAWR	4.33
AQSLYIANHAY	4.33
LMSLKQTPLSR	4.33
IATEDKSPVDT	4.33
ACVIDDRSPDT	4.33
PSEKEVEPKQC	4.33
KIILTYKVPQS	4.33
KPNKLQCCQNL	4.33
PPSTRKKVTIK	4.33
EETKEDAEEKQ	4.33
QNSTSSHLGSS	4.33
AFWKYQRPEGL	4.33
EEICRRVKDLD	4.33
QNMIVEECGCS	4.33
YANTLLQLGMM	4.33
VDEFSTLVAES	4.33
DSLSSPTLLAL	4.33
TKAKKAAPKKK	4.33
DLLHQYREANQ	4.33
LHSDHSAPSSK	4.33
FINRLTGYLRN	4.33
ANNQVGLAPVA	4.33
LPNKLKFGGWI	4.33
AKQIVQKSLGL	4.33
ESDINTFIYMA	4.33
GGAAHKPGRKP	4.33
LLSPQAQVEDS	4.33
LSTLLSLTETS	4.33
FRKLIRERALG	4.33
LYTRVAVWGNK	4.33
RLKGDKMRDEL	4.33
FLKQESFPTSE	4.33
WGQGTTVTVSS	4.33
VQDEAMRTLAS	4.33
TGNIRPVDMEI	4.33
TPEEDEGQSQP	4.33
INGNICHERYV	4.33
FRRPLPKKPKK	4.33
IPALDNFLDKL	4.33
IAKNLRGCGLY	4.33
GVAVLDNPYPV	4.33
MKALIENVSDS	4.33
DDEKDGDKIAI	4.33
LGGRQMHWPPG	4.33
HGWHGGRRLPE	4.33
VILRKNPNYDL	4.33
LSLSRTPADGR	4.33
VSFKTSKQHKA	4.33
GERRPAYLPQY	4.33
SEDTAVYYCAR	4.33
AREGQQSFHLS	4.33
CGKSCVSPVKA	4.33
SSLVKAMGNCA	4.33
YTSKKESCVGL	4.33
PNWATQDSGFY	4.33
QWVPYISLQER	4.33
FDQRDLADEPS	4.33
LGGSREARGSV	4.33
LSSFTSYENPT	4.33
YKKAPTNEFYA	4.33
SQLSQQEGIKM	4.33
IAREWTQKYAM	4.33
APGPSTSTITI	4.33
ILFLVMQREWR	4.33
KQDTITTKKYI	4.33
ASGGPTIEEVD	4.33
DLMKPDVTNLG	4.33
VLKLLEPALSQ	4.33
DPLNSPSLLAL	4.33
PTWEGYDPSWN	4.33
QNQYTKTNDVR	4.33
MTRYFYLFPGN	4.33
VEETVQAMEVE	4.33
GGVTGWKSKCC	4.33
IKAMLSIDVAE	4.33
SSGDSSDSDSN	4.33
LAKTGKKKKRD	4.33
TLRAENKSQCC	4.33
RPDSWCALALA	4.33
DLRKSGEAAGY	4.33
EKKINRDRLQY	4.33
DVANLENESKV	4.33
QGVLVTVSSGS	4.33
FGKKKGPNANS	4.33
RALPLPQSSTC	4.33
GESASILGAVT	4.33
AGQSVLLQLPQ	4.33
QKDLQEKISGK	4.33
RGRRASRGYKN	4.33
ENVLDFNGCTL	4.33
TQSLFTACYTY	4.33
MTVEVSDWTVP	4.33
QAPPVYLDVLG	4.33
LPIFNRISVSE	4.33
VKSIRSGYEVM	4.33
QRMHLKQYELL	4.33
VTYERVSKRLA	4.33
NCPSPQAAVQQ	4.33
IREETVSLRKD	4.33
MELLECVSKCA	4.33
FKKHCSYQDIL	4.33
LDNISFPKQGS	4.33
VLLVPGPEKQN	4.33
RTASHPVSASE	4.33
TALTPDACYPD	4.33
RRRLSVLQDLD	4.33
LAYEQVAKALK	4.33
ENSSDFQSNIA	4.33
QTAPQYSPKIN	4.33
RRQAALGVKRK	4.33
WGQGTLLIVSS	4.33

QEANSKTSCPC	4.33
DSSNASSEPSS	4.33
VKPKKAAPKKK	4.33
QLSAAACGCGG	4.33
DPDSYHRRDFW	4.33
HLHNNHRRGNL	4.33
GSSSCPTGSSG	4.33
VQDLPRLSIAM	4.33
GGASAMLIQKL	4.33
QERIAHQRMGD	4.33
APKKAEWLDQT	4.33
CVQEISDVVQR	4.33
SAQAPPPAAAK	4.33
PDIKRLLKVAI	4.33
QAMLLRIARIP	4.33
KDSGCSSPGAH	4.33
IVVVIVIYFFT	4.33
KLKLISSDSED	4.33
PHQLYGVVPQA	4.33
LARYLDEINLL	4.33
RGGGTEPGGRS	4.33
PLVQRGSANGL	4.33
RGPSPPPMAGG	4.33
KRIPLTRTTTF	4.33
PEFVDIINAKQ	4.33
GVPGAWPGLPV	4.33
PEDGTALCFIF	4.33
KDCPSEPPCSQ	4.33
YSRALYLALLK	4.33
SAIVEQSWNDS	4.33
ETPDCFWKYCV	4.33
AKTQNQKQRKK	4.33
VFMGRVYDPRA	4.33
ANLQERLRRKE	4.33
ITTRTLKARMD	4.33
EPPEPFEYIDD	4.33
DFFFFPFLSAA	4.33
FYNFADIQMGI	4.33
GRSPPGTSGST	4.33
ISVVMLLPGIW	4.33
EGLEDYFSEDS	4.33
KATPIYLDILG	4.33
GTGGQVACAEN	4.33
KPTMQASRRCC	4.33
VFYTVWKKAKP	4.33
LAFSAKSHIQA	4.33
NECFQFKQMYG	4.33
LFEKLYLLTEK	4.33
GIVEPSQNEES	4.33
DIENENCMHTN	4.33
RAPMECQESWK	4.33
PEKKWDKYTIR	4.33
FSATPRYMTYN	4.33
SFNTHFEMEEL	4.33
EGRKKMFESES	4.33
GEPTSDLSDID	4.33
QHPYFSDFCPP	4.33
LAWVSSQFYNK	4.33
LGNELPKFYDE	4.33
IKENLKDCGLF	4.33
VLSIFLYSNSD	4.33
GPASPALNQGS	4.33
REGRNPQLNQQ	4.33
FMAQALQEYNN	4.33
LISFLIFLIVG	4.33
AALPPAPSLLR	4.33
MARQWTKRYAT	4.33
KLLKQKEKKNE	4.33
SETSEASYSGL	4.33
GLVLENSDSLQ	4.33
EMASHLRRSQY	4.33
NFLHVDPRKSN	4.33
YFGAFKVRDID	4.33
FRDSSRLTDIE	4.33
LGLLSPWLSEH	4.33
STCPLCRETFF	4.33
LLDFSSWLGGY	4.33
PLDRAMAELAA	4.33
QELSATVTTDC	4.33
VSYAAGALTVH	4.33
SLPNTLVFGQH	4.33
EDPDEDAIPIT	4.33
PKMIPEFFCDT	4.33
QSTNTAVTLTG	4.33
AQHHIYRLVKD	4.33
LFLEVFEDQEV	4.33
DLLRYMFSRDK	4.33
SPNCLQELLHE	4.33
DVNVLTKAKSQ	4.33
NPTYRFLEERP	4.33
GLPGTKCCKKP	4.33
IKEILEKKLSL	4.33
AVASALSSRYH	4.33
YSIHTYLWHRQ	4.33
ELRSLCCSICS	4.33
NCFRTHEPENV	4.33
AVKIQSSSKST	4.33
ENSSDYQSNLA	4.33
TQKSLSLSPGK	4.33
SNFKSPVKTIR	4.33
TEWIEKTVQAS	4.33
LMWLLVRSYQY	4.33
SYVEVLVPLPQ	4.33
RAAWVREPLEE	4.33
LSSSQVSAHMV	4.33
FARKAKKGKND	4.33
SPAAPGQEDGA	4.33
QRIHMRENLLM	4.33
KKNKKKEKVEA	4.33
GGIGERPQPGF	4.33
QIALNEKLVNL	4.33
PEAGESSTGGP	4.33
QVYCPDCAKKL	4.33
EPLPEKTQESL	4.33
AYLLVYMKMEC	4.33
NYVEEDYSEYC	4.33
PTKWLCLHQKT	4.33
VPSPGPSHHIP	4.33
HSKDHTSTPNP	4.33
MTSSDVVAGSD	4.33
RAKIDNYIPIF	4.33
DDDEDRLVIAT	4.33
QHQQMEQGARP	4.33
VHWIKKTMKRY	4.33
EENPYARFENN	4.33
KRKRTRPTKSS	4.33
AGIDCSAEEAA	4.33
GITVAVHKMAS	4.33
LDHIMEVLVGS	4.33
KLESKLDYKPV	4.33
DGEGTAETIIF	4.33
ADNDNASSFTM	4.33
VEAETTTTFSP	4.33
QAAAETLSEVA	4.33
TDNNIVVYKGE	4.33
PGASGEQCLIM	4.33
IRLDGAIRMQP	4.33
DSMDTSNKEEK	4.33

SISYLTCCNFN	4.33
KTPMVKFSAFS	4.33
VAGRTAGDRLC	4.33
EDSDEEPDHEE	4.33
EHQRVHTGEKP	4.33
DKAGLGKVKMK	4.33
TPSASNDDQQE	4.33
MTPRKLSFDFQ	4.33
QLEAFLKKLIG	4.33
KMNRSVSLTIY	4.33
TNRTGRIRTLP	4.33
LVLLQGARGFA	4.33
AAPYSYDCTKY	4.33
KGTKSEQLSNP	4.33
AHVGYSMYHLT	4.33
GNTTLESTDTL	4.33
TTALPDEDDDL	4.33
AVEHCKRHVWN	4.33
DWIHHYVPKKP	4.33
VLIENLKKASQ	4.33
ENEKKKQKKAS	4.33
SPLQCIHSPDL	4.33
RALSPGRESPK	4.33
TEVKDYLAAIA	4.33
WKKENNKDKFP	4.33
QGCMGLPCVVM	4.33
REDPDVCLKAP	4.33
ESVLLFTQIKR	4.33
RRSKKVQRGSA	4.33
TELKFPSINHD	4.33
TVLLVKDKEQS	4.33
LDYIEPWERNA	4.33
AKKKANLQYYA	4.33
IQPHGGAQYVS	4.33
RTALINFLVQD	4.33
LPKLLTKVKGS	4.33
IRQRSRKGLLH	4.33
NRNPFSSCSIP	4.33
FWKFPHLAVGF	4.33
ISGYYGFYSHS	4.33
LNIAKKLRRQF	4.33
TNLQESLRSKE	4.33
SWKENSPLNVS	4.33
MEGAWSMSASH	4.33
EEESPVNFPIY	4.33
ACLMGKGMKRV	4.33
QKILDGGNKEN	4.33
MDYNETHHEHS	4.33
ESKGSENCKVQ	4.33
LYSMILALIND	4.33
PRTAGPSSLHL	4.33
ALQVFDPLAKT	4.33
IHHRSQKGLLH	4.33
CPSVWKKTKNS	4.33
EQVKRSTYFFS	4.33
IIIIIVWVVSS	4.33
NHYKIHIEEDP	4.33
RFITANQYEMV	4.33
PREEVVQKEQE	4.33
QFYPLGIVQHD	4.33
GDCRKKQDTPH	4.33
REMEEGEYEEA	4.33
IHCLQNHPEHM	4.33
EQKEDQKEEKQ	4.33
GAPPEMASNRK	4.33
LYPRFVQRCTP	4.33
MLPAADGAGPR	4.33
AASHLFPFEKL	4.33
DTNEFHIPLVT	4.33
WGQGDLVTVSS	4.33
DVVCTRVYVRE	4.33
EFQVLVKKISQ	4.33
CPAITQNNFLT	4.33
SEGGPPGAEPQ	4.33
APAPQPSSCSC	4.33
SRRTKEEELQC	4.33
RRHRETQHGLK	4.33
PKAKKAAAKKK	4.33
YDPLMLKKPNS	4.33
EISDSEDDSCK	4.33
YYGSVWGQGTL	4.33
IRELTANFNFS	4.33
TWHSCPEDEAQ	4.33
IITKYQAYDVS	4.33
GYLDVGSKPMY	4.33
LPGSPNPEEPI	4.33
EAAVAIKAMAK	4.33
HRKPKPSSSCC	4.33
LFLIISSVASL	4.33
IVPILEKMQDK	4.33
GPCHGNQTESH	4.33
IDIFSDYANFK	4.33
RVEIMEEESEQ	4.33
YRRVIERLAQE	4.33
CRTRKRTCRRH	4.33
QKTESVDNEGE	4.33
ASSSLAKDTSS	4.33
GQGLEVYNWYD	4.33
PVTPESSSGRM	4.33
MLSTVAVLTLG	4.33
YAGIDATAEEA	4.33
DMLETLQITQS	4.33
AVFGADVLDQV	4.33
EASFFGAFLVG	4.33
EPRPLRRESEI	4.33
SSQVQEIEELM	4.33
KKEKEQKEKDK	4.33
KSKVTNGVGKK	4.33
KSKRGRGRPGR	4.33
MFSRFLNKQPY	4.33
GEDLTKNHDEL	4.33
GGFTWPSCVKK	4.33
HLALHMKRHQN	4.33
PPQNVLAVECR	4.33
LPVPVFKRKIP	4.33
QLPYICQFGIV	4.33
KSNLDRALGRQ	4.33
QPNSGSPQPAP	4.33
PDYISWGTQEQ	4.33
RIGQRKHLVLN	4.33
EHQRIHTGETP	4.33
SWSEWASVPCS	4.33
KFRKSKKKKRY	4.33
KARKKSSCQLL	4.33
TMKGSSTPVKN	4.33
EQGGLPKKLIF	4.33
SSEVKEVEENI	4.33
DTKKQKTDEDD	4.33
YECGKSFCMNS	4.33
TPEGNQNTSES	4.33
EVSASLAKQGL	4.33
NIRELSEGGSS	4.33
PLDSDMPLLDS	4.33
EARGVHTQRMQ	4.33
PNSKHKKIIFF	4.33
RTAFCCNGEKS	4.33
YAGQLPEEPSP	4.33
RGLFVQALPSS	4.33

LLEPIPYEFMA	4.33
DRAEAEEEEEE	4.33
EKDKPSLRVIS	4.33
RHMAAFRSVLS	4.33
INAQKIVLITK	4.33
DIILSLFLNDT	4.33
LQDVVSERANH	4.33
EQSEGSGTDDE	4.33
VAEIQKRLSES	4.33
SHTKEENGLAL	4.33
RRPGLPPSVSN	4.33
VGLMQQQKSFR	4.33
NQYFELAKFLA	4.33
PVGSAAPQGSP	4.33
IDISSYIEQTR	4.33
VTKIDHILDAL	4.33
TPVGYGMYHLS	4.33
KKINKDRAKDE	4.33
SAAKLMDTFDS	4.33
TERMFNAENGK	4.33
ARGMFTAEDLR	4.33
WGEGTLVTISS	4.33
RAITNNQYEIV	4.33
VKTHSEDKSHE	4.33
QEDGKSHRQRS	4.33
RDLSEAHFAPC	4.33
TEPLRDFSAMS	4.33
DDIRKPGESEE	4.33
PIDDKRWNLKA	4.33
GSGVSLTANKV	4.33
PEPAQGALGCL	4.33
AQHHVYKLVKD	4.33
PIPEVDLDDLS	4.33
VGVRLRTEASS	4.33
IRPPRRLVAPS	4.33
EEQKQQPQLLK	4.33
ETLKSLGALKK	4.33
KVETERAQLED	4.33
GYRNVYVDFAL	4.33
CHRRSHIDEKP	4.33
LHQNVHVGEKP	4.33
TTVMNPKFAES	4.33
ENGKICWVKEE	4.33
MGQRMFEDLFE	4.33
LQEPGSKTYSK	4.33
AQPLVVPWEAS	4.33
SGCSPIEEEAH	4.33
FLGKVVNPTEA	4.33
SRSMSQEAQRG	4.33
QVSITGFFQRK	4.33
DISNTDYFFPR	4.33
NAASASASNST	4.33
IDLTSASYTMI	4.33
VRSEFYQELIK	4.33
QDMKKRKYDPS	4.33
LINQKKMSPPL	4.33
KKFKAKGTIDL	4.33
ENLFIGGTKPP	4.33
HQWIVLLSFQL	4.33
WPRGYLKGYPR	4.33
RLFSINECKIF	4.33
GGSGGYGSRRF	4.33
ALDVANKIGII	4.33
NQQGPHTPSIP	4.33
LALEAAVLEAT	4.33
EKEVICPWESL	4.33
YPILKGFRKTT	4.33
LLNACCQGGFI	4.33
RNNRIGFALAR	4.33
VEKTVAPAECS	4.33
SQVCTHLDALK	4.33
KPQITTEPHAT	4.33
HLKKLAVSSAA	4.33
QEGKLQKLAQL	4.33
SDLQKTFEIEL	4.33
VTKQDLGPVPM	4.33
SHQHLSKQKQL	4.33
LNAHETFRCNF	4.33
TKQRIDEFEAM	4.33
CSYSVKGFTKQ	4.33
GTKHWTMNLDR	4.33
SGRRTSDVRRP	4.33
GPLDKWRALHS	4.33
WFPSTYVEEDE	4.33
QRRQRKSRRTI	4.33
RKDKAKSCTVM	4.33
SGYLLGKVGNT	4.33
PPDRVRLLPAP	4.33
INTLSVEGSLL	4.33
RSIMFTVQNED	4.33
PDELSGWDKDF	4.33
KQINPKRPRAL	4.33
FCCSHQQQYDC	4.33
WALENRDEAHS	4.33
EDREKWQMPHT	4.33
DLFLGRWFRSR	4.33
PEAAVISNGEH	4.33
DKKNTNKCSLG	4.33
PQGLDTTPKPH	4.33
PGNSKRMGVSS	4.33
NKCGKTYSHKS	4.33
GGLLSPGPEAT	4.33
AALQHCPKKKL	4.33
GCKVNTAPQES	4.33
LYAGYCNHENM	4.33
TWLREDLQNTR	4.33
EKRSSLVTKIY	4.33
LEAVGGTVVLE	4.33
SSEDSASGSDE	4.33
GKSRESMIQLF	4.33
GNPQVPVHFDG	4.33
ECGTPSVHAGL	4.33
RGLEELSLAQT	4.33
RHQKVHTAGRL	4.33
EGEGEEEGEEY	4.33
KKTGEGFYKYK	4.33
SLATAGSAFQP	4.33
LKDYSDITSSK	4.33
SMFKTFGKDSG	4.33
RLMSHQRTHTG	4.33
QRVHTGEGEAP	4.33
TVPTILGLLLT	4.33
LLHASEPESSS	4.33
DEGCCSACVIL	4.33
LRKSFASLFSD	4.33
PRKRVPDHHPC	4.33
LGGNKKTLGTP	4.33
LATEELLQVAR	4.33
VTTELNIREST	4.33
WGQGTLVTVSS	4.33
QLKMRCDSLML	4.33
AKGVKEEVKLA	4.33
NLIRHQRTHSG	4.33
SSASKGKNKAA	4.33
RQGGAPDAGQE	4.33
STFSGFLVFPL	4.33
TRRTLSMDKGF	4.33
SLRPATVADHE	4.33

LLQGEEEGEED	4.33
YRHYQGEEKSD	4.33
KASIYQNQNSS	4.33
VLCQGCSQAGP	4.33
INVVNEGNYSG	4.33
SSSKQCPLQAW	4.33
PYELKKGMSDI	4.33
SAPKKSKADGQ	4.33
LPKAGGKPFCA	4.33
IFSPKGKEQKT	4.33
PWSQHYHQGYY	4.33
GEKPPEKKCIC	4.33
QKPTKQEEFYA	4.32
MKRVQQSSGPA	4.32
RPPVMSQGGRY	4.32
KTDKEHELLNA	4.32
FLGKVVDPTKP	4.32
VPQQDVILPEY	4.32
ERTHTGEKPYE	4.32
TLAPPLPSASS	4.32
FGQGTRLQIKR	4.32
DQVCRQVFKKK	4.32
ANQDPASIMFN	4.32
AGRGRGRGRPH	4.32
LKAQAQSQKAK	4.32
CNLAQHKKIHT	4.32
DVIEEYFKCKK	4.32
FQSNLVPADPE	4.32
AVYPNAKVELV	4.32
LKSDERPVHIK	4.32
AKHEGEKRYTA	4.32
HLYLFPGHPPT	4.32
VWQSKRTEGIV	4.32
QKEVTSVTSWM	4.32
AMVQAPADAPE	4.32
NLLKHQNVHKG	4.32
GSLPRLTSDAK	4.32
DAAEGPSDIPD	4.32
RHWLEKNFSKR	4.32
AQAVAPQPCGC	4.32
LKSENLLEEEN	4.32
KWIQETIQANS	4.32
PWSNSHFPHEN	4.32
DRNYVPGKVRQ	4.32
SAVYYSACISG	4.32
ITDFTMQFVSS	4.32
NFSYVSPELQP	4.32
WATNCNDDETF	4.32
NQEREGIGYPF	4.32
FGEGTRLQIKR	4.32
GTGPRMTAAYI	4.32
ATGRKIARALF	4.32
EDRSGSGSEED	4.32
GALATPPPAPT	4.32
SDDDMGFGLFD	4.32
TDKSKSNMKGF	4.32
PCQSKYPPKSK	4.32
SHLTVHESIHT	4.32
CCGCCGGDGPV	4.32
DPQLASKMGLH	4.32
TQAPNAQECGC	4.32
RKRRKMRQRSK	4.32
IQQGETPGLDD	4.32
SRVRDKRADIL	4.32
EEIKKAQRTGA	4.32
TTRSHRDSEDI	4.32
EMEEDTVTNGS	4.32
IMRAGMSSLKG	4.32
KWIKDTIVANP	4.32
VNPKFEHLLED	4.32
INSGAVECPAS	4.32
SSAAQGAFQGN	4.32
KCGLQGFDGIV	4.32
RVYLLMKKLAF	4.32
GLSSNFNEWYV	4.32
NGHIKHEKKVK	4.32
LYPQSRGLVPK	4.32
SSCSLDEPSHT	4.32
SVLSMHRNIHT	4.32
CRKCHTARAQT	4.32
PPLEEDDEEGE	4.32
FGPGTKVDIKR	4.32
LSQNKVGSQNY	4.32
SNRSSPATHSP	4.32
NSGVSQSPLVQ	4.32
ANLVTHQRIHT	4.32
VIPVLIFQAYG	4.32
GEDDRDSANGG	4.32
PGGWSPPALWK	4.32
MIGVLAGMALI	4.32
LKERLAAFYGV	4.32
RRKRKSLQRHK	4.32
KKPKKKSCLLL	4.32
ASTSKSESSQK	4.32
TEWVGATTDWS	4.32
EEISDPPEDDE	4.32
DKRLATKQSSG	4.32
PQRSQKASVTP	4.32
LFNPDCATACK	4.32
GLIALLSTGSK	4.32
GKRAQLRAKAN	4.32
QKRNNVLKFTS	4.32
STFLQQHPAAF	4.32
DAEEMEAKAED	4.32
HEHITANFTQY	4.32
FMNPRMERLIS	4.32
ENGYLMEAAPE	4.32
DSFLKAVPSQK	4.32
DSENHIAGQAN	4.32
TTRPASSPSTT	4.32
IMREKYSKCSS	4.32
TYYCQQYNSYP	4.32
PRPLSEPPRPT	4.32
GGDAPAAGEDA	4.32
RLTQHQKIHMG	4.32
VAVRGGGGKQV	4.32
RSWIEETMRDK	4.32
KHEESGCCVAH	4.32
DQKASRNESTF	4.32
SLMDDYLGLVS	4.32
THTGEKSYKYE	4.32
YFTDLNNYDEY	4.32
FSLWSHEQTHN	4.32
DWGVDELIITD	4.32
VACNNFFWENS	4.32
KEARNVTMETE	4.32
RDKRADVGEFF	4.32
ARLSKIHLEKY	4.32
CDDGTSVKLCT	4.32
TNNLRPKKKVK	4.32
PCQPKYPPKSK	4.32
LVTEVQVVQKT	4.32
GNELGLKEITY	4.32
KLQREAHVPLG	4.32
GCSMTDLSALC	4.32
FGQGTRVEIKR	4.32
ISVVSTDDDLA	4.32
HHEKIHTGEKP	4.32

TANLQTKEFNF	4.32
KLFNGEFTKTE	4.32
GYIKGIFGFRS	4.32
HGKNRRQSMLF	4.32
TYYCQQYNSYS	4.32
SITSSDSECDI	4.32
HRSIRHSSIQE	4.32
LSRRCPSQSKY	4.32
PAEKSLSLGTQ	4.32
FGGGTNVEIKR	4.32
DDDDEEEEDDY	4.32
KPTTEEKKPAA	4.32
KMLRHCPSKRK	4.32
FGPGTKVEMTR	4.32
NYVEVIVPLPQ	4.32
NPLLMSAEECV	4.32
FGQGTRVENKG	4.32
TKTRSSGWKSN	4.32
FGGGTKVDFKR	4.32
ANVGAGKKPKE	4.32
YLRKIKARKGK	4.32
SPRHSRSRSRT	4.32
KITPIQSKEAY	4.32
GFNMSSFKLKE	4.32
RKKKDLKIRKK	4.32
LPSIQAIPCAP	4.32
AQLKPPATSDA	4.32
PTRPGMTRPDR	4.32
TTSSSRKSFKS	4.32
QQQQWPGRRHR	4.32
DISASSTEQIL	4.32
TVKDLISRLLQ	4.32
QKEGRCSHSLN	4.32
PQKVKEKMKNA	4.32
AFGSSVTFSFR	4.32
SVSYLFSHVPL	4.32
KESTLKFRTEI	4.32
KHKIIHTGEKP	4.32
TMEEILEESEI	4.32
YYFCLQHDNFP	4.32
LSGNSRELVLD	4.32
KQSSILNQLLL	4.32
AVLLSLAEEDY	4.32
QKYDGIILPGK	4.32
FGVASKVESKR	4.32
QKLMQVVPQET	4.32
IIPVLVVQAQR	4.32
VEVGRLHGALP	4.32
SSTLATHKKIH	4.32
TSDQEDIKATE	4.32
QTLKRDKEKKN	4.32
TVAVFCHAYFA	4.32
SRSRSRSNERK	4.32
PMSANLVDLGS	4.32
QILKSSKTAKE	4.32
NMKGMMGFNNM	4.32
ATGDQVPFKEQ	4.32
VYIVYLSDVFV	4.32
KLTTDMDPSQQ	4.32
TLTTSPRLPVF	4.32
TLWGIQKELQF	4.32
FVVETLICGVV	4.32
EFLPHSICAMQ	4.32
TSILDSAETKN	4.32
EGTSINFCMEL	4.32
QLTDTQMLDEL	4.32
SRSRSRSSSRD	4.32
HYRLPRHTLDS	4.32
QQLRKLRENSS	4.32
VAAIKAALAQG	4.32
KDLDNRGLSRT	4.32
PQFSYSASSTA	4.32
PRASNRSCAIM	4.32
VHKIIITEPSD	4.32
DREKQDKRLSR	4.32
LQEKEREFQEV	4.32
LLQKHSRNSQG	4.32
CYFIHVLKRTD	4.32
AGAAENAFRAP	4.32
ALADMLETPEP	4.32
DHLALHMKRHM	4.32
LTHSGYGDGSD	4.32
PNSMVASPIEA	4.32
STSTPFLSLPE	4.32
HEHEGKKRRKE	4.32
ILNYMIRKMAK	4.32
AGRGWGAGAGQ	4.32
VYYCQQYYSTP	4.32
YIIEGFENMPR	4.32
LCLQQPVERKL	4.32
ILTPENRKHCP	4.32
KISFVIPCNNQ	4.32
RNKEQREEMSG	4.32
KGTSVIITNGS	4.32
ESLQPEPSHPY	4.32
QHVTEAFQFHF	4.32
LASSIGCTLGL	4.32
EEGEEDEGEDD	4.32
QSSQSSQRYSR	4.32
LPLLENAETPQ	4.32
ALIIGLSVGLN	4.32
KARKHAEALGL	4.32
TLCCFCCPCLK	4.32
FSLRRKSRSKD	4.32
ASVTEGQQNEQ	4.32
SPQCQPLHNEL	4.32
GNGPMSVCGRC	4.32
QKSDSSLDAEV	4.32
FKRPLKKKMEK	4.32
LCCWCIKELLD	4.32
HDSLAIERKIK	4.32
FGQGTKLQIKR	4.32
FLGANVQYMRV	4.32
LHWLRNSAHLS	4.32
KRRRESECKAA	4.32
LELKYVCYGGL	4.32
SYYFDLTDGLS	4.32
ELLPNSGCQMQ	4.32
ICDVLFLIGQS	4.32
FGQGTKVEIKR	4.32
LKAAHYHTHKE	4.32
GGSKKYKEFKF	4.32
AASKNLQTSAS	4.32
SGLAVIASDLP	4.32
LTRLKQIGALQ	4.32
AAKDQATAEQP	4.32
QSPGNGTDASH	4.32
TTEGYQPPPVY	4.32
GVNGTVPLTHI	4.32
QIDDLYSIIKV	4.32
NCDSSDPTAYL	4.32
KKFTEDFGQEG	4.32
KPNNDDPCLIS	4.32
GPDGHPGPGTA	4.32
ELLGRAATPAR	4.32
QKYVSDLELSA	4.32
PAGPRELAEHE	4.32
ATEKPLGELWK	4.32

Table S3. Sequences and affinities of one 14-3-3-binding peptide panel (Panni S, et al. *Proteomics* 11, 128-143)

Yeast 14-3-3 (1/1) [1163]

Peptide	LogBLU
IPAWLSLPSTK	4.87
LRRTLSLGKSG	4.86
RQFSKSTTSGL	4.80
RFSRRSDSGVH	4.78
SRIPFSERKLK	4.78
KAVHFSEGGVR	4.76
IAAGASLPQLG	4.75
TVVVQSLIRRR	4.71
KPKSISSSLNR	4.66
ITRSFSVTFNM	4.63
ISFFSSLRPQE	4.61
LTITRSFSVTF	4.60
IEV-RSAGGNG	4.59
NDKDQSLPLLR	4.58
RANVFSVLRTI	4.58
NRISHSSSTTR	4.57
KSISSSLNRIS	4.55
S-TFRSEKGLL	4.54
QRRLSSLSEFN	4.54
VFGPNSSFVRH	4.54
NLKSRSTPSSG	4.54
SKRKNSLNIVA	4.53
LMFIRSTNSWF	4.52
TFEPRSRFLRT	4.52
SLNRISHSSST	4.51
ITKPKSISSSL	4.50
GKQSSSLLSRL	4.49
NWRHASFLCSF	4.49
KLKSSSLLHLA	4.48
IRTQGSFLRRS	4.48
FGPNSSFVRHA	4.47
INVTKSAPPSG	4.47
FQRATSEARTA	4.46
PQ-LRSNTRHH	4.46
KSAPPSGWRQV	4.45
LRKLRSLVPNI	4.44
RQQRASLPLIH	4.43
LKTSASVRSRI	4.43
KQSLLSL-FEI	4.41
LTIAKSFGRHQ	4.41
LTIAKSFGRHQ	4.41
RPLTLSHGSLE	4.40
LPKLKSNPSVP	4.40
TQFWNSLTRRV	4.39
KRHIRSVHSTE	4.39
TKFVRSLVREI	4.39
RLHNISWRILN	4.39
RLSSLSEFNDP	4.39
FQMVSSQNRAQ	4.38
ISHSSSTTRQQ	4.38
RDAHQSLLATR	4.38
LKRTRSMGLLD	4.38
LHNRTSFHRIN	4.37
VTRPLSLKTDI	4.36
AALSKSLACID	4.36
RS-DISMNSSF	4.35
IRFSISSKAAH	4.35
RILNLSWRLHN	4.35
LIKAFSENITK	4.35
ISMNSSFKILP	4.35
LQTAMSLGTVT	4.35

FIMAASLGAS-	4.34
TFGQFSMDSPH	4.33
PNGNLSLDWLN	4.33
TKEVASLPNKS	4.33
KAFRRSEHLKR	4.32
KKFSRSDNLSQ	4.32
KKFSRSDNLSQ	4.32
KKFSRSDNLSQ	4.32
PISFFSSLRPQ	4.31
ET-WKSLVSTR	4.31
EAVFKSLGVKS	4.31
FERDCSVQRRH	4.31
VGGI-SVLKSK	4.30
KRNFLSWKRGL	4.30
VVISESLPGFS	4.30
KLLIGSLRAK-	4.29
ANIFSSVKCFG	4.29
CSCSGST-EIV	4.29
PTPAASAPAPA	4.28
SNCTTSTTPLW	4.28
PEPFRSDVLRN	4.28
RISHSSSTTRQ	4.27
HICPKSFKRSE	4.26
WKSLVSTRVIG	4.25
LHTTLSAPAES	4.25
INNNHSHNSSH	4.25
TTINNSNPNFK	4.24
CIPPISFFSSL	4.24
Q-LSASFSLKN	4.24
WLSLPSTKRIV	4.24
KIASPSIPQ-L	4.23
KRNSSSGNFIF	4.22
FTDPFSLIKTR	4.21
DGKPVSLPLL-	4.21
LSASFSLKNGD	4.21
HKLKKSLDSIP	4.21
RTRTMSVFDNV	4.20
LGTSTSGKNKS	4.17
LRRRKSAVPLM	4.17
DLRASSLQNGI	4.17
SKIGSSLRRRK	4.16
RKSLLSGLFLS	4.16
LKDTASLATNW	4.15
EIAGLSP-ERR	4.15
LFQMVSSQNRA	4.14
LFSAHSLPMDV	4.14
LSIPISCPLIG	4.14
DDTNLSRRRFS	4.14
NSKIGSSLRRR	4.13
LKRNSSSGNFI	4.12
QKLRASLVPGT	4.12
NKS-SSVGVNR	4.11
SNLSSSQQFIK	4.11
ECDVAS-NM-P	4.11
KADAKSVNHLK	4.10
NSKRISMPTKN	4.10
NSDL-SRRQRA	4.09
SLGVKSLGGGA	4.09
KVRSPSSSFRA	4.09
VRSPSSSFRAG	4.09
RQRRLSSLSEF	4.09
KSFKRSEHLKR	4.09
HRRARSTSSFG	4.08
L-SELSLIPTR	4.08

STNTNSPLLRR	4.08
KSMTPSRRSSV	4.07
QQFFLSDMNLN	4.07
LNHKNSSNSKP	4.07
DKI-SSVATPQ	4.06
MANPLSDSASF	4.06
KIFKVSDRRAL	4.05
AGPIKS-GHRF	4.05
NGVATSFSRKK	4.05
IMGFIS-HNGN	4.05
KN-TASVAASK	4.04
FNRWHSESWAN	4.04
KKRLGSFTRAK	4.04
VSST-SPKVRS	4.03
KKGAASNRTKF	4.02
S-RRKSMTPSR	4.02
KVHKGSLIKKG	4.01
RSPSSSFRAGS	4.01
IAETDSWESFF	4.01
GSFLSSSFRHG	4.01
MKSFNSDL-SR	4.01
SFLSSSFRHGS	4.00
MSGLTSQPSIN	4.00
AGSFLSSSFRH	4.00
RRQRASLPIID	4.00
KHLGRSEGNPV	4.00
KDNRLSLPNGA	4.00
LIELVSFAPFK	3.99
GLTKQSESFLA	3.99
A-PNTSLPPSI	3.99
IRSVHSTERPF	3.99
KSKTLSLIQS-	3.99
IWDL-SSAQKI	3.98
NEHSTS-TFRS	3.98
PKINPSNRNTN	3.97
RSDNLSQHLKT	3.97
ISKTVSSTIPR	3.97
MFSSASVRALI	3.97
LKQ-LSASFSL	3.96
MGSRKSSFPQK	3.96
NWAKQSLLSL-	3.96
LLISSSGPIKT	3.94
DMFRTSQGGRP	3.94
RGIC-SLTPAQ	3.94
RARSTSSFGVI	3.94
ALN-NSLDATT	3.93
HIDSKSEFAHR	3.93
PRLNGSMKSLK	3.91
HDEGLSLRRTL	3.90
SFLRRSQLTIT	3.90
EPSI-SNLFLD	3.89
SFRHGSINTPR	3.89
KKRQRSSTKIN	3.88
LSVPGSPRDLR	3.88
ANNSISSPE-T	3.88
PTSLHSRNLSL	3.88
HSRNLSLPIGK	3.87
IKSSTSVQSSA	3.87
RTFDSSWNWAK	3.87
NT-SSSFS-NP	3.86
TKSQESPLNKK	3.86
T-GTRSLETHN	3.85
AKFI-SALFDW	3.85
RRKSSSVTLSP	3.85

NSNSNSTGNLN	3.85
SSKPVSLP-GA	3.84
NGSMKSLKPLN	3.82
TSCSSSFDDHF	3.81
ESFFVSVRKAI	3.81
RSTAQSNKSIL	3.81
LVPTASFFIAS	3.81
VMEVTSSFF-R	3.80
TNQFPSMTNSR	3.80
NKKPTSLHSRN	3.80
PNMLQSVREGR	3.80
NRDTVSDGHLE	3.78
RSISWSVIDRW	3.78
DKKSGSLTFNS	3.78
DISMNSSFKIL	3.76
FKRCESLNGNQ	3.76
ITKDNSVGKIL	3.75
RRRKSSSVTLS	3.74
QNQETSLNLGL	3.74
DPVRKSLLSGL	3.74
PSMTNSRNSIS	3.74
IRSTNSWFDVT	3.74
VGDKVSARNLA	3.74
G-GFLSENSEF	3.73
SESKLSLETLF	3.73
AQQHTSIKDNR	3.73
STTDNSLHLKA	3.73
DKMFVSNPIRK	3.72
SRNSISHSIEF	3.72
GSRRNSSVRKK	3.72
SNKLTSDDVRR	3.70
NSISHSLDLWN	3.69
NIGPGSVPKIL	3.69
KERRPS-RRKS	3.69
LGKQSSSLLSR	3.69
LLL-QSEG-PI	3.69
DKKSNSALFRA	3.69
RKLKFSNRFLP	3.68
IVKPGSRRNSS	3.68
WEGLRS-G-LT	3.67
LQTMESRGVHF	3.67
DLIRNSGEKRA	3.67
TVVTLSEPVQG	3.66
SS-FNSLNIDS	3.66
LIIVLSEMWRF	3.65
EGTQSSENLPR	3.65
SRRNSSVRKKK	3.64
DLAQPSLSSAT	3.64
KAVAFSQ-PHF	3.64
AQRVASLAD-R	3.64
LKVSGSKKTVV	3.63
LKVSGSKKTVV	3.63
PKASDSSQSFR	3.62
SGKNKSACPSS	3.62
HEVTTSIGKRI	3.61
GLPPLSFDSPL	3.61
SRRRFSDVITN	3.61
RNPSPSIVKPG	3.61
NSSVASSTTSR	3.60
KALAGSEINWR	3.60
LSPTISHNNNN	3.59
PASVSST-SPK	3.59
MEVTSSFF-RG	3.58
KQATVSPNTRR	3.58

NETLISPRAQQ	3.58
NSISHSIEFWN	3.57
SNSSVSLKSQH	3.57
VVLLFSAHSLP	3.57
SLPNKSTISKT	3.56
VRSVHSNERPF	3.56
RLVWQSQVGPK	3.56
HSIASSFVTLK	3.55
S-NPQSLGPAS	3.55
RSDNLSQHIKT	3.55
GLI-HSGFISK	3.53
GGNKGSLGKLA	3.51
MTSILSFNRDQ	3.51
SSRNESPVRTI	3.51
NTLLISGPFKV	3.51
PHFS-STTGSS	3.51
THGMFSSASVR	3.51
LDFPDSVMDFF	3.50
TFAGHSLGE-A	3.49
EAETFSSFRPD	3.48
LLKGTSQASRL	3.47
DT-SSSQGNRR	3.47
ENLNRSIELFK	3.47
VARPLSVPGSP	3.45
KRQRSSTKINN	3.45
AFVHAS-PKEL	3.45
QRRANSPARIG	3.44
AVSITSFGFGQ	3.44
DVIP-SLDIKN	3.43
NIIAASRRH	3.43
PNT-SSSFS-N	3.42
DKIGRSVPAPG	3.42
A-RGHSIRANV	3.42
LIQS-SLLDKP	3.41
K-RGRSVDDSD	3.41
FGRPRS-KNDQ	3.40
LGKSGSTNSL-	3.39
Q-PHFS-STTG	3.39
GLVSESLKAIL	3.37
PPPSSSLNPGA	3.37
MSRQPSP-KFP	3.37
NRFPSSTSSSN	3.36
ADD-FSLGVNP	3.36
VNITMSTNRVP	3.36
CSAQPSVQSSF	3.36
GDKI-SSVATP	3.35
PSVQSSFAGTW	3.34
NNLMGSQARSN	3.34
MSTFNS-SQPK	3.34
ISHAGSLTNLS	3.34
KILPDSNRILN	3.33
NGVA-SSLPDN	3.32
HFEKNSFEQFC	3.32
HFEKNSFEQFC	3.32
IKHRTS-ESAT	3.31
TERRGSTISPT	3.31
IKQR-SQLNI-	3.31
ASPFHSHLLVP	3.31
DDRTCSESSSR	3.30
AVIVDSVLAIR	3.30
DTSCSSSFDDH	3.30
ICIMLSLKQPV	3.29
RAFQASIKE-K	3.29
HGMFSSASVRA	3.27

KLASSSGLPIN	3.27
KNK-ES-DAAL	3.25
SNVFGSVLKKQ	3.25
GNLNSS-FNSL	3.24
RTS-ESATTFW	3.23
RTS-ESATTFW	3.23
LFDLDSVRG-S	3.23
KQQKPSLKTSN	3.22
PKW-PSEDVAA	3.22
RWHSESWANQL	3.21
DDFHKSTFNLK	3.21
TPNT-SSSFS-	3.21
SSSSTSLATNH	3.20
RRLANSLDFPD	3.20
NKAALSKSLAC	3.19
FPQKESLFGRP	3.19
SVRG-SNEWKG	3.18
VGTANS-G-KH	3.18
SGPPQSL-GLN	3.17
ASVAASKRKNS	3.16
AETFSSFRPDM	3.16
ARRIPSGTKSQ	3.16
QQQPLSQPLPP	3.16
KSPSISLSSEA	3.15
HLKADSNKNRD	3.14
NHKNSSNSKPT	3.13
NNPNKSLQIGE	3.13
SMFKQSLIELM	3.13
TFNS-SQPKES	3.13
VNKSKSLLDII	3.12
LANKCSMASRI	3.12
MRLL-SCFEAD	3.12
NWAADSVSSRV	3.12
HHTMNSKIGSS	3.11
ETEVVSRNLCG	3.11
GHSIASSFVTL	3.11
PIRKWSE-QAT	3.11
LGGLTSDEKLR	3.10
NWSKDSDVTWL	3.09
QPSVQSSFAGT	3.07
LTFFNSI-WCM	3.07
QKLLASFFFAV	3.06
PAPKIS-KKGA	3.05
LFNSPSDRVKM	3.05
TNKVFSKPRFG	3.04
TFRINS-ERVM	3.04
SMA-DSPVDRA	3.04
SGLFLSEANSN	3.03
KLVHLSNG-KM	3.02
T-GDLSVLPTR	3.02
SPTPMSRQPSP	3.02
NNSENSEERII	3.02
DARR-SEVVTS	3.01
EFSSMSD-LFL	3.01
FGTNVSEWAFS	3.00
GPLRSS-SMA-	3.00
SQMHPSLKLEV	3.00
VQVVKSNGLVC	3.00
TRHRFSRRQSM	2.99
PVKDLSLVFGN	2.99
VKLKSSSLLHL	2.99
SDSSQSFRIVN	2.98
GSRKSSFPQKE	2.98
AINSMSGLTSQ	2.98

FLNNNSINNNH	2.96
HNTAHSE-ASK	2.96
MLQM-SKIRRH	2.95
SHNNNSPSIAN	2.95
NLMVLSQLRAC	2.95
NLMVLSQLRAC	2.95
LSGSISERIVD	2.94
KILPTSQAHHA	2.94
PLKRNSSSGNF	2.94
MARGVSMLNKS	2.94
GCHGLSHRARA	2.93
GSTQLSWVISS	2.92
SK-LHSWKKSD	2.92
SGSTNSL-DLA	2.92
STNMKSFNSDL	2.91
SHNNSSTNMKS	2.91
ERFTKSKTLSL	2.91
HGVPASSPMKA	2.90
SFRAGSFLSSS	2.89
ADSVSSRVRG-	2.89
TDSRNSISHSI	2.88
VQLFNSPSDRV	2.88
DFEALS-NI-N	2.87
LGVFPS--EPW	2.87
LGVFPS--EPW	2.87
FKLASSSGLPI	2.87
NVKLKSSSLLH	2.87
KNLVVSGPPQS	2.86
LQSRRSRSPFQ	2.86
TKFIVSH-ALD	2.86
LTSNPSEKSNK	2.85
NNSISSPE-TF	2.85
WDL-SSAQKIL	2.84
LNKS-SSVGVN	2.84
GPISISRPIRQ	2.84
GVRAVSITSFG	2.84
KD-LLSIPISC	2.83
ITTNVSGFGA-	2.81
ITTNVSGFGA-	2.81
QIITRSEKIVS	2.80
LTNL-SELSLI	2.80
LCASGSFDF-N	2.80
AGTIISPH-DS	2.79
ARTFDSSWNWA	2.79
GENVDSSDLLV	2.78
NILKSSPAQDQ	2.78
LKSNPSVPHLH	2.78
DNLGTSTSGKN	2.78
LHVLLSSKRFD	2.78
GVREGSL-SLL	2.78
PGELRS-LKGA	2.78
TRSFLSPLETD	2.78
DRQLSSLFLDG	2.77
LPTALSTHMFL	2.76
PLPANSQLVNR	2.76
ARSTSSFGVID	2.75
AVTIQSKVRTF	2.75
SSHNNSSTNMK	2.75
SMLNKS-SSVG	2.75
KQEDSSIMNEP	2.75
NSNLSSSQQFI	2.75
QLVNRSVLKGS	2.74
LG-VSSLVEPS	2.74
LEDFVSSEEAL	2.73

ENVDSSDLLVL	2.73
EDFVSSEEALK	2.73
KSHLQSNQL-S	2.71
GFLFGSRVMIA	2.71
KSSVSSANTIN	2.71
GARLISHAGSL	2.70
KPTLGSMFKQS	2.70
AADSVSSRVRG	2.70
LDTSCSSSFDD	2.70
LEAEPSI-SNL	2.69
FNILKSSPAQD	2.69
TIDLNSLL-K-	2.68
TIDLNSLL-K-	2.68
NGIRFSISSKA	2.68
NKGRISR-LAN	2.68
TGNLNSS-FNS	2.68
EVVTSSFPSMT	2.67
SRRSRSPFQHI	2.67
FPASSSTGGIS	2.67
NLNGSSVFENV	2.66
FEKL-SDLMKF	2.66
KGDLWSLVGIP	2.66
ETGE-SVKLLK	2.65
TDCALSLVISC	2.64
KASDSSQSFRI	2.64
SFFIASQLQEQ	2.62
MLLISSSGPIK	2.62
VETKFSKLIGR	2.62
HSWKKSDVKPQ	2.61
ELCQKSPVMAD	2.61
DVAINSMSGLT	2.60
TKNSKSIAAAP	2.60
RDSSNSAQN-L	2.60
LK-MNSHARRA	2.57
ALSG-SMDFSK	2.57
RHHLSSSVPIE	2.57
DLIPISAK-QK	2.56
HHLSSSVPIEA	2.56
MDFSASPFASG	2.56
ITKDLSLLKTI	2.56
REHHSSVK-AS	2.55
DRVGLSL-EVQ	2.55
FALGKSNDPVK	2.54
MNLNGSSVFEN	2.54
LSLIQS-SLLD	2.54
TPLH-SCQ-GL	2.53
ETLDFSLVTPT	2.53
FLSENSEFADK	2.53
A-QFASPVRWI	2.52
V-KAISAQADK	2.52
FKVD-SHTL-F	2.51
AGKTVSAK-IM	2.51
AGKTVSAK-IM	2.51
LRSS-SMA-DS	2.51
KLSRGSVERVK	2.51
NNHIDSLSVDR	2.50
TRQHGSHMLL-	2.50
TLSHGSLEHVL	2.50
TNTSASTNTNS	2.49
PVNRLSRLINT	2.49
AIQHKSIPINS	2.48
TGDTSSFLPVD	2.48
NMLLISSSGPI	2.48
LGHA-SRAKVK	2.47

SRKKKSQPSK-	2.47
VREL-SFVVFA	2.46
HEDRLSTHKQK	2.46
PKESNSSVSLK	2.45
SNQL-SNQLPL	2.44
NSMGLSSILTS	2.44
TNSRNSISHSL	2.43
K-VHVSEVGNC	2.43
TSGLFSNFADK	2.43
AKVKFSVQKND	2.43
NPLSDSASFLS	2.42
GKKVTSMTPAP	2.42
SSVASSTTSRA	2.42
IKALDSERSIS	2.40
RTERLSDLLDW	2.40
RTERLSDLLDW	2.40
LLFRPSPVLFI	2.40
DMDSASQRASH	2.40
NNRFPSSTSSS	2.39
SRGPISISRPI	2.39
LSRLISSKMPG	2.39
QNGQLSLSKTS	2.39
SERSISWSVID	2.39
LTGEWSTKFD-	2.38
GVIGESE-KDK	2.38
KVFKPSQGMVV	2.36
FAGTWSAKEAV	2.35
SKDKKSGSLTF	2.34
TRIMISVLNKA	2.34
TD--QSI-AK-	2.34
EKGLLSATQFT	2.34
SRNSISHSLDL	2.33
VGVNRSILNGS	2.33
DFSKDSGITKP	2.32
NMGGPSKVEET	2.32
KESNSSVSLKS	2.32
AGSDESWTQKL	2.32
TKSSVSSANTI	2.31
KIPGSSKEHKI	2.30
PNRQFSKSTTS	2.30
MQHSNSIMDFS	2.30
IPKIASPSIPQ	2.30
ADLVKSHLQSN	2.29
KKSLDSIPMSK	2.29
VVVTTSRFSKQ	2.29
IAVLDS-TPST	2.29
KKEKKSKKEKK	2.28
DKKVDSVQTTR	2.28
R-LCASGSFDF	2.28
SKNIQSKDS-I	2.27
GQLSLSKTSSR	2.27
LKRNGSTLEQK	2.26
VFDNVSPFKKT	2.26
CQ-GLSEVTKL	2.26
ATTGLSNDMPF	2.25
NIMNRSNDALI	2.25
LSWVISSHGEV	2.25
SISLSSEALAK	2.25
NNERLSMDIQK	2.24
ASSTTSRAHVR	2.24
TVAI-SHEDRL	2.24
VAPLKSQDIPI	2.24
HSHNSSHNNNS	2.24
DLNLKSRSTPS	2.24

QGPLRSS-SMA	2.23
LDSERSISWSV	2.23
FSRRQSMDIPS	2.23
SGLDQSRIPFS	2.23
LGRKTSNGGRI	2.23
TEDLTSLS-LN	2.22
RAAAHSADVFT	2.21
HNINRSKKRDG	2.21
TIENDSEIKST	2.21
ALGKQSSSLLS	2.21
FFKKDSLWQSE	2.21
SASFLSEENED	2.21
PKSISSSLNRI	2.21
FKATSSTDEES	2.21
I-GVVSSFNTV	2.21
GVPASSPMKAG	2.20
EIGGGSPIRKW	2.20
GTGLMSANNII	2.19
VGLFMSAVFEA	2.19
PSSTSSSNRDN	2.19
GVPETSVRVIA	2.19
PNGANSNLFID	2.18
HLTDASD-F-M	2.18
PSPMLSISNLT	2.18
TVWTASPRLDP	2.18
PSTMTSILSFN	2.18
EELNDSNMDAL	2.18
DDEIVSVDTKD	2.16
TQNLASKVKEN	2.16
KEKESSKKRKL	2.16
NMKATSNTLEE	2.16
SNGNTSSKTVV	2.16
GVRTFSQKEMA	2.16
GDGM-SESKLS	2.15
SIDAISQVINI	2.15
FLPVDSCDIVD	2.15
QTLDKSPTNKV	2.15
SWVISSHGEVK	2.15
NI-T-SGIVLI	2.15
PANISSQKKKA	2.15
SSSLLSRLISS	2.15
EDMGPSVSMAV	2.15
HEHSSSIFITP	2.15
SAREKSA-KFF	2.15
AQPSLSSATPQ	2.15
RFSISSKAAHH	2.15
GIALCSRWGGN	2.14
RNKSPSISLSS	2.14
KNLGPSIKEEF	2.14
PRKRKSITTID	2.14
IAVLRSKEWFQ	2.14
KCSMASRIDN-	2.13
SEGLVSESLKA	2.13
LVAKDSVAKFI	2.13
ARGMASK-LHS	2.13
PIAFTSDHIET	2.13
L-GWDS-MMAL	2.13
ASLTDSSNESL	2.13
DSSSVSEDSAV	2.13
RLPAGSDESWT	2.13
NDQISSFIGVL	2.12
VNKTNSHLPAG	2.12
TPAEMSRPATT	2.12
QDDIGSRLKEV	2.12

DSQ-NSSDDEE	2.12
LEHIDSKSEFA	2.12
PFDKKSNSALF	2.12
NESIKSVVSGG	2.12
SQVDSSSVSED	2.11
SLTFNSKNIQS	2.11
RVLSGSISERI	2.11
TNQQMSTMKTR	2.11
HVLLSSKRFDI	2.11
LPKASSKKKNI	2.11
IDHFLSMCQNP	2.10
DLTSLS-LNEP	2.10
DSDWDSVSNDS	2.10
IKEVCSAMKRF	2.10
DLRVLSGSISE	2.10
DD-VSSDLLLN	2.09
NSANNSISSPE	2.09
IFDVTSNGLKK	2.09
VSQALSG-SMD	2.09
DVETHSIVHSD	2.09
LSDSASFLSEE	2.09
FKGLQSGDLER	2.09
FISKASAKNKG	2.09
LLP-ASKHGVT	2.09
TKEKESSKKRK	2.09
NKLFVSKEHAP	2.09
VNNTPSPAEVG	2.09
LFVLVSVVEAF	2.09
GLKPGSIDAIS	2.09
KGHEHSSSIFI	2.09
SEDEDSDWDSV	2.08
MHIKTSKDIAV	2.08
ITLAISDKNLG	2.08
SVSLKSQHSDK	2.08
QQNPPSANGIK	2.08
TGFDTSTVITK	2.08
TLIKASKCVEQ	2.08
STGGISHSTVT	2.07
DLGVASFHGTS	2.07
AALSSSGNDSK	2.07
SVSEDSAVFKA	2.07
KAFLDSMAQK-	2.07
SEEK-SQELEQ	2.07
PVDVQSQVDSS	2.06
KFLESSKEIDP	2.06
SHESNSKSDVK	2.06
RIDN-SEEPSN	2.06
FINTMSAWVNM	2.06
LLDEESRLPAG	2.06
GACATSVESVD	2.06
HSGFISKASAK	2.06
SRLISSKMPGG	2.06
VFKATSSTDEE	2.06
SPTPQSSHSNI	2.06
VELITSINVEN	2.06
VGDVVSTTAVI	2.06
QASRLS-HKDD	2.06
KVKEESTRKNR	2.06
DSIPMSKTIKD	2.05
LDNPNSTNNDV	2.05
TSIANSNGNGN	2.05
HNEAESQLRAA	2.05
AREHHSSVK-A	2.05
GAHDRSLDNPN	2.05

SPPQPSNNERL	2.05
GNDDTSNQRTE	2.05
MLIFGSGFGSA	2.05
VSATSSNDNSA	2.05
TAHELSMQTVA	2.05
GTAVASMTACA	2.05
WLENPSNTPDK	2.05
AEVGASDVAIE	2.05
SLEVASE-IET	2.05
VFALNSILTEE	2.05
NPSEKSNKPTK	2.05
GEVKQSSKPVS	2.05
TRNDNSSRFGK	2.05
SLNDDSLHDLA	2.05
AARLGSEADAK	2.05
NLPKASSKKKN	2.05
TRNDNSSRFGK	2.05
FDKDTSIIGAR	2.04
LSEANSNSNNH	2.04
QSQVDSSSVSE	2.04
VAPNLSELIGE	2.04
SDDDTSSQSSS	2.03
DQNLNSPDNNR	2.03
PAESDSITEEE	2.03
HEWKESEISNS	2.03
DSEEESSSEEE	2.03
VEEENSATVQH	2.03
SAPAESDSITE	2.03
FQDTFSGALGK	2.03
CAGFPSRWTFE	2.03
SVLKGSVTPAN	2.03
ERG-GSDDENS	2.03
IDNETSKSTSP	2.02
PITDNSSVASS	2.02
KLGGRSNPDAV	2.02
NSSSNSSDDD-	2.02
VFTTVSQITAF	2.02
SILEDSLENNE	2.02
SMGLSSILTSN	2.01
LQKLISQ-QVA	2.01
TSLPPSILEDS	2.01
DS-TPSTNEP-	2.01
STTGSSINELW	2.01
PVGENSNGGGD	2.01
TGWVDSKTKEP	2.00
QHEKLS-INTH	2.00
LIKNKSGKIFV	2.00
SILENSTNDRK	2.00
SSGNDSKGHEH	2.00
DVAGGSTQLSW	2.00
EANSNSNNHNT	2.00
SSILTSNPSEK	2.00
LEKLRSNKMHN	2.00
RIPEWSSQ-VG	2.00
AVNGSSIKGQI	2.00
MDPVLSTTNVS	2.00
LDHDVSQALSG	2.00
SIMDFSASPFA	2.00
TRRRKSSSVTL	2.00
LEKVFSPMNPI	2.00
FQEEGSFEFGN	1.99
TAAIDSVVSQI	1.99
DEPIKSIMDGI	1.99
MPTKNSKSIAA	1.99

SEVETSLKELI	1.99
TRNDASLSADE	1.99
TSKSTSPTLEN	1.99
FEDLKSKGLIP	1.99
SQSSSSSHSDD	1.99
VTALASLSPPQ	1.98
TPCTVSPDFVA	1.98
EIE-CSAQPSV	1.98
VVGTLSRNGKP	1.98
TRPMTSPAIAQ	1.98
SSTSSSNRDNE	1.98
EVLLQSLDPNV	1.98
MWDDESGFFFD	1.98
LQILNSGIIPG	1.98
VNDQISSFIGV	1.98
EVLKASTNETL	1.98
SEISNSWK-II	1.97
VQVEESAKLQE	1.97
IKDKASLNDDS	1.97
LDSTKSSVSSA	1.97
KKPLQSRRSRS	1.97
GNCSGSGMGGV	1.97
LNKNTSDDDTS	1.97
SKQAASTVQIL	1.97
E--DNSSNNSS	1.96
MMLENSDLSPN	1.96
SLTNLSKQAAS	1.96
ALDKDSTKEVA	1.96
DPLDHSITTSA	1.96
GVDLSSAASAS	1.96
HLKEVS-KLEN	1.96
PADNDS-LIPQ	1.95
EVVNWSKDSDV	1.95
SRRSDSGVHSP	1.95
LIEIQSILAEL	1.95
QVDSSSVSEDS	1.95
F-VQASQMHPS	1.95
SEKIVSNLN-S	1.95
DTSSQSSSSSH	1.95
LITLGSNDTRN	1.94
NGNTSSKTVVT	1.94
SIDKSSVILDL	1.94
LKVIPSLNDSE	1.94
TRKEASLTDSS	1.94
GAGVPSLEVAS	1.94
NDLIPSGVFWL	1.94
SNLN-SQALVA	1.94
AHNKNS-DEGR	1.94
KQVEISLVNGA	1.94
NKIFFSSEDED	1.94
NDNTNSMQSIL	1.94
SSVTLSPTISH	1.94
SEVVTSSFPSM	1.94
KEVFVSDGENV	1.94
VAVNGSSIKGQ	1.94
QFSMDSPHRTD	1.94
KKQEDSSIMNE	1.94
LRRNPSPSIVK	1.94
EDDT-SSSQGN	1.93
SLTDSSNESLH	1.93
DNENNSMGLSS	1.93
EILC-SKDAKE	1.93
DWDSVSNDSEF	1.93
GM-SESKLSLE	1.93

SMDIPSKNRNT	1.93
TKMRDSSNSAQ	1.93
PAKNNSFTVEA	1.93
ANTINSNTSHD	1.92
N-SSSSDSEQQ	1.92
EEEKSSACTLS	1.92
TRMEISEEEKM	1.92
FLG-VSSLVEP	1.92
INSNTSHDPVR	1.92
DL-KTSKAAQD	1.92
HSITTSAMWKE	1.92
QQNLDSTKSSV	1.91
DSSNESLHKVV	1.91
LLVPASDLINK	1.91
QFANDSLITKD	1.91
DDDTSSQSSSS	1.91
AIATGSNTRHR	1.91
AK-ETSILEHS	1.91
SILEHSGIRLI	1.91
GGGHHSFEDAH	1.91
KVRKLSRGSVE	1.91
FNEIKSTLVVD	1.91
ESNSKSDVKIS	1.91
EAFIASGITDP	1.90
SQVNTSQGPVA	1.90
SIPINSATEHL	1.90
MGGGASNFNNM	1.90
DDLKTSASVRS	1.90
TSSQSSSSSHS	1.90
SACTLS-ILEE	1.90
DREVVSEAINI	1.90
ESNDNSHNNVN	1.90
IESMSSIQ-VE	1.90
SLPGFSAGETS	1.90
QSSSSSHSDDE	1.89
TEEEKSSACTL	1.89
HIDSLSVDREN	1.89
DDT-SSSQGNR	1.89
KESEISNSWK-	1.89
ME-HISNTDET	1.89
SEEESSSEEEQ	1.88
VVSGESGAGKT	1.88
VVSGESGAGKT	1.88
PPETESDHFDT	1.88
EINEHSTS-TF	1.88
NKMHNSIVMIQ	1.88
NNISSSSVNST	1.88
IVDIGSNGIRF	1.88
SDDENSKRISM	1.88
SASVRSRIITL	1.88
FDFHVSKRSEV	1.88
NFLDTSCSSSF	1.88
TRHHLSSSVPI	1.88
TPPPSSSLNPG	1.88
RIMQHSNSIMD	1.88
KNSSNSKPTQQ	1.88
PLQSPSPSSVP	1.87
QIEKGSTFGIN	1.87
PLARVSKDKKS	1.87
SSSSDSEQQFI	1.87
VKVSISHDDLQ	1.87
NETSKSTSPTL	1.87
TKQSESFLAQV	1.87
EVKQSSKPVSL	1.87

LENMGSRKSSF	1.86
EPFEASKETAE	1.86
DKQDISSSTRV	1.86
EGSL-SLLPKE	1.86
VATSFSRKKAR	1.86
IEG-FSPRNES	1.86
DSTKSSVSSAN	1.86
KQPKASDSSQS	1.86
NISSSSVNSTG	1.86
SEDDESDDENK	1.86
SPRNESIKSVV	1.86
GHEHSSSIFIT	1.86
MGPSVSMAVQA	1.86
TDVKVSISHDD	1.85
G-QEHSTSVSP	1.85
ACTERSRGPIS	1.85
SDDIISQVDPI	1.85
LADVMSIESLV	1.85
LEQAGSKKPGP	1.85
STLSDSDTETE	1.85
PSLKTSNVTAL	1.85
AQK-ASIVGVD	1.85
GFAPISEVMED	1.85
HVSKRSEVETS	1.85
ETIRISCAGFP	1.85
ETIRISCAGFP	1.85
GT-MLSNLLQE	1.84
GT-MLSNLLQE	1.84
Q-LQISQAIK-	1.84
QPSLSSATPQQ	1.84
TTSRFSKQVTD	1.84
PFNQGSKQDVE	1.83
DN-SSSSDSEQ	1.83
QKIPGSSKEHK	1.83
RNDNSSRFGK-	1.83
RNDNSSRFGK-	1.83
EMKHASVIMDP	1.83
QDISSSTRVEF	1.83
KEVKTSPDAKK	1.83
TALKTSIQHVG	1.83
GGG-FSAAGMT	1.83
QFIEDSQ-NSS	1.82
EHFNPSTGEKT	1.82
QEHSTSVSPPP	1.82
ERLLSSNQFNE	1.82
KQVQRSPSPIH	1.82
FGDAESLTPLH	1.82
ADLRASSLQNG	1.82
ALASLSPPQPS	1.81
DIIFSSFHPDI	1.81
HSTSVSPPPAD	1.81
NEGVPSPMLSI	1.81
RGVRESGHDTT	1.81
KMRDSSNSAQN	1.81
SSNNSSSNSSD	1.81
SPKVRSPSSSF	1.81
AEEIPSEDQNE	1.81
KT-GISDDIIS	1.81
EEESSSEEEQR	1.80
KEAALSSSGND	1.80
SDVKISGNDND	1.80
ELKEESKKQLE	1.80
LKSQHSDKKDN	1.80
SLSKTSSRKGK	1.79

KLGILSLLDEE	1.79
KLGILSLLDEE	1.79
AEVIDSVGDKV	1.79
ITPPPSSSLNP	1.79
TSTKVSVEGVN	1.79
CEKKFSRSDNL	1.79
KKIKSSTSVQS	1.79
KLLGRSDDL-D	1.79
STDEESWFKAL	1.79
ENNLQSTEQTL	1.79
GSGFGSADDT-	1.79
DKKDNSTIPNP	1.79
VAIQLSKRDVV	1.79
VQNNRSTAQSN	1.78
VILPMSPNHGT	1.78
TVKDKSK-QIG	1.78
TKEKVSNGGVG	1.78
GMGGVSALRGM	1.78
DTFDGSDLRVL	1.77
GNIPESEEK-S	1.77
LSKTSSRKGKN	1.77
DSIDKSSVILD	1.77
RSVDDSDVSTL	1.77
DTRNKSPSISL	1.77
IKELRSKG-PI	1.77
VMSIESLVEVV	1.77
ENSDLSPNDLN	1.77
IREN-SAMIFE	1.77
EEIEWSFIEFN	1.76
NSISNSNSNST	1.76
ITDNSSVASST	1.76
IPEWSSQ-VG-	1.76
PQSSHSNIGPQ	1.76
KAKAPSGLDQS	1.76
PTPQSSHSNIG	1.76
LTEGGSEQMAD	1.76
LSLVISCNDSI	1.76
TPANISSQKKK	1.76
GATGHSQGLVT	1.76
VKKQKSARGIE	1.76
RDIIFSSFHPD	1.76
MKFLESSKEID	1.76
SSQSSSSSHSD	1.76
IKSVVSGGNMM	1.75
PLLIASRP-AP	1.75
SSSNSSDDD-D	1.75
NNVNKSKSLLD	1.74
QNEVKSLKEEI	1.74
DLHLKSALHVQ	1.74
LVGGKSTVQNE	1.74
QTRRGSEDDT-	1.74
MNTINSTNVH-	1.74
VEIGPSPTLAG	1.73
DTF-NSQ-AE-	1.73
SK-TNSEGEIV	1.73
QLVPPSLLN-D	1.73
ISCNDSIDKSS	1.72
RWGLPSGRQDG	1.72
KAPDESVEQLV	1.71
KTDNESNHL-E	1.71
SSSSHSDDEEH	1.71
VAD-ESPIPQE	1.71
QLNSGSEEKEP	1.71
DTNPNSNMNTT	1.70

NNNSPSIANNT	1.70
PSMTDSRNSIS	1.70
AQFMVSNKLTS	1.70
ATVCFSGDMLQ	1.70
KVTPTSKVVGD	1.70
HQHATSPKTNL	1.70
MEMIISSPSDG	1.70
TNTNTSASTNT	1.70
RGSTISPTTTI	1.70
VHTHDSAGTAV	1.70
NHSHNSSHNNN	1.70
SVK-ASPNLNM	1.70
NDKNESATINE	1.69
ASFLCSFITQD	1.69
KQEILSRKKKS	1.69
NSSNNSSSNSS	1.69
FPSSTSSSNRD	1.69
GSDEESDNGEF	1.68
SQ-NSSDDEEE	1.68
DDDN-SSSSDS	1.68
RLDNTSIANSN	1.68
NKSTISKTVSS	1.68
FLPVASPFHSH	1.68
PITKESQPTIE	1.68
HVD-LSHEWKE	1.67
PSLNDSEDINR	1.67
LLVALSNEPAA	1.67
DDSDVSTLRRA	1.66
LDNNESLILIT	1.66
FTGQGSQEQGM	1.66
PETGLSR-HPN	1.66
MGQDISETDME	1.66
CEDFVSRPDRT	1.65
EPVKASLLLHV	1.65
GNIT-SEEPRE	1.64
EVGNCSGSGMG	1.64
ASLGAS-VEFD	1.64
QTIVVSGESGA	1.63
GAGKGSIGAEV	1.63
MNNNESEAENQ	1.62
LSPASSSSTSL	1.62
PIDDVSAFGDA	1.62
MVRTISRLDNT	1.62
ATSVESVDIGV	1.61
SFHGTSTKAND	1.60
IVTVRSEMGEP	1.60
RSTPSSGNNHI	1.59
SPSPSSVPQNP	1.59
ELLKSSDKVML	1.59
LLGIVSAAAPI	1.59
HSE-ASKHVSP	1.58
PSISLSSEALA	1.58
IDEIISIAQKH	1.57
SATPPSNTSSN	1.57
TSSFPSMTDSR	1.57
DATTMSAFDNN	1.57
SFANDSVMANP	1.57
AKLATSGVDNI	1.57
SPH-DSMLVKC	1.57
VKCSCSGST-E	1.57
TSVQSSATPPS	1.57
STTNVSATSSN	1.57
IISSPSDGQVK	1.57
HSIVHSDLLND	1.57

RVADRSQKVET	1.57
IDSVVSQIEKG	1.57
VGVDLSSAASA	1.57
HSQLNSGSEEK	1.57
AQSNKSILENS	1.57
KIFFSSEDEDS	1.57
RGAGHSPHHVN	1.57
ENTVESFIMAA	1.56
DDN-SSSSDSE	1.54
I-D-NSV-TGD	1.54
AESQSS-LDDM	1.54
DSGVHSPITDN	1.53
LR-PASQSEDD	1.53
MNEQNSKAELH	1.52
PLNEISAGELN	1.52
SAGETSGFLNK	1.52
KKSQPSK-TNS	1.52
QLHKASANNTT	1.52
NDASLSADEPN	1.51
DHQDHSEMDTL	1.51
FERLLSSNQFN	1.51
KQDISSSTRVE	1.49
HNVNTSRG-D-	1.48
NLKTVSPEVVN	1.48
EAALSSSGNDS	1.48
T-EEFSSMSD-	1.46
EEDIISVVKMI	1.45
SQPKESNDNSH	1.45
H-PKESNSSVS	1.45
SVSNDSEF-AD	1.45
SLVEPSKVGQF	1.43
IEEDDSKGLIR	1.43
SILNGSVTVPI	1.43
IDNQKSKDMEL	1.43
SKSTTSGLFSN	1.43
TLN-GSDEESD	1.41
LSLGKSGSTNS	1.41
LNDMDSASQRA	1.40
ENIELSPHQHA	1.40
NPINDSHFNEN	1.40
EMIISSPSDGQ	1.40
NVSATSSNDNS	1.40
PDIKCSNCTTS	1.40
ETVTKSKADMH	1.40
IHDIESFANDS	1.40
WKPLDSAIDHH	1.40
PSNTSSNPDIK	1.40
TAVIESVVNQP	1.40
LLNEDSGIAQR	1.40
QN-LDSPKIQK	1.40
GTSQASRLS-H	1.40
VETILSGKARI	1.40
VTHPHSQLNSG	1.40
TDSWESFFVSV	1.40
SNNSSSNSSDD	1.38
IFNSDSEEESS	1.38
VVVRISKDDLK	1.38
GTVTTSVLPQT	1.38
HQVEMSETEQK	1.36
GPGGASGLGVL	1.32
IDDSLS-DLVN	1.26
ASKHVSPTPQS	1.26
SRSTPSSGNNH	1.23
GGISHSTVTNV	1.20

DT-GFSILDIV	1.15
PMLSISNLTQE	1.11
LFKLASSSGLP	1.11
SSNDNSANNSI	1.08
AIDHHSGDTNP	1.08
LKKIKSSTSVQ	1.08
PPSNTSSNPDI	1.08
MLVKCSCSGST	1.08
STSVQSSATPP	1.08
KRHVRSVHSNE	0.90
CDKKFSRSDNL	0.78
NMNTVSPTPMS	0.30
AEVKSVNDTP	0.30
SKTVSSTIPRE	0.30

Table S4. The MolSurf descriptor [Norinder U, et al. *J Comput Chem* 1998, 19: 51-59]

AA	1	2	3	4	5	6	7	8	9	10	11	12	13	14	15	16	17	18	19	20	21	22
Ala	-47.427	25.895	-0.43	-0.708	8.095	-30.767	28.045	-0.561	-0.743	-2.644	124.669	1.103	0.952	0.559	0.725	1.261	37.988	-0.093	0.168	0.227	0.507	0.311
Arg	-44.933	25.115	-0.436	-0.714	7.147	-27.758	30.297	-0.569	-0.713	-1.969	214.078	3.309	2.189	0.584	1.16	1.576	135.583	1.333	1.135	0.552	1.16	0.687
Asn	-38.725	28.492	-0.45	-0.72	5.196	-16.326	32.051	-0.575	-0.738	-4.028	156.011	1.456	1.305	0.644	1.052	1.759	72.969	-0.753	0.432	0.665	1.052	0.595
Asp	-41.817	20.034	-0.437	-0.715	7.008	-28.807	24.856	-0.566	-0.744	-3.322	152.513	1.85	1.242	0.62	0.58	1.532	68.631	-0.22	0.372	0.589	0.453	1.511
CyS	-40.684	28.519	-0.445	-0.701	5.876	-24.929	36.075	-0.459	-0.731	-2.837	148.596	1.893	1.281	0.534	0.534	1.553	65.063	0.477	0.407	0.343	0.394	0.54
Gln	-43.968	18.338	-0.439	-0.716	6.673	-29.471	32.462	-0.546	-0.737	-2.934	178.958	2.122	1.623	0.647	1.015	1.501	95.356	-0.344	0.641	0.63	1.015	0.96
Glu	-38.677	17.585	-0.441	-0.715	6.389	-20.348	22.057	-0.58	-0.748	-3.779	170.073	2.371	1.484	0.605	0.647	1.425	87.73	0.384	0.551	0.557	0.559	1.299
Gly	-45.272	26.781	-0.438	-0.726	6.789	-24.925	33.62	-0.561	-0.759	-3.606	105.64	0.592	0.725	0.67	0.734	1.414	7.206	-0.616	0.014	0.163	0.321	0.112
His	-47.134	21.156	-0.432	-0.686	7.695	-29.818	21.51	-0.504	-0.723	-1.662	183.436	2.397	1.732	0.603	0.867	1.473	100.603	0.249	0.722	0.566	0.867	1.473
Ile	-46.949	24.836	-0.435	-0.688	7.421	-18.1	25.003	-0.554	-0.726	-1.881	177.699	2.608	1.672	0.415	0.711	1.311	98.084	1.529	0.718	0.189	0.651	0.309
Leu	-46.288	26.079	-0.435	-0.7	7.406	-19.63	29.344	-0.548	-0.736	-3.649	182.107	2.676	1.735	0.383	0.691	1.381	102.422	1.678	0.768	0.153	0.687	0.284
Lys	-42.745	20.469	-0.442	-0.713	6.415	-25.582	27.29	-0.57	-0.75	-3.412	199.806	2.98	1.991	0.476	0.886	1.377	116.134	1.092	0.916	0.316	0.886	0.316
Met	-38.92	23.648	-0.448	-0.719	5.583	-30.625	30.62	-0.526	-0.759	-3.694	191.341	3.043	1.891	0.481	0.702	1.442	107.039	1.581	0.84	0.314	0.466	0.414
Pro	-43.888	20.816	-0.435	-0.692	7.291	-29.901	20.033	-0.492	-0.729	-1.563	204.284	3.428	2.064	0.48	0.736	1.298	121.451	2.021	0.976	0.327	0.473	0.199
Phe	-44.254	11.816	-0.431	-0.709	7.811	-32.026	12.404	-0.564	-0.777	-2.734	153.003	1.788	1.305	0.457	0.736	1.426	77.446	1.005	0.481	0.149	0.405	0.171
Ser	-43.559	25.417	-0.44	-0.724	6.616	-28.065	26.081	-0.552	-0.74	-3.029	131.385	1.323	1.003	0.601	0.766	1.677	44.705	-0.733	0.204	0.555	0.766	0.36
Thr	-43.967	22.944	-0.44	-0.722	6.602	-25.206	24.962	-0.558	-0.734	-2.734	149.855	1.772	1.246	0.532	0.739	1.641	67.162	-0.043	0.389	0.405	0.739	0.366
Trp	-45.131	20.079	-0.43	-0.687	7.877	-29.553	18.09	-0.492	-0.725	-1.292	236.955	4.108	2.587	0.536	0.785	1.344	153.493	2.379	1.379	0.454	0.571	1.344
Tyr	-43.217	23.203	-0.436	-0.693	7.207	-24.4	19.868	-0.49	-0.729	-1.668	212.749	3.397	2.159	0.557	0.73	1.632	128.937	1.547	1.038	0.472	0.516	1.632
Val	-43.874	21.386	-0.442	-0.712	6.34	-26.173	32.336	-0.553	-0.718	-0.815	160.979	1.997	1.429	0.437	0.713	1.4	79.965	1.117	0.521	0.165	0.494	0.216

Table S5. The VHSE descriptor [Mei H, et al. *Biopolymers* 2005, 80: 775-786]

AA	1	2	3	4	5	6	7	8
Ala	0.15	-1.11	-1.35	-0.92	0.02	-0.91	0.36	-0.48
Arg	-1.47	1.45	1.24	1.27	1.55	1.47	1.30	0.83
Asn	-0.99	0.00	-0.37	0.69	-0.55	0.85	0.73	-0.80
Asp	-1.15	0.67	-0.41	-0.01	-2.68	1.31	0.03	0.56
Cys	0.18	-1.67	-0.46	-0.21	0.00	1.2	-1.61	-0.19
Gln	-0.96	0.12	0.18	0.16	0.09	0.42	-0.20	-0.41
Glu	-1.18	0.40	0.1	0.36	-2.16	-0.17	0.91	0.02
Gly	-0.20	-1.53	-2.63	2.28	-0.53	-1.18	2.01	-1.34
His	-0.43	-0.25	0.37	0.19	0.51	1.28	0.93	0.65
Ile	1.27	-0.14	0.3	-1.80	0.3	-1.61	-0.16	-0.13
Ala	1.36	0.07	0.26	-0.80	0.22	-1.37	0.08	-0.62
Lys	-1.17	0.70	0.70	0.80	1.64	0.67	1.63	0.13
Met	1.01	-0.53	0.43	0.00	0.23	0.10	-0.86	-0.68
Phe	1.52	0.61	0.96	-0.16	0.25	0.28	-1.33	-0.20
Pro	0.22	-0.17	-0.50	0.05	-0.01	-1.34	-0.19	3.56
Ser	-0.67	-0.86	-1.07	-0.41	-0.32	0.27	-0.64	0.11
Thr	-0.34	-0.51	-0.55	-1.06	-0.06	-0.01	-0.79	0.39
Trp	1.5	2.06	1.79	0.75	0.75	-0.13	-1.01	-0.85
Tyr	0.61	1.60	1.17	0.73	0.53	0.25	-0.96	-0.52
Val	0.76	-0.92	-0.17	-1.91	0.22	-1.40	-0.24	-0.03

Table S6. The ST_scale descriptor [Yang L, et al. *Amino Acids* 2010, 38: 805-816]

AA	1	2	3	4	5	6	7	8
Ala	−1.552	−0.791	−0.627	0.237	−0.461	−2.229	0.283	1.221
Arg	−0.059	0.731	−0.013	−0.096	−0.253	0.300	1.256	0.854
Asn	−0.888	−0.057	−0.651	−0.214	0.917	0.164	−0.14	−0.166
Asp	−0.907	−0.054	−0.781	−0.248	1.120	0.101	−0.245	−0.075
Cys	−1.276	−0.401	0.134	0.859	−0.196	−0.720	0.639	−0.857
Gln	−0.622	0.228	−0.193	−0.105	0.418	0.474	0.172	0.408
Glu	−0.629	0.390	−0.38	−0.366	0.635	0.514	0.175	0.367
Gly	−1.844	−0.018	−0.184	0.573	−0.728	−3.317	0.166	2.522
His	−0.225	0.361	0.079	−1.037	0.568	0.273	1.208	−0.001
Ile	−0.785	−1.010	−0.349	−0.097	−0.402	1.091	−0.139	−0.764
Leu	−0.826	−0.379	0.038	−0.059	−0.625	1.025	−0.229	−0.129
Lys	−0.504	0.245	0.297	−0.065	−0.387	1.011	0.525	0.553
Met	−0.693	0.498	0.658	0.457	−0.231	1.064	0.248	−0.778
Phe	−0.019	0.024	1.08	−0.220	−0.937	0.570	−0.357	0.278
Pro	−1.049	−0.407	−0.067	−0.066	−0.813	−0.89	0.021	−0.894
Ser	−1.343	−0.311	−0.917	−0.049	0.549	−1.533	0.166	0.280
Thr	−1.061	−0.928	−0.911	−0.063	0.538	−0.775	−0.147	−0.717
Trp	0.853	0.039	0.260	−1.163	0.160	−0.202	1.010	0.195
Tyr	0.308	0.569	1.100	−0.464	−0.144	−0.354	−1.099	0.162
Val	−1.133	−0.893	−0.325	0.303	−0.561	−0.175	−0.020	−0.311

Table S7. The VSGETAWAY descriptor [Tong J, et al. *Acta Phys Chim Sin* 2007, 23: 37-43]

AA	1	2	3	4	5	6	7	8	9	10
Ala	−13.3292	3.6829	2.2015	−1.4080	1.8732	−2.0962	−2.5160	3.5103	−0.5706	1.5013
Arg	−11.2913	−13.1394	5.3609	−6.7621	−3.5033	2.8519	1.6329	−0.3612	1.0842	−1.7434
Asn	−4.6803	−2.8190	−2.4010	5.4900	−4.3388	−0.3456	1.0276	−1.9336	−2.6566	1.9034
Asp	1.2855	0.9420	−1.2927	5.1455	−3.6391	1.9940	−3.7780	−0.2168	1.7607	−2.6527
Cys	9.1403	−0.4953	−0.3565	−2.4070	4.3414	1.2868	−3.7403	−2.5182	−2.7351	−3.8324
Gln	−18.8109	9.4759	8.5528	1.7770	3.4446	2.0659	1.6643	−3.7220	−0.6091	−0.1897
Glu	4.8136	−3.6530	0.6884	4.6949	3.2083	3.4907	0.7508	3.9803	−2.4151	−1.2698
Gly	2.3145	1.2451	−9.0973	−2.9242	0.1405	2.9439	1.5991	−3.1366	−0.5333	1.8836
His	7.0189	7.2638	1.3166	−1.0982	−1.3657	1.3162	0.3668	0.0559	4.3363	0.8044
Ile	4.0021	3.1324	−6.0055	−0.6737	3.4485	1.7852	4.0495	1.9852	1.1993	−0.8973
Leu	2.5262	−3.3520	3.1267	1.4733	1.1690	4.4285	0.3656	2.0470	1.3917	2.4648
Lys	−2.0569	−5.9173	−2.3141	5.1890	0.6859	−4.4031	3.3484	−1.5518	0.0055	−0.8094
Met	−5.3564	−2.5982	−1.5658	−1.5809	4.2002	−6.7597	0.4828	0.4241	2.3380	−1.4409
Phe	3.1423	−0.6288	−0.8385	3.0728	−0.9124	−0.3338	−1.6472	−0.8950	3.7509	−0.6515
Pro	11.7111	8.5536	3.6184	−2.8284	−6.2283	−2.3249	3.2271	1.5940	−2.1652	−2.4779
Ser	−10.2951	0.8968	−0.8369	0.5678	−2.2146	−1.3259	−1.1883	2.4772	−1.5573	0.7491
Thr	−6.3073	1.0871	−3.9725	−1.8504	−3.1014	−1.0617	−3.0306	−0.2457	−0.2105	0.8411
Trp	−3.2399	1.1922	−6.8036	−4.4173	0.8739	0.4566	−0.9949	0.4824	−1.2334	1.0674
Tyr	17.5268	−3.1396	6.0743	−1.2177	1.6572	−2.2498	−0.9616	−1.1068	−0.6444	3.4173
Val	11.8859	−1.7292	4.5446	−0.2425	0.2610	−1.7189	−0.6579	−0.8688	−0.5358	1.3326

Table S8. Statistics of the pQSAR modeling of 10 SH3-binding peptide panels by systematically combining five MLMs and four AADs

MolSurf

Human amphiphysin SH3 (1/1)

MLM	R_{fit}^2	R_{prd}^2	R_{cv}^2	$RMSE_{fit}$	$RMSE_{cv}$	$RMSE_{prd}$
PLS	0.6607	0.6155	0.5659	0.5014	0.5672	0.5489
GP	0.6891	0.6374	0.5891	0.4800	0.5518	0.5331
RF	0.8854	0.6345	0.5719	0.2914	0.5633	0.5352
SVM	0.9381	0.6172	0.6062	0.2124	0.5402	0.5477
LibSVM	0.6111	0.5535	0.5513	0.3178	0.3592	0.3631

Yeast amphiphysin SH3 (1/1)

MLM	R_{fit}^2	R_{prd}^2	R_{cv}^2	$RMSE_{fit}$	$RMSE_{cv}$	$RMSE_{prd}$
PLS	0.6610	0.5940	0.6190	0.5089	0.5395	0.5452
GP	0.7154	0.6229	0.6523	0.4663	0.5154	0.5254
RF	0.8949	0.5965	0.6583	0.2833	0.5109	0.5435
SVM	0.8156	0.6542	0.6763	0.3754	0.4973	0.5032
LibSVM	0.6395	0.5620	0.5941	0.2845	0.3177	0.3246

Yeast Boil SH3 (1/1)

MLM	R_{fit}^2	R_{prd}^2	R_{cv}^2	$RMSE_{fit}$	$RMSE_{cv}$	$RMSE_{prd}$
PLS	0.3579	0.2760	0.1855	0.5286	0.5954	0.6099
GP	0.4171	0.2962	0.1869	0.5037	0.5949	0.6013
RF	0.8018	0.2930	0.2101	0.2937	0.5863	0.6027
SVM	0.5086	0.2717	0.1914	0.4624	0.5932	0.6117
LibSVM	0.3036	0.2578	0.1830	0.3408	0.3739	0.4300

Yeast Boi2 SH3 (1/1)

MLM	R_{fit}^2	R_{prd}^2	R_{cv}^2	$RMSE_{fit}$	$RMSE_{cv}$	$RMSE_{prd}$
PLS	0.2391	0.0747	0.1352	0.5296	0.5646	0.4468
GP	0.3766	0.0988	0.1544	0.4794	0.5583	0.4410
RF	0.7708	0.0972	0.1338	0.2907	0.5651	0.4414
SVM	0.5009	0.1324	0.1774	0.4289	0.5507	0.4327
LibSVM	0.2829	0.1593	0.1615	0.2872	0.3151	0.1826

Yeast endophilin SH3 (1/1)

MLM	R_{fit}^2	R_{prd}^2	R_{cv}^2	$RMSE_{fit}$	$RMSE_{cv}$	$RMSE_{prd}$
PLS	0.5794	0.5316	0.5124	0.5464	0.5883	0.5848
GP	0.6060	0.5535	0.5714	0.5289	0.5400	0.5709
RF	0.8549	0.5306	0.4930	0.3209	0.5999	0.5854
SVM	0.7768	0.6315	0.5979	0.3980	0.5343	0.5186
LibSVM	0.5583	0.5539	0.5059	0.3171	0.3527	0.3312

Yeast Myosin5 SH3 (1/1)

MLM	R_{fit}^2	R_{prd}^2	R_{cv}^2	$RMSE_{fit}$	$RMSE_{cv}$	$RMSE_{prd}$
PLS	0.4664	0.3467	0.3470	0.4214	0.4662	0.4062
GP	0.4898	0.3766	0.3603	0.4121	0.4614	0.3968
RF	0.8186	0.2864	0.3317	0.2457	0.4716	0.4246
SVM	0.6013	0.3541	0.3464	0.3643	0.4664	0.4039
LibSVM	0.4237	0.3454	0.3001	0.2368	0.2601	0.1819

Yeast Rvs167 SH3 (1/1)

MLM	R_{fit}^2	R_{prd}^2	R_{cv}^2	$RMSE_{fit}$	$RMSE_{cv}$	$RMSE_{prd}$
PLS	0.4019	0.3113	0.2634	0.5958	0.6612	0.7026
GP	0.4901	0.3633	0.2786	0.5502	0.6544	0.6756
RF	0.8328	0.4286	0.3988	0.3151	0.5974	0.6400
SVM	0.9459	0.4021	0.3614	0.1792	0.6157	0.6547
LibSVM	0.3528	0.3039	0.2312	0.4579	0.4983	0.5869

Yeast Sho1 SH3 (1/1)

MLM	R_{fit}^2	R_{prd}^2	R_{cv}^2	$RMSE_{fit}$	$RMSE_{cv}$	$RMSE_{prd}$
PLS	0.5351	0.3592	0.3647	0.4947	0.5783	0.5401
GP	0.6310	0.4025	0.4033	0.4407	0.5605	0.5216
RF	0.8586	0.5434	0.4878	0.2728	0.5193	0.4560
SVM	0.9210	0.3872	0.4137	0.2039	0.5556	0.5282
LibSVM	0.3527	0.2578	0.2420	0.3980	0.4333	0.3842

Yeast Yfr024 SH3 (1/1)

MLM	R_{fit}^2	R_{prd}^2	R_{cv}^2	$RMSE_{fit}$	$RMSE_{cv}$	$RMSE_{prd}$
PLS	0.6148	0.4900	0.4956	0.5908	0.6761	0.6689
GP	0.6820	0.5187	0.5390	0.5368	0.6463	0.6498
RF	0.8494	0.4593	0.4387	0.3694	0.7132	0.6888
SVM	0.7462	0.5487	0.5508	0.4795	0.5508	0.6292
LibSVM	0.5233	0.4849	0.4351	0.4658	0.5346	0.4987

Yeast Yhr016c SH3 (1/1)

MLM	R_{fit}^2	R_{prd}^2	R_{cv}^2	$RMSE_{fit}$	$RMSE_{cv}$	$RMSE_{prd}$
PLS	0.4979	0.5127	0.4098	0.6217	0.6741	0.6666
GP	0.6062	0.5680	0.4233	0.5531	0.6664	0.6276
RF	0.8674	0.6407	0.5249	0.3195	0.6048	0.5724
SVM	0.6559	0.5996	0.4510	0.5147	0.6501	0.6042
LibSVM	0.4489	0.5473	0.3790	0.4671	0.5120	0.4796

ST_scale

Human amphiphysin SH3 (1/1)

MLM	R_{fit}^2	R_{prd}^2	R_{cv}^2	$RMSE_{fit}$	$RMSE_{cv}$	$RMSE_{prd}$
PLS	0.6417	0.6309	0.5675	0.5153	0.5675	0.5378
GP	0.9759	0.6620	0.6048	0.1337	0.5412	0.5146
RF	0.8840	0.6330	0.5018	0.2932	0.6076	0.5361
SVM	0.7607	0.5866	0.5991	0.4211	0.5866	0.5691
LibSVM	0.5771	0.5520	0.5323	0.3547	0.3856	0.3731

Yeast amphiphysin SH3 (1/1)

MLM	R_{fit}^2	R_{prd}^2	R_{cv}^2	$RMSE_{fit}$	$RMSE_{cv}$	$RMSE_{prd}$
PLS	0.5975	0.5689	0.5448	0.6029	0.6412	0.5856
GP	0.9779	0.5770	0.5563	0.1413	0.6330	0.5800
RF	0.8760	0.5851	0.5568	0.3347	0.6327	0.5745
SVM	0.8147	0.6335	0.5934	0.4091	0.6060	0.5399
LibSVM	0.5194	0.4836	0.4890	0.4473	0.4730	0.4152

Yeast Boil SH3 (1/1)

MLM	R_{fit}^2	R_{prd}^2	R_{cv}^2	$RMSE_{fit}$	$RMSE_{cv}$	$RMSE_{prd}$
PLS	0.1945	0.0960	0.0658	0.8390	0.9035	0.7295
GP	0.2448	0.1403	0.0861	0.8132	0.8936	0.7114
RF	0.7943	0.1738	0.1472	0.4240	0.8632	0.6974
SVM	0.3088	0.1698	0.1360	0.7772	0.8689	0.6991
LibSVM	0.1605	0.1418	0.0932	0.7698	0.8045	0.5213

Yeast Boi2 SH3 (1/1)

MLM	R_{fit}^2	R_{prd}^2	R_{cv}^2	$RMSE_{fit}$	$RMSE_{cv}$	$RMSE_{prd}$
PLS	0.3101	-0.0483	0.1431	0.4876	0.5437	0.5339
GP	0.1850	0.0799	0.1290	0.5302	0.5482	0.5002
RF	0.7968	0.1765	0.1918	0.2647	0.5280	0.4732
SVM	0.4350	0.0286	0.1508	0.4415	0.5413	0.5139
LibSVM	0.1989	0.0886	0.1226	0.2875	0.3056	0.2488

Yeast endophilin SH3 (1/1)

MLM	R_{fit}^2	R_{prd}^2	R_{cv}^2	$RMSE_{fit}$	$RMSE_{cv}$	$RMSE_{prd}$
PLS	0.4519	0.3623	0.3922	0.7431	0.7825	0.8379
GP	0.4680	0.3708	0.3967	0.7321	0.7796	0.8323
RF	0.8428	0.3796	0.3881	0.3992	0.7851	0.8265
SVM	0.7310	0.4102	0.4581	0.5206	0.7388	0.8058
LibSVM	0.4209	0.3617	0.3893	0.5944	0.6237	0.7247

Yeast Myosin5 SH3 (1/1)

MLM	R_{fit}^2	R_{prd}^2	R_{cv}^2	$RMSE_{fit}$	$RMSE_{cv}$	$RMSE_{prd}$
PLS	0.4381	0.1454	0.3058	0.4498	0.5000	0.4623
GP	0.4759	0.1853	0.3252	0.4344	0.3252	0.4514
RF	0.8269	0.2866	0.3130	0.2496	0.4973	0.4223
SVM	0.6393	0.2684	0.3504	0.3604	0.4837	0.4277
LibSVM	0.3306	0.2449	0.2472	0.2794	0.2965	0.2008

Yeast Rvs167 SH3 (1/1)

MLM	R_{fit}^2	R_{prd}^2	R_{cv}^2	$RMSE_{fit}$	$RMSE_{cv}$	$RMSE_{prd}$
PLS	0.3829	0.1865	0.2280	0.7028	0.7861	0.7783
GP	0.3379	0.1994	0.2279	0.7280	0.7861	0.7721
RF	0.8266	0.4006	0.3465	0.3725	0.7232	0.6681
SVM	0.5148	0.2541	0.2479	0.6231	0.7727	0.7484
LibSVM	0.2300	0.1033	0.1452	0.6866	0.7224	0.6947

Yeast Sho1 SH3 (1/1)

MLM	R_{fit}^2	R_{prd}^2	R_{cv}^2	$RMSE_{fit}$	$RMSE_{cv}$	$RMSE_{prd}$
PLS	0.4009	0.0886	0.1929	0.6253	0.7258	0.6040
GP	0.2788	0.2085	0.1749	0.6861	0.7338	0.5628
RF	0.8535	0.4379	0.4399	0.3092	0.6046	0.4743
SVM	0.6957	0.1558	0.2336	0.4456	0.7072	0.5813
LibSVM	0.1976	0.1745	0.1322	0.5574	0.5862	0.3368

Yeast Yfr024 SH3 (1/1)

MLM	R_{fit}^2	R_{prd}^2	R_{cv}^2	$RMSE_{fit}$	$RMSE_{cv}$	$RMSE_{prd}$
PLS	0.4984	0.4194	0.3703	0.8365	0.9373	0.9684
GP	0.5099	0.4077	0.3814	0.8269	0.9270	0.9782
RF	0.8339	0.4099	0.3489	0.4814	0.9531	0.9763
SVM	0.6157	0.3839	0.4331	0.7323	0.8894	0.9976
LibSVM	0.3734	0.3451	0.3152	0.9540	1.0161	1.1613

Yeast Yhr016c SH3 (1/1)

MLM	R_{fit}^2	R_{prd}^2	R_{cv}^2	$RMSE_{fit}$	$RMSE_{cv}$	$RMSE_{prd}$
PLS	0.4939	0.4618	0.3882	0.6716	0.7384	0.6939
GP	0.5118	0.4775	0.3920	0.6596	0.7361	0.6837
RF	0.8647	0.5493	0.5145	0.3743	0.6578	0.6349
SVM	0.6502	0.5274	0.4330	0.5584	0.7108	0.6502
LibSVM	0.3826	0.4260	0.3263	0.5988	0.6366	0.5861

VSGETAWAY

Human amphiphysin SH3 (1/1)

MLM	R_{fit}^2	R_{prd}^2	R_{cv}^2	$RMSE_{fit}$	$RMSE_{cv}$	$RMSE_{prd}$
PLS	0.7061	0.6405	0.6074	0.4667	0.5394	0.5308
GP	0.7066	0.6411	0.6081	0.4663	0.5389	0.5303
RF	0.8869	0.6348	0.5035	0.2895	0.6065	0.5350
SVM	0.8665	0.6188	0.6429	0.3146	0.5144	0.5465
LibSVM	0.6776	0.6328	0.6298	0.2554	0.2911	0.2932

Yeast amphiphysin SH3 (1/1)

MLM	R_{fit}^2	R_{prd}^2	R_{cv}^2	$RMSE_{fit}$	$RMSE_{cv}$	$RMSE_{prd}$
PLS	0.9010	0.3846	0.8737	0.4079	0.4607	0.5507
GP	0.9010	0.3864	0.8738	0.4079	0.4605	0.5499
RF	0.9288	0.3714	0.2333	0.3460	1.1351	0.5566
SVM	0.7725	-0.0976	0.7417	0.6183	0.6589	0.7355
LibSVM	0.7339	0.4789	0.7103	0.2575	0.2823	0.4708

Yeast Boil SH3 (1/1)

MLM	R_{fit}^2	R_{prd}^2	R_{cv}^2	$RMSE_{fit}$	$RMSE_{cv}$	$RMSE_{prd}$
PLS	0.3729	0.2589	0.1773	0.5196	0.5952	0.6276
GP	0.4071	0.2594	0.2051	0.5053	0.5850	0.6274
RF	0.7966	0.2191	0.1146	0.2960	0.6175	0.6443
SVM	0.4039	-0.0623	0.2257	0.5066	0.5774	0.7515
LibSVM	0.3673	0.2707	0.2289	0.3084	0.3499	0.4804

Yeast Boi2 SH3 (1/1)

MLM	R_{fit}^2	R_{prd}^2	R_{cv}^2	$RMSE_{fit}$	$RMSE_{cv}$	$RMSE_{prd}$
PLS	0.2336	0.0713	0.0609	0.5107	0.5653	0.4985
GP	0.2771	0.0878	0.1483	0.4960	0.5383	0.4941
RF	0.7838	0.0984	0.1028	0.2712	0.5538	0.4900
SVM	0.2473	0.0138	0.1238	0.5061	0.5460	0.5137
LibSVM	0.2605	0.0834	0.1384	0.2671	0.2954	0.2567

Yeast endophilin SH3 (1/1)

MLM	R_{fit}^2	R_{prd}^2	R_{cv}^2	$RMSE_{fit}$	$RMSE_{cv}$	$RMSE_{prd}$
PLS	0.5724	0.2222	0.4800	0.5555	0.6126	0.6653
GP	0.5726	0.2226	0.4802	0.5554	0.6125	0.6651
RF	0.8611	0.3333	0.4556	0.3166	0.6268	0.6160
SVM	0.4913	0.0677	0.4369	0.6059	0.6375	0.7284
LibSVM	0.5618	0.2812	0.5065	0.3188	0.3569	0.4424

Yeast Myosin5 SH3 (1/1)

MLM	R_{fit}^2	R_{prd}^2	R_{cv}^2	$RMSE_{fit}$	$RMSE_{cv}$	$RMSE_{prd}$
PLS	0.4259	0.3638	0.3002	0.4125	0.4554	0.4879
GP	0.4791	0.3969	0.3125	0.3929	0.4514	0.4856
RF	0.8111	0.3028	0.2271	0.2366	0.4786	0.5107
SVM	0.4216	0.3325	0.2813	0.4140	0.4615	0.4997
LibSVM	0.4239	0.2673	0.2935	0.2008	0.2266	0.2979

Yeast Rvs167 SH3 (1/1)

MLM	R_{fit}^2	R_{prd}^2	R_{cv}^2	$RMSE_{fit}$	$RMSE_{cv}$	$RMSE_{prd}$
PLS	0.3929	0.3384	0.2037	0.6057	0.6937	0.6754
GP	0.4178	0.3434	0.2512	0.5922	0.6727	0.6719
RF	0.8242	0.4638	0.1889	0.3260	0.7001	0.6072
SVM	0.3863	0.0577	0.2295	0.6090	0.6824	0.8049
LibSVM	0.3826	0.3093	0.2606	0.4354	0.4831	0.4869

Yeast Sho1 SH3 (1/1)

MLM	R_{fit}^2	R_{prd}^2	R_{cv}^2	$RMSE_{fit}$	$RMSE_{cv}$	$RMSE_{prd}$
PLS	0.4590	-0.3077	0.1549	0.5628	0.1549	0.5010
GP	0.1783	-0.0524	0.1196	0.6936	0.7180	0.4494
RF	0.8613	0.0271	0.4521	0.2850	0.5664	0.4321
SVM	0.3365	-0.0993	0.1960	0.6233	0.6861	0.4593
LibSVM	0.3429	0.0272	0.2155	0.4644	0.5079	0.4473

Yeast Yfr024 SH3 (1/1)

MLM	R_{fit}^2	R_{prd}^2	R_{cv}^2	$RMSE_{fit}$	$RMSE_{cv}$	$RMSE_{prd}$
PLS	0.5698	0.4382	0.4203	0.6206	0.7204	0.6929
GP	0.5887	0.4245	0.4366	0.6068	0.7178	0.6936
RF	0.8407	0.3966	0.3146	0.3777	0.7833	0.7177
SVM	0.5905	0.0992	0.4542	0.6055	0.6990	0.8770
LibSVM	0.5206	0.3501	0.4369	0.4530	0.5191	0.7065

Yeast Yhr016c SH3 (1/1)

MLM	R_{fit}^2	R_{prd}^2	R_{cv}^2	$RMSE_{fit}$	$RMSE_{cv}$	$RMSE_{prd}$
PLS	0.5289	0.3984	0.3784	0.6154	0.7069	0.6230
GP	0.5548	0.3721	0.3959	0.5982	0.6969	0.6364
RF	0.8639	0.5437	0.3976	0.3308	0.6959	0.5425
SVM	0.5407	0.0509	0.4108	0.6076	0.6882	0.7824
LibSVM	0.4959	0.3724	0.4215	0.4465	0.4952	0.7636

VHSE

Human amphiphysin SH3 (1/1)

MLM	R_{fit}^2	R_{prd}^2	R_{cv}^2	$RMSE_{fit}$	$RMSE_{cv}$	$RMSE_{prd}$
PLS	0.5806	0.5316	0.3535	0.3107	0.3675	0.3670
GP	0.5961	0.5461	0.3255	0.2992	0.3797	0.3556
RF	0.9528	0.6718	0.7398	0.0349	0.1206	0.2571
SVM	0.5540	0.4963	0.4889	0.5749	0.6154	0.6283
LibSVM	0.5589	0.5050	0.5125	0.3565	0.3919	0.4015

Yeast amphiphysin SH3 (1/1)

MLM	R_{fit}^2	R_{prd}^2	R_{cv}^2	$RMSE_{fit}$	$RMSE_{cv}$	$RMSE_{prd}$
PLS	0.8641	0.3072	0.8335	0.4765	0.5275	0.5851
GP	0.8668	0.3147	0.8349	0.4719	0.5252	0.5808
RF	0.9470	0.4973	0.2074	0.2975	1.1509	0.4984
SVM	0.9992	0.2277	0.8831	0.0356	0.4421	0.6177
LibSVM	0.7008	0.4255	0.6817	0.2971	0.3144	0.3694

Yeast Boil SH3 (1/1)

MLM	R_{fit}^2	R_{prd}^2	R_{cv}^2	$RMSE_{fit}$	$RMSE_{cv}$	$RMSE_{prd}$
PLS	0.2302	0.1506	0.1017	0.5758	0.6219	0.6719
GP	0.3042	0.1614	0.1009	0.5474	0.6222	0.6677
RF	0.8019	0.2361	0.1461	0.2921	0.6064	0.6372
SVM	0.5710	0.2206	0.2112	0.4298	0.5828	0.6437
LibSVM	0.2575	0.1177	0.1794	0.3439	0.3685	0.7691

Yeast Boi2 SH3 (1/1)

MLM	R_{fit}^2	R_{prd}^2	R_{cv}^2	$RMSE_{fit}$	$RMSE_{cv}$	$RMSE_{prd}$
PLS	0.2736	-0.0416	0.1059	0.4972	0.5516	0.5279
GP	0.2146	0.0277	0.1411	0.5169	0.5406	0.5101
RF	0.7953	0.1049	0.1354	0.2639	0.5424	0.4894
SVM	0.2867	0.0585	0.1507	0.4927	0.5376	0.5019
LibSVM	0.2430	0.0377	0.1505	0.2725	0.2931	0.3251

Yeast endophilin SH3 (1/1)

MLM	R_{fit}^2	R_{prd}^2	R_{cv}^2	$RMSE_{fit}$	$RMSE_{cv}$	$RMSE_{prd}$
PLS	0.8815	0.3684	0.8557	0.4670	0.5152	0.6024
GP	0.8819	0.3700	0.8560	0.4661	0.5148	0.6016
RF	0.9184	0.1952	0.3009	0.3874	1.1341	0.6800
SVM	0.9595	0.4610	0.8857	0.2729	0.4585	0.5565
LibSVM	0.7244	0.3565	0.7084	0.2899	0.3053	0.6506

Yeast Myosin5 SH3 (1/1)

MLM	R_{fit}^2	R_{prd}^2	R_{cv}^2	$RMSE_{fit}$	$RMSE_{cv}$	$RMSE_{prd}$
PLS	0.9116	0.3753	0.8917	0.3424	0.3790	0.4905
GP	0.9158	0.3464	0.8921	0.3341	0.3782	0.5017
RF	0.9538	0.2573	-0.0456	0.2476	1.1776	0.5348
SVM	0.9416	0.1272	0.8842	0.2783	0.3919	0.5797
LibSVM	0.7181	0.0737	0.7040	0.1794	0.1874	0.5208

Yeast Rvs167 SH3 (1/1)

MLM	R_{fit}^2	R_{prd}^2	R_{cv}^2	$RMSE_{fit}$	$RMSE_{cv}$	$RMSE_{prd}$
PLS	0.2473	0.1479	0.0977	0.6744	0.7384	0.7654
GP	0.2580	0.1636	0.1353	0.6696	0.7229	0.7584
RF	0.8381	0.5041	0.2435	0.3128	0.6762	0.5840
SVM	0.2282	0.3015	0.1168	0.6829	0.7306	0.6930
LibSVM	0.2396	0.3038	0.1270	0.5210	0.5603	0.6054

Yeast Sho1 SH3 (1/1)

MLM	R_{fit}^2	R_{prd}^2	R_{cv}^2	$RMSE_{fit}$	$RMSE_{cv}$	$RMSE_{prd}$
PLS	0.3900	-0.3302	0.2200	0.5960	0.6739	0.5033
GP	0.3946	-0.2175	0.2683	0.5937	0.2683	0.4815
RF	0.8724	0.0265	0.4844	0.2726	0.5479	0.4307
SVM	0.2092	-0.4037	0.1571	0.6486	0.7005	0.5170
LibSVM	0.2987	0.0456	0.2197	0.4604	0.4907	0.5698

Yeast Yfr024 SH3 (1/1)

MLM	R_{fit}^2	R_{prd}^2	R_{cv}^2	$RMSE_{fit}$	$RMSE_{cv}$	$RMSE_{prd}$
PLS	0.4876	0.2796	0.3407	0.6773	0.7682	0.7843
GP	0.5066	0.2542	0.3512	0.6646	0.7621	0.7980
RF	0.8476	0.4652	0.3547	0.3694	0.7600	0.6757
SVM	0.7832	-0.7192	0.5160	0.4406	0.6583	1.2116
LibSVM	0.4346	0.4360	0.3546	0.5363	0.5963	1.4898

Yeast Yhr016c SH3 (1/1)

MLM	R_{fit}^2	R_{prd}^2	R_{cv}^2	$RMSE_{fit}$	$RMSE_{cv}$	$RMSE_{prd}$
PLS	0.4554	0.1426	0.3169	0.6615	0.7410	0.7437
GP	0.4544	0.1578	0.3377	0.6623	0.7297	0.7371
RF	0.8719	0.5898	0.4487	0.3209	0.6657	0.5144
SVM	0.8774	-2.4738	0.4840	0.3139	0.6441	1.1969
LibSVM	0.3969	0.3202	0.3398	0.5356	0.5705	1.0873

Table S9. Statistics of the pQSAR modeling of 8 PDZ-binding peptide panels by systematically combining five MLMs and four AADs

MolSurf

Human CALP PDZ (1/1)

MLM	R_{fit}^2	R_{prd}^2	R_{cv}^2	$RMSE_{fit}$	$RMSE_{cv}$	$RMSE_{prd}$
PLS	0.9992	-1.3386	0.9953	0.0604	0.1493	0.1977
GP	0.9998	-1.6076	0.9953	0.0291	0.1482	0.2087
RF	0.9863	-4.1889	0.9819	0.2543	0.2924	0.2944
SVM	0.9980	-146.520	0.9956	0.0962	0.1444	1.5699
LibSVM	0.9950	0.0099	0.9940	0.0181	0.0210	2.4034

Human NHERF1 PDZ (1/2)

MLM	R_{fit}^2	R_{prd}^2	R_{cv}^2	$RMSE_{fit}$	$RMSE_{cv}$	$RMSE_{prd}$
PLS	0.9982	-1.2987	0.9856	0.1021	0.2863	0.3580
GP	0.9989	-1.1895	0.9866	0.0790	0.2758	0.3494
RF	0.9802	-3.2873	0.9705	0.3355	0.4093	0.4890
SVM	0.9999	-2.4003	0.9874	0.0270	0.2674	0.4355
LibSVM	0.9836	0.0171	0.9803	0.0689	0.0810	3.0530

Human NHERF1 PDZ (2/2)

MLM	R_{fit}^2	R_{prd}^2	R_{cv}^2	$RMSE_{fit}$	$RMSE_{cv}$	$RMSE_{prd}$
PLS	0.4456	-0.4254	0.0130	0.1543	0.2059	0.3016
GP	0.6131	-0.4095	-0.0168	0.1289	0.2090	0.2999
RF	0.7467	-0.1427	-0.0028	0.1043	0.2075	0.2700
SVM	0.7923	-0.4674	-0.0349	0.0945	0.2108	0.3060
LibSVM	0.6106	0.0543	0.0531	0.0224	0.0412	0.0898

Human NHERF2 PDZ (1/2)

MLM	R_{fit}^2	R_{prd}^2	R_{cv}^2	$RMSE_{fit}$	$RMSE_{cv}$	$RMSE_{prd}$
PLS	0.9934	-0.4625	0.9886	0.1817	0.2378	0.2887
GP	0.9981	-0.5693	0.9858	0.0983	0.2655	0.2990
RF	0.9872	-0.8454	0.9795	0.2524	0.3197	0.3243
SVM	0.9970	-40.1514	0.9916	0.1219	0.2041	1.5313
LibSVM	0.9872	0.0017	0.9846	0.0466	0.0547	2.5097

Human NHERF2 PDZ (2/2)

MLM	R_{fit}^2	R_{prd}^2	R_{cv}^2	$RMSE_{fit}$	$RMSE_{cv}$	$RMSE_{prd}$
PLS	0.9972	-0.3574	0.9948	0.1258	0.1721	0.2084
GP	0.9998	-0.4338	0.9950	0.0322	0.1695	0.2142
RF	0.9867	-4.9468	0.9811	0.2758	0.3295	0.4363
SVM	0.9986	-97.6511	0.9962	0.0901	0.1477	1.7770
LibSVM	0.9954	0.0160	0.9945	0.0205	0.0238	3.4417

Human SYNA1 PDZ (1/1)

MLM	R_{fit}^2	R_{prd}^2	R_{cv}^2	$RMSE_{fit}$	$RMSE_{cv}$	$RMSE_{prd}$
PLS	0.9545	-0.0968	0.8825	0.2253	0.3619	0.3507
GP	0.9969	-0.5767	0.8881	0.0591	0.3533	0.4205
RF	0.9468	0.9287	0.9029	0.2434	0.3290	0.4651
SVM	0.9673	-3.7634	0.9060	0.1910	0.3237	0.7309
LibSVM	0.7369	0.0242	0.6992	0.1203	0.1356	0.2991

Human PSD95 PDZ (1/1)

MLM	R_{fit}^2	R_{prd}^2	R_{cv}^2	$RMSE_{fit}$	$RMSE_{cv}$	$RMSE_{prd}$
PLS	0.1797	0.1490	0.1358	0.0789	0.0810	0.0820
GP	0.2810	0.1980	0.0783	0.1739	0.0783	0.0796
RF	0.8432	0.4210	0.4398	0.0345	0.0652	0.0676
SVM	0.3257	0.0552	0.0297	0.0715	0.0858	0.0864
LibSVM	0.1773	0.1466	0.1272	0.0069	0.0074	0.0073

ST_scale

Human CALP PDZ (1/1)

MLM	R_{fit}^2	R_{prd}^2	R_{cv}^2	$RMSE_{fit}$	$RMSE_{cv}$	$RMSE_{prd}$
PLS	0.4520	-0.2376	-1.9953	0.1057	0.2472	0.1784
GP	0.7409	-0.2005	-0.0232	0.0727	0.1445	0.1758
RF	0.7429	-0.3356	-0.0180	0.0786	0.1563	0.1338
SVM	0.4789	-0.5698	-0.1721	0.1119	0.1678	0.1451
LibSVM	0.5067	0.0290	0.0151	0.0158	0.0279	0.0172

Human NHERF1 PDZ (1/2)

MLM	R_{fit}^2	R_{prd}^2	R_{cv}^2	$RMSE_{fit}$	$RMSE_{cv}$	$RMSE_{prd}$
PLS	0.4615	-0.0268	-1.2408	0.2223	0.4534	0.2593
GP	0.3806	0.0197	0.0454	0.2384	0.2959	0.2533
RF	0.7229	0.0006	0.0298	0.1594	0.2984	0.2558
SVM	0.8840	-0.0712	-0.0476	0.1032	0.3100	0.2648
LibSVM	0.5975	0.0153	0.0675	0.0541	0.0880	0.0792

Human NHERF1 PDZ (2/2)

MLM	R_{fit}^2	R_{prd}^2	R_{cv}^2	$RMSE_{fit}$	$RMSE_{cv}$	$RMSE_{prd}$
PLS	0.9926	-14.5033	0.9300	0.1969	0.6053	0.7063
GP	0.9914	-17.7330	0.9278	0.2126	0.6149	0.7764
RF	0.9827	-4.8180	-0.3729	0.3014	2.6816	0.4327
SVM	0.9903	-4.9391	0.9432	0.2248	0.5454	0.4372
LibSVM	0.9909	0.1015	0.9869	0.0353	0.0489	3.3910

Human NHERF2 PDZ (1/2)

MLM	R_{fit}^2	R_{prd}^2	R_{cv}^2	$RMSE_{fit}$	$RMSE_{cv}$	$RMSE_{prd}$
PLS	0.4745	-0.3585	-5.4183	0.1912	0.6681	0.2766
GP	0.3599	0.0339	-0.0550	0.2110	0.2592	0.2438
RF	0.7262	-0.0986	0.0325	0.1380	0.2594	0.2488
SVM	0.8180	-0.2407	-0.0724	0.1125	0.2731	0.2644
LibSVM	0.5085	0.0005	0.0864	0.0408	0.0642	0.0696

Human NHERF2 PDZ (2/2)

MLM	R_{fit}^2	R_{prd}^2	R_{cv}^2	$RMSE_{fit}$	$RMSE_{cv}$	$RMSE_{prd}$
PLS	0.9765	-32.8448	0.9331	0.3725	0.6290	0.7925
GP	0.9933	-22.6983	0.9388	0.1997	0.6015	0.6631
RF	0.9933	-22.6983	0.9388	0.1997	0.6015	0.6631
SVM	0.9966	-14.4466	0.9824	0.1419	0.3229	0.5354
LibSVM	0.9948	0.0721	0.9962	0.0190	0.0249	3.8942

Human SYNA1 PDZ (1/1)

MLM	R_{fit}^2	R_{prd}^2	R_{cv}^2	$RMSE_{fit}$	$RMSE_{cv}$	$RMSE_{prd}$
PLS	0.9911	-3.7429	0.7940	0.2038	0.9831	0.7544
GP	0.9832	-3.1907	0.6724	0.2810	1.2397	0.7091
RF	0.9432	-3.4783	-0.2508	0.5160	2.4224	0.7330
SVM	0.9927	-0.9518	0.0751	0.1846	2.0830	0.4839
LibSVM	0.9838	0.1794	0.9770	0.0543	0.0749	3.4597

Human PSD95 PDZ (1/1)

MLM	R_{fit}^2	R_{prd}^2	R_{cv}^2	$RMSE_{fit}$	$RMSE_{cv}$	$RMSE_{prd}$
PLS	0.1564	0.0776	0.1262	0.0808	0.0823	0.0771
GP	0.1864	0.0933	0.1386	0.0794	0.0817	0.0765
RF	0.8546	0.3862	0.4400	0.0336	0.0659	0.0630
SVM	0.1766	-0.0120	0.0606	0.0799	0.0853	0.0808
LibSVM	0.1617	0.0986	0.1215	0.0078	0.0082	0.0072

VSGETAWAY

Human CALP PDZ (1/1)

MLM	R_{fit}^2	R_{prd}^2	R_{cv}^2	$RMSE_{fit}$	$RMSE_{cv}$	$RMSE_{prd}$
PLS	0.5888	-0.0067	-0.9094	0.0966	0.2082	0.1317
GP	0.8606	0.0068	-0.1803	0.0563	0.1637	0.1308
RF	0.7508	-0.2094	-0.3230	0.0752	0.1733	0.1443
SVM	0.6544	-0.1396	-0.0547	0.0886	0.1547	0.1401
LibSVM	0.7218	0.0952	0.0353	0.0114	0.0220	0.0185

Human NHERF1 PDZ (1/2)

MLM	R_{fit}^2	R_{prd}^2	R_{cv}^2	$RMSE_{fit}$	$RMSE_{cv}$	$RMSE_{prd}$
PLS	0.6880	-0.0317	-0.5882	0.1718	0.3876	0.2386
GP	0.5021	0.0563	0.0837	0.2127	0.2944	0.2282
RF	0.7683	0.0680	0.1918	0.1480	0.2765	0.2268
SVM	0.7524	0.0578	0.0752	0.1530	0.2957	0.2280
LibSVM	0.7522	0.0982	0.0891	0.0426	0.0878	0.0512

Human NHERF1 PDZ (2/2)

MLM	R_{fit}^2	R_{prd}^2	R_{cv}^2	$RMSE_{fit}$	$RMSE_{cv}$	$RMSE_{prd}$
PLS	0.5106	0.2355	-1.3922	0.1572	0.3475	0.1892
GP	0.6741	0.0721	-0.1316	0.1283	0.2390	0.2085
RF	0.7230	0.0590	0.0077	0.1183	0.2238	0.2099
SVM	0.6503	0.0002	-0.0581	0.1329	0.2341	0.2164
LibSVM	0.6977	0.2269	0.0000	0.0272	0.0538	0.0464

Human NHERF2 PDZ (1/2)

MLM	R_{fit}^2	R_{prd}^2	R_{cv}^2	$RMSE_{fit}$	$RMSE_{cv}$	$RMSE_{prd}$
PLS	0.5726	-0.2249	-0.6927	0.1653	0.3290	0.2767
GP	0.6633	-0.3173	0.0072	0.1467	0.2519	0.2870
RF	0.7449	-0.1563	-0.0276	0.1277	0.2563	0.2689
SVM	0.6598	-0.5709	0.0824	0.1475	0.2422	0.3134
LibSVM	0.6515	0.0015	0.0496	0.0333	0.0613	0.0966

Human NHERF2 PDZ (2/2)

MLM	R_{fit}^2	R_{prd}^2	R_{cv}^2	$RMSE_{fit}$	$RMSE_{cv}$	$RMSE_{prd}$
PLS	0.5143	-0.0036	-0.1571	0.1186	-0.1571	0.1634
GP	0.7852	-0.2599	-0.0751	0.0788	0.1764	0.1831
RF	0.7239	-0.1594	-0.1963	0.0864	0.1861	0.1756
SVM	0.7143	-0.1805	-0.0526	0.0909	0.1745	0.1772
LibSVM	0.7692	0.0027	0.0105	0.0124	0.0297	0.0284

Human SYNA1 PDZ (1/1)

MLM	R_{fit}^2	R_{prd}^2	R_{cv}^2	$RMSE_{fit}$	$RMSE_{cv}$	$RMSE_{prd}$
PLS	0.5117	-24.3809	-0.2462	1.6663	2.6621	1.9883
GP	0.0204	-13.9456	-0.3561	2.3602	2.7770	1.5258
RF	0.9615	-5.4351	-0.6156	0.4681	3.0311	1.0012
SVM	0.0265	0.1066	-0.2394	2.3529	2.6549	0.3730
LibSVM	0.9775	0.2117	0.9688	0.0895	0.1206	3.4753

Human PSD95 PDZ (1/1)

MLM	R_{fit}^2	R_{prd}^2	R_{cv}^2	$RMSE_{fit}$	$RMSE_{cv}$	$RMSE_{prd}$
PLS	0.1253	0.1113	0.0869	0.0769	0.0786	0.0891
GP	0.1350	0.1140	0.0888	0.0765	0.0785	0.0890
RF	0.8238	0.3944	0.3515	0.0345	0.0662	0.0735
SVM	0.1983	0.0630	-0.0485	0.0736	0.0842	0.0915
LibSVM	0.1543	0.1048	0.0886	0.0078	0.0082	0.0099

VHSE

Human CALP PDZ (1/1)

MLM	R_{fit}^2	R_{prd}^2	R_{cv}^2	$RMSE_{fit}$	$RMSE_{cv}$	$RMSE_{prd}$
PLS	0.9421	-72.5966	0.5991	0.5197	1.3678	1.5538
GP	0.9655	-72.8389	0.7072	0.4012	1.1690	1.5564
RF	0.9769	-7.1528	-0.4185	0.3287	2.5729	0.5172
SVM	0.9835	-4.8166	0.7104	0.2778	1.1625	0.4368
LibSVM	0.9969	0.0254	0.9954	0.0119	0.0165	2.8088

Human NHERF1 PDZ (1/2)

MLM	R_{fit}^2	R_{prd}^2	R_{cv}^2	$RMSE_{fit}$	$RMSE_{cv}$	$RMSE_{prd}$
PLS	0.4905	-0.4614	-2.1738	0.2025	0.5054	0.3719
GP	0.4362	-0.0613	-0.0787	0.2130	0.2946	0.3170
RF	0.6879	-0.1163	0.0936	0.1585	0.2701	0.3251
SVM	0.8855	-0.0870	-0.1656	0.0960	0.3063	0.3208
LibSVM	0.6094	0.0046	0.0075	0.0468	0.0828	0.1002

Human NHERF1 PDZ (2/2)

MLM	R_{fit}^2	R_{prd}^2	R_{cv}^2	$RMSE_{fit}$	$RMSE_{cv}$	$RMSE_{prd}$
PLS	0.3739	-0.0950	-3.9264	0.1840	0.5161	0.1989
GP	0.3597	0.0297	0.0107	0.1861	0.2313	0.1873
RF	0.7453	-0.0231	-0.1308	0.1174	0.2473	0.1923
SVM	0.5058	-0.0537	-0.0369	0.1635	0.2368	0.1951
LibSVM	0.5072	0.0714	0.0043	0.0327	0.0574	0.0348

Human NHERF2 PDZ (1/2)

MLM	R_{fit}^2	R_{prd}^2	R_{cv}^2	$RMSE_{fit}$	$RMSE_{cv}$	$RMSE_{prd}$
PLS	0.5497	-75.0352	-0.0173	1.5029	2.2590	1.6153
GP	0.9214	-281.801	0.3436	0.6280	1.8147	3.1151
RF	0.9682	-20.0771	-0.4184	0.3996	2.6675	0.8504
SVM	0.9983	-0.6858	0.9852	0.0931	0.2721	0.2405
LibSVM	0.9897	0.0354	0.9848	0.0382	0.0541	2.2645

Human NHERF2 PDZ (2/2)

MLM	R_{fit}^2	R_{prd}^2	R_{cv}^2	$RMSE_{fit}$	$RMSE_{cv}$	$RMSE_{prd}$
PLS	0.9999	-216.507	0.8830	0.0282	0.8263	1.6674
GP	0.9911	-185.949	0.8760	0.2284	0.8505	1.5459
RF	0.9760	-45.7933	-0.4257	0.3743	2.8845	0.7734
SVM	0.9986	0.0415	0.9930	0.0898	0.2016	0.1107
LibSVM	0.9965	0.0069	0.9944	0.0167	0.0251	3.1643

Human SYNA1 PDZ (1/1)

MLM	R_{fit}^2	R_{prd}^2	R_{cv}^2	$RMSE_{fit}$	$RMSE_{cv}$	$RMSE_{prd}$
PLS	0.9312	0.2263	0.1077	0.1062	0.3823	0.3276
GP	0.6984	0.3391	0.1953	0.2223	0.3631	0.3028
RF	0.8191	0.3824	0.4005	0.1722	0.3134	0.2928
SVM	0.7242	0.0689	0.1880	0.2126	0.3647	0.3594
LibSVM	0.8778	0.2766	0.1295	0.0814	0.1529	0.1189

Human PSD95 PDZ (1/1)

MLM	R_{fit}^2	R_{prd}^2	R_{cv}^2	$RMSE_{fit}$	$RMSE_{cv}$	$RMSE_{prd}$
PLS	0.1589	0.1102	0.1254	0.0773	0.0789	0.0857
GP	0.1580	0.1135	0.1281	0.0774	0.0787	0.0855
RF	0.8419	0.4640	0.3979	0.0335	0.0654	0.0665
SVM	0.1256	0.0518	0.0366	0.0788	0.0828	0.0884
LibSVM	0.1610	0.1077	0.1227	0.0068	0.0072	0.0081

Table S10. Statistics of the pQSAR modeling of 1 14-3-3-binding peptide panel by systematically combining five MLMs and four AADs

MolSurf

Yeast 14-3-3 (1/1)

MLM	R_{fit}^2	R_{prd}^2	R_{cv}^2	$RMSE_{fit}$	$RMSE_{cv}$	$RMSE_{prd}$
PLS	0. 7938	0. 3072	0. 7613	0. 6224	0. 6696	0. 8095
GP	0. 8338	0. 3770	0. 7884	0. 5587	0. 6305	0. 7677
RF	0. 9267	0. 2747	0. 7414	0. 3711	0. 6970	0. 8283
SVM	0. 8199	0. 3867	0. 3824	0. 3943	0. 7301	0. 7617
LibSVM	0. 4078	0. 3164	0. 2876	0. 6043	0. 6809	0. 7692

ST_scale

Yeast 14-3-3 (1/1)

MLM	R_{fit}^2	R_{prd}^2	R_{cv}^2	$RMSE_{fit}$	$RMSE_{cv}$	$RMSE_{prd}$
PLS	0. 7453	0. 2754	0. 6869	0. 6724	0. 7545	0. 8302
GP	0. 7421	0. 2650	0. 6896	0. 6766	0. 7422	0. 8361
RF	0. 9095	0. 1530	−0. 2998	0. 4007	1. 5188	0. 8976
SVM	0. 8784	−0. 0528	0. 7010	0. 4646	0. 7284	1. 0007
LibSVM	0. 4946	0. 1988	0. 4629	0. 6267	0. 6541	1. 7871

VSGETAWAY

Yeast 14-3-3 (1/1)

MLM	R_{fit}^2	R_{prd}^2	R_{cv}^2	$RMSE_{fit}$	$RMSE_{cv}$	$RMSE_{prd}$
PLS	0. 4213	0. 3698	0. 2820	0. 7082	0. 7888	0. 7712
GP	0. 4203	0. 3724	0. 3006	0. 7087	0. 7785	0. 7696
RF	0. 8064	0. 2550	0. 2354	0. 4095	0. 8140	0. 8385
SVM	0. 6273	0. 3639	0. 3067	0. 5683	0. 7751	0. 7748
LibSVM	0. 4074	0. 3518	0. 2893	0. 5965	0. 6768	0. 7013

VHSE

Yeast 14-3-3 (1/1)

MLM	R_{fit}^2	R_{prd}^2	R_{cv}^2	$RMSE_{fit}$	$RMSE_{cv}$	$RMSE_{prd}$
PLS	0. 4344	0. 3625	0. 3341	0. 7048	0. 7687	0. 7446
GP	0. 4334	0. 3669	0. 3548	0. 7091	0. 7566	0. 7420
RF	0. 8116	0. 3456	0. 2562	0. 4088	0. 8124	0. 7715
SVM	0. 5211	0. 3886	0. 3614	0. 6519	0. 7527	0. 7292
LibSVM	0. 4003	0. 3025	0. 3097	0. 6085	0. 6703	0. 6342
